# Visible-light mediated catalytic asymmetric radical deuteration at non-benzylic positions

**DOI:** 10.1038/s41467-022-32238-8

**Published:** 2022-08-01

**Authors:** Qinglong Shi, Meichen Xu, Rui Chang, Devenderan Ramanathan, Beatriz Peñin, Ignacio Funes-Ardoiz, Juntao Ye

**Affiliations:** 1 Shanghai Key Laboratory for Molecular Engineering of Chiral Drugs, Frontiers Science Center for Transformative Molecules, School of Chemistry and Chemical Engineering, Shanghai Jiao Tong University, Shanghai, 200240 China; 2 Department of Chemistry, Centro de Investigación en Síntesis Química (CISQ), Universidad de La Rioja, Madre de Dios 53, 26006 Logroño, Spain

**Keywords:** Asymmetric catalysis, Synthetic chemistry methodology, Photocatalysis

## Abstract

Site- and enantioselective incorporation of deuterium into organic compounds is of broad interest in organic synthesis, especially within the pharmaceutical industry. While catalytic approaches relying on two-electron reaction manifolds have allowed for stereoselective delivery of a formal deuteride (D^–^) or deuteron (D^+^) at benzylic positions, complementary strategies that make use of one-electron deuterium atom transfer and target non-benzylic positions remain elusive. Here we report a photochemical approach for asymmetric radical deuteration by utilizing readily available peptide- or sugar-derived thiols as the catalyst and inexpensive deuterium oxide as the deuterium source. This metal-free platform enables four types of deuterofunctionalization reactions of exocyclic olefins and allows deuteration at non-benzylic positions with high levels of enantioselectivity and deuterium incorporation. Computational studies reveal that attractive non-covalent interactions are responsible for stereocontrol. We anticipate that our findings will open up new avenues for asymmetric deuteration.

## Abstract (web summary)

Catalytic asymmetric deuterations rely largely on two-electron reaction manifolds and are mostly limited to benzylic positions. Here, a metal-free platform using peptide- or sugar-derived chiral thiols and deuterium oxide allows for asymmetric open-shell deuteration at non-benzylic positions under visible-light irradiation.

## Introduction

**Fig. 1:**
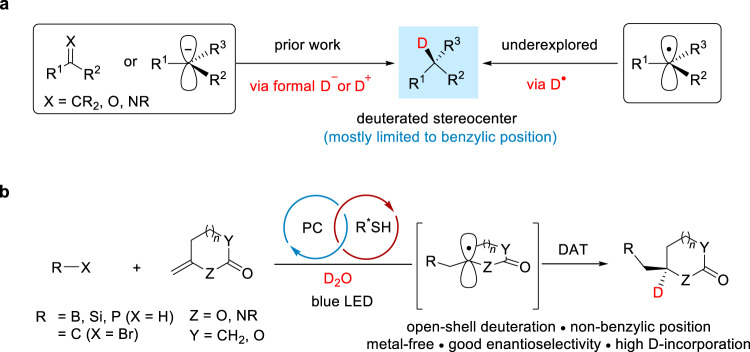
Strategies for the construction of deuterated stereocenters. **a** Catalytic asymmetric deuteration via a closed-shell mechanism involving formal deuteride (D^–^) or deuteron (D^+^) or via an open-shell deuterium atom transfer (DAT) pathway. **b** This work, enantioselective deuterofunctionalization of olefins using a chiral thiol catalyst and deuterium oxide (D_2_O). *PC* photocatalyst, *LED* light-emitting diode.

Deuterium is a stable and non-radioactive isotope of hydrogen and deuterium-labeled compounds are widely used in a broad range of disciplines^1–6^. While some applications need deuterated compounds with high overall deuterium content without considering site- and stereoselectivity, others require deuteration at a distinct position and/or in a stereoselective manner. In the pharmaceutical industry, for example, site- and enantioselective incorporation of deuterium into drug molecules can slow down drug metabolism and potential epimerization of stereocenters, among other benefits, thereby improving drug efficacy^6^. Approval of the first deuterated drug, deutetrabenazine, by the US Food and Drug Administration in 2017 has further spurred the development of novel deuteration methods. While considerable progress has been made on regioselective non-asymmetric deuteration^2–4^, asymmetric deuteration remains underexplored. In this regard, protocols employing enantioenriched starting materials have emerged, allowing for stereoretentive hydrogen isotope exchange^7–11^ and highly diastereoselective deuteration^12,13^. In contrast, catalytic asymmetric deuteration approaches using prochiral or racemic substrates are still limited, mainly due to the challenges associated with identifying a chiral catalyst capable of binding with the commonly used deuterating reagents such as deuterium gas, deuterium oxide, and deuterated solvents. While the use of chiral transition metal complexes^14–17^, enzymes^18–20^, and small-molecule catalysts such as chiral phosphoric acids^21–23^ have met with some success, deuterations are mostly restricted to benzylic positions in these studies. Moreover, from a mechanistic perspective, the deuteration event in the existing approaches typically proceeds through a two-electron manifold where the deuterium atom is introduced to stereocenters as a formal deuteride (D^–^) or deuteron (D^+^) (Fig. 1a), with notable exceptions being disclosed recently in the deuterium labeling experiments of Hyster’s^18,19^ and Jiang’s^23^ work, where a radical deuteration pathway^24^ is operative when a deuterated enzyme cofactor or deuterated Hantzsch ester is utilized as the deuterium source.

Motivated by the growing interest in merging asymmetric organocatalysis with photocatalysis^25–32^ and in view of the paucity of practical methods for asymmetric radical deuterations, we recently questioned whether a photocatalytic radical deuteration could be achieved in a highly enantioselective and cost-effective fashion. In particular, we hypothesized that a combination of chiral thiols with deuterium oxide (D_2_O) might be a potential solution, given the widespread use of achiral thiols as a catalyst for non-asymmetric radical deuteration^4,33–36^ and the encouraging stereocontrol that chiral thiol catalysts exerted in a handful of prior work^37–40^. Additionally, the following features make this strategy promising: 1) uncatalyzed background deuteration—a common issue when carbon anions are involved—would be inhibited as prochiral carbon radicals are virtually unreactive towards D_2_O due to the high bond dissociation energy (BDE) of the O–D bond (119 kcal/mol for HO–H bond)^24,41^, 2) deuterium atom would be covalently bonded to the chiral thiol catalyst through facile in-situ hydrogen/deuterium exchange^4^, thereby enhancing enantiofacial discrimination for the deuteration event. If successful, this strategy would not only introduce a complementary and mechanistically distinct approach to construct deuterated stereocenters, but also enable asymmetric deuteration in a metal-free manner, a feature that would be appealing to the pharmaceutical industry.

Herein, we report a photochemical approach for catalytic, asymmetric radical deuteration at non-benzylic positions in the context of deuteroboration, deuterosilylation, deuterophosphinoylation, and deuterodifluoroalkylation of exocyclic olefins using inexpensive D_2_O and readily available thiol catalysts derived from peptides or sugars (Fig. 1b).

## Results and discussion

### Reaction development

During our investigations on photoinduced hydroalkylation of olefins under the joint catalysis of Lewis base-borane and thiol^42^, we serendipitously observed the addition of *N*-heterocyclic carbene (NHC)–BH_3_ complex^43,44^ **1a** onto olefins. While photoinduced hydroboration of olefins using NHC–BH_3_ complexes have been disclosed recently by several groups^45–49^, asymmetric version of the reaction remains unexplored. We thus chose the reaction of **1a** and exocyclic olefin **2a** as a model reaction to evaluate our hypothesis using chiral thiols that are easily prepared from comercially available chiral sources such as sugars, amino acids, and peptides (Table 1). While Roberts have shown that sugar-derived thiols such as **S1** are competent catalysts for radical hydrosilylation of olefins under thermal conditions, only a single product with high enantiomeric ratio (97.5:2.5 er) was obatained using a sterically very demanding substrate, with low to moderate enantioselectivity for all the other substrates^37^. We started our investigation by using **S1** as the deuterium atom transfer (DAT) catalyst and readily available 4DPAIPN as the organophotocatalyst in a binary solvent mixture of toluene and D_2_O (3:1) at 10 °C. While the desired product **3a** was obatined in 56% yield with 96% D upon isolation, a very low er was observed (entry 1). Other chiral pool-derived thiols such as **S2**–**S4** also provided the product in almost racemic form (entries 2–4). To our delight, when cysteine-derived *β*-turn-containing peptidic thiol **S5**, which was recently developed by Miller and Knowles for the deracemization of ureas^39^, was tested under our conditions, **3a** was obatined in 67% yield with 93:7 er and high levels of deuterium incorporation (94% D) at the α–N position (entry 5). Thiol **S6** with a leucine unit gave same er but lower yield of **3a** while thiol **S7** with a cyclopropane moiety provided slightly lower er (entries 6 and 7). We then examined the influence of D_2_O on the reactivity and enantioselectivity of the reaction. Increasing the amount of D_2_O (toluene:D_2_O = 1:1) significantly lowered the yield of **3a** but increased the deuterium incorporation to 97% (entry 8). In contrast, decreasing the amount of D_2_O (toluene:D_2_O = 4:1) had negnigible influence on the reaction yield but diminished the deuterium incorporation to 90% (entry 9). Interestingly, the enantioselectivity reamined the same under these conditions. However, when the reaction was carried out in the absence of D_2_O, the er dropped to 88:12, although the reaction efficiency was maintained (entry 10). Extending the reaction time to 72 h further improved the yield of **3a** to 73% (entry 11). Importantly, control experiments confirmed that the photocatalyst, visible light, and the thiol are essential for the reaction (entries 12–14).

**Table 1:** Reaction optimization^a^


Entry	R*SH	Solvent	Yield /%^b^	D /%^c^	er^d^
1	**S1**	toluene:D_2_O (3:1)	56	96	48:52
2	**S2**	toluene:D_2_O (3:1)	79	90	51:49
3	**S3**	toluene:D_2_O (3:1)	42	90	53:47
4	**S4**	toluene:D_2_O (3:1)	50	95	58:42
5	**S5**	toluene:D_2_O (3:1)	67	94	93:7
6	**S6**	toluene:D_2_O (3:1)	49	96	93:7
7	**S7**	toluene:D_2_O (3:1)	68	94	92:8
8	**S5**	toluene:D_2_O (1:1)	43	97	93:7
9	**S5**	toluene:D_2_O (4:1)	71	90	93:7
10	**S5**	toluene	73	–	88:12
11^e^	**S5**	toluene:D_2_O (3:1)	73	94	93:7
12^f^	**S5**	toluene:D_2_O (3:1)	N.D.	–	–
13^g^	**S5**	toluene:D_2_O (3:1)	N.D.	–	–
14^h^	**–**	toluene:D_2_O (3:1)	N.D.	–	–


*er* enantiomeric ratio, *N.D.* Not detected.

^a^Unless otherwise noted, all reactions were carried with **1a** (0.2 mmol), **2a** (0.1 mmol), 4DPAIPN (1 mol%), R*SH (15 mol%), toluene (0.75 mL), D_2_O (0.25 mL) under 10 °C for 48 h with irradiation from a 30 W blue LED.

^b^Isolated yield of **3a**.

^c^Determined by ^1^H NMR analysis of the isolated product.

^d^Determined by chiral HPLC analysis.

^e^Reaction time: 72 h.

^f^No photocatalyst.

^g^Without light irradiation.

^h^No thiol catalyst.

### Reaction scope

**Fig. 2:**
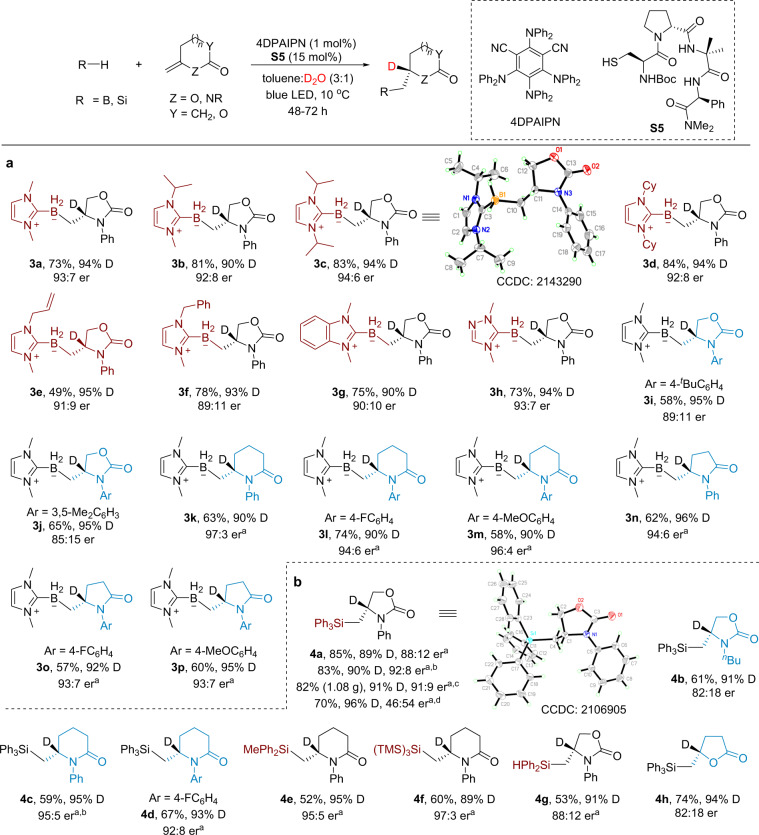
Peptidic thiol-catalyzed enantioselective deuterofunctionalization of exocyclic olefins. **a** Enantioselective deuteroboration. **b** Enantioselective deuterosilylation. Yield, deuterium incorporation, and er are for isolated products; see Supplementary Note 2.3 for experimental details. ^a^Reaction was conducted at rt. ^b^**S7** was used instead of **S5**. ^c^Gram-scale reaction with 4DPAIPN (0.5 mol%) and **S7** (10 mol%). ^d^**S1** was used instead of **S5**.

With the optimized conditions in hand, the scope and limitations of the deuteroboration reaction was explored (Fig. 2a). NHC boranes with various substituents on nitrogen and 1,2,4-triazol-5-ylidene borane all underwent the reaction smoothly, providing the desired products **3a**–**3h** in good yields with high levels of deuterium incorporation and with er ranging from 89:11 to 94:6. Other Lewis base-borane complexes such as Ph_3_P–BH_3_ and DMAP–BH_3_ were evaluated but no reactivity was observed under the current conditions. In addition to 2-oxazolidinone-based olefins, exocyclic olefins on 2-piperidinones and 2-pyrrolidinones are also viable substrates, furnishing the corresponding products **3i**–**3p** in 57-74% yield with er up to 97:3. Interestingly, high resolution mass spectra (HRMS) analysis indicated that the BH_2_ moieties in all these products were also partially deuterated. We also briefly examined the deuterosilylation reactions given that Roberts’ early studies on the hydrosilylation reaction under thermal conditions mostly gave low to moderate enantioselectivities^37^. Under room temperature, the reaction of triphenylsilane with **2a** in the presence of **S5** afforded the desired product **4a** in 85% yield with 89% D and 88:12 er (Fig. 2b). The er was improved to 92:8 upon using **S7** as the thiol catalyst. To demonstrate the practicality of the reaction, we scaled up the reaction with lower catalyst loadings and 1.08 gram of **4a** was obtained with comparable results as that of the small scale reaction. By comparison, the reaction using Roberts’ optimal thiol **S1** under otherwise identical conditions provided **4a** in 70% yield with 96% D but with very low enantioselectivity (46:54 er). Replacing the phenyl group on nitrogen with *n*-butyl group produced **4b** with a decreased er (82:18) while the use of 2-piperidinone-based olefins afforded **4c** and **4d** in moderate yields with good enantioselectivities. Other silanes such as diphenylmethylsilane, tris(trimethylsilyl)silane, and diphenylmethylsilane were also tolerated (**4e**–**4g**). γ-Lactone-based olefin afforded the product **4h** in good yield with modest levels of enantioselectivity. 1,1-Disubstituted olefins in acyclic systems were tested but typically gave the corresponding products in racemic form (Supplementary Fig. 1), in line with the long-standing challenge of controlling the stereoselectivity of acyclic radicals^50^.The absolute configurations of the deuterated stereocenters were determined by X-ray crystallographic analysis to be *R* and *S* for products **3c** and **4a**, respectively. At the end of the reactions, the D_2_O can be recovered using a separatory funnel if desired. When the recycled D_2_O was subjected to the standard conditions for the synthesis of **4a**, the deuterium content of **4a** dropped from 90% to ~80%, although the yield and enantioselectivity were similar to those of the standard conditions (Supplementary Fig. 2). These observations suggest that partial H/D exchange occurred for the D_2_O during the reaction, leading to a decreased deuterium content for the recycled D_2_O.

**Fig. 3:**
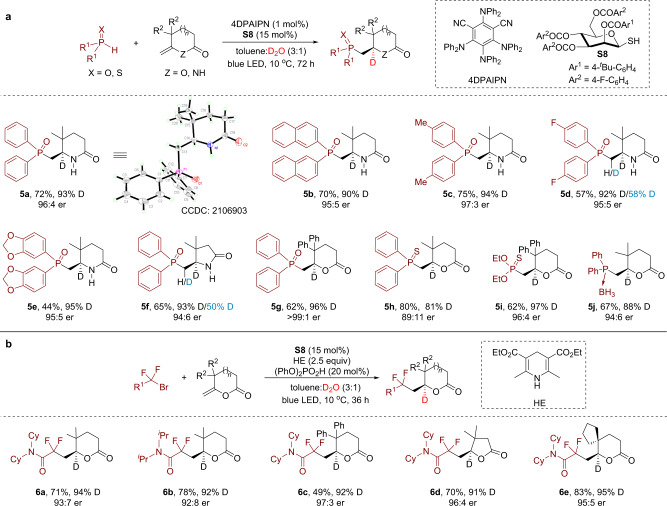
Sugar-derived thiol-catalyzed enantioselective deuterofunctionalization of exocyclic olefins. **a** Enantioselective deuterophosphinoylation. **b** Enantioselective deuterodifluoroalkylation. See Supplementary Note 2.3 for experimental details.

Next, we turned our attention to explore the applicability of this deuteration strategy in other photocatalytic systems. Given the versatile utilities of organophosphorous compounds and the similar bond strength of P–H bond (BDE = 79 kcal/mol for diphenylphosphine oxide)^51^ and B–H bond of NHC–boranes (BDE = 74–80 kcal/mol)^43^, we hypothesized that phosphorous-centered radicals might be formed in a similar fashion and engage in alkene deuterophosphinoylation. While inital trials with peptidic thiol catalysts proved fruitless, we were pleased to find that highly enantioselective deuterophosphinoylation could be achieved using a new *β*-mannose-derived thiol catalyst **S8** (Fig. 3a). Using diarylphosphine oxides as the phosphinoyl radical precursor, various methylenelactams and methylenelactones were well tolerated to deliver the desired products **5a**–**5g** in 44–75% yields with good levels of enantioselectivity (94:6–>99:1 er). The structure and absolute configuration of **5a** was determined to be *R* using single-crystal X-ray diffraction. Interestingly, for products **5d** and **5f**, partial deuteration at the α-P position was also observed. Other phosphorous-centered radical precursors such as diphenylphosphine sulfide and diethyl thiophosphite are also compatible (**5h** and **5i**). Of particular note is that diphenylphosphine borane complex is also a suitable substrate for this transformation, as exemplified by the synthesis of **5j**. For deuterophosphinoylation and deuterodifluoroalkylation reactions, the substitutes at the allylic positions were found to be very important as much lower conversions and enantioselectivities were observed using an olefin devoid of such substituents (Supplementary Fig. 3). The reason behind this observation remains unknown and is currently under investigation.

Having demonstrated the generality of this enantioselective deuterofunctionalization strategy for introducing heteroatoms to olefins, we sought to extend it to alkene deuteroalkylation. We chose α-bromodifluoroacetamides as alkyl radical precursors given their widespread applications in radical chemistry and the importance of difluoroalkylated compounds^52^. To our delight, using thiol **S8** as the DAT catalyst and Hantzsch ester (HE) as an electron donor, photoreductive deuterodifluoroalkylation reactions ocurred smoothly to furnish *gem*-difluoro- and deuterium-containing products **6a**−**6e** in modest to good yields with high levels of enantioselectivity and deuterium incorporation (Fig. 3b).

### Product derivatization

**Fig. 4:**
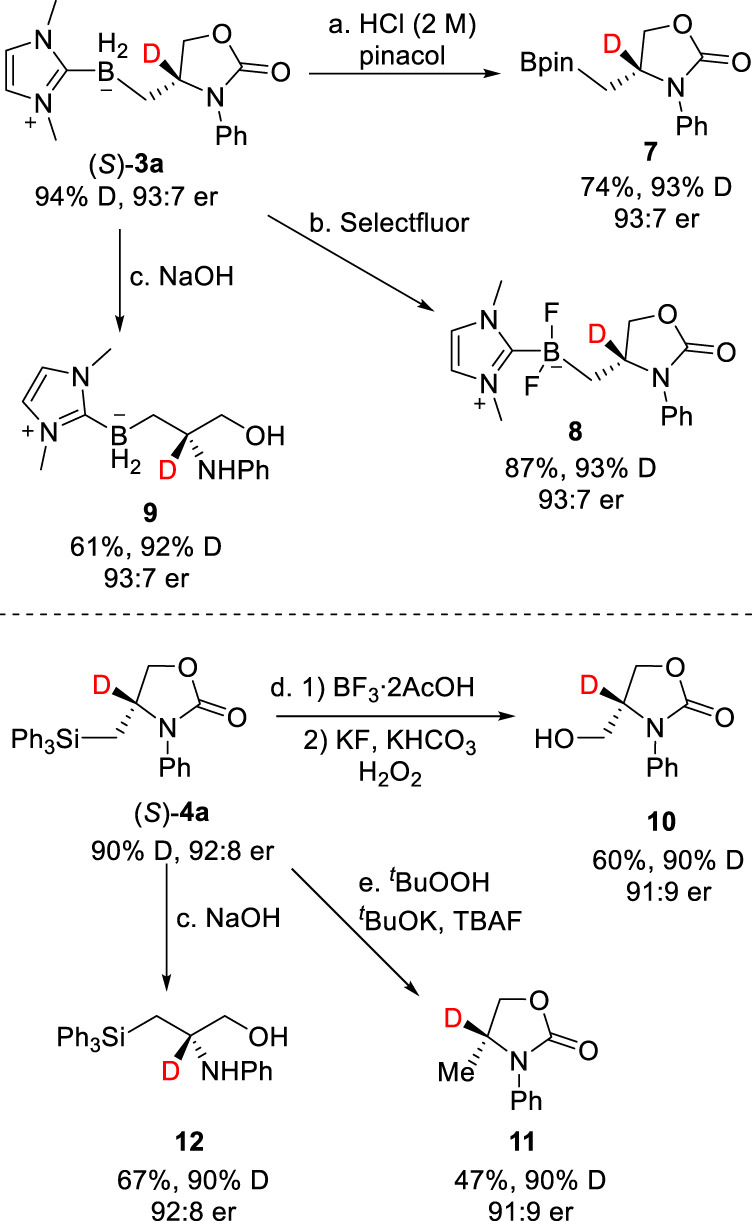
Product derivatization. See Supplementary Note 2.4 for experimental details.

The deuterated products obtained in this study can be easily elaborated to provide versatile chiral building blocks without erosion of enantiopurity and deuterium content (Fig. 4). For example, NHC-borane **3a** was treated with 2M HCl and pinacol to provide synthetically useful alkyl pinacol boronic ester **7**^46,49^. In addition, **3a** could be transformed into difluoroborane **8** using Curran’s approach^53^. Hydrolysis under basic conditions afforded valuable α-deuterated 1,2-amino alcohol derivative **9**. For 2-oxazolidinone **4a**, manipulation of the C–Si bond under oxidative conditions^54^ provides synthetically useful 4-hydroxymethyl-substituted oxazolidinone **10** or 4-methyl-substituted oxazolidinone **11** while hydrolysis afforded silicon-containing 1,2-amino alcohol derivative **12**.

### Mechanistic studies

**Fig. 5:**
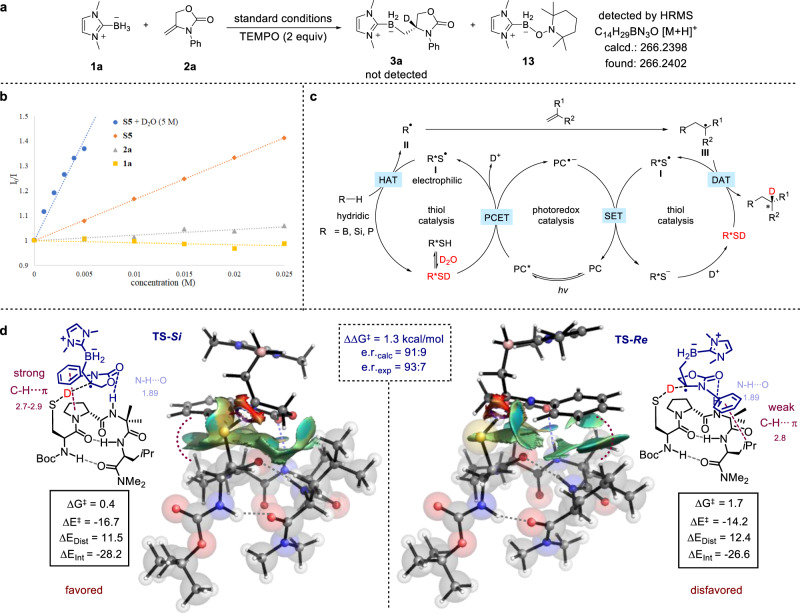
Experimental and computational studies of the mechanistic aspects of the reaction. **a** Radical trap experiment with TEMPO. **b** Stern−Volmer Plot. **c** Proposed catalytic cycle. **d** DFT calculated *Re* and *Si* transition states, including non-covalent interactions (NCI), and distortion/interaction analyses. Energies are in kcal/mol and distances are in Å. See Supplementary Note 2.11 for full details. TEMPO = 2,2,6,6-tetramethyl-1-piperidinyloxy.

Subsequently, preliminary mechanistic studies were carried out to shed some light on the mechanism of the reactions. We focused on the deuteroboration reaction of **1a** and **2a** catalyzed by thiol **S5**. First, addition of 2,2,6,6-tetramethylpiperidine-1-oxyl (TEMPO) to the reaction mixture completely inhibited the reaction with radical adduct **13** being detected by HRMS (Fig. 5a), suggesting a radical-based pathway. Stern−Volmer quenching experiments revealed that the excited state of the photoredox catalyst 4DPAIPN [E_1/2_(PC^*^/PC^•−^) = +0.90 V vs SCE]^55^ could be quenched by the peptidic thiol catalyst **S5** (E_p/2_ = +1.41 V versus SCE) but not by the NHC–borane **1a** (E_p/2_ = +0.76 V versus SCE)^49^, although reductive quenching by **1a** is thermodynamically more favorable (Fig. 5b). Intrigued by these observations, we further investigated the influence of water on redox potentials and the quenching rate. While the oxidation potential of **S5** and the reduction potential of 4DPAIPN were largely unchanged in the presence of water (Supplementary Figs. 13 and 14), the quenching rate of thiol increased significantly when D_2_O was present (blue line in Fig. 5b), suggesting that D_2_O is not merely a deuterium source in the reaction. As concerted proton−coupled electron transfer (PCET) with water being the the proton acceptor is a very common process in biological systems^56–58^, we attributed the increased quenching rate of thiol in the presence of D_2_O to a concerted oxidative PCET process where water is the proton acceptor. In line with this proposal, we observed that the reaction of **1a** and **2a** was much faster in the presence of D_2_O than in its absence (Supplementary Table 7). Finally, the quantum yield of the reaction of **1a** and **2a** was determined to be 0.76%, indicating that a radical chain-based mechanism is unlikely. While further mechanistic investigations for the other three types of reactions are currently underway in our laboratory, a plausible mechanism was proposed based on prior work^44,46,59^ and our experimental observations (Fig. 5c). Photoexcitation of the photocatalyst (PC) with visible light would produce the excited stated PC^*^, which oxidizes a thiol catalyst to generate an electrophilic thiyl radical **I** via a PCET process. A polarity-matched hydrogen atom transfer (HAT) event then occurs between the thiyl radical and a hydridic R–H (R=B, Si or P) bond of the substrate^60,61^. Subsequent radical addition to the olefin furnishes a prochiral and nucleophilic carbon-centered radical **III**, which undergoes polarity-matched and stereoselective DAT with the in-situ generated deuterated chiral thiol (R^*^SD) to deliver the desired deuterated product and regenerate the thiyl radical. Finally, single-electron reduction of the thiyl radical by the reduced state of the photocatalyst (PC^•–^) would regenerate the ground-state photocatalyst and the deuterated thiol after protonation.

### Computational studies

To elucidate the origin of enantioselectivity for the DAT step, we performed density functional theory (DFT) calculations at the CPCM(Toluene) PBE0/6-311++G(3d,2p)//ωB97xD/6-31+G(d,p) level of theory using thiol **S6** as the catalyst for the reaction of **1a** and **2a** (Fig. 5d, see Supplementary Information for computational details). After conformation analysis of the peptide catalyst based on Miller’s pioneering studies on *β*-turn-containing tetrapeptides^62,63^, it was found that the approach of the radical adduct in the transition state (TS) is dictated by the C=O**···**H–N interaction in the backbone (highlighted with blue dash lines). Moreover, the *Si* and *Re* faces of the carbon radical interact differently with the peptidic thiol in the transition states due to non-covalent dispersion interactions^64,65^, with **TS-*Si*** displaying strong C–H**···**π interactions between the proline and the phenyl ring of the radical adduct. In contrast, only weak C−H**···**π interactions are identified in **TS-*Re***. As depicted in the NCI plot and quantified in the distortion/interaction analysis^66^, the interaction between the radical adduct and the thiol catalyst is stronger by 1.6 kcal/mol in **TS-*Si***. In addition, the overall activation energy difference considering entropic contributions is calculated to be 1.3 kcal/mol (ΔΔG^≠^), corresponding to a theoretical er of 91:9 at 10 °C in favor of *R* enantiomer, which is in close agreement with the experimentally observed sense and magnitude of enantioinduction (93:7 er).

In summary, by merging organocatalysis with photoredox catalysis, highly enantioselective radical deuteration at non-benzylic positions has been achieved using peptide- or sugar-derived thiol catalysts and D_2_O. This metal-free approach is uniformly effective for deuteroboration, deuterosilylation, deuterophosphinoylation, and deuterodifluoroalkylation of exocyclic olefins. We anticipate that this catalytic asymmetric deuteration strategy will be applicable to other radical reactions terminated with a hydrogen atom transfer event and will be of guiding significance and practical utility.

## Methods

### General procedure for the deuteroboration of olefins

To an oven-dried 16 × 60 mm vial containing a dry Teflon stir bar were charged with 4DPAIPN (0.8 mg, 0.001 mmol), thiol catalyst **S5** (9.0 mg, 0.015 mmol), and NHC–BH_3_ **1a** (22.2 mg, 0.2 mmol). After sequential addition of dry toluene (0.75 mL), D_2_O (0.25 mL), and olefin **2a** (17.5 mg, 0.1 mmol), the reaction mixture was flushed with nitrogen gas for two minutes and then the vial was sealed with a cap and parafilm. The vial was placed in a cooling station and a 30 W blue LED (λ_max_ = 441 nm) was then placed at the top of the cooling station, which is connected to a chiller to maintain the temperature of the cooling water at 10 °C. The reaction mixture was stirred at 10 °C under irradiation with a stirring rate of 400 r/min for 72 h. When the reaction is complete as monitored by thin layer chromatography and gas chromatography–mass spectrometry, CH_2_Cl_2_ (10 mL) and H_2_O (5 mL) were added, the organic layer was separated and the aqueous layer was extracted with CH_2_Cl_2_ (10 mL ×3). The combined organic layer was washed with brine and dried over anhydrous Na_2_SO_4_. After filtration and evaporation, the residue was purified by chromatography on silica gel to afford the desired product.

## Supplementary information

Supplementary Information

Description of Additional Supplementary Files

Supplementary Data 1

## Data Availability

All data generated or analyzed during this study are included in this Article and the Supplementary Information and Supplementary Data files. Details about materials and methods, experimental procedures, mechanistic studies, characterization data, computational details, NMR and HPLC spectra are available in the Supplementary Information. Calculated coordinates are available in the Supplementary Data file. Crystallographic data for compounds **3c**, **4a**, and **5a** are available free of charge from the Cambridge Crystallographic Data Centre (CCDC) under reference number 2143290, 2106905, and 2106903, respectively (https://www.ccdc.cam.ac.uk/structures).
