# Supplementary material for: Visible-light mediated catalytic asymmetric radical deuteration at non-benzylic positions
Source: Nat Commun. 2022 Aug 1;13:4453. doi: 10.1038/s41467-022-32238-8 (PMC9343372; doi:10.1038/s41467-022-32238-8)
Supplement: Supplementary file 1 — Supplementary Information [file 41467_2022_32238_MOESM1_ESM.pdf]

SUPPLEMENTARY INFORMATION

Visible-Light Mediated Catalytic Asymmetric Radical Deuteration at Non-Benzylic Positions

Qinglong Shi,[1,ǂ] Meichen Xu,[1,ǂ] Rui Chang,[1] Devenderan Ramanathan,[1] Beatriz Peñín,[2] Ignacio Funes-Ardoiz,[2]* and Juntao Ye[1]*

[1]Shanghai Key Laboratory for Molecular Engineering of Chiral Drugs, Frontiers Science Center for Transformative Molecules, School of Chemistry and Chemical Engineering, Shanghai Jiao Tong University, Shanghai 200240, China.

[2]Department of Chemistry, Centro de Investigación en Síntesis Química (CISQ), Universidad de La Rioja, Madre de Dios 53, 26006 Logroño, Spain.

ǂ These authors contributed equally to this work.

*Corresponding author. Email: juntaoye@sjtu.edu.cn (J.Y.); ignacio.funesa@unirioja.es (I.F.A.)

Table of Contents

1. Supplementary Methods……………………………........………………………………………3

1.1 General Information.….……………………………........……………………………………3

2. Supplementary Notes………………………………........……………………………………5

2.1 Synthesis of Catalysts and Substrates……………........……………………………………5

2.2 Optimization Studies.….………………………........……………………………………12

2.3 Synthesis and Characterization of Products……........……………………………………15

2.4 Synthetic Applications.…………………………........……………………………………47

2.5 Radical Trap Experiment........…………………………………………………………51

2.6 UV-Vis Absorption Spectra of **1a**, **2a**, and **S5**..…......…...………………………………52

2.7 Cyclic Voltammetry Measurements……………........……………………………………54

2.8 Stern-Volmer Experiments....………………........……………………………………56

2.9 Quantum Yield Measurements………………........………………………………………62

2.10 X-Ray Crystallographic Data of Compounds **3c**, **4a**, and **5a**……....……………………68

2.11 Computational Details ....………………....……………………………………72

2.12 NMR Spectra and HPLC Spectra……………………...………………………………74

3. Supplementary References ....……………....……………………………………210

1. Supplementary Methods

1.1. General Information

Unless otherwise stated all reactions were set up under nitrogen atmosphere utilizing oven-dried glassware. Solvents were either purchased from Adamas or J&K (Sure/Seal bottles) or dried with activated 4 Å molecular sieves and stored in a glovebox under nitrogen atmosphere. Column chromatography was performed using silica gel (200-300 mesh). All other reagents were purchased from various commercial sources and used as received. A blue LED (λ_{max} = 441 nm, 30 W) was used as the light source for all the photo-reactions. Reactions were monitored by thin-layer chromatography on Leyan silica gel plates (60 GF254) which were rendered visible by ultraviolet light and/or spraying with basic $KMnO_4$ solution, followed by heating.

^{1}H NMR spectra were recorded on a Bruker AVIII 400 (400 MHz) spectrometer and are reported in ppm, relative to tetramethylsilane (TMS, δ 0 ppm) or residual solvent signals ($CDCl_3$ referenced at δ 7.26 ppm, CD_3OD referenced at δ 3.31 ppm). Data are reported as follows: (brs = broad singlet, s = singlet, d = doublet, t = triplet, q = quartet, m = multiplet; coupling constant(s) in Hz; integration). ^{13}C NMR spectra were recorded on a Bruker AVIII 400 (100 MHz) spectrometer and are reported in ppm, relative to residual solvent signals ($CDCl_3$ referenced at δ 77.0 ppm, CD_3OD referenced at δ 49.0 ppm). Note: due to quadrupole broadening and spin–spin coupling with boron, resonances of hydrogen atoms bonded to the boron atom are weak (sometimes absent due to partial deuteration) and are thus not integrated in ^{1}H NMR spectra. In addition, the two carbon atoms attached to the boron atom are not detected in the ^{13}C NMR spectra for the same reason. High resolution mass spectra (HRMS) were performed at Instrumental Analysis Center of Shanghai Jiao Tong University with electrospray spectrometer Waters Micromass Q-TOF Premier Mass Spectrometer. Enantiomeric ratios (e.r.) were determined by chiral High Performance Liquid Chromatography (HPLC) analysis. UV detection was monitored at 214 nm at the same time. HPLC samples were dissolved in HPLC grade isopropanol (IPA) and hexane unless otherwise stated. HPLC analysis on chiral stationary phase was performed on a Shimadzu LC-2030 Plus-series instrument. Chiralpak AD-H, OD-H, OJ-H, IC-H, or AS-H columns were used with hexane and IPA being the eluents. Gas chromatography-mass spectrometry (GC-MS) analysis were performed on a Shimadzu GCMS-QP2020NX system. Liquid chromatography-mass spectrometry (LC-MS) analysis were performed on a Shimadzu

LCMS 2020 system. Melting points were measured with microscope WRX-4 (Shanghai Yice). Optical rotations were measured on Anton Paar MCP100 automatic polarimeter using a 100 mm path-length cell at 589 nm and reported as follows; $[\alpha]_{\lambda}^{T\ ℃}$ (c = g/100 mL, solvent).

2. Supplementary Notes

2.1. Synthesis of Catalysts and Substrates

Note 1: All the thiols used in this study were synthesized from commercially available amino acids/peptides or sugars in 2-4 steps.

Note 2: The cost of D_2O is 2.72 \$/g from Aldrich and 0.63 \$/g from Bide, a domestic chemical supplier. For comparison, the costs of other commonly used deuterated sources from Aldrich are as follows: CD_3OD—13.31 \$/g; d_8-^{i}PrOH—14.93 \$/g; D_2—149.1 \$/L (from Energy Chemical); $NaBD_4$—140.98 \$/g; $LiAlD_4$—1000.59 \$/g (prices accessed on June 13, 2022).

The photocatalyst 4CzIPN[1] and 4DPAIPN,[2] **S2**,[3] **S3**,[4,5] **S4-S7**,[6,7] **S9-S14**,[6,7] **S1** and **S8**,[8-10] olefins,[11-15] and *N*-heterocyclic carbene (NHC)–BH_3 complexes[16,17] were synthesized according to the reported procedures.

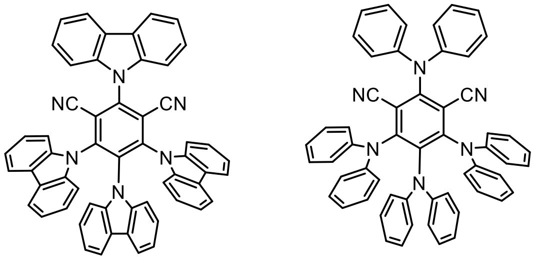

4CzIPN 4DPAIPN

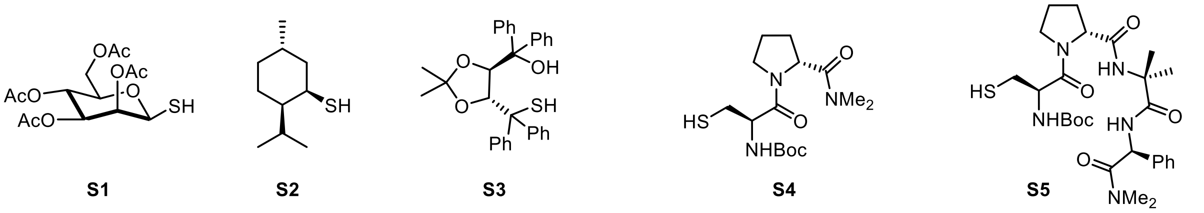

S1 **S2** **S3** **S4** **S5**

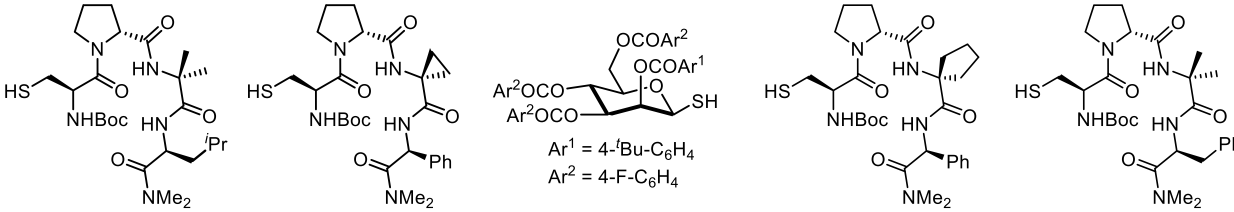

S6 **S7** **S8** **S9** **S10**

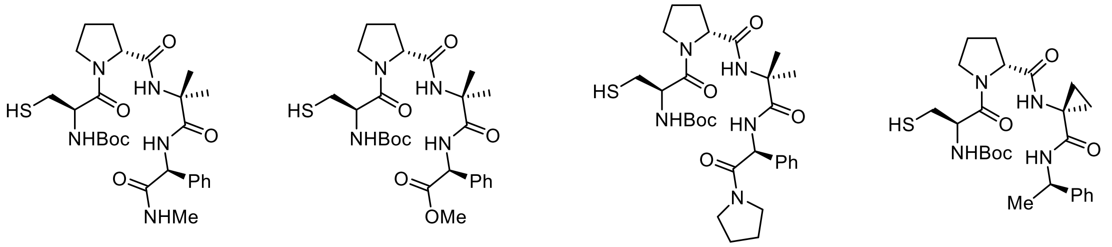

S11 **S12** **S13** **S14**

The thiol catalyst **S4-S6** and **S9-S14** were synthesized as follows:[6,7]

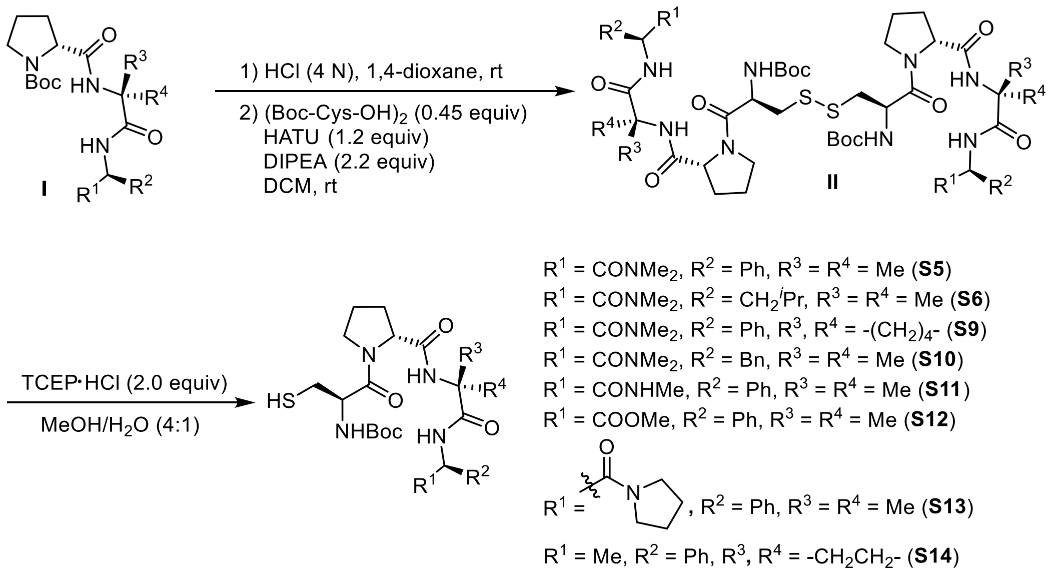

Typical procedure for the synthesis of S9: In a 250 mL round-bottomed flask equipped with a stir bar, **I**[6] (4.26 g, 8.7 mmol) was treated with HCl (4 N in 1,4-dioxane, 22.5 mL) and the reaction mixture was stirred at room temperature for 1 h. The excess HCl was removed under a stream of nitrogen atmosphere and the reaction mixture was concentrated in vacuo to give a foamy white solid. The residue was dissolved in CH_2Cl_2 (40 mL) and $^{i}Pr_2NEt$ (3.23 mL, 19 mmol) was added. $(Boc\text{-}Cys\text{-}OH)_2$ (1.84 g, 4.18 mmol) was added, followed by O-(7-Azabenzotriazol-1-yl)-*N,N,N',N'*-tetramethyluronium hexafluorophosphate (HATU) (3.99 g, 10.4 mmol), and the reaction mixture was stirred at room temperature for 12 h. The reaction mixture was transferred to a separatory funnel, diluted with EtOAc (200 mL), and washed with aqueous citric acid (2 × 200 mL), saturated aqueous $NaHCO_3$ (1 × 200 mL) and brine (1 × 200 mL). The organic phases were then dried over Na_2SO_4, filtered, and concentrated in vacuo to afford the crude product **II**, which was used for the next step without further purification.

In a 100 mL round-bottomed flask equipped with a stir bar, **II** and tris(2-carboxyethyl)phosphine hydrochloride (TCEP•HCl, 1.8 g, 6.2 mmol) were added and then dissolved in $MeOH/H_2O$ (40 mL, v/v = 4/1). The reaction mixture was stirred at room temperature for 2 h, then the reaction was quenched with H_2O, extracted with CH_2Cl_2 (3 × 20 mL), and dried over Na_2SO_4. After evaporation, the residue was purified by flash column chromatography (PE/EA = 1/2) to give **S9** as a foamy white solid (1.53 g, 43% yield over 2 steps): $[\alpha]_D^{25}$ = -27.2 (*c* = 0.36, $CHCl_3$); **m.p.** 174-176 ℃; **^{1}H NMR** (400 MHz, $CDCl_3$) δ = 7.83

(d, J = 8.0 Hz, 1 H), 7.47-7.36 (m, 2 H), 7.35-7.22 (m, 3 H), 7.16 (d, J = 8.8 Hz, 1 H), 6.94 (brs, 1 H), 5.94 (d, J = 8.0 Hz, 1 H), 4.72-4.46 (m, 1 H), 4.44-4.25 (m, 1 H), 3.80-3.54 (m, 2 H), 3.06-2.85 (m, 7 H), 2.76-2.63 (m, 1 H), 2.53-2.40 (m, 1 H), 2.33-1.51 (m, 12 H), 1.42 (s, 9 H); **^{13}C NMR** (100 MHz, $CDCl_3$) δ = 172.7, 170.8, 170.1, 169.7, 155.6, 137.5, 128.6, 127.8, 127.7, 79.6, 66.9, 66.7, 55.0, 53.4, 47.3, 39.6, 37.0, 36.1, 35.3, 28.2, 27.8, 25.9, 25.2, 24.7, 24.3; **HRMS** (ESI) calcd for $C_{29}H_{44}N_5O_6S$ [M+H$^+$]: 590.3007, found: 590.3014.

S4-S6 and **S10-S14** were prepared similarly according to the above procedures.

S4, (R)-*tert*-butyl-2-(dimethylcarbamoyl)pyrrolidine-1-carboxylate was used instead of **I** following the above procedure. Colorless oil: $[\alpha]_D^{25}$ = 16.3 (c = 0.25, $CHCl_3$); **^{1}H NMR** (400 MHz, $CDCl_3$) δ = 5.55 (d, J = 8.0 Hz, 0.74 H), 5.38 (d, J = 9.6 Hz, 0.16 H), 5.11-5.00 (m, 0.14 H), 4.88-4.78 (m, 0.85 H), 4.70-4.55 (m, 0.74 H), 4.18-4.07 (m, 0.21 H), 3.88-3.57 (m, 2 H), 3.18-3.08 (m, 3 H), 3.04-2.95 (m, 3 H), 2.94-2.84 (m, 0.84 H), 2.84-2.65 (m, 1.20 H), 2.27-2.11 (m, 1.70 H), 2.04-1.87 (m, 2.30 H), 1.58 (t, J = 8.8 Hz, 1 H), 1.44 (s, 9 H); **^{13}C NMR** (100 MHz, $CDCl_3$) δ = 171.1, 168.1, 155.0, 79.8, 57.3, 56.7, 54.1, 53.9, 47.3, 46.8, 36.9, 36.1, 35.8, 30.7, 28.5, 28.1, 26.7, 26.5, 24.5, 22.3; **HRMS** (ESI) calcd for $C_{15}H_{27}N_3NaO_4S$ [M+Na$^+$]: 368.1614, found: 368.1617.

S5, white solid: $[\alpha]_D^{25}$ = 34.6 (c = 0.21, $CHCl_3$); **m.p.** 178-181 ℃; **^{1}H NMR** (400 MHz, $CDCl_3$) δ = 7.74 (d, J = 8.0 Hz, 1 H), 7.44-7.23 (m, 5 H), 7.11-6.80 (m, 2 H), 5.90 (d, J = 7.6 Hz, 1 H), 4.63-4.46 (m, 1 H), 4.40-4.28 (m, 1 H), 3.75-3.59 (m, 2 H), 3.03-2.85 (m, 7 H), 2.76-2.65 (m, 1 H), 2.28-1.81 (m, 4 H), 1.64 (t, J = 8.8 Hz, 1 H), 1.55 (s, 3 H), 1.43 (s, 9 H), 1.33 (s, 3 H); **^{13}C NMR** (100 MHz, $CDCl_3$) δ = 173.0, 170.6, 169.9, 169.6, 155.6, 137.3, 128.6, 127.9, 127.6, 79.6, 60.7, 57.0, 55.0, 53.3, 47.3, 36.9, 36.1, 28.2, 27.9, 27.2, 25.8, 25.0, 23.3; **HRMS** (ESI) calcd for $C_{27}H_{42}N_5O_6S$ [M+H$^+$]: 564.2850, found: 564.2847.

S6, white solid: $[\alpha]_D^{25}$ = -61.4 (c = 0.36, $CHCl_3$); **m.p.** 187-188 ℃; **^{1}H NMR** (400 MHz, $CDCl_3$) δ = 7.31-7.17 (m, 2 H), 6.65 (s, 1 H), 5.03-4.91 (m, 1 H), 4.58-4.47 (m, 1 H), 4.29-4.20 (m, 1 H), 3.76-3.58 (m, 2 H), 3.23-2.85 (m, 8 H), 2.74-2.63 (m, 1 H), 2.25-2.03 (m, 3 H), 1.99-1.86 (m, 1 H), 1.69-1.52 (m, 6 H), 1.51-1.36 (m, 12 H), 1.03-0.86 (m, 6 H); **^{13}C NMR** (100 MHz, $CDCl_3$) δ = 173.5, 172.0, 170.9, 169.9, 155.7, 79.5, 61.1, 57.2, 55.0, 47.4, 46.7, 41.6, 37.1, 35.9, 28.3, 28.2, 27.8, 25.6, 25.2, 24.5, 23.6, 23.2, 21.9; **HRMS** (ESI) calcd for $C_{25}H_{45}N_5NaO_6S$ [M+Na$^+$]: 566.2983, found: 566.2988.

S10, white solid, $[\alpha]_D^{25}$ = -62.4 (c = 0.34, $CHCl_3$); **m.p.** 158-161 ℃; **^{1}H NMR** (400 MHz, $CDCl_3$) δ = 7.55-7.32 (m, 2 H), 7.31-7.13 (m, 5 H), 6.55 (brs, 1 H), 5.22-5.04 (m, 1 H), 4.62-4.48 (m, 1 H), 4.32-4.15 (m, 1 H), 3.79-3.55 (m, 2 H), 3.18-2.94 (m, 3 H), 2.92-2.81 (m, 6 H), 2.79-2.68 (m, 1 H), 2.27-2.01 (m, 3 H), 2.00-1.81 (m, 1 H), 1.66 (t, J = 8.8 Hz, 1 H), 1.52 (s, 3 H), 1.44 (s, 9 H), 1.24 (s, 3 H); **^{13}C NMR** (100 MHz, $CDCl_3$) δ = 173.2, 171.0, 170.0, 155.7, 136.9, 129.3, 128.1, 126.5, 79.4, 61.1, 57.2, 55.1, 49.4, 47.4, 38.7, 37.0, 35.8, 28.3, 28.2, 27.6, 25.7, 25.3, 23.5; **HRMS** (ESI) calcd for $C_{28}H_{43}N_5NaO_6S$ $[M+Na^+]$: 600.2826, found: 600.2820.

S11, white solid, $[\alpha]_D^{25}$ = -34.7 (c = 0.34, $CHCl_3$); **m.p.** 183-186 ^oC; **^{1}H NMR (**400 MHz, $CDCl_3$) δ = 7.64 (d, J = 8.4 Hz, 1 H), 7.51-7.43 (m, 2 H), 7.40-7.25 (m, 3 H), 6.86-6.61 (m, 2 H), 5.82 (d, J = 8.4 Hz, 1 H), 5.56 (d, J = 8.4 Hz, 1 H), 4.59-4.47 (m, 1 H), 4.25-4.14 (m, 1 H), 3.78-3.64 (m, 2 H), 2.83-2.69 (m, 4 H), 2.64-2.54 (m, 1 H), 2.24-2.03 (m, 3 H), 2.02-1.89 (m, 1 H), 1.58 (s, 3 H), 1.54-1.37 (m, 13 H); **^{13}C NMR** (100 MHz, $CDCl_3$) δ = 173.8, 172.4, 170.9, 169.5, 155.1, 137.3, 128.5, 127.9, 127.8, 80.2, 61.0, 57.6, 57.0, 54.1, 47.8, 28.7, 28.2, 27.3, 26.4, 26.1, 25.1, 23.4; **HRMS** (ESI) calcd for $C_{26}H_{39}N_5NaO_6S$ $[M+Na^+]$: 572.2513, found: 572.2511.

S12, white solid, $[\alpha]_D^{25}$ = 37.9 (c = 0.40, $CHCl_3$); **m.p.** 153-156 ℃; **^{1}H NMR** (400 MHz, $CDCl_3$) δ = 7.75 (d, J = 8.0 Hz, 1 H), 7.52-7.42 (m, 2 H), 7.40-7.20 (m, 3 H), 6.92 (brs, 1 H), 6.12 (d, J = 8.4 Hz, 1 H), 5.66 (d, J = 7.6 Hz, 1 H), 4.63-4.41 (m, 1 H), 4.37-3.27 (m, 1 H), 3.81-3.63 (m, 5 H), 2.91-2.79 (m, 1 H), 2.72-2.61 (m, 1 H), 2.23-2.02 (m, 3 H), 2.00-1.85 (m, 1 H), 1.65-1.53 (m, 4 H), 1.48-1.33 (m, 12 H); **^{13}C NMR** (100 MHz, $CDCl_3$) δ = 173.6, 171.6, 171.0, 169.7, 155.3, 136.5, 128.6, 128.1, 127.3, 79.9, 61.0, 57.1, 56.0, 54.7, 52.6, 47.5, 28.4, 28.2, 26.7, 25.8, 25.0, 24.0; **HRMS** (ESI) calcd for $C_{26}H_{38}N_4NaO_7S$ $[M+Na^+]$: 573.2353, found: 573.2348.

S13, white solid, $[\alpha]_D^{25}$ = 3.95 (c = 0.38, $CHCl_3$); **m.p.** 204-205 ℃; **^{1}H NMR** (400 MHz, $CDCl_3$) δ = 7.75 (d, J = 8.0 Hz, 1 H), 7.47-7.23 (m, 6 H), 7.03 (brs, 1 H), 5.71 (d, J = 7.6 Hz, 1 H), 4.62-4.48 (m, 1 H), 4.41-4.30 (m, 1 H), 3.76-3.51 (m, 4 H), 3.48-3.33 (m, 1 H), 3.21-2.92 (m, 2 H), 2.77-2.62 (m, 1 H), 2.28-2.13 (m, 2 H), 2.08-1.63 (m, 7 H), 1.56 (s, 3 H), 1.43 (s, 9 H), 1.30 (s, 3 H); **^{13}C NMR** (100 MHz, $CDCl_3$) δ = 172.9, 170.5, 170.0, 167.9, 155.7, 137.3, 128.6, 127.9, 127.8, 79.5, 60.8, 57.1, 55.1, 54.8, 47.3, 46.3, 46.1, 28.3, 27.9, 27.5, 25.9, 25.8, 25.2, 23.7, 23.2; **HRMS** (ESI) calcd for $C_{29}H_{43}N_5NaO_6S$ $[M+Na^+]$: 612.2826, found: 612.2828.

S14, white solid, $[\alpha]_D^{25}$ = 29.7 (c = 0.36, $CHCl_3$); **m.p.** 186-187 ℃; **^{1}H NMR** (400 MHz, $CDCl_3$) δ = 7.68 (d, J = 8.0 Hz, 1 H), 7.42-7.34 (m, 3 H), 7.33-7.25 (m, 2 H), 7.22-7.15 (m, 1 H), 5.52-5.38 (m, 1 H), 5.12-4.96 (m, 1 H), 4.47-4.26 (m, 2 H), 4.00-3.86 (m, 1 H), 3.80-3.63 (m, 1

H), 2.91-2.80 (m, 1 H), 2.79-2.67 (m, 1 H), 2.29-2.12 (m, 1 H), 2.08-1.90 (m, 3 H), 1.69-1.60 (m, 2 H), 1.51-1.46 (m, 4 H), 1.43 (s, 9 H), 1.01-0.87 (m, 2 H); **^{13}C NMR** (100 MHz, $CDCl_3$) δ = 172.6, 170.70, 170.66, 155.8, 144.4, 128.2, 126.4, 80.7, 61.5, 54.4, 49.1, 47.8, 34.2, 29.2, 28.2, 25.4, 24.7, 22.5, 16.6; **HRMS** (ESI) calcd for $C_{25}H_{37}N_4O_5S$ [M+H$^+$]: 505.2479, found: 505.2474.

The thiol catalyst **S8** was synthesized as follows:[8-10]

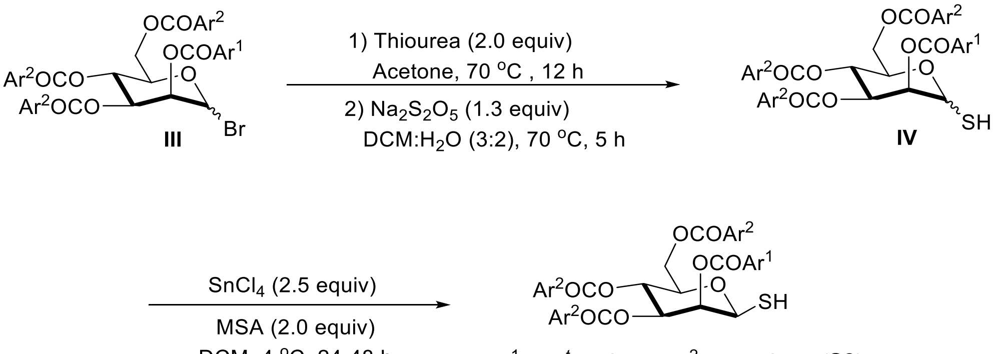

Ar[1] = 4-^{t}Bu-C_6H_4, Ar[2] = 4-F-C_6H_4 (**S8**)

A stirred solution of compound **III**[8] (7.7 g, 10.0 mmol) and thiourea (1.52 g, 20.0 mmol) in acetone (30 mL) was refluxed under N_2 atmosphere for 12 h. The reaction was cooled to room temperature, after which the solvent was removed under reduced pressure to give the isothiouronium salt as colorless foam. To a suspension of this salt in water (24 mL) and dichloromethane (36 mL) was added $Na_2S_2O_5$ (2.99 g, 15.73 mmol) and the mixture was stirred under reflux for 5 h until all the solid had dissolved. The reaction was cooled to room temperature, the organic layer was separated and the aqueous layer was extracted with dichloromethane. The combined organic phase was dried and concentrated under reduced pressure. The residue was purified by flash column chromatography to give the *α,β*-anomers **IV** (5.31 g, 73%) as colorless foam.

The *α,β*-anomers **IV** (1.0 g) was dissolved in CH_2Cl_2 (5 mL) under nitrogen and $SnCl_4$ (1.0 M in CH_2Cl_2, 2.5 equiv, 3.5 mL), followed by methane sulfonic acid (1.0 M in CH_2Cl_2, 2.0 equiv, 2.8 mL) was added slowly. The reaction mixture was stirred at 4 ℃ for 24 h. The reaction was diluted with EtOAc and quenched by addition of 1.0 M $KHSO_4$. The aqueous layer was extracted with EtOAc. The combined organic phase was washed with saturated aqueous $NaHCO_3$ and brine, dried over Na_2SO_4, and concentrated under reduced pressure. The residue was purified by flash column chromatography (PE/CH_2Cl_2/Ether = 14/10/1) to give the *β*-anomer **S8** as colorless foam (0.54 g, 54%); **m.p.** 91-93 ℃; **^{1}H NMR** (400 MHz, $CDCl_3$) δ = 8.19-8.10 (m, 2 H), 8.03-7.96 (m, 2 H), 7.95-7.88 (m, 2 H), 7.84-7.76 (m, 2 H), 7.47-7.40 (m, 2 H), 7.16-7.08 (m, 2 H), 7.06-6.99 (m, 2 H), 6.98-6.90 (m, 2 H), 6.02-5.92 (m, 1 H), 5.89 (d, J = 3.2 Hz, 1 H), 5.61 (dd, J_1 = 10.0 Hz, J_2 = 3.2 Hz, 1 H), 5.17 (d, J = 10.0 Hz, 1 H), 4.74 (dd, J_1 = 12.0 Hz, J_2 = 2.4 Hz, 1 H), 4.45 (dd, J_1 = 12.2 Hz, J_2 = 4.2 Hz, 1 H), 4.21-4.10 (m, 1 H), 2.64 (d, J= 10.0 Hz, 1 H), 1.38 (s, 9

H); **^{13}C NMR** (100 MHz, $CDCl_3$) δ = 166.0 (d, $J_{C\text{-}F}$ = 254.0 Hz), 165.9 (d, $J_{C\text{-}F}$ = 253.2 Hz), 165.8 (d, $J_{C\text{-}F}$ = 252.8 Hz), 165.2, 165.0, 164.6, 164.3, 157.7, 132.39 (d, $J_{C\text{-}F}$ = 9.2 Hz), 132.38 (d, $J_{C\text{-}F}$ = 9.5 Hz), 132.33 (d, $J_{C\text{-}F}$ = 9.4 Hz), 129.8, 126.04 (d, J = 3.0 Hz), 125.99, 125.7, 125.0-124.8 (m), 115.71 (d, J = 21.9 Hz), 115.63 (d, J = 21.9 Hz), 115.55 (d, J = 22.0 Hz), 76.9, 76.6, 73.1, 72.3, 66.1, 62.8, 35.2, 31.0; **^{19}F NMR** (376 MHz, $CDCl_3$) δ = -104.1, -104.5, -105.1; **HRMS** (ESI) calcd for $C_{38}H_{33}F_3NaO_9S$ [M+Na$^+$]: 745.1690, found: 745.1676.

2.2. Optimization Studies

Supplementary Table 1. Optimization of deuteroboration[a]

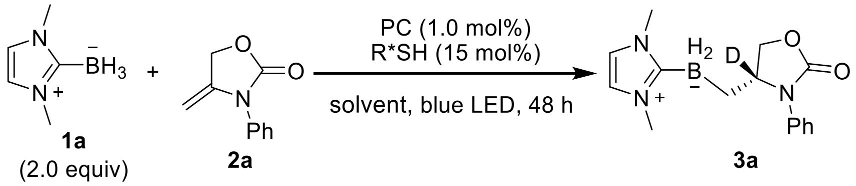

Entry	PC	R*SH	solvent	Yield/%[b]	D/%[c]	er[d]
1	4DPAIPN	**S1**	toluene:D_2O (3:1)	56	96	48:52
2	4DPAIPN	**S2**	toluene:D_2O (3:1)	79	90	51:49
3	4DPAIPN	**S3**	toluene:D_2O (3:1)	42	90	53:47
4	4DPAIPN	**S4**	toluene:D_2O (3:1)	50	95	58:42
5	4DPAIPN	**S5**	toluene:D_2O (3:1)	67	94	93:7
6	4DPAIPN	**S6**	toluene:D_2O (3:1)	49	96	93:7
7	4DPAIPN	**S7**	toluene:D_2O (3:1)	68	94	92:8
8	4DPAIPN	**S10**	toluene:D_2O (3:1)	78	92	86:14
9	4DPAIPN	**S5**	toluene:D_2O (1:1)	43	97	93:7
10	4DPAIPN	**S5**	toluene:D_2O (4:1)	71	90	93:7
11	4DPAIPN	**S5**	toluene	73	-	88:12
12	4CzIPN	**S5**	toluene:D_2O (3:1)	51	95	93:7
13[e]	4DPAIPN	**S5**	toluene:D_2O (3:1)	73	94	93:7
14[f]	4DPAIPN	**S5**	toluene:D_2O (3:1)	N.D.	-	-
15[g]	4DPAIPN	**S5**	toluene:D_2O (3:1)	N.D.	-	-
16[h]	4DPAIPN	**S5**	toluene:D_2O (3:1)	N.D.	-	-

[a] Unless otherwise noted, all reactions were carried with **1a** (0.2 mmol), **2a** (0.1 mmol), PC (1 mol%), R*SH (15 mol%), toluene (0.75 mL), D_2O (0.25 mL) under 10 ℃ for 48 h with irradiation from a 30 W blue LED. The stirring rate for all the reactions is 400 r/min. [b] Isolated yield of **3a**. [c] Determined by ^1H NMR analysis of the isolated product. [d] Determined by chiral HPLC analysis. [e] Reaction time: 72 h. [f] No photocatalyst. [g] Without light irradiation. [h] No thiol catalyst. N.D. = Not detected.

Supplementary Table 2. Optimization of deuterosilylation using olefin **2k**[a]

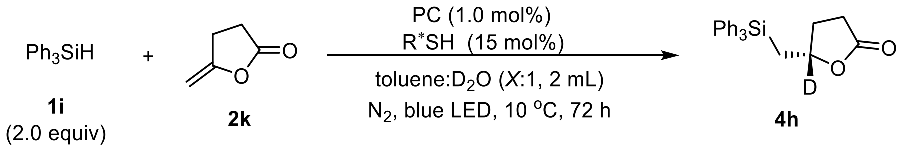

Entry	PC	R*SH	*X*	Yield/%[b]	D/%[c]	er[d]
1	4CzIPN	**S5**	3	80	93	81:19
2	4CzIPN	**S7**	3	79	91	74:26
3[e]	4CzIPN	**S7**	3	48	66	80:20
4	4DPAIPN	**S5**	3	74	94	82:18
5	4DPAIPN	**S5**	2	63	95	83:17
6	4DPAIPN	**S5**	1	52	98	82:18
7	4DPAIPN	**S8**	3	70	92	38:62
8	4DPAIPN	**S9**	3	14	90	78:22
9	4DPAIPN	**S10**	3	48	91	71:29
10	4DPAIPN	**S11**	3	67	91	69:31
11	4DPAIPN	**S12**	3	71	92	66:34
12	4DPAIPN	**S13**	3	24	90	77:23
13	4DPAIPN	**S14**	3	52	91	55:45

[a] Unless otherwise noted, all reactions were carried with **1i** (0.4 mmol), **2k** (0.2 mmol), PC (1 mol%), R*SH (15 mol%), toluene:D_2O (*X*:1, 2 mL) under 10 ℃ for 72 h with irradiation from a 30 W blue LED. The stirring rate for all the reactions is 400 r/min. [b] Isolated yield of **4h**. [c] Determined by ^{1}H NMR analysis of the isolated product. [d] Determined by chiral HPLC analysis. [e] Reaction conducted at 0 ℃.

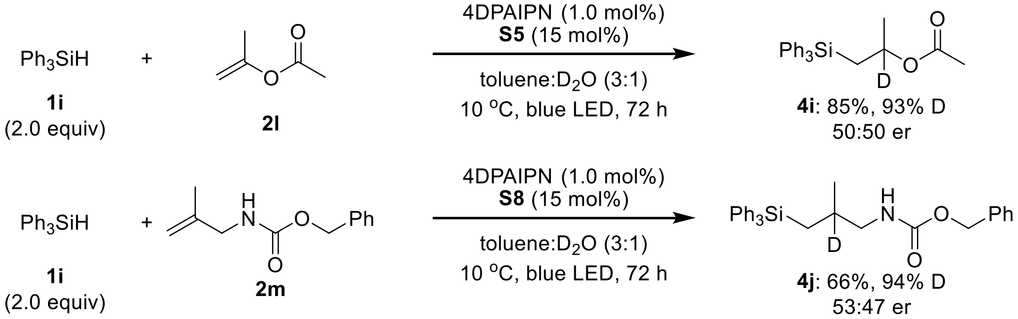

Supplementary Figure 1. Preliminary investigations on deuterosilylation reactions of acyclic 1,1-disubstituted olefins.

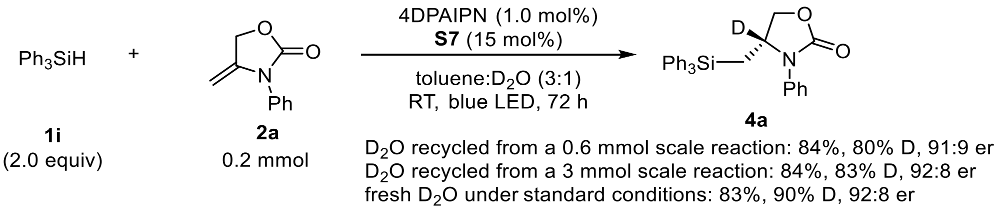

Supplementary Figure 2. Investigations on the use of recycled D_2O.

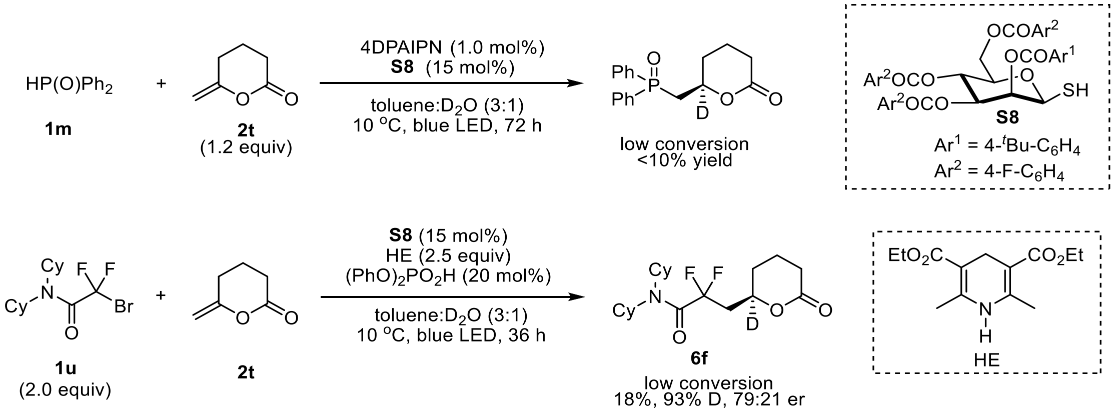

Supplementary Figure 3. Preliminary investigations on deuterophosphinoylation and deuterodifluoroalkylation reactions of **2t**.

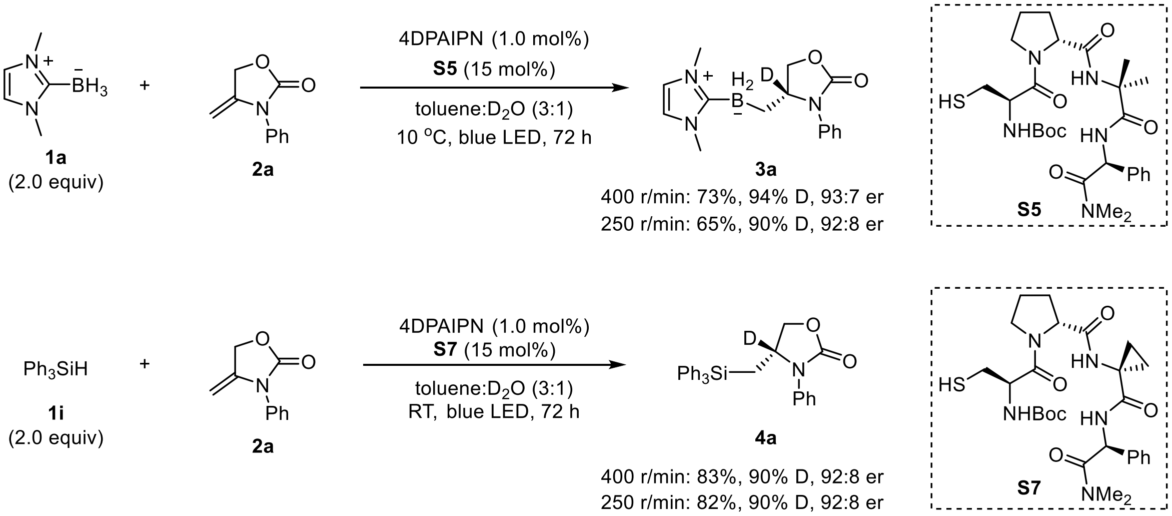

Supplementary Figure 4. Investigations on the influence of stirring rate.

2.3. Synthesis and Characterization of Products

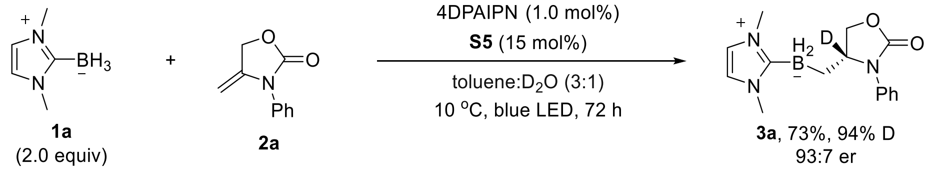

Typical procedure I: To an oven-dried 16 × 60 mm vial containing a dry Teflon stir bar were charged with 4DPAIPN (0.8 mg, 0.001 mmol), thiol catalyst **S5** (9.0 mg, 0.015 mmol), and NHC−BH_3 **1a** (22.2 mg, 0.2 mmol). After sequential addition of dry toluene (0.75 mL), D_2O (0.25 mL), and olefin **2a** (17.5 mg, 0.1 mmol), the reaction mixture was flushed with nitrogen gas for two minutes and then the vial was sealed with a cap and parafilm. The vial was placed in a cooling station and a 30 W blue LED (λ_{max} = 441 nm) was then placed on the top of the cooling station (**Supplementary Figure 5**), which is connected to a chiller to maintain the temperature of the cooling water at 10 ℃. The reaction mixture was stirred at 10 ℃ under irradiation with a stirring rate of 400 r/min for 72 h. When the reaction is complete as monitored by TLC and GC, CH_2Cl_2 (10 mL) and H_2O (5 mL) were added, the organic layer was separated and the aqueous layer was extracted with CH_2Cl_2 (10 mL × 3). The combined organic layer was washed with brine and dried over anhydrous Na_2SO_4. After filtration and evaporation, the residue was purified by preparative thin layer chromatography (eluent: petroleum ether/ethtyl acetate = 1/1) to afford **3a** (20.8 mg, 73%, 94% D) as a white solid: 93:7 er (HPLC conditions: Chiralcel OD-H column, hexane/*i*-PrOH = 60/40, 0.5 mL/min, λ = 214 nm, t_R (major) = 12.9 min, t_R (minor) = 14.8 min); $[\alpha]_D^{25}$ = -66.8 (*c* = 0.27, $CHCl_3$); **m.p.** 100-102 ℃; **^{1}H NMR** (400 MHz, $CDCl_3$) δ = 7.47-7.37 (m, 2 H), 7.37-7.28 (m, 2 H), 7.14-7.04 (m, 1 H), 6.76 (s, 2 H), 4.56 (d, *J* = 8.4 Hz, 1 H), 4.50-4.41 (m, 0.06 H), 4.27 (d, *J* = 8.4 Hz, 1 H), 3.67 (s, 6 H), 0.93 (d, *J* = 10.4 Hz, 1 H), 0.45 (d, *J* = 12.8 Hz, 1 H); **^{13}C NMR** (100 MHz, $CDCl_3$) δ = 156.5, 137.6, 128.6, 124.0, 121.7, 121.6, 120.3, 69.2, 59.1 ($J_{C\text{-}D}$ = 21.9 Hz), 35.7; **^{11}B NMR** (128 MHz, $CDCl_3$) δ = -30.3 (t, *J* = 67.5 Hz). HRMS analysis indicated that the BH_2 moiety of the product was also partially deuterated (see the first entry of the spectrum below).

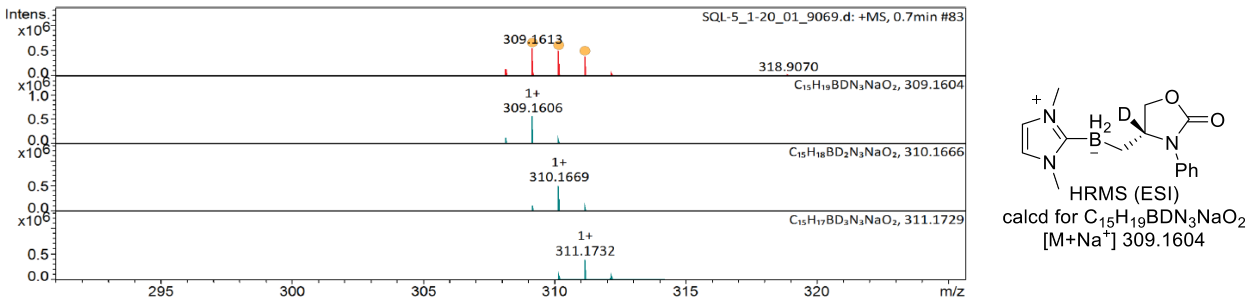

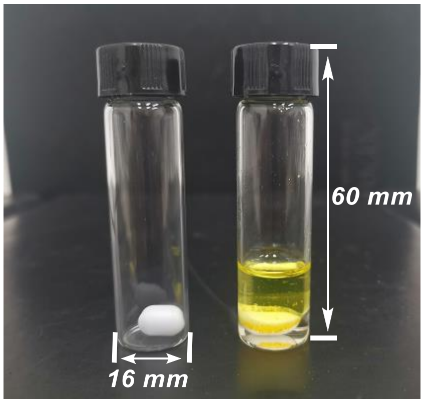

A

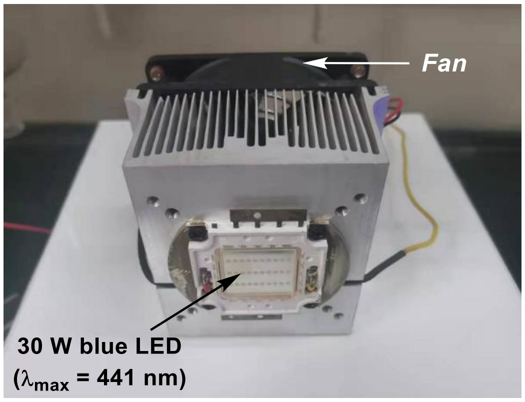

B

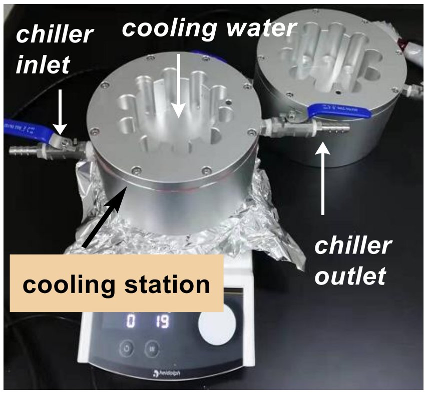

C

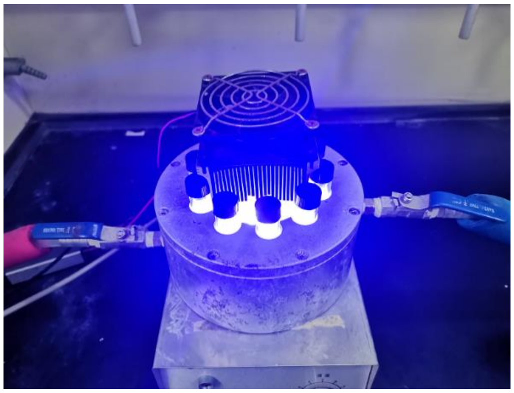

D

Supplementary Figure 5. **Reaction setup.** (**A**) 8-mL vial used for the reaction. (**B**) 30 W blue LED (λ_{max} = 441 nm) used for the reaction. (**C**) A custom-made cooling station, connected to a chiller to maintain the reaction temperature at 10 ℃. (**D**) Reaction setup (covered by a cardboard box when the light is on).

The following compounds **3-5** were prepared according to the above **Typical Procedure I** unless otherwise stated. All the racemic samples for HPLC measurement were also prepared according to this procedure but using Ph_3SiSH or CySH instead of a chiral thiol catalyst.

Synthesis of **3b:**

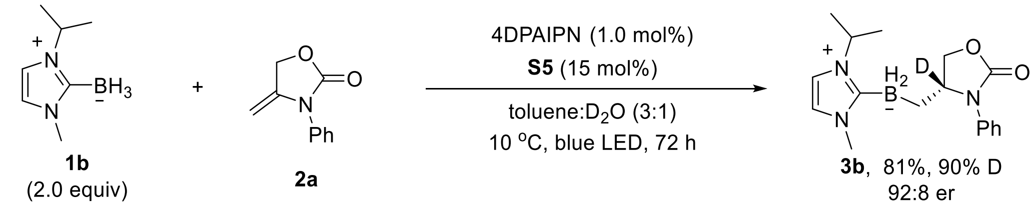

The reaction of 4DPAIPN (1.0 mg, 0.001 mmol), **1b** (27.9 mg, 0.2 mmol), **2a** (17.4 mg, 0.1 mmol), **S5** (8.6 mg, 0.015 mmol), toluene (0.75 mL) and D_2O (0.25 mL) afforded **3b** (25.5 mg, 81%, 90% D) (eluent: petroleum ether/ethtyl acetate = 1/1) as a white solid: 92:8 er (HPLC conditions: Chiralcel OD-H column, hexane/*i*-PrOH = 60/40, 0.5 mL/min, λ = 214 nm, t_R (major) = 10.4 min, t_R (minor) = 11.4 min); $[\alpha]_D^{25}$ = -72.8 (*c* = 0.17, $CHCl_3$); **m.p.** 64-65 °C; **^{1}H NMR** (400 MHz, $CDCl_3$) δ = 7.49-7.39 (m, 2 H), 7.38-7.28 (m, 2 H), 7.14-7.04 (m, 1 H), 6.91-6.85 (m, 1 H), 6.83-6.76 (m, 1 H), 5.06-4.89 (m, 1 H), 4.57 (d, *J* = 8.4 Hz, 1 H), 4.47-4.34 (m, 0.1 H), 4.25 (d, *J* = 8.4 Hz, 1 H), 3.68 (s, 3 H), 1.43-1.29 (m, 6 H), 0.94 (d, *J* = 12.8 Hz, 1 H), 0.38 (d, *J* = 12.8 Hz, 1 H); **^{13}C NMR** (100 MHz, $CDCl_3$) δ = 156.5, 137.7, 128.6, 124.0, 121.8, 120.8, 114.9, 69.4, 59.4 (t, $J_{C\text{-}D}$ = 21.8 Hz), 49.5, 35.4, 23.1, 23.0; **^{11}B NMR** (128 MHz, $CDCl_3$) δ = -30.6 (m). HRMS analysis indicated that the BH_2 moiety of the product was also partially deuterated (see the first entry of the spectrum below).

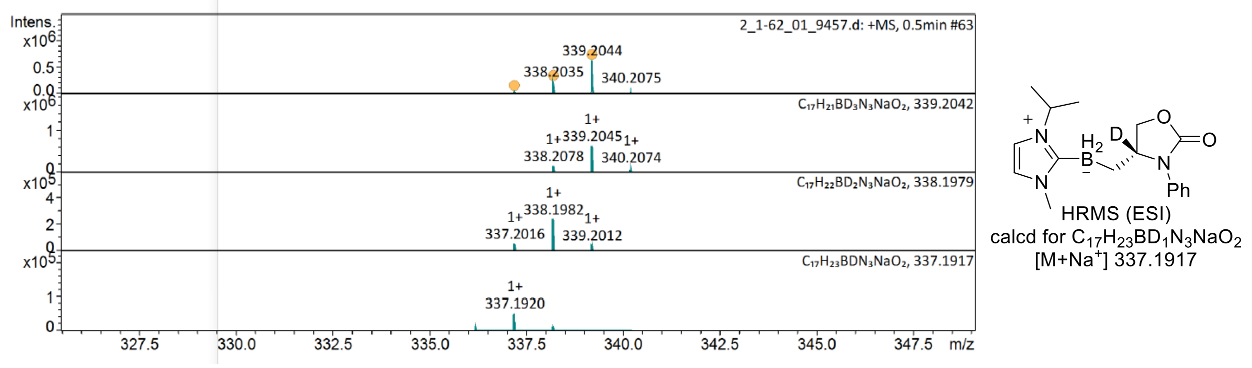

Synthesis of **3c:**

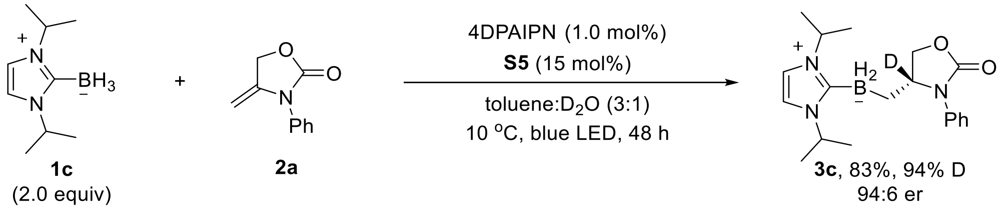

The reaction of 4DPAIPN (1.0 mg, 0.001 mmol), **1c** (33.4 mg, 0.2 mmol), **2a** (17.4 mg, 0.1

mmol), **S5** (9.3 mg, 0.015 mmol), toluene (0.75 mL) and D_2O (0.25 mL) afforded **3c** (28.4 mg, 83%, 94% D) (eluent: petroleum ether/ethtyl acetate = 1/1) as a white solid: 94:6 er (HPLC conditions: Chiralcel OD-H column, hexane/*i*-PrOH = 75/25, 0.5 mL/min, λ = 214 nm, t_R (major) = 11.7 min, t_R (minor) = 13.1 min); $[\alpha]_D^{25}$ = -74.4 (*c* = 0.16, $CHCl_3$); **m.p.** 147-150 °C; **^{1}H NMR** (400 MHz, $CDCl_3$) δ = 7.51-7.42 (m, 2 H), 7.39-7.28 (m, 2 H), 7.14-7.04 (m, 1 H), 6.91 (s, 2 H), 5.13-4.89 (m, 2 H), 4.58 (d, *J* = 8.4 Hz, 1 H), 4.49-4.39 (m, 0.06 H), 4.25 (d, *J* = 8.4 Hz, 1 H), 1.40-1.35 (m, 12 H), 0.94 (d, *J* = 12.8 Hz, 1 H), 0.36 (d, *J* = 12.8 Hz, 1 H); **^{13}C NMR** (100 MHz, $CDCl_3$) δ = 156.5, 137.7, 128.6, 123.9, 121.7, 115.4, 69.4, 59.5 (t, $J_{C\text{-}D}$ = 21.5 Hz), 49.1, 23.13, 23.11; **^{11}B NMR** (128 MHz, $CDCl_3$) δ = -30.0 (t, *J* = 63.8 Hz). HRMS analysis indicated that the BH_2 moiety of the product was also partially deuterated (see the first entry of the spectrum below).

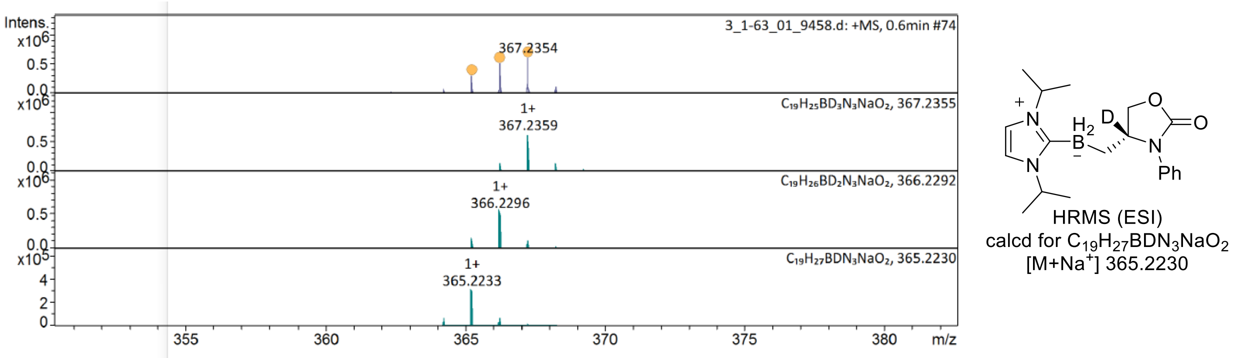

Synthesis of **3d:**

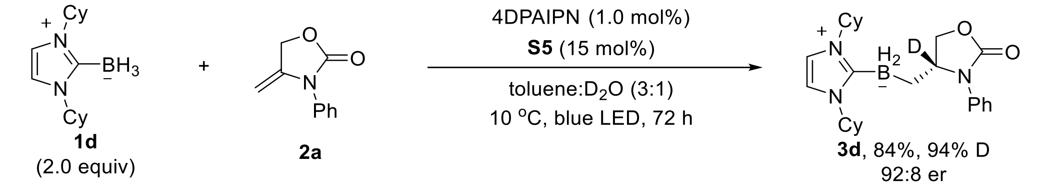

The reaction of 4DPAIPN (1.0 mg, 0.001 mmol), **1d** (49.2 mg, 0.2 mmol), **2a** (17.6 mg, 0.1 mmol), **S5** (9.2 mg, 0.015 mmol), toluene (0.75 mL) and D_2O (0.25 mL) afforded **3d** (35.6 mg, 84%, 94% D) (eluent: petroleum ether/ethtyl acetate = 1/1) as an oil: 92:8 er (HPLC conditions: Chiralcel OD-H column, hexane/*i*-PrOH = 85/15, 0.5 mL/min, λ = 214 nm, t_R (major) = 16.2 min, t_R (minor) = 20.3 min); $[\alpha]_D^{25}$ = -61.2 (*c* = 0.10, $CHCl_3$); **^{1}H NMR** (400 MHz, $CDCl_3$) δ = 7.52-7.40 (m, 2 H), 7.37-7.28 (m, 2 H), 7.14-7.02 (m, 1 H), 6.88 (s, 2 H), 4.70-4.51 (m, 3 H), 4.44-4.37 (m, 0.06 H), 4.23 (d, *J* = 8.4 Hz, 1 H), 2.01-1.68 (m, 10 H), 1.54-1.33 (m, 8 H), 1.32-1.10 (m, 2 H), 0.94 (d, *J* = 12.8 Hz, 1 H), 0.32 (d, *J* = 12.4 Hz, 1 H); **^{13}C NMR** (100 MHz, $CDCl_3$) δ

= 156.5, 137.8, 128.6, 123.9, 121.6, 115.9, 69.6, 56.7, 33.9, 33.8, 25.5, 25.2; **^{11}B NMR** (128 MHz, $CDCl_3$) δ = -29.9 (t, J = 56.6 Hz). HRMS analysis indicated that the BH_2 moiety of the product was also partially deuterated (see the first entry of the spectrum below).

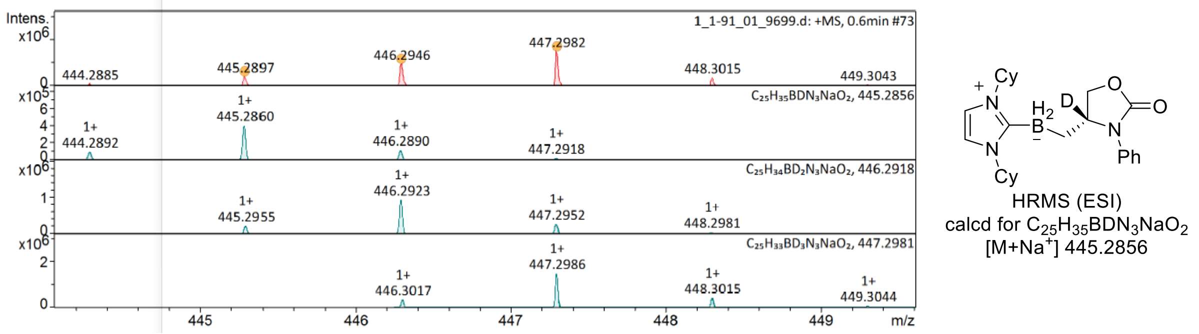

Synthesis of **3e:**

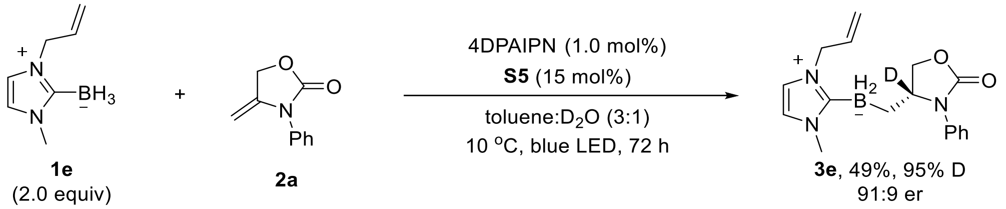

The reaction of 4DPAIPN (0.9 mg, 0.001 mmol), **1e** (27.2 mg, 0.2 mmol), **2a** (17.4 mg, 0.1 mmol), **S5** (8.7 mg, 0.015 mmol), toluene (0.75 mL) and D_2O (0.25 mL) afforded **3e** (15.3 mg, 49%, 95% D) (eluent: petroleum ether/ethtyl acetate = 1/1) as an oil: 91:9 er (HPLC conditions: Chiralcel OD-H column, hexane/i-PrOH = 80/20, 0.5 mL/min, λ = 214 nm, t_R (major) = 26.1 min, t_R (minor) = 33.7 min); $[\alpha]_D^{25}$ = -78.5 (c = 0.19, $CHCl_3$); **^{1}H NMR** (400 MHz, $CDCl_3$) δ = 7.47-7.37 (m, 2 H), 7.37-7.28 (m, 2 H), 7.15-7.03 (m, 1 H), 6.80 (s, 2 H), 5.95-5.73 (m, 1 H), 5.25 (d, J = 10.4 Hz, 1 H), 5.15 (d, J = 16.8 Hz, 1 H), 4.69 (d, J = 6.0 Hz, 2 H), 4.56 (d, J = 8.4 Hz, 1 H), 4.46-4.41 (m, 0.05 H), 4.25 (d, J = 8.4 Hz, 1 H), 3.69 (s, 3 H), 1.05-0.79 (m, 1 H), 0.48-0.33 (m, 1 H); **^{13}C NMR** (100 MHz, $CDCl_3$) δ = 156.5, 137.7, 132.4, 128.6, 123.9, 121.7, 120.6, 119.1, 118.9, 69.3, 50.8, 35.7; **^{11}B NMR** (128 MHz, $CDCl_3$) δ = -30.2 (t, J = 70.2 Hz). HRMS analysis indicated that the BH_2 moiety of the product was also partially deuterated (see the first entry of the spectrum below).

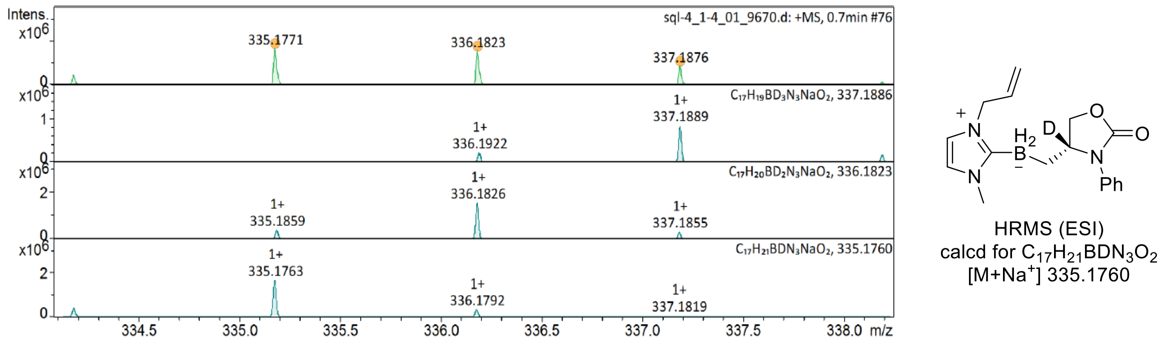

Synthesis of **3f:**

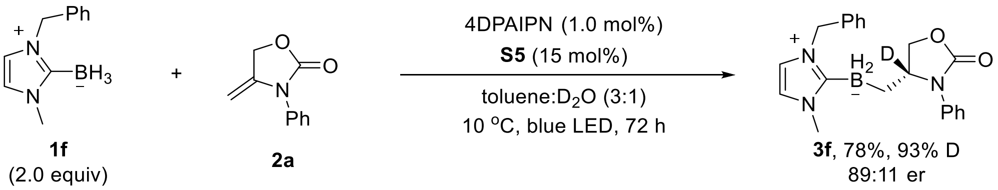

The reaction of 4DPAIPN (1.0 mg, 0.001 mmol), **1f** (37.1 mg, 0.2 mmol), **2a** (17.5 mg, 0.1 mmol), **S5** (8.9 mg, 0.015 mmol), toluene (0.75 mL) and D_2O (0.25 mL) afforded **3f** (27.0 mg, 78%, 93% D) (eluent: petroleum ether/ethtyl acetate = 1/1) as an oil: 89:11 er (HPLC conditions: Chiralcel OD-H column, hexane/*i*-PrOH = 60/40, 1.0 mL/min, λ = 214 nm, t_R (major) = 8.0 min, t_R (minor) = 10.0 min); $[\alpha]_D^{25}$ = -58.0 (*c* = 0.16, $CHCl_3$); **^{1}H NMR** (400 MHz, $CDCl_3$) δ = 7.48-7.27 (m, 7 H), 7.21-7.03 (m, 3 H), 6.78 (d, *J* = 2.0 Hz, 1 H), 6.70 (d, *J* = 1.6 Hz, 1 H), 5.37-5.16 (m, 2 H), 4.54 (d, *J* = 8.4 Hz, 1 H), 4.46-4.36 (m, 0.07 H), 4.31-4.17 (m, 1 H), 3.70 (s, 3 H), 0.93 (d, *J* = 12.0 Hz, 1 H), 0.42 (d, *J* = 12.0 Hz, 1 H); **^{13}C NMR** (100 MHz, $CDCl_3$) δ = 156.5, 137.7, 135.6, 128.9, 128.6, 128.3, 127.8, 123.9, 121.7, 120.8, 119.1, 69.3, 59.3 (t, $J_{C\text{-}D}$ = 23.5 Hz), 51.9, 35.7; **^{11}B NMR** (128 MHz, $CDCl_3$) δ = -30.1 (t, *J* = 68.4 Hz). HRMS analysis indicated that the BH_2 moiety of the product was also partially deuterated (see the first entry of the spectrum below).

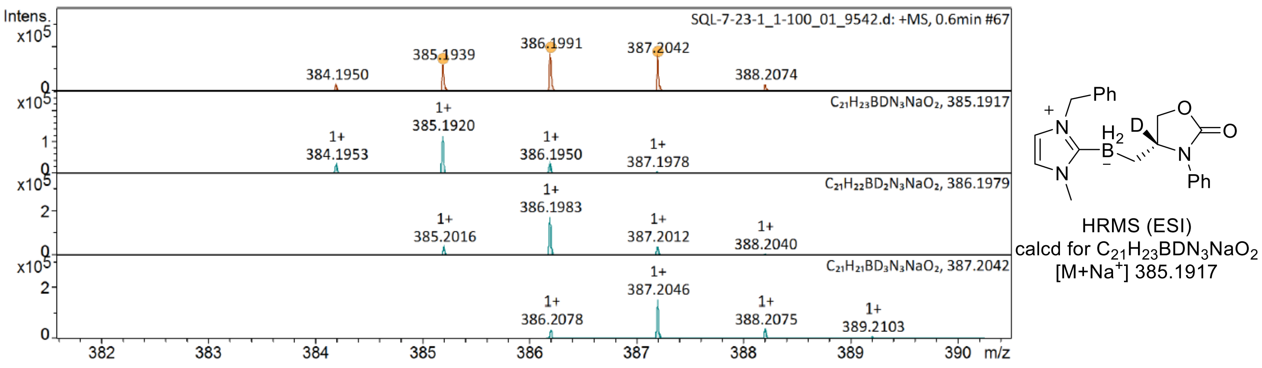

Synthesis of **3g:**

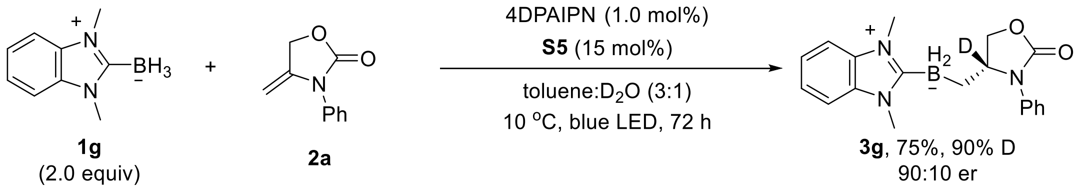

The reaction of 4DPAIPN (1.0 mg, 0.001 mmol), **1g** (32.5 mg, 0.2 mmol), **2a** (17.4 mg, 0.1 mmol), **S5** (8.5 mg, 0.015 mmol), toluene (0.75 mL) and D_2O (0.25 mL) afforded **3g** (25.2 mg, 75%, 90% D) (eluent: petroleum ether/ethtyl acetate = 1/1) as a white solid: 90:10 er (HPLC conditions: Chiralcel OD-H column, hexane/*i*-PrOH = 70/30, 0.5 mL/min, λ = 214 nm, t_R (major) = 18.6 min, t_R (minor) = 20.8 min); $[\alpha]_D^{25}$ = -79.6 (*c* = 0.11, $CHCl_3$); **m.p.** 145-147 ℃; **^{1}H NMR** (400 MHz, $CDCl_3$) δ = 7.45-7.28 (m, 6 H), 7.25-7.17 (m, 2 H), 7.09-6.94 (m, 1 H), 4.70-4.50 (m, 1.10 H), 4.37 (d, *J* = 8.4 Hz, 1 H), 3.87 (s, 6 H), 1.07-0.95 (m, 1 H), 0.77-0.59 (m, 1 H); **^{13}C NMR** (100 MHz, $CDCl_3$) δ = 156.5, 137.5, 133.0, 128.5, 124.2, 123.9, 121.4, 110.7, 68.9, 32.0; **^{11}B NMR** (128 MHz, $CDCl_3$) δ = -30.3 (t, *J* = 84.8 Hz). HRMS analysis indicated that the BH_2 moiety of the product was also partially deuterated (see the first entry of the spectrum below).

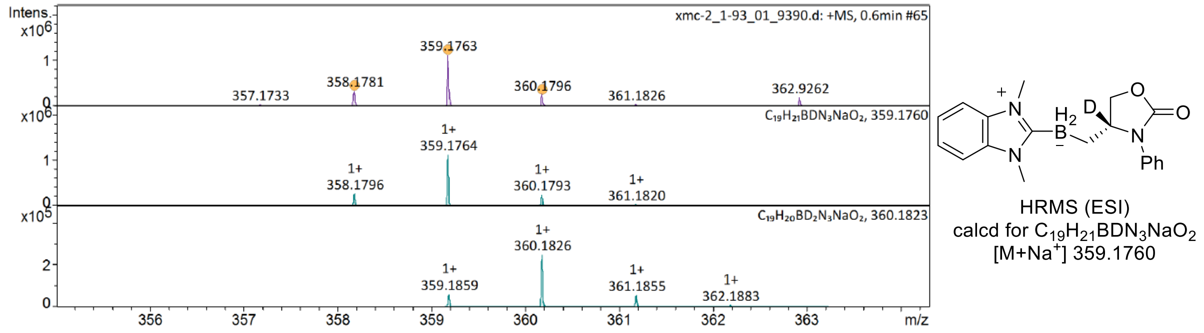

Synthesis of **3h:**

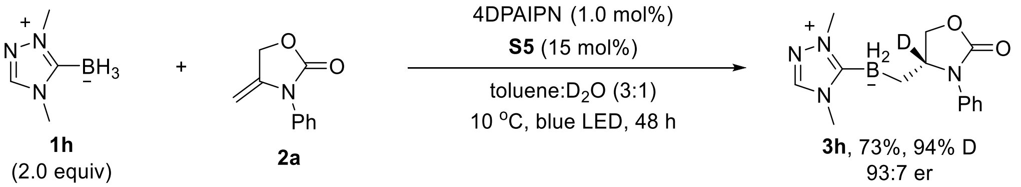

The reaction of 4DPAIPN (1.1 mg, 0.001 mmol), **1h** (22.2 mg, 0.2 mmol), **2a** (17.4 mg, 0.1 mmol), **S5** (8.6 mg, 0.015 mmol), toluene (0.75 mL) and D_2O (0.25 mL) afforded **3h** (20.9 mg, 73%, 94% D) (eluent: petroleum ether/ethtyl acetate = 1/1) as a white solid: 93:7 er (HPLC conditions: Chiralcel OD-H column, hexane/*i*-PrOH = 60/40, 0.5 mL/min, λ = 214 nm, t_R (major)

= 16.5 min, t_R (minor) = 18.3 min); $[\alpha]_D^{25}$ = -71.8 (*c* = 0.28, $CHCl_3$); **m.p.** 72-75 ℃; **^{1}H NMR** (400 MHz, $CDCl_3$) δ = 7.83 (s, 1 H), 7.43-7.29 (m, 4 H), 7.15-7.05 (m, 1 H), 4.60-4.45 (m, 1.06 H), 4.30 (d, *J* = 8.4 Hz, 1 H), 3.87 (s, 3 H), 3.66 (s, 3 H), 1.03-0.80 (m, 1 H), 0.70-0.50 (m, 1 H); **^{13}C NMR** (100 MHz, $CDCl_3$) δ = 156.4, 141.4, 137.5, 128.7, 124.1, 121.4, 68.8, 58.6 (t, $J_{C\text{-}D}$ = 21.6 Hz), 38.0, 33.5; **^{11}B NMR** (128 MHz, $CDCl_3$) δ = -30.8 (t, *J* = 84.1 Hz). HRMS analysis indicated that the BH_2 moiety of the product was also partially deuterated (see the first entry of the spectrum below).

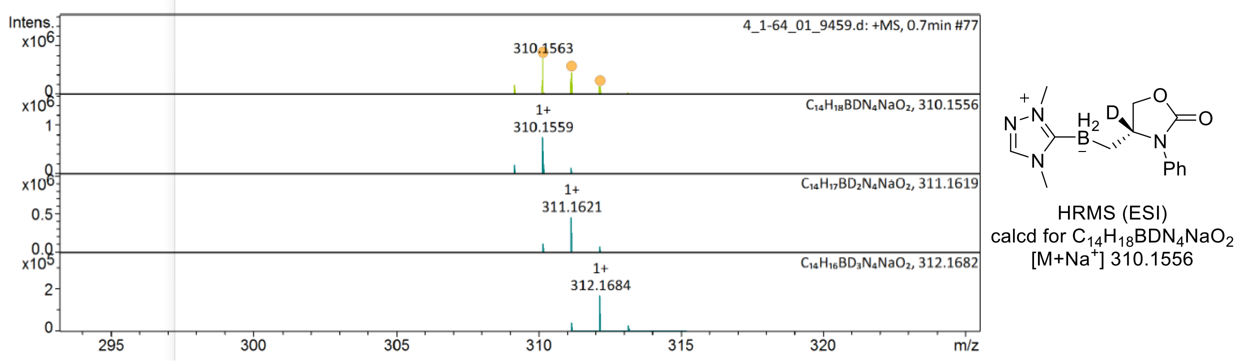

Synthesis of **3i:**

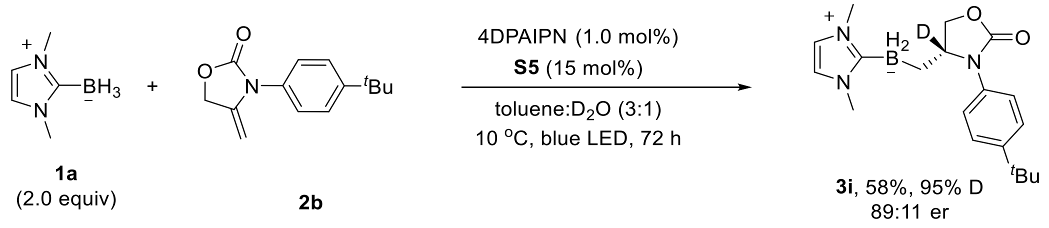

The reaction of 4DPAIPN (1.0 mg, 0.001 mmol), **1a** (22.3 mg, 0.2 mmol), **2b** (23.3 mg, 0.1 mmol), **S5** (8.8 mg, 0.015 mmol), toluene (0.75 mL) and D_2O (0.25 mL) afforded **3i** (20.0 mg, 58%, 95% D) (eluent: petroleum ether/ethtyl acetate = 1/1) as a white solid: 89:11 er (HPLC conditions: Chiralcel AD-H column, hexane/*i*-PrOH = 85/15, 1.0 mL/min, λ = 214 nm, t_R (minor) = 14.1 min, t_R (major) = 15.2 min); $[\alpha]_D^{25}$ = -61.4 (*c* = 0.36, $CHCl_3$); **m.p.** 114-117 ℃; **^{1}H NMR** (400 MHz, $CDCl_3$) δ = 7.38-7.28 (m, 4 H), 6.76 (s, 2 H), 4.55 (d, *J* = 8.4 Hz, 1 H), 4.47-4.37 (m, 0.05 H), 4.24 (d, *J* = 8.4 Hz, 1 H), 3.67 (s, 6 H), 1.29 (s, 9 H), 0.94 (d, *J* =12.4 Hz, 1 H), 0.45 (d, *J* = 12.8 Hz, 1 H); **^{13}C NMR** (100 MHz, $CDCl_3$) δ =156.7, 146.9, 134.9, 125.5, 121.4, 120.3, 69.3, 35.7, 34.3, 31.3; **^{11}B NMR** (128 MHz, $CDCl_3$) δ = -30.3 (t, *J* = 65.9 Hz). HRMS analysis indicated that the BH_2 moiety of the product was also partially deuterated (see the first entry of the spectrum below).

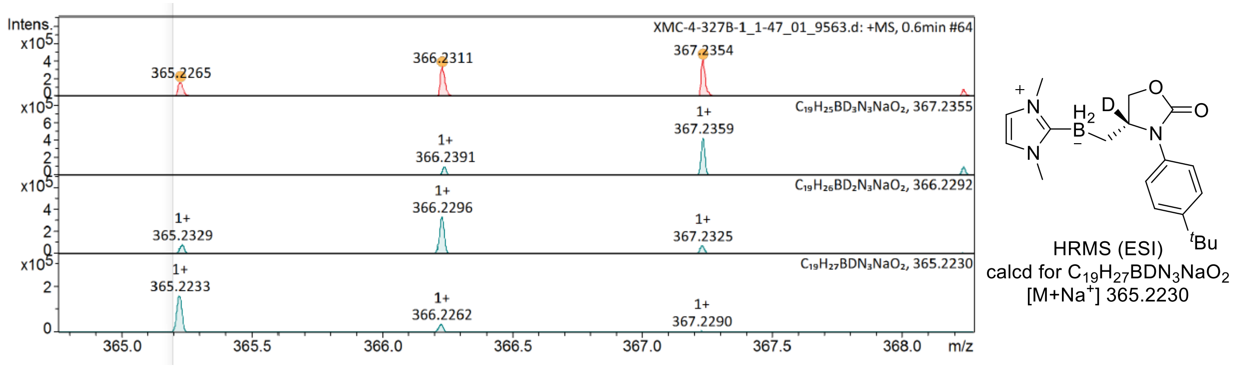

Synthesis of **3j:**

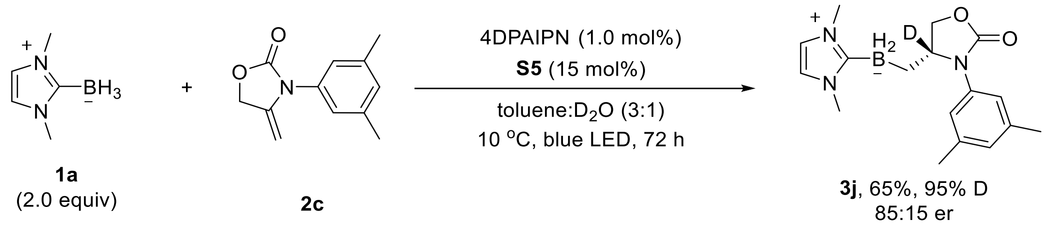

The reaction of 4DPAIPN (1.0 mg, 0.001 mmol), **1a** (22.3 mg, 0.2 mmol), **2c** (20.3 mg, 0.1 mmol), **S5** (8.5 mg, 0.015 mmol), toluene (0.75 mL) and D_2O (0.25 mL) afforded **3j** (20.4 mg, 65%, 95% D) (eluent: petroleum ether/ethtyl acetate = 1/1) as a white solid: 85:15 er (HPLC conditions: Chiralcel OD-H column, hexane/*i*-PrOH = 60/40, 0.5 mL/min, λ = 214 nm, t_R (major) = 11.1 min, t_R (minor) = 16.4 min); $[\alpha]_D^{25}$ = -54.5 (c = 0.17, $CHCl_3$); **m.p.** 104-106 ℃; **^{1}H NMR** (400 MHz, $CDCl_3$) δ = 7.00 (s, 2 H), 6.78-6.68 (m, 3 H), 4.54 (d, J = 8.4 Hz, 1 H), 4.44-4.37 (m, 0.05 H), 4.24 (d, J = 8.4 Hz, 1 H), 3.68 (s, 6 H), 2.29 (s, 6 H), 0.91 (d, J = 12.8 Hz, 1 H), 0.44 (d, J = 12.0 Hz, 1 H); **^{13}C NMR** (100 MHz, $CDCl_3$) δ = 156.6, 138.2, 137.4, 126.0, 120.2, 119.78, 119.77, 69.3, 35.7, 21.4; **^{11}B NMR** (128 MHz, $CDCl_3$) δ = -30.3 (t, J = 67.4 Hz). HRMS analysis indicated that the BH_2 moiety of the product was also partially deuterated (see the first entry of the spectrum below).

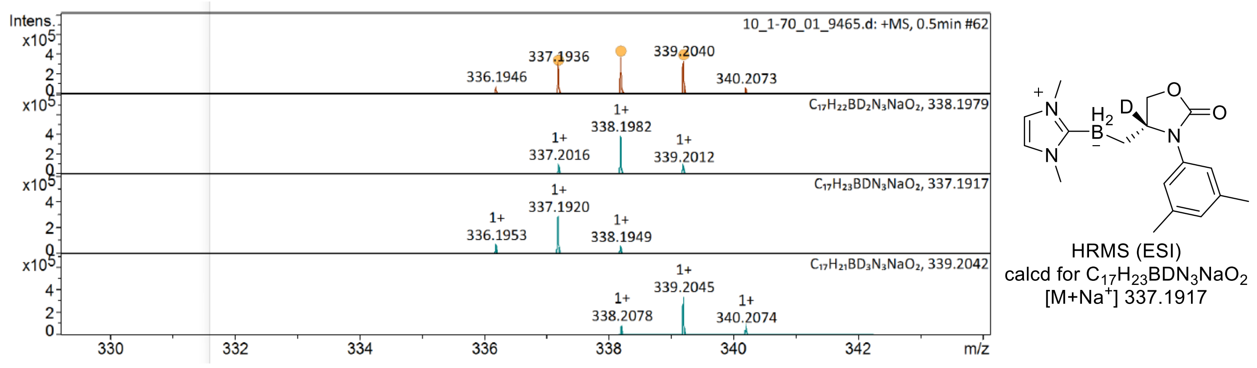

Synthesis of **3k:**

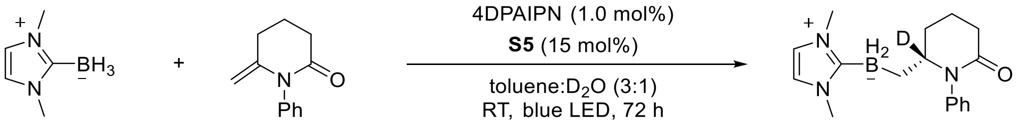

1a (2.0 equiv) **2d** **3k**, 63%, 90% D 97:3 er

The reaction of 4DPAIPN (0.9 mg, 0.001 mmol), **1a** (22.5 mg, 0.2 mmol), **2d** (18.8 mg, 0.1 mmol), **S5** (8.7 mg, 0.015 mmol), toluene (0.75 mL) and D_2O (0.25 mL) under room temperature afforded **3k** (18.0 mg, 63%, 90% D) (eluent: CH_2Cl_2/MeOH = 20/1) as an oil: 97:3 er (HPLC conditions: Chiralcel AD-H column, hexane/*i*-PrOH = 90/10, 0.5 mL/min, λ = 214 nm, t_R (major) = 102.0 min, t_R (minor) = 108.8 min); $[\alpha]_D^{25}$ = -74.0 (*c* = 0.05, $CHCl_3$); **^{1}H NMR** (400 MHz, $CDCl_3$) δ = 7.37-7.24 (m, 2 H), 7.22-7.08 (m, 3 H), 6.71 (s, 2 H), 3.61-3.48 (m, 6.10 H), 2.61-2.38 (m, 2 H), 2.16-1.96 (m, 3 H), 1.84-1.69 (m, 1 H), 0.65-0.36 (m, 2 H); **^{13}C NMR** (100 MHz, $CDCl_3$) δ = 170.3, 142.8, 128.4, 128.2, 126.0, 120.0, 63.8 ($J_{C\text{-}D}$ = 20.7 Hz), 35.6, 33.0, 27.9, 18.1; **^{11}B NMR** (128 MHz, $CDCl_3$) δ = -29.1 (t, *J* = 66.9 Hz). HRMS analysis indicated that the BH_2 moiety of the product was also partially deuterated (see the first entry of the spectrum below).

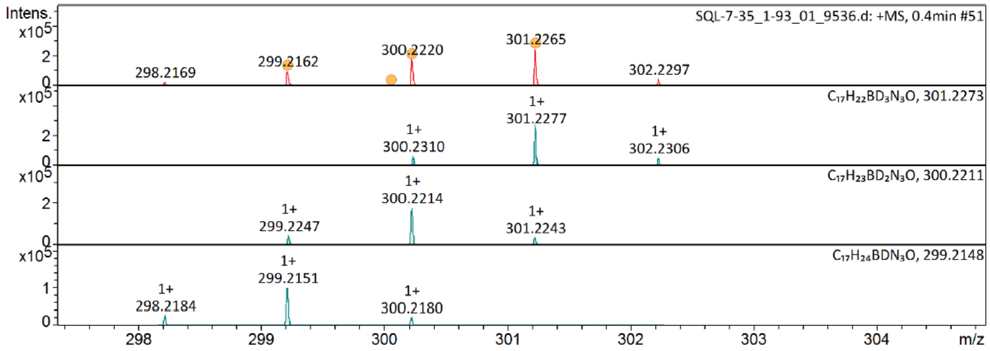

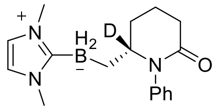

HRMS (ESI)
calcd for $C_{17}H_{24}BDN_3O$
[M+H$^+$] 299.2148

Synthesis of **3l:**

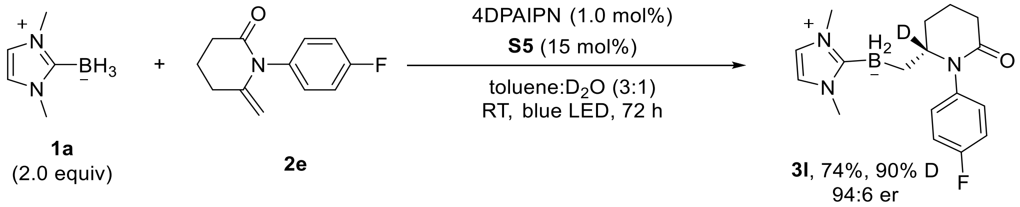

The reaction of 4DPAIPN (1.0 mg, 0.001 mmol), **1a** (22.7 mg, 0.2 mmol), **2e** (20.5 mg, 0.1 mmol), **S5** (8.9 mg, 0.015 mmol), toluene (0.75 mL) and D_2O (0.25 mL) under room temperature afforded **3l** (23.3 mg, 74%, 90% D) (eluent: CH_2Cl_2/MeOH = 20/1) as an oil: 94:6 er (HPLC conditions: Chiralcel OD-H column, hexane/*i*-PrOH = 80/20, 1 mL/min, λ = 214 nm, t_R (major) = 12.9 min, t_R (minor) = 14.3 min); $[\alpha]_D^{25}$ = -46.9 (c = 0.27, $CHCl_3$); **^{1}H NMR** (400 MHz, $CDCl_3$) δ = 7.16-7.05 (m, 2 H), 7.04-6.94 (m, 2 H), 6.74 (s, 2 H), 3.61-3.53 (m, 6.10 H), 2.58-2.40 (m, 2 H), 2.11-1.93 (m, 3 H), 1.82-1.69 (m, 1 H), 0.59-0.34 (m, 2 H); **^{13}C NMR** (100 MHz, $CDCl_3$) δ = 170.6, 161.9, 159.5, 138.6, 129.7 (d, $J_{C\text{-}F}$ = 8.3 Hz), 120.1, 115.2 (d, $J_{C\text{-}F}$ = 22.2 Hz), 35.6, 33.0, 28.0, 18.1; **^{11}B NMR** (128 MHz, $CDCl_3$) δ = -29.2 (t, J = 66.8 Hz); **^{19}F NMR** (376 MHz, $CDCl_3$) δ = -116.6. HRMS analysis indicated that the BH_2 moiety of the product was also partially deuterated (see the first entry of the spectrum below).

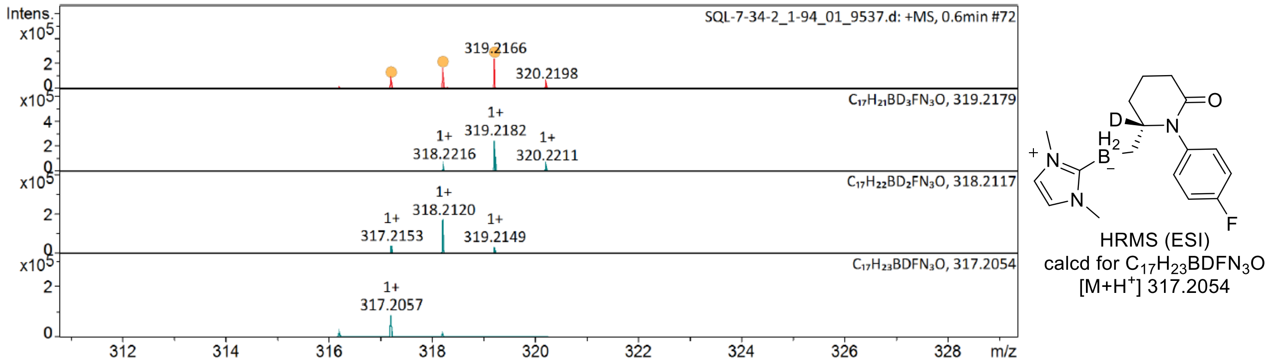

Synthesis of **3m:**

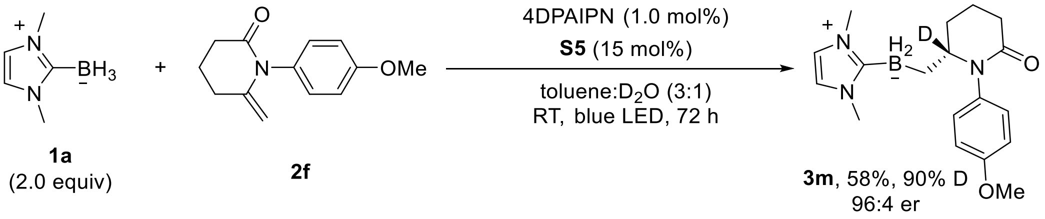

The reaction of 4DPAIPN (0.9 mg, 0.001 mmol), **1a** (22.3 mg, 0.2 mmol), **2f** (21.5 mg, 0.1 mmol), **S5** (8.9 mg, 0.015 mmol), toluene (0.75 mL) and D_2O (0.25 mL) under room temperature afforded **3m** (19.1 mg, 58%, 90% D) (eluent: CH_2Cl_2/MeOH = 20/1) as an oil: 96:4 er (HPLC conditions: Chiralcel OD-H column, hexane/*i*-PrOH = 70/30, 1 mL/min, λ = 214 nm, t_R (major) = 10.7 min, t_R (minor) = 15.6 min); $[\alpha]_D^{25}$ = -82.1 (*c* = 0.19, $CHCl_3$); **^{1}H NMR** (400 MHz, $CDCl_3$) δ = 7.10-6.99 (m, 2 H), 6.91-6.80 (m , 2 H), 6.73 (s, 2 H), 3.79 (s, 3 H), 3.69-3.48 (m, 6.10 H), 2.60-2.40 (m, 2 H), 2.10-1.93 (m, 3 H), 1.81-1.67 (m, 1 H), 0.59 (d, *J* = 12.8 Hz, 1 H), 0.43 (d, *J* = 13.2 Hz, 1 H); **^{13}C NMR** (100 MHz, $CDCl_3$) δ = 170.6, 157.5, 135.6, 129.1, 120.0, 113.8, 55.4, 35.6, 33.0, 27.9, 18.1; **^{11}B NMR** (128 MHz, $CDCl_3$) δ = -29.1 (t, *J* = 67.6 Hz). HRMS analysis indicated that the BH_2 moiety of the product was also partially deuterated (see the first entry of the spectrum below).

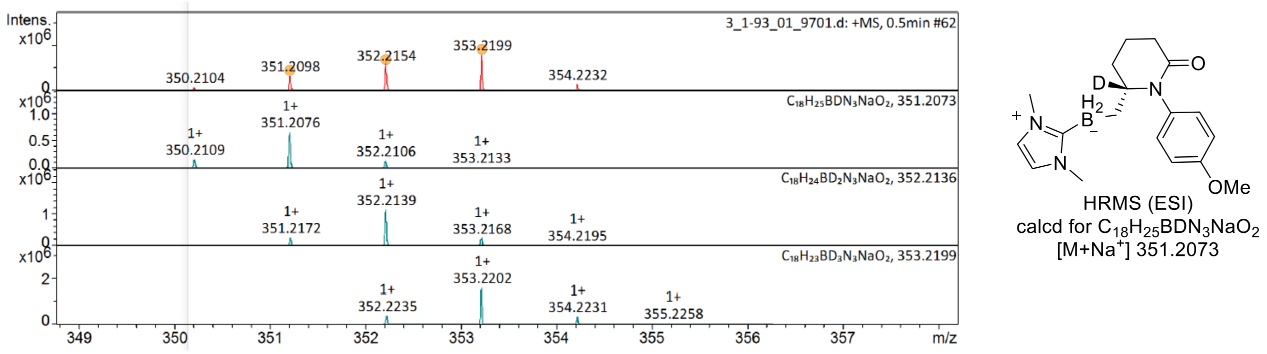

Synthesis of **3n:**

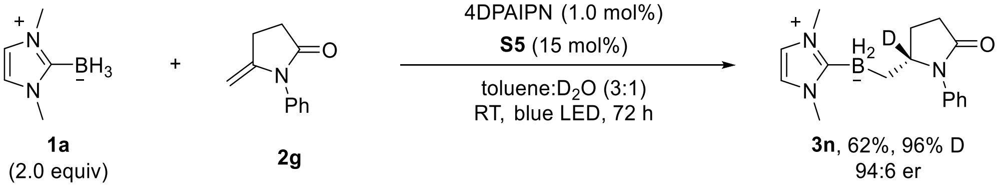

The reaction of 4DPAIPN (0.9 mg, 0.001 mmol), **1a** (22.0 mg, 0.2 mmol), **2g** (17.4 mg, 0.1 mmol), **S5** (8.9 mg, 0.015 mmol), toluene (0.75 mL) and D_2O (0.25 mL) under room temperature afforded **3n** (17.6 mg, 62%, 96% D) (eluent: PE/EA = 1/1) as a white solid: 94:6 er (HPLC conditions: Chiralcel AD-H column, hexane/*i*-PrOH = 75/25, 0.5 mL/min, λ = 214 nm, t_R (minor) = 19.1 min, t_R (major) = 21.9 min); $[\alpha]_D^{25}$ = -49.6 (*c* = 0.14, $CHCl_3$); **m.p.** 79-81 ℃; **^{1}H NMR** (400 MHz, $CDCl_3$) δ = 7.44-7.36 (m, 2 H), 7.35-7.28 (m, 2 H), 7.15-7.05 (m, 1 H), 6.75 (s, 2 H), 4.26-4.17 (m, 0.04 H), 3.66 (s, 6 H), 2.68-2.57 (m, 1 H), 2.54-2.41 (m, 1 H), 2.36-2.25 (m, 1 H), 1.99-1.87 (m, 1 H), 0.81 (d, *J* = 12.4 Hz, 1 H), 0.34 (d, *J* = 12.8 Hz, 1 H); **^{13}C NMR** (100 MHz,

CDCl3) δ = 174.8, 138.7, 128.4, 124.5, 123.7, 120.1, 35.7, 32.2, 25.5; **^{11}B NMR** (128 MHz, $CDCl_3$) δ = -29.7 (t, *J* = 64.3 Hz). HRMS analysis indicated that the BH_2 moiety of the product was also partially deuterated (see the first entry of the spectrum below).

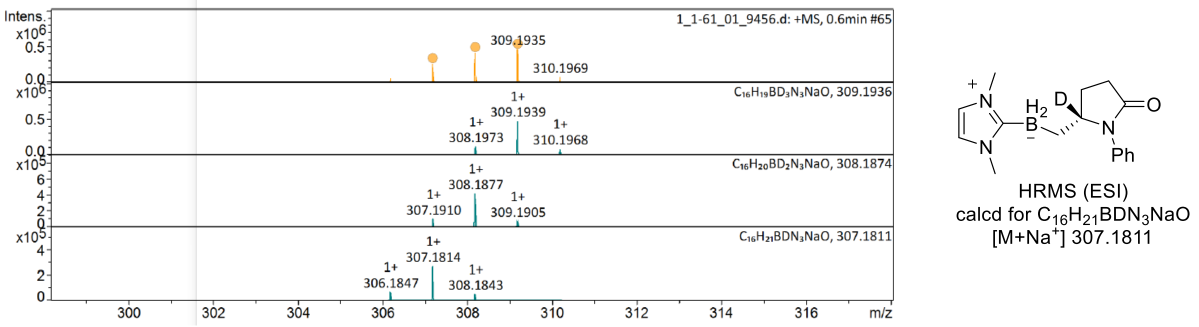

Synthesis of **3o:**

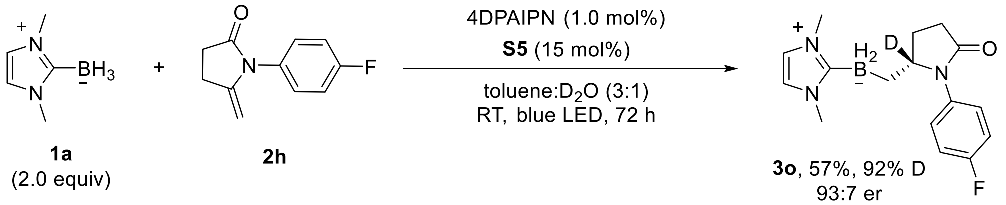

The reaction of 4DPAIPN (1.1 mg, 0.001 mmol), **1a** (22.2 mg, 0.2 mmol), **2h** (19.1 mg, 0.1 mmol), **S5** (8.8 mg, 0.015 mmol), toluene (0.75 mL) and D_2O (0.25 mL) under room temperature afforded **3o** (17.2 mg, 57%, 92% D) (eluent: CH_2Cl_2/MeOH = 20/1) as a white solid: 93:7 er (HPLC conditions: Chiralcel AD-H column, hexane/*i*-PrOH = 75/25, 0.5 mL/min, λ = 214 nm, t_R (minor) = 18.4 min, t_R (major) = 21.3 min); $[\alpha]_D^{25}$ = -52.2 (*c* = 0.07, $CHCl_3$); **m.p.** 96-100 ℃; **^{1}H NMR** (400 MHz, $CDCl_3$) δ = 7.40-7.29 (m, 2 H), 7.08-6.94 (m, 2 H), 6.76 (s, 2 H), 4.22-4.08 (m, 0.08 H), 3.67 (s, 6 H), 2.68-2.54 (m, 1 H), 2.54-2.40 (m, 1 H), 2.37-2.23 (m, 1 H), 1.97-1.84 (m, 1 H), 0.75 (d, *J* = 12.4 Hz, 1 H), 0.29 (d, *J* = 12.4 Hz, 1 H); **^{13}C NMR** (100 MHz, $CDCl_3$) δ = 174.8, 160.9, 158.5, 134.7, 125.6 (d, $J_{C\text{-}F}$ = 7.9 Hz), 120.1, 115.2 (d, $J_{C\text{-}F}$ = 22.2 Hz), 35.7, 32.0, 25.6; **^{11}B NMR** (128 MHz, $CDCl_3$) δ = -29.7 (t, *J* = 67.2 Hz); **^{19}F NMR** (376 MHz, $CDCl_3$) δ = -117.9. HRMS analysis indicated that the BH_2 moiety of the product was also partially deuterated (see the first entry of the spectrum below).

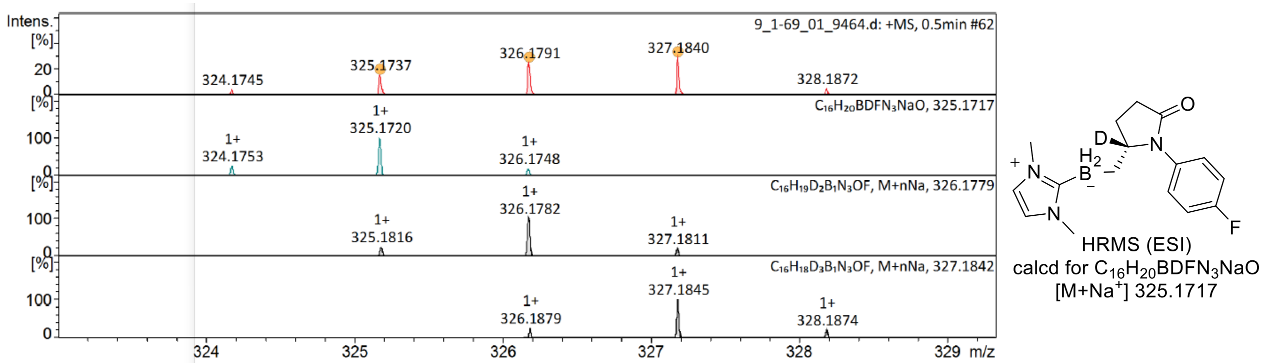

Synthesis of **3p:**

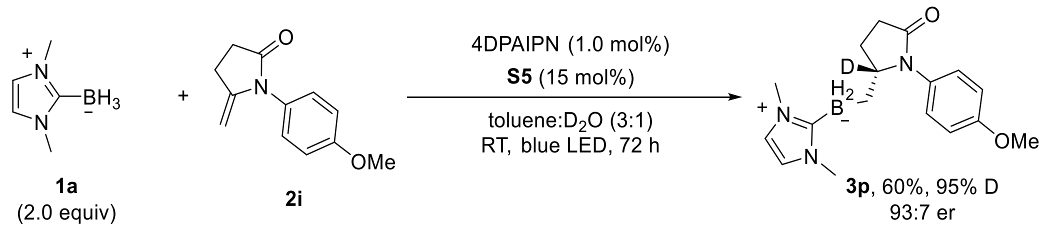

The reaction of 4DPAIPN (0.9 mg, 0.001 mmol), **1a** (22.1 mg, 0.2 mmol), **2i** (20.1 mg, 0.1 mmol), **S5** (8.9 mg, 0.015 mmol), toluene (0.75 mL) and D_2O (0.25 mL) under room temperature afforded **3p** (18.6 mg, 60%, 95% D) (eluent: CH_2Cl_2/MeOH = 20/1) as an oil: 93:7 er (HPLC conditions: Chiralcel AD-H column, hexane/*i*-PrOH = 60/40, 0.5 mL/min, λ = 214 nm, t_R (minor) = 13.0 min, t_R (major) = 14.8 min); $[\alpha]_D^{25}$ = -35.6 (*c* = 0.16, $CHCl_3$); **^{1}H NMR** (400 MHz, $CDCl_3$) δ = 7.31-7.22 (m, 2 H), 6.92-6.83 (m, 2 H), 6.75 (s, 2 H), 4.19-4.04 (m, 0.05 H), 3.78 (s, 3 H), 3.67 (s, 6 H), 2.68-2.39 (m, 2 H), 2.37-2.24 (m, 1 H), 1.97-1.82 (m, 1 H), 0.76 (d, *J* = 12.4 Hz, 1 H), 0.28 (d, *J* = 12.8 Hz, 1 H); **^{13}C NMR** (100 MHz, $CDCl_3$) δ = 174.7, 156.7, 131.7, 125.6, 120.1, 113.8, 55.4, 35.7, 32.0, 25.8; **^{11}B NMR** (128 MHz, $CDCl_3$) δ = -29.7 (t, *J* = 64.2 Hz). HRMS analysis indicated that the BH_2 moiety of the product was also partially deuterated (see the first entry of the spectrum below).

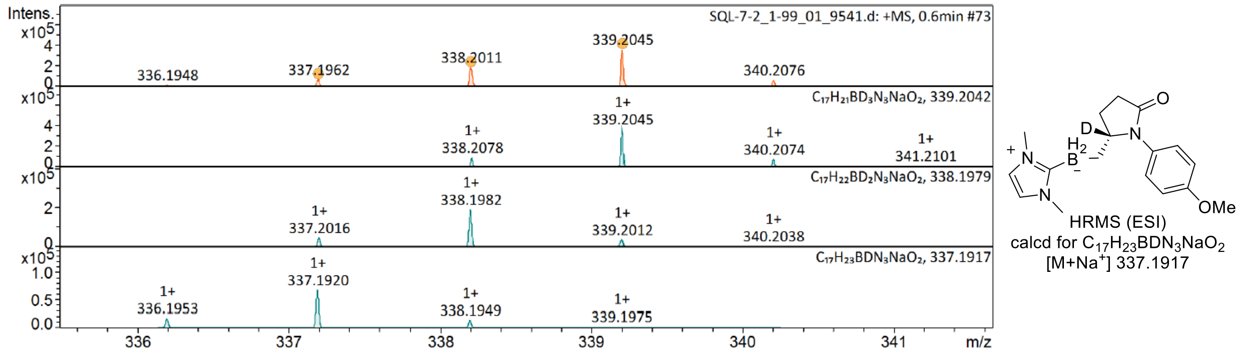

Synthesis of **4a:**

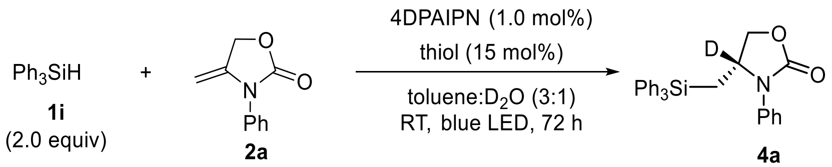

S7 as the thiol catalyst: 83%, 90% D, 92:8 er
S5 as the thiol catalyst: 85%, 89% D, 88:12 er
S1 as the thiol catalyst: 70%, 96% D, 46:54 er

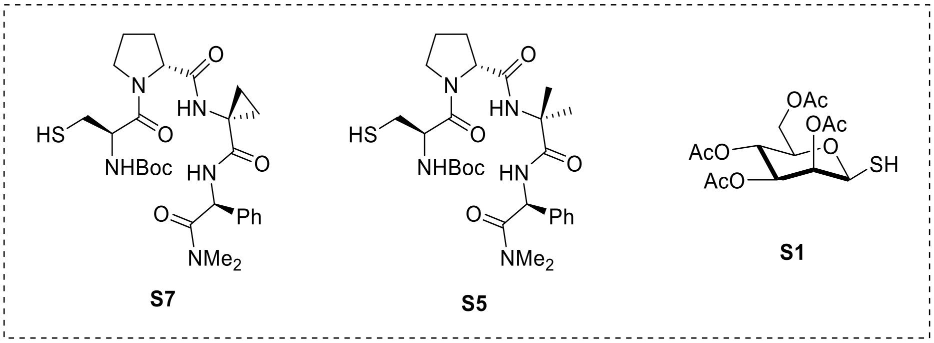

The reaction of 4DPAIPN (1.8 mg, 0.002 mmol), **1i** (107.2 mg, 0.4 mmol), **2a** (35.2 mg, 0.2 mmol), **S7** (17.8 mg, 0.03 mmol), toluene (1.5 mL) and D_2O (0.5 mL) under room temperature afforded **4a** (72.3 mg, 83%, 90% D) (purified by chromatography on silica gel, eluent: petroleum ether/ethyl acetate = 5/1) as a white solid: 92:8 er (HPLC conditions: Chiralcel AD-H column, hexane/*i*-PrOH = 90/10, 1.0 mL/min, λ = 214 nm, t_R (major) = 7.6 min, t_R (minor) = 11.5 min); $[\alpha]_D^{25}$ = +2.61 (*c* = 0.15, $CHCl_3$); **m.p.** 110-111 ℃; **^{1}H NMR** (400 MHz, $CDCl_3$) δ = 7.58-7.24 (m, 19 H), 7.22-7.14 (m, 1 H), 4.71-4.51 (m, 0.1 H), 3.95 (d, *J* = 9.2 Hz, 1 H), 3.62 (d, *J* = 8.8 Hz, 1 H), 2.05 (d, *J* = 14.8 Hz, 1 H), 1.61 (d, *J* = 14.8 Hz, 1 H); **^{13}C NMR** (100 MHz, $CDCl_3$) δ = 155.4, 136.2, 135.4, 133.0, 130.2, 129.2, 128.3, 125.4, 122.7, 68.5, 17.9; **HRMS** (ESI) calcd for $C_{28}H_{24}DNNaO_2Si$ $[M+Na^+]$: 459.1610, found: 459.1602.

The reaction of 4DPAIPN (0.9 mg, 0.001 mmol), **1i** (52.3 mg, 0.2 mmol), **2a** (17.4 mg, 0.1 mmol), **S5** (8.8 mg, 0.015 mmol), toluene (0.75 mL) and D_2O (0.25 mL) under room temperature afforded **4a** (37.1 mg, 85%, 89% D) (purified by chromatography on silica gel, eluent: petroleum ether/ethyl acetate = 5/1) as a white solid: 88:12 er (HPLC conditions: Chiralcel AD-H column, hexane/*i*-PrOH = 90/10, 1.0 mL/min, λ = 214 nm, t_R (major) = 7.6 min, t_R (minor) = 11.5 min); **^{1}H NMR** (400 MHz, $CDCl_3$) δ = 7.72-7.02 (m, 20 H), 4.66-4.50 (m, 0.11 H), 3.96 (d, *J* = 8.8 Hz, 1 H), 3.63 (d, *J* = 8.8 Hz, 1 H), 2.05 (d, *J* = 14.8 Hz, 1 H), 1.61 (d, *J* = 14.8 Hz, 1 H).

The reaction of 4DPAIPN (0.8 mg, 0.001 mmol), **1i** (52.5 mg, 0.2 mmol), **2a** (17.3 mg, 0.1 mmol), **S1** (6.2 mg, 0.015 mmol), toluene (0.75 mL) and D_2O (0.25 mL) under room temperature

afforded **4a** (30.5 mg, 70%, 96% D) (purified by chromatography on silica gel, eluent: petroleum ether/ethyl acetate = 5/1) as a white solid: 46:54 er (HPLC conditions: Chiralcel AD-H column, hexane/*i*-PrOH = 90/10, 1.0 mL/min, λ = 214 nm, t_R (minor) = 7.6 min, t_R (major) = 11.5 min); **^{1}H NMR** (400 MHz, $CDCl_3$) δ = 7.75-7.09 (m, 20 H), 4.66-4.52 (m, 0.04 H), 3.96 (d, *J* = 9.2 Hz, 1 H), 3.63 (d, *J* = 9.2 Hz, 1 H), 2.05 (d, *J* = 14.8 Hz, 1 H), 1.61 (d, *J* = 14.8 Hz, 1 H).

Gram-scale synthesis of **4a** with **S7**:

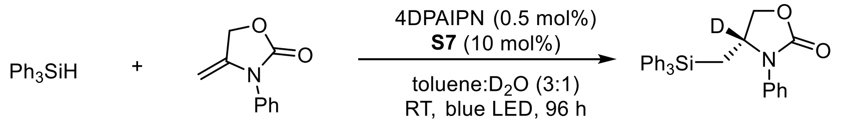

1i (2.0 equiv) **2a** **4a**, 1.08 g 82%, 91% D, 91:9 er

The reaction of 4DPAIPN (12.0 mg, 0.015 mmol), **1i** (1.5261 g, 6.0 mmol), **2a** (525.3 mg, 3.0 mmol), **S7** (168.3 mg, 0.3 mmol), toluene (22.5 mL) and D_2O (7.5 mL) under room temperature afforded **4a** (1.08 g, 82%, 91% D) (purified by chromatography on silica gel, eluent: petroleum ether/ethyl acetate = 5/1) as a white solid: 91:9 er (HPLC conditions: Chiralcel AD-H column, hexane/*i*-PrOH = 90/10, 1.0 mL/min, λ = 214 nm, t_R (major) = 7.6 min, t_R (minor) = 11.4 min); **^{1}H NMR** (400 MHz, $CDCl_3$) δ = 7.66-7.06 (m, 20 H), 4.73-4.45 (m, 0.09 H), 3.96 (d, *J* = 9.2 Hz, 1 H), 3.62 (d, *J* = 8.8 Hz, 1 H), 2.05 (d, *J* = 14.4 Hz, 1 H), 1.61 (d, *J* = 14.8 Hz, 1 H).

Synthesis of **4a** using recycled D_2O:

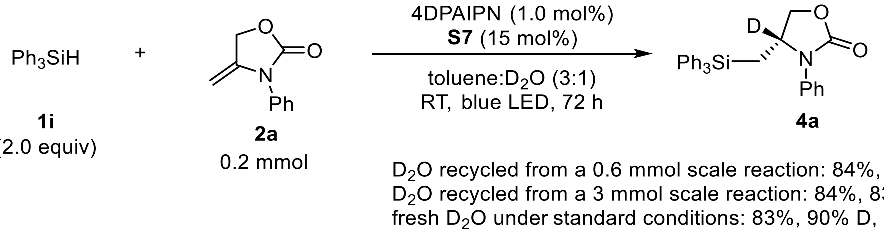

1i (2.0 equiv) **2a** 0.2 mmol **4a**

D_2O recycled from a 0.6 mmol scale reaction: 84%, 80% D, 91:9 er
D_2O recycled from a 3 mmol scale reaction: 84%, 83% D, 92:8 er
fresh D_2O under standard conditions: 83%, 90% D, 92:8 er

The reaction of 4DPAIPN (1.8 mg, 0.002 mmol), **1i** (104.2 mg, 0.4 mmol), **2a** (35.0 mg, 0.2 mmol), **S7** (16.9 mg, 0.03 mmol), toluene (1.5 mL) and D_2O (0.5 mL, recycled from a 0.6 mmol scale reaction) under room temperature afforded **4a** (73.3 mg, 84%, 80% D) (purified by chromatography on silica gel, eluent: petroleum ether/ethyl acetate = 5/1) as a white solid: 91:9 er (HPLC conditions: Chiralcel AD-H column, hexane/*i*-PrOH = 90/10, 1.0 mL/min, λ = 214 nm, t_R (major) = 7.6 min, t_R (minor) = 11.3 min); **^{1}H NMR** (400 MHz, $CDCl_3$) δ = 7.79-7.05 (m, 20

H), 4.68-4.50 (m, 0.20 H), 3.97 (d, J = 8.8 Hz, 1 H), 3.63 (d, J = 8.8 Hz, 1 H), 2.05 (d, J = 14.4 Hz, 1 H), 1.61 (d, J = 14.8 Hz, 1 H).

The reaction of 4DPAIPN (1.7 mg, 0.002 mmol), **1i** (104.1 mg, 0.4 mmol), **2a** (35.0 mg, 0.2 mmol), **S7** (17.0 mg, 0.03 mmol), toluene (1.5 mL) and D_2O (0.5 mL, recycled from a 3.0 mmol scale reaction) under room temperature afforded **4a** (73.3 mg, 84%, 83% D) (purified by chromatography on silica gel, eluent: petroleum ether/ethyl acetate = 5/1) as a white solid: 92:8 er (HPLC conditions: Chiralcel AD-H column, hexane/*i*-PrOH = 90/10, 1.0 mL/min, λ = 214 nm, t_R (major) = 7.7 min, t_R (minor) = 11.5 min); **^{1}H NMR** (400 MHz, $CDCl_3$) δ = 7.59-7.09 (m, 20 H), 4.64-4.54 (m, 0.17 H), 3.96 (d, J = 8.8 Hz, 1 H), 3.63 (d, J = 9.2 Hz, 1 H), 2.05 (d, J = 14.8 Hz, 1 H), 1.61 (d, J = 14.8 Hz, 1 H).

Synthesis of **4b:**

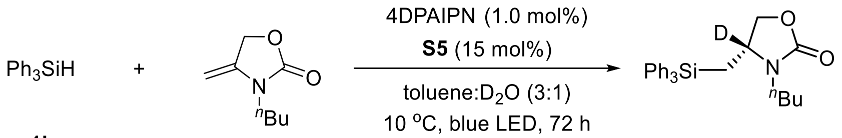

1i (2.0 equiv) **2j** **4b**, 61%, 91% D 82:18 er

The reaction of 4DPAIPN (1.7 mg, 0.002 mmol), **1i** (106.2 mg, 0.4 mmol), **2j** (29.9 mg, 0.2 mmol), **S5** (17.7 mg, 0.03 mmol), toluene (1.5 mL) and D_2O (0.5 mL) afforded **4b** (50.8 mg, 61%, 91% D) (purified by chromatography on silica gel, eluent: petroleum ether/ethyl acetate = 5/1) as a white solid: 82:18 er (HPLC conditions: Chiralcel AD-H column, hexane/*i*-PrOH = 96/4, 1.0 mL/min, λ = 214 nm, t_R (minor) = 9.5 min, t_R (major) = 11.6 min); $[\alpha]_D^{25}$ = -17.3 (c = 0.41, $CHCl_3$); **m.p.** 83-84 °C; **^{1}H NMR** (400 MHz, $CDCl_3$) δ = 7.61-7.49 (m, 6 H), 7.49-7.32 (m, 9 H), 4.04-3.92 (m, 0.09 H), 3.76 (d, J = 8.8 Hz, 1 H), 3.54-3.40 (m, 2 H), 3.19-3.00 (m, 1 H), 2.06 (d, J = 14.4 Hz, 1 H), 1.58-1.22 (m, 5 H), 0.91 (t, J = 7.2 Hz, 3 H); **^{13}C NMR** (100 MHz, $CDCl_3$); 157.7, 135.3, 133.2, 130.1, 128.3, 68.7, 52.1 (t, $J_{C\text{-}D}$ = 21.8 Hz), 40.7, 29.3, 19.9, 17.3, 13.7; **HRMS** (ESI) calcd for $C_{26}H_{28}DNNaO_2Si$ [M+Na^+]: 439.1923, found: 439.1921.

Synthesis of **4c:**

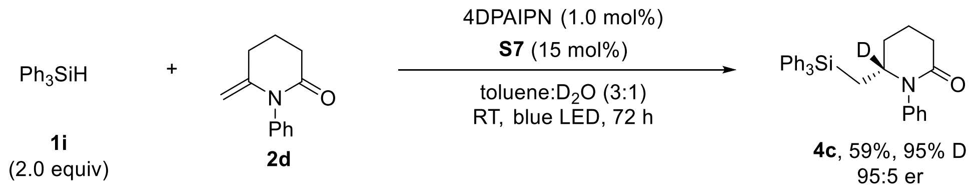

The reaction of 4DPAIPN (0.8 mg, 0.001 mmol), **1i** (52.4 mg, 0.24 mmol), **2d** (18.9 mg, 0.1 mmol), **S7** (8.9 mg, 0.015 mmol), toluene (0.75 mL) and D_2O (0.25 mL) under room temperature afforded **4c** (26.6 mg, 59%, 95% D) (eluent: petroleum ether/ethyl acetate = 5/1) as an oil: 95:5 er (HPLC conditions: Chiralcel AD-H column, hexane/*i*-PrOH = 80/20, 0.5 mL/min, λ = 214 nm, t_R (major) = 8.9 min, t_R (minor) = 9.8 min); $[\alpha]_D^{25}$ = -2.4 (*c* = 0.13, $CHCl_3$); **^{1}H NMR** (400 MHz, $CDCl_3$) δ = 7.52-7.20 (m, 18 H), 7.18-7.08 (m, 2 H), 4.15-4.00 (m, 0.05 H), 2.58-2.47 (m, 1 H), 2.46-2.33 (m, 1 H), 2.02-1.54 (m, 5 H), 1.53-1.41 (m, 1 H); **^{13}C NMR** (100 MHz, $CDCl_3$) δ = 170.0, 141.4, 135.4, 133.9, 129.7, 129.3, 128.4, 128.0, 127.1, 32.5, 29.1, 18.1, 17.5; **HRMS** (ESI) calcd for $C_{30}H_{28}DNNaOSi$ [M+Na$^+$]: 471.1973, found: 471.1975.

Synthesis of **4d:**

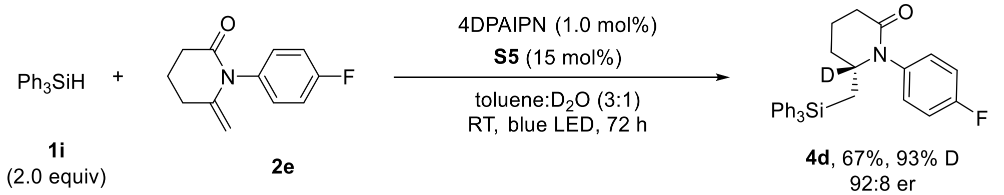

The reaction of 4DPAIPN (1.0 mg, 0.001 mmol), **1i** (52.3 mg, 0.24 mmol), **2e** (20.5 mg, 0.1 mmol), **S5** (9.0 mg, 0.015 mmol), toluene (0.75 mL) and D_2O (0.25 mL) under room temperature afforded **4d** (31.4 mg, 67%, 93% D) (eluent: petroleum ether/ethyl acetate = 1/1) as an oil: 92:8 er (HPLC conditions: Chiralcel OD-H column, hexane/*i*-PrOH = 80/20, 0.5 mL/min, λ = 214 nm, t_R (major) = 11.8 min, t_R (minor) = 12.7 min); $[\alpha]_D^{25}$ = -10.6 (*c* = 0.11, $CHCl_3$); **^{1}H NMR** (400 MHz, $CDCl_3$) δ = 7.47-7.26 (m, 15 H), 7.13-7.01 (m, 4 H), 4.07-3.96 (m, 0.07 H), 2.58-2.32 (m, 2 H), 1.98-1.73 (m, 3 H), 1.73-1.43 (m, 3 H); **^{13}C NMR** (100 MHz, $CDCl_3$) δ = 170.2, 135.4, 133.8, 130.1, 130.0, 129.7, 128.0, 116.2, 116.0, 32.5, 29.2, 18.3, 17.6, **^{19}F NMR** (376 MHz, $CDCl_3$) δ = -114.9; **HRMS** (ESI) calcd for $C_{30}H_{27}DFNNaOSi$ [M+Na$^+$]: 489.1879, found: 489.1879.

Synthesis of **4e:**

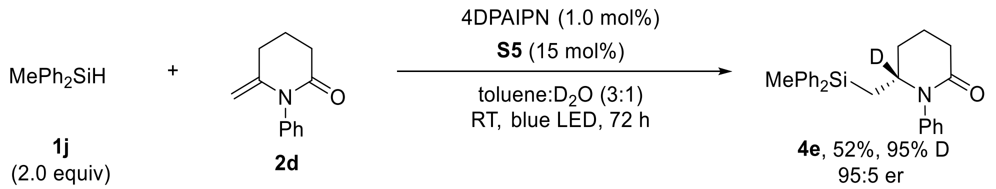

The reaction of 4DPAIPN (0.9 mg, 0.001 mmol), **1j** (40.6 mg, 0.2 mmol), **2d** (18.8 mg, 0.1 mmol), **S5** (8.9 mg, 0.015 mmol), toluene (0.75 mL) and D_2O (0.25 mL) under room temperature afforded **4e** (20.1 mg, 52%, 95% D) (eluent: petroleum ether/ethyl acetate = 2/1) as an colorless oil: 95:5 er (HPLC conditions: Chiralcel OD-H column, hexane/*i*-PrOH = 90/10, 0.5 mL/min, λ = 214 nm, t_R (major) = 25.0 min, t_R (minor) = 30.0 min); $[\alpha]_D^{25}$ = -16.8 (*c* = 0.17, $CHCl_3$); **^{1}H NMR** (400 MHz, $CDCl_3$) δ = 7.45-7.19 (m, 13 H), 7.17-7.09 (m, 2 H), 4.04-3.88 (m, 0.05 H), 2.63-2.36 (m, 2 H), 2.00-1.84 (m, 2 H), 1.78-1.47 (m, 3 H), 1.41-1.30 (m, 1 H), 0.50 (s, 3 H); **^{13}C NMR** (100 MHz, $CDCl_3$) δ = 169.9, 141.4, 136.4, 135.5, 134.2, 134.1, 129.44, 129.41, 129.2, 128.3, 127.97, 127.95, 127.1, 32.6, 29.6, 19.7, 17.8, -3.8; **HRMS** (ESI) calcd for $C_{25}H_{26}DNNaOSi$ [M+Na$^+$]: 409.1817, found: 409.1716.

Synthesis of **4f:**

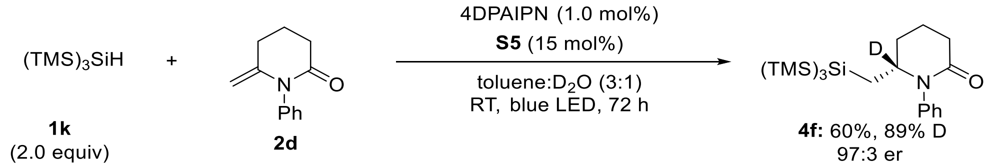

The reaction of 4DPAIPN (1.0 mg, 0.001 mmol), **1k** (50.2 mg, 0.2 mmol), **2d** (18.7 mg, 0.1 mmol), **S5** (8.9 mg, 0.015 mmol), toluene (0.75 mL) and D_2O (0.25 mL) under room temperature afforded **4f** (26.3 mg, 60%, 89% D) (eluent: petroleum ether/ethyl acetate = 2/1) as a white solid: 97:3 er (HPLC conditions: Chiralcel OD-H column, hexane/*i*-PrOH = 90/10, 0.5 mL/min, λ = 214 nm, t_R (major) = 9.8 min, t_R (minor) = 10.6 min); $[\alpha]_D^{25}$ = -58.5 (*c* = 0.19, $CHCl_3$); **m.p.** 108-112 °C; **^{1}H NMR** (400 MHz, $CDCl_3$) δ = 7.45-7.34 (m, 2 H), 7.31-7.22 (m, 1 H), 7.19-7.09 (m, 2 H), 3.90-3.81 (m, 0.11 H), 2.64-2.44 (m, 2 H), 2.20-2.08 (m, 1 H), 2.07-1.74 (m, 3 H), 1.25-1.16 (m, 1 H), 1.04-0.93 (m, 1 H), 0.06 (s, 27 H); **^{13}C NMR** (100 MHz, $CDCl_3$) δ = 169.7, 141.7, 129.1, 128.3, 127.1, 32.6, 29.2, 17.9, 13.2, 1.1; **HRMS** (ESI) calcd for $C_{21}H_{40}DNNaO_2Si_4$ [M+Na$^+$]: 459.2220, found: 459.2221.

Synthesis of **4g:**

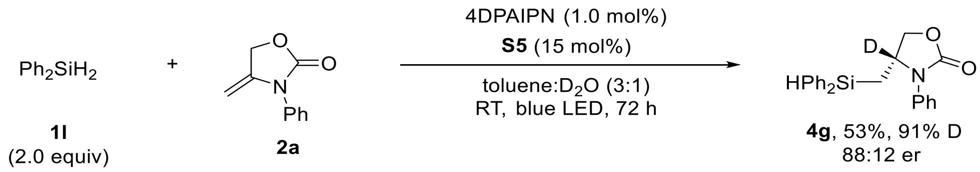

The reaction of 4DPAIPN (0.9 mg, 0.001 mmol), **1l** (37 μL, 0.2 mmol), **2a** (17.4 mg, 0.1 mmol), **S5** (8.6 mg, 0.015 mmol), toluene (0.75 mL) and D_2O (0.25 mL) under room temperature afforded **4g** (19.0 mg, 53%, 91% D) (purified by chromatography on silica gel, eluent: petroleum ether/ethyl acetate = 5/1) as a white solid: 88:12 er (HPLC conditions: Chiralcel AD-H column, hexane/*i*-PrOH = 80/20, 1.0 mL/min, λ = 214 nm, t_R (major) = 6.3 min, t_R (minor) = 7.6 min); $[\alpha]_D^{25}$ = -2.7 (*c* = 0.09, $CHCl_3$); **m.p.** 103-105 °C; **^{1}H NMR** (400 MHz, $CDCl_3$) δ = 7.75-6.99 (m, 15 H), 4.97-4.85 (m, 1 H), 4.60-4.44 (m, 0.09 H), 4.35 (d, *J* = 8.8 Hz, 1 H), 3.98 (d, *J* = 8.8 Hz, 1 H), 1.89-1.75 (m, 1 H), 1.47-1.37 (m, 1 H); **^{13}C NMR** (100 MHz, $CDCl_3$) δ = 155.5, 136.1, 135.0, 134.94, 133.91, 132.0, 131.8, 130.5, 130.4, 129.2, 128.5, 128.3, 125.3, 122.2, 122.0, 68.2, 17.1; **HRMS** (ESI) calcd for $C_{22}H_{20}DNNaO_2Si$ [M+Na$^+$]: 383.1297, found: 383.1298.

Synthesis of **4h:**

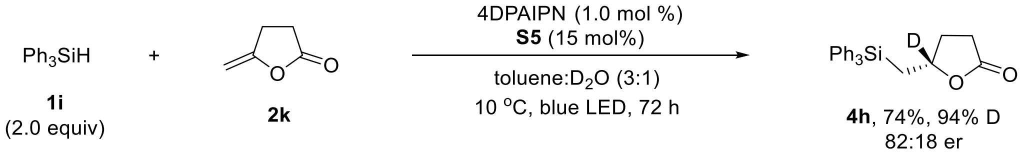

The reaction of 4DPAIPN (1.6 mg, 0.002 mmol), **1i** (105.0 mg, 0.4 mmol), **2k** (13.2 mg, 0.2 mmol), **S5** (17.5 mg, 0.03 mmol), toluene (1.5 mL) and D_2O (0.5 mL) afforded **4h** (34.7 mg, 74%, 94% D) (purified by chromatography on silica gel, eluent: petroleum ether/ethyl acetate = 5/1) as a white solid: 82:18 er (HPLC conditions: Chiralcel AD-H column, hexane/*i*-PrOH = 96/4, 1.0 mL/min, λ = 214 nm, t_R (major) = 10.2 min, t_R (minor) = 11.2 min); $[\alpha]_D^{25}$ = +5.53 (*c* = 0.36, $CHCl_3$); **m.p.** 85-86 °C; **^{1}H NMR** (400 MHz, $CDCl_3$) δ = 7.64-7.48 (m, 6 H), 7.48-7.25 (m, 9 H), 4.78-4.67 (m, 0.06 H), 2.47-2.29 (m, 2 H), 2.20 (d, *J* = 14.4 Hz, 1 H), 2.00-1.90 (m, 1 H), 1.78 (d, *J* = 14.8 Hz, 1 H), 1.71-1.57 (m, 1 H); **^{13}C NMR** (100 MHz, $CDCl_3$) δ = 176.8, 135.5, 133.6, 129.8, 128.1, 79.0 (t, J_{C-D} = 22.7 Hz), 30.6, 29.4, 21.3; **HRMS** (ESI) calcd for $C_{23}H_{21}DNaO_2Si$ [M+Na$^+$]: 382.1344, found: 382.1343.

Synthesis of **4i:**

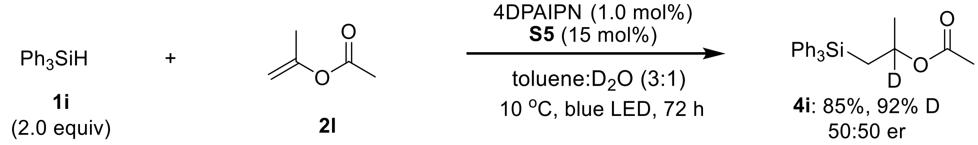

The reaction of 4DPAIPN (1.6 mg, 0.002 mmol), **1i** (104.3 mg, 0.4 mmol), **2l** (20.0 mg, 0.2 mmol), **S5** (16.8 mg, 0.03 mmol), toluene (1.5 mL) and D_2O (0.5 mL) afforded **4i** (61.8 mg, 85%, 92% D) (purified by chromatography on silica gel, eluent: petroleum ether/ethyl acetate = 5/1) as an oil: 50:50 er (HPLC conditions: Chiralcel OJ-H column, hexane/*i*-PrOH = 99/1, 1.0 mL/min, λ = 214 nm, t_R (major) = 7.6 min, t_R (minor) = 11.2 min); ^{1}**H NMR** (400 MHz, $CDCl_3$) δ = 7.65-7.47 (m, 6 H), 7.45-7.29 (m, 9 H), 5.21-5.09 (m, 0.08 H), 1.95 (d, J = 14.8 Hz, 1 H), 1.75-1.62 (m, 4 H), 1.19 (s, 3 H); ^{13}**C NMR** (100 MHz, $CDCl_3$) δ = 170.3, 135.6, 134.5, 129.5, 127.9, 68.8 (t, $J_{C\text{-}D}$ = 23.1 Hz), 23.4, 21.8, 21.0; **HRMS** (ESI) calcd for $C_{23}H_{23}DNaO_2Si$ [M+Na$^+$]: 384.1501, found: 384.1500.

Synthesis of **4j:**

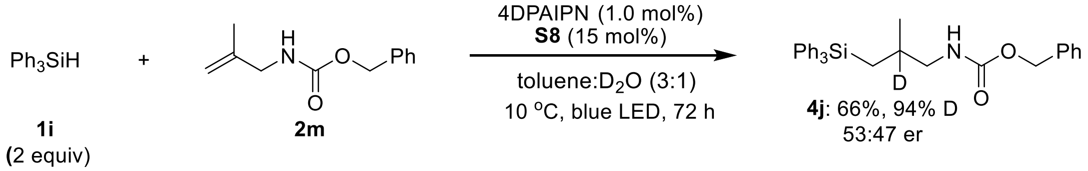

The reaction of 4DPAIPN (1.8 mg, 0.002 mmol), **1i** (104.2 mg, 0.4 mmol), **2m** (40.7 mg, 0.2 mmol), **S8** (22.1 mg, 0.03 mmol), toluene (1.5 mL) and D_2O (0.5 mL) afforded **4j** (61.6 mg, 66%, 94% D) (purified by chromatography on silica gel, eluent: petroleum ether/ethyl acetate = 5/1) as an oil: 53:47 er (HPLC conditions: Chiralcel AD-H column, hexane/*i*-PrOH = 96/4, 1.0 mL/min, λ = 214 nm, t_R (major) = 12.9 min, t_R (minor) = 18.2 min); ^{1}**H NMR** (400 MHz, $CDCl_3$) δ = 7.62-7.16 (m, 20 H), 5.06 (s, 2 H), 4.67 (s, 1 H), 3.15-2.95 (m, 2 H), 1.97-1.83 (m, 0.06 H), 1.49 (d, J = 15.2 Hz, 1 H), 1.24 (d, J = 14.8 Hz, 1 H), 0.80 (s, 3 H); ^{13}**C NMR** (100 MHz, $CDCl_3$) δ = 156.5, 136.6, 135.6, 135.1, 129.5, 128.5, 128.1, 127.9, 66.6, 49.7, 20.5, 18.2; **HRMS** (ESI) calcd for $C_{30}H_{30}DNNaO_2Si$ [M+Na$^+$]: 489.2079, found: 489.2078.

Synthesis of **5a:**

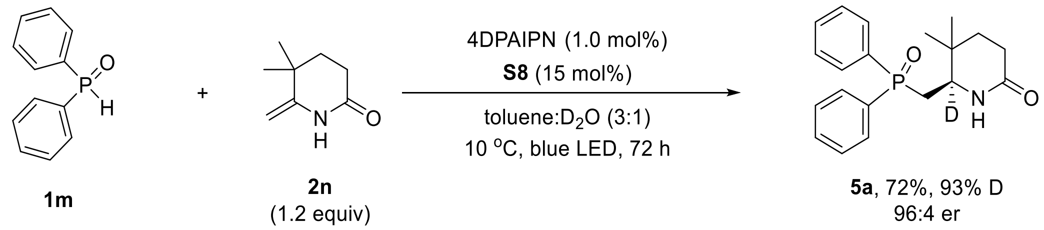

1m **2n** (1.2 equiv) **5a**, 72%, 93% D 96:4 er

The reaction of 4DPAIPN (1.5 mg, 0.002 mmol), **1m** (40.6 mg, 0.2 mmol), **2n** (33.8 mg, 0.24 mmol), **S8** (21.7 mg, 0.03 mmol), toluene (1.5 mL) and D_2O (0.5 mL) afforded **5a** (42.9 mg, 72%, 93% D) (purified by chromatography on silica gel, eluent: dichloromethane/methanol = 30/1) as a white solid: 96:4 er, (HPLC conditions: Chiralcel AD-H column, hexane/*i*-PrOH = 65/35, 0.5 mL/min, λ = 214 nm, t_R (major) = 14.0 min, t_R (minor) =15.1 min); $[\alpha]_D^{25}$ = -80.6 (*c* = 0.21, $CHCl_3$); **m.p.** 172-173 ℃; **^{1}H NMR** (400 MHz, $CDCl_3$) δ = 7.87-7.65 (m, 4 H), 7.64-7.45 (m, 6 H), 7.34 (brs, 1 H), 3.37 (t, *J* = 11.2 Hz, 0.07 H), 2.44-2.09 (m, 4 H), 1.64-1.49 (m, 2 H), 1.02-0.84 (m, 6 H); **^{13}C NMR** (100 MHz, $CDCl_3$) δ = 170.6, 133.1 (d, $J_{C\text{-}P}$ = 99.9 Hz), 132.24 (d, $J_{C\text{-}P}$ = 2.6 Hz), 132.19 (d, $J_{C\text{-}P}$ = 2.8 Hz), 131.1 (d, $J_{C\text{-}P}$ = 9.0 Hz), 130.4 (d, $J_{C\text{-}P}$ = 98.1 Hz), 130.3 (d, $J_{C\text{-}P}$ = 9.6 Hz), 128.9 (d, $J_{C\text{-}P}$ = 7.4 Hz), 128.8 (d, $J_{C\text{-}P}$ = 7.6 Hz), 34.9, 32.0 (d, $J_{C\text{-}P}$ = 11.6 Hz), 30.1 (d, $J_{C\text{-}P}$ = 70.1 Hz), 28.2, 26.7, 18.6; **^{31}P NMR** (162 MHz, $CDCl_3$) δ = 33.4; **HRMS** (ESI) calcd for $C_{20}H_{23}DNNaO_2P$ [M+Na$^+$]: 365.1500, found: 365.1497.

Synthesis of **5b:**

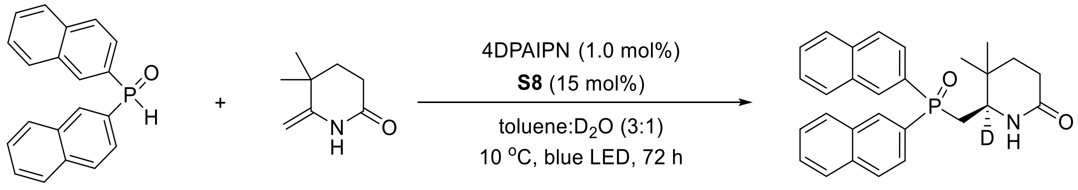

1n **2n** (1.2 equiv) **5b**, 70%, 90% D 95:5 er

The reaction of 4DPAIPN (1.7 mg, 0.002 mmol), **1n** (60.4 mg, 0.2 mmol), **2n** (34.6 mg, 0.24 mmol), **S8** (21.8 mg, 0.03 mmol), toluene (1.5 mL) and D_2O (0.5 mL) afforded **5b** (63.2 mg, 70%, 90% D) (purified by chromatography on silica gel, eluent: dichloromethane/methanol = 20/1) as a white solid: 95:5 er, (HPLC conditions: Chiralcel OD-H column, hexane/*i*-PrOH = 80/20, 1 mL/min, λ = 214 nm, t_R (minor) = 71.6 min, t_R (major) = 82.0 min); $[\alpha]_D^{25}$ = -104.4 (*c* = 0.24, $CHCl_3$); **m.p.** 209-211 ℃; **^{1}H NMR** (400 MHz, $CDCl_3$) δ = 8.58 (d, *J* = 13.2 Hz, 1 H), 8.32 (d, *J* = 13.6 Hz, 1 H), 8.01-7.83 (m, 6 H), 7.76-7.52 (m, 6 H), 7.46 (brs, 1 H), 3.43 (t, *J* =

11.2 Hz, 0.10 H), 2.62-2.51 (m, 1 H), 2.42-2.22 (m, 3 H), 1.58-1.45 (m, 2 H), 1.00 (s, 3 H), 0.92 (s, 3 H); **^{13}C NMR** (100 MHz, $CDCl_3$) δ = 170.6, 134.8 (d, $J_{C\text{-}P}$ = 2.4 Hz), 134.7 (d, $J_{C\text{-}P}$ = 2.5 Hz), 134.1 (d, $J_{C\text{-}P}$ = 7.8 Hz), 132.6 (d, $J_{C\text{-}P}$ = 12.6 Hz), 132.4 (d, $J_{C\text{-}P}$ = 13.1 Hz), 132.3 (d, $J_{C\text{-}P}$ = 9.1 Hz), 130.2 (d, $J_{C\text{-}P}$ = 100.4 Hz), 129.1-128.7 (m), 128.5, 128.4, 127.9-127.8 (m), 127.4 (d, $J_{C\text{-}P}$ = 98.4 Hz), 127.3, 127.2, 125.3-125.0 (m), 35.0, 32.1 (d, $J_{C\text{-}P}$ = 11.7 Hz), 30.0 (d, $J_{C\text{-}P}$ = 70.3 Hz), 28.3, 26.8, 18.7; **^{31}P NMR** (162 MHz, $CDCl_3$) δ = 33.7; **HRMS** (ESI) calcd for $C_{28}H_{28}DNO_2P$ [M+H$^+$]: 443.1993, found: 443.1995.

Synthesis of **5c:**

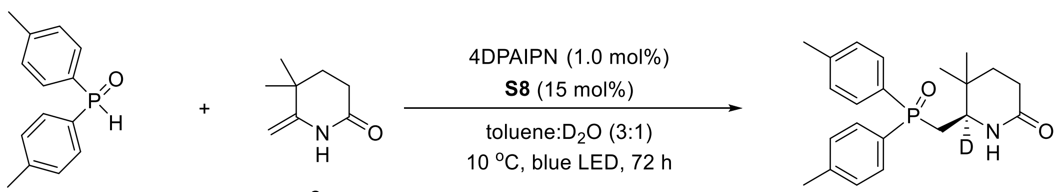

1o + **2n** (1.2 equiv) → **5c**, 75%, 94% D, 97:3 er

The reaction of 4DPAIPN (1.9 mg, 0.002 mmol), **1o** (46.1 mg, 0.2 mmol), **2n** (36.6 mg, 0.24 mmol), **S8** (22.0 mg, 0.03 mmol), toluene (1.5 mL) and D_2O (0.5 mL) afforded **5c** (55.5 mg, 75%, 94% D) (purified by chromatography on silica gel, eluent: dichloromethane/methanol = 30/1) as a white solid: 97:3 er (HPLC conditions: Chiralcel AD-H column, hexane/*i*-PrOH = 60/40, 0.5 mL/min, λ = 214 nm, t_R (major) = 15.5 min, t_R (minor) = 24.9 min); $[\alpha]_D^{25}$ = -76.0 (*c* = 0.24, $CHCl_3$); **m.p.** 115-118 ℃; **^{1}H NMR** (400 MHz, $CDCl_3$) δ = 7.72-7.50 (m, 4 H), 7.44-7.16 (m, 5 H), 3.44-3.25 (m, 0.06 H), 2.49-2.21 (m, 9 H), 2.19-2.03 (m, 1 H), 1.63-1.47 (m, 2 H), 0.95 (s, 3 H), 0.90 (s, 3 H); **^{13}C NMR** (100 MHz, $CDCl_3$) δ = 170.6, 142.74-142.61 (m), 131.1 (d, $J_{C\text{-}P}$ = 9.3 Hz), 130.3 (d, $J_{C\text{-}P}$ = 9.9 Hz), 130.1 (d, $J_{C\text{-}P}$ = 102.4 Hz), 129.7-129.3 (m), 127.3 (d, $J_{C\text{-}P}$ = 100.5 Hz), 56.7 (t, $J_{C\text{-}D}$ = 24.7 Hz), 35.0, 32.0 (d, $J_{C\text{-}P}$ = 11.6 Hz), 30.3 (d, $J_{C\text{-}P}$ = 70.3 Hz), 28.2, 26.8, 21.6-21.4 (m), 18.7; **^{31}P NMR** (162 MHz, $CDCl_3$) δ = 33.7; **HRMS** (ESI) calcd for $C_{22}H_{28}DNO_2P$ [M+H$^+$]: 371.1993, found: 371.1992.

Synthesis of **5d:**

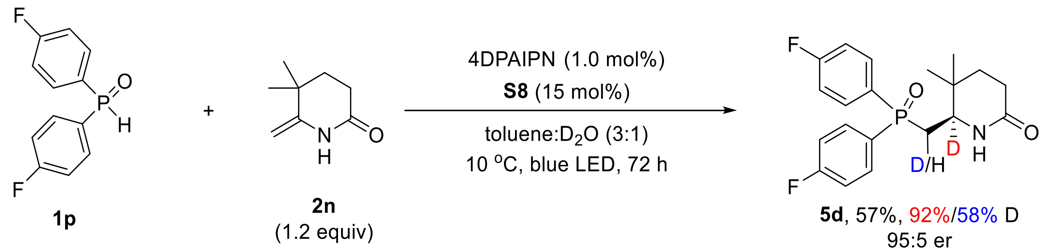

The reaction of 4DPAIPN (2.0 mg, 0.002 mmol), **1p** (47.4 mg, 0.2 mmol), **2n** (34.2 mg, 0.24 mmol), **S8** (21.7 mg, 0.03 mmol), toluene (1.5 mL) and D_2O (0.5 mL) afforded **5d** (43.3 mg, 57%, 92%/58% D) (purified by chromatography on silica gel, eluent: dichloromethane/methanol = 30/1) as a white solid: 95:5 er (HPLC conditions: Chiralcel AS-H column, hexane/*i*-PrOH = 80/20, 0.5 mL/min, λ = 214 nm, t_R (minor) = 22.2 min, t_R (major) = 32.0 min); $[\alpha]_D^{25}$ = -66.8 (*c* = 0.40, $CHCl_3$); **m.p.** 150-152 ℃; **^{1}H NMR** (400 MHz, $CDCl_3$) δ = 7.91-7.58 (m, 4 H), 7.37-7.09 (m, 5 H), 3.39-3.3.26 (m, 0.08 H), 2.46-2.24 (m, 2.42 H), 2.23-2.10 (m, 1 H), 1.67-1.49 (m, 2 H), 1.03-0.80 (m, 6 H); **^{13}C NMR** (100 MHz, $CDCl_3$) δ = 170.7, 166.5 (dd, J_1 = 10.8 Hz, J_2 = 3.3 Hz), 163.9 (dd, J_1 = 10.6 Hz, J_2 = 3.3 Hz), 133.7-133.4 (m), 132.8 (dd, J_1 = 11.1 Hz, J_2 = 8.9 Hz), 128.9 (dd, J_1 = 102.9 Hz, J_2 = 3.2 Hz), 126.1 (dd, J_1 = 101.4 Hz, J_2 = 3.1 Hz), 116.8-116.2 (m), 34.9, 32.0 (d, $J_{C\text{-}P}$ = 11.7 Hz), 30.4 (d, $J_{C\text{-}P}$ = 71.3 Hz), 28.2, 26.7, 18.7; **^{19}F NMR** (376 MHz, $CDCl_3$) δ = -105.47, -105.48; **^{31}P NMR** (162 MHz, $CDCl_3$) δ = 32.4; **HRMS** (ESI) calcd for $C_{20}H_{20}D_2F_2NNaO_2P$ [M+Na$^+$]: 402.1374, found: 402.1370.

Synthesis of **5e:**

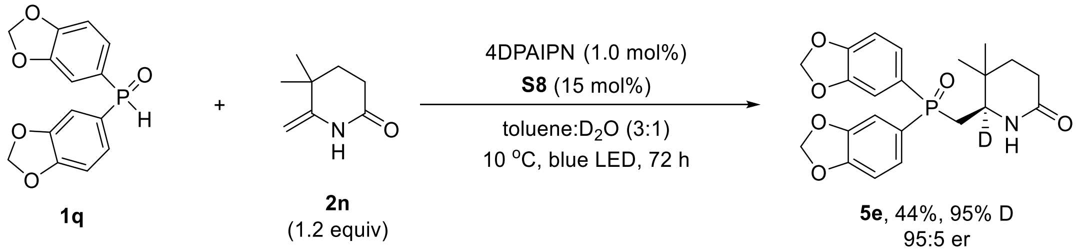

The reaction of 4DPAIPN (1.9 mg, 0.002 mmol), **1q** (58.0 mg, 0.2 mmol), **2n** (34.7 mg, 0.2 mmol), **S8** (21.6 mg, 0.03 mmol), toluene (1.5 mL) and D_2O (0.5 mL) afforded **5e** (37.5 mg, 44%, 95% D) (purified by chromatography on silica gel, eluent: dichloromethane/methanol = 30/1) as a white solid: 95:5 er (HPLC conditions: Chiralcel AD-H column, hexane/*i*-PrOH = 70/30, 1 mL/min, λ = 214 nm, t_R (major) = 18.6 min, t_R (minor) = 22.4 min); $[\alpha]_D^{25}$ = -71.5 (*c* =

0.22, $CHCl_3$); **m.p.** 185-186 ℃; **^{1}H NMR** (400 MHz, $CDCl_3$) δ = 7.36-7.03 (m, 5 H), 7.01-6.83 (m, 2 H), 6.20-5.87 (m, 4 H), 3.37 (t, *J* = 11.2 Hz, 0.05 H), 2.42-2.19 (m, 3 H), 2.15-1.94 (m, 1 H), 1.66-1.50 (m, 2 H), 1.01-0.82 (m, 6 H); **^{13}C NMR** (100 MHz, $CDCl_3$) δ = 170.7, 151.1 (d, J_{C-P} = 2.9 Hz), 151.0 (d, J_{C-P} = 2.9 Hz), 148.5-148.1 (m), 126.3(d, J_{C-P} = 104.6 Hz), 126.4 (d, J_{C-P} = 9.9 Hz), 125.6 (d, J_{C-P} = 10.6 Hz), 123.4 (d, J_{C-P} = 102.9 Hz), 110.3 (d, J_{C-P} = 11.9 Hz), 109.8 (d, J_{C-P} = 12.5 Hz), 109.1 (d, J_{C-P} = 9.0 Hz), 108.9 (d, J_{C-P} = 9.4 Hz), 101.8 (d, J_{C-P} = 6.6 Hz), 35.0, 32.0 (d, J_{C-P} = 11.7 Hz), 30.4 (d, J_{C-P} = 71.6 Hz), 28.2, 26.8, 18.7; **^{31}P NMR** (162 MHz, $CDCl_3$) δ = 33.8; **HRMS** (ESI) calcd for $C_{22}H_{23}DNNaO_6P$ [M+Na$^+$]: 453.1296, found: 453.1298.

Synthesis of **5f:**

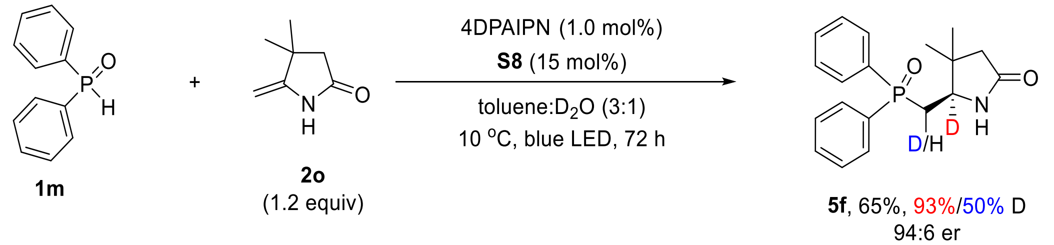

1m

2o
(1.2 equiv)

5f, 65%, 93%/50% D
94:6 er

The reaction of 4DPAIPN (2.0 mg, 0.002 mmol), **1m** (40.6 mg, 0.2 mmol), **2o** (34.9 mg, 0.24 mmol), **S8** (22.0 mg, 0.03 mmol), toluene (1.5 mL) and D_2O (0.5 mL) afforded **5f** (42.7 mg, 65%, 93%/50% D) (purified by chromatography on silica gel, eluent: dichloromethane/methanol = 30/1) as a white solid: 94:6 er (HPLC conditions: Chiralcel AD-H column, hexane/*i*-PrOH = 60/40, 0.5 mL/min, λ = 214 nm, t_R (major) = 12.1 min, t_R (minor) = 12.9 min); $[\alpha]_D^{25}$ = -78.1 (*c* = 0.28, $CHCl_3$); **m.p.** 149-153 ℃; **^{1}H NMR** (400 MHz, $CDCl_3$) δ = 7.85-7.66 (m, 4 H), 7.63-7.43 (m, 6 H), 6.70 (brs, 1 H), 3.54-3.44 (m, 0.07 H), 2.41-2.31 (m, 0.5 H), 2.30-1.94 (m, 3 H), 1.09-1.00 (m, 6 H); **^{13}C NMR** (100 MHz, $CDCl_3$) δ = 175.6, 132.8 (d, J_{C-P} = 99.8 Hz), 132.3-132.1 (m), 131.3, 130.8 (d, J_{C-P} = 9.1 Hz), 130.3 (d, J_{C-P} = 9.7 Hz), 129.0 (d, J_{C-P} = 6.2 Hz), 128.8 (d, J_{C-P} = 5.3 Hz), 45.6, 39.1 (d, J_{C-P} = 11.9 Hz), 29.6 (d, J_{C-P} = 70.7 Hz), 25.6, 22.6; **^{31}P NMR** (162 MHz, $CDCl_3$) δ = 32.4; **HRMS** (ESI) calcd for $C_{19}H_{20}D_2NNaO_2P$ [M+Na$^+$]: 352.1406, found: 352.1401.

Synthesis of **5g:**

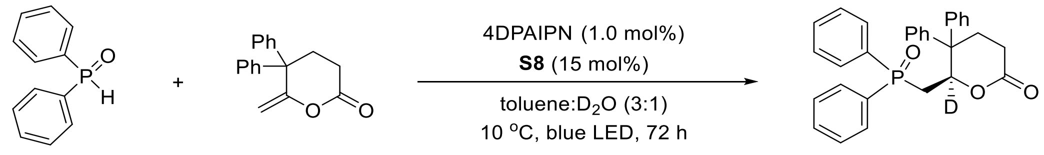

1m **2p** (1.2 equiv) **5g**, 62%, 96% D >99:1 er

The reaction of 4DPAIPN (1.7 mg, 0.002 mmol), **1m** (40.3 mg, 0.2 mmol), **2p** (63.7 mg, 0.24 mmol), **S8** (22.6 mg, 0.03 mmol), toluene (1.5 mL) and D_2O (0.5 mL) afforded **5g** (57.9 mg, 62%, 96% D) (purified by chromatography on silica gel, eluent: dichloromethane/methanol = 30/1) as a white solid: >99:1 er (HPLC conditions: Chiralcel AD-H column, hexane/*i*-PrOH = 70/30, 1 mL/min, λ = 214 nm, t_R (minor) = 11.6 min, t_R (major) = 14.7 min); $[\alpha]_D^{25}$ = -147.1 (*c* = 0.28, $CHCl_3$); **m.p.** 234-236 ℃; **^{1}H NMR** (400 MHz, $CDCl_3$) δ = 7.81-7.61 (m, 2 H), 7.59-7.02 (m, 18 H), 5.82 (t, *J* = 10.6 Hz, 0.04 H), 2.84-2.66 (m, 1 H), 2.65-2.37 (m, 3 H), 2.32-2.09 (m, 2 H); **^{13}C NMR** (100 MHz, $CDCl_3$) δ = 168.3, 143.2 (d, $J_{C\text{-}P}$ = 10.1 Hz), 133.1 (d, $J_{C\text{-}P}$ = 100.6 Hz), 131.95 (d, $J_{C\text{-}P}$ = 100.8 Hz), 131.86 (d, $J_{C\text{-}P}$ = 2.9 Hz), 131.78 (d, $J_{C\text{-}P}$ = 2.6 Hz), 131.0 (d, $J_{C\text{-}P}$ = 9.5 Hz), 130.5 (d, $J_{C\text{-}P}$ = 9.5 Hz), 128.9 (d, $J_{C\text{-}P}$ = 13.8 Hz), 128.6 (d, $J_{C\text{-}P}$ = 4.5 Hz), 128.5 (d, $J_{C\text{-}P}$ = 4.7 Hz), 127.9 (d, $J_{C\text{-}P}$ = 12.5 Hz), 127.1 (d, $J_{C\text{-}P}$ = 31.3 Hz), 48.5 (d, $J_{C\text{-}P}$ = 9.2 Hz), 33.5 (d, $J_{C\text{-}P}$ = 70.2 Hz), 27.9, 27.4; **^{31}P NMR** (162 MHz, $CDCl_3$) δ = 29.4; **HRMS** (ESI) calcd for $C_{30}H_{26}DNaO_3P$ [M+Na$^+$]: 490.1653, found: 490.1649.

Synthesis of **5h:**

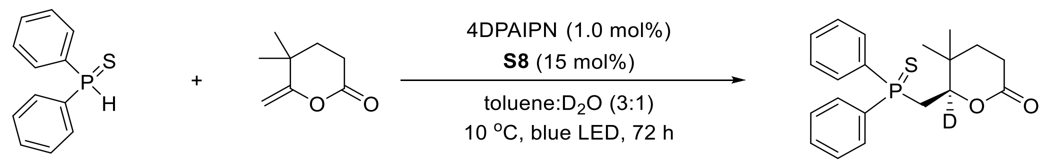

1r **2q** (1.2 equiv) **5h,** 80%, 81% D 89:11 er

The reaction of 4DPAIPN (1.9 mg, 0.002 mmol), **1r** (42.8 mg, 0.2 mmol), **2q** (33.0 mg, 0.24 mmol), **S8** (21.6 mg, 0.03 mmol), toluene (1.5 mL) and D_2O (0.5 mL) afforded **5h** (57.0 mg, 80%, 81% D) (purified by chromatography on silica gel, eluent: petroleum ether/ethyl acetate = 5/1) as a white solid: 89:11 er (HPLC conditions: Chiralcel OD-H column, hexane/*i*-PrOH = 88/12, 1 mL/min, λ = 214 nm, t_R (minor) = 9.1 min, t_R (major) = 9.9 min); $[\alpha]_D^{25}$ = -83.1 (*c* = 0.24, $CHCl_3$); **m.p.** 151-153 ℃; **^{1}H NMR** (400 MHz, $CDCl_3$) δ = 8.04-7.76 (m, 4 H), 7.62-7.33 (m, 6

H), 4.81 (dd, J_1 = 15.4 Hz, J_2 = 9.0 Hz, 0.19 H), 3.06-2.76 (m, 1 H), 2.57-2.24 (m, 3 H), 1.86-1.73 (m, 1 H), 1.66-1.54 (m, 1 H), 1.09-0.87 (m, 6 H); **^{13}C NMR** (100 MHz, $CDCl_3$) δ = 170.1, 133.9 (d, $J_{C\text{-}P}$ = 82.8 Hz), 131.8 (d, $J_{C\text{-}P}$ = 10.7 Hz), 131.6 (d, $J_{C\text{-}P}$ = 3.1 Hz), 131.5 (d, $J_{C\text{-}P}$ = 2.9 Hz), 131.4, 130.7 (d, $J_{C\text{-}P}$ = 10.0 Hz), 128.7 (d, $J_{C\text{-}P}$ = 12.1 Hz), 128.2 (d, $J_{C\text{-}P}$ = 12.6 Hz), 81.0-80.4 (m), 34.1 (d, $J_{C\text{-}P}$ = 55.9 Hz), 34.0, 32.4 (d, $J_{C\text{-}P}$ = 10.1 Hz), 27.2, 26.2, 19.8; **^{31}P NMR** (162 MHz, $CDCl_3$) δ = 42.6; **HRMS** (ESI) calcd for $C_{20}H_{23}DO_2PS$ [M+H$^+$]: 360.1292, found: 360.1291.

Synthesis of **5i:**

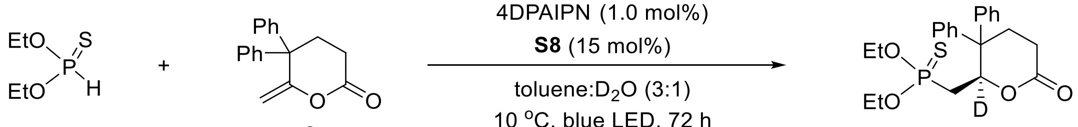

1s **2p** (1.2 equiv) **5i**, 62%, 97% D 96:4 er

The reaction of 4DPAIPN (2.0 mg, 0.002 mmol), **1s** (30.5 mg, 0.2 mmol), **2p** (65.0 mg, 0.24 mmol), **S8** (22.2 mg, 0.03 mmol), toluene (1.5 ml) and D_2O (0.5 mL) afforded **5i** (51.9 mg, 62%, 97% D) (purified by chromatography on silica gel, eluent: petroleum ether/ethyl acetate = 5/1) as a white solid: 96:4 er (HPLC conditions: Chiralcel IC-H column, hexane/*i*-PrOH = 80/20, 0.5 mL/min, λ = 214 nm, t_R (minor) = 15.6 min, t_R (major) = 17.8 min); $[\alpha]_D^{25}$ = -195.4 (*c* = 0.25, $CHCl_3$); **m.p.** 78-79 ℃; **^{1}H NMR** (400 MHz, $CDCl_3$) δ = 7.41-7.19 (m, 8 H), 7.17-7.09 (m, 2 H), 5.85-5.75 (m, 0.03 H), 4.27-3.90 (m, 4 H), 2.76-2.50 (m, 3 H), 2.34-2.15 (m, 2 H), 1.86-1.70 (m, 1 H), 1.29 (t, *J* = 7.0 Hz, 3 H), 1.24 (t, *J* = 7.2 Hz, 3 H); **^{13}C NMR** (100 MHz, $CDCl_3$) δ = 168.7, 143.2 (d, $J_{C\text{-}P}$ = 15.4 Hz), 128.9 (d, $J_{C\text{-}P}$ = 8.2 Hz), 127.3 (d, $J_{C\text{-}P}$ = 28.7 Hz), 127.1 (d, $J_{C\text{-}P}$ = 26.0 Hz), 63.7 (d, $J_{C\text{-}P}$ = 6.3 Hz), 61.9 (d, $J_{C\text{-}P}$ = 6.4 Hz), 48.0 (d, $J_{C\text{-}P}$ = 13.9 Hz), 37.8 (d, $J_{C\text{-}P}$ = 112.9 Hz), 27.5 (d, $J_{C\text{-}P}$ = 24.0 Hz), 16.2 (d, $J_{C\text{-}P}$ = 7.1 Hz), 16.0 (d, $J_{C\text{-}P}$ = 7.5 Hz); **^{31}P NMR** (162 MHz, $CDCl_3$) δ = 94.9; **HRMS** (ESI) calcd for $C_{22}H_{26}DNaO_4PS$ [M+Na$^+$]: 442.1323, found: 442.1320.

Synthesis of **5j:**

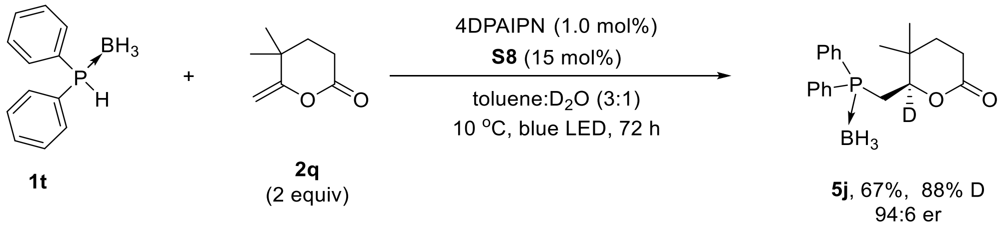

The reaction of 4DPAIPN (1.6 mg, 0.002 mmol), **1t** (37.0 mg, 0.19 mmol), **2q** (56.0 mg, 0.4 mmol), **S8** (21.6 mg, 0.03 mmol), toluene (1.5 ml) and D_2O (0.5 mL) afforded **5j** (42.3 mg, 67%, 88% D) (purified by chromatography on silica gel, eluent: petroleum ether/ethyl acetate = 2/1) as a white solid: 94:6 er (HPLC conditions: Chiralcel AD-H column, hexane/*i*-PrOH = 70/30, 0.8 mL/min, λ = 214 nm, t_R (minor) = 6.1 min, t_R (major) = 8.1 min); $[\alpha]_D^{25}$ = -32.4 ° (*c* = 0.17, $CHCl_3$); **m.p.** 117-119 ℃; **^{1}H NMR** (400 MHz, $CDCl_3$) δ = 7.88-7.75 (m, 2 H), 7.75-7.62 (m, 2 H), 7.57-7.33 (m, 6 H), 4.55-4.43 (m, 0.12 H), 2.70-2.57 (m, 1 H), 2.54-2.36 (m, 2 H), 2.29-2.18 (m, 1 H), 1.79-1.68 (m, 1 H), 1.65-1.54 (m, 1 H), 1.50-0.55 (m, 9 H); **^{13}C NMR** (100 MHz, $CDCl_3$) δ = 170.1, 132.9 (d, J_{C-P} = 9.8 Hz), 131.9 (d, J_{C-P} = 9.1 Hz), 131.5 (d, J_{C-P} = 2.5 Hz), 131.2 (d, J_{C-P} = 2.5 Hz), 130.0 (d, J_{C-P} = 57.4 Hz), 128.3 (d, J_{C-P} = 9.9 Hz), 128.5 (d, J_{C-P} = 10.4 Hz), 127.5 (d, J_{C-P} = 55.0 Hz), 81.4 (t, J_{C-D} = 23.4 Hz), 33.9, 32.6 (d, J_{C-P} = 8.8 Hz), 27.6 (d, J_{C-P} = 36.5 Hz), 27.2, 26.2, 19.4; **^{31}P NMR** (162 MHz, $CDCl_3$) δ = 16.2 (d, *J* = 77.8 Hz); **^{11}B NMR** (128 MHz, $CDCl_3$) δ = -39.4; **HRMS** (ESI) calcd for $C_{20}H_{25}BDNaO_2P$ [M+Na$^+$]: 364.1718, found: 364.1726.

Synthesis of **6a:**

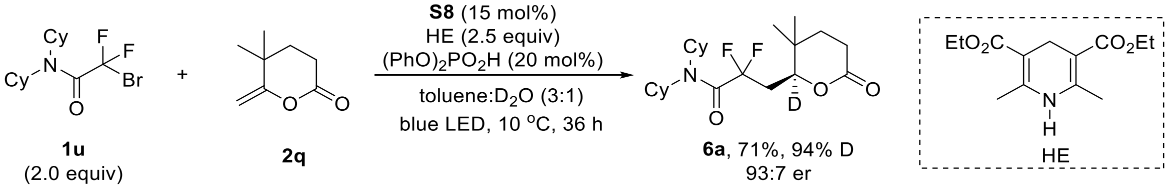

Typical procedure II: To an oven-dried 16 x 60 mm vial containing a dry Teflon stir bar were charged with Hantzsch Ester (HE, 63.2 mg, 0.25 mmol), **1u** (68.0 mg, 0.2 mmol), **S8** (10.9 mg, 0.015 mmol), and $(PhO)_2PO_2H$ (4.9 mg, 0.02 mmol), the vial was then taken into a N_2-filled glovebox where dry toluene (0.75 mL), **2q** (14.2 mg, 0.1 mmol), and D_2O (0.25 mL) were added sequentially. The vial was sealed with a cap and parafilm and then taken out of the glovebox. The vial was placed in a cooling station and a 30 W blue LED (λ = 441 nm) was then placed on the top of the cooling station (**Supplementary Figure 5**), which is connected to a chiller to maintain the temperature of the cooling water at 10 ℃. The reaction mixture was stirred at 10 ℃ under irradiation with a stirring rate of 400 r/min for 36 h. When the reaction is complete as monitored by TLC and GC, CH_2Cl_2 (10 mL) and H_2O (5 mL) were added, the organic layer was separated and the aqueous layer was extracted with CH_2Cl_2 (10 mL × 3). The combined organic layer was washed with brine and dried over anhydrous Na_2SO_4. After filtration and evaporation, the residue was purified by chromatography on silica gel (eluent: petroleum ether/ethyl acetate = 20/1 to 15/1 to 5/1) to afford **6a** (28.1 mg, 71%, 94% D) as a faint yellow oil: 93:7 er (HPLC conditions: Chiralcel AD-H column, hexane/*i*-PrOH = 90/10, 0.5 mL/min, λ = 214 nm, t_R (major) = 10.0 min, t_R (minor) = 10.9 min); $[\alpha]_D^{25}$ = -44.8 (*c* = 0.16, $CHCl_3$); **^{1}H NMR** (400 MHz, $CDCl_3$) δ = 4.43 (dd, J_1 = 9.2 Hz, J_2 = 1.2 Hz, 0.06 H), 4.02 (tt, J_1 = 11.6 Hz, J_2 = 3.5 Hz, 1 H), 3.01 (tt, J_1 = 11.8 Hz, J_2 = 3.8 Hz, 1 H), 2.66-2.26 (m, 6 H), 1.91-1.02 (m, 23 H), 0.99 (s, 3 H); **^{13}C NMR** (100 MHz, $CDCl_3$) δ = 170.8, 162.0 (t, J_{C-F} = 27.1 Hz), 118.9 (t, J_{C-F} = 257.1 Hz), 80.6 (t, J_{C-D} = 24.0 Hz), 57.1, 36.3-35.6 (m), 34.11, 34.08, 31.9, 31.8, 30.9, 29.0, 28.9, 27.1, 26.4, 26.3, 26.23, 26.20, 25.6, 25.5, 25.22, 25.17, 19.3; **^{19}F NMR** (376 MHz, $CDCl_3$) δ = -97.8 (d, *J* = 279.4 Hz), -100.4 (d, *J* = 279.0 Hz); **HRMS** (ESI) calcd for $C_{22}H_{34}DF_2NNaO_3$ [M+Na$^+$]: 423.2540, found: 423.2537.

The following compounds **6b-6f** were prepared according to the above **Typical Procedure II** unless otherwise stated. All the racemic samples for HPLC measurement were also prepared according to this procedure but using CySH instead of the thiol catalyst **S8**.

Synthesis of **6b:**

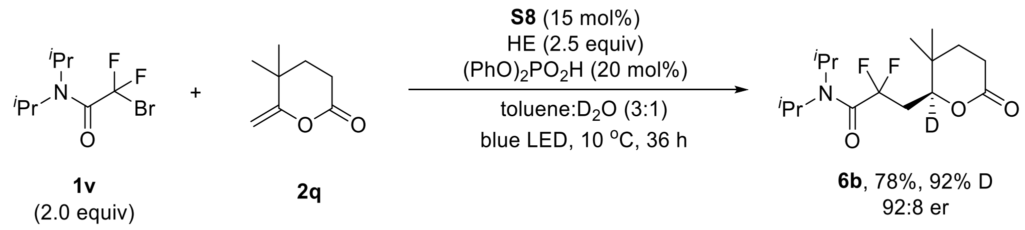

The reaction of HE (63.4 mg, 0.25 mmol), **1v** (51.8 mg, 0.2 mmol), **S8** (10.9 mg, 0.015 mmol), $(PhO)_2PO_2H$ (5.1 mg, 0.02 mmol), toluene (0.75 mL), **2q** (14.4 mg, 0.1 mmol) and D_2O (0.25 mL) afforded **6b** (25.6 mg, 78%, 92% D) (eluent: petroleum ether/ethyl acetate = 20/1 to 10/1 to 5/1) as a faint yellow oil: 92:8 er (HPLC conditions: Chiralcel OD-H column, hexane/*i*-PrOH = 90/10, 0.5 mL/min, λ = 214 nm, t_R (major) = 10.6 min, t_R (minor) = 11.4 min); $[\alpha]_D^{25}$ = -40.1 (c = 0.18, $CHCl_3$); **^{1}H NMR** (400 MHz, $CDCl_3$) δ = 4.56-4.40 (m, 1.08 H), 3.56-3.42 (m, 1 H), 2.66-2.44 (m, 3 H), 2.44-2.27 (m, 1 H), 1.86-1.74 (m, 1 H), 1.73-1.61 (m, 1 H), 1.45-1.37 (m, 6 H), 1.26-1.18 (m, 6 H), 1.05 (s, 3 H), 0.99 (s, 3 H); **^{13}C NMR** (100 MHz, $CDCl_3$) δ = 170.7, 161.7 (t, $J_{C\text{-}F}$ = 27.3 Hz), 118.7 (t, $J_{C\text{-}F}$ = 255.6 Hz), 48.4 (t, J = 7.5 Hz), 46.9, 35.9 (t, J = 22.9 Hz), 34.1, 31.8, 27.1, 26.2, 20.5, 20.4, 19.81, 19.78, 19.3; **^{19}F NMR** (376 MHz, $CDCl_3$) δ = -97.8 (d, J = 279.4 Hz), -100.6 (d, J = 279.4 Hz); **HRMS** (ESI) calcd for $C_{16}H_{26}DF_2NNaO_3$ [M+Na$^+$]: 343.1914, found: 343.1914.

Synthesis of **6c:**

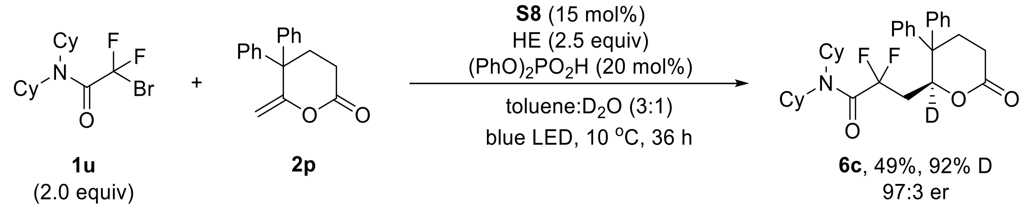

The reaction of HE (63.4 mg, 0.25 mmol), **1u** (67.8 mg, 0.2 mmol), **S8** (10.8 mg, 0.015 mmol), $(PhO)_2PO_2H$ (5.3 mg, 0.02 mmol), toluene (0.75 mL), **2p** (26.4 mg, 0.1 mmol) and D_2O (0.25 mL) afforded **6c** (25.8 mg, 49%, 92% D) (eluent: petroleum ether/ethyl acetate = 15/1 to 8/1) as a faint yellow oil: 97:3 er (HPLC conditions: Chiralcel AD-H column, hexane/*i*-PrOH = 95/5, 1 mL/min, λ = 214 nm, t_R (major) = 9.5 min, t_R (minor) = 10.6 min); $[\alpha]_D^{25}$ = -113.3 (c = 0.16, $CHCl_3$); **^{1}H NMR** (400 MHz, $CDCl_3$) δ = 7.40-7.25 (m, 6 H), 7.25-7.13 (m, 4 H), 5.70-5.64 (m, 0.08 H), 4.06-3.90 (m, 1 H), 3.05-2.90 (m, 1 H), 2.88-2.75 (m, 1 H), 2.72-2.49 (m, 3 H),

2.40-1.96 (m, 4 H), 1.86-0.99 (m, 18 H); ^{13}**C NMR** (100 MHz, $CDCl_3$) δ = 169.1, 161.7, 143.3, 143.24, 143.21, 128.9, 128.8, 127.4, 127.11, 127.07, 127.06, 126.8, 57.1, 47.8, 47.7, 30.9, 29.1, 28.9, 27.3, 26.99, 26.97, 26.4, 26.3, 25.6, 25.5, 25.22, 25.20; ^{19}**F NMR** (376 MHz, $CDCl_3$) δ = -100.1 (d, J = 285.0 Hz), -102.6 (d, J = 285.0 Hz); **HRMS** (ESI) calcd for $C_{32}H_{38}DF_2NNaO_3$ [M+Na$^+$]: 547.2853, found: 547.2848.

Synthesis of **6d:**

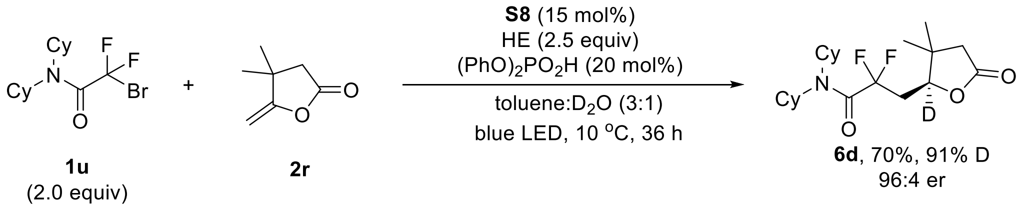

The reaction of HE (63.3 mg, 0.25 mmol), **1u** (67.5 mg, 0.2 mmol), **S8** (10.8 mg, 0.015 mmol), $(PhO)_2PO_2H$ (5.0 mg, 0.02 mmol), toluene (0.75 mL), **2r** (13.0 mg, 0.1 mmol) and D_2O (0.25 mL) afforded **6d** (27.0 mg, 70%, 91% D) (eluent: petroleum ether/ethyl acetate = 20/1 to 15/1 to 10/1) as a faint yellow oil: 96:4 er (HPLC conditions: Chiralcel AD-H column, hexane/*i*-PrOH = 90/10, 0.5 mL/min, λ = 214 nm, t_R (major) = 9.4 min, t_R (minor) = 10.4 min); $[\alpha]_D^{25}$ = -40.6 (c = 0.18, $CHCl_3$); ^{1}**H NMR** (400 MHz, $CDCl_3$) δ = 4.44 (dd, J_1 = 9.6 Hz, J_2 = 1.6 Hz, 0.09 H), 4.03 (tt, J_1 = 11.7 Hz, J_2 = 3.5 Hz, 1 H), 3.02 (tt, J_1 = 12.0 Hz, J_2 = 3.8 Hz, 1 H), 2.66-2.25 (m, 6 H), 1.99-0.99 (m, 24 H); ^{13}**C NMR** (100 MHz, $CDCl_3$) δ = 175.5, 161.7 (t, J = 27.0 Hz), 118.70 (t, J = 257.4 Hz), 81.4 (t, $J_{C\text{-}D}$ = 23.1 Hz), 57.2-57.0 (m), 44.1, 39.7, 39.6, 34.7-34.2 (m), 30.8, 28.9, 26.27, 26.25, 25.51, 25.47, 25.14, 25.09, 24.1, 21.0; ^{19}**F NMR** (376 MHz, $CDCl_3$) δ = -97.0 (d, J = 281.6 Hz), -100.7 (d, J = 281.6 Hz); **HRMS** (ESI) calcd for $C_{21}H_{32}DF_2NNaO_3$ [M+Na$^+$]: 409.2383, found: 409.2387.

Synthesis of **6e:**

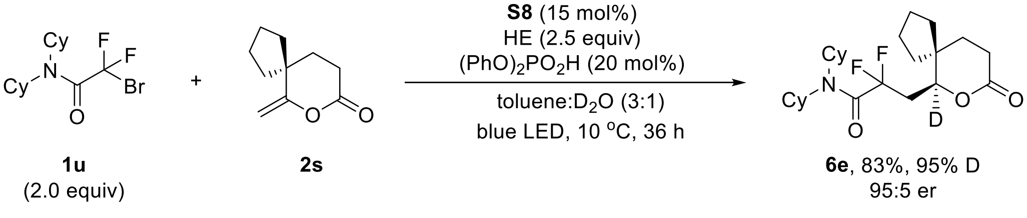

The reaction of HE (63.8 mg, 0.25 mmol), **1u** (67.8 mg, 0.2 mmol), **S8** (10.7 mg, 0.015 mmol), $(PhO)_2PO_2H$ (5.2 mg, 0.02 mmol), toluene (0.75 mL), **2s** (17.0 mg, 0.1 mmol) and D_2O (0.25 mL) afforded **6e** (35.5 mg, 83%, 95% D) (eluent: petroleum ether/ethyl acetate = 20/1 to 15/1 to 7/1) as a faint yellow oil: 95:5 er (HPLC conditions: Chiralcel OD-H column, hexane/*i*-PrOH = 98/2, 1.0 mL/min, λ = 214 nm, t_R (major) = 9.6 min, t_R (minor) = 10.6 min); $[\alpha]_D^{25}$ = -35.0 (c = 0.16, $CHCl_3$); **^{1}H NMR** (400 MHz, $CDCl_3$) δ = 4.60-4.53 (m, 0.05 H), 4.03 (tt, J_1 = 11.6 Hz, J_2 = 3.6 Hz, 1 H), 3.01 (tt, J_1 = 11.9 Hz, J_2 = 3.7 Hz, 1 H), 2.70-2.30 (m, 6 H), 1.94-1.01 (m, 28 H); **^{13}C NMR** (100 MHz, $CDCl_3$) δ = 170.7, 161.9 (t, $J_{C\text{-}F}$ = 27.0 Hz), 119.0 (t, $J_{C\text{-}F}$ = 257.7 Hz), 80.3 ($J_{C\text{-}D}$ = 21.6 Hz), 57.2-57.0 (m), 43.6, 37.1 (t, J = 22.7 Hz), 35.7, 32.5, 30.9, 30.8, 29.0, 28.9, 27.3, 26.33, 26.32, 25.6, 25.5, 25.3, 25.20, 25.16, 24.8; **^{19}F NMR** (376 MHz, $CDCl_3$) δ = -97.8 (d, J = 280.9 Hz), -100.2 (d, J = 280.9 Hz); **HRMS** (ESI) calcd for $C_{24}H_{36}DF_2NNaO_3$ [M+Na$^+$]: 449.2696, found: 449.2689.

Synthesis of **6f:**

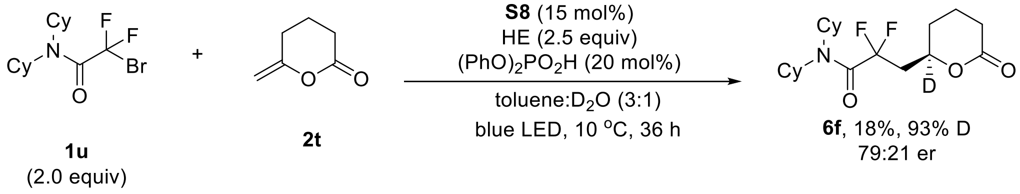

The reaction of HE (63.3 mg, 0.25 mmol), **1u** (67.6 mg, 0.2 mmol), **S8** (10.6 mg, 0.015 mmol), $(PhO)_2PO_2H$ (5.0 mg, 0.02 mmol), toluene (0.75 mL), **2t** (11.6 mg, 0.1 mmol) and D_2O (0.25 mL) afforded **6f** (6.7 mg, 18%, 93% D) (eluent: petroleum ether/ethyl acetate = 20/1 to 15/1 to 7/1) as a faint yellow oil. Unreacted **2t** accounts for the rest of the mass balance. **6f**: 79:21 er (HPLC conditions: Chiralcel OJ-H column, hexane/*i*-PrOH = 99.7/0.3, 0.5 mL/min, λ = 214 nm, t_R (minor) = 40.6 min, t_R (major) = 46.5 min); $[\alpha]_D^{25}$ = -19.3 (c = 0.22, $CHCl_3$); **^{1}H NMR** (400 MHz, $CDCl_3$) δ = 4.77-4.63 (m, 0.07 H), 4.07-3.93 (m, 1 H), 3.09-2.95 (m, 1 H), 2.68-2.30 (m, 6 H), 2.18-1.01 (m, 22 H); **^{13}C NMR** (100 MHz, $CDCl_3$) δ = 171.1, 161.8, 118.6 (t, J = 256.7 Hz), 57.3-57.0 (m), 41.1 (t, J = 23.2 Hz), 30.9, 29.1, 29.0, 28.2, 26.4, 25.60, 25.57, 25.2, 18.2; **^{19}F NMR** (376 MHz, $CDCl_3$) δ = -96.3 (d, J = 282.0 Hz), -99.1 (d, J = 282.0 Hz); **HRMS** (ESI) calcd for $C_{20}H_{31}DF_2NO_3$ [M+H$^+$]: 373.2408, found: 373.2400.

2.4. Synthetic Applications

Synthesis of **7:**

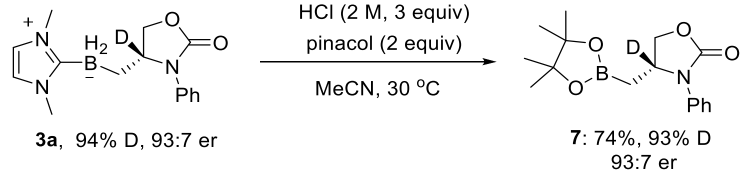

To a solution of **3a** (28.5 mg, 0.1 mmol) in CH_3CN (2 mL) was added HCl (2 M in water, 150 μL, 0.3 mmol) and pinacol (24.9 mg, 0.2 mmol). The reaction mixture was stirred at 30 °C for 1.5 h and then evaporated. The crude material was purified by flash column chromatography on silica gel (eluent: petroleum ether/ethyl acetate = 5/1) to give pinacol boronate ester **7** (22.5 mg, 74%, 93% D) as a colorless oil: 93:7 er (HPLC conditions: Chiralcel OD-H column, hexane/i-PrOH = 80/20, 0.5 mL/min, λ = 214 nm, t_R (major) = 11.8 min, t_R (minor) = 13.2 min); $[\alpha]_D^{25}$ = -45.7 (c = 0.30, $CHCl_3$); **^{1}H NMR** (400 MHz, $CDCl_3$) δ = 7.46-7.32 (m, 4 H), 7.24-7.10 (m, 1 H), 4.68-4.52 (m, 1.07 H), 4.06 (d, *J* = 8.8 Hz, 1 H), 1.41 (d, *J* = 16.4 Hz, 1 H), 1.34-1.15 (m, 12 H) 1.07 (d, *J* = 16.0 Hz, 1 H); **^{13}C NMR** (100 MHz, $CDCl_3$) δ = 155.9, 136.4, 129.1, 125.4, 122.7, 83.8, 75.0, 68.6, 24.9, 24.8, 24.6; **^{11}B NMR** (128 MHz, $CDCl_3$) δ = 32.9; **HRMS** (ESI) calcd for $C_{16}H_{21}BDNNaO_4$ $[M+Na^+]$: 327.1597, found: 327.1601.

Synthesis of **8:**

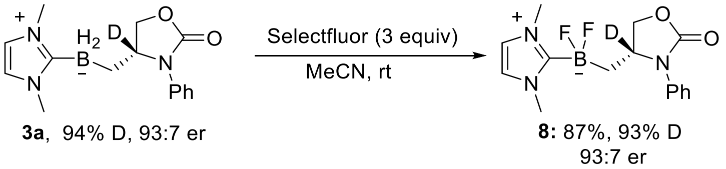

To a solution of **3a** (28.6 mg, 0.1 mmol) in CH_3CN (2 mL) was added selectfluor (107.5 mg, 0.3 mmol) at room temperature under nitrogen atmosphere. After addition, the reaction mixture was stirred at room temperature for 2 h and then evaporated. The crude material was purified by flash column chromatography on silica gel (eluent: petroleum ether/ethyl acetate = 2/3) to give **8** (27.7 mg, 87%, 93% D) as a colorless oil: 93:7 er (HPLC conditions: Chiralcel OD-H column, hexane/*i*-PrOH = 65/35, 0.5 mL/min, λ = 214 nm, t_R (major) = 17.2 min, t_R (minor) = 19.4 min); $[\alpha]_D^{25}$ = -83.5 (*c* = 0.10, $CHCl_3$); **^{1}H NMR** (400 MHz, $CDCl_3$) δ = 7.51-7.28 (m, 4 H), 7.19-7.03 (m, 1 H), 6.82 (s, 2 H), 4.74-4.49 (m, 1.07 H), 4.31 (d, *J* = 8.8 Hz, 1 H), 3.80 (s, 6 H), 1.03-0.71 (m, 1 H), 0.63-0.42 (m, 1 H); **^{13}C NMR** (100 MHz, $CDCl_3$) δ = 156.3, 137.4, 128.8, 124.2, 121.9,

121.6, 69.6, 69.5, 54.7 (t, $J_{C\text{-}D}$ = 22.3 Hz), 36.3 (t, J = 4.8 Hz), 29.7; **^{11}B NMR** (128 MHz, $CDCl_3$) δ = 5.26; **^{19}F NMR** (376 MHz, $CDCl_3$) δ = -155.1 (m), -157.2 (m); **HRMS** (ESI) calcd for $C_{15}H_{17}BDF_2N_3NaO_2$ [M+Na$^+$]: 345.1415, found: 345.1419.

Synthesis of **9:**

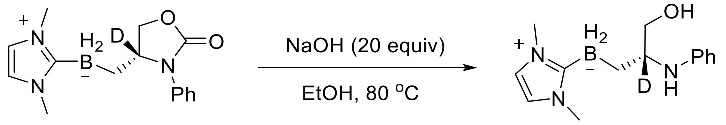

3a, 94% D, 93:7 er

9: 61%, 92% D
93:7 er

To a solution of **3a** (28.4 mg, 0.1 mmol) in EtOH (2 mL) was added NaOH (80.8 mg, 1 mmol), the reaction mixture was then heated to 80 °C for 10 h. After the reaction reached completion, EtOAc (5 mL) and H_2O (5 mL) were added. The organic layer was separated and the aqueous layer was extracted with EtOAc (5 mL x 3). The combined organic phase was dried over anhydrous Na_2SO_4, and then concentrated under reduced pressure. The residue was purified by chromatography on silica gel (eluent: petroleum ether/ethyl acetate = 20/1) to afford **9** (15.9 mg, 61%, 92% D) as a colorless oil: 93:7 er (HPLC conditions: Chiralcel OD-H column, hexane/*i*-PrOH = 90/10, 0.5 mL/min, λ = 214 nm, t_R (major) = 83.4 min, t_R (minor) = 90.7 min); $[\alpha]_D^{25}$ = -6.8 (*c* = 0.53, $CHCl_3$); **^{1}H NMR** (400 MHz, $CDCl_3$) δ = 7.18-7.04 (m, 2 H), 6.76 (s, 2 H), 6.68-6.57 (m, 3 H), 3.75 (d, J = 10.8 Hz, 1 H), 3.70-3.60 (m, 7 H), 3.31-2.33 (m, 2.08 H), 0.76-0.54 (m, 2 H); **^{13}C NMR** (100 MHz, $CDCl_3$) δ = 148.8, 129.0, 120.2, 116.8, 113.8, 66.7, 56.8 (t, $J_{C\text{-}D}$ = 19.9 Hz), 35.7; **^{11}B NMR** (128 MHz, $CDCl_3$) δ = -29.5 (t, J = 67.8 Hz). HRMS analysis indicated that the BH_2 moiety of the product was also partially deuterated (see the first entry of the spectrum below).

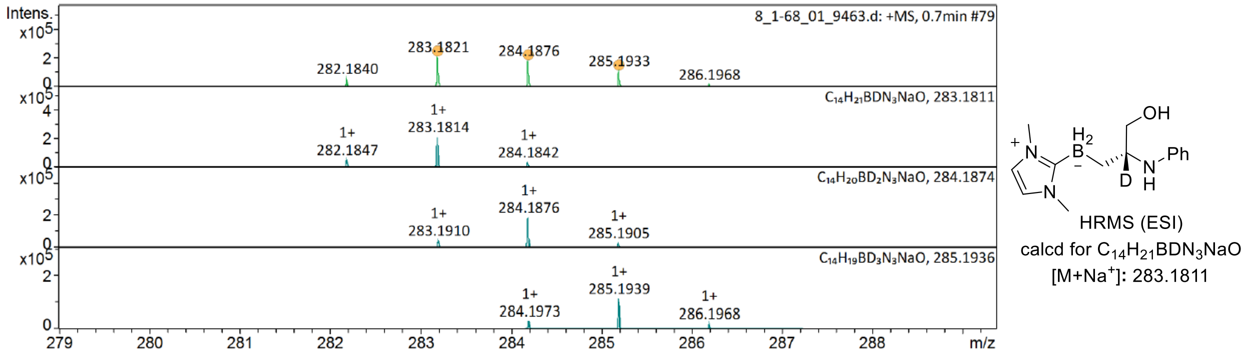

Synthesis of **10:**

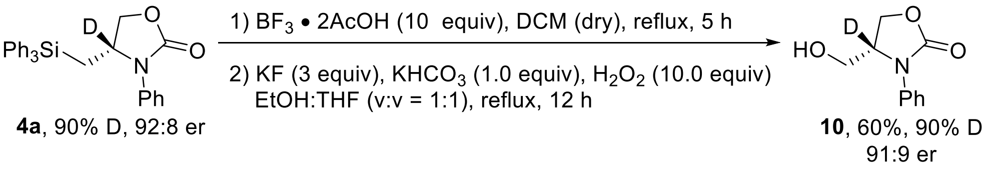

To a solution of **4a** (43.7 mg, 0.1 mmol) in dry CH_2Cl_2 (1 mL) was added BF_3•2AcOH (140 µL, 1 mmol), the mixture was then heated to reflux for 5 h. The reaction mixture was cooled to ambient temperature, and poured into a saturated aqueous solution of sodium bicarbonate. The aqueous layer was extracted with EtOAc. The combined organic layer was dried over anhydrous Na_2SO_4 and concentrated. To a solution of the residue in THF–EtOH (1.2 mL, v/v = 1/1) was added $KHCO_3$ (10.5 mg, 0.1 mmol), KF (17.9 mg, 0.3 mmol), and a 30% aqueous solution of H_2O_2 (120 µL, 1 mmol). The mixture was heated to reflux for 12 h. When the reaction is complete as monitored by TLC, the mixture was poured into an aqueous solution of sodium bicarbonate. The aqueous layer was extracted with EtOAc (10 mL × 3), and then the combined organic layer was washed with brine and dried over anhydrous Na_2SO_4. After filtration and evaporation, the residue was purified by chromatography on silica gel (eluent: petroleum ether/ethyl acetate = 1/1) to afford **10** (11.7 mg, 60%, 90% D) as a white solid: 91:9 er (HPLC conditions: Chiralcel OD-H column, hexane/*i*-PrOH = 80/20, 0.5 mL/min, λ = 214 nm, t_R (major) = 18.1 min, t_R (minor) = 20.6 min); $[\alpha]_D^{25}$ = -40.0 (*c* = 0.15, $CHCl_3$); **m.p.** 111-113 ℃; **^{1}H NMR** (400 MHz, CD_3OD) δ = 7.59-7.46 (m, 2 H), 7.45-7.34 (m, 2 H), 7.27-7.16 (m, 1 H), 4.66-4.49 (m, 1.10 H), 4.43 (d, *J* = 8.4 Hz, 1 H), 3.66 (d, *J* = 12.0 Hz, 1 H), 3.55 (d, *J* = 12.0 Hz, 1 H); **^{13}C NMR** (100 MHz, CD_3OD) δ = 158.7, 137.9, 130.1, 126.7, 124.0, 66.0, 60.3, 59.0 (t, $J_{C\text{-}D}$ = 22.1 Hz); **HRMS** (ESI) calcd for $C_{10}H_{10}DNNaO_3$ [$M+Na^+$]: 217.0694, found: 217.0696.

Synthesis of **11:**

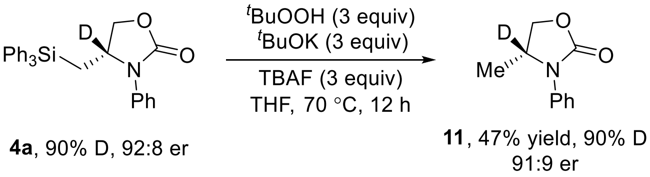

Under nitrogen atmosphere, ^{t}BuOK (68.2 mg, 0.6 mmol), THF (2.0 mL), and ^{t}BuOOH (70% in water, 79.3 mg, 0.6 mmol) were added to a 10 mL flame-dried Schlenk flask at 0 °C. The mixture was stirred for 10 minutes, then a solution of **4a** (87.4 mg, 0.2 mmol) in 1 mL of THF

and 0.6 mL of TBAF (1.0 M in THF, 0.6 mmol) were added sequentially, the resulted mixture was stirred at 70 °C for 12 h. The reaction mixture was allowed to cool to room temperature before ether (5 mL) and H_2O (5 mL) were added. The organic layer was separated and the aqueous layer was extracted with ether (5 mL x 3). The combined organic phase was dried over anhydrous Na_2SO_4 and then concentrated under reduced pressure. The residue was purified by chromatography on silica gel (eluent: petroleum ether/ethyl acetate = 5/1) to afford **11** (16.8 mg, 47%, 90% D) as a colorless oil : 91:9 er (HPLC conditions: Chiralcel AD-H column, hexane/*i*-PrOH = 90/10, 1 mL/min, λ = 214 nm, t_R (major) = 10.9 min, t_R (minor) = 12.1 min); $[\alpha]_D^{25}$ = -53.8 (*c* = 0.11, $CHCl_3$); **^{1}H NMR** (400 MHz, $CDCl_3$) δ = 7.46-7.34 (m, 4 H), 7.23-7.15 (m, 1 H), 4.63-4.47 (m, 1.10 H), 4.02 (d, *J* = 8.4 Hz, 1 H), 1.33 (s, 3 H); **^{13}C NMR** (100 MHz, $CDCl_3$) δ = 155.7, 136.5, 129.1, 125.2, 122.0, 121.9, 68.5, 51.9 (t, J_{C-D} = 22.0 Hz), 18.3; **HRMS** (ESI) calcd for $C_{10}H_{10}DNNaO_2$ [M+Na$^+$]: 201.0745, found: 201.0744.

Synthesis of **12:**

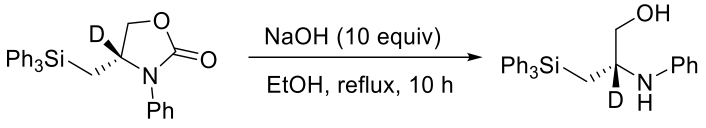

4a, 90% D, 92:8 er

12, 67%, 90% D
92:8 er

To a solution of **4a** (43.7 mg, 0.1 mmol) in EtOH (1 mL) was added NaOH (41.2 mg, 1 mmol), the reaction mixture was then heated to reflux for 10 h. The reaction mixture was allowed to cool to room temperature, EtOAc (5 mL) and H_2O (5 mL) were added. The organic layer was separated and the aqueous layer was extracted with EtOAc (5 mL x 3). The combined organic phase was dried over anhydrous Na_2SO_4, and then concentrated under reduced pressure. The residue was purified by chromatography on silica gel (eluent: petroleum ether/ethyl acetate = 5/1) to afford **12** (27.5 mg, 67%, 90% D) as a colorless oil: 92:8 er (HPLC conditions: Chiralcel OJ-H column, hexane/*i*-PrOH = 85/15, 1 mL/min, λ = 214 nm, t_R (minor) = 9.3 min, t_R (major) = 14.3 min); $[\alpha]_D^{25}$ = +9.3 (*c* = 0.23, $CHCl_3$); **^{1}H NMR** (400 MHz, $CDCl_3$) δ = 7.55-7.44 (m, 6 H),7.43-7.24 (m, 9 H), 7.06 (t, *J* = 8.0 Hz, 2 H), 6.69 (t, *J* = 7.4 Hz, 1 H), 6.29 (d, *J* = 7.6 Hz, 2 H), 3.73-3.66 (m, 0.1 H), 3.61 (d, *J* = 11.2 Hz, 1 H)), 3.43 (d, *J* = 10.8 Hz, 1 H), 3.30-2.42 (m, 2 H), 1.87-1.16 (m, 2 H); **^{13}C NMR** (100 MHz, $CDCl_3$) δ = 145.9, 135.6, 134.3, 129.6, 129.2, 128.0, 118.3, 114.1, 65.8, 16.7; **HRMS** (ESI) calcd for $C_{27}H_{27}DNOSi$ [M+H$^+$]: 411.1997, found: 411.2002.

2.5. Radical Trap Experiment

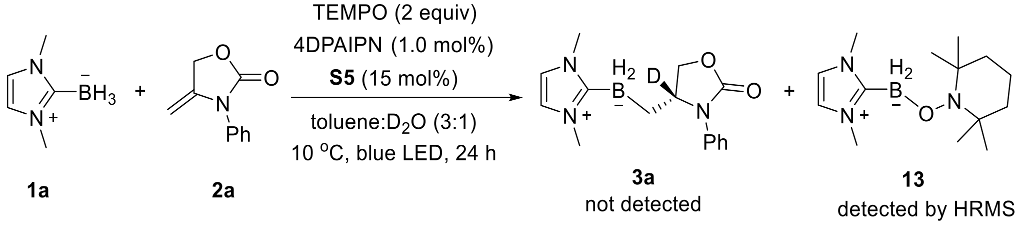

To an oven-dried 16 x 60 mm vial containing a dry Teflon stir bar were charged with photocatalyst 4DPAIPN (1.0 mg, 0.001 mmol), thiol catalyst **S5** (8.5 mg, 0.015 mmol) and NHC−BH_3 **1a** (21.8 mg, 0.2 mmol). After sequential addition of dry toluene (0.75 mL), D_2O (0.25 mL), and olefin **2a** (17.5 mg, 0.1 mmol), the reaction mixture was flushed with nitrogen gas for two minutes and then the vial was sealed with a cap and parafilm. The vial was placed in a cooling station and a 30 W blue LED was then placed on the top of the cooling station, which is connected to a chiller to maintain the temperature of the cooling water at 10 ℃ (**Supplementary Figure 5**). After stirring with irradiation for 24 hours, a small portion of the reaction mixture was diluted with MeCN and filtered. The filtrate was subjected to high resolution mass spectra (HRMS) analysis. Product **3a** was not detected, instead **13** was observed: HRMS (ESI) calculated for **13** $C_{14}H_{29}BN_3O$ $[M+H^+]$: 266.2398, found: 266.2402 (**Supplementary Figure 6**).

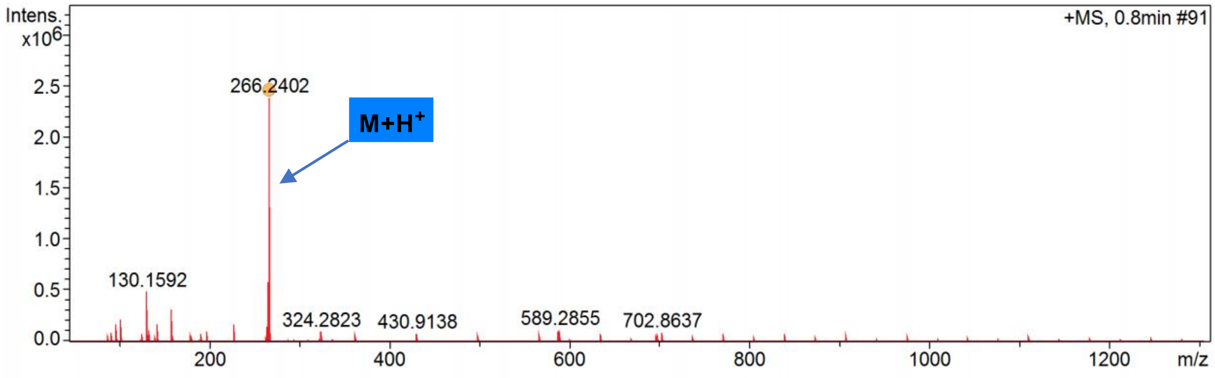

Meas. m/z	#	Ion Formula	m/z	Sum Formula	err [ppm]	mSigma	# mSigma	Score	rdb	e¯ Conf	N-Rule
266.2402	1	C14H29BN3O	266.2398	C14H28BN3O	-0.5	4.2	1	100.00	3.0	even	ok

Supplementary Figure 6. The HRMS spectrum of the crude reaction mixture.

2.6. UV-Vis Absorption Spectra of 1a, 2a, and S5

UV-Vis absorption spectra of **1a**, **2a**, **S5** and their combinations were measured (in toluene, **Supplementary Figure 7-10**). None of the individual reaction components can absorb in the visible region and none of combinations showed obvious bathochromic shift, excluding the possibility of forming EDA complex under the reaction conditions.

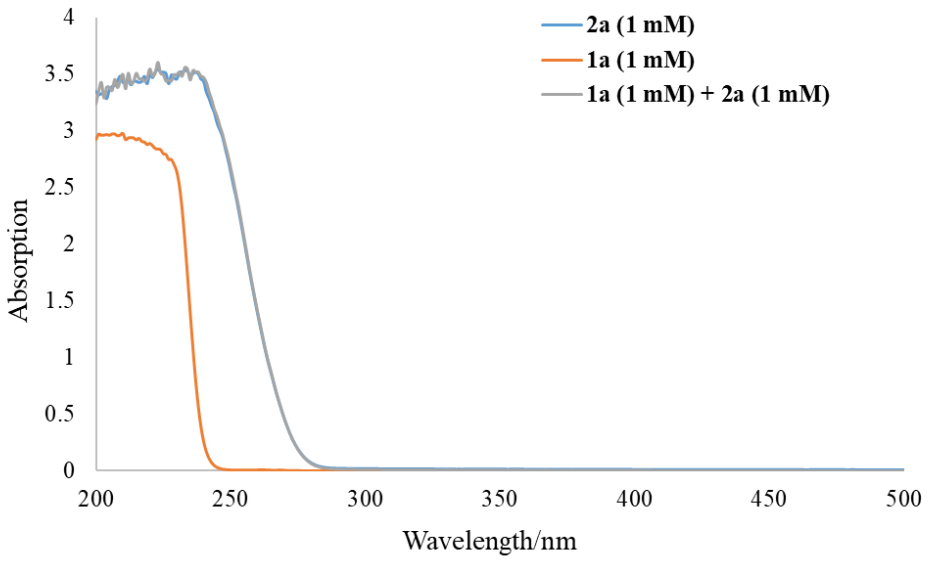

Supplementary Figure 7. UV-Vis spectra of **1a**, **2a,** and their mixture.

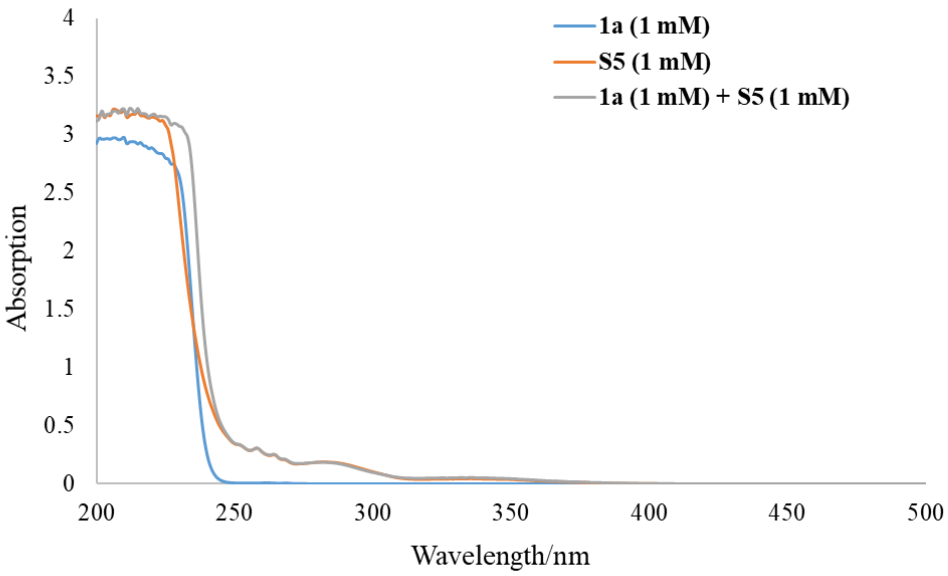

Supplementary Figure 8. UV-Vis spectra of **1a**, **S5,** and their mixture.

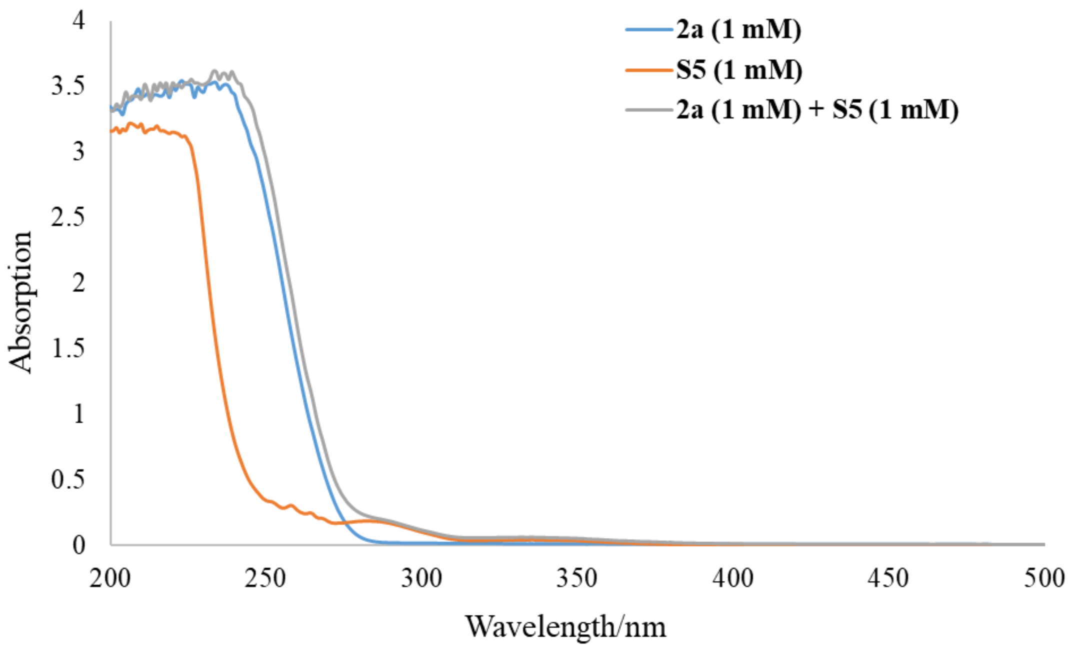

Supplementary Figure 9. UV-Vis spectra of **2a**, **S5,** and their mixture.

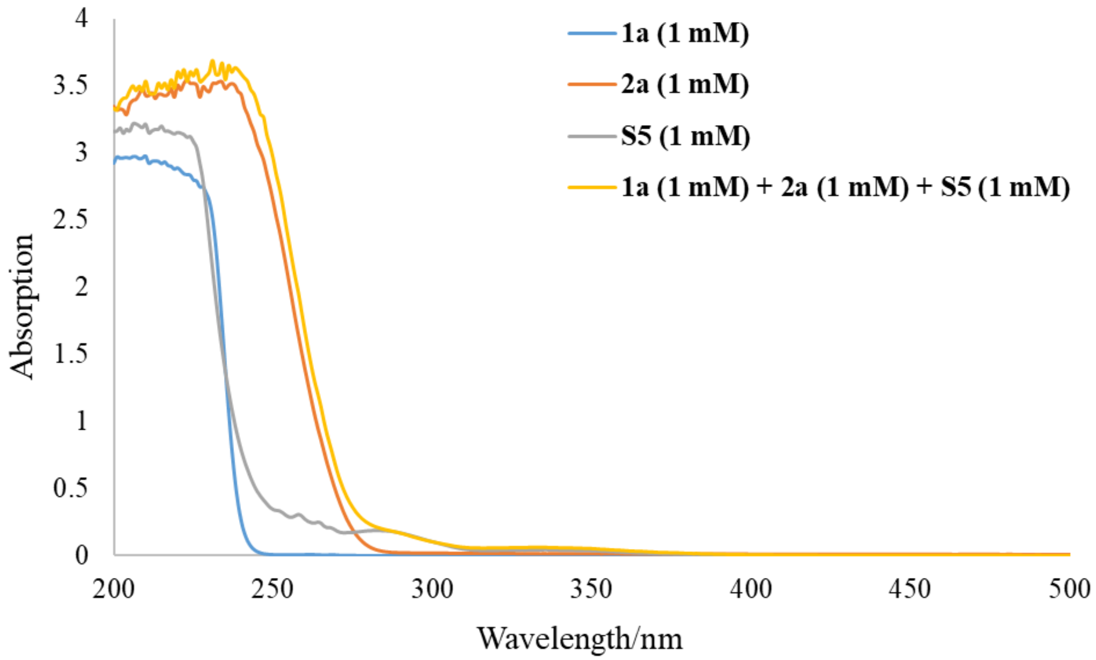

Supplementary Figure 10. UV-Vis spectra of **1a**, **2a**, **S5,** and their mixture.

2.7. Cyclic Voltammetry Measurements

Cyclic voltammetry (CV) experiments of **2a**, **S5**, and 4DPAIPN were recorded on a CHI 600E electrochemical workstation. Electrolyte solution was prepared by dissolving the substrates (0.05 mmol, 5 mM) and tetrabutylammonium hexafluorophosphate (387.4 mg, 1 mmol, 100 mM) in MeCN (10 mL) and bubbling with nitrogen for two minutes. Measurements were performed in a 3-compartment electrochemical cell, in which glassy carbon electrode (GCE) was used as a working electrode, silver-silver chloride (Ag/AgCl) in saturated KCl as the reference electrode, and Pt wire as the counter electrode. The scan rate was set at 100 mV/s.

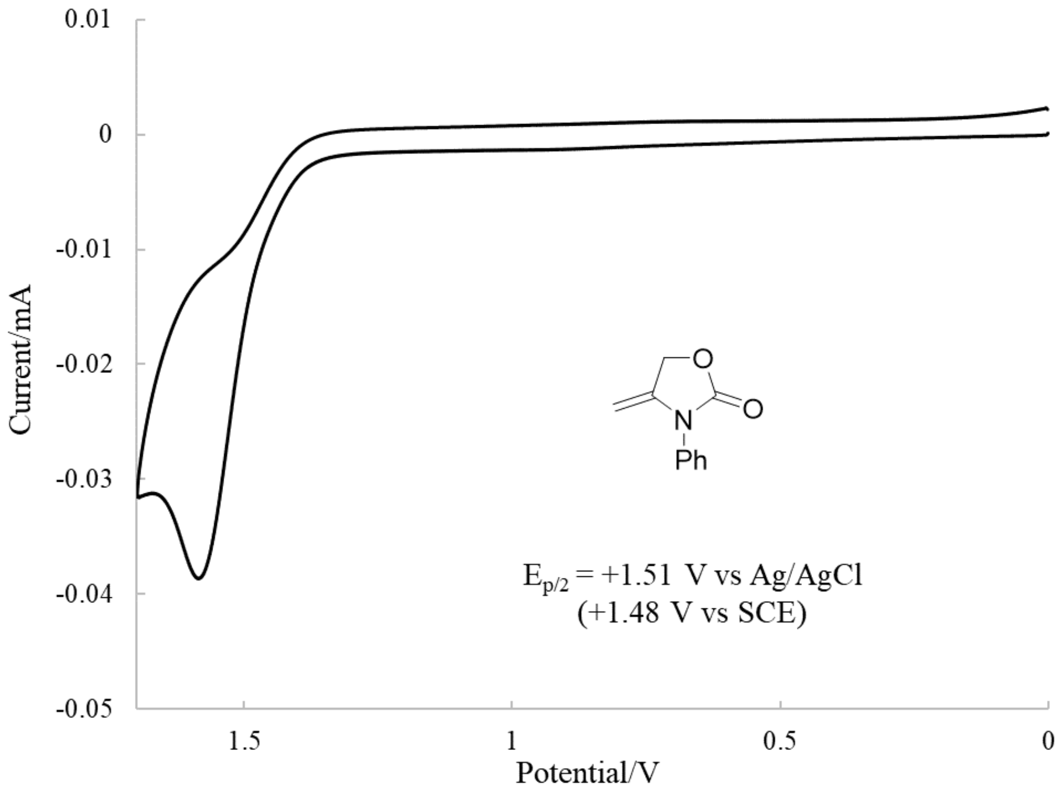

Supplementary Figure 11. CV curve of **2a**.

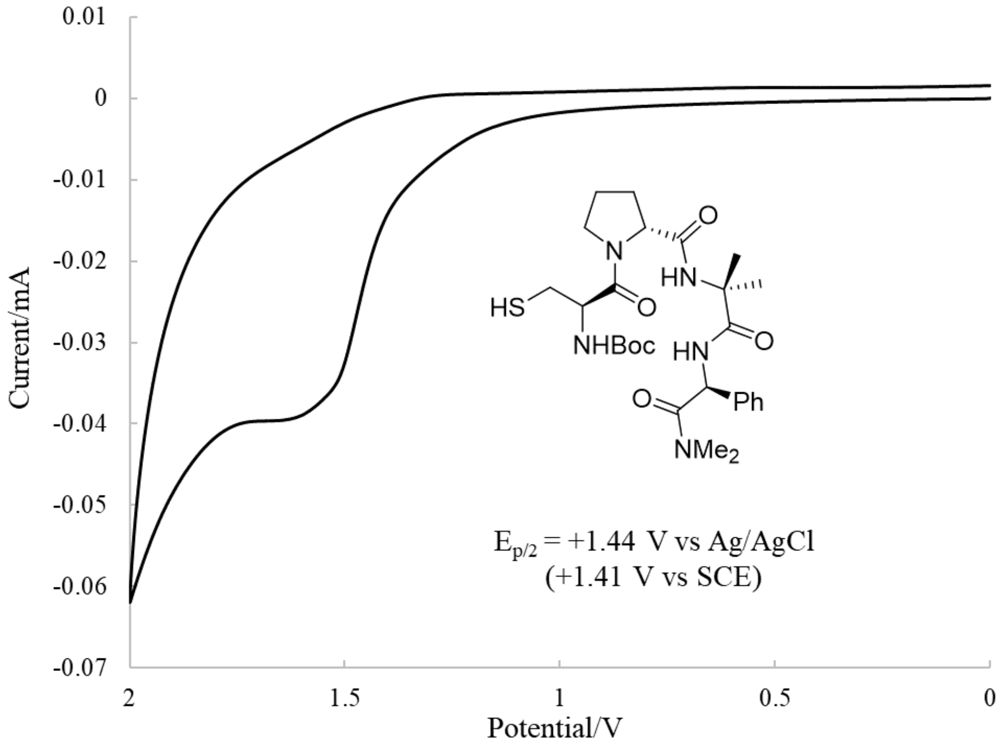

Supplementary Figure 12. CV curve of **S5**.

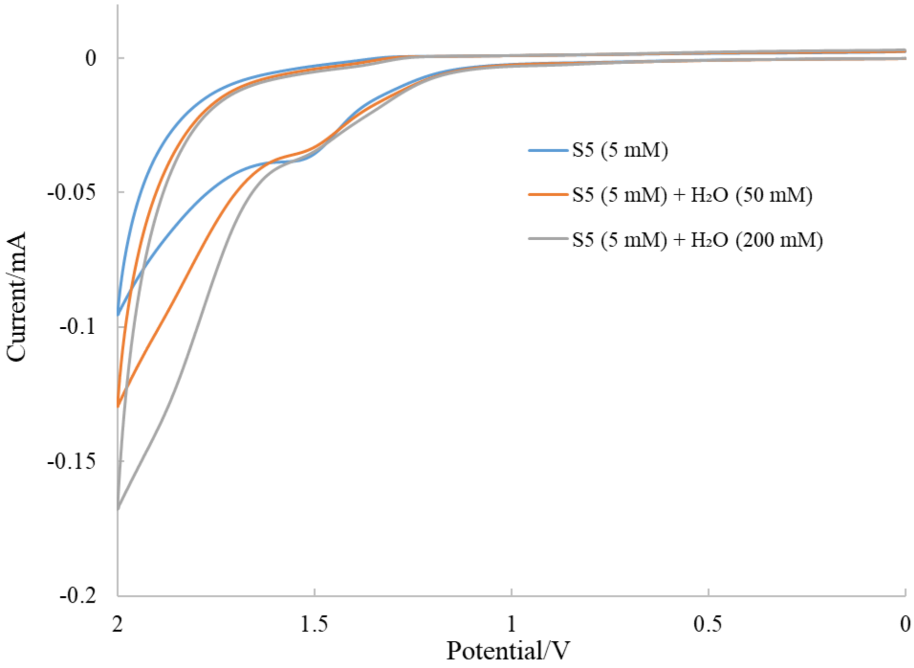

Supplementary Figure 13. CV curve of **S5** in the presence of H_2O.

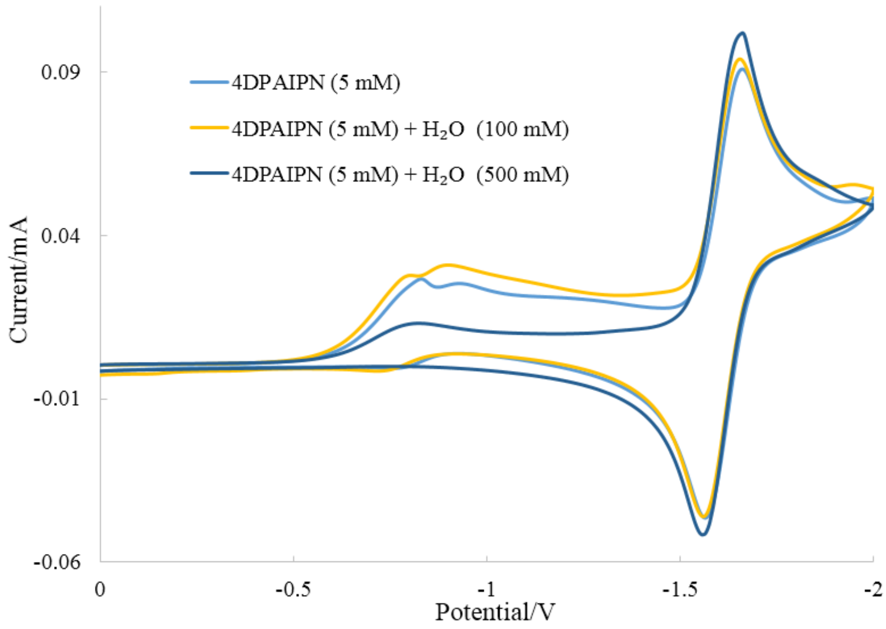

Supplementary Figure 14. CV curve of 4DPAIPN in the presence of H_2O.

2.8. Stern-Volmer Experiments

Stern-Volmer luminescence quenching experiments were carried out with freshly prepared solutions of 4DPAIPN (10^{-5} M) at room temperature. For **1a** and **2a**, the solutions were prepared in toluene and was irradiated at 350 nm and the luminescence were measured at 523 nm. For **S5**, the solution was prepared in DMF due to its poor solubility in toluene and was irradiated at 350 nm and the luminescence were measured at 530 nm. For each sample, the luminescence was acquired three times and averaged (**Supplementary Tables 3-6**), the averages of the results were used for the graphical representation (**Supplementary Figures 15-18**).

Supplementary Table 3. Fluorescence quenching data with solutions of 4DPAIPN and **1a**.

Species	Concentration (mM)
4DPAIPN	0.01
**1a**	varied

**[Sub] (mM)**	**Scan 1**	**Scan 2**	**Scan 3**	**Average**	**I_0/I**
0	1352	1351	1351	1351	1.000
5	1341	1342	1342	1342	1.007
10	1353	1353	1355	1354	0.998
15	1370	1371	1370	1370	0.986
20	1394	1393	1394	1394	0.969
25	1368	1368	1367	1368	0.988

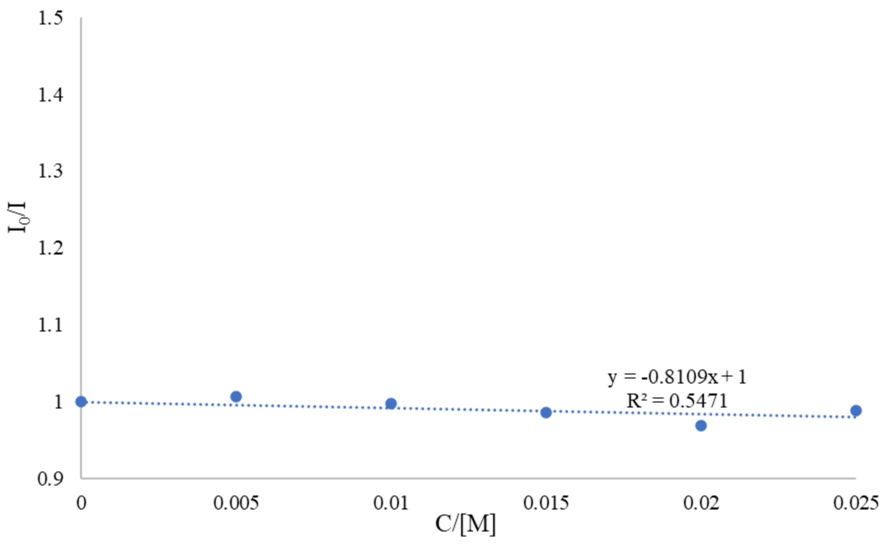

Supplementary Figure 15. Stern-Volmer plot of 4DPAIPN quenching with varying concentration of **1a**.

Supplementary Table 4. Fluorescence quenching data with solutions of 4DPAIPN and **2a**.

Species	Concentration (mM)
4DPAIPN	0.01
**2a**	varied

[Sub] (mM)	Scan 1	Scan 2	Scan 3	Average	I_0/I
0	1266	1266	1267	1266	1.000
5	1259	1258	1256	1258	1.006
10	1261	1251	1237	1250	1.013
15	1216	1212	1207	1212	1.045
20	1221	1220	1219	1220	1.038
25	1195	1196	1196	1196	1.059

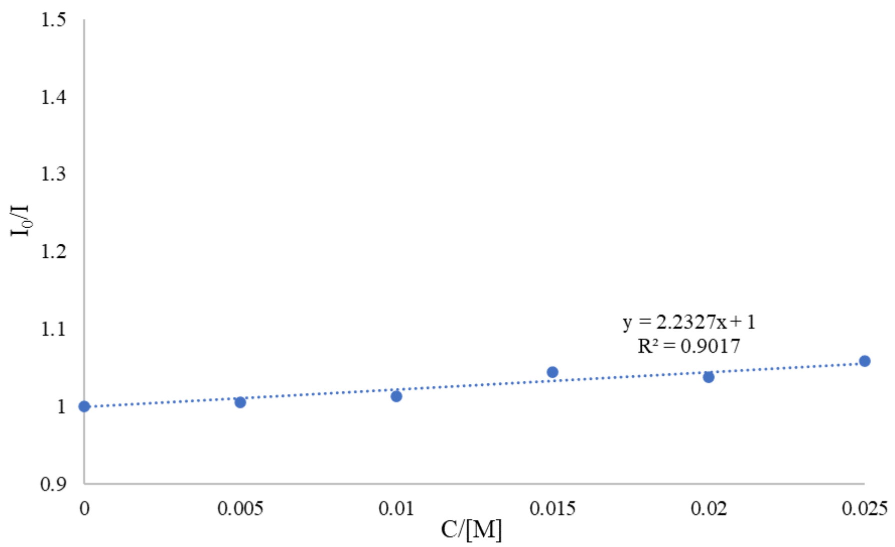

Supplementary Figure 16. Stern-Volmer plot of 4DPAIPN quenching with varying concentration of **2a**.

Supplementary Table 5. Fluorescence quenching data with solutions of 4DPAIPN and **S5**.

Species	Concentration (mM)
4DPAIPN	0.01
**S5**	varied

[Sub] (mM)	Scan 1	Scan 2	Scan 3	Average	I_0/I
0	1020	1021	1021	1021	1.000
5	946	943	948	946	1.079
10	874	874	874	874	1.168
15	818	819	818	818	1.248
20	764	766	767	766	1.333
25	723	723	723	723	1.412

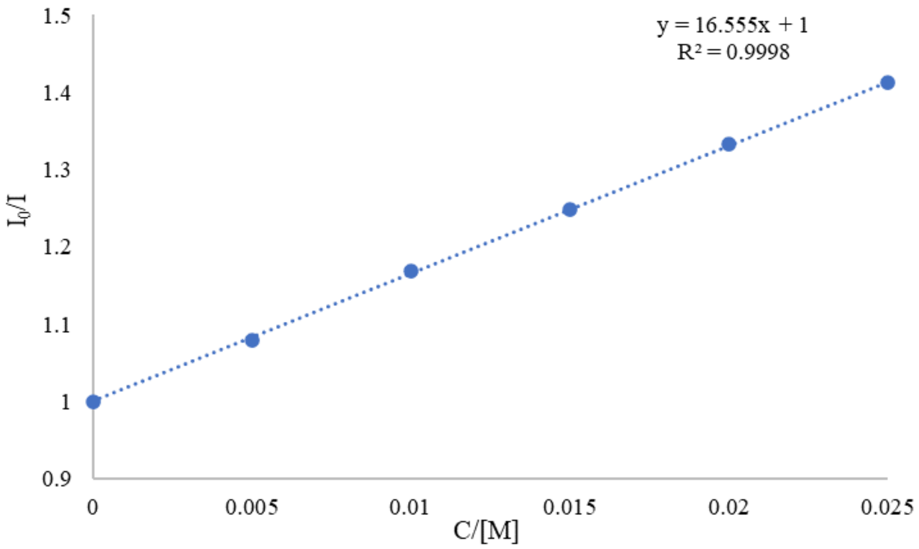

Supplementary Figure 17. Stern-Volmer plot of 4DPAIPN quenching with varying concentration of **S5**.

Supplementary Table 6. Fluorescence quenching data with solutions of 4DPAIPN, and **S5** in the presence of D_2O (5.0 M).

Species	Concentration (mM)
4DPAIPN	0.01
D_2O	5000
**S5**	varied

**[Sub] (mM)**	**Scan 1**	**Scan 2**	**Scan 3**	**Average**	**I_0/I**
0	1153	1156	1157	1155	1.000
1	1035	1033	1035	1034	1.117
2	969	969	969	969	1.192
3	913	913	914	913	1.265
4	868	868	868	868	1.331
5	843	843	843	843	1.370

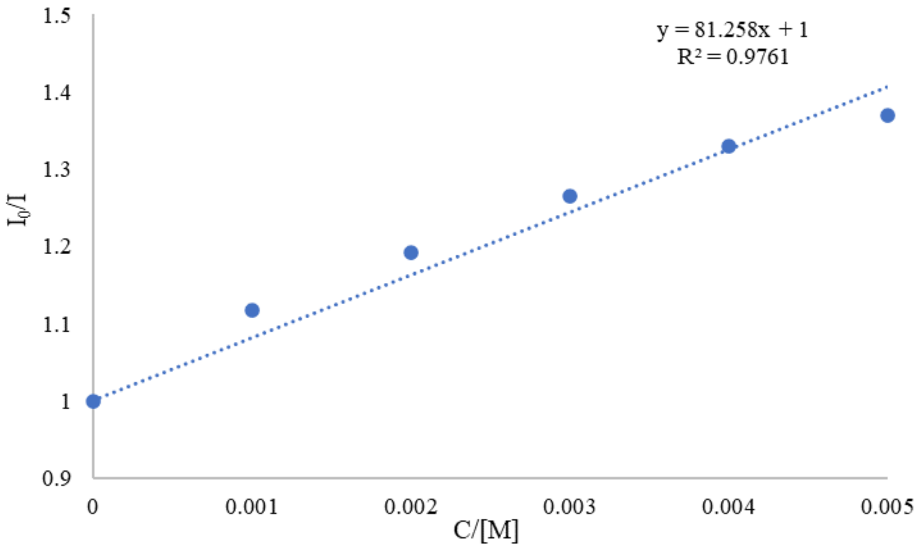

Supplementary Figure 18. Stern-Volmer plot of 4DPAIPN quenching with varying concentration of **S5** in the presence of D_2O (5.0 M).

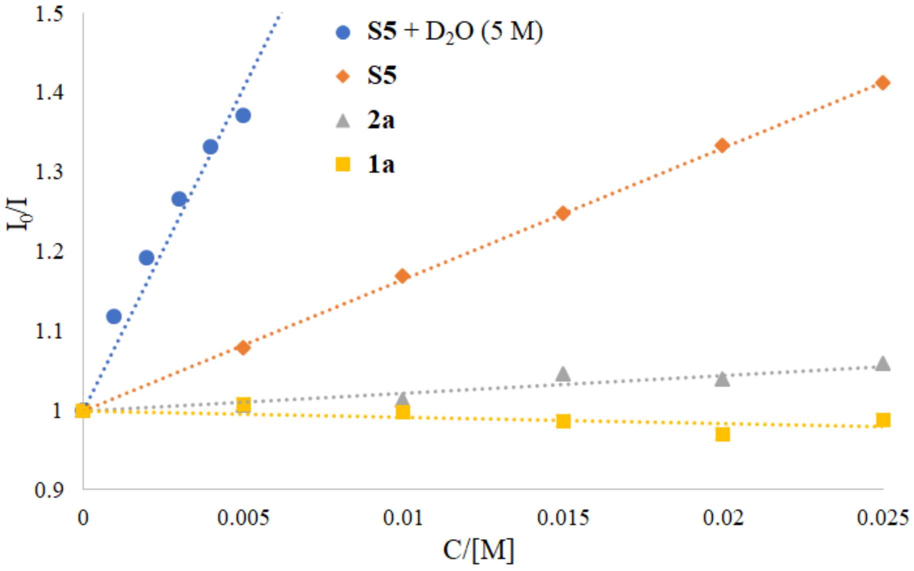

Supplementary Figure 19. Summary of Stern-Volmer plots.

Supplementary Table 7. The influence of D_2O on reaction rate.

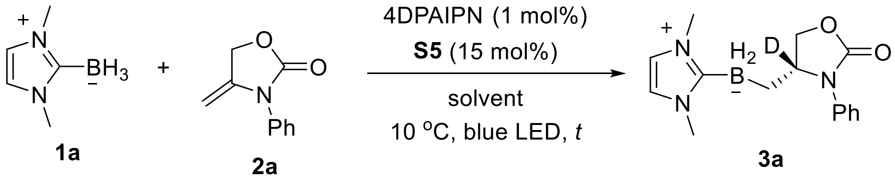

NMR yield of **3a** / *t*	toluene:D_2O (3:1)	toluene
2 h	16%	n.d.
4 h	26%	10%
8 h	n.d.	22%

n.d. : Not Determined

We briefly investigated the initial product formation rates for reactions with or without D_2O and found that the reaction with D_2O was much faster than the reaction without D_2O as shown above.

2.9. Quantum Yield Measurements

2.9.1. Emission spectrum of light source

Emission spectrum of the blue LED (30 W) was recorded on a Steady-State & Time-Resolved Fluorescence Spectrofluorometer (**Supplementary Figure 20**). According to the spectrum, emission range of light source is 400–500 nm, and the maximum emission wavelength is 441 nm.

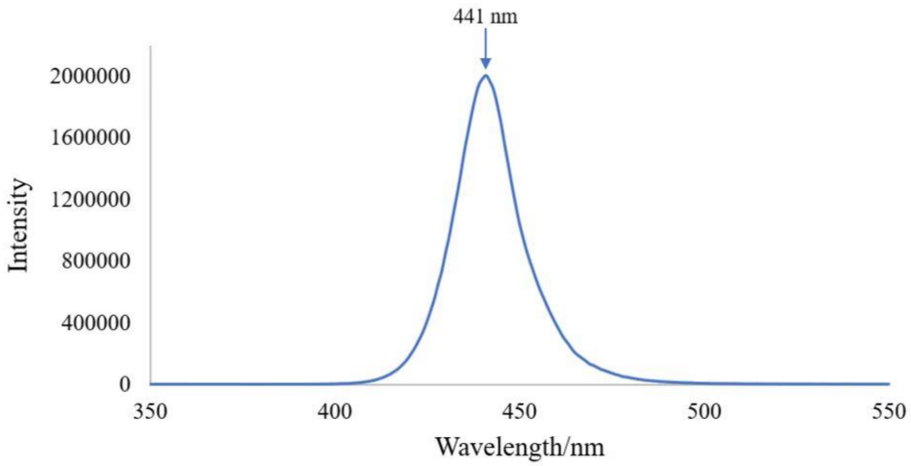

Supplementary Figure 20. Emission spectrum of the blue LED.

Quantum yield measurements were determined using standard ferrioxalate chemical actinometry as described by Yoon,[18] Ritter,[19] Alemán,[20] and Glorius.[21] In this part, we used a 30 W blue LED (λ = 441 nm) as light source to determine the quantum yield.

2.9.2. Determination of the photon flux at λ = 441 nm:

The photon flux of the 30 W blue LED was determined by monitoring the photoreduction of Fe(III) in potassium ferrioxalate to Fe(II), upon complexation with 1,10-phenanthroline:

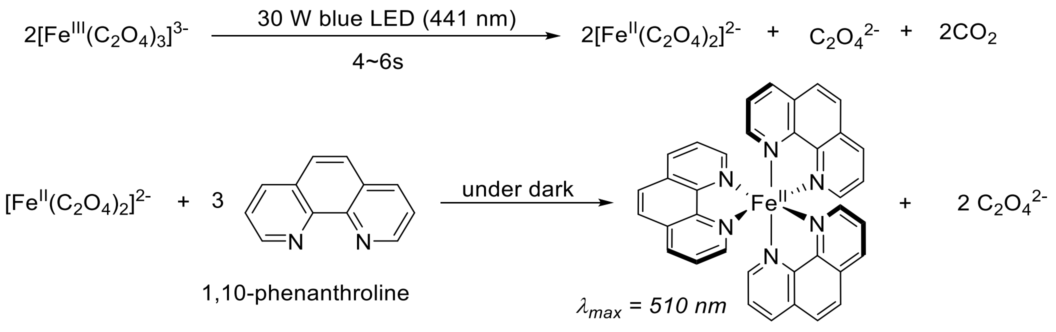

The following solutions were prepared:

a. Actinometer solution: 589.5 mg (1.2 mmol) of potassium ferrioxalate trihydrate and 278 μL of H_2SO_4 96% were added to a 100 mL volumetric flask and filled to the mark with Nanopure water.

b. Phenanthroline-buffer solution: 100 mg (0.55 mmol) of 1, 1,10-phenanthroline, 9.88 g (120.4 mmol) of sodium acetate and 2.0 mL of H_2SO_4 96% were added to a 100 mL volumetric flask and filled to the mark with Nanopure water.

1 mL of the actinometer solution was added to a vial (16 × 60 mm) and was irradiated by the 30 W blue LED for 4 seconds. After the irradiation, the mixture was quantitatively transferred to a 5 mL volumetric flask containing 1.0 mL of the phenanthroline-buffer solution. Then, the flask was filled to the mark with Nanopure water, wrapped up with aluminum foil, and was left in the dark for 1 h to ensure the quantitative formation of $Fe^{II}(phen)_3^{2+}$ complex. This procedure was repeated one more time by changing the irradiation time to 6 seconds. Additionally, the experiment of a control sample was carried out under dark, following the same sample treatment.

The absorbance of each solution at λ = 510 nm was measured using a Perkin-Elmer Lambda 35 UV-Vis spectrophotometer, establishing the blank with Nanopure water. For each sample, the absorbance was acquired three times and averaged. According to Lambert-Beer law, the moles of Fe(II) in each sample are related to the absorbance:

$$n\,[Fe^{2+}] = \frac{V \cdot \Delta A}{l \cdot \varepsilon}$$

Where:

- *ΔA* is the absorbance difference between irradiated sample and non-irradiated sample.
- *V* is the volume (in L) of the measurement sample (5 mL).
- *ε* is the extinction coefficient of the complex $Fe^{II}(phen)_3^{2+}$ at λ = 510 nm (11100 L mol^{-1} cm^{-1})
- *l* is the optical path of the sample in the spectrophotometer (1 cm).

The photon flux can be calculated using the following equation:

$$photon\ flux = \frac{n\,[Fe^{2+}]}{\Phi \cdot t \cdot f}$$

Where:

- Φ is the quantum yield for the photoreduction of ferrioxalate at λ = 441 nm, which is 1.11.[22,23]
- t is the reaction time (4 s or 6 s).
- f is the fraction of light absorbed, and is calculated as $f = 1 - 10^{-A_{441\,nm}}$

  where $A_{441\ nm}$ is the absorbance of the actinometer solution at λ = 441 nm, which is 0.3877 according to the UV-Vis spectra of actinometer solution (**Supplementary Figure 21**).

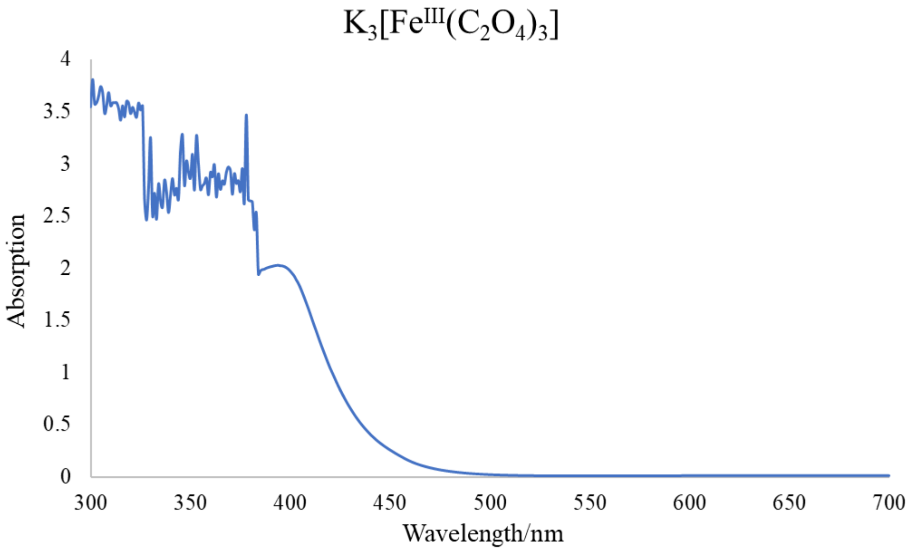

Supplementary Figure 21. UV-Vis spectra of actinometer solution (12 mM in aqueous solution).

The photon flux of the 30 W blue LED (λ = 441 nm) was thus determined to be 2.65*10^{-7} einsten s^{-1} (**Supplementary Table 8**).

Supplementary Table 8. UV-Vis absorption data and calculated results of photo flux.

scan time	UV-Vis absorption at 510 nm		
	A_{4s}	A_{6s}	A_{dark}
1	1.480	2.195	0.016
2	1.644	2.452	0.019
Average	1.562	2.324	0.017
ΔA (=A-A_{dark})	1.545	2.306	-
n [Fe^{2+}] (mol)	6.96*10^{-7}	1.04*10^{-6}	
Photon flux (einstein/s)	2.654*10^{-7}	2.642*10^{-7}	
	(average) 2.648*10^{-7}		

2.9.3. Determination of quantum yield at λ = 441 nm:

Once we have determined the photon flux of the 30 W blue LED (λ = 441 nm), the same equation was employed for the determination of the quantum yield of our photoreaction system. For that, the moles of product for a given time was determined.

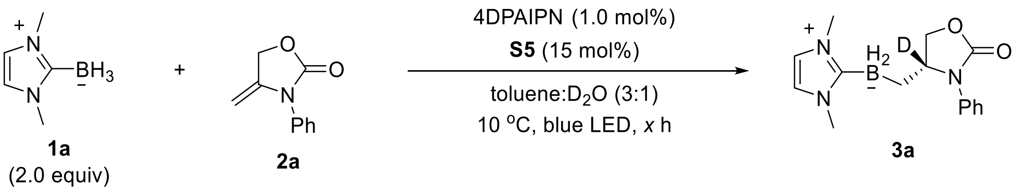

Following the **Typical Procedure I**, the reaction of **1a** and **2a** was carried out under the standard conditions and was stopped after 2 h of irradiation. The solvent of reaction mixture was

evaporated in vacuo. The amount of product **3a** was determined to be 1.6*10^{-5} mol by ^{1}H NMR analysis of the crude reaction mixture using CH_2Br_2 as the internal standard. Another reaction with an irradiation time of 4 h was also carried out and the amount of **3a** was determined to be 2.6*10^{-5} mol.

$$\Phi' = \frac{\text{n}\,[prod.]}{\text{photon flux} \cdot t' \cdot f'}$$

Where:

- n [prod.] is the amount of **3a** (in mol) that has been formed during the irradiation time.
- *t'* (s) is the irradiation time (in seconds).
- *f'* is the fraction of light absorbed, and is calculated as $f' = 1 - 10^{-A_{441\,nm}}$

  where $A_{441\ \mathrm{nm}}$ is the absorbance of PC solution at 441 nm, and was determined to be 2.5939 according to the UV-Vis spectra of 1 mM solution of 4DPAIPN in toluene (**Supplementary Figure 22**).

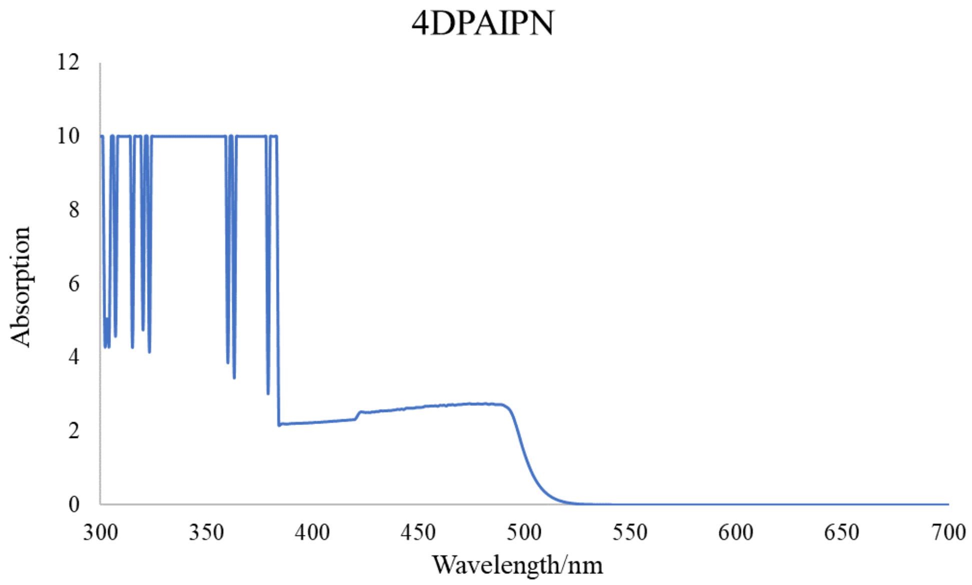

Supplementary Figure 22. UV-Vis spectra of 4DPAIPN solution (1 mM in toluene)

The quantum yield of the reaction was thus determined to be 0.0076 (average of two runs, **Supplementary Table 9**).

Supplementary Table 9. Quantum yield in the presence of D_2O.

entry	reaction time/s	NMR yield/%	n[prod.]/mol	quantum yield (Φ’)
1	7200	16	$1.6*10^{-5}$	0.0084
2	14400	26	$2.6*10^{-5}$	0.0068

$$\Phi' = (0.0084+0.0068)/2 = 0.76\%$$

We also measured the quantum yield of the reaction without D_2O, which is homogenous but also gave a very low value (Φ’ = 0.28%, see the **Supplementary Table 10** below), indicating that the low quantum yield is largely related to the slow reaction rate.

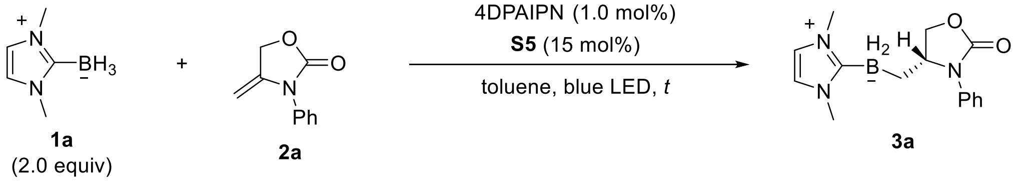

Supplementary Table 10. Quantum yield in the absence of D_2O.

entry	reaction time/s	NMR yield/%	n[prod.]/mol	quantum yield (Φ’)
1	14400	10	$1.0*10^{-5}$	0.0026
2	28800	22	$2.2*10^{-5}$	0.0029

$$\Phi' = (0.0026+0.0029)/2 = 0.28\%$$

2.10. X-Ray Crystallographic Data of Compounds 3c, 4a, and 5a

2.10.1. X-Ray Crystallographic Data of Compound 3c

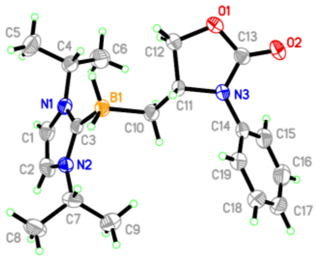

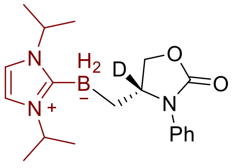

3c

Supplementary Figure 23. X-Ray Structure of Compound **3c** (CCDC 2143290). Crystals suitable for X-ray structure analysis were obtained via recrystallization from tetrahydrofuran and pentane.

Supplementary Table 11. Crystal data and structure refinement for **3c**.

Identification code	**3c**
Empirical formula	$C_{19}H_{28}BN_3O_2$
Formula weight	341.25
Temperature	173(2) K
Wavelength	1.54178 A
Crystal system, space group	Monoclinic, P2(1)
Unit cell dimensions	a = 5.8190(2) A alpha = 90 deg.
	b = 15.2860(4) A beta = 94.197(2) deg.
c = 21.4445(5) A	gamma = 90 deg.
Volume	1902.36(9) A^3
Z, Calculated density	4, 1.191 Mg/m^3
Absorption coefficient	0.609 mm^-1
F(000)	736
Crystal size	0.180 x 0.160 x 0.140 mm
Theta range for data collection	3.554 to 68.137 deg.
Limiting indices	-6<=h<=6, -18<=k<=18, -25<=l<=25
Reflections collected / unique	26076 / 6870 [R(int) = 0.0539]
Completeness to theta = 67.679	99.9 %
Absorption correction	Semi-empirical from equivalents
Refinement method	Full-matrix least-squares on F^2

Data / restraints / parameters	6870 / 1 / 471
Goodness-of-fit on F^2	1.049
Final R indices [I>2sigma(I)]	R1 = 0.0423, wR2 = 0.1063
R indices (all data)	R1 = 0.0484, wR2 = 0.1098
Absolute structure parameter	-0.12(11)
Extinction coefficient	n/a
Largest diff. peak and hole	0.193 and -0.188 e.A^-3

2.10.2 X-Ray Crystallographic Data of Compound 4a

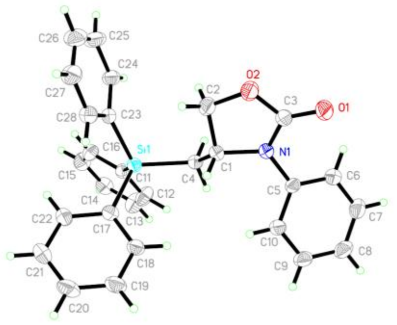

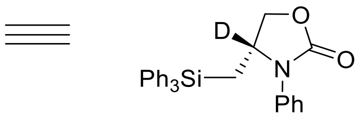

4a

Supplementary Figure 24. X-Ray Structure of Compound **4a** (CCDC 2106905). Crystals suitable for X-ray structure analysis were obtained via recrystallization from tetrahydrofuran and pentane.

Supplementary Table 12. Crystal data and structure refinement for **4a**.

Identification code	**4a**	
Empirical formula	$C_{28}H_{25}NO_2Si$	
Formula weight	435.58	
Temperature	299(2) K	
Wavelength	1.54178 A	
Crystal system, space group	Trigonal, P3(2)	
Unit cell dimensions	a = 9.6154(4) A	alpha = 90 deg.
	b = 9.6154(4) A	beta = 90 deg.
	c = 44.006(3) A	gamma = 120 deg.
Volume	3523.5(4) A^3	

Z, Calculated density	6, 1.232 Mg/m^3
Absorption coefficient	1.070 mm^-1
F(000)	1380
Crystal size	0.200 x 0.200 x 0.200 mm
Theta range for data collection	3.012 to 68.567 deg.
Limiting indices	-11<=h<=11, -10<=k<=11, -51<=l<=49
Reflections collected / unique	29000 / 8470 [R(int) = 0.0795]
Completeness to theta = 67.679	99.8 %
Absorption correction	Semi-empirical from equivalents
Refinement method	Full-matrix least-squares on F^2
Data / restraints / parameters	8470 / 3 / 577
Goodness-of-fit on F^2	1.011
Final R indices [I>2sigma(I)]	R1 = 0.0470, wR2 = 0.1279
R indices (all data)	R1 = 0.0632, wR2 = 0.1376
Absolute structure parameter	0.031(16)
Extinction coefficient	n/a
Largest diff. peak and hole	0.328 and -0.218 e. A^-3

2.10.3 X-Ray Crystallographic Data of Compound 5a

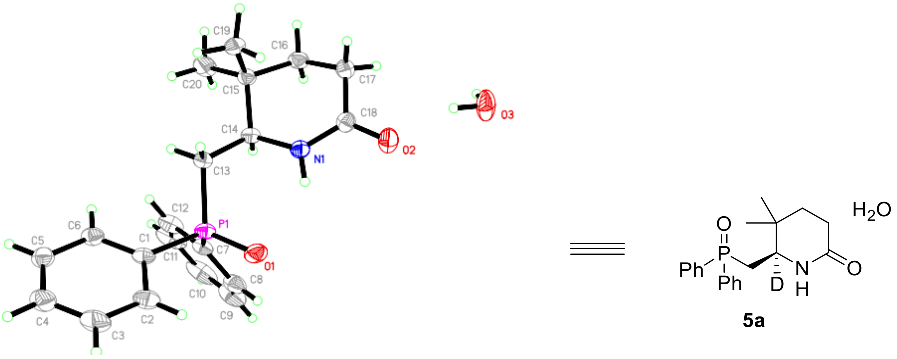

Supplementary Figure 25. X-Ray Structure of Compound **5a** (CCDC 2106903). Crystals suitable for X-ray structure analysis were obtained via recrystallization from methanol and pentane.

Supplementary Table 13. Crystal data and structure refinement for **5a**.

Identification code	**5a**
Empirical formula	$C_{20}H_{26}NO_3P$
Formula weight	359.39
Temperature	295(2) K
Wavelength	1.54178 A
Crystal system, space group	Hexagonal, P6(5)
Unit cell dimensions	a = 10.174(2) A alpha = 90.00(3) deg.
	b = 10.174(2) A beta = 90.00(3) deg.
	c = 34.443(7) A gamma = 120.00(3) deg.
Volume	3087.7(14) A^3
Z, Calculated density	6, 1.160 Mg/m^3
Absorption coefficient	1.318 mm^-1
F(000)	1152
Crystal size	0.200 x 0.200 x 0.200 mm
Theta range for data collection	5.019 to 68.322 deg.
Limiting indices	-12<=h<=12, -11<=k<=12, -41<=l<=41
Reflections collected / unique	32039 / 3772 [R(int) = 0.0447]
Completeness to theta = 67.679	99.9 %
Refinement method	Full-matrix least-squares on F^2
Data / restraints / parameters	3772 / 1 / 240
Goodness-of-fit on F^2	1.028
Final R indices [I>2sigma(I)]	R1 = 0.0329, wR2 = 0.0851
R indices (all data)	R1 = 0.0359, wR2 = 0.0884
Absolute structure parameter	0.038(8)
Extinction coefficient	n/a
Largest diff. peak and hole	0.128 and -0.119 e. A^-3

2.11. Computational Details

2.11.1. General Information

DFT calculations were performed in Gaussian 16 (C.01) program package.[24] All the structures were optimized using the ωB97xD functional[25] and the 6-31+G(d,p) basis set. Frequency calculations were carried out at that level of theory to analyze the nature of stationary points as transition states (one imaginary frequency) or minima (no imaginary frequencies). The Goodvibes program[26] was used to obtain the thermochemistry corrections at 283.15K, including quasi-harmonic corrections with the method proposed by Grimme,[27] with the cut-off value set as 100 wavenumbers. To further refine the potential energies, single point energy calculations were done with a larger basis set (6-311++G(3d,2p)) and PBE0[28] as the functional. Benchmarking calculations using M06-2X and ωB97xD were also done to further confirm the robustness of the method, showing similar performance for this reaction. The ωB97xD functional was selected for optimizations due to its general good performance in geometry optimization.[29] Implicit solvation was included in all the calculations, including geometry optimization-frequency calculations and single points, using the CPCM implicit solvent model[30,31] and toluene as the solvent.

Conformation of the peptide catalyst was chosen based on the structural analysis of similar tetrapeptide catalysts by X-ray and DFT calculations reported by Miller and co-workers.[32] Thiol **S6**, which showed the same enantioselectivity as thiol **S5**, was thus chosen as the catalyst for our calculation.

NCI analyses were performed using the NCIplot program[33,34] and all the 3D representations were prepared in PyMOL.

2.11.2. Benchmarking of the computational method

Supplementary Table 14. Comparison of different DFT methods on the **TS-*Re*** vs **TS-*Si*** activation energy difference and calculated enantiomeric ratio. All energies in kcal/mol.

Method	$\Delta\Delta G^{\ddagger}_{Re\text{-}Si}$	er
PBE0	1.24	90:10
M062X	2.15	98:2
ωB97xD	2.14	98:2
Exp.	1.45	93:7

2.12 NMR Spectra and HPLC Spectra

^{1}H NMR (400 MHz, room temperature, $CDCl_3$)

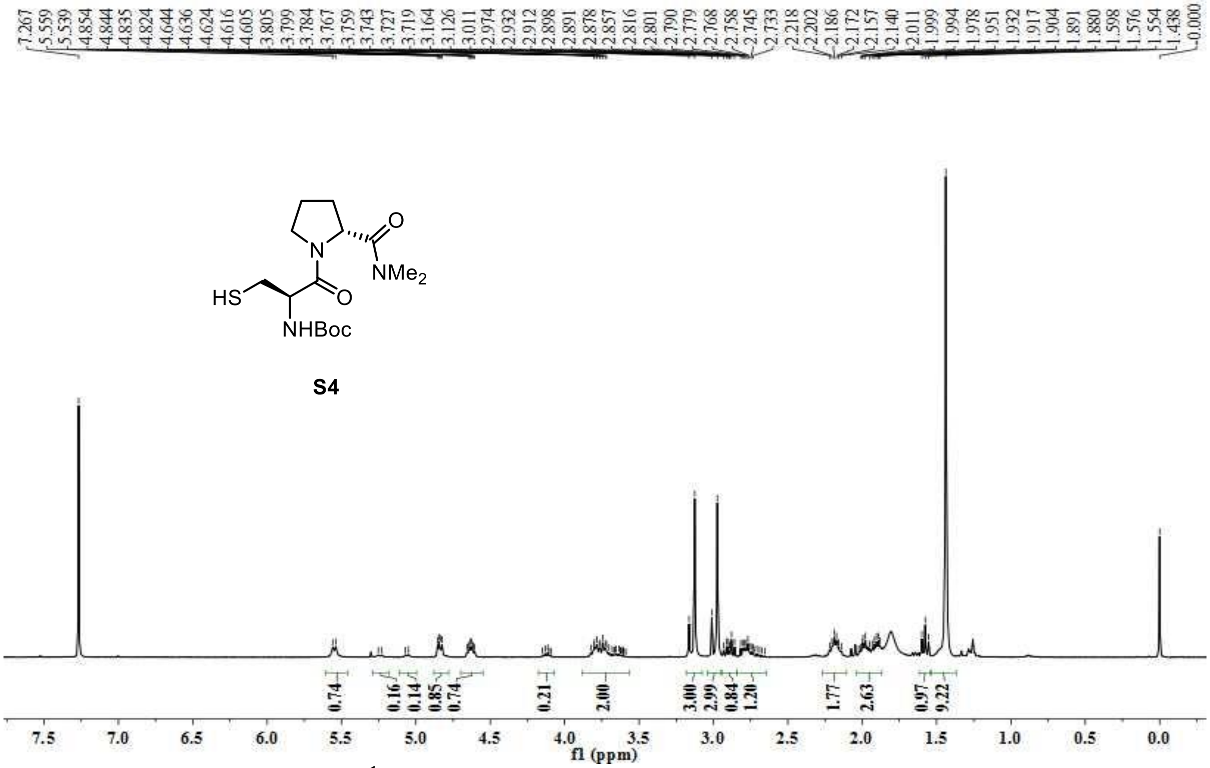

Supplementary Figure 26. ^{1}H NMR spectrum of compound **S4**

^{13}C NMR (100 MHz, room temperature, $CDCl_3$)

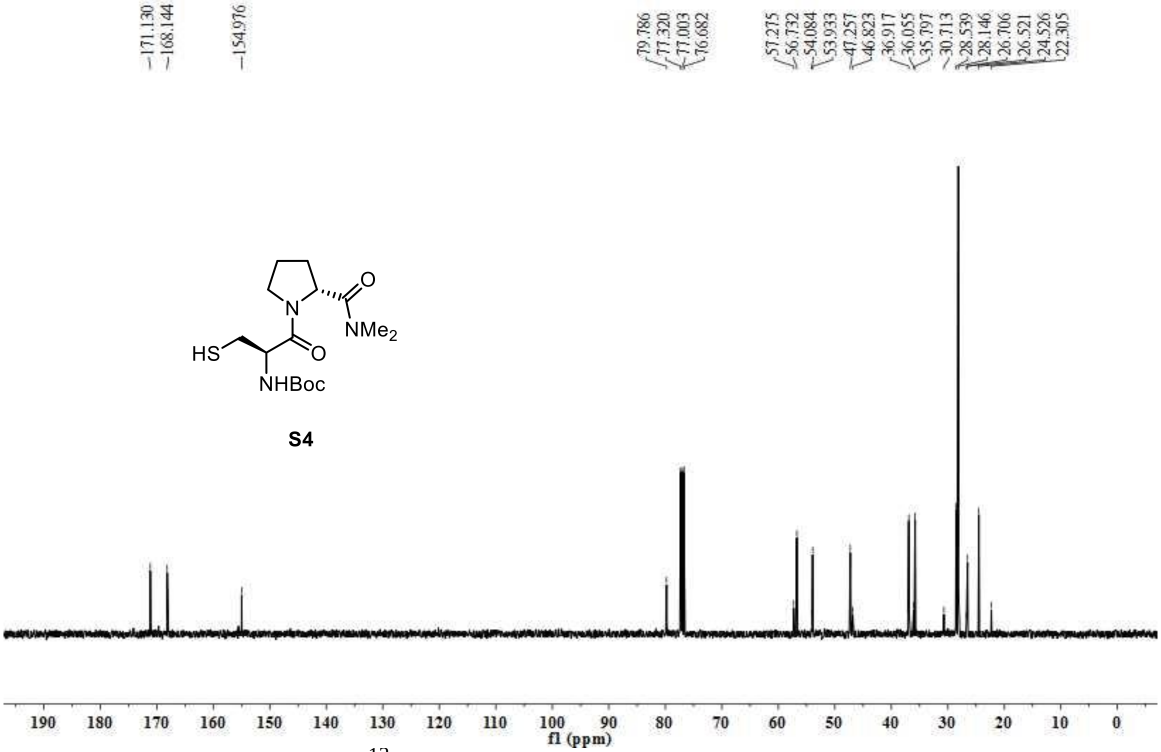

Supplementary Figure 27. ^{13}C NMR spectrum of compound **S4**

^{1}H NMR (400 MHz, room temperature, $CDCl_3$)

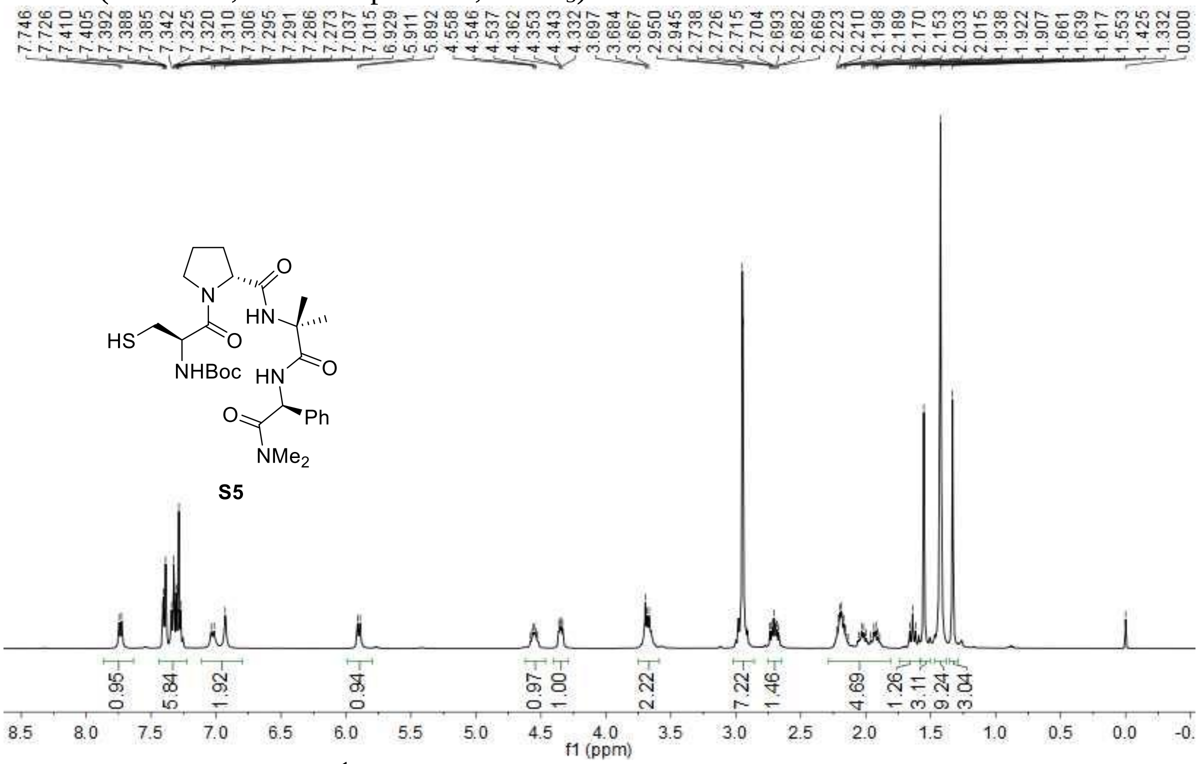

Supplementary Figure 28. ^{1}H NMR spectrum of compound **S5**

^{13}C NMR (100 MHz, room temperature, $CDCl_3$)

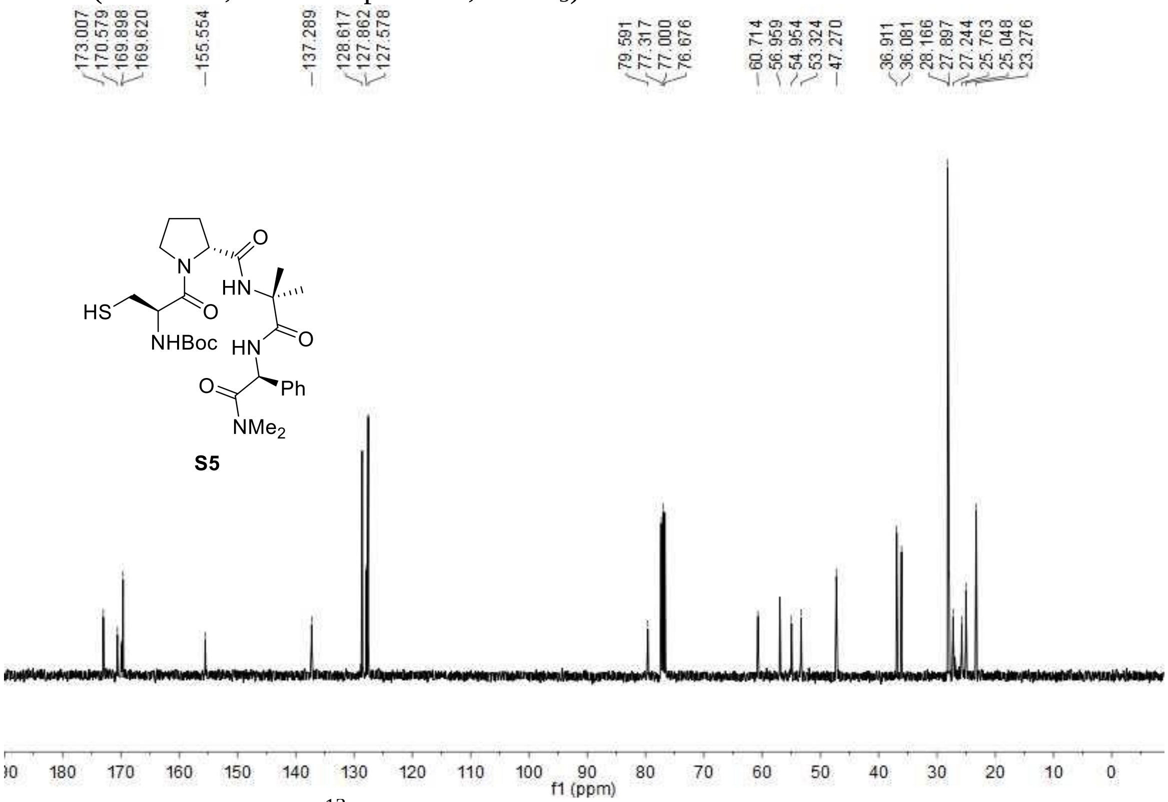

Supplementary Figure 29. ^{13}C NMR spectrum of compound **S5**

^{1}H NMR (400 MHz, room temperature, $CDCl_3$)

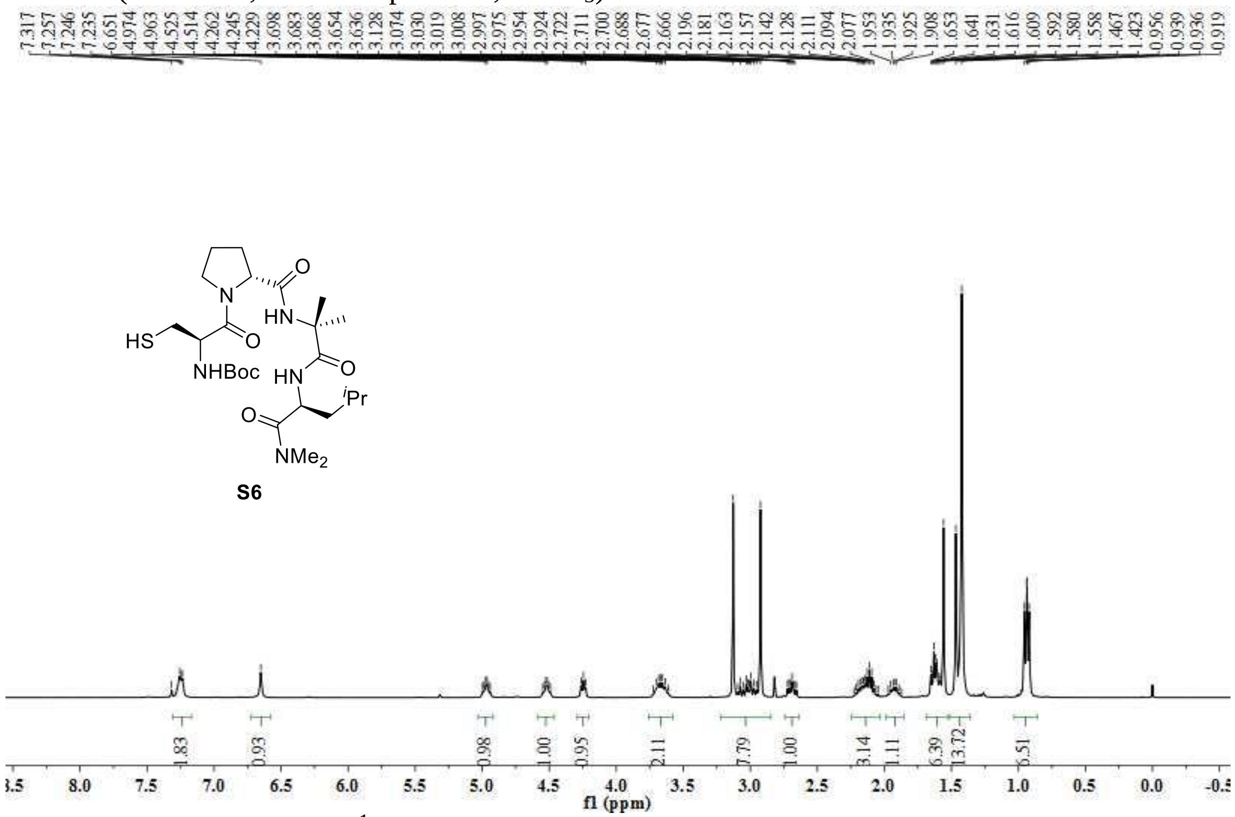

Supplementary Figure 30. ^{1}H NMR spectrum of compound **S6**

^{13}C NMR (100 MHz, room temperature, $CDCl_3$)

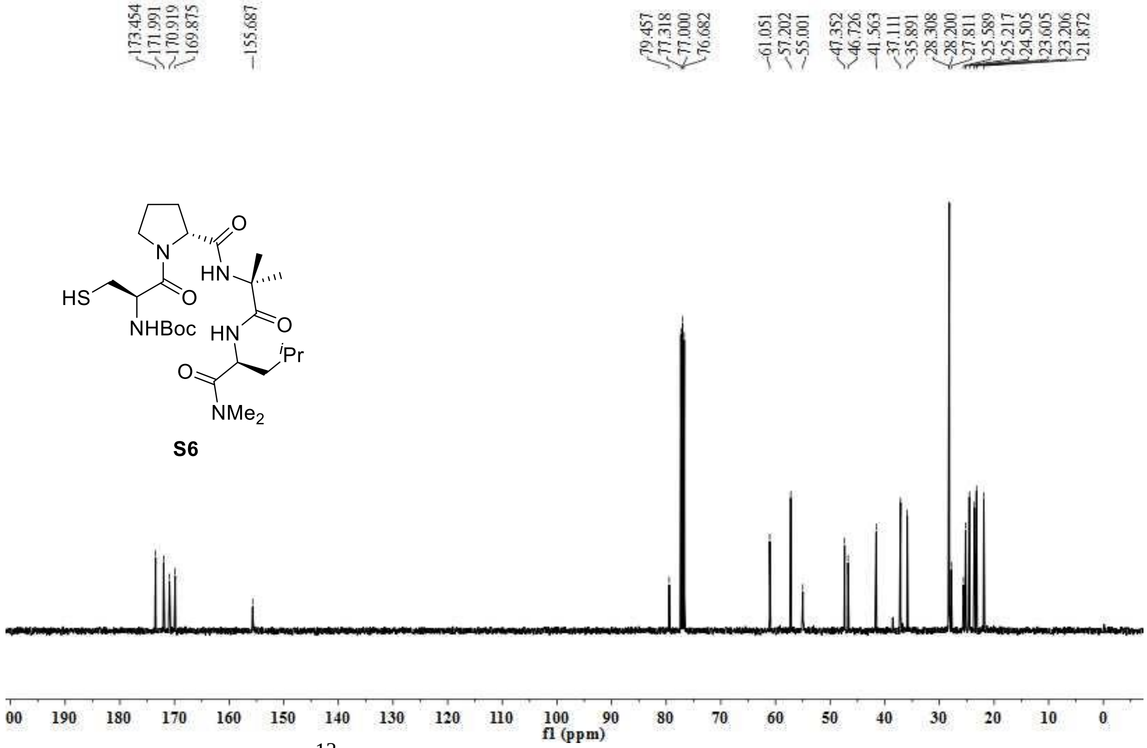

Supplementary Figure 31. ^{13}C NMR spectrum of compound **S6**

^{1}H NMR (400 MHz, room temperature, $CDCl_3$)

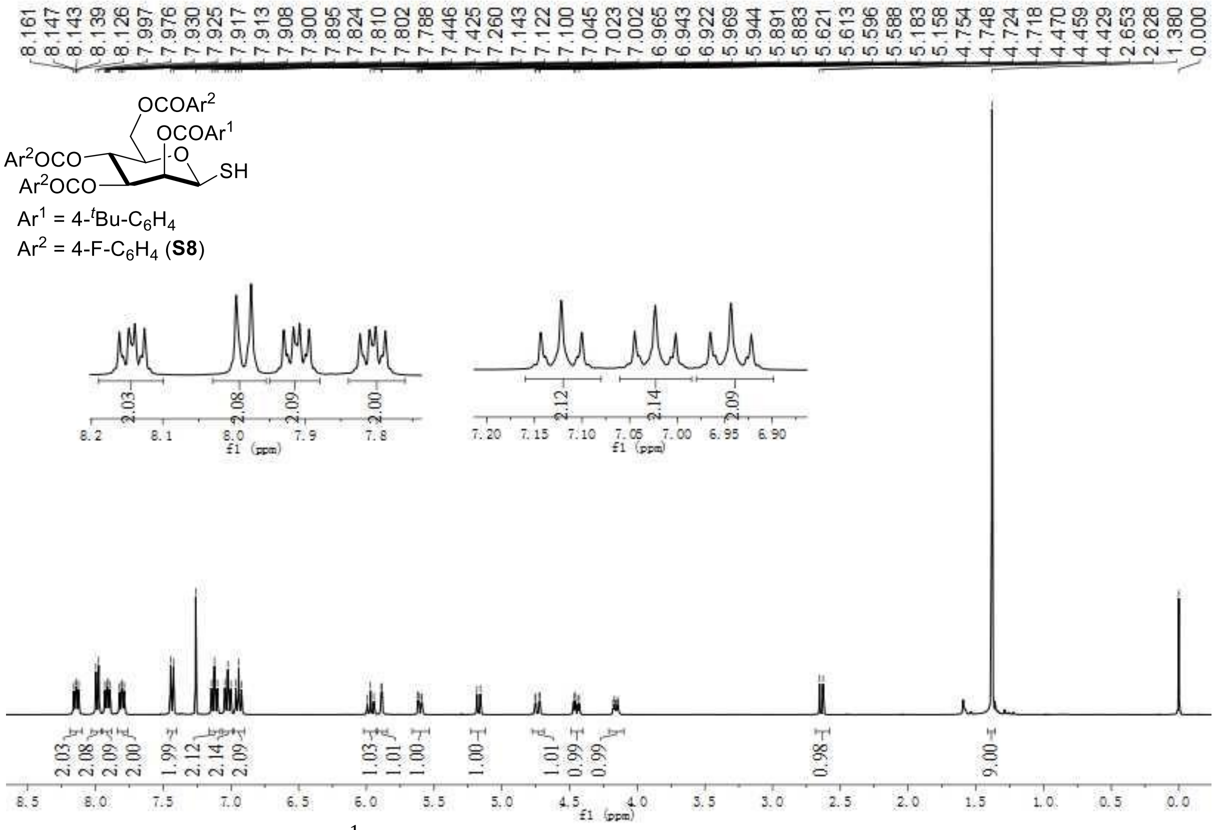

Supplementary Figure 32. ^{1}H NMR spectrum of compound **S8**

^{13}C NMR (100 MHz, room temperature, $CDCl_3$)

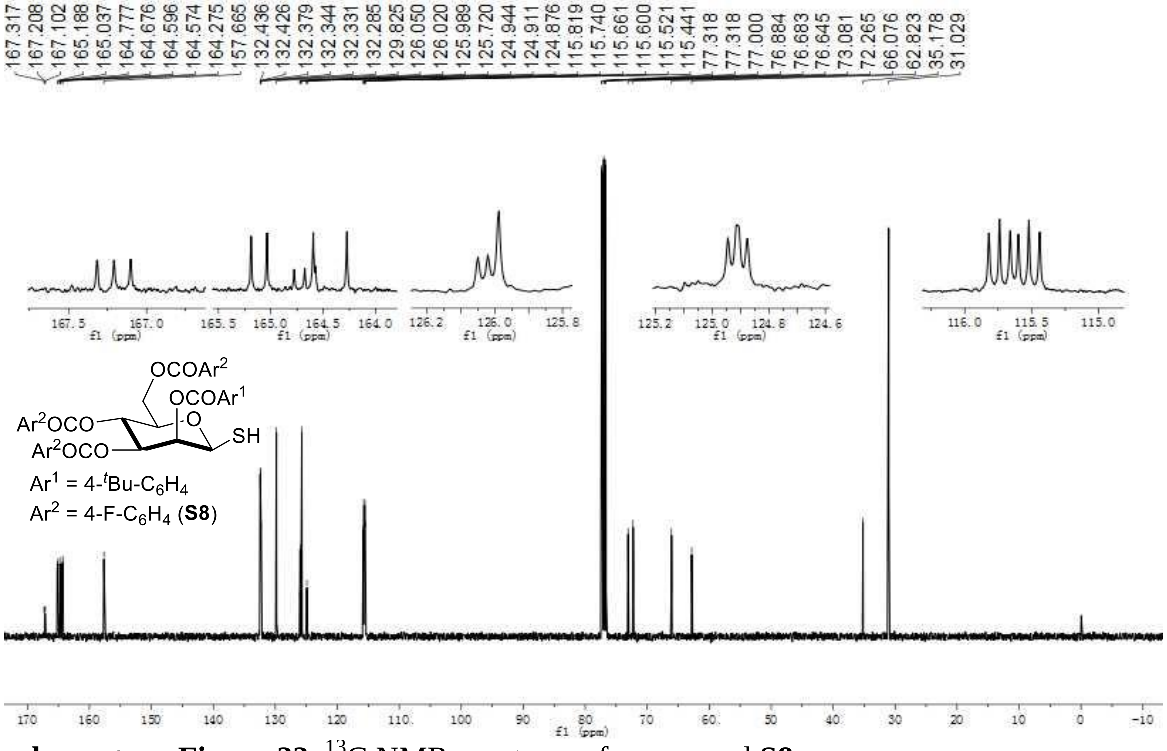

Supplementary Figure 33. ^{13}C NMR spectrum of compound **S8**

^{19}F NMR (376 MHz, room temperature, $CDCl_3$)

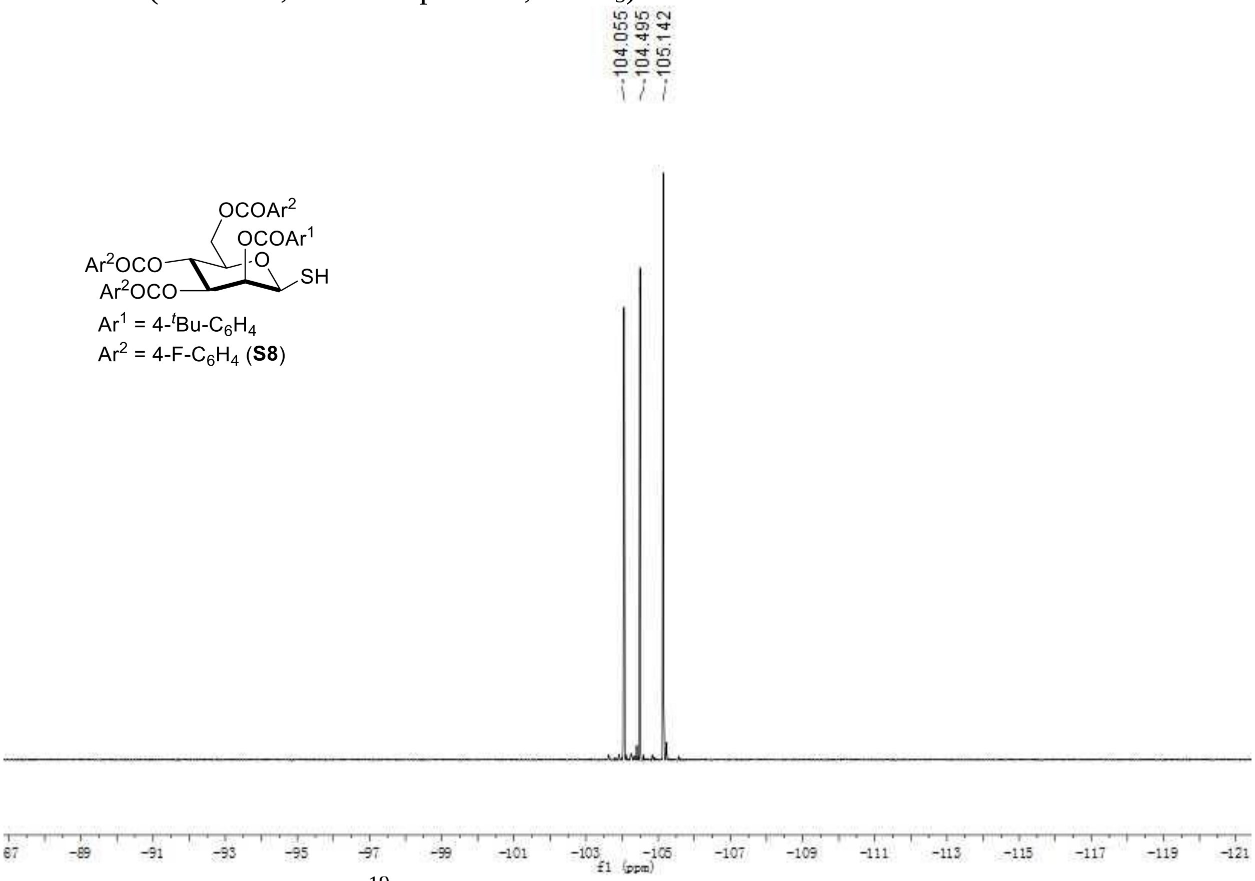

Supplementary Figure 34. ^{19}F NMR spectrum of compound **S8**

^{1}H NMR (400 MHz, room temperature, $CDCl_3$)

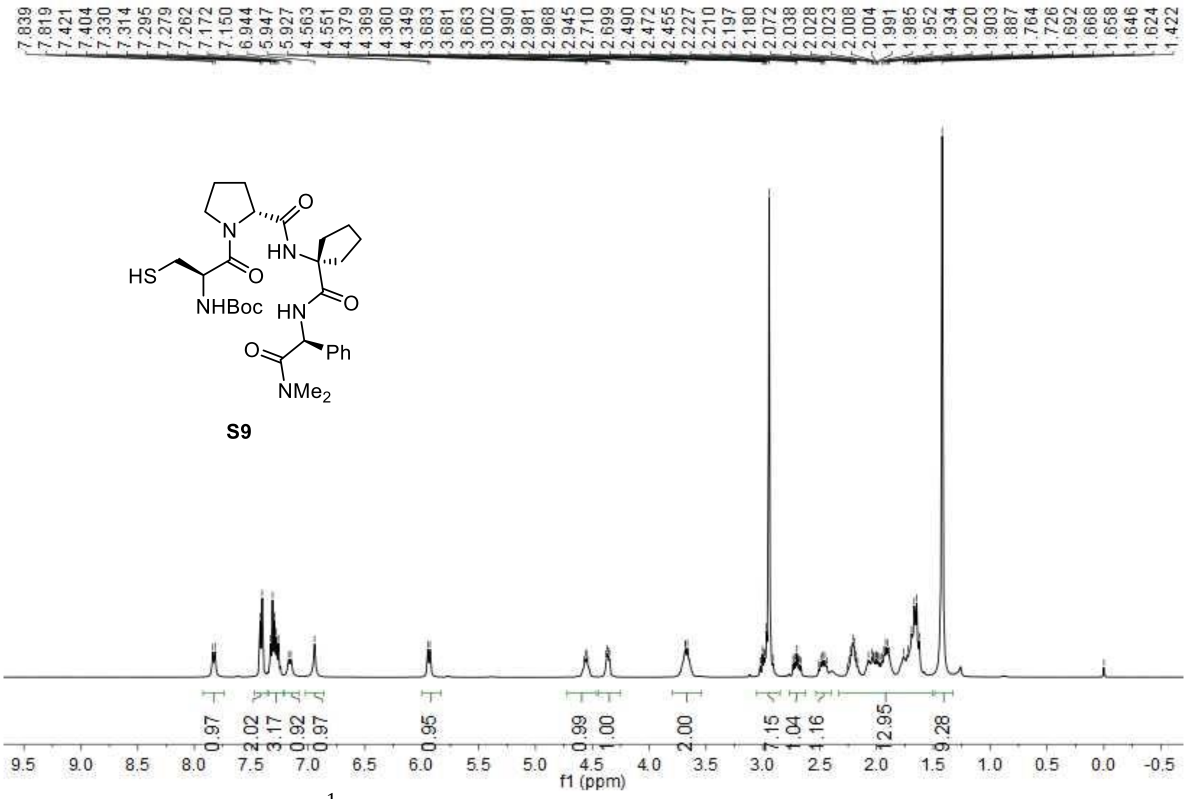

Supplementary Figure 35. ^{1}H NMR spectrum of compound **S9**

^{13}C NMR (100 MHz, room temperature, $CDCl_3$)

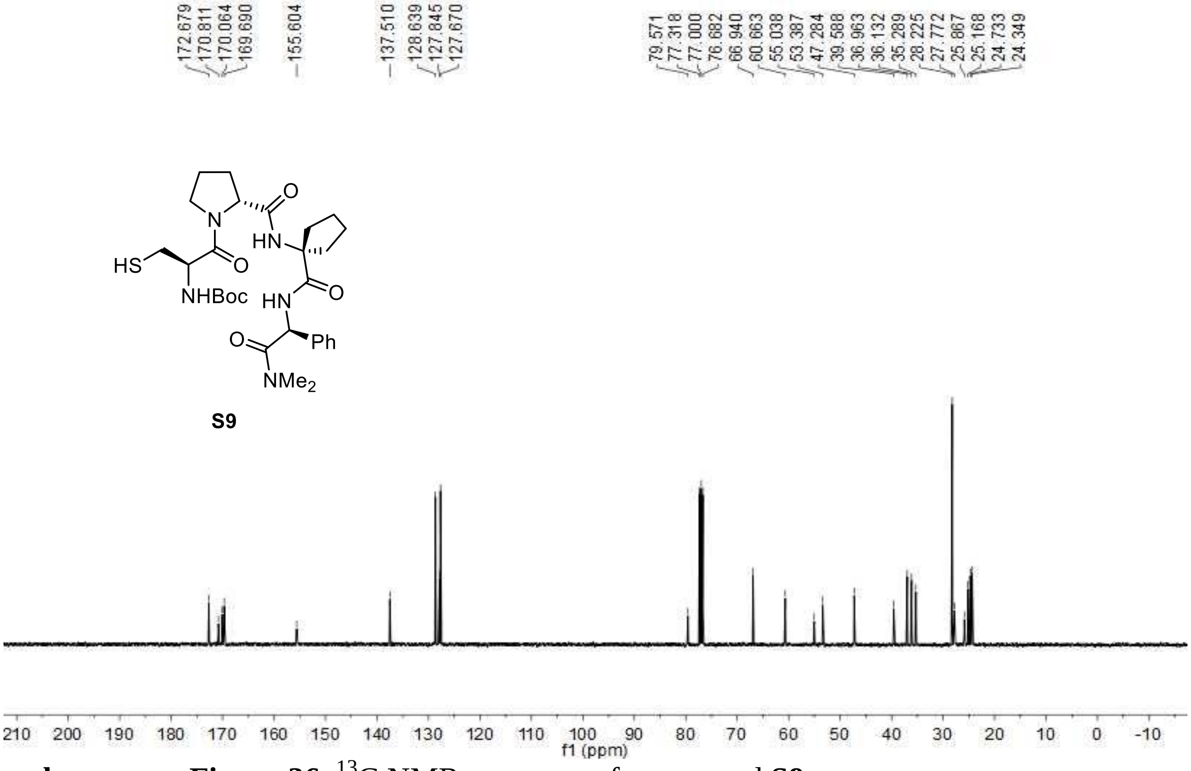

Supplementary Figure 36. ^{13}C NMR spectrum of compound **S9**

^{1}H NMR (400 MHz, room temperature, $CDCl_3$)

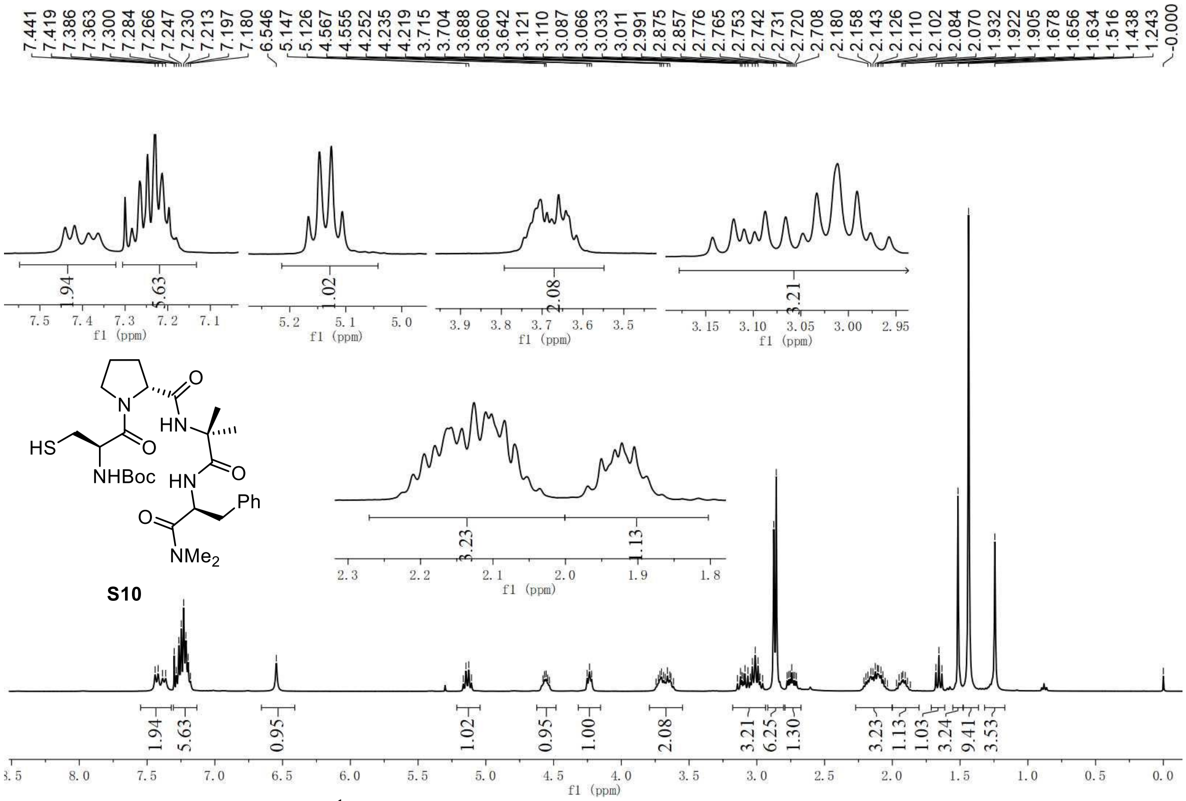

Supplementary Figure 37. ^{1}H NMR spectrum of compound **S10**

^{13}C NMR (100 MHz, room temperature, $CDCl_3$)

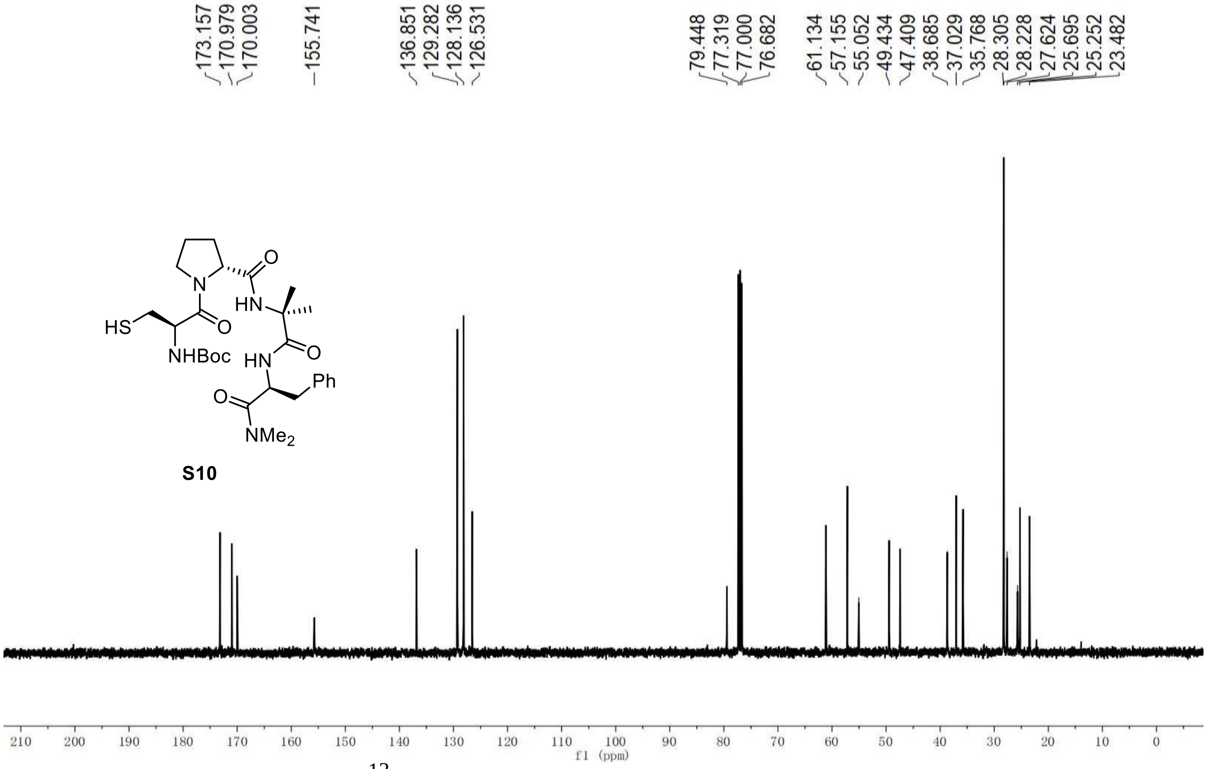

Supplementary Figure 38. ^{13}C NMR spectrum of compound **S10**

^{1}H NMR (400 MHz, room temperature, $CDCl_3$)

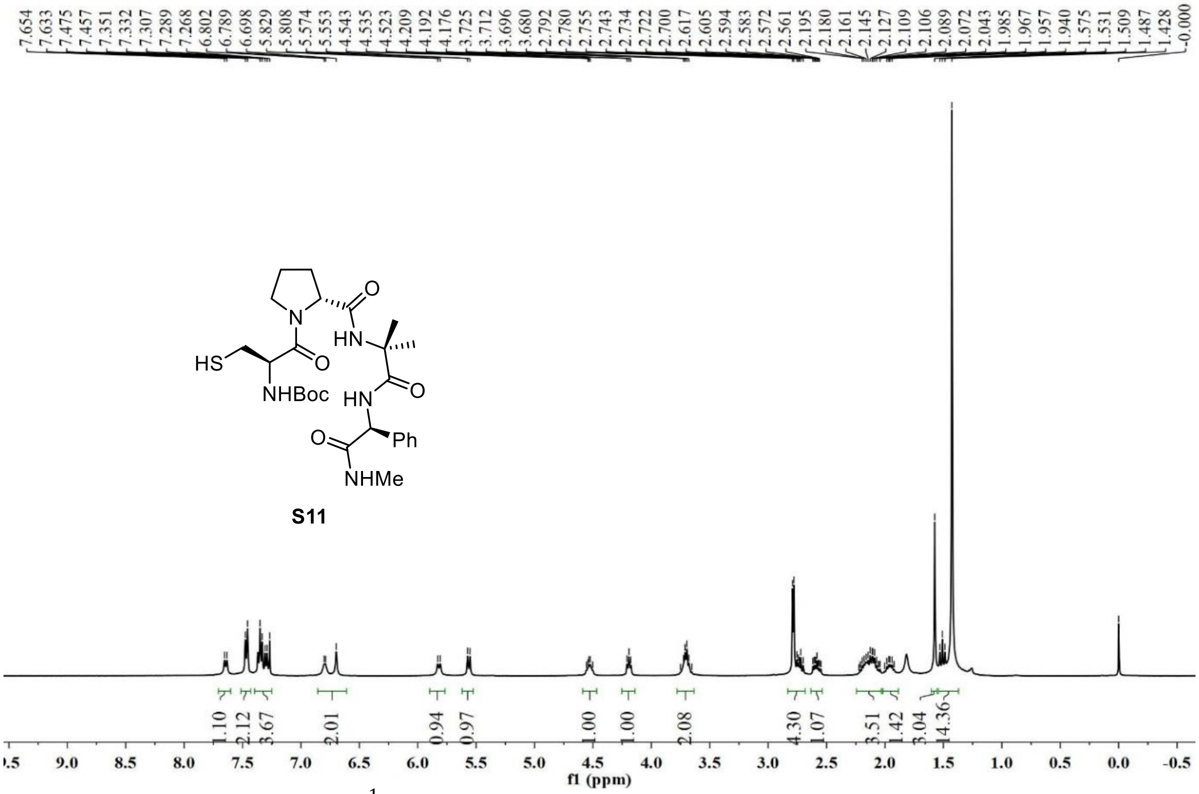

Supplementary Figure 39. ^{1}H NMR spectrum of compound **S11**

^{13}C NMR (100 MHz, room temperature, $CDCl_3$)

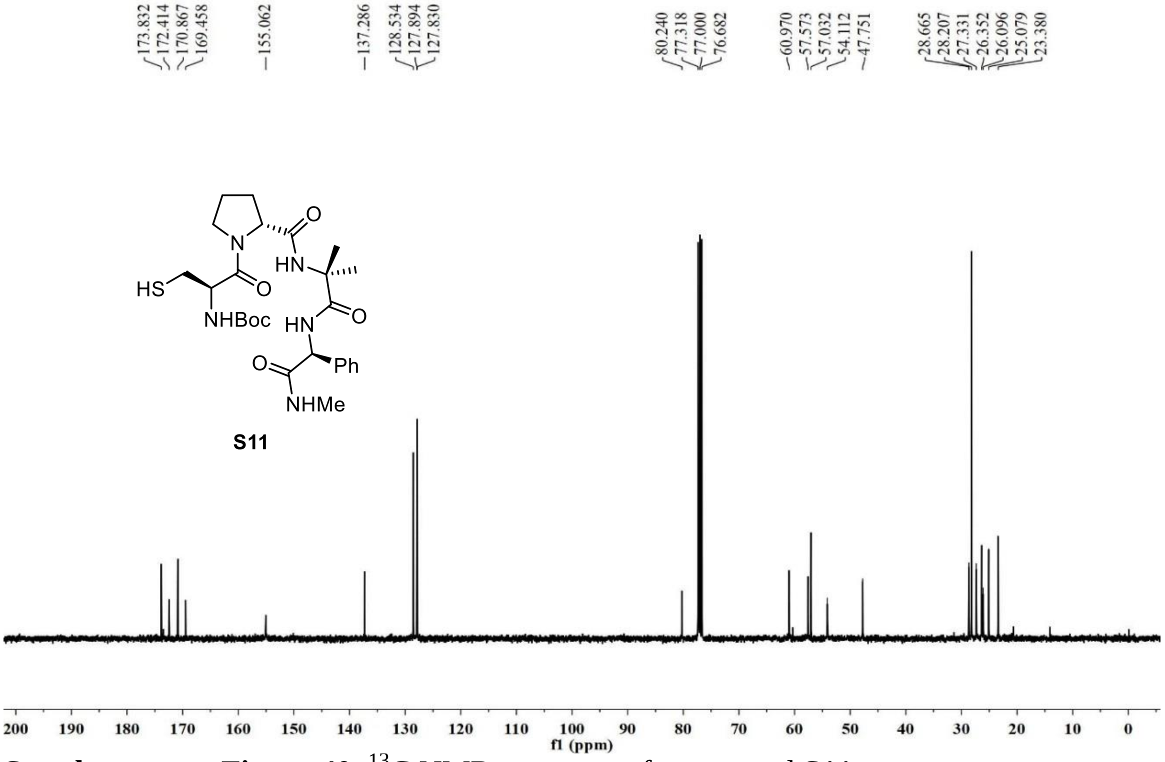

Supplementary Figure 40. ^{13}C NMR spectrum of compound **S11**

^{1}H NMR (400 MHz, room temperature, $CDCl_3$)

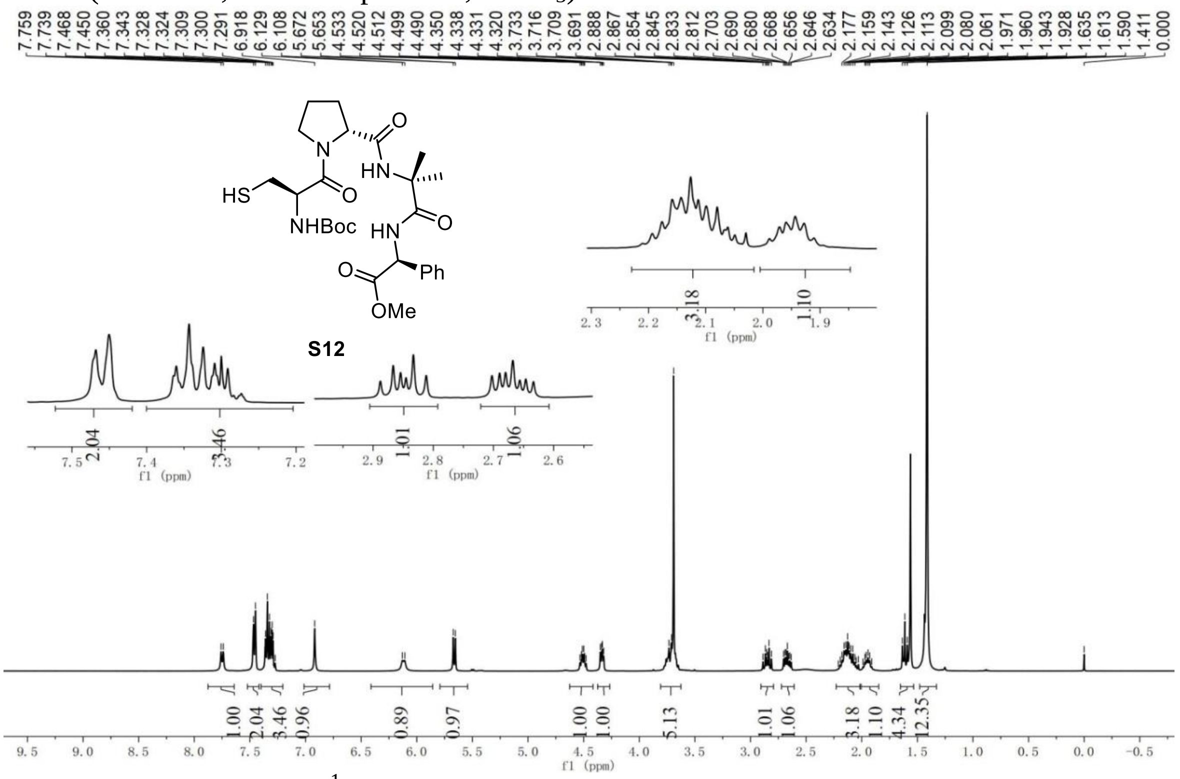

Supplementary Figure 41. ^{1}H NMR spectrum of compound **S12**

^{13}C NMR (100 MHz, room temperature, $CDCl_3$)

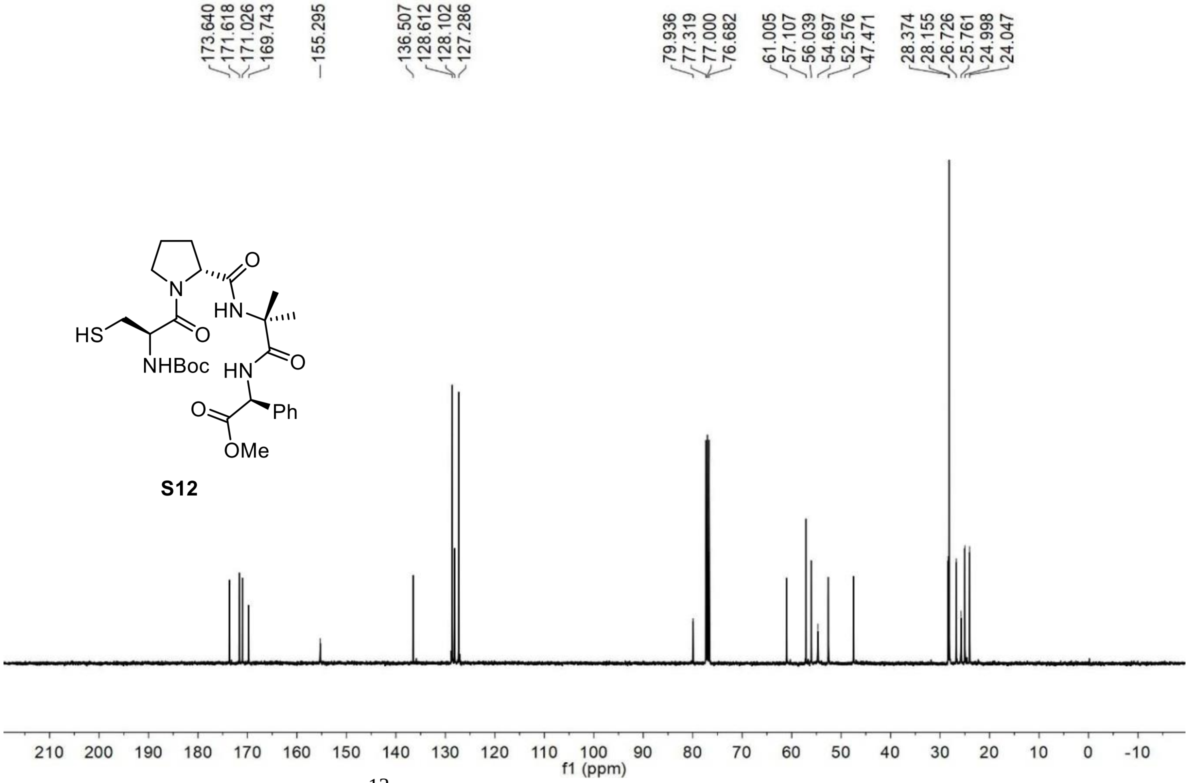

Supplementary Figure 42. ^{13}C NMR spectrum of compound **S12**

^{1}H NMR (400 MHz, room temperature, $CDCl_3$)

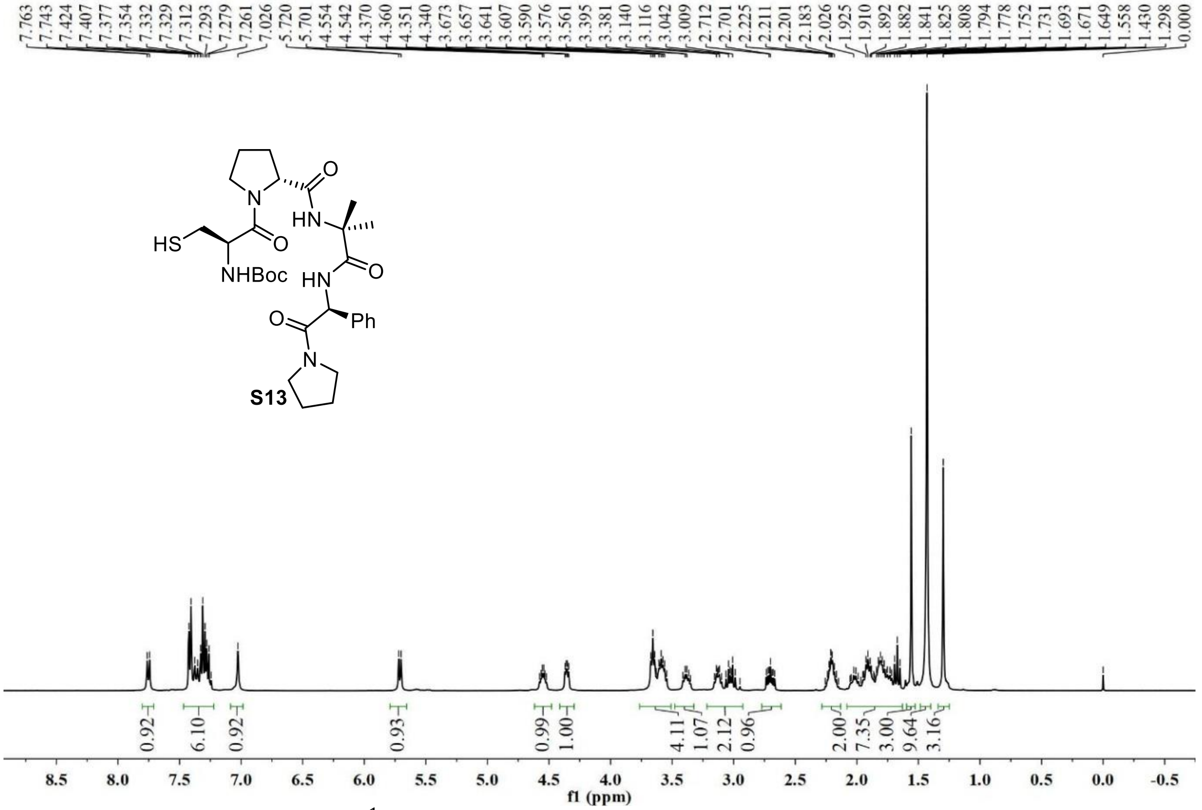

Supplementary Figure 43. ^{1}H NMR spectrum of compound **S13**

^{13}C NMR (100 MHz, room temperature, $CDCl_3$)

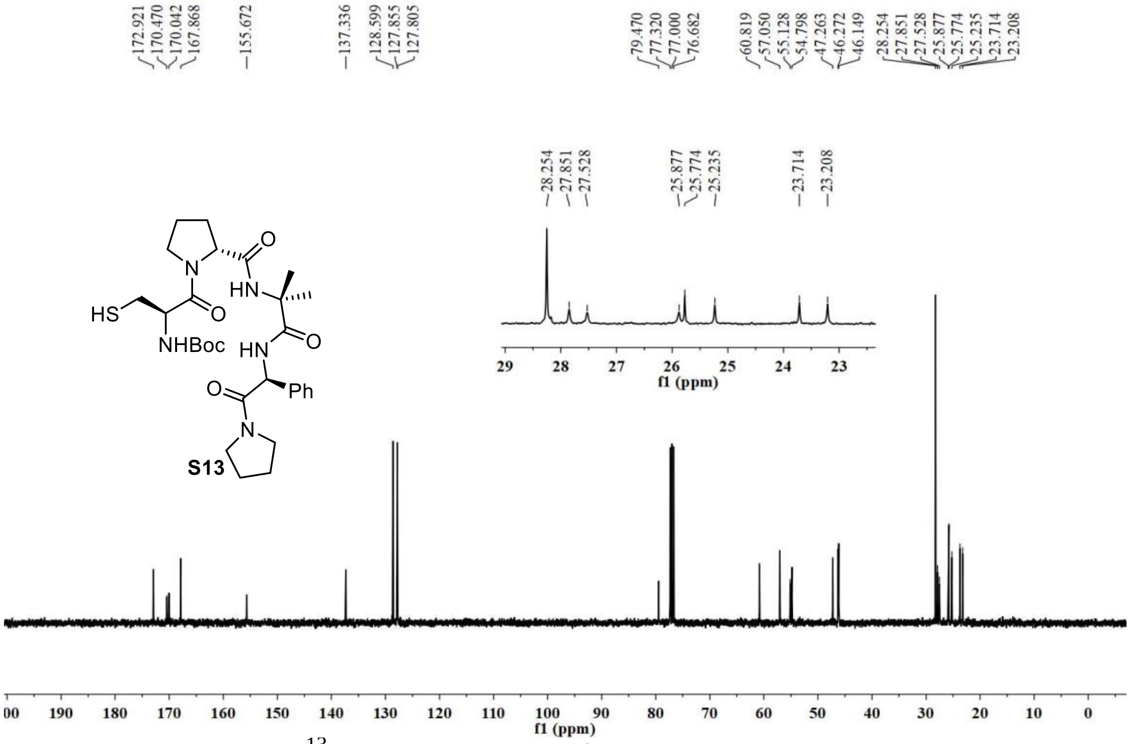

Supplementary Figure 44. ^{13}C NMR spectrum of compound **S13**

^{1}H NMR (400 MHz, room temperature, $CDCl_3$)

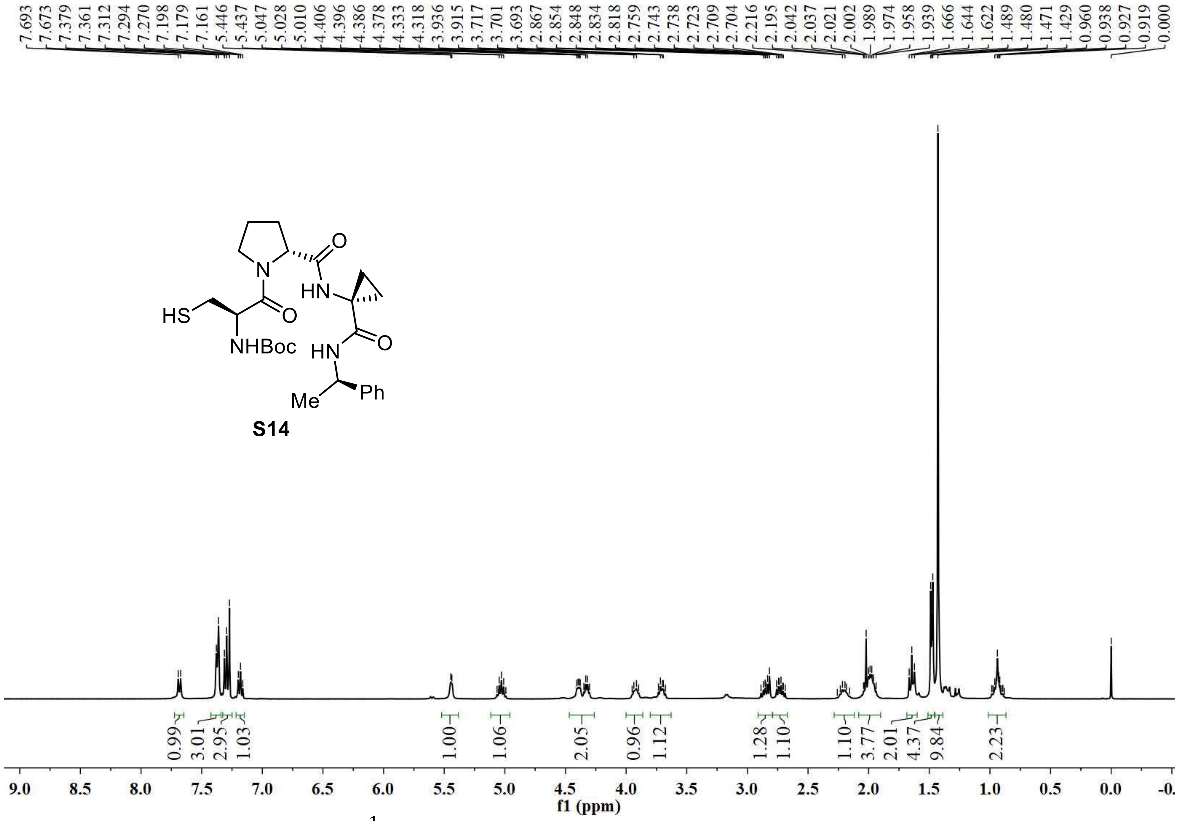

Supplementary Figure 45. ^{1}H NMR spectrum of compound **S14**

^{13}C NMR (100 MHz, room temperature, $CDCl_3$)

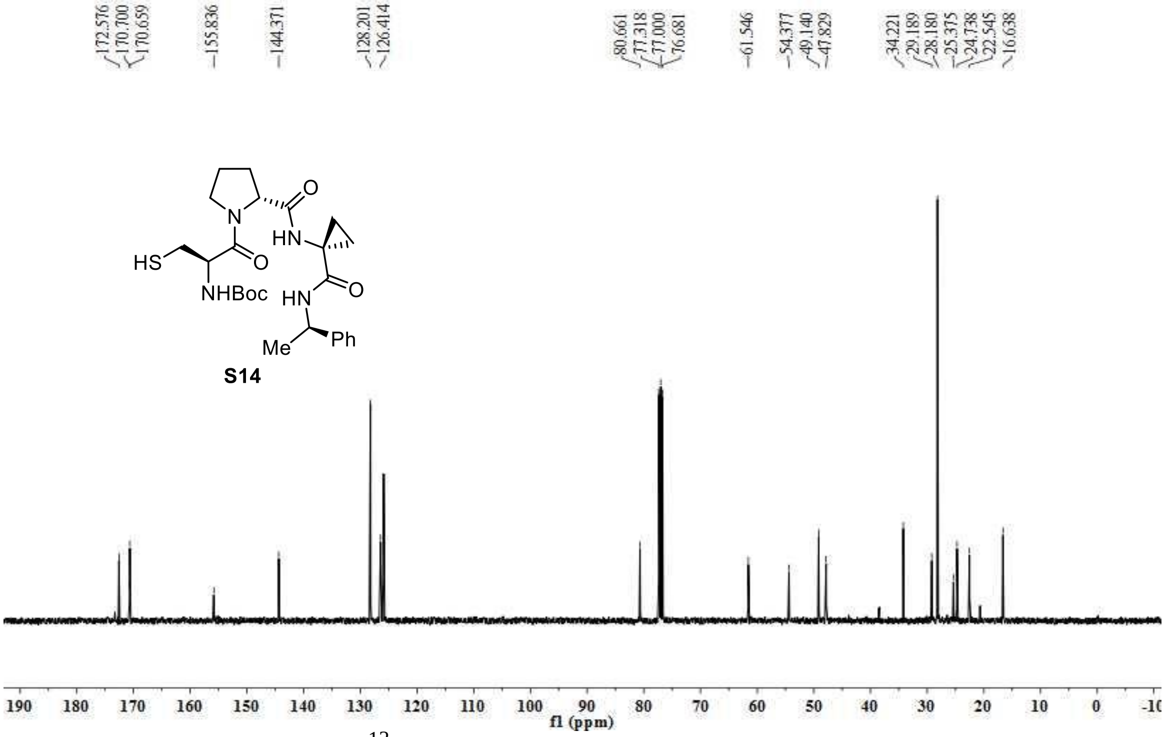

Supplementary Figure 46. ^{13}C NMR spectrum of compound **S14**

^{1}H NMR (400 MHz, room temperature, $CDCl_3$)

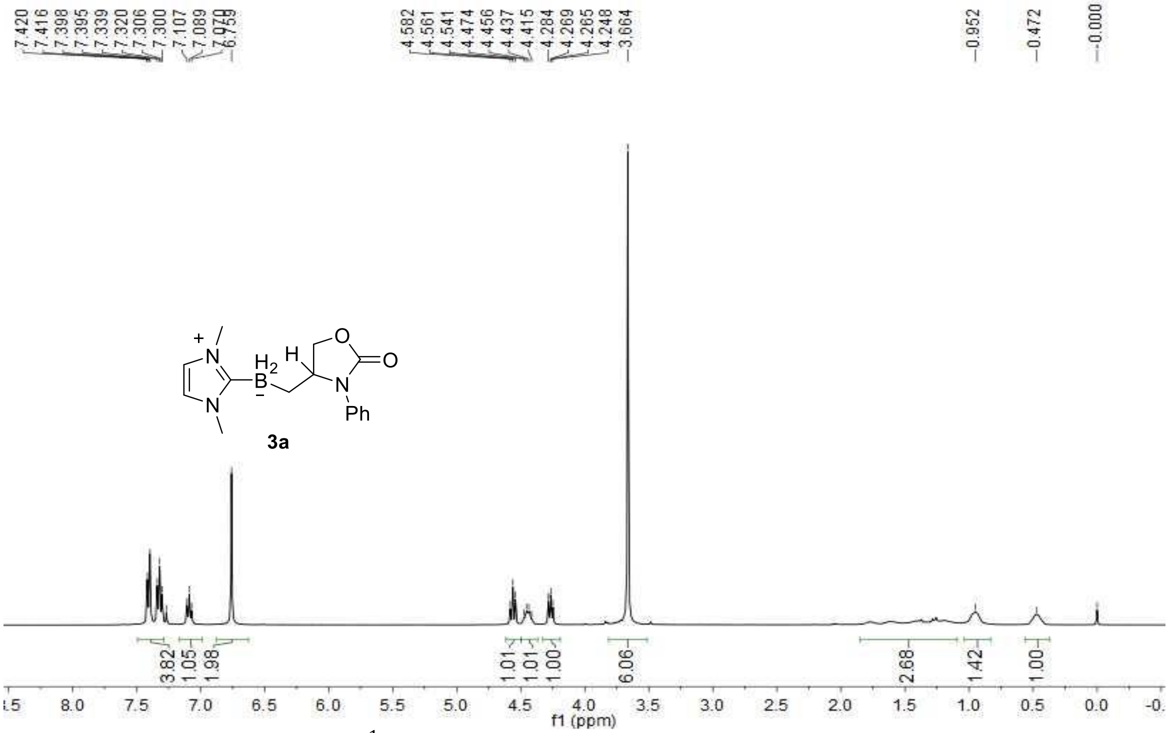

Supplementary Figure 47. ^{1}H NMR spectrum of racemic and non-deuterated compound **3a**

^{1}H NMR (400 MHz, room temperature, $CDCl_3$)

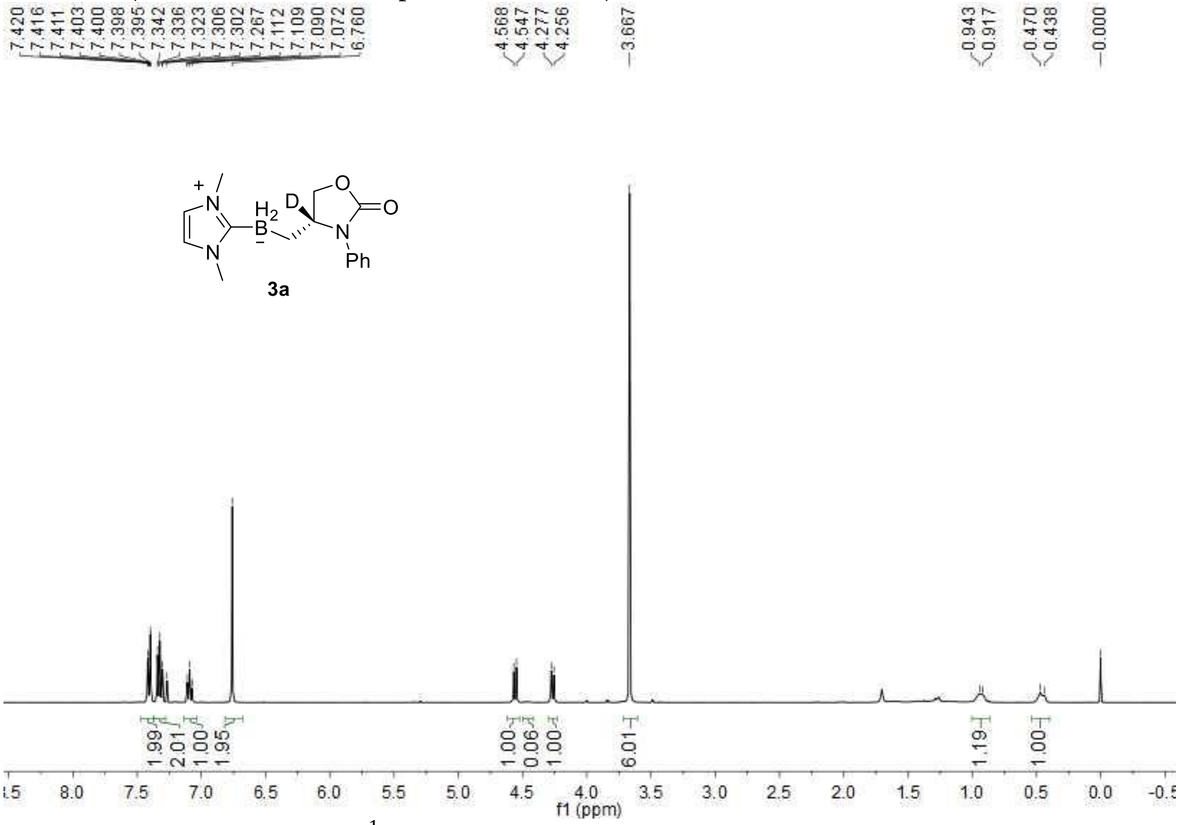

Supplementary Figure 48. ^{1}H NMR spectrum of compound **3a**

^{13}C NMR (100 MHz, room temperature, $CDCl_3$)

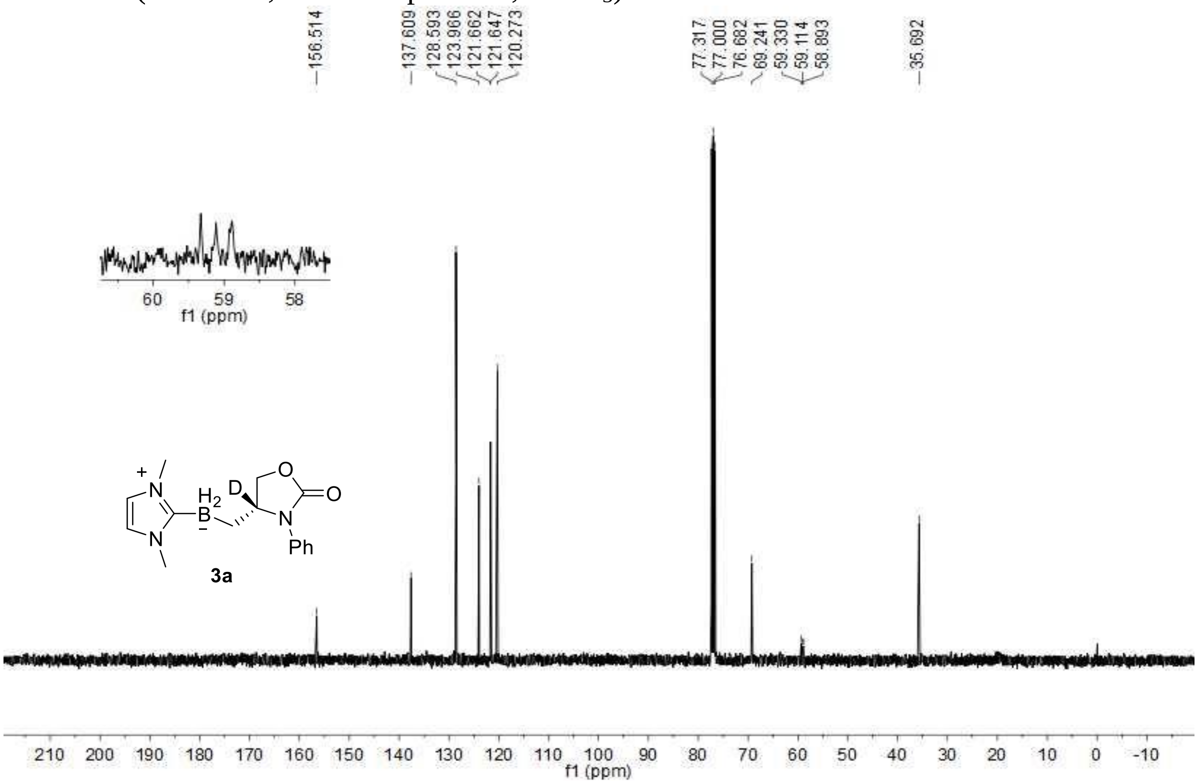

Supplementary Figure 49. ^{13}C NMR spectrum of compound **3a**

^{11}B NMR (128 MHz, room temperature, $CDCl_3$)

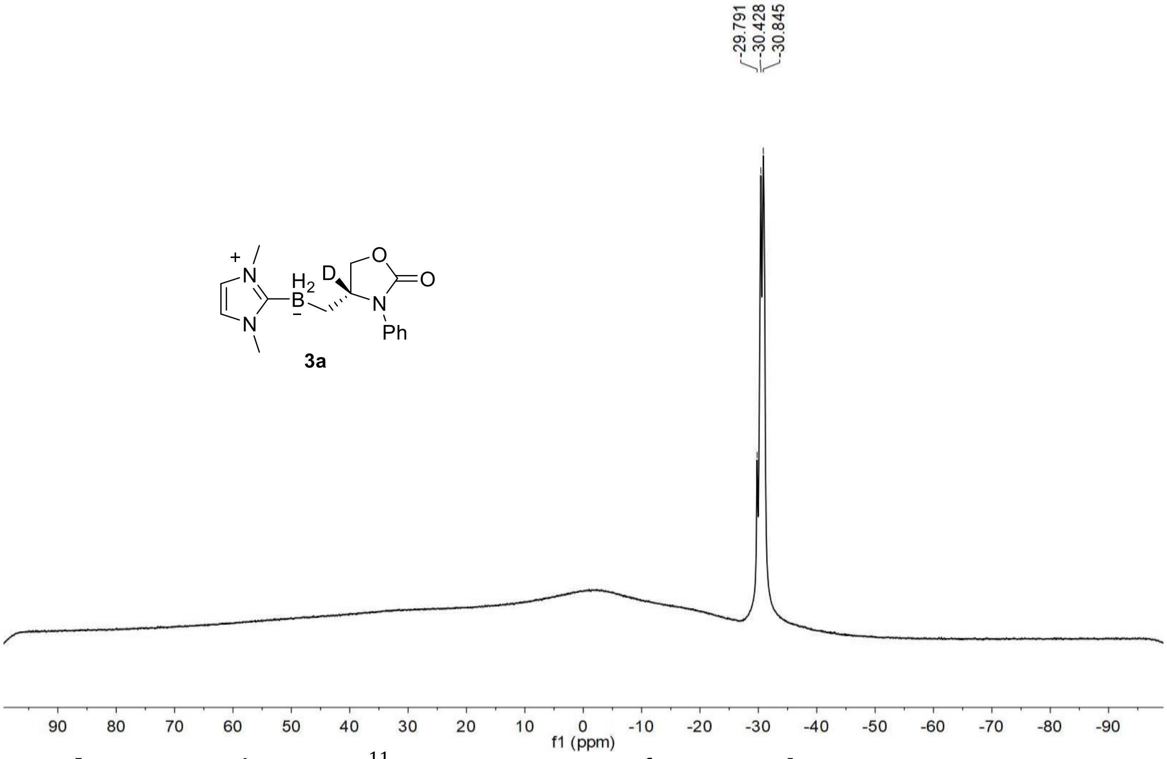

Supplementary Figure 50. ^{11}B NMR spectrum of compound **3a**

HPLC spectrum of racemic **3a**

Vial#	: 11
Data File	: SQL-6-280-2-RACEMIC(1).lcd
Method File	: 4OD-H-60-0.5-214.lcm
Date Acquired	: 12/9/2021 10:34:07 AM
Date Processed	: 1/25/2022 12:35:14 AM

<Chromatogram View>

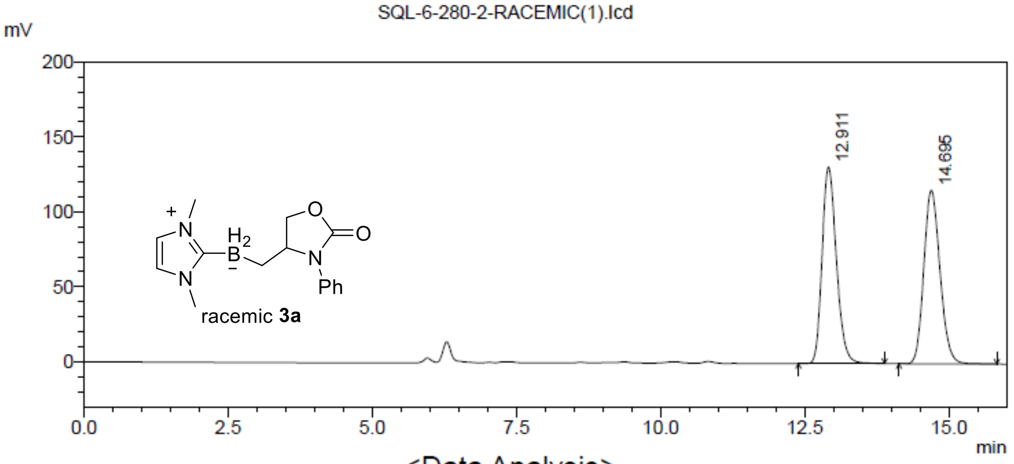

<Data Analysis>

Detector A 214nm

Pesk #	Ret. Time	Height	Area	Area%
1	12.911	131195	2261994	49.950
2	14.695	115749	2266525	50.050
Total		246943	4528519	100.000

Supplementary Figure 51. HPLC spectrum of racemic **3a**

HPLC spectrum of **3a**

Sample Name	:
Tray#	: 1
Vial#	: 14
Data File	: sql-6-354-CHIRAL(1).lcd
Method File	: 4OD-H-60-0.5-214.lcm
Date Acquired	: 12/31/2021 7:17:22 PM
Date Processed	: 1/25/2022 12:34:49 AM

<Chromatogram View>

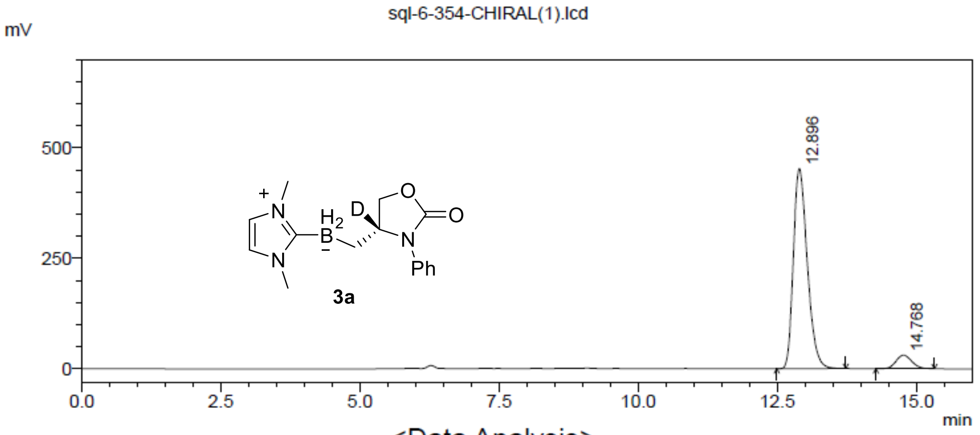

<Data Analysis>

Detector A 214nm

Pesk #	Ret. Time	Height	Area	Area%
1	12.896	452969	7902770	92.864
2	14.768	30863	607314	7.136
Total		483832	8510084	100.000

Supplementary Figure 52. HPLC spectrum of **3a**

^{1}H NMR (400 MHz, room temperature, $CDCl_3$)

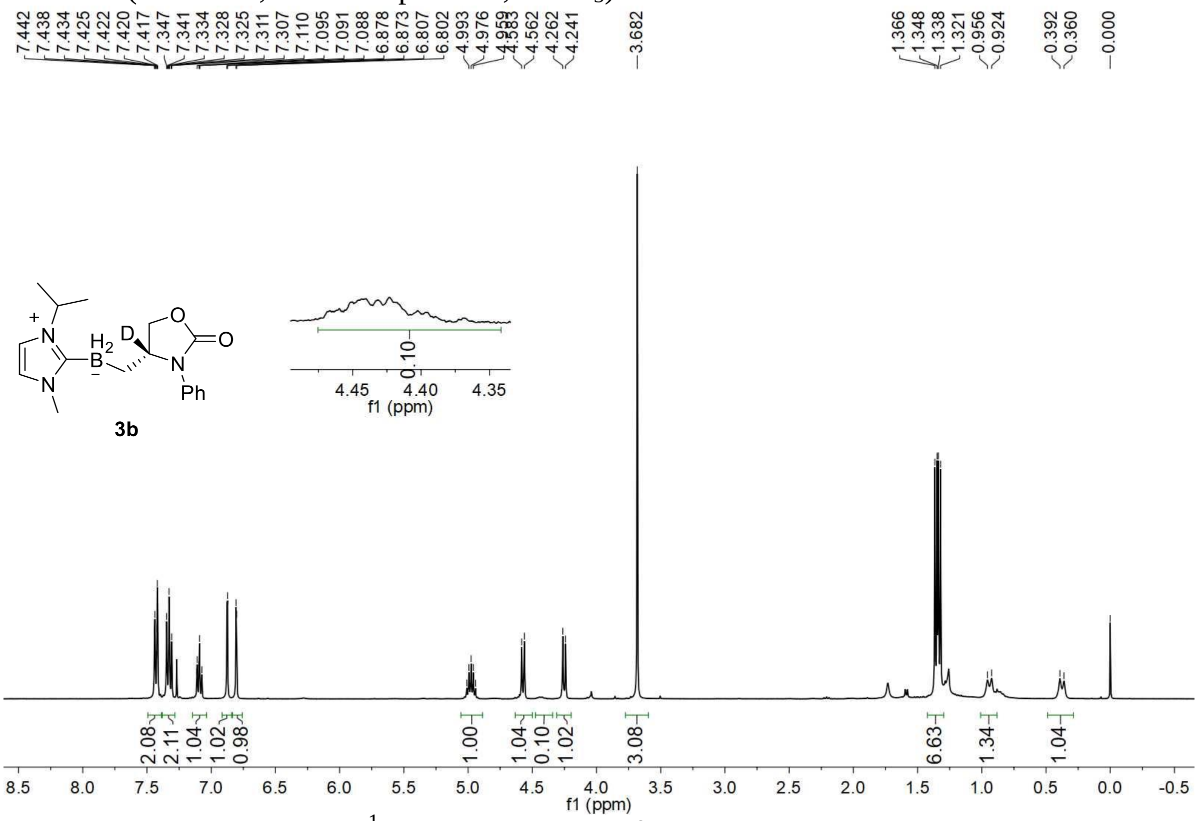

Supplementary Figure 53. ^{1}H NMR spectrum of compound **3b**

^{13}C NMR (100 MHz, room temperature, $CDCl_3$)

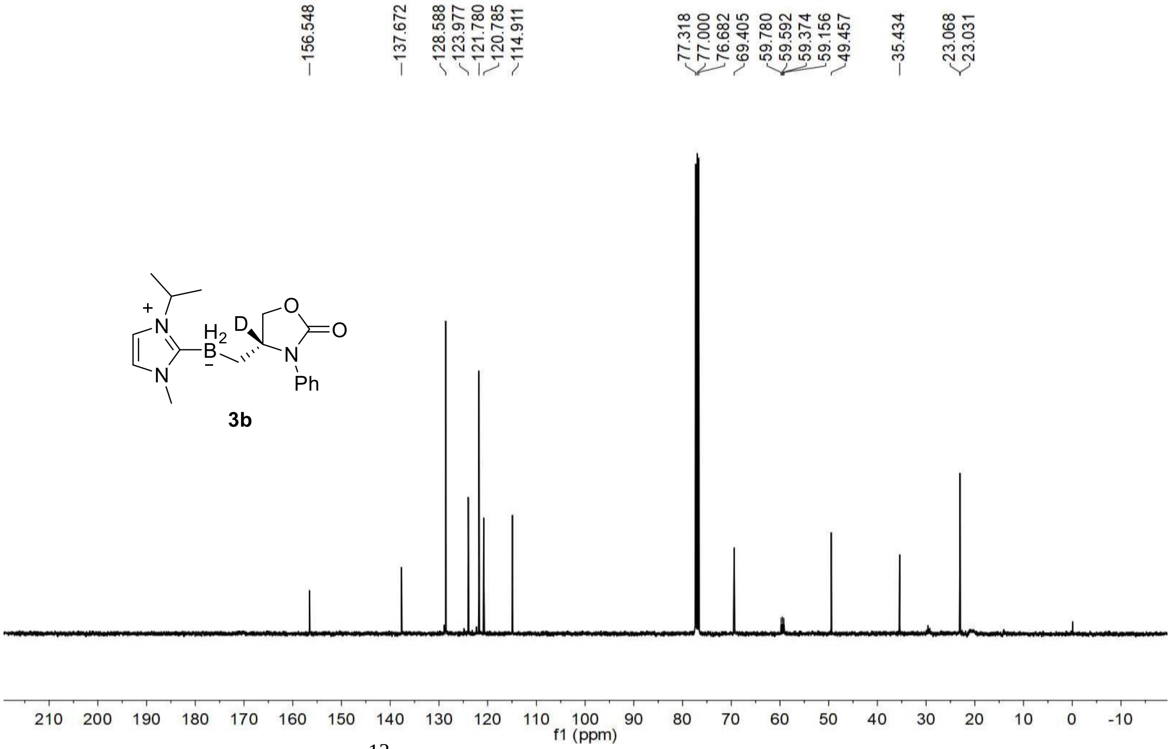

Supplementary Figure 54. ^{13}C NMR spectrum of compound **3b**

^{11}B NMR (128 MHz, room temperature, $CDCl_3$)

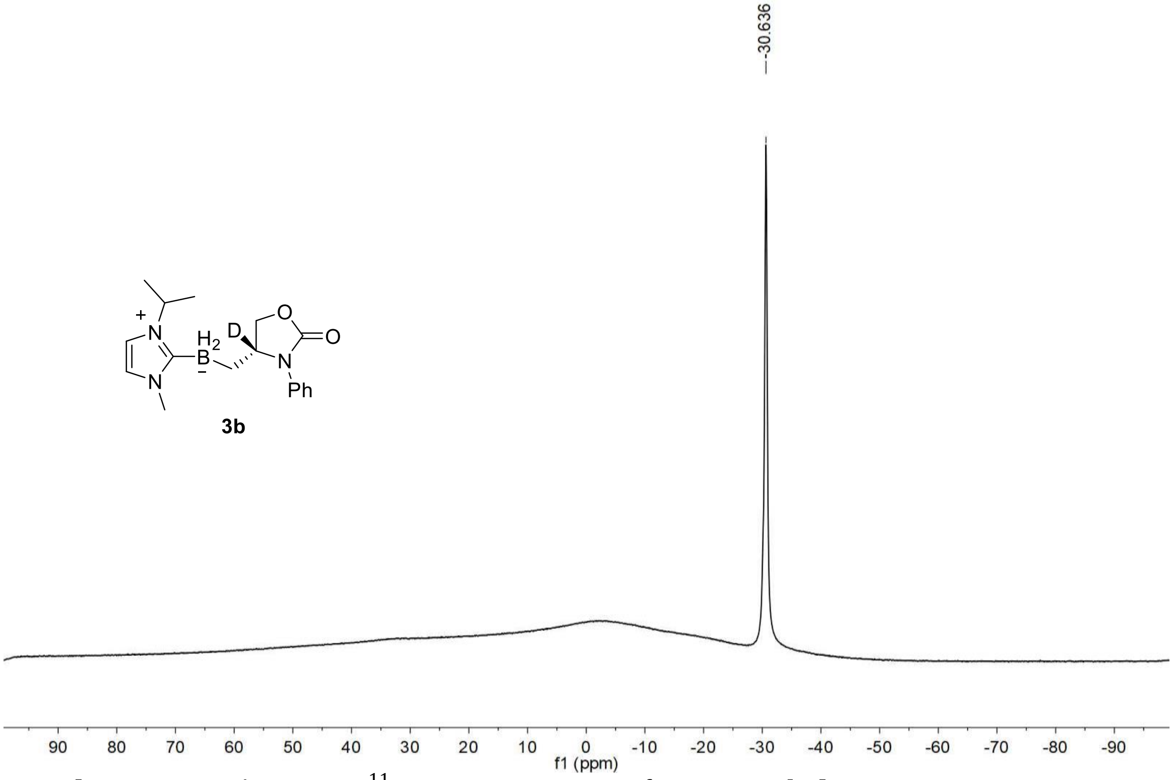

Supplementary Figure 55. ^{11}B NMR spectrum of compound **3b**

HPLC spectrum of racemic **3b**

Sample Name	:
Tray#	: 1
Vial#	: 5
Data File	: SQL-6-287-1-RAC-(3).lcd
Method File	: 4OD-H-65-0.5-214.lcm
Date Acquired	: 12/11/2021 7:13:52 PM
Date Processed	: 1/25/2022 12:40:35 AM

<Chromatogram View>

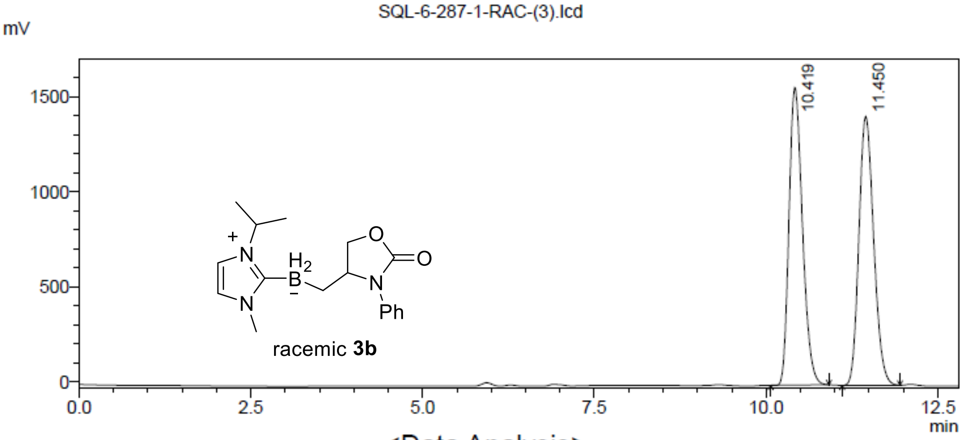

<Data Analysis>

Detector A 214nm

Pesk #	Ret. Time	Height	Area	Area%
1	10.419	1565993	21335172	50.142
2	11.450	1414750	21214392	49.858
Total		2980743	42549564	100.000

Supplementary Figure 56. HPLC spectrum of racemic **3b**

HPLC spectrum of **3b**

Tray#	: 1
Vial#	: 12
Data File	: sql-6-355-CHIRAL-(3).lcd
Method File	: 4OD-H-60-0.5-214.lcm
Date Acquired	: 1/5/2022 12:26:22 AM
Date Processed	: 1/25/2022 12:39:58 AM

<Chromatogram View>

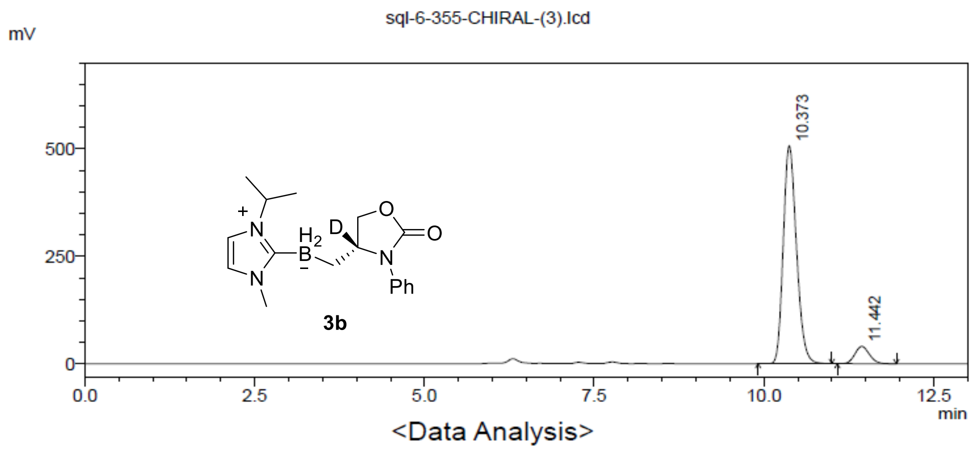

<Data Analysis>

Detector A 214nm

Pesk #	Ret. Time	Height	Area	Area%
1	10.373	507061	6857512	91.964
2	11.442	40263	599216	8.036
Total		547324	7456728	100.000

Supplementary Figure 57. HPLC spectrum of **3b**

^{1}H NMR (400 MHz, room temperature, $CDCl_3$)

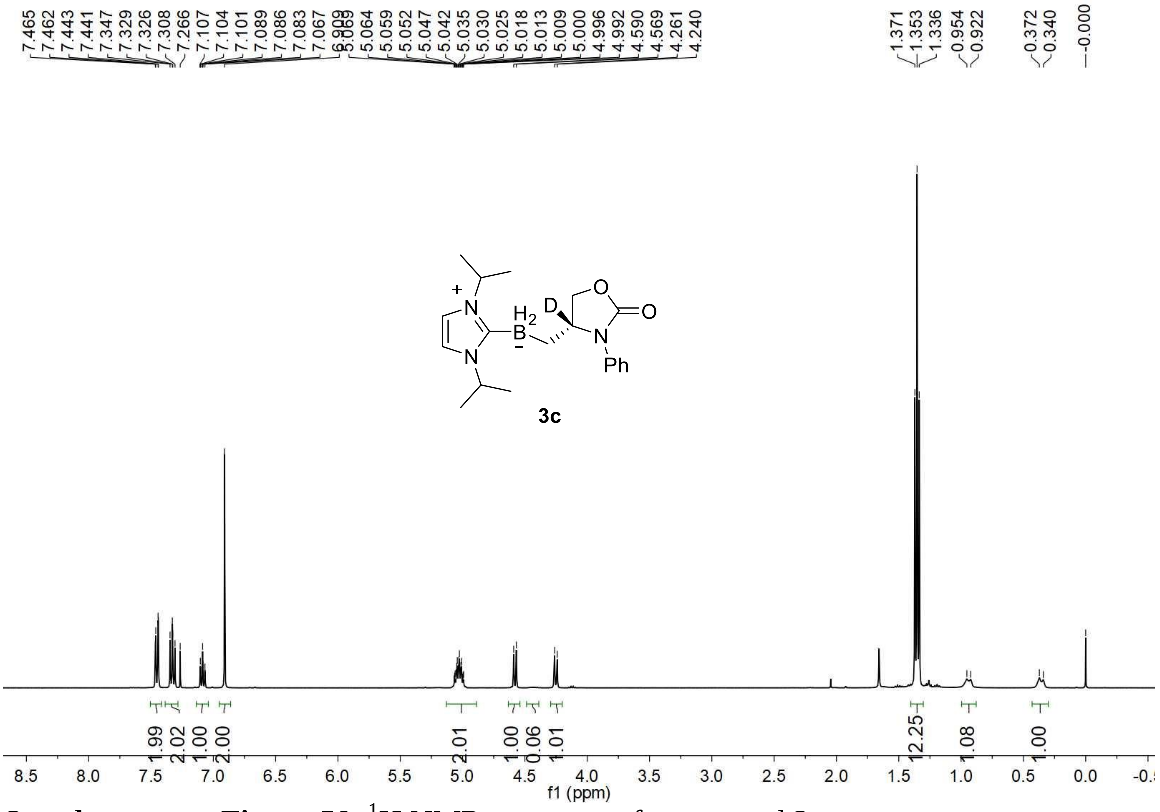

Supplementary Figure 58. ^{1}H NMR spectrum of compound **3c**

^{13}C NMR (100 MHz, room temperature, $CDCl_3$)

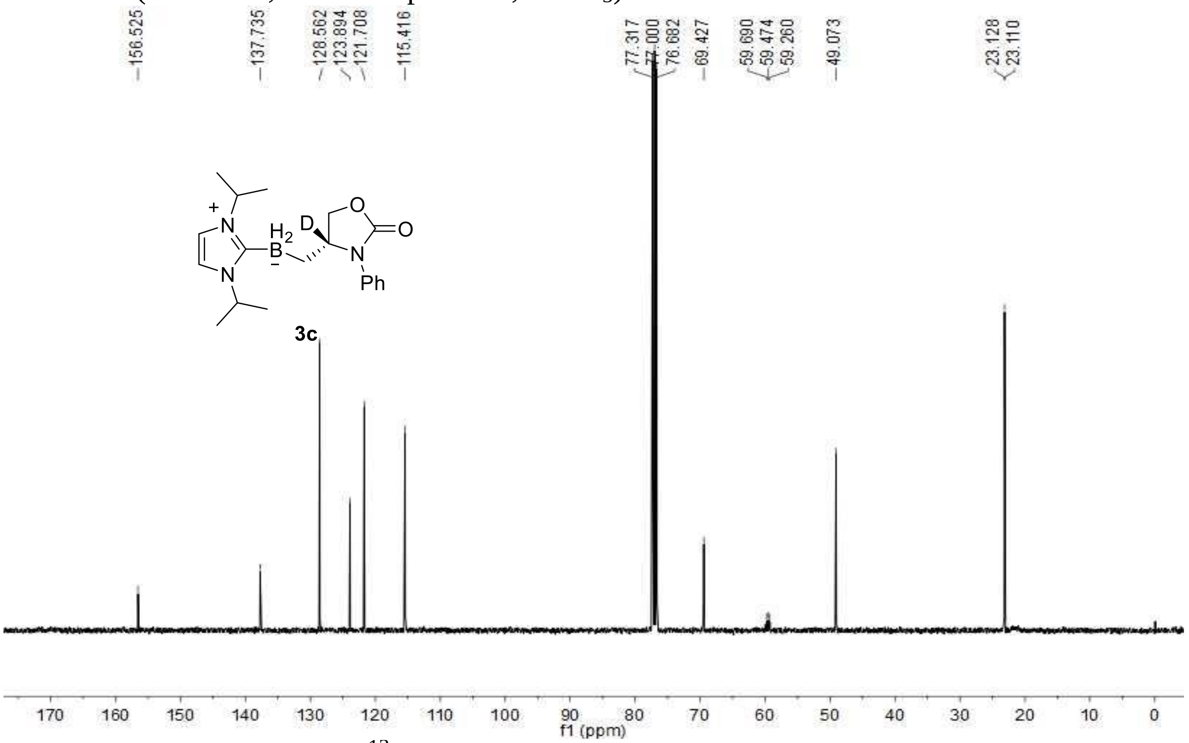

Supplementary Figure 59. ^{13}C NMR spectrum of compound **3c**

^{11}B NMR (128 MHz, room temperature, $CDCl_3$)

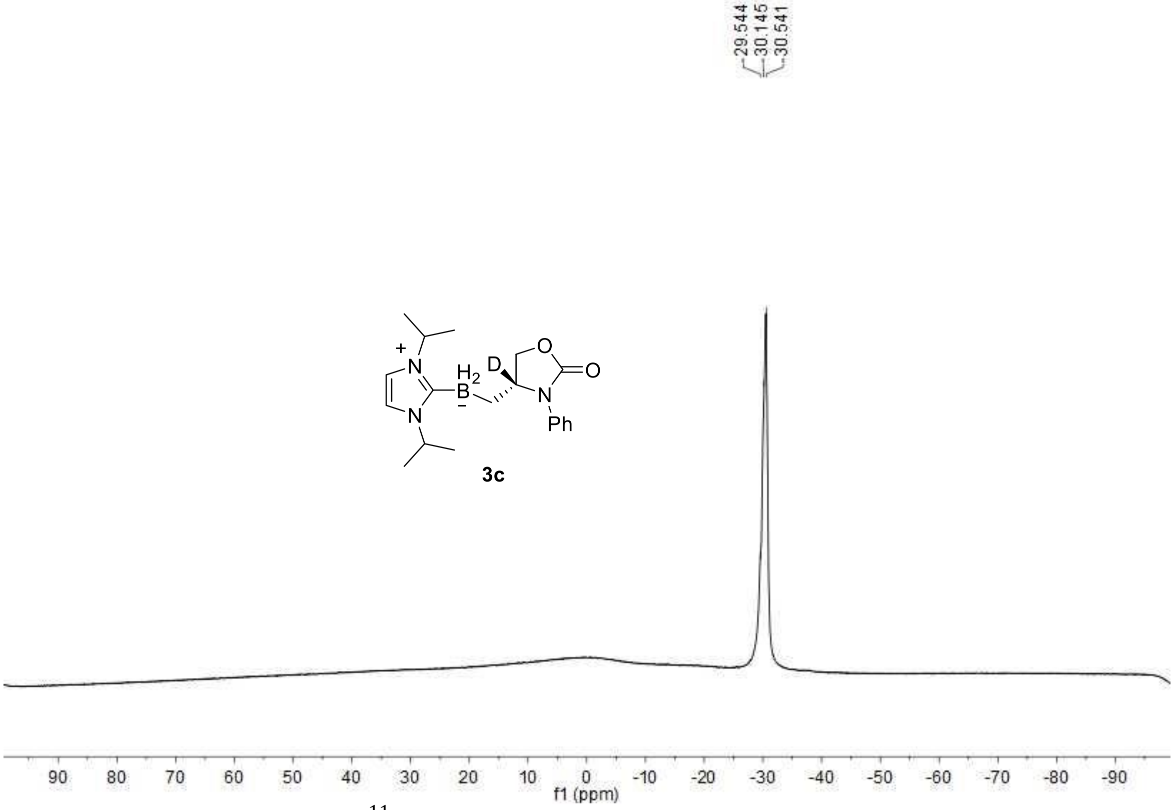

Supplementary Figure 60. ^{11}B NMR spectrum of compound **3c**

HPLC spectrum of racemic **3c**

Sample Name	:
Tray#	: 1
Vial#	: 11
Data File	: sql-6-348-1-RAC-(4).lcd
Method File	: 4OD-H-75-0.5-214.lcm
Date Acquired	: 1/3/2022 3:28:10 PM
Date Processed	: 1/25/2022 12:42:12 AM

<Chromatogram View>

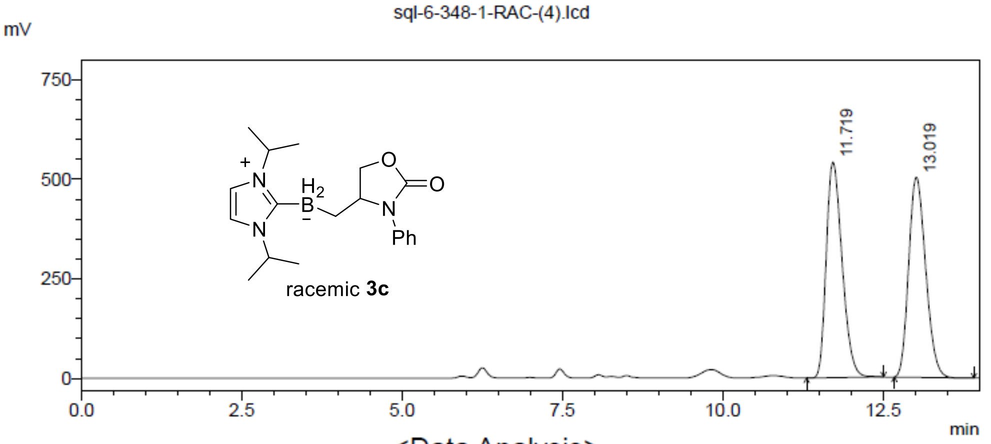

<Data Analysis>

Detector A 214nm

Pesk #	Ret. Time	Height	Area	Area%
1	11.719	541719	9337584	50.146
2	13.019	502811	9283379	49.854
Total		1044529	18620963	100.000

Supplementary Figure 61. HPLC spectrum of racemic **3c**

HPLC spectrum of **3c**

Sample Name	:
Tray#	: 1
Vial#	: 13
Data File	: sql-6-348-2-CHIRAL-(4).lcd
Method File	: 4OD-H-75-0.5-214.lcm
Date Acquired	: 1/3/2022 7:09:30 PM
Date Processed	: 1/25/2022 1:47:07 AM

<Chromatogram View>

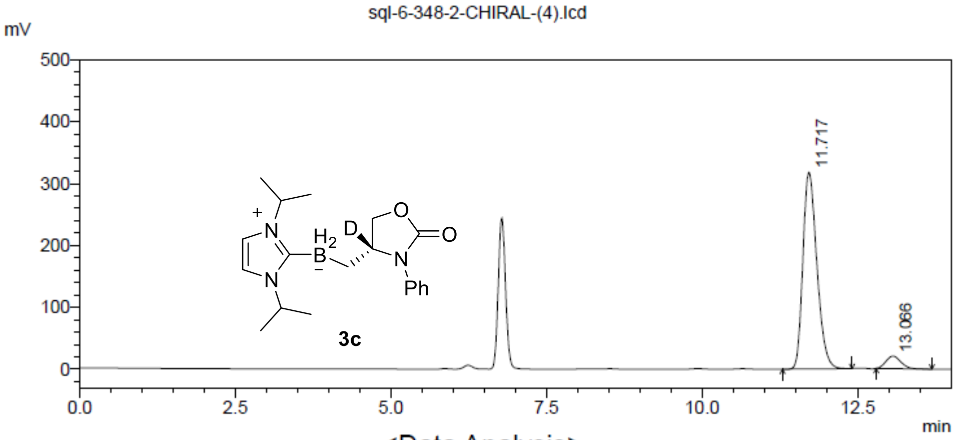

<Data Analysis>

Detector A 214nm

Pesk #	Ret. Time	Height	Area	Area%
1	11.717	317796	4961302	93.684
2	13.066	20193	334489	6.316
Total		337989	5295791	100.000

Supplementary Figure 62. HPLC spectrum of **3c**

^{1}H NMR (400 MHz, room temperature, $CDCl_3$)

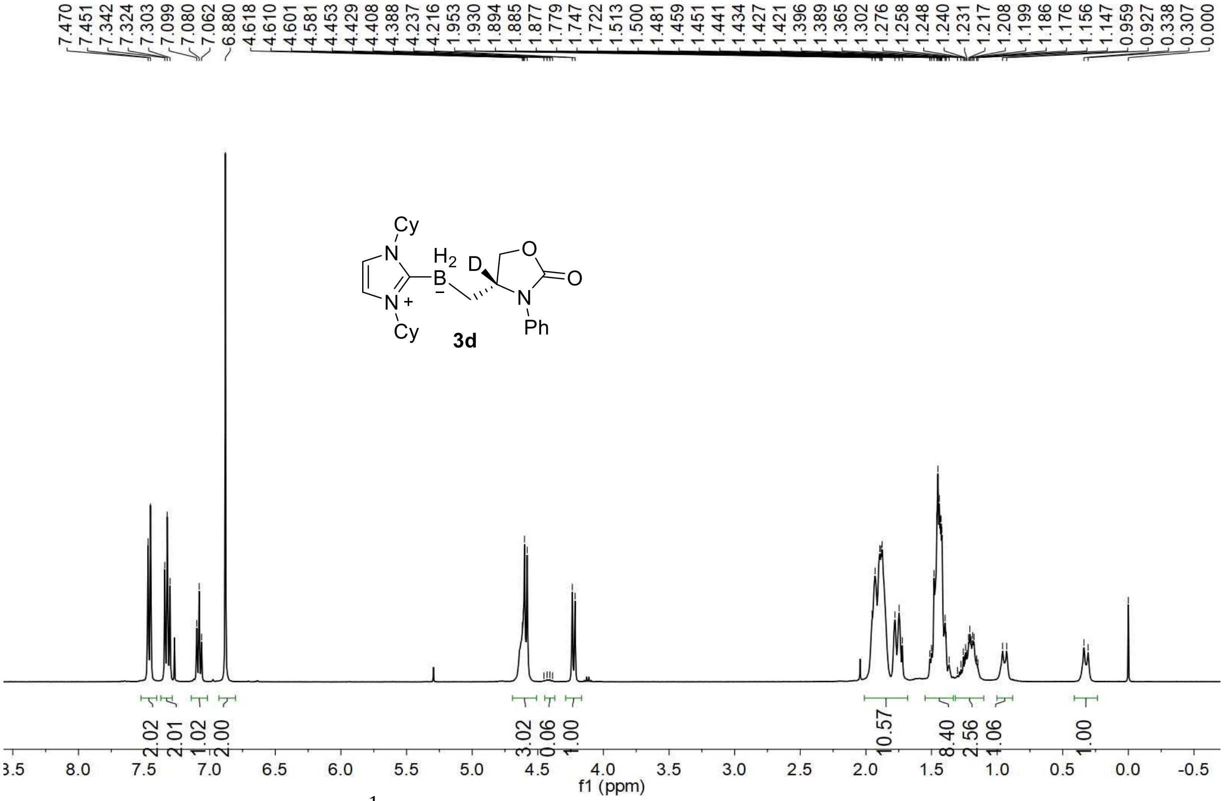

Supplementary Figure 63. ^{1}H NMR spectrum of compound **3d**

^{13}C NMR (100 MHz, room temperature, $CDCl_3$)

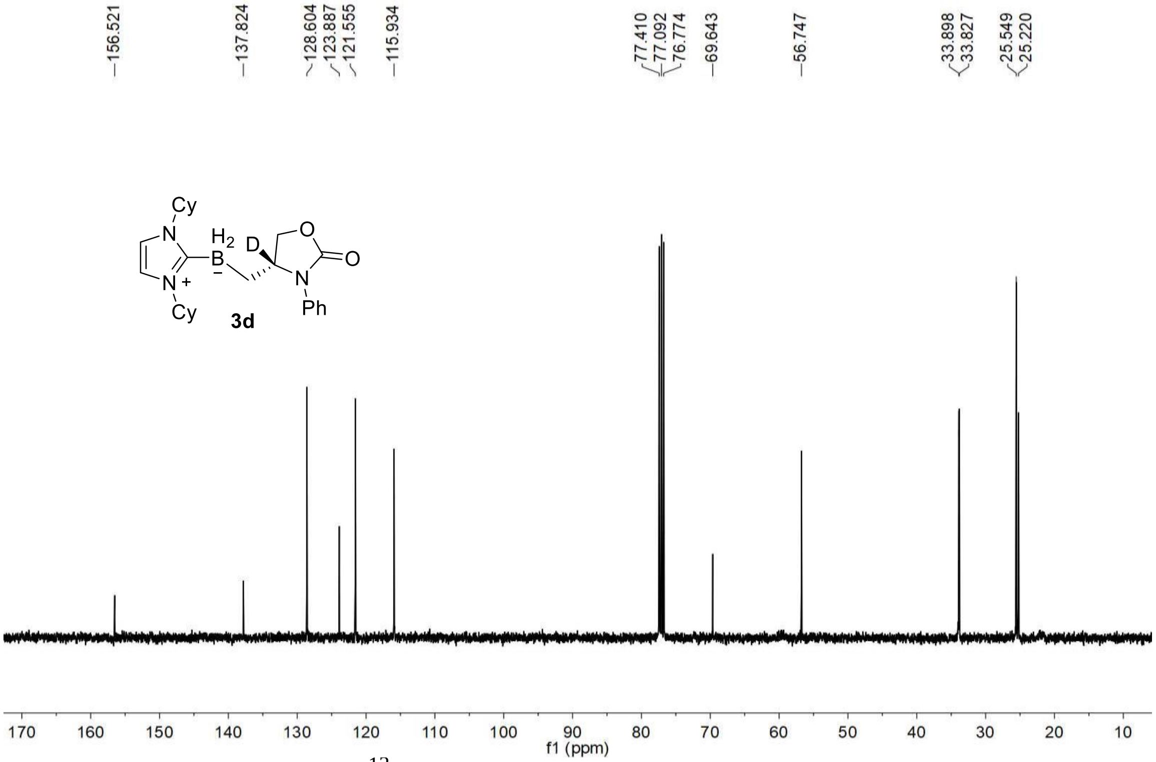

Supplementary Figure 64. ^{13}C NMR spectrum of compound **3d**

^{11}B NMR (128 MHz, room temperature, $CDCl_3$)

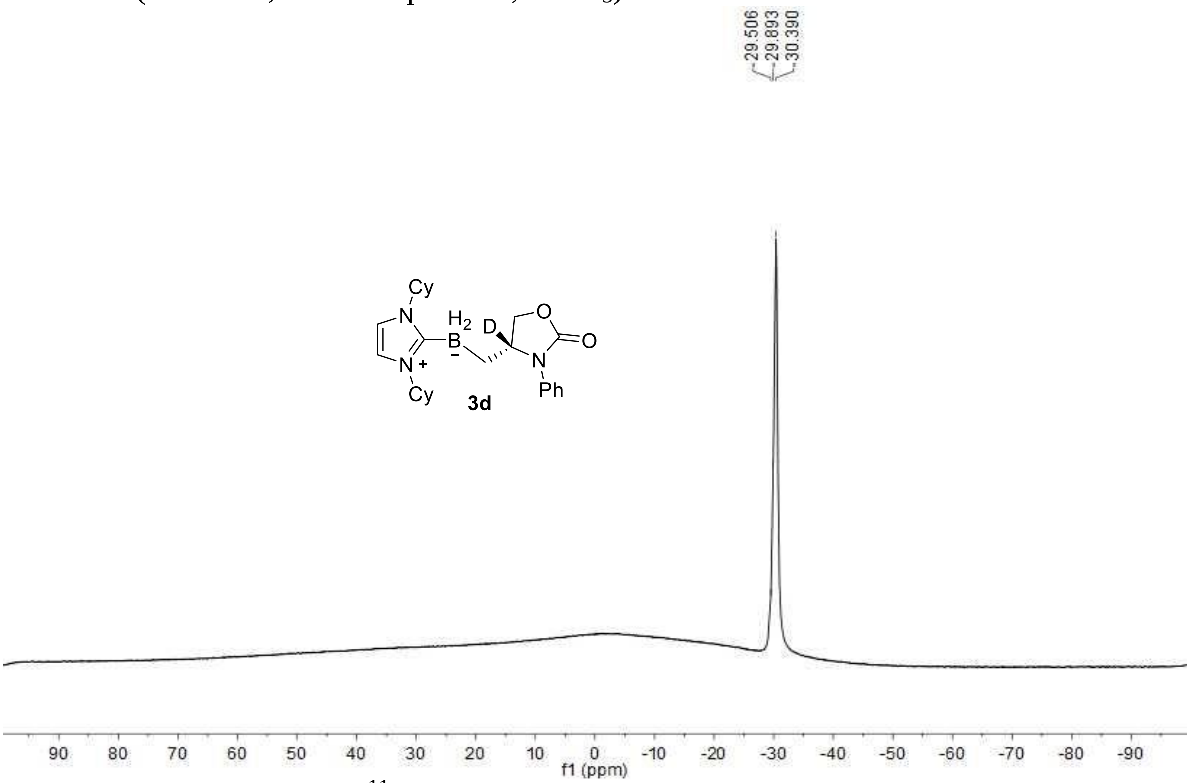

Supplementary Figure 65. ^{11}B NMR spectrum of compound **3d**

HPLC spectrum of racemic **3d**

Tray#	: 1
Vial#	: 10
Data File	: sql-7-74-1.lcd
Method File	: 4OD-H-85-0.5-214.lcm
Date Acquired	: 2/21/2022 7:56:42 PM
Date Processed	: 2/21/2022 8:27:39 PM

<Chromatogram View>

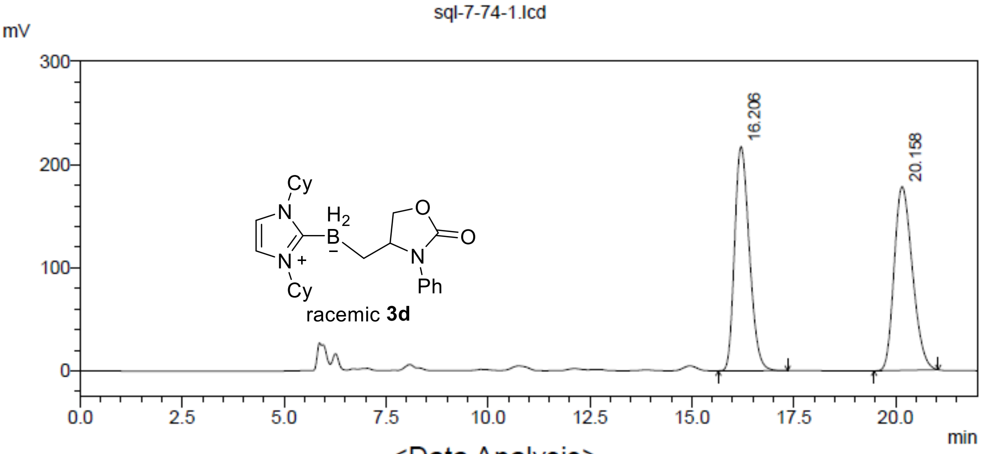

<Data Analysis>

Detector A 214nm

Pesk #	Ret. Time	Height	Area	Area%
1	16.206	217179	5470916	49.623
2	20.158	178045	5553977	50.377
Total		395224	11024893	100.000

Supplementary Figure 66. HPLC spectrum of racemic **3d**

HPLC spectrum of **3d**

Sample Name :
Tray# : 1
Vial# : 11
Data File : sql-7-74-2.lcd
Method File : 4OD-H-85-0.5-214.lcm
Date Acquired : 2/21/2022 7:32:04 PM
Date Processed : 2/21/2022 7:54:57 PM

<Chromatogram View>

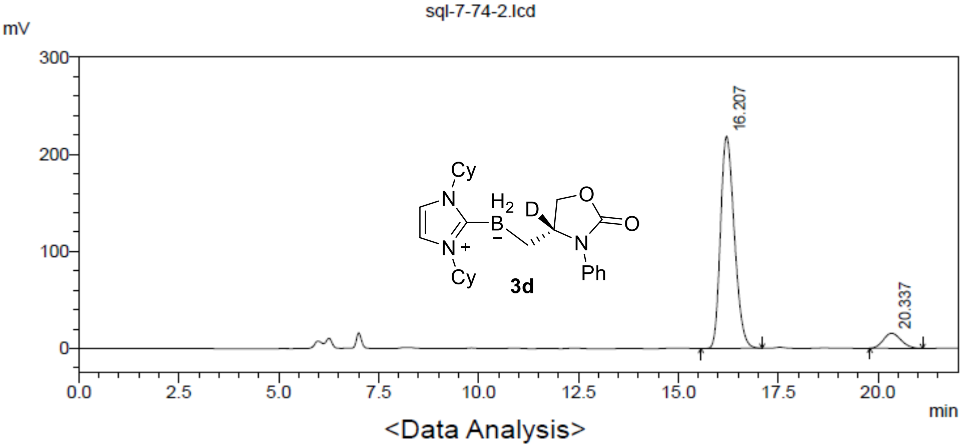

<Data Analysis>

Detector A 214nm

Pesk #	Ret. Time	Height	Area	Area%
1	16.207	218366	5275761	91.692
2	20.337	15537	478046	8.308
Total		233903	5753807	100.000

Supplementary Figure 67. HPLC spectrum of **3d**

^{1}H NMR (400 MHz, room temperature, $CDCl_3$)

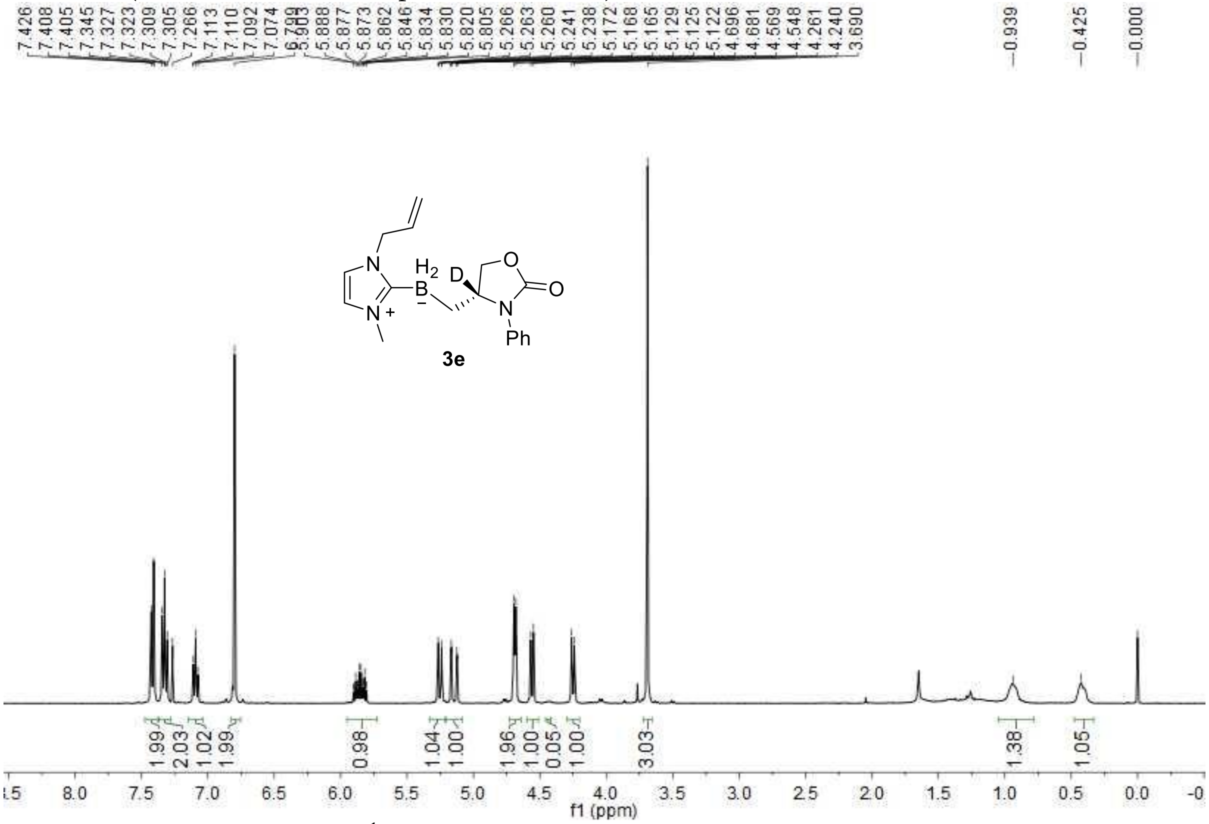

Supplementary Figure 68. ^{1}H NMR spectrum of compound **3e**

^{13}C NMR (100 MHz, room temperature, $CDCl_3$)

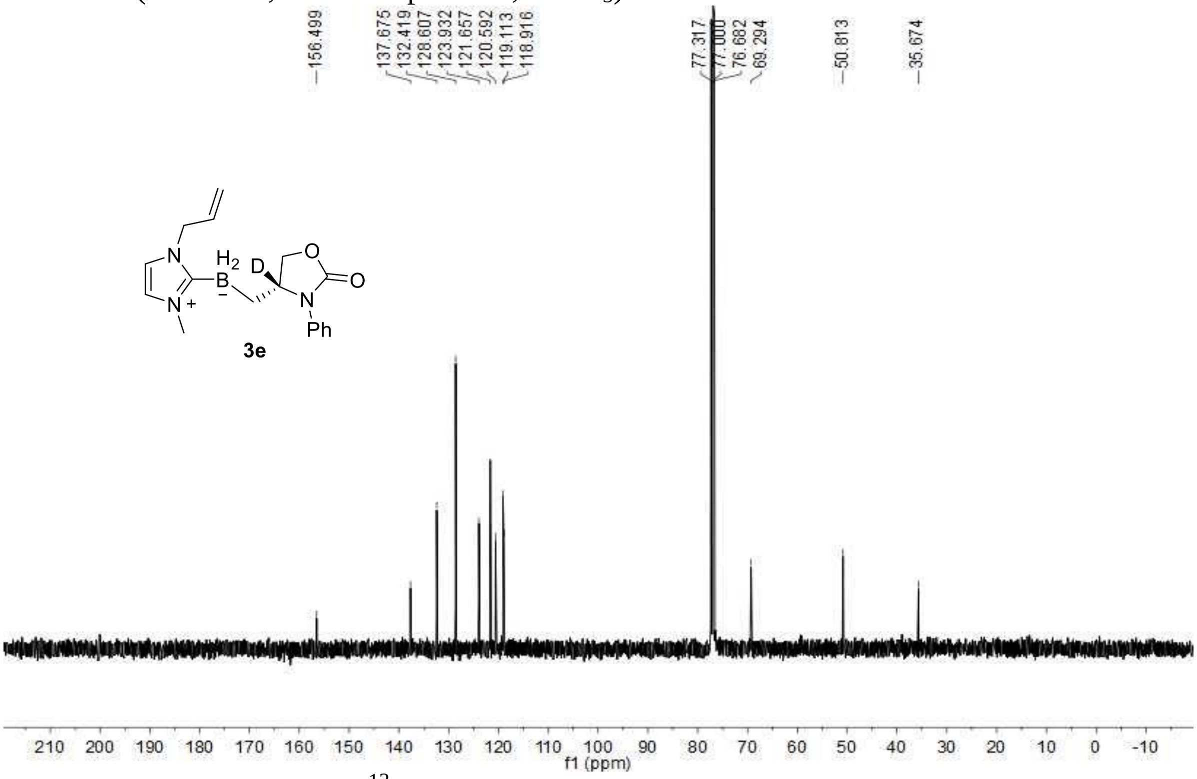

Supplementary Figure 69. ^{13}C NMR spectrum of compound **3e**

^{11}B NMR (128 MHz, room temperature, $CDCl_3$)

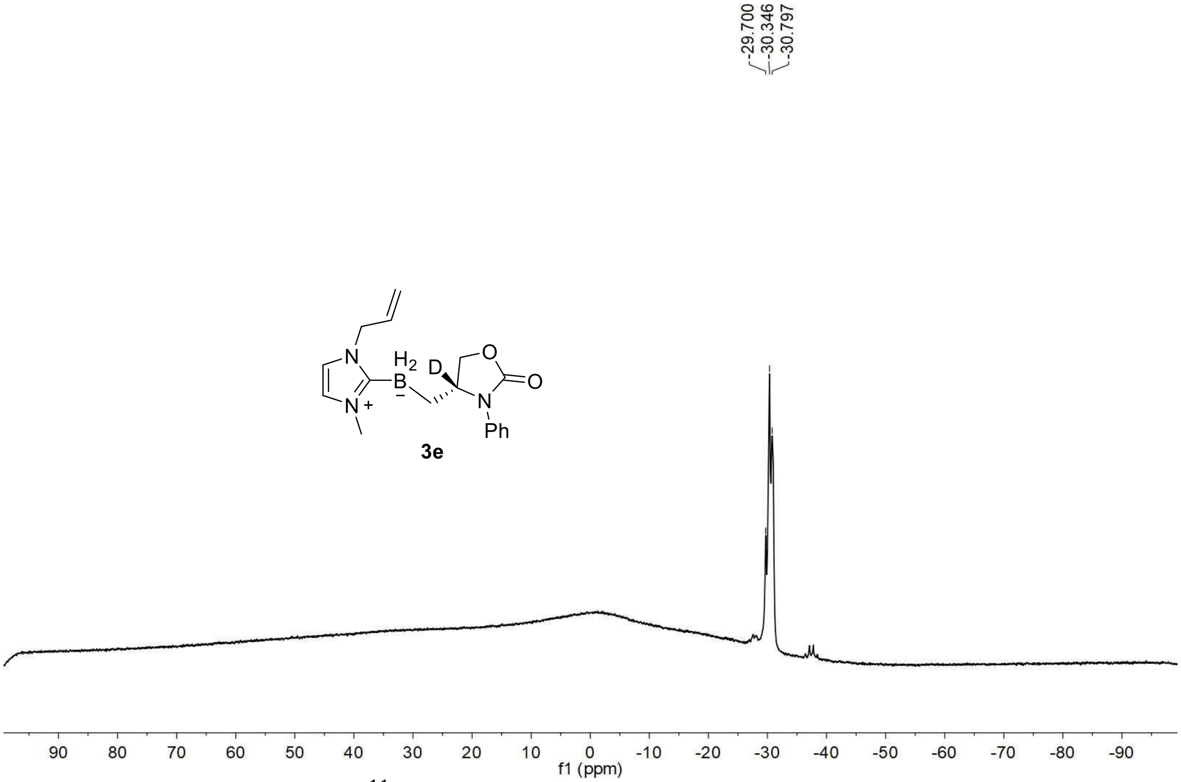

Supplementary Figure 70. ^{11}B NMR spectrum of compound **3e**

HPLC spectrum of racemic **3e**

Sample Name	:
Tray#	: 1
Vial#	: 12
Data File	: sql-7-73-1-1.lcd
Method File	: 4OD-H-80-0.5-214.lcm
Date Acquired	: 2/22/2022 2:17:55 PM
Date Processed	: 2/22/2022 4:32:32 PM

<Chromatogram View>

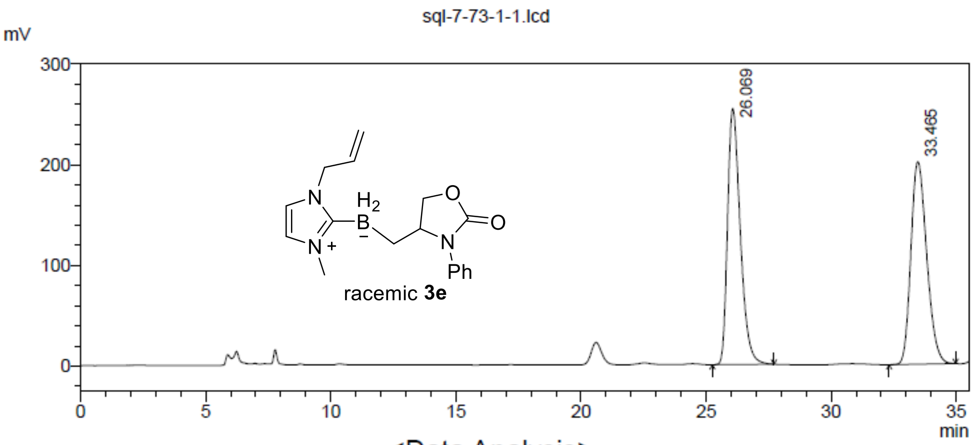

<Data Analysis>

Detector A 214nm

Pesk #	Ret. Time	Height	Area	Area%
1	26.069	254307	8974856	50.360
2	33.465	201219	8846546	49.640
Total		455526	17821402	100.000

Supplementary Figure 71. HPLC spectrum of racemic **3e**

HPLC spectrum of **3e**

Sample Name	:
Tray#	: 1
Vial#	: 20
Data File	: sql-7-73-2-2.lcd
Method File	: 4OD-H-80-0.5-214.lcm
Date Acquired	: 2/21/2022 11:47:26 PM
Date Processed	: 2/22/2022 9:11:31 AM

<Chromatogram View>

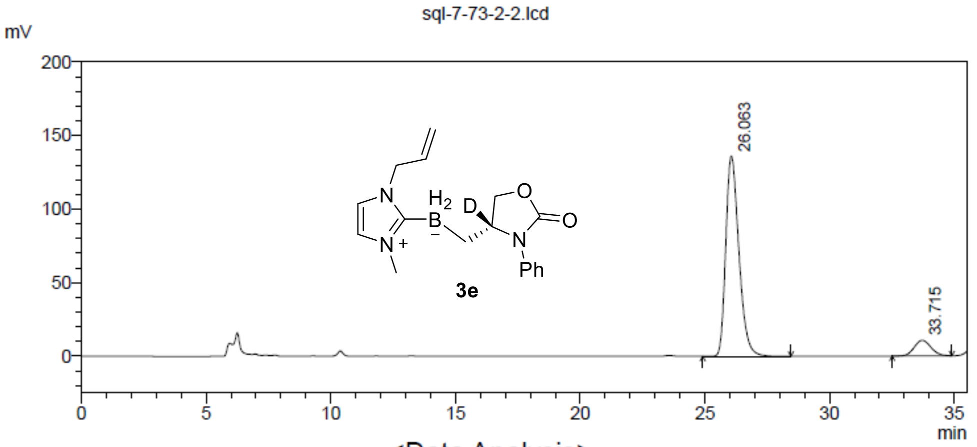

<Data Analysis>

Detector A 214nm

Pesk #	Ret. Time	Height	Area	Area%
1	26.063	136222	4985968	91.048
2	33.715	10594	490244	8.952
Total		146816	5476212	100.000

Supplementary Figure 72. HPLC spectrum of **3e**

^{1}H NMR (400 MHz, room temperature, $CDCl_3$)

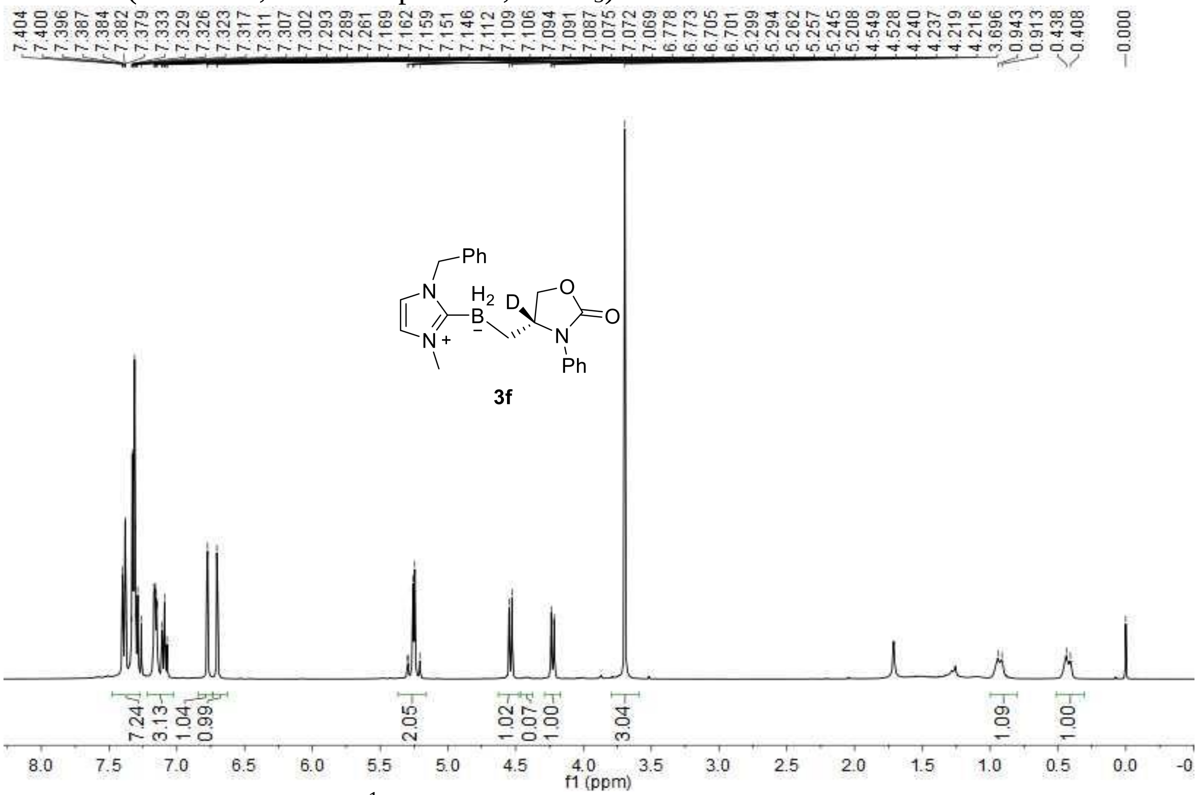

Supplementary Figure 73. ^{1}H NMR spectrum of compound **3f**

^{13}C NMR (100 MHz, room temperature, $CDCl_3$)

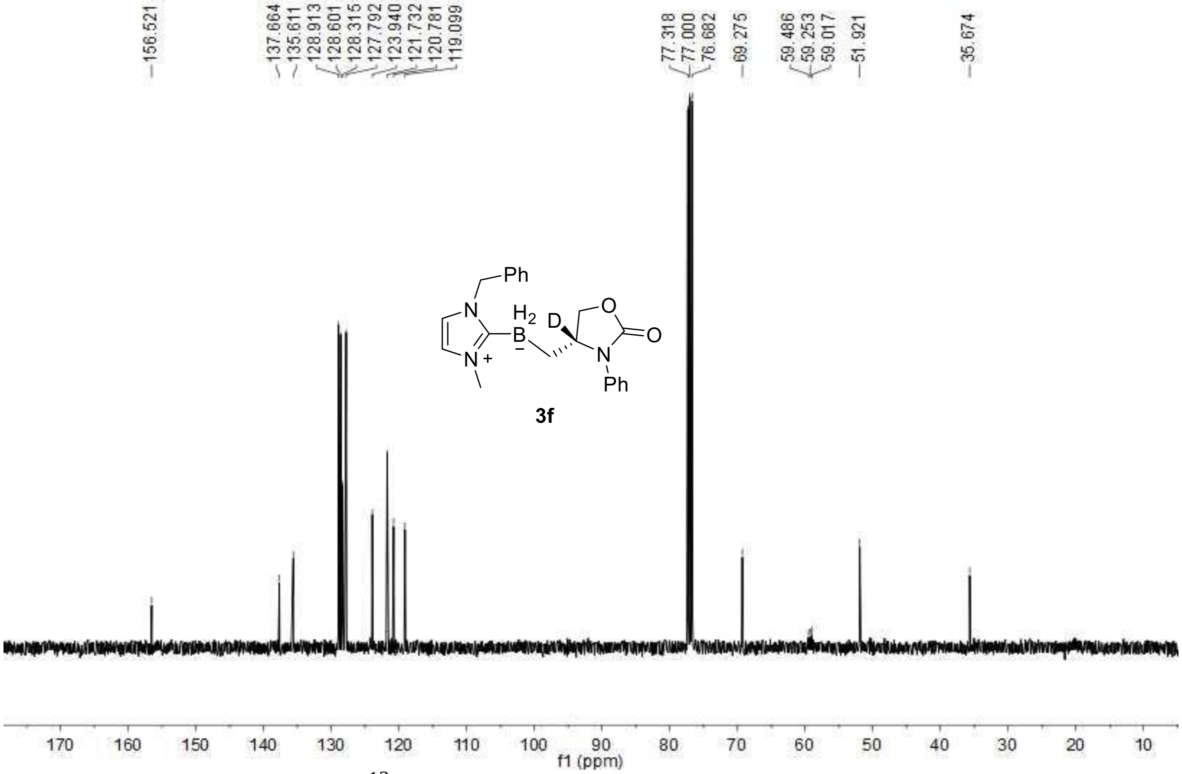

Supplementary Figure 74. ^{13}C NMR spectrum of compound **3f**

^{11}B NMR (128 MHz, room temperature, $CDCl_3$)

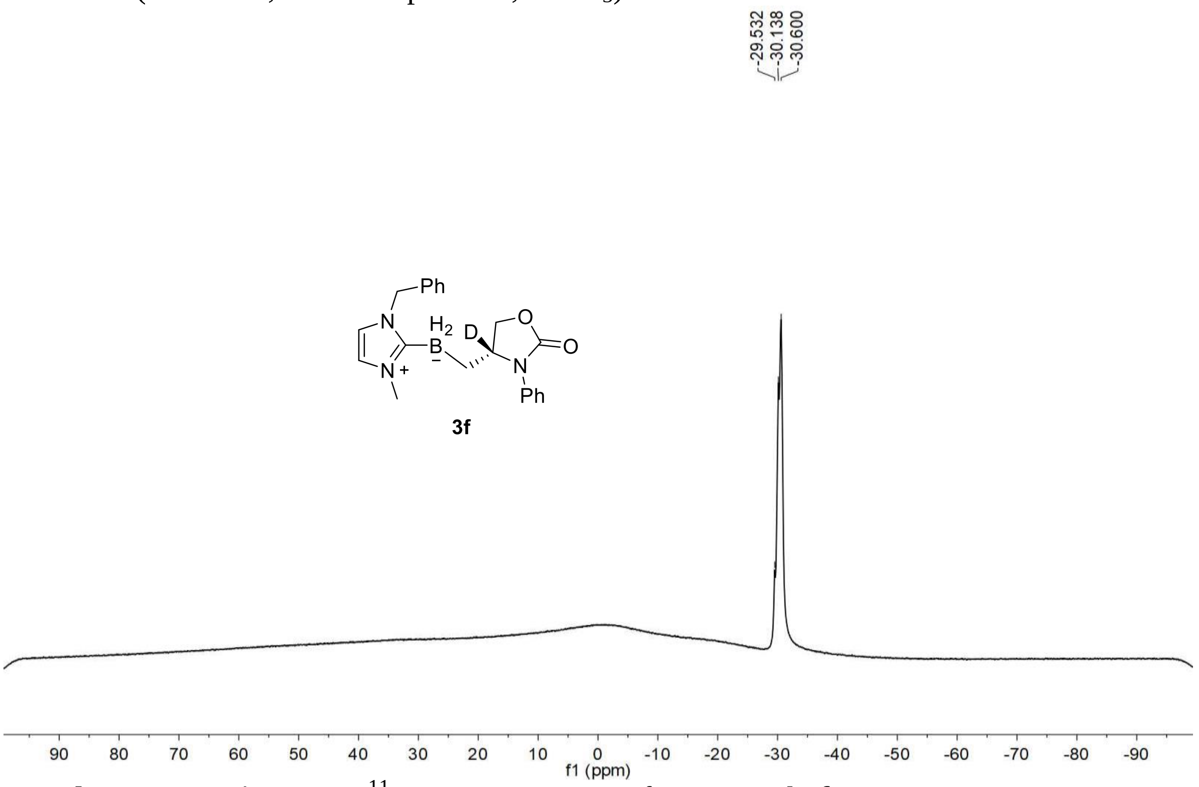

Supplementary Figure 75. ^{11}B NMR spectrum of compound **3f**

HPLC spectrum of racemic **3f**

Tray#	: 1
Vial#	: 11
Data File	: SQL-7-23-1-1-(6).lcd
Method File	: 4OD-H-60-1.0-214.lcm
Date Acquired	: 1/23/2022 8:28:50 AM
Date Processed	: 1/25/2022 12:47:09 AM

<Chromatogram View>

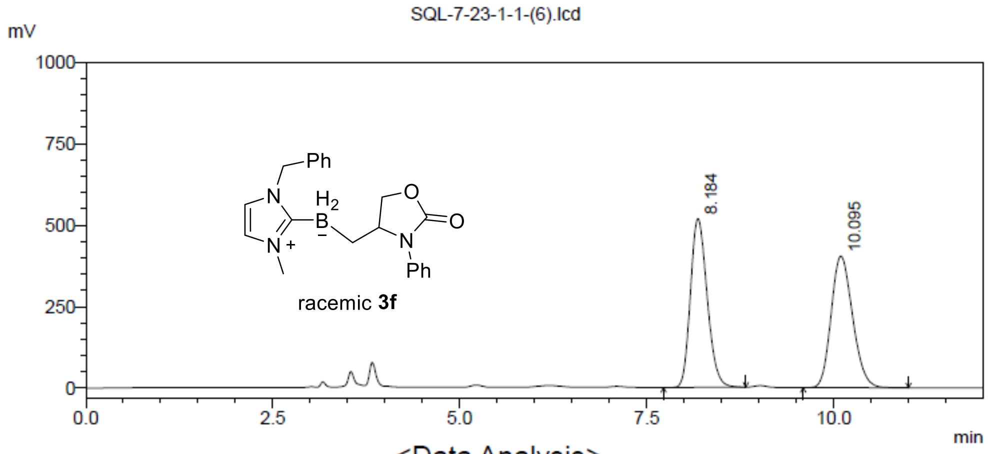

<Data Analysis>

Detector A 214nm

Pesk #	Ret. Time	Height	Area	Area%
1	8.184	518456	8079575	50.042
2	10.095	404174	8066099	49.958
Total		922630	16145675	100.000

Supplementary Figure 76. HPLC spectrum of racemic **3f**

HPLC spectrum of **3f**

Sample Name	:
Tray#	: 1
Vial#	: 12
Data File	: SQL-7-23-2-1-(6).lcd
Method File	: 4OD-H-60-1.0-214.lcm
Date Acquired	: 1/23/2022 8:44:46 AM
Date Processed	: 1/25/2022 12:46:57 AM

<Chromatogram View>

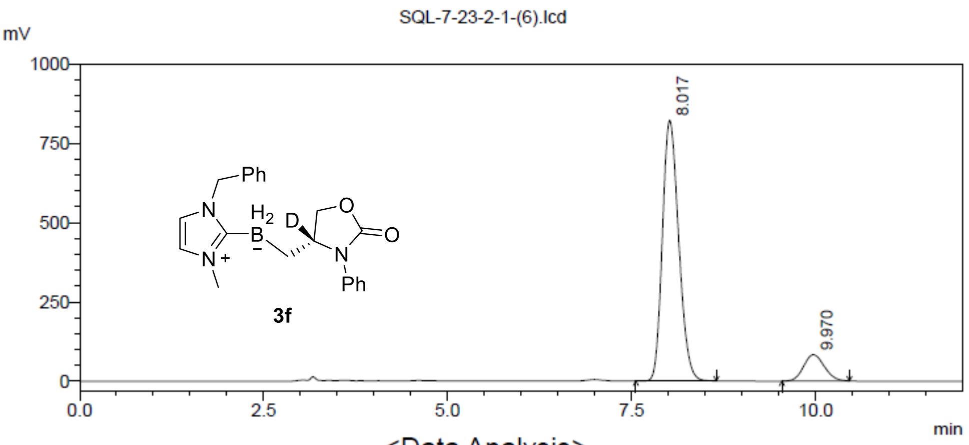

<Data Analysis>

Detector A 214nm

Pesk #	Ret. Time	Height	Area	Area%
1	8.017	822021	12674836	88.647
2	9.970	82970	1623241	11.353
Total		904991	14298076	100.000

Supplementary Figure 77. HPLC spectrum of **3f**

^{1}H NMR (400 MHz, room temperature, $CDCl_3$)

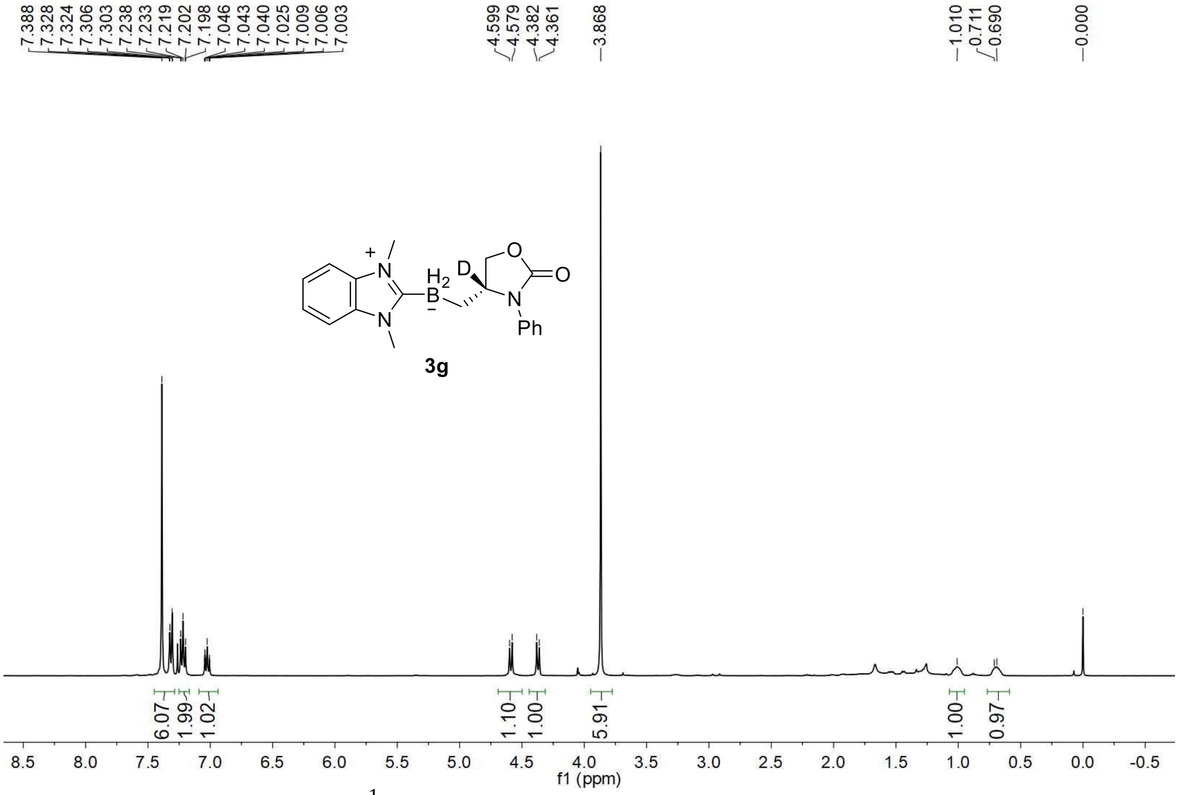

Supplementary Figure 78. ^{1}H NMR spectrum of compound **3g**

^{13}C NMR (100 MHz, room temperature, $CDCl_3$)

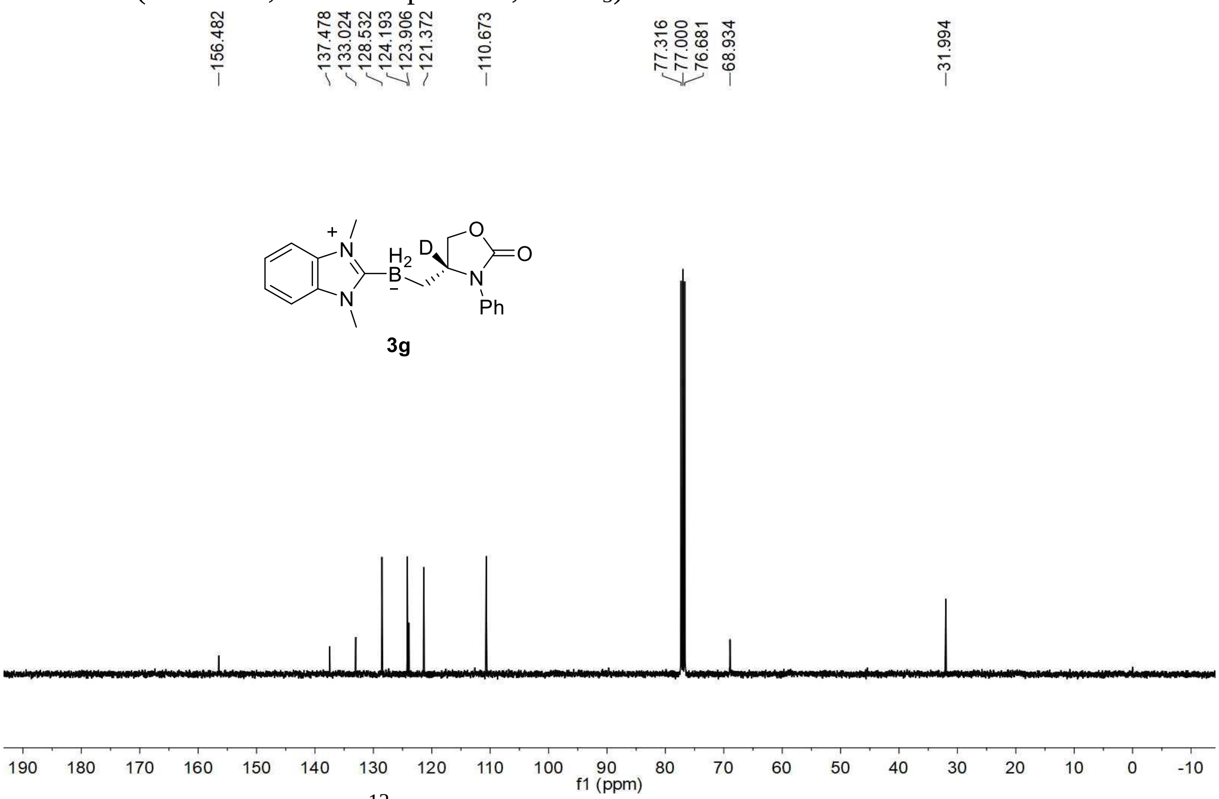

Supplementary Figure 79. ^{13}C NMR spectrum of compound **3g**

^{11}B NMR (128 MHz, room temperature, $CDCl_3$)

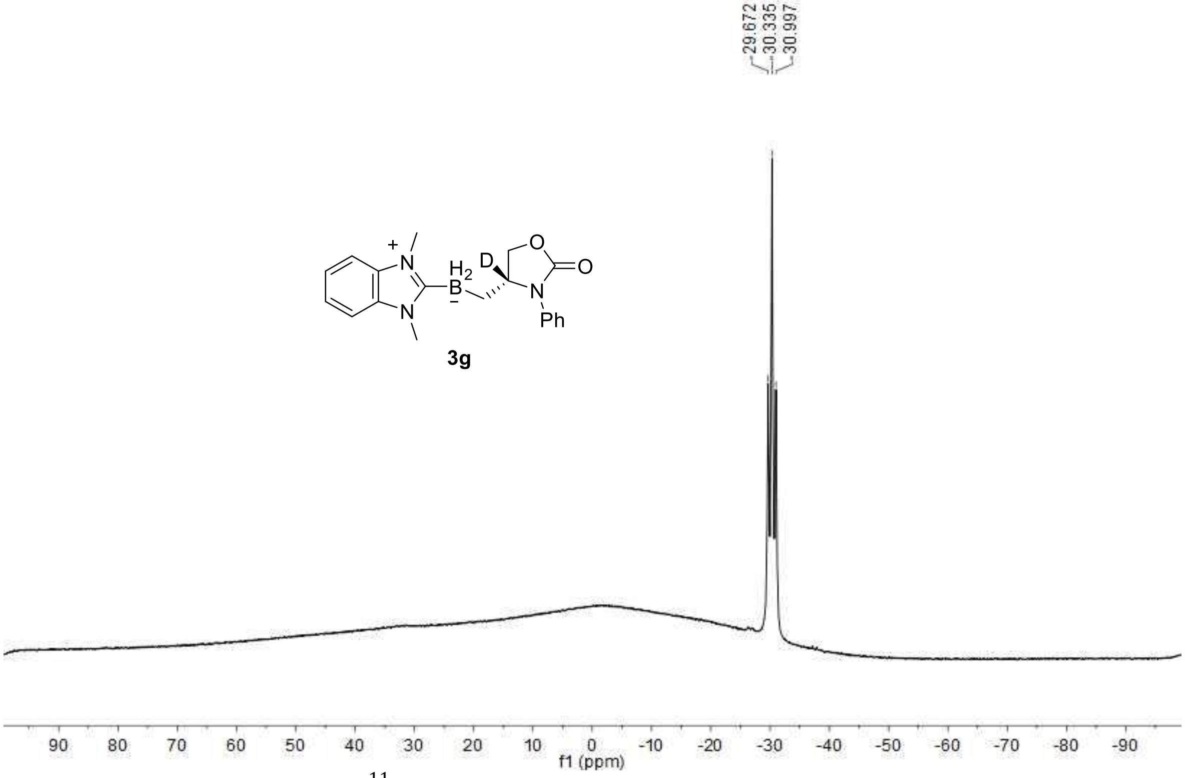

Supplementary Figure 80. ^{11}B NMR spectrum of compound **3g**

HPLC spectrum of racemic **3g**

Tray#	: 1
Vial#	: 11
Data File	: sql-6-360-1-4-CHIRAL-(5).lcd
Method File	: 4OD-H-70-0.5-214.lcm
Date Acquired	: 1/7/2022 4:50:51 PM
Date Processed	: 1/25/2022 12:43:05 AM

<Chromatogram View>

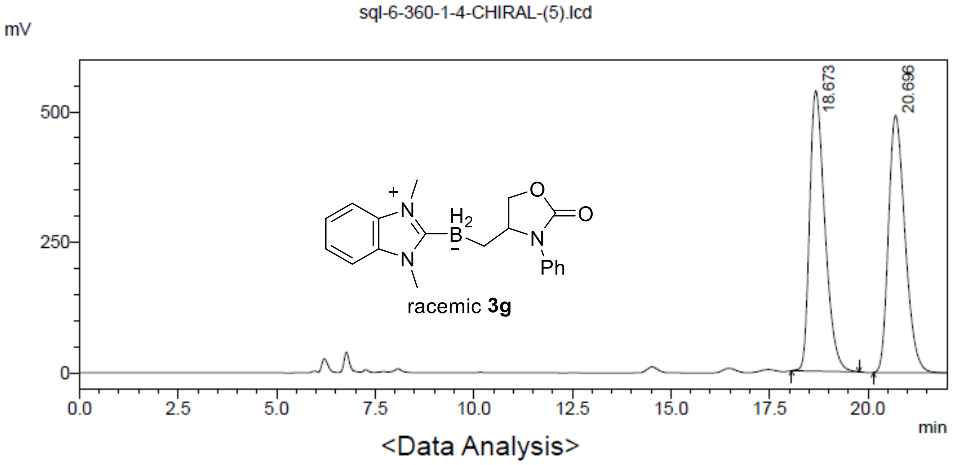

<Data Analysis>

Detector A 214nm

Pesk #	Ret. Time	Height	Area	Area%
1	18.673	537145	14398686	49.984
2	20.696	492652	14407750	50.016
Total		1029797	28806437	100.000

Supplementary Figure 81. HPLC spectrum of racemic **3g**

HPLC spectrum of **3g**

Sample Name	:
Tray#	: 1
Vial#	: 10
Data File	: sql-6-360-2-1-CHIRAL-(5).lcd
Method File	: 4OD-H-70-0.5-214.lcm
Date Acquired	: 1/7/2022 5:16:57 PM
Date Processed	: 1/25/2022 12:43:42 AM

<Chromatogram View>

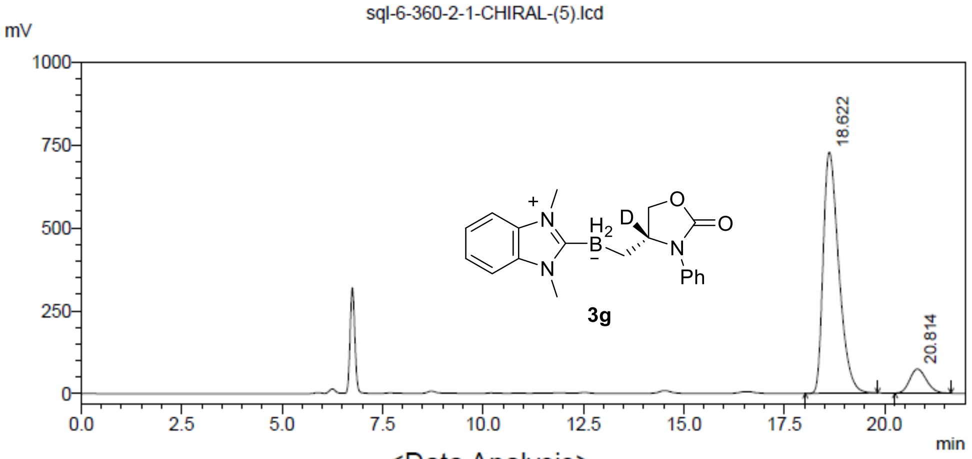

<Data Analysis>

Detector A 214nm

Pesk #	Ret. Time	Height	Area	Area%
1	18.622	727839	19663686	90.293
2	20.814	73038	2114051	9.707
Total		800877	21777737	100.000

Supplementary Figure 82. HPLC spectrum of **3g**

^{1}H NMR (400 MHz, room temperature, $CDCl_3$)

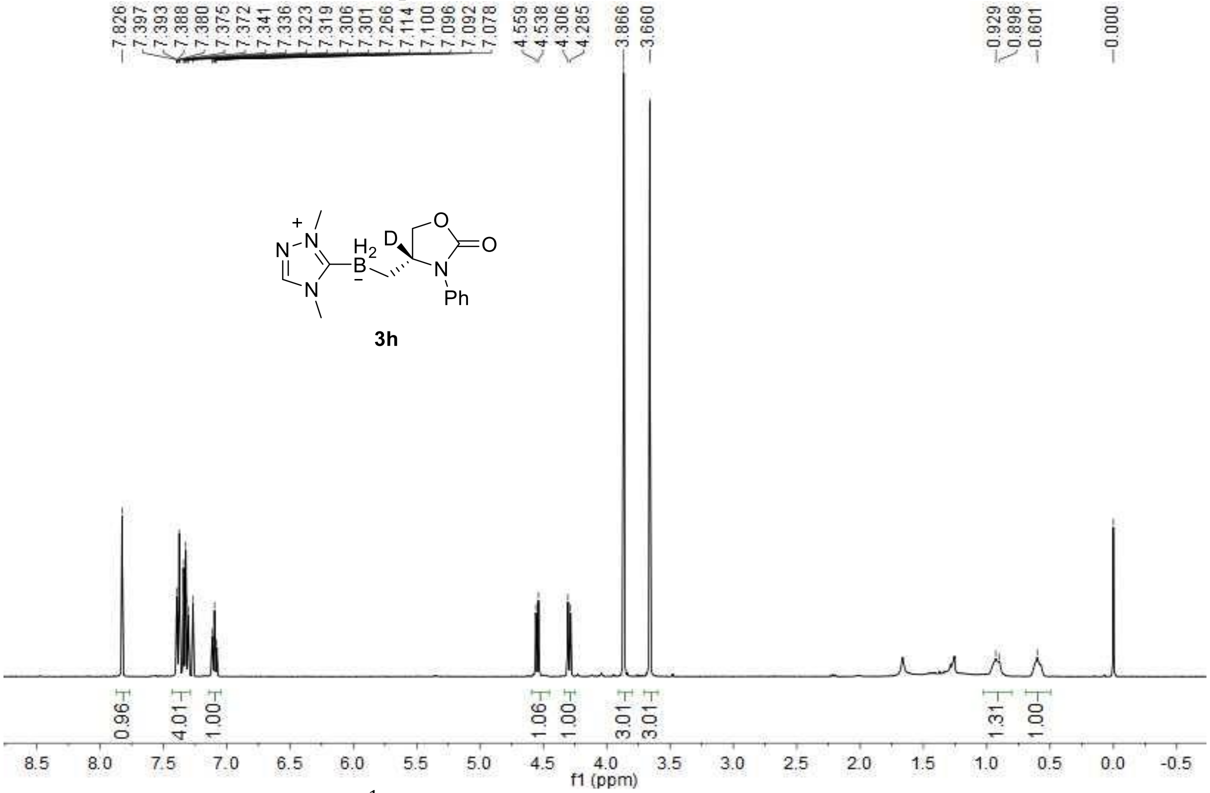

Supplementary Figure 83. ^{1}H NMR spectrum of compound **3h**

^{13}C NMR (100 MHz, room temperature, $CDCl_3$)

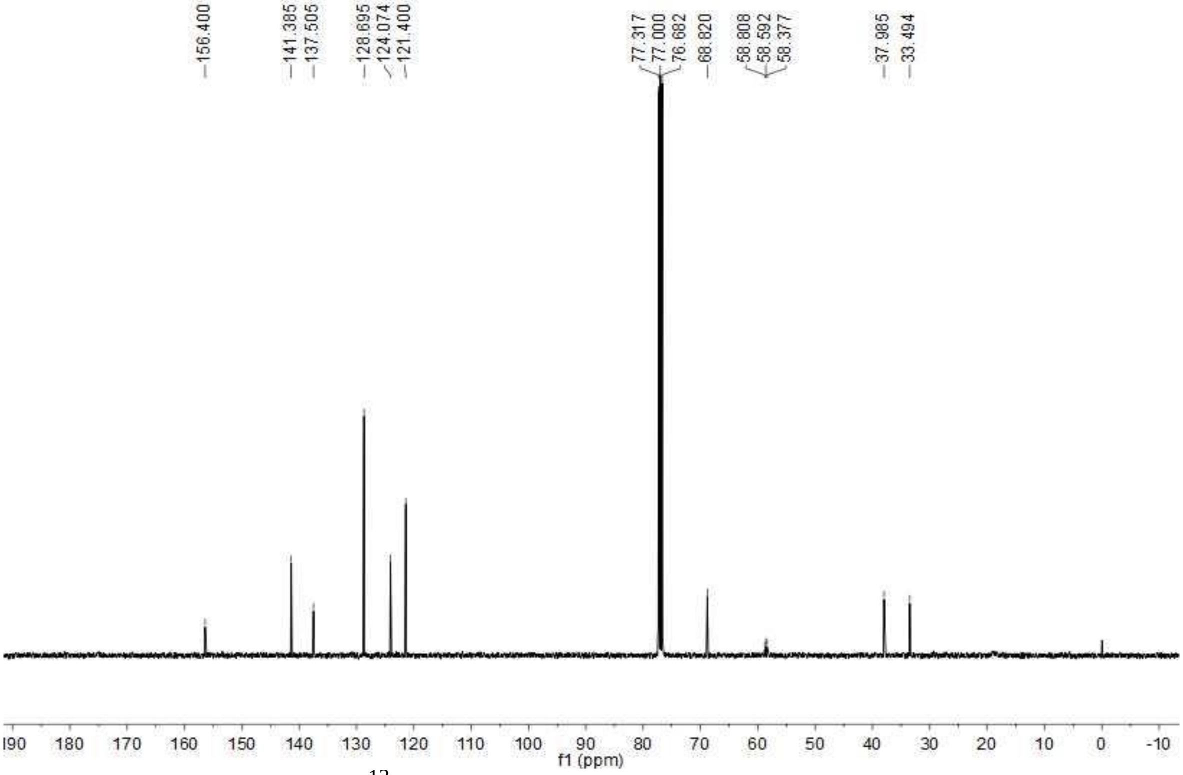

Supplementary Figure 84. ^{13}C NMR spectrum of compound **3h**

^{11}B NMR (128 MHz, room temperature, $CDCl_3$)

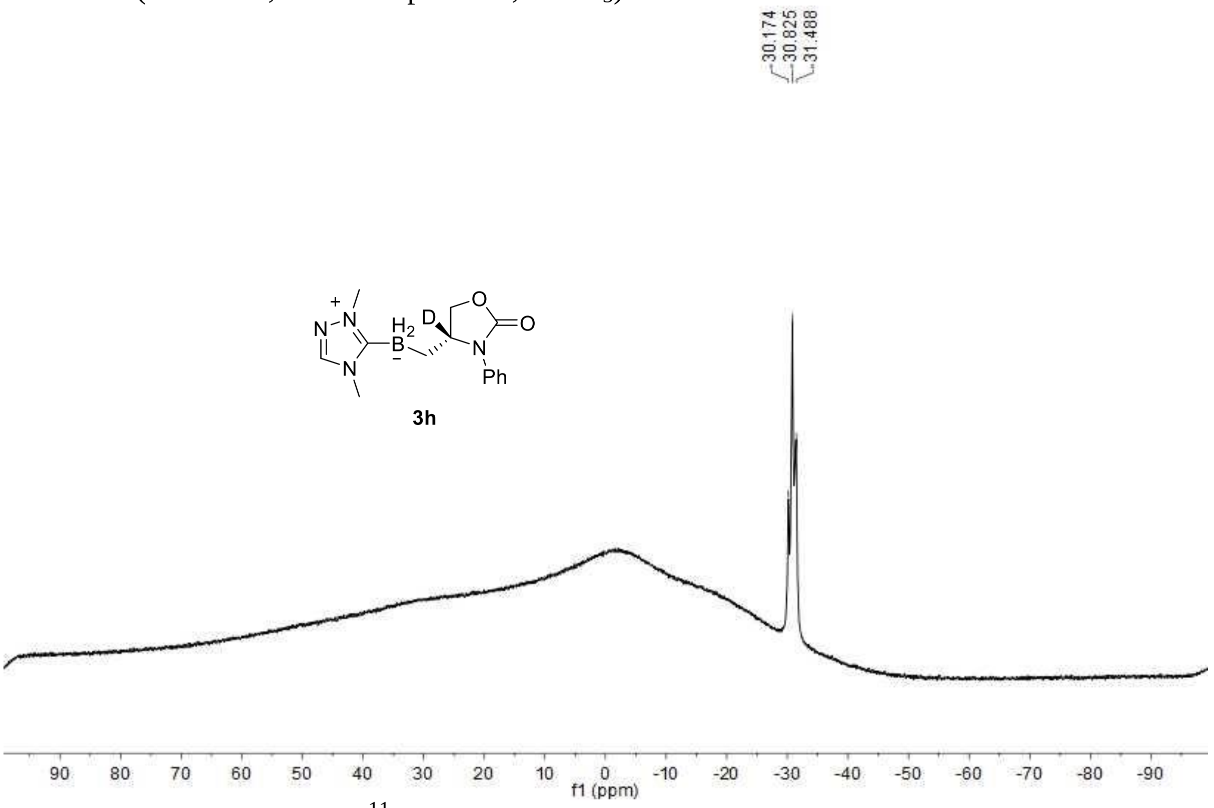

Supplementary Figure 85. ^{11}B NMR spectrum of compound **3h**

HPLC spectrum of racemic **3h**

Tray#	: 1
Vial#	: 3
Data File	: sql-6-353-1-4-(3N)-(2).lcd
Method File	: 4OD-H-60-0.5-214.lcm
Date Acquired	: 1/4/2022 10:02:25 PM
Date Processed	: 1/25/2022 12:37:13 AM

<Chromatogram View>

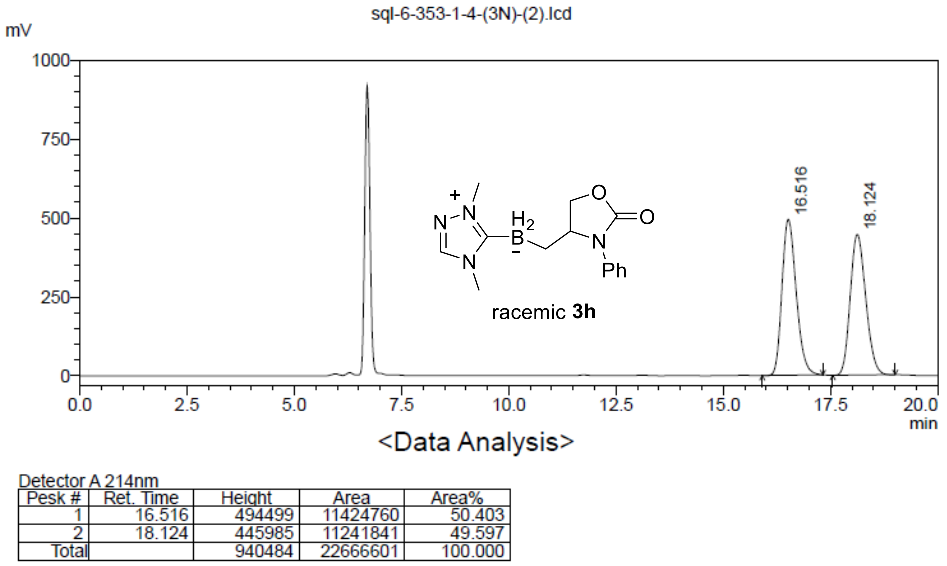

<Data Analysis>

Detector A 214nm

Pesk #	Ret. Time	Height	Area	Area%
1	16.516	494499	11424760	50.403
2	18.124	445985	11241841	49.597
Total		940484	22666601	100.000

Supplementary Figure 86. HPLC spectrum of racemic **3h**

HPLC spectrum of **3h**

Vial#	: 10
Data File	: sql-6-353-2-(3N)-CHIRAL-(2).lcd
Method File	: 4OD-H-60-0.5-214.lcm
Date Acquired	: 1/4/2022 11:34:10 PM
Date Processed	: 1/25/2022 12:37:49 AM

<Chromatogram View>

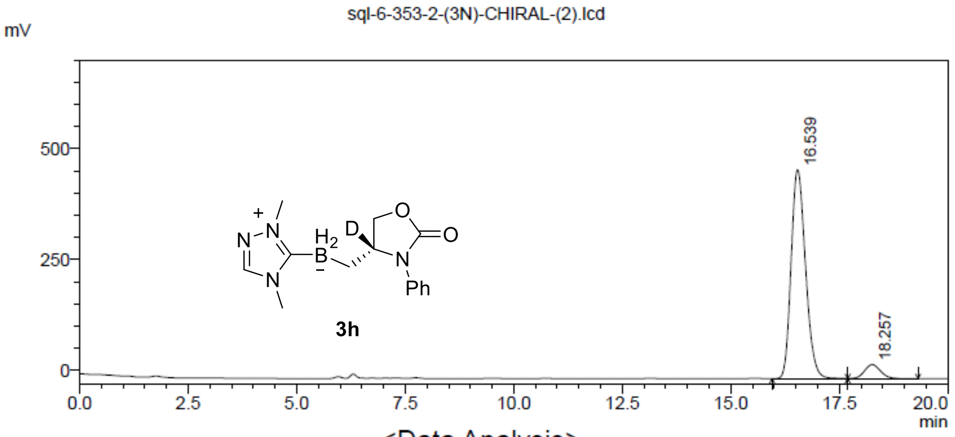

<Data Analysis>

Detector A 214nm

Pesk #	Ret. Time	Height	Area	Area%
1	16.539	471492	10889523	93.002
2	18.257	31608	819411	6.998
Total		503100	11708934	100.000

Supplementary Figure 87. HPLC spectrum of **3h**

^{1}H NMR (400 MHz, room temperature, $CDCl_3$)

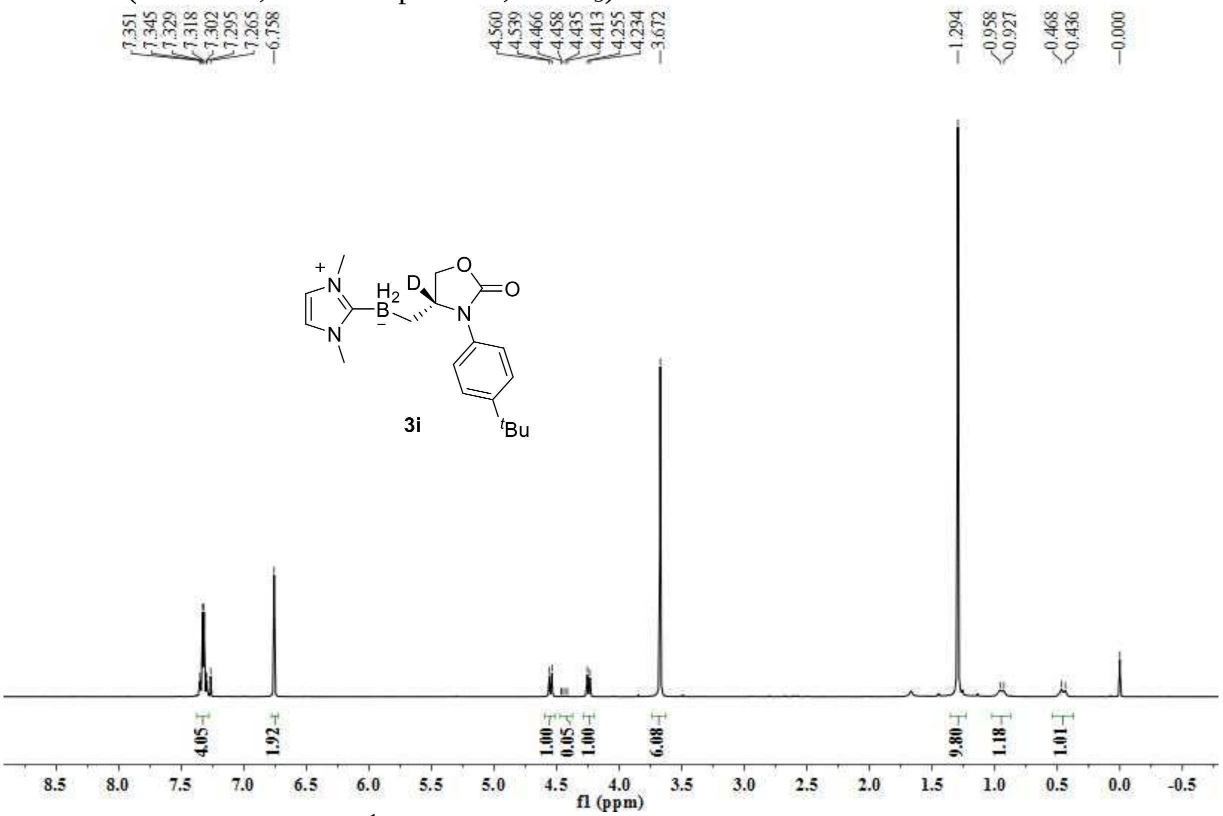

Supplementary Figure 88. ^{1}H NMR spectrum of compound **3i**

^{13}C NMR (100 MHz, room temperature, $CDCl_3$)

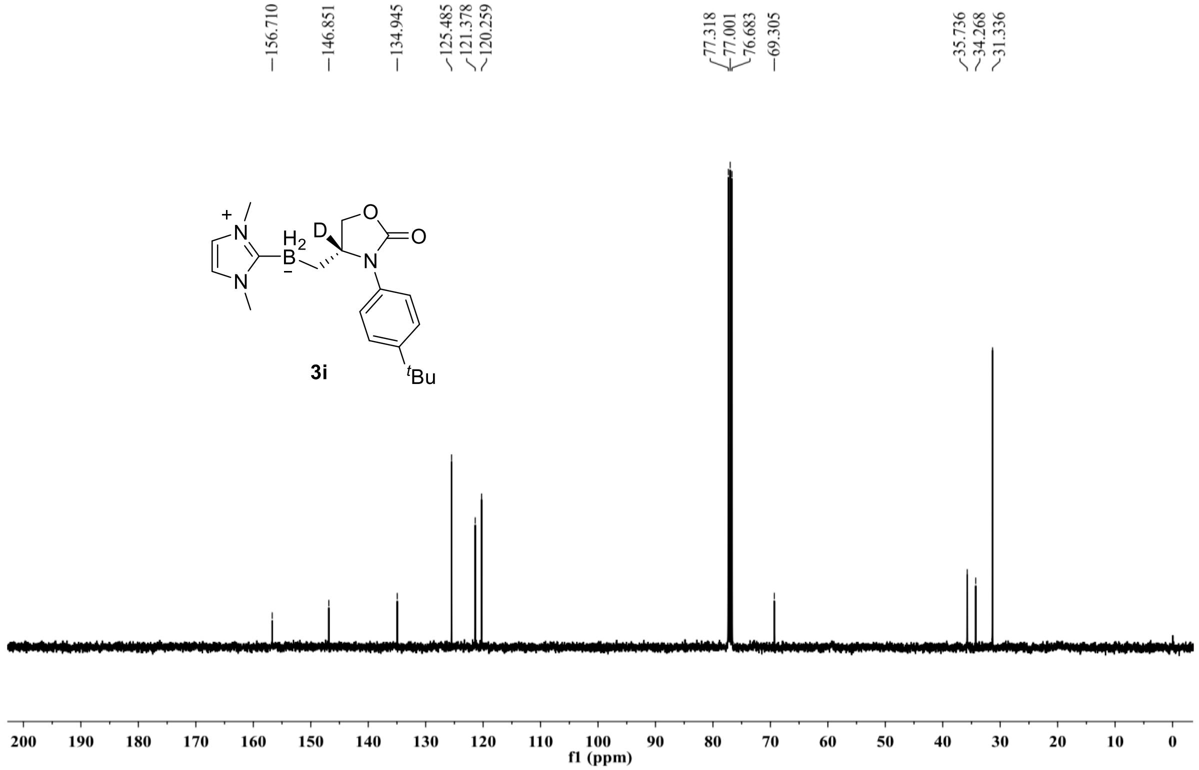

Supplementary Figure 89. ^{13}C NMR spectrum of compound **3i**

^{11}B NMR (128 MHz, room temperature, $CDCl_3$)

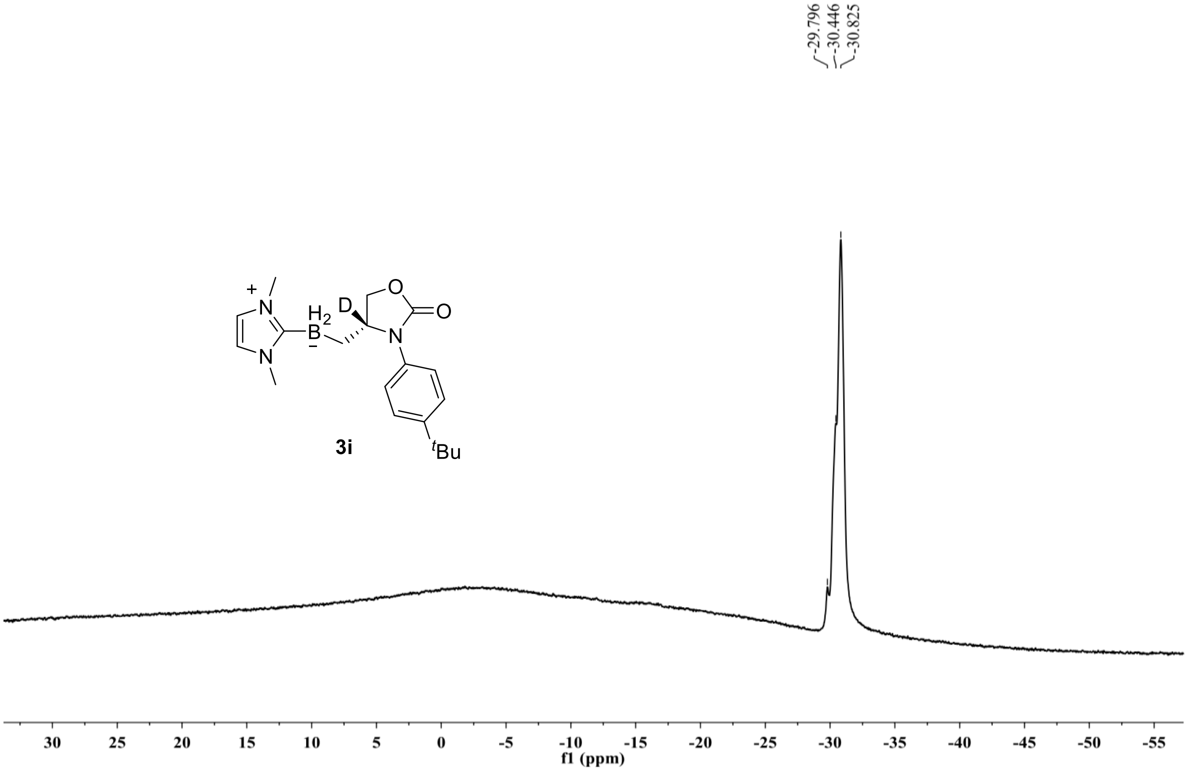

Supplementary Figure 90. ^{11}B NMR spectrum of compound **3i**

HPLC spectrum of racemic **3i**

Tray#	: 1
Vial#	: 46
Data File	: XMC-4-327A-3-3.lcd
Method File	: 3AD-H-85-1-214.lcm
Date Acquired	: 2/11/2022 2:34:15 PM
Date Processed	: 2/11/2022 3:52:34 PM

<Chromatogram View>

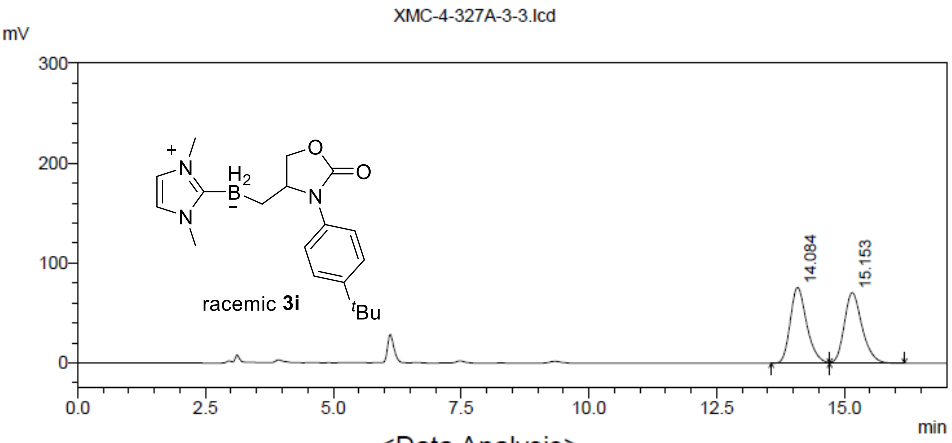

<Data Analysis>

Detector A 214nm

Pesk #	Ret. Time	Height	Area	Area%
1	14.084	75980	1709516	49.898
2	15.153	70633	1716478	50.102
Total		146613	3425994	100.000

Supplementary Figure 91. HPLC spectrum of racemic **3i**

HPLC spectrum of **3i**

Sample Name	:
Tray#	: 1
Vial#	: 47
Data File	: XMC-4-327b-1.lcd
Method File	: 3AD-H-85-1-214.lcm
Date Acquired	: 2/11/2022 2:54:07 PM
Date Processed	: 2/11/2022 3:53:58 PM

<Chromatogram View>

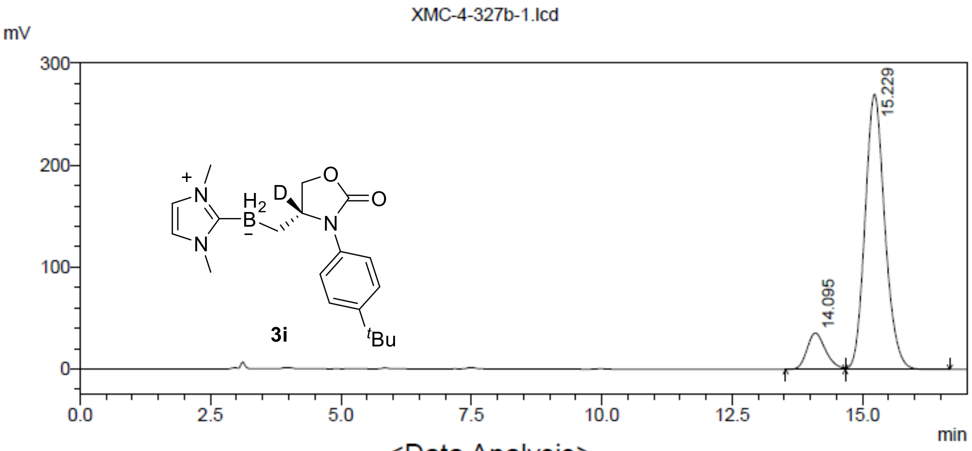

<Data Analysis>

Detector A 214nm

Pesk #	Ret. Time	Height	Area	Area%
1	14.095	35648	886744	11.051
2	15.229	269809	7137628	88.949
Total		305457	8024372	100.000

Supplementary Figure 92. HPLC spectrum of **3i**

^{1}H NMR (400 MHz, room temperature, $CDCl_3$)

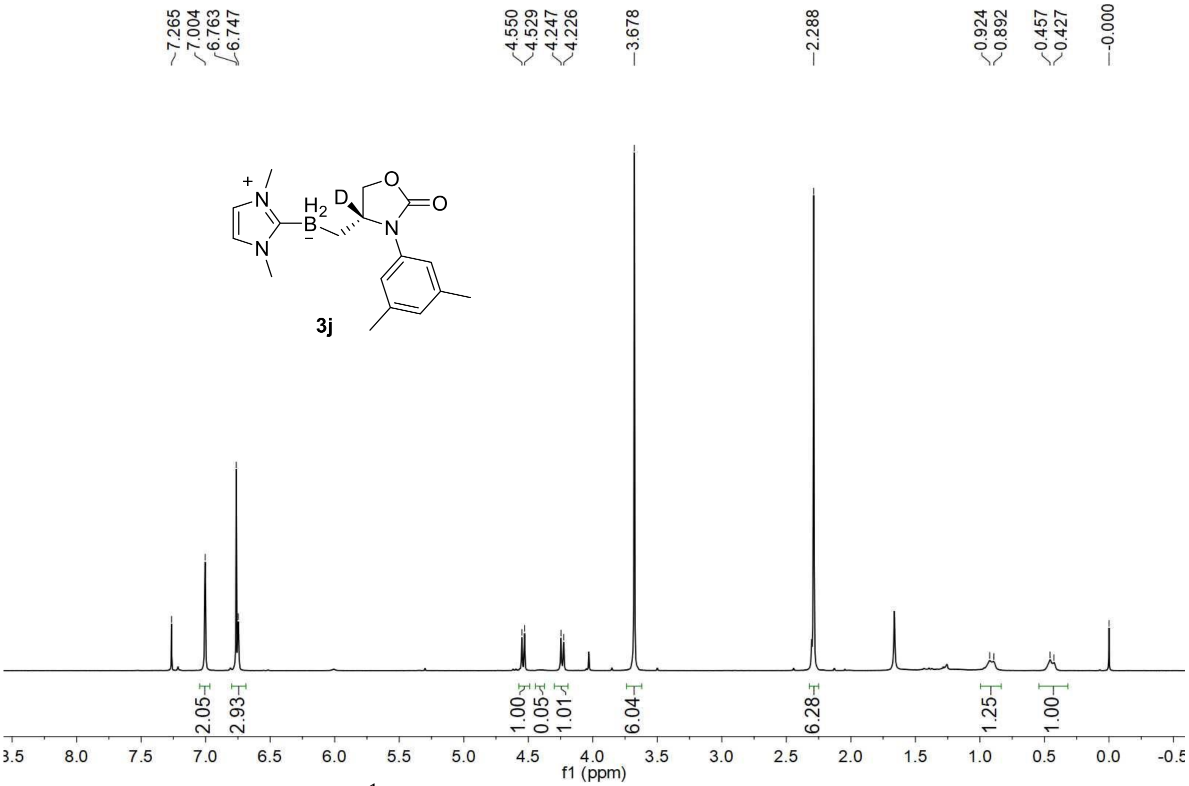

Supplementary Figure 93. ^{1}H NMR spectrum of compound **3j**

^{13}C NMR (100 MHz, room temperature, $CDCl_3$)

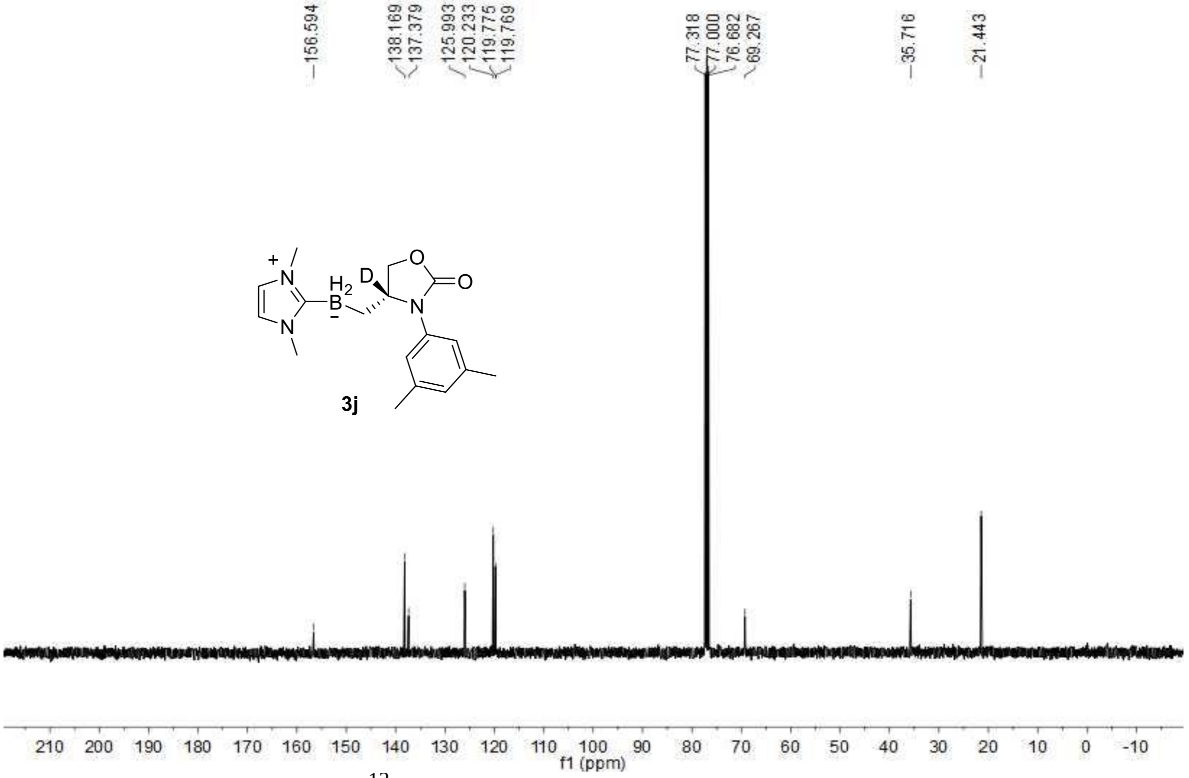

Supplementary Figure 94. ^{13}C NMR spectrum of compound **3j**

^{11}B NMR (128 MHz, room temperature, $CDCl_3$)

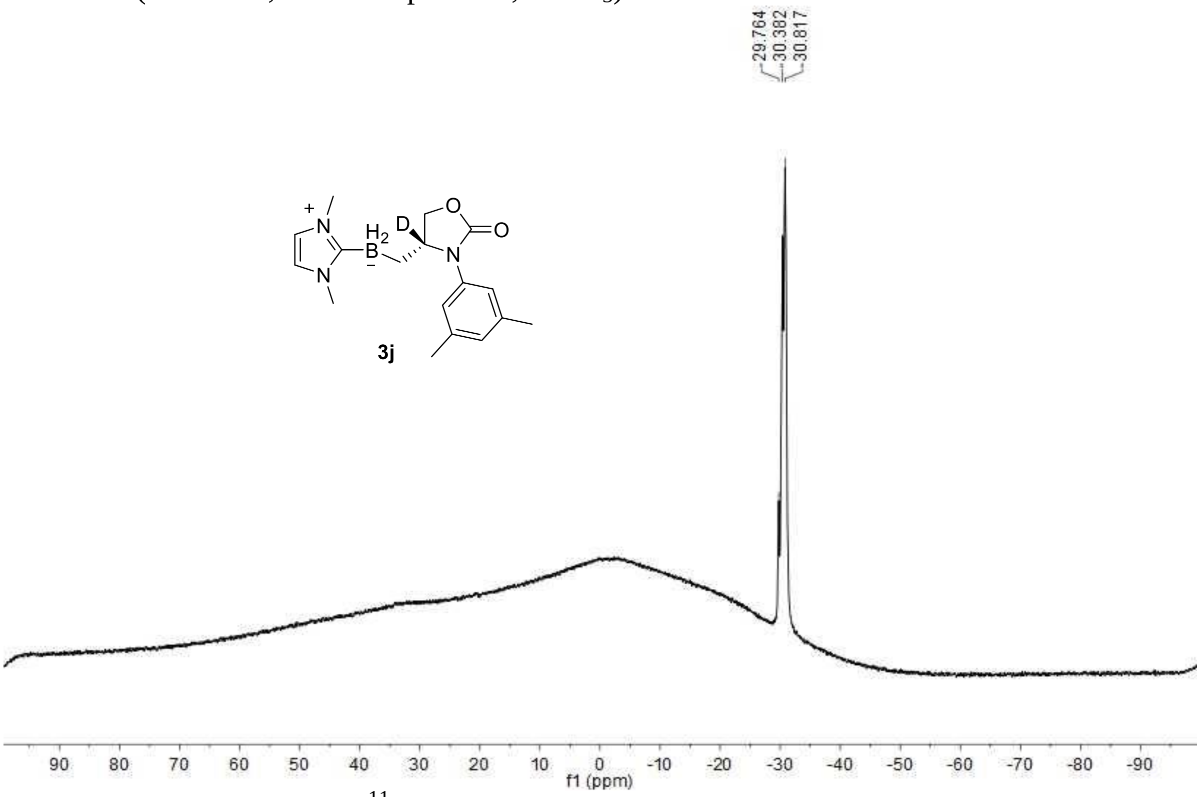

Supplementary Figure 95. ^{11}B NMR spectrum of compound **3j**

HPLC spectrum of racemic **3j**

Tray#	: 1
Vial#	: 20
Data File	: sql-6-405-1-1-(11).lcd
Method File	: 4OD-H-60-0.5-214.lcm
Date Acquired	: 1/19/2022 12:09:04 AM
Date Processed	: 1/25/2022 1:18:26 AM

<Chromatogram View>

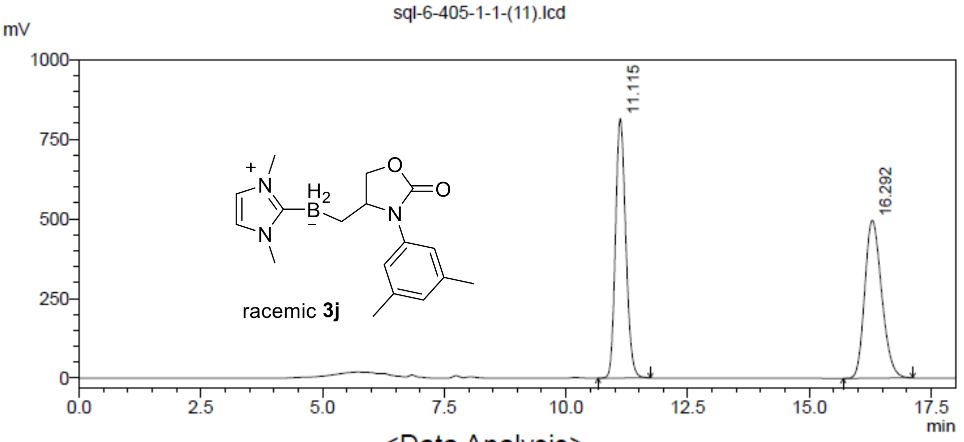

<Data Analysis>

Detector A 214nm

Pesk #	Ret. Time	Height	Area	Area%
1	11.115	815076	11988374	49.950
2	16.292	496312	12012280	50.050
Total		1311388	24000653	100.000

Supplementary Figure 96. HPLC spectrum of racemic **3j**

HPLC spectrum of **3j**

Tray#	: 1
Vial#	: 21
Data File	: sql-6-405-2-(11).lcd
Method File	: 4OD-H-60-0.5-214.lcm
Date Acquired	: 1/18/2022 11:48:30 PM
Date Processed	: 1/25/2022 1:19:16 AM

<Chromatogram View>

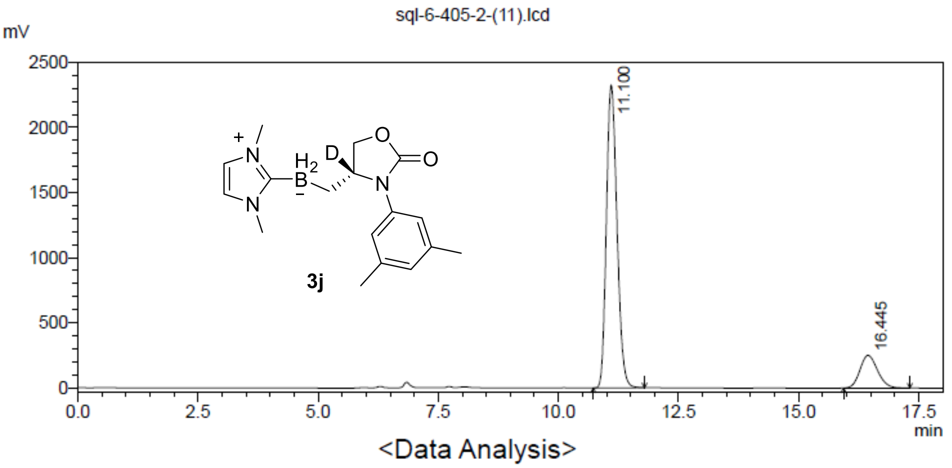

<Data Analysis>

Detector A 214nm

Pesk #	Ret. Time	Height	Area	Area%
1	11.100	2324860	35582622	84.809
2	16.445	251081	6373788	15.191
Total		2575940	41956410	100.000

Supplementary Figure 97. HPLC spectrum of **3j**

^{1}H NMR (400 MHz, room temperature, $CDCl_3$)

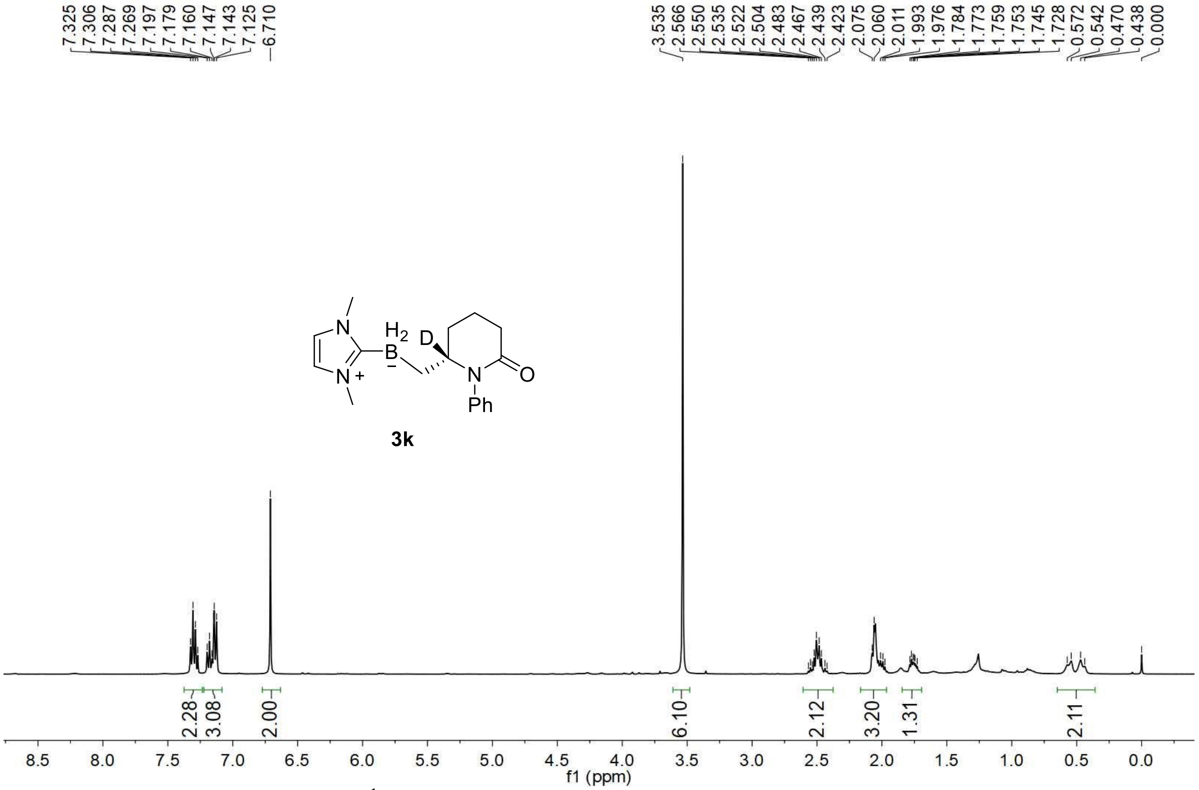

Supplementary Figure 98. ^{1}H NMR spectrum of compound **3k**

^{13}C NMR (100 MHz, room temperature, $CDCl_3$)

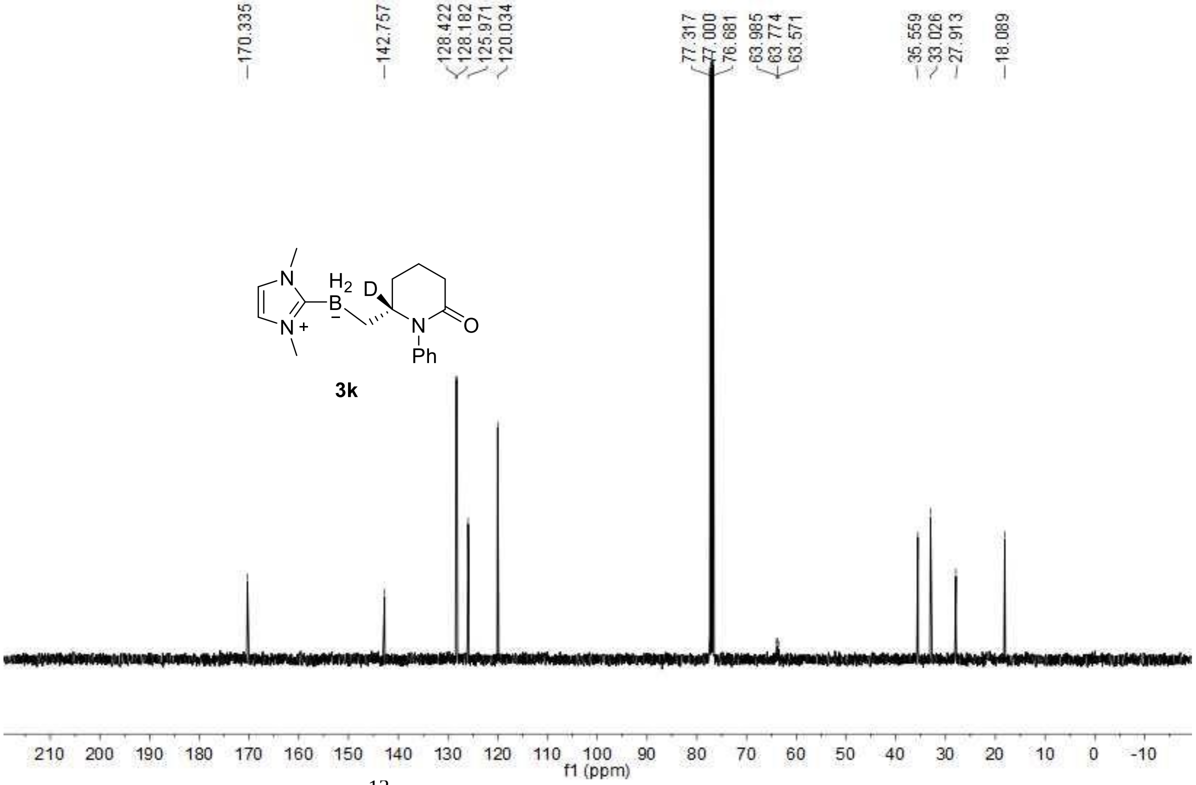

Supplementary Figure 99. ^{13}C NMR spectrum of compound **3k**

^{11}B NMR (128 MHz, room temperature, $CDCl_3$)

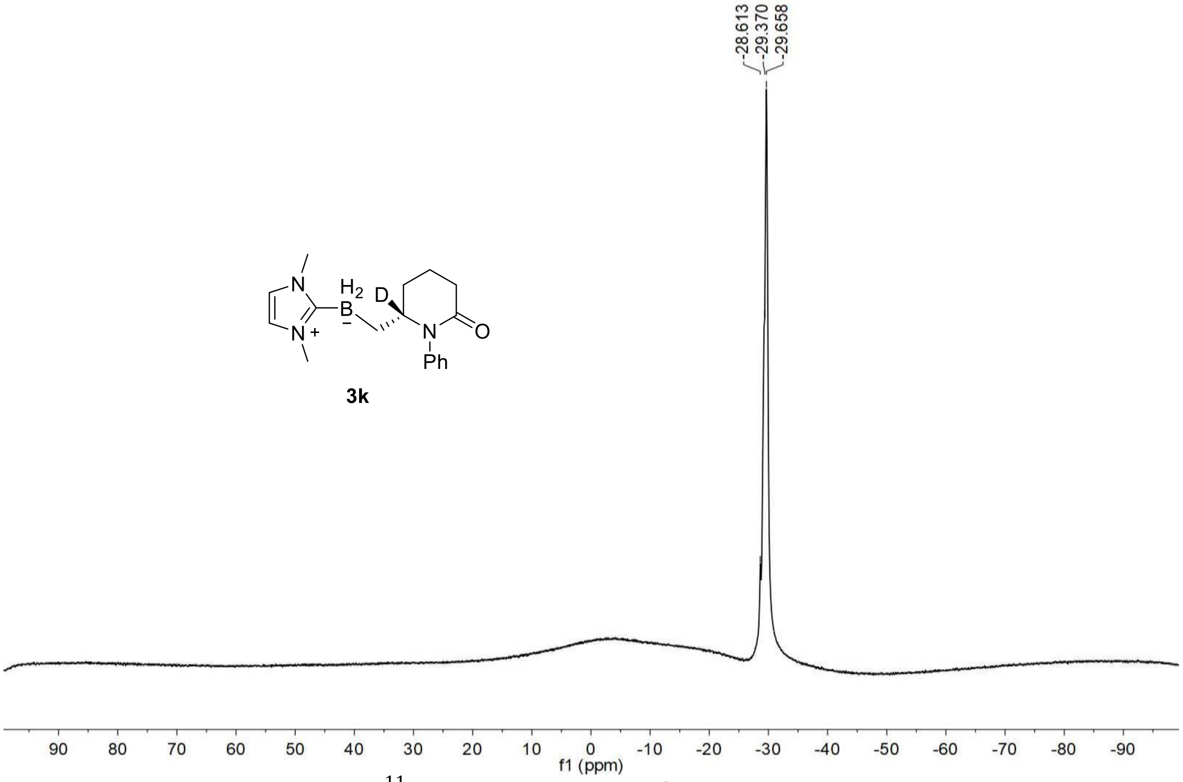

Supplementary Figure 100. ^{11}B NMR spectrum of compound **3k**

HPLC spectrum of racemic **3k**

Sample Name	:
Tray#	: 1
Vial#	: 28
Data File	: sql-6-382-1-4-(10).lcd
Method File	: 3AD-H-90-0.5-214-100MIN.lcm
Date Acquired	: 1/20/2022 2:51:06 PM
Date Processed	: 1/25/2022 1:04:36 AM

<Chromatogram View>

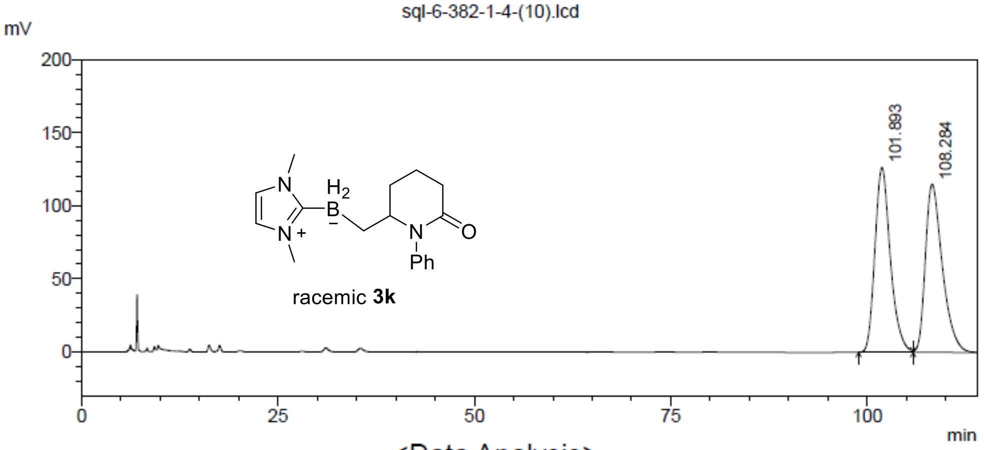

<Data Analysis>

Detector A 214nm

Pesk #	Ret. Time	Height	Area	Area%
1	101.893	126566	17323282	50.030
2	108.284	115079	17302226	49.970
Total		241645	34625508	100.000

Supplementary Figure 101. HPLC spectrum of racemic **3k**

HPLC spectrum of **3k**

Tray#	: 1
Vial#	: 29
Data File	: sql-7-4-(10).lcd
Method File	: 3AD-H-90-0.5-214-100MIN.lcm
Date Acquired	: 1/20/2022 4:52:17 PM
Date Processed	: 1/25/2022 1:04:53 AM

<Chromatogram View>

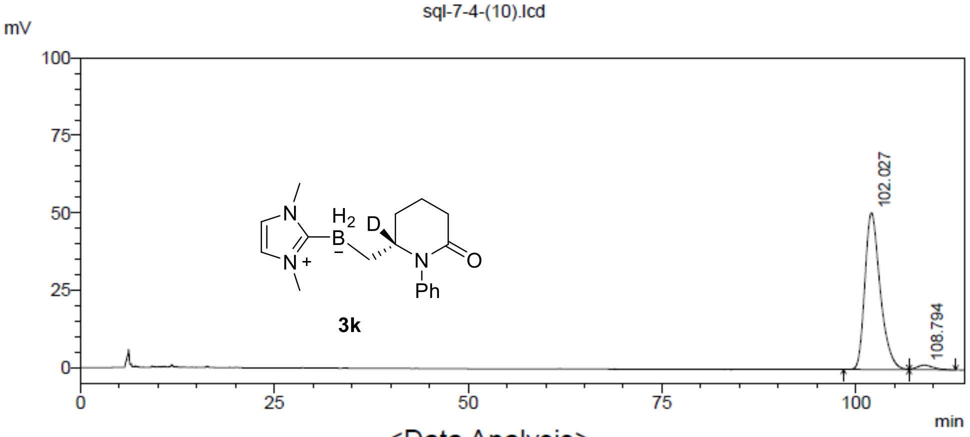

<Data Analysis>

Detector A 214nm

Pesk #	Ret. Time	Height	Area	Area%
1	102.027	50643	6963556	96.973
2	108.794	1471	217381	3.027
Total		52114	7180937	100.000

Supplementary Figure 102. HPLC spectrum of **3k**

^{1}H NMR (400 MHz, room temperature, $CDCl_3$)

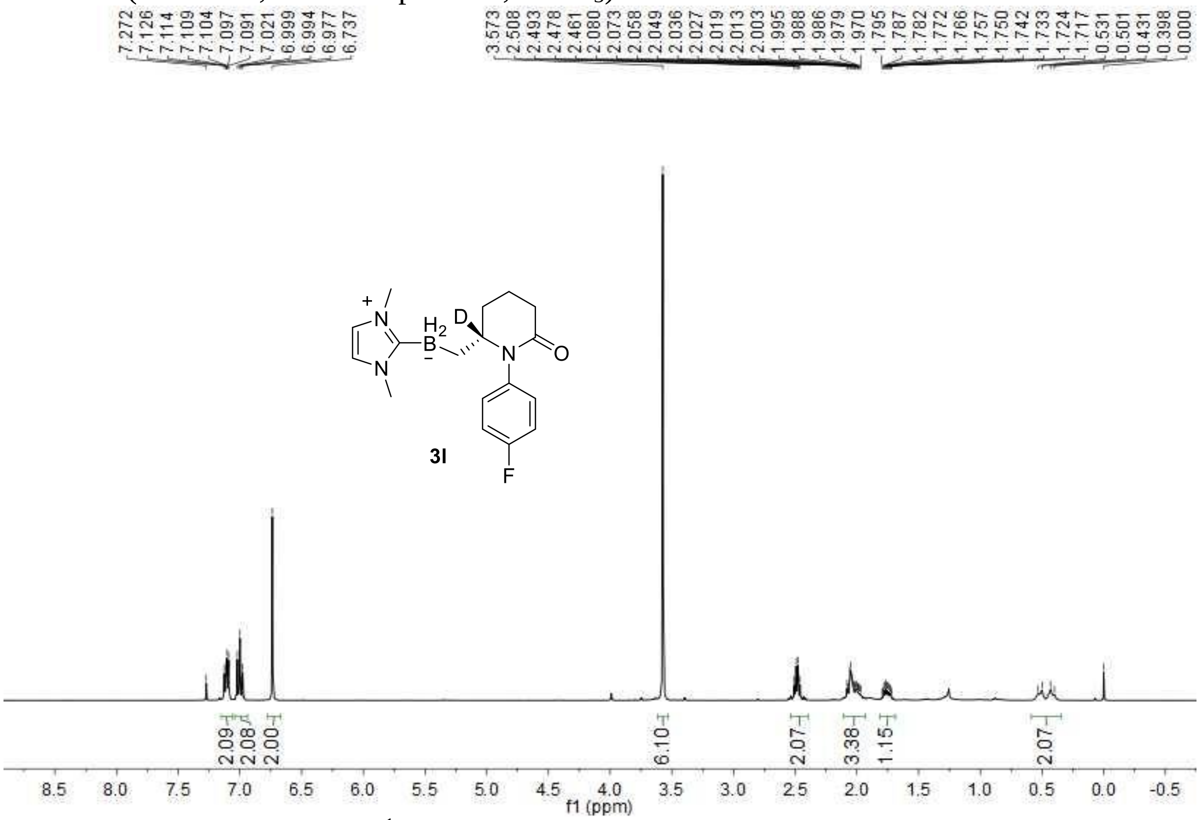

Supplementary Figure 103. ^{1}H NMR spectrum of compound **3l**

^{13}C NMR (100 MHz, room temperature, $CDCl_3$)

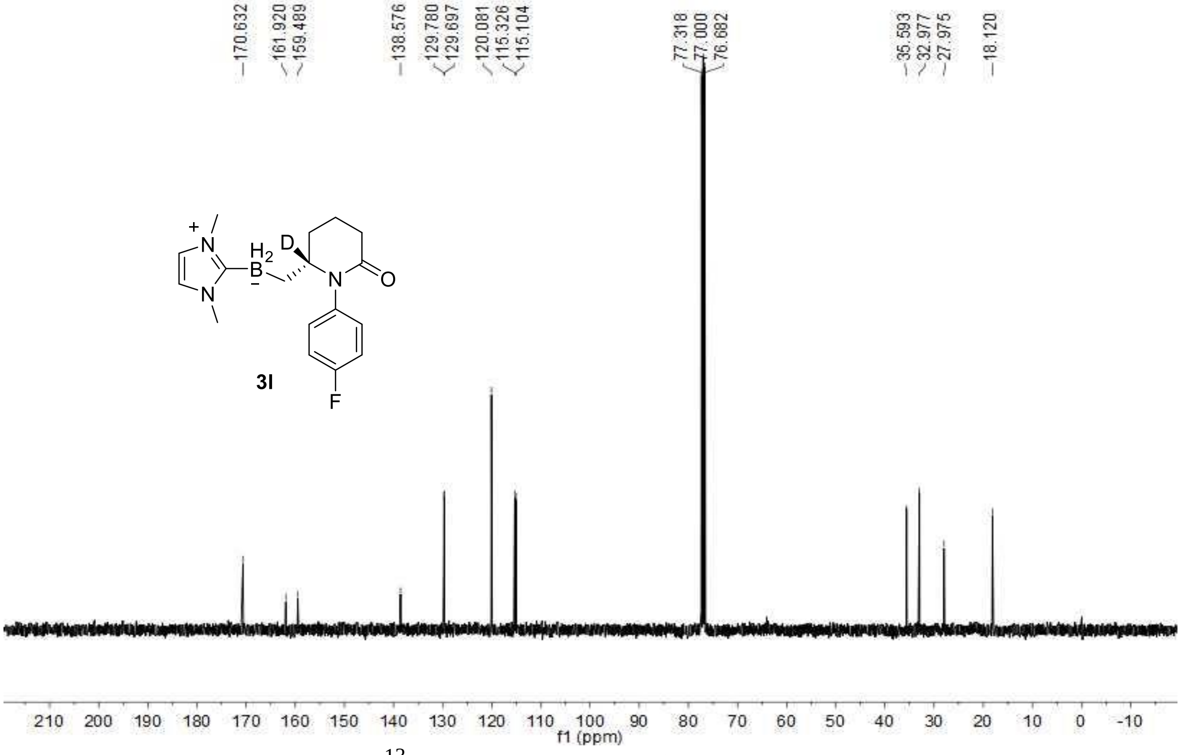

Supplementary Figure 104. ^{13}C NMR spectrum of compound **3l**

^{11}B NMR (128 MHz, room temperature, $CDCl_3$)

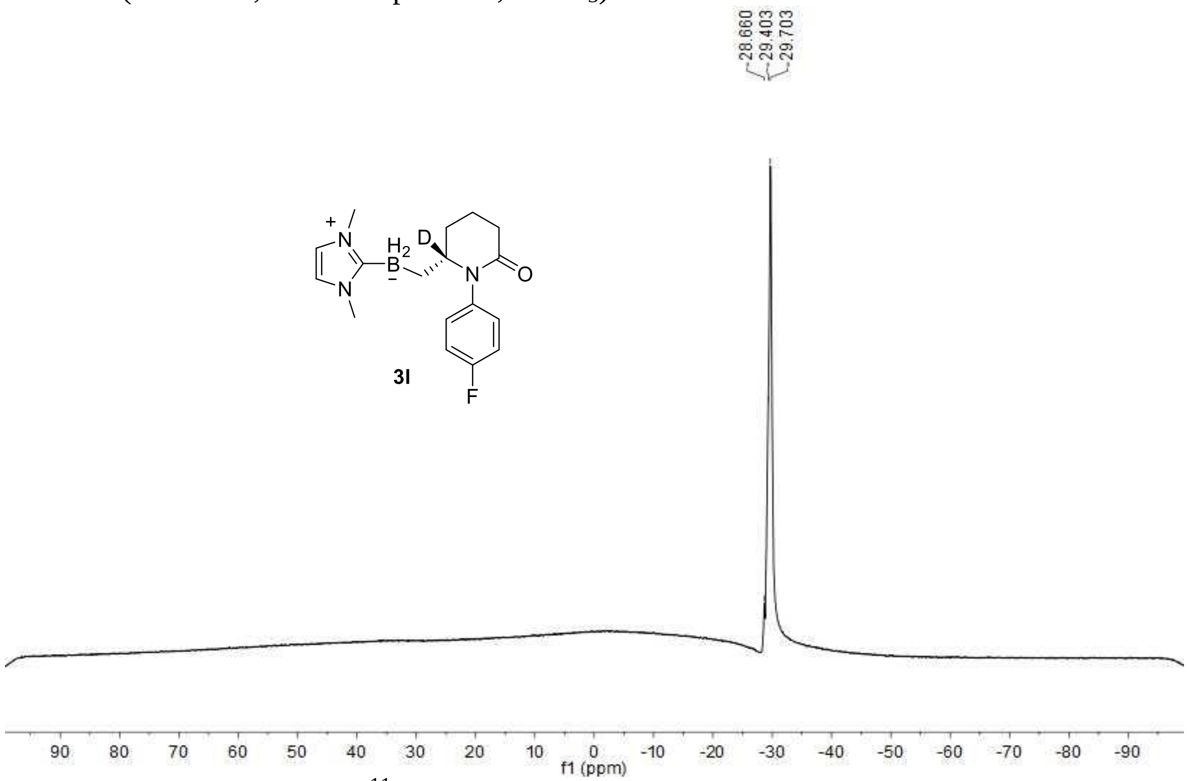

Supplementary Figure 105. ^{11}B NMR spectrum of compound **3l**

^{19}F NMR (376 MHz, room temperature, $CDCl_3$)

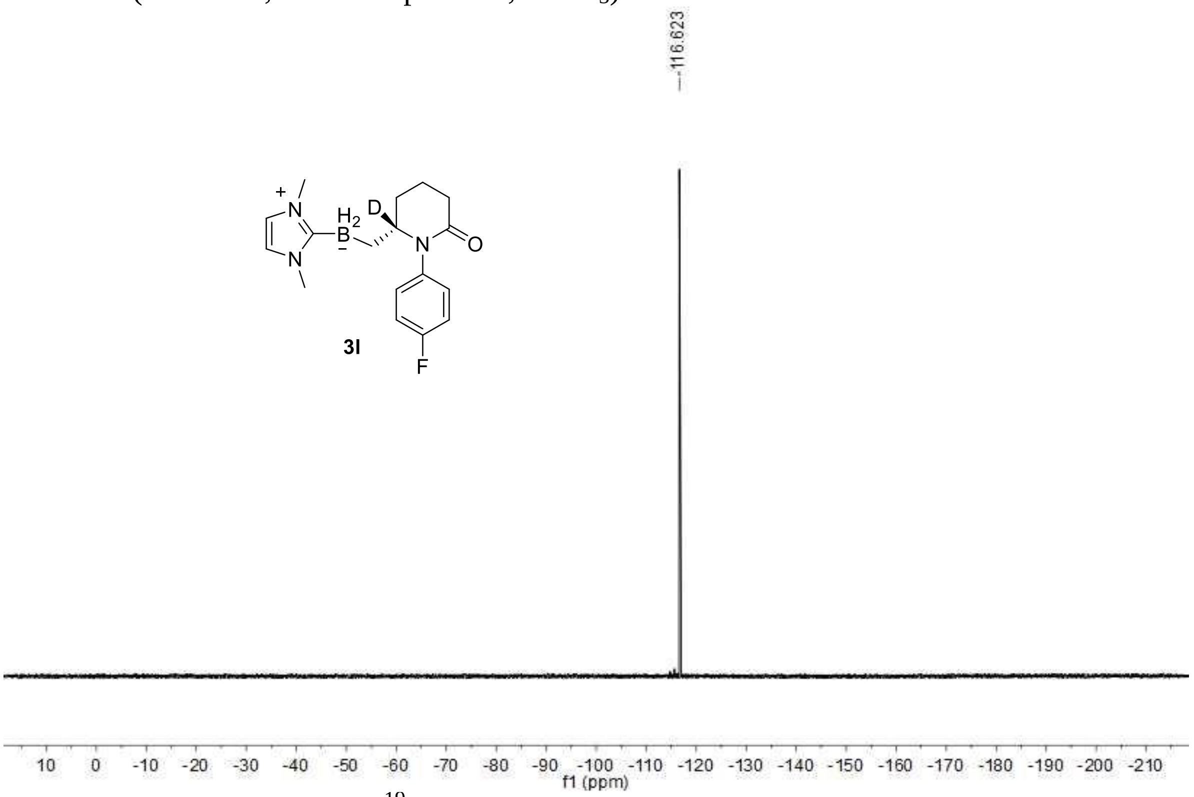

Supplementary Figure 106. ^{19}F NMR spectrum of compound **3l**

HPLC spectrum of racemic **3l**

Tray#	: 1
Vial#	: 46
Data File	: SQL-7-29-1-2-3.lcd
Method File	: 4OD-H-80-1.0-214.lcm
Date Acquired	: 1/23/2022 6:18:49 PM
Date Processed	: 1/23/2022 7:26:38 PM

<Chromatogram View>

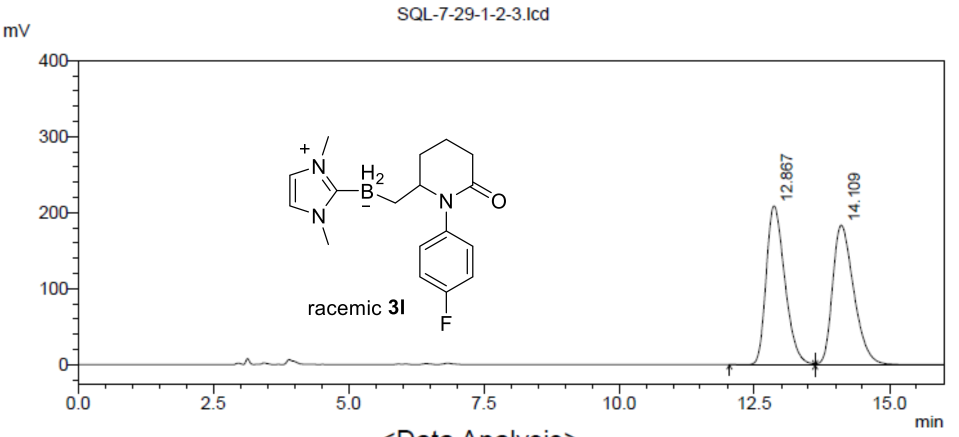

<Data Analysis>

Detector A 214nm

Pesk #	Ret. Time	Height	Area	Area%
1	12.867	209000	5032770	49.825
2	14.109	183659	5068182	50.175
Total		392659	10100951	100.000

Supplementary Figure 107. HPLC spectrum of racemic **3l**

HPLC spectrum of **3l**

Tray#	: 1
Vial#	: 1
Data File	: sql-7-29-2-1.lcd
Method File	: 4OD-H-80-1.0-214.lcm
Date Acquired	: 1/25/2022 5:53:25 AM
Date Processed	: 1/25/2022 8:22:21 AM

<Chromatogram View>

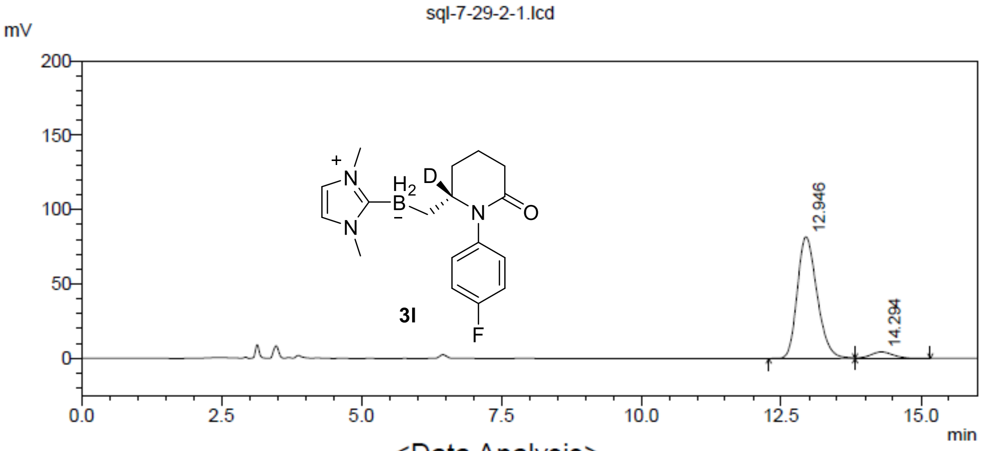

<Data Analysis>

Detector A 214nm

Pesk #	Ret. Time	Height	Area	Area%
1	12.946	81853	2015753	94.224
2	14.294	4380	123562	5.776
Total		86232	2139315	100.000

Supplementary Figure 108. HPLC spectrum of **3l**

^{1}H NMR (400 MHz, room temperature, $CDCl_3$)

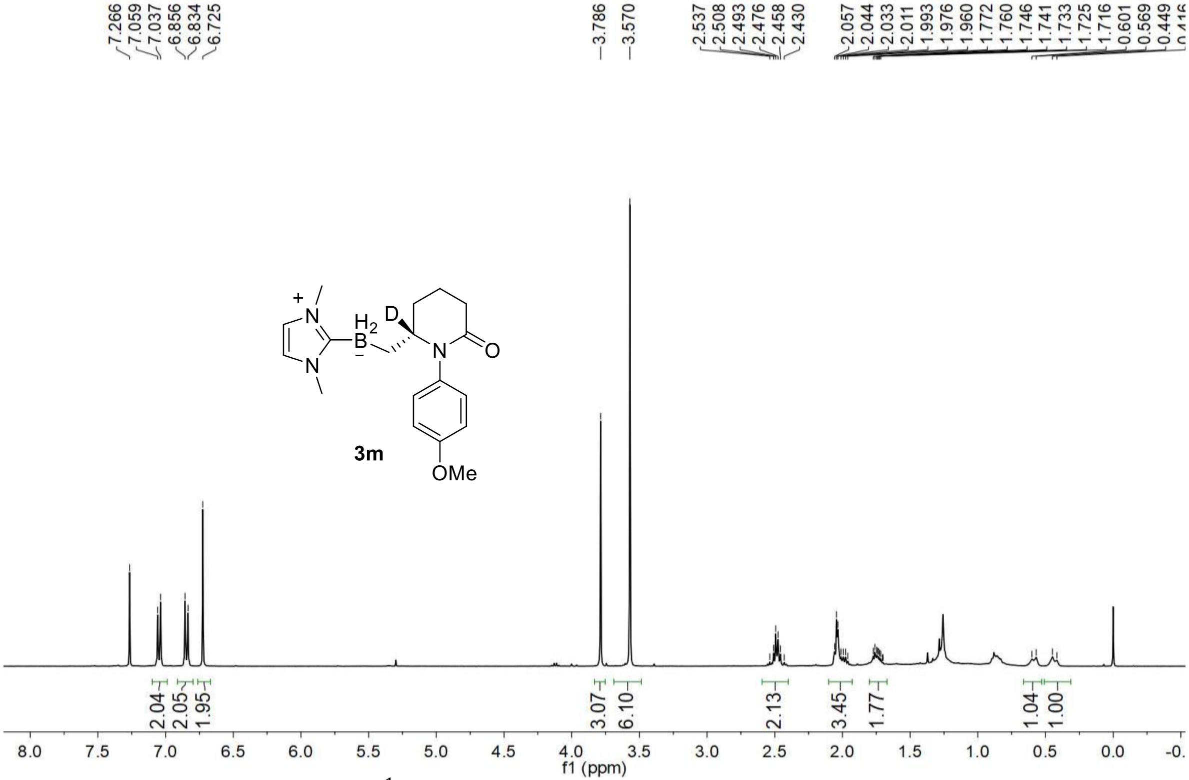

Supplementary Figure 109. ^{1}H NMR spectrum of compound **3m**

^{13}C NMR (100 MHz, room temperature, $CDCl_3$)

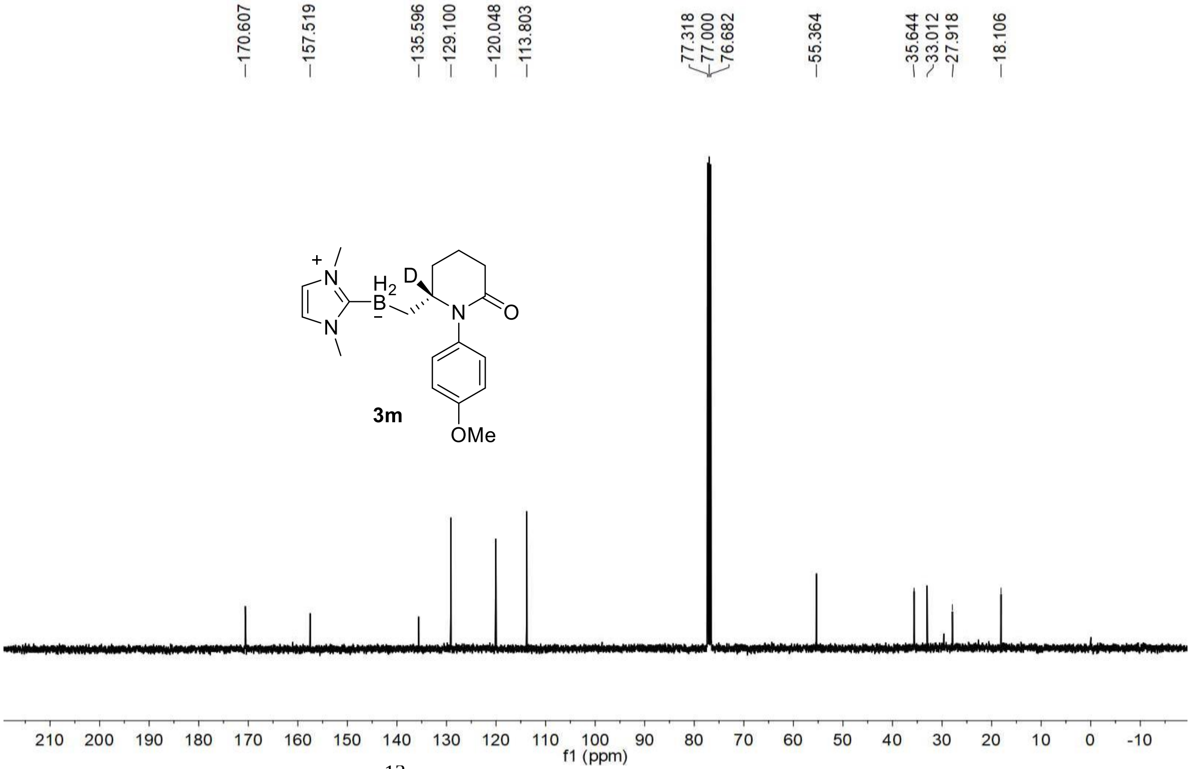

Supplementary Figure 110. ^{13}C NMR spectrum of compound **3m**

^{11}B NMR (128 MHz, room temperature, $CDCl_3$)

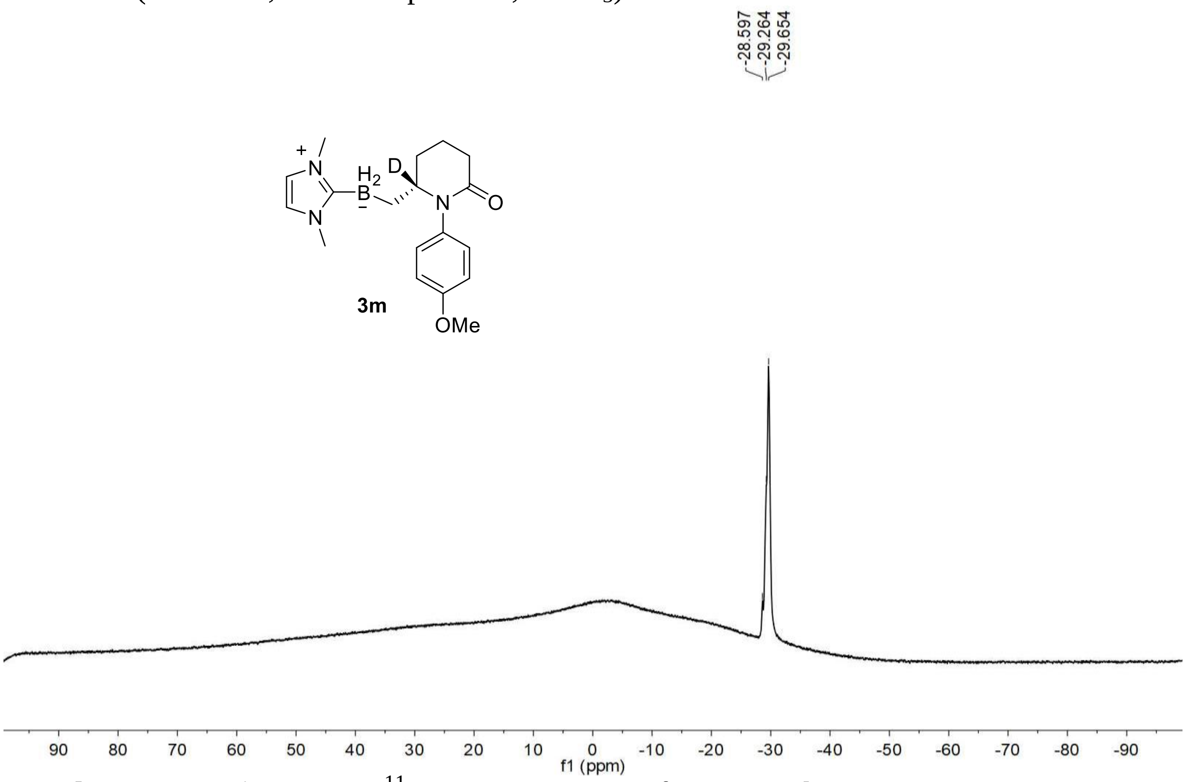

Supplementary Figure 111. ^{11}B NMR spectrum of compound **3m**

HPLC spectrum of racemic **3m**

Sample Name	:
Tray#	: 1
Vial#	: 10
Data File	: sql-7-50-1.lcd
Method File	: 4OD-H-70-1.0-214.lcm
Date Acquired	: 2/15/2022 2:35:56 PM
Date Processed	: 2/15/2022 3:42:15 PM

<Chromatogram View>

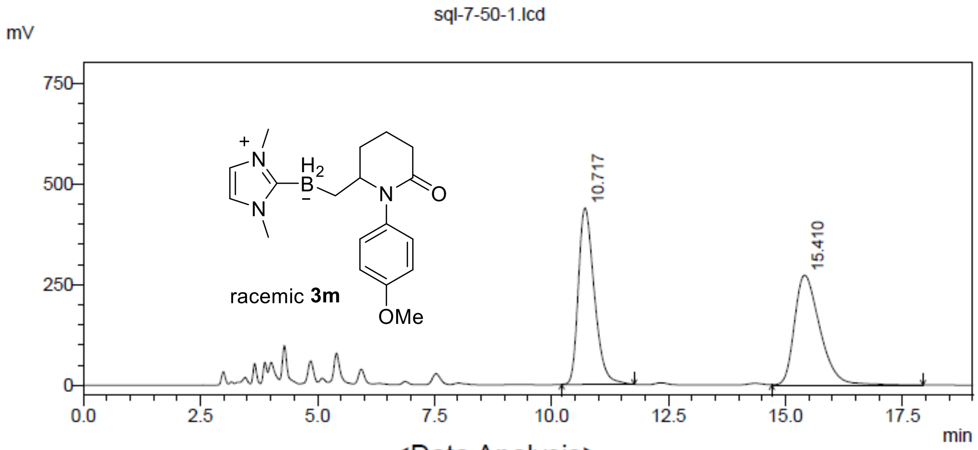

<Data Analysis>

Detector A 214nm

Pesk #	Ret. Time	Height	Area	Area%
1	10.717	437864	10414336	50.161
2	15.410	272954	10347531	49.839
Total		710818	20761868	100.000

Supplementary Figure 112. HPLC spectrum of racemic **3m**

HPLC spectrum of **3m**

Sample Name	:
Tray#	: 1
Vial#	: 11
Data File	: sql-7-50-2.lcd
Method File	: 4OD-H-70-1.0-214.lcm
Date Acquired	: 2/15/2022 2:57:45 PM
Date Processed	: 2/15/2022 3:41:22 PM

<Chromatogram View>

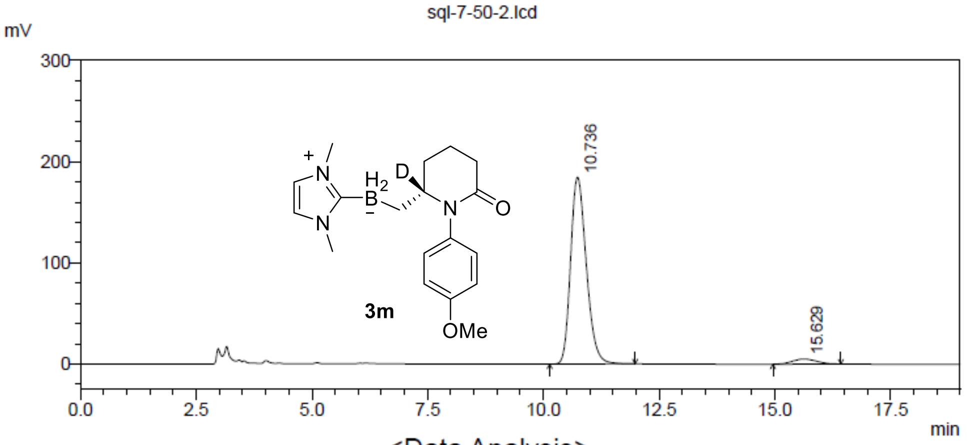

<Data Analysis>

Detector A 214nm

Pesk #	Ret. Time	Height	Area	Area%
1	10.736	184656	4366000	96.006
2	15.629	5003	181638	3.994
Total		189658	4547639	100.000

Supplementary Figure 113. HPLC spectrum of **3m**

^{1}H NMR (400 MHz, room temperature, $CDCl_3$)

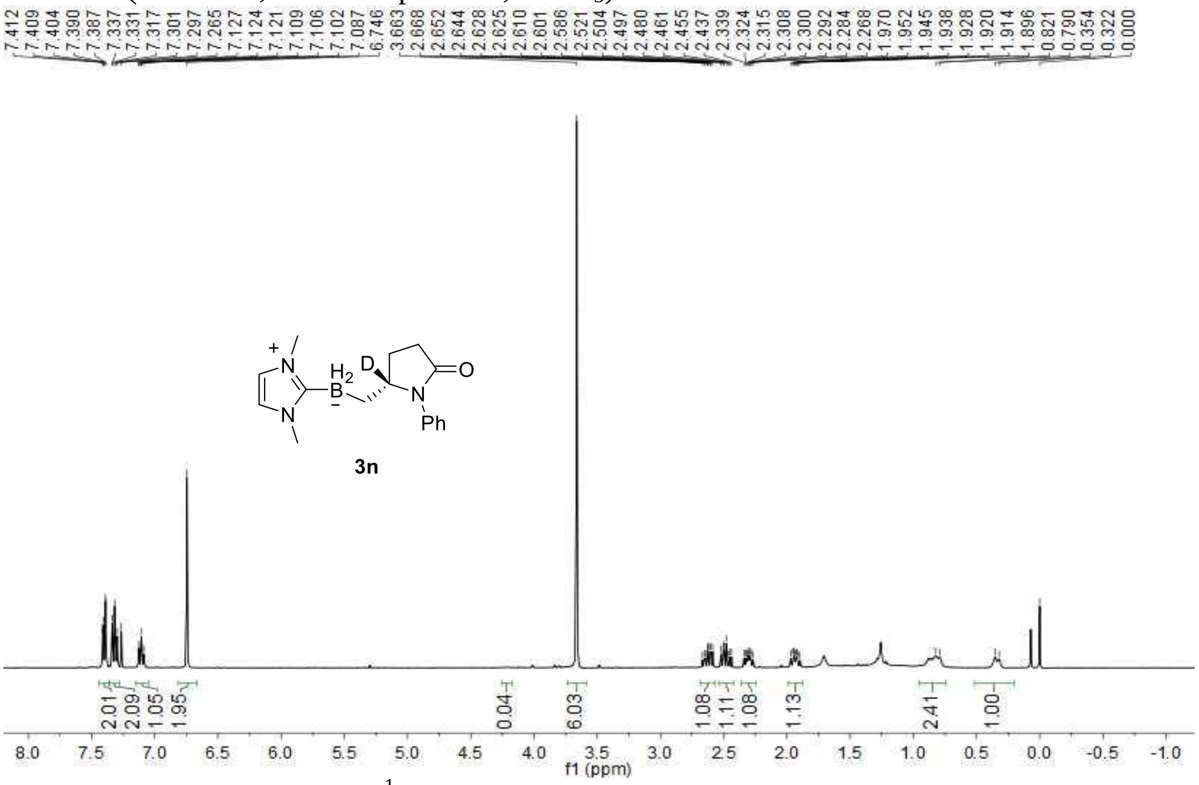

Supplementary Figure 114. ^{1}H NMR spectrum of compound **3n**

^{13}C NMR (100 MHz, room temperature, $CDCl_3$)

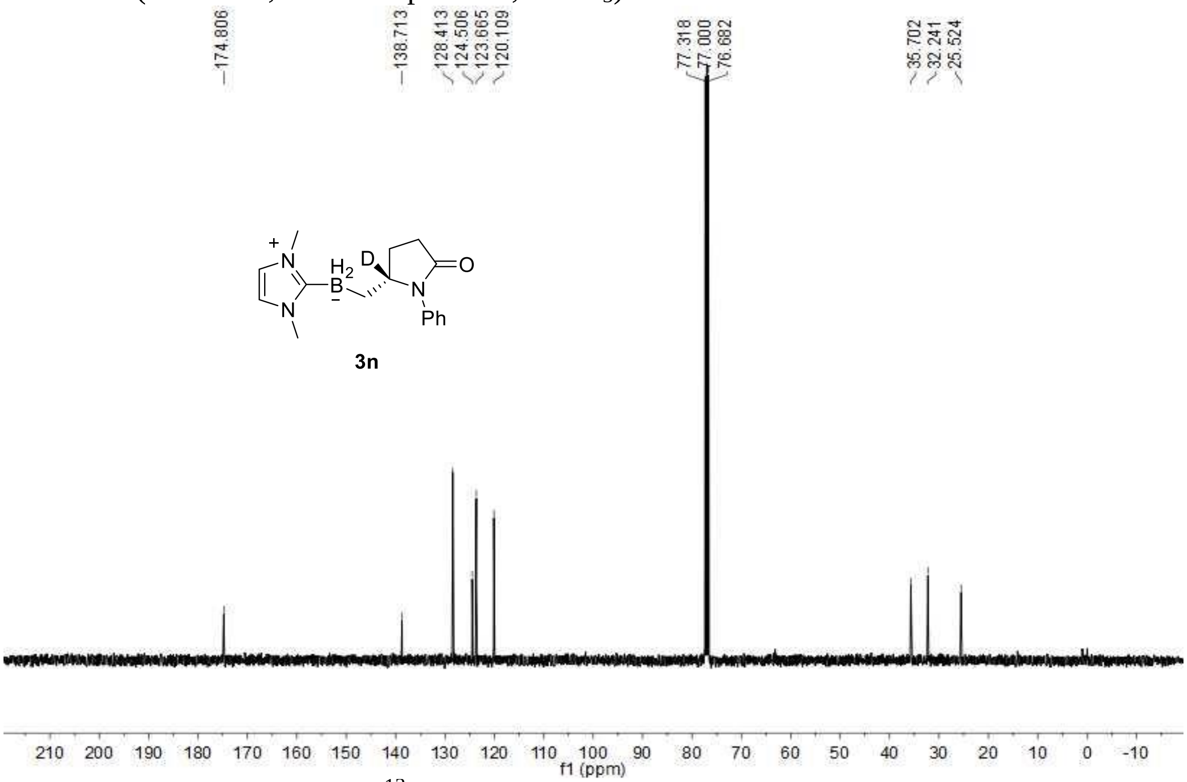

Supplementary Figure 115. ^{13}C NMR spectrum of compound **3n**

^{11}B NMR (128 MHz, room temperature, $CDCl_3$)

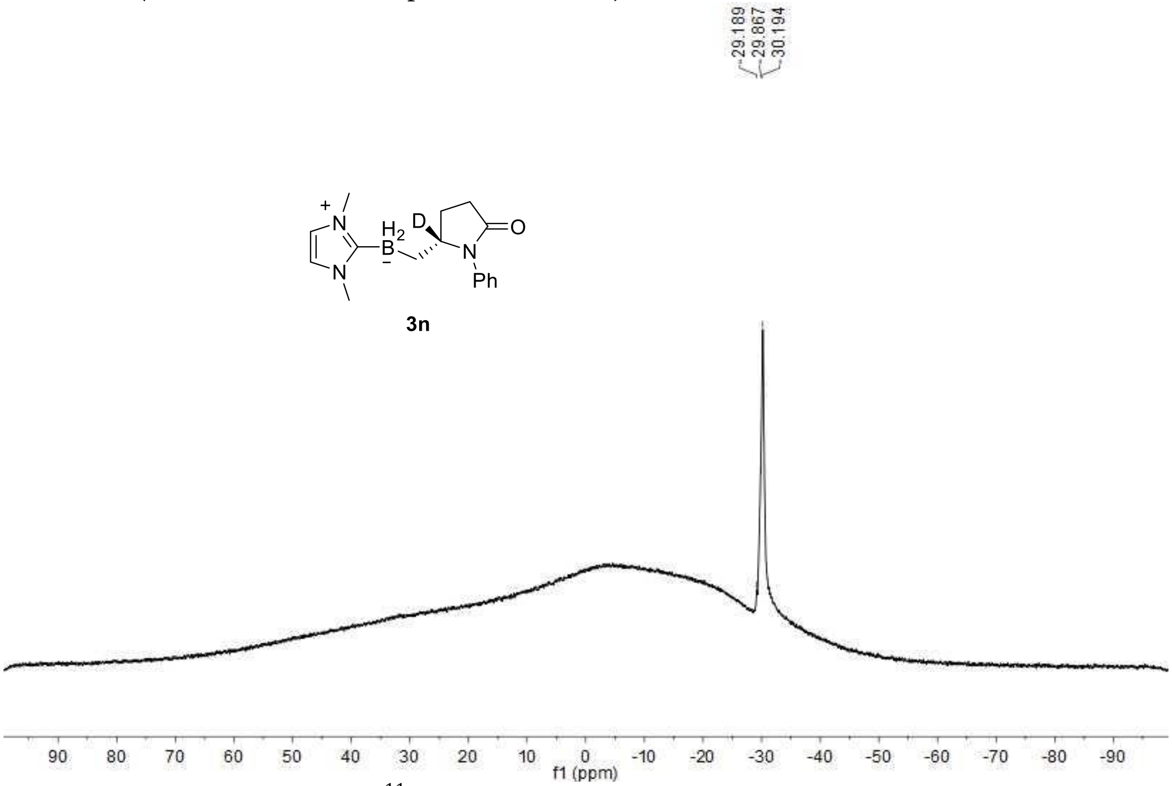

Supplementary Figure 116. ^{11}B NMR spectrum of compound **3n**

HPLC spectrum of racemic **3n**

Tray#	: 1
Vial#	: 10
Data File	: sql-6-351-10.lcd
Method File	: 3AD-H-75-0.5-214-50MIN.lcm
Date Acquired	: 2/2/2022 11:32:22 AM
Date Processed	: 2/2/2022 11:56:46 AM

<Chromatogram View>

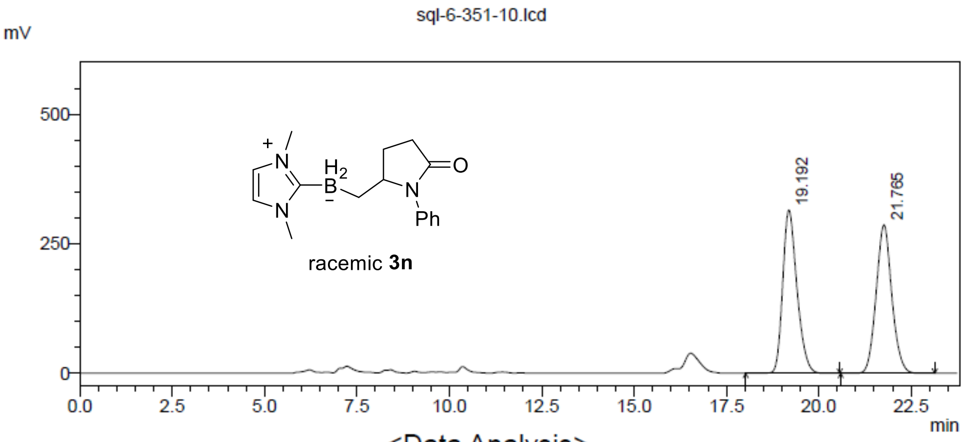

<Data Analysis>

Detector A 214nm

Pesk #	Ret. Time	Height	Area	Area%
1	19.192	315322	8469121	49.935
2	21.765	286879	8491288	50.065
Total		602200	16960408	100.000

Supplementary Figure 117. HPLC spectrum of racemic **3n**

HPLC spectrum of **3n**

Tray#	: 1
Vial#	: 12
Data File	: sql-6-357-1.lcd
Method File	: 3AD-H-75-0.5-214-50MIN.lcm
Date Acquired	: 2/2/2022 11:57:23 AM
Date Processed	: 2/2/2022 12:22:19 PM

<Chromatogram View>

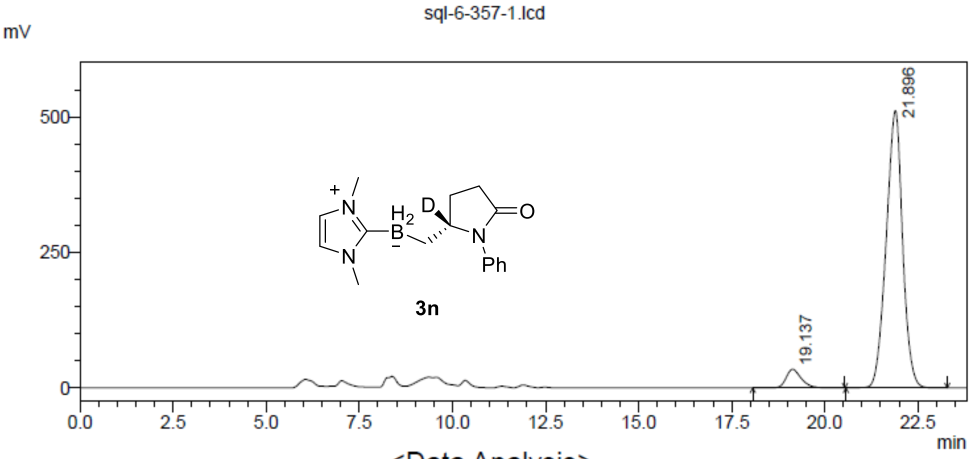

<Data Analysis>

Detector A 214nm

Pesk #	Ret. Time	Height	Area	Area%
1	19.137	34267	962167	5.774
2	21.896	511696	15701001	94.226
Total		545963	16663168	100.000

Supplementary Figure 118. HPLC spectrum of **3n**

^1H NMR (400 MHz, room temperature, $CDCl_3$)

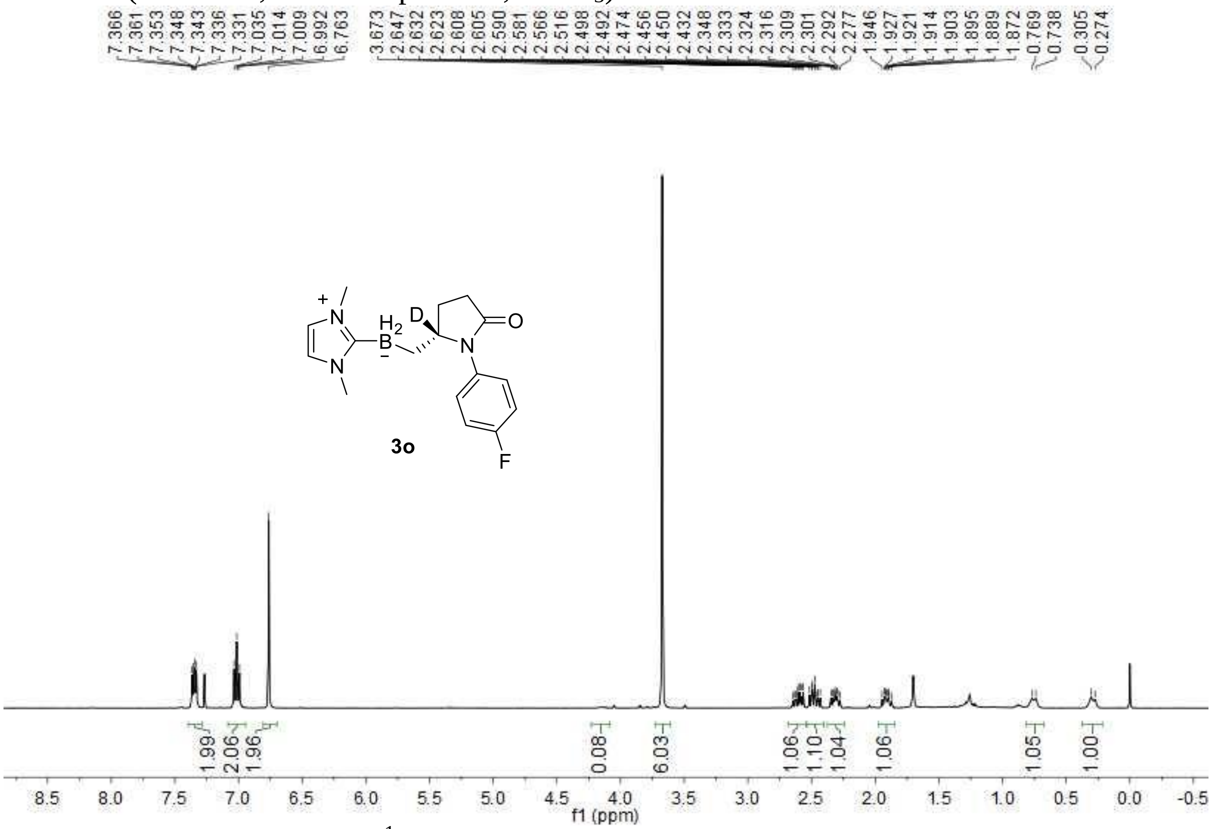

Supplementary Figure 119. ^1H NMR spectrum of compound **3o**

^{13}C NMR (100 MHz, room temperature, $CDCl_3$)

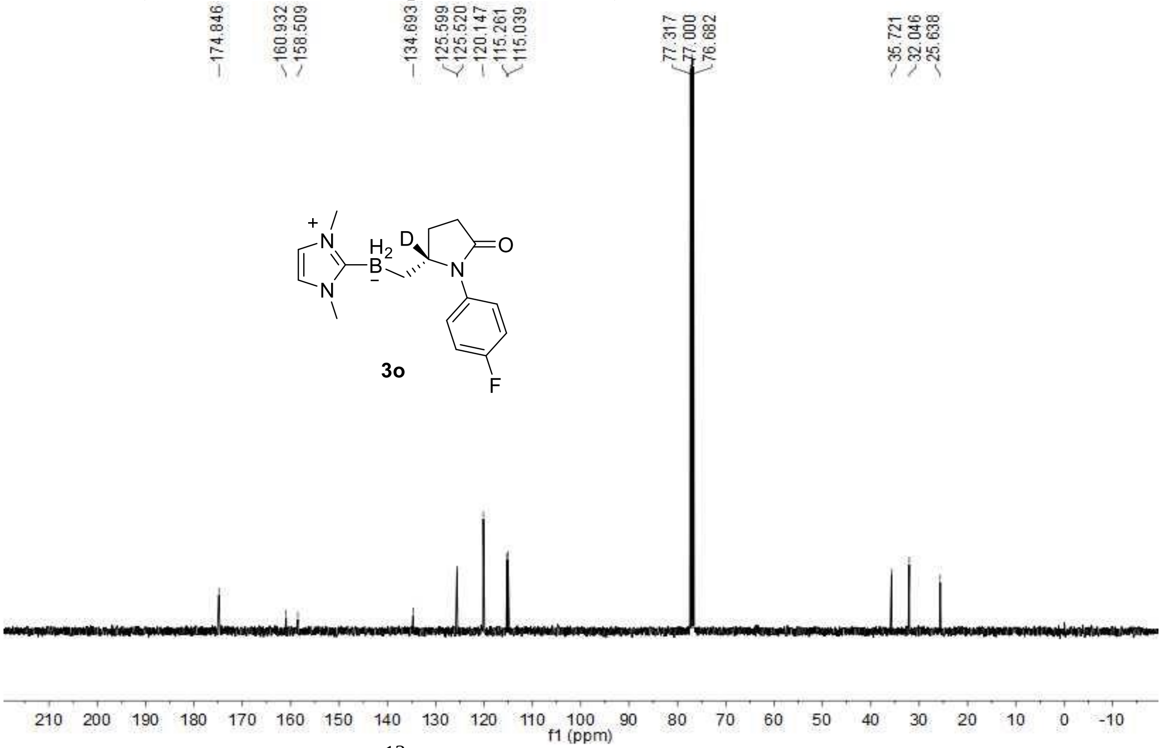

Supplementary Figure 120. ^{13}C NMR spectrum of compound **3o**

^{11}B NMR (128 MHz, room temperature, $CDCl_3$)

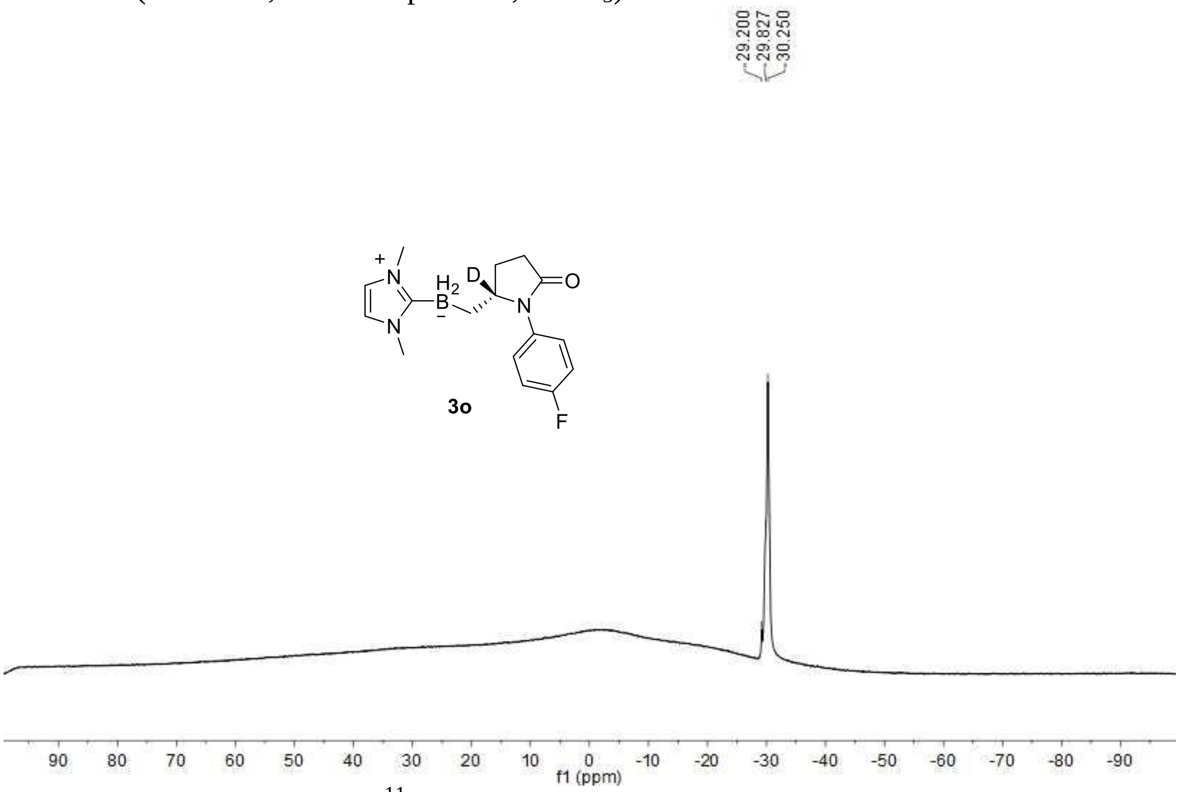

Supplementary Figure 121. ^{11}B NMR spectrum of compound **3o**

^{19}F NMR (376 MHz, room temperature, $CDCl_3$)

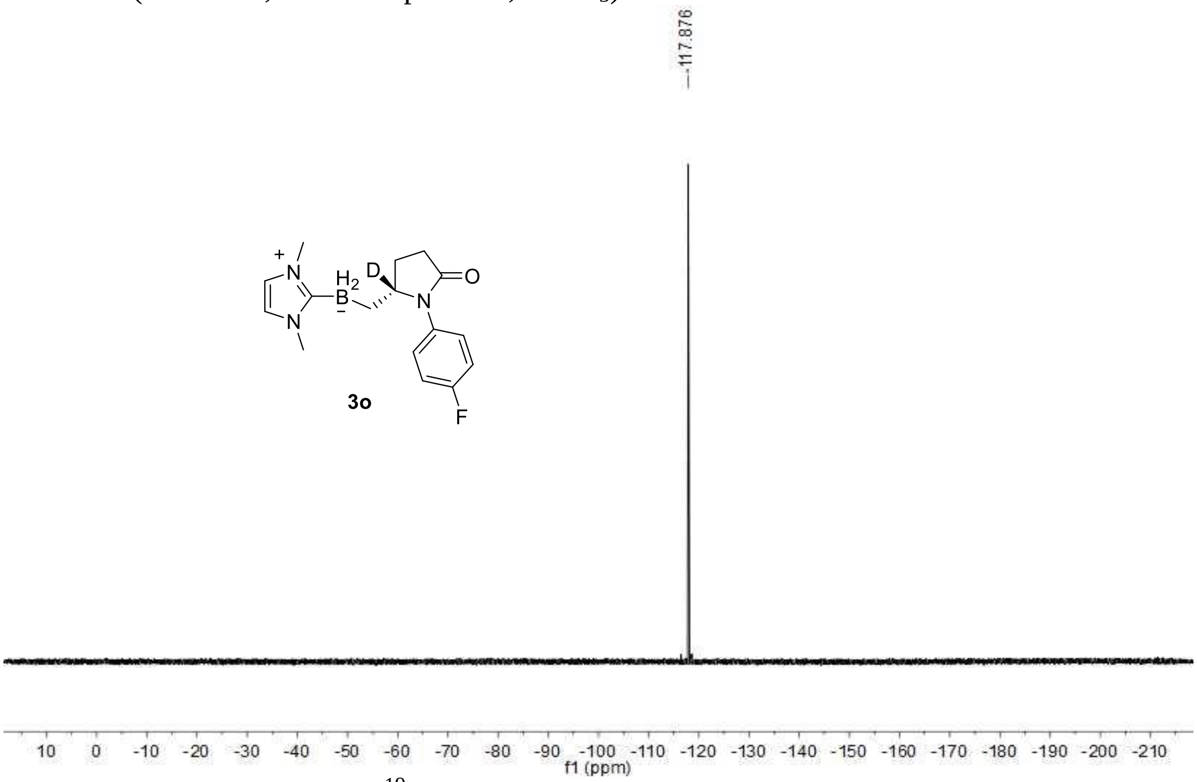

Supplementary Figure 122. ^{19}F NMR spectrum of compound **3o**

HPLC spectrum of racemic **3o**

Tray#	: 1
Vial#	: 10
Data File	: sql-6-398-1-(12).lcd
Method File	: 3AD-H-75-0.5-214-50MIN.lcm
Date Acquired	: 1/25/2022 2:31:00 AM
Date Processed	: 1/25/2022 8:49:47 AM

<Chromatogram View>

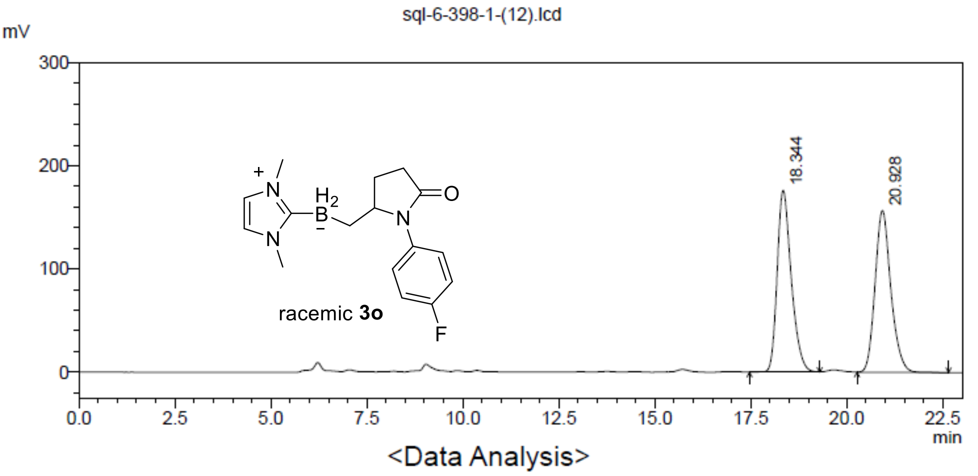

<Data Analysis>

Detector A 214nm

Pesk #	Ret. Time	Height	Area	Area%
1	18.344	176393	4524873	50.149
2	20.928	157007	4498063	49.851
Total		333400	9022936	100.000

Supplementary Figure 123. HPLC spectrum of racemic **3o**

HPLC spectrum of **3o**

Tray#	: 1
Vial#	: 11
Data File	: sql-7-19-4-(12).lcd
Method File	: 3AD-H-75-0.5-214-50MIN.lcm
Date Acquired	: 1/25/2022 2:58:06 AM
Date Processed	: 1/25/2022 8:55:00 AM

<Chromatogram View>

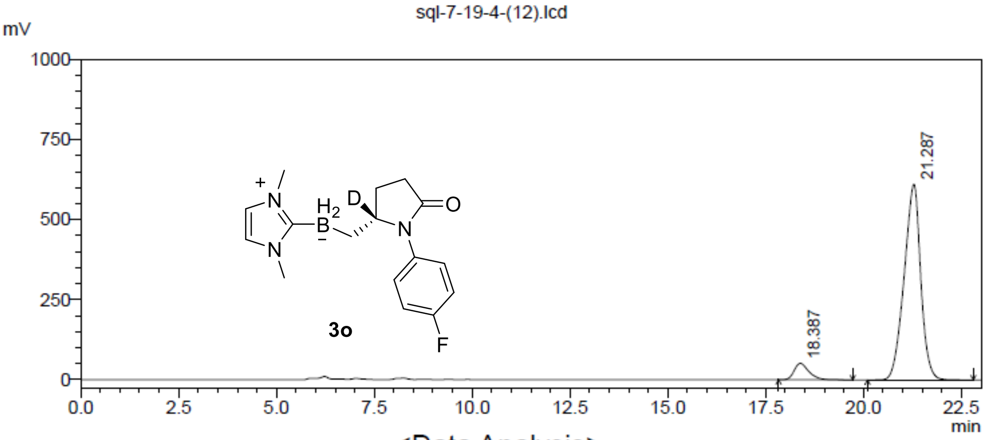

<Data Analysis>

Detector A 214nm

Pesk #	Ret. Time	Height	Area	Area%
1	18.387	51587	1340140	6.935
2	21.287	610328	17984484	93.065
Total		661915	19324625	100.000

Supplementary Figure 124. HPLC spectrum of **3o**

^{1}H NMR (400 MHz, room temperature, $CDCl_3$)

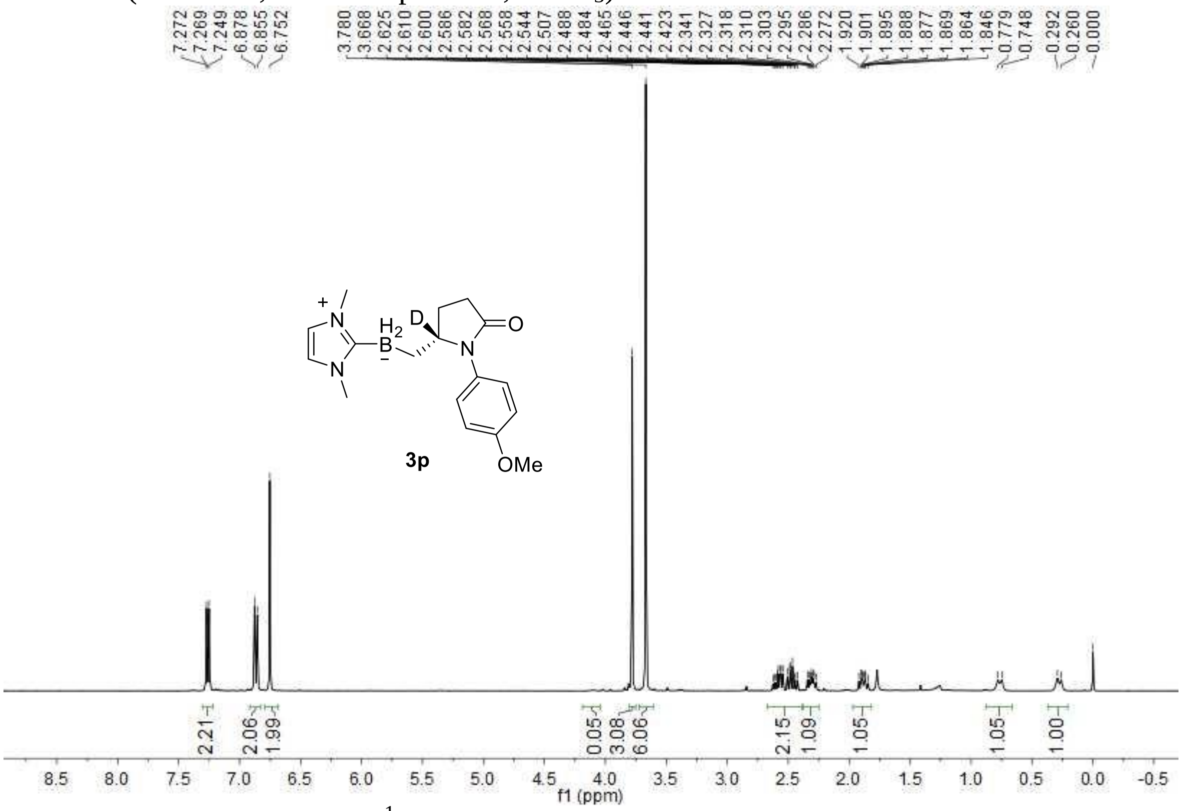

Supplementary Figure 125. ^{1}H NMR spectrum of compound **3p**

^{13}C NMR (100 MHz, room temperature, $CDCl_3$)

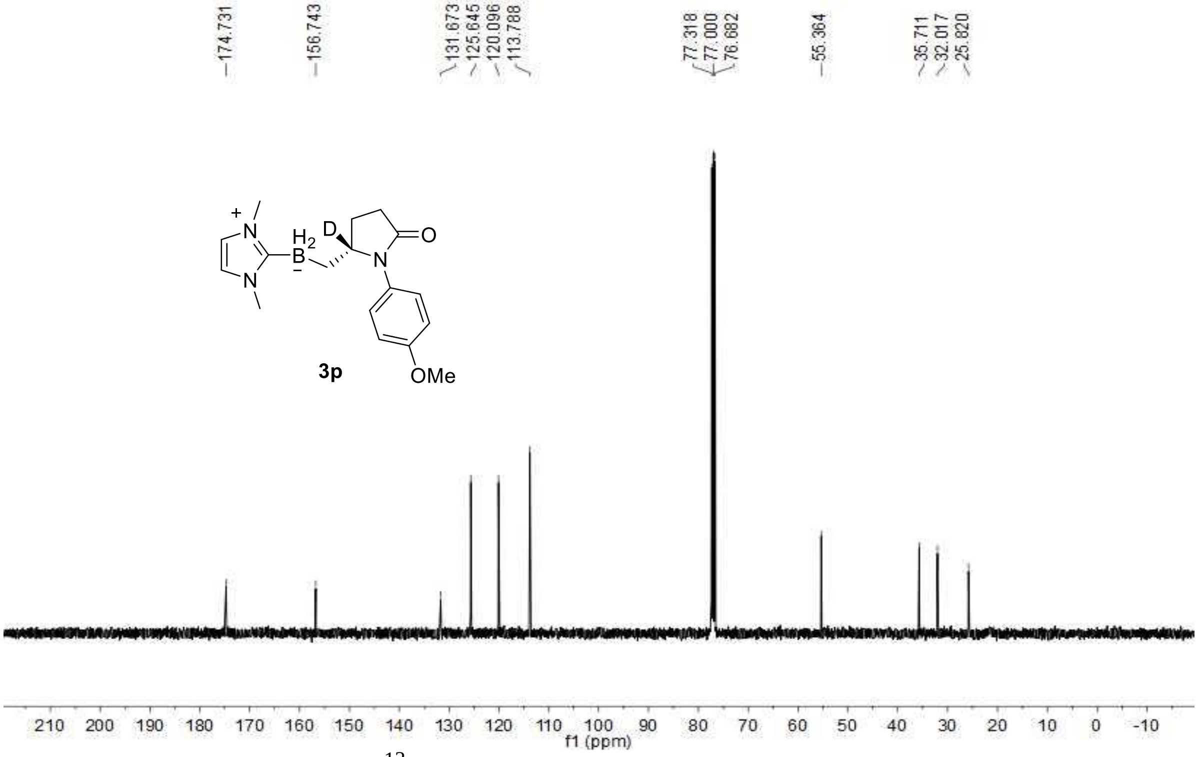

Supplementary Figure 126. ^{13}C NMR spectrum of compound **3p**

^{11}B NMR (128 MHz, room temperature, $CDCl_3$)

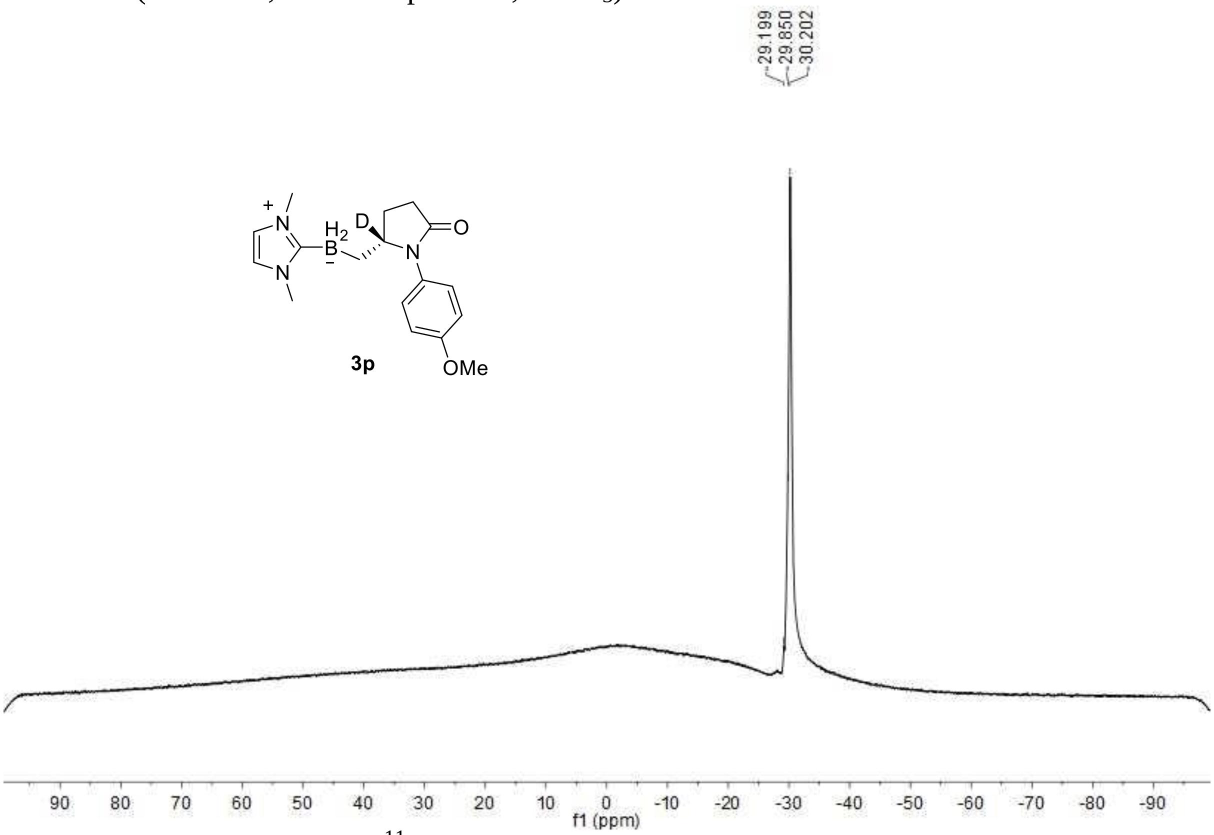

Supplementary Figure 127. ^{11}B NMR spectrum of compound **3p**

HPLC spectrum of racemic **3p**

Tray#	: 1
Vial#	: 14
Data File	: sql-6-399-1-(13).lcd
Method File	: 3AD-H-60-0.5-214.lcm
Date Acquired	: 1/25/2022 4:38:17 AM
Date Processed	: 1/25/2022 8:52:12 AM

<Chromatogram View>

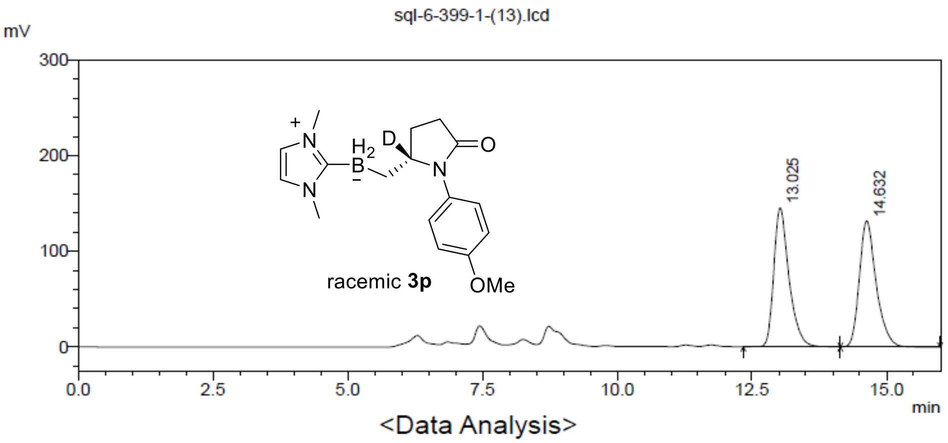

<Data Analysis>

Detector A 214nm

Pesk #	Ret. Time	Height	Area	Area%
1	13.025	145609	2889270	50.093
2	14.632	132127	2878585	49.907
Total		277736	5767855	100.000

Supplementary Figure 128. HPLC spectrum of racemic **3p**

HPLC spectrum of **3p**

Tray#	: 1
Vial#	: 15
Data File	: sql-7-20-3-(13).lcd
Method File	: 3AD-H-60-0.5-214.lcm
Date Acquired	: 1/25/2022 4:57:24 AM
Date Processed	: 1/25/2022 8:51:38 AM

<Chromatogram View>

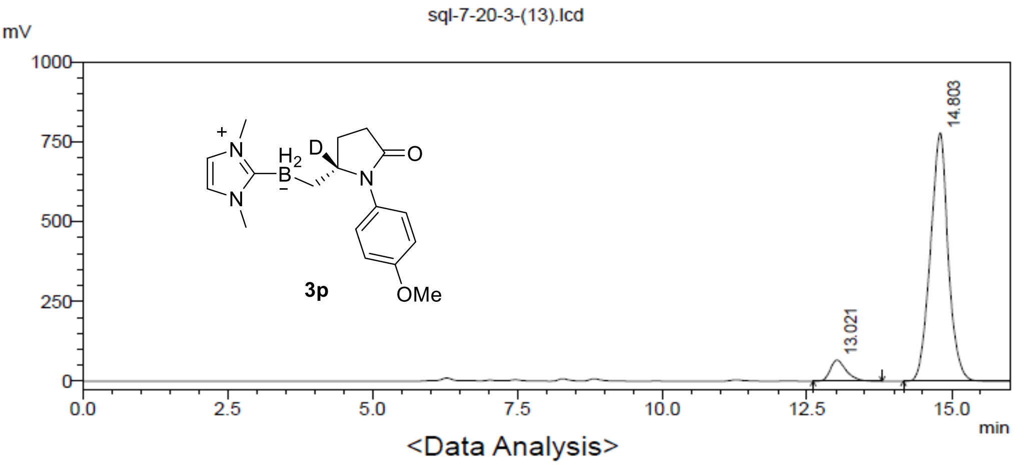

<Data Analysis>

Detector A 214nm

Pesk #	Ret. Time	Height	Area	Area%
1	13.021	66563	1292331	7.216
2	14.803	778007	16617160	92.784
Total		844570	17909491	100.000

Supplementary Figure 129. HPLC spectrum of **3p**

^{1}H NMR (400 MHz, room temperature, $CDCl_3$)

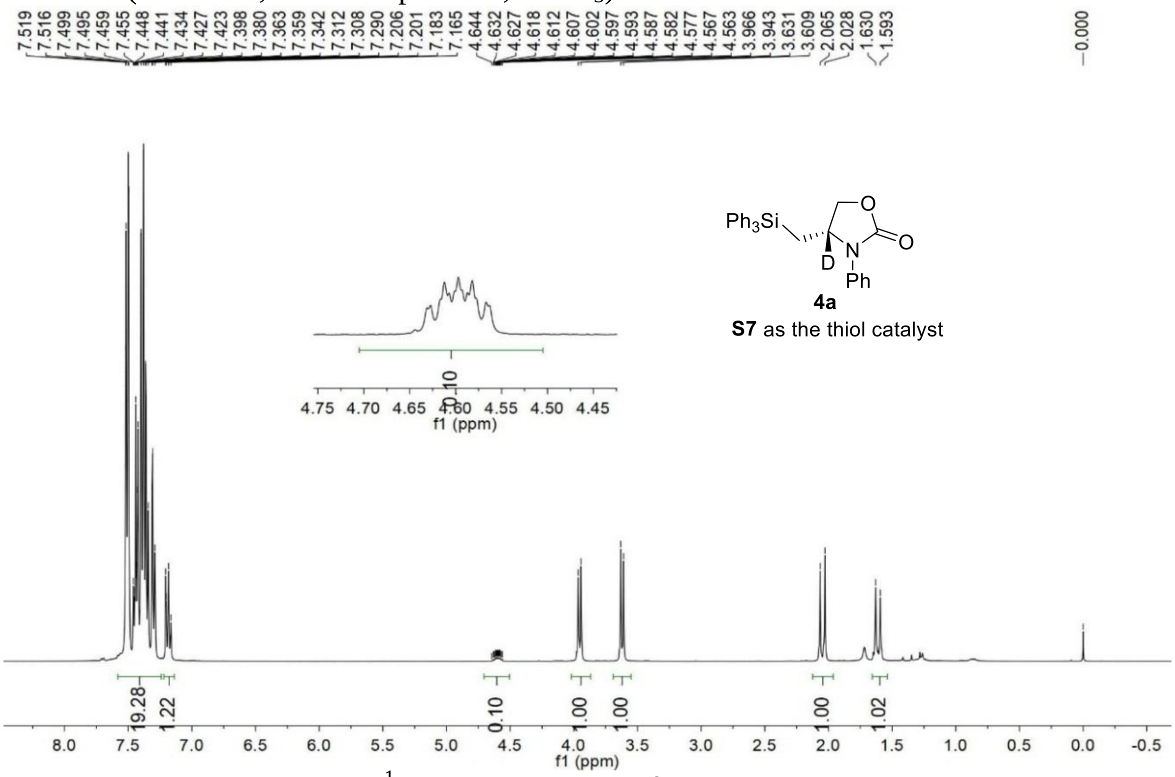

Supplementary Figure 130. ^{1}H NMR spectrum of compound **4a** (**S7** as the thiol catalyst)

^{1}H NMR (400 MHz, room temperature, $CDCl_3$)

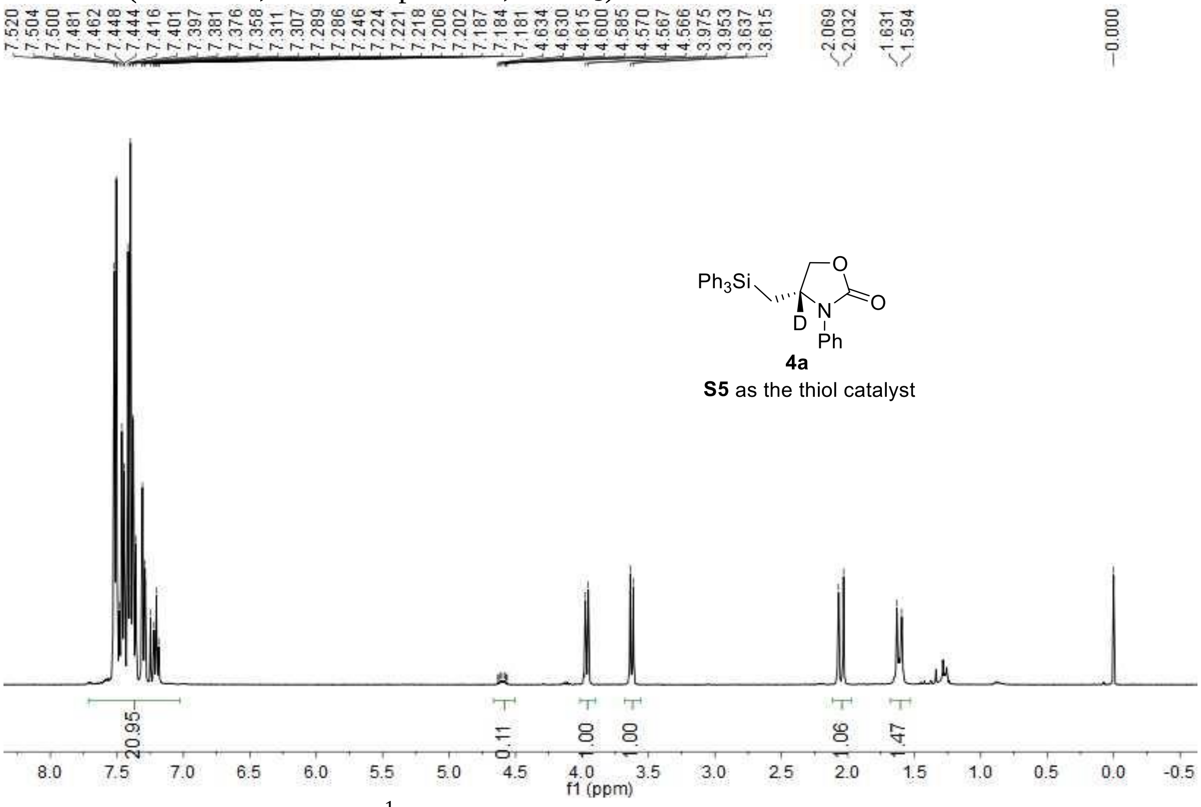

Supplementary Figure 131. ^{1}H NMR spectrum of compound **4a** (**S5** as the thiol catalyst)

^{1}H NMR (400 MHz, room temperature, $CDCl_3$)

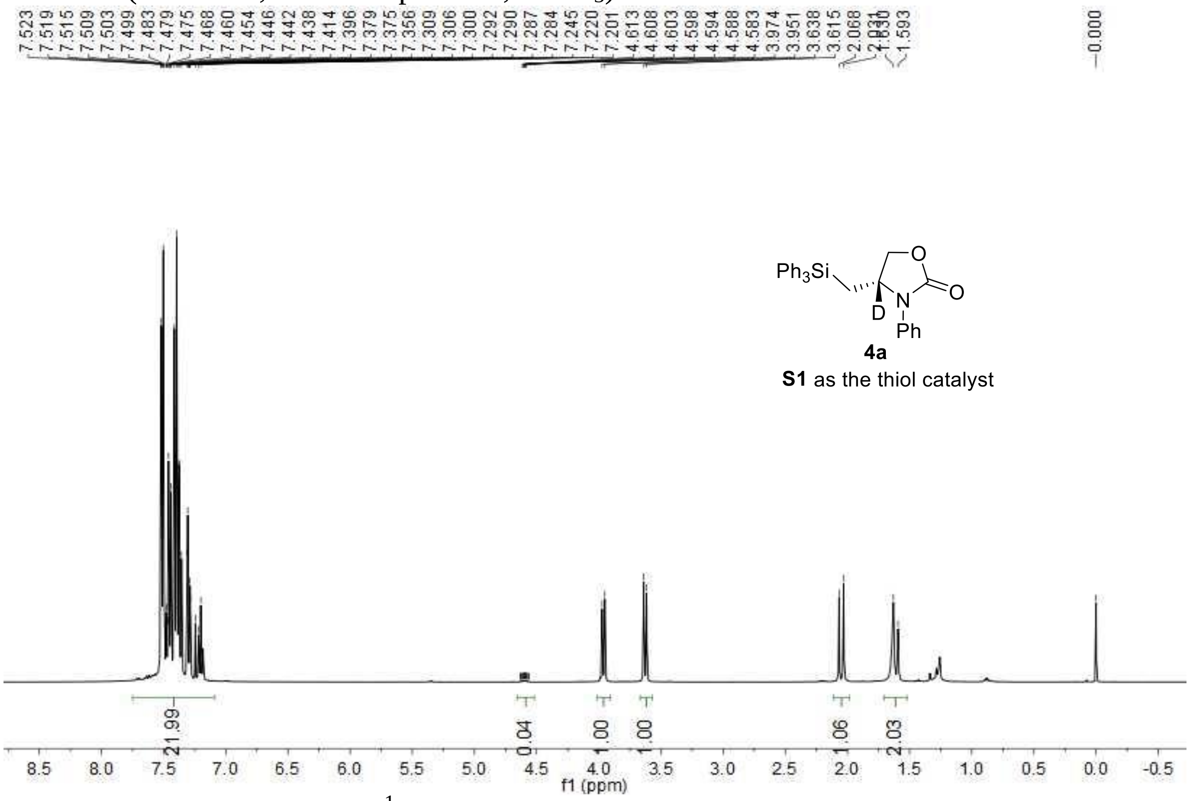

Supplementary Figure 132. ^{1}H NMR spectrum of compound **4a** (**S1** as the thiol catalyst)

^{13}C NMR (100 MHz, room temperature, $CDCl_3$)

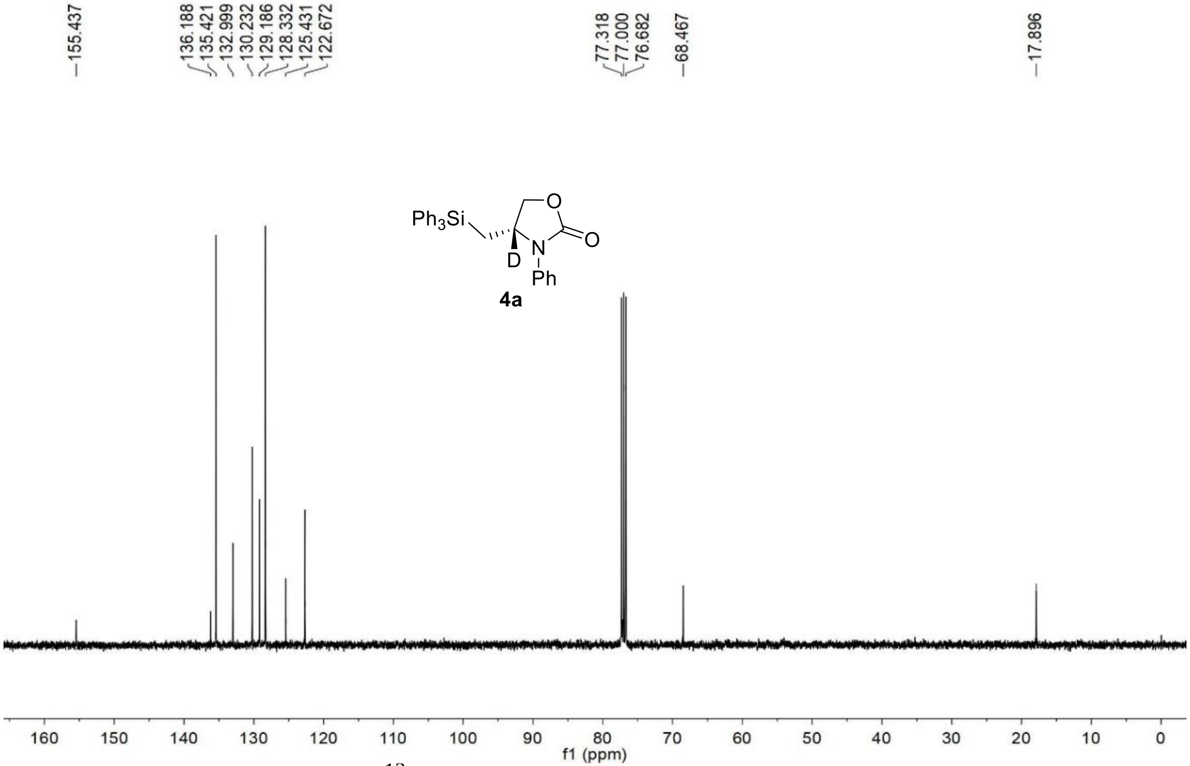

Supplementary Figure 133. ^{13}C NMR spectrum of compound **4a**

HPLC spectrum of racemic **4a**

Tray#	: 1
Vial#	: 46
Data File	: sql-5-320-1.lcd
Method File	: 3AD-H-90-1-214.lcm
Date Acquired	: 2/25/2022 9:19:32 PM
Date Processed	: 2/25/2022 11:04:00 PM

<Chromatogram View>

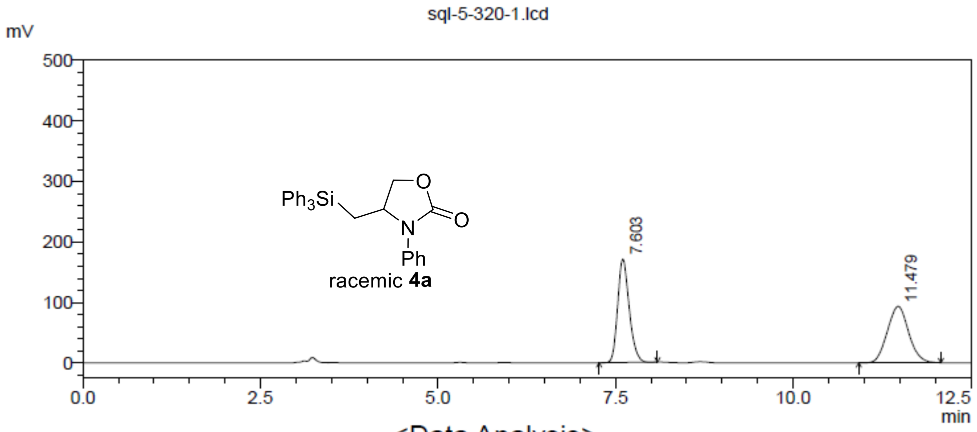

<Data Analysis>

Detector A 214nm

Pesk #	Ret. Time	Height	Area	Area%
1	7.603	170746	1930467	49.802
2	11.479	93099	1945791	50.198
Total		263846	3876258	100.000

Supplementary Figure 134. HPLC spectrum of racemic **4a**

HPLC spectrum of **4a** (**S7** as the thiol catalyst)

Sample Name	:
Tray#	: 1
Vial#	: 47
Data File	: sql-6-349-3.lcd
Method File	: 3AD-H-90-1-214.lcm
Date Acquired	: 2/25/2022 9:34:52 PM
Date Processed	: 2/25/2022 11:04:17 PM

<Chromatogram View>

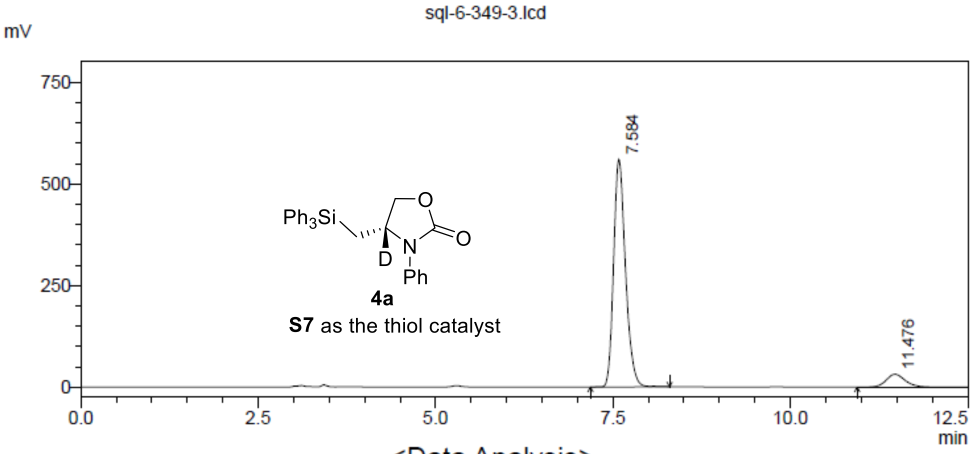

<Data Analysis>

Detector A 214nm

Pesk #	Ret. Time	Height	Area	Area%
1	7.584	559691	6514632	91.624
2	11.476	31839	595547	8.376
Total		591530	7110179	100.000

Supplementary Figure 135. HPLC spectrum of **4a** (**S7** as the thiol catalyst)

HPLC spectrum of **4a** (**S5** as the thiol catalyst)

Sample Name	:
Tray#	: 1
Vial#	: 48
Data File	: sql-7-82-1.lcd
Method File	: 3AD-H-90-1-214.lcm
Date Acquired	: 2/25/2022 11:05:43 PM
Date Processed	: 2/25/2022 11:22:57 PM

<Chromatogram View>

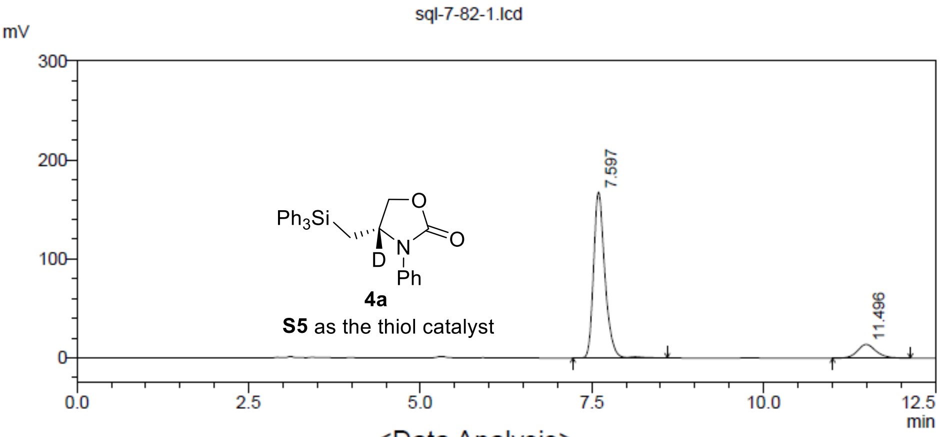

<Data Analysis>

Detector A 214nm

Pesk #	Ret. Time	Height	Area	Area%
1	7.597	167514	1925298	88.010
2	11.496	13904	262281	11.990
Total		181417	2187579	100.000

Supplementary Figure 136. HPLC spectrum of **4a** (**S5** as the thiol catalyst)

HPLC spectrum of **4a** (**S1** as the thiol catalyst)

Sample Name	:
Tray#	: 1
Vial#	: 13
Data File	: SQL-7-15.lcd
Method File	: 3AD-H-90-1-214.lcm
Date Acquired	: 1/23/2022 3:29:41 PM
Date Processed	: 2/28/2022 10:25:34 AM

<Chromatogram View>

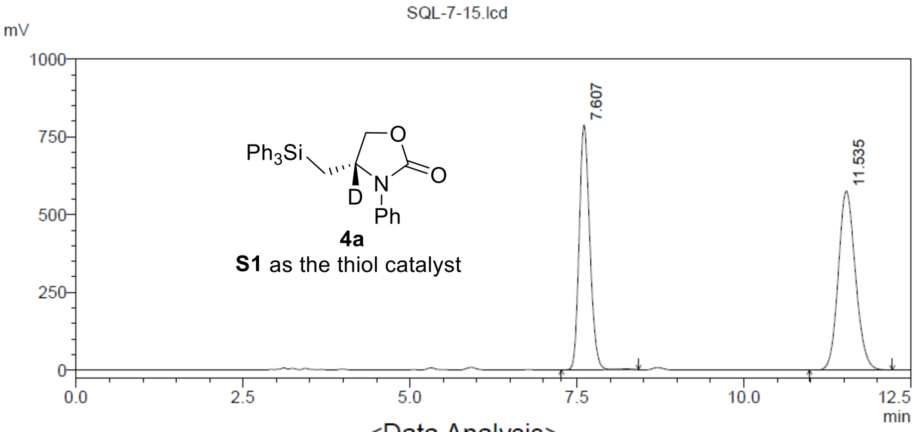

<Data Analysis>

Detector A 214nm

Pesk #	Ret. Time	Height	Area	Area%
1	7.607	787175	8714383	45.855
2	11.535	575690	10289857	54.145
Total		1362864	19004240	100.000

Supplementary Figure 137. HPLC spectrum of **4a** (**S1** as the thiol catalyst)

^{1}H NMR (400 MHz, room temperature, $CDCl_3$)

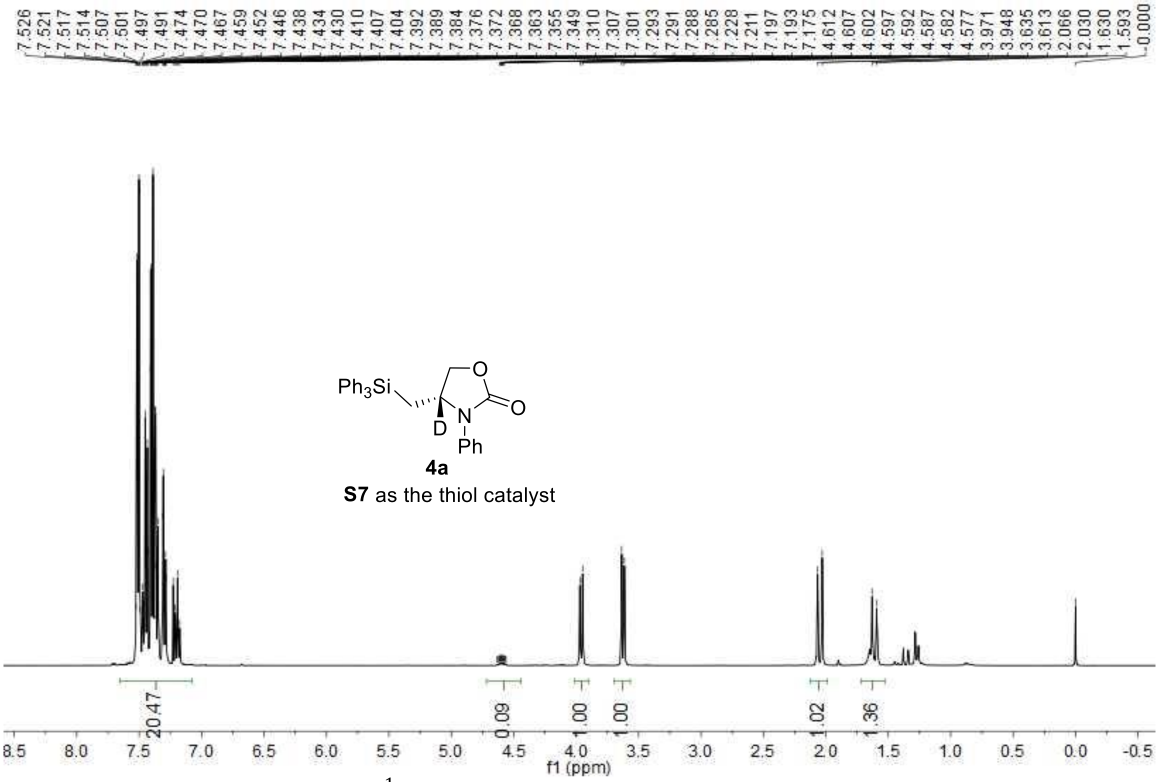

Supplementary Figure 138. ^{1}H NMR spectrum of compound **4a** (gram-scale reaction)

HPLC spectrum of racemic **4a**

Tray#	: 1
Vial#	: 46
Data File	: sql-5-320-1.lcd
Method File	: 3AD-H-90-1-214.lcm
Date Acquired	: 2/25/2022 9:19:32 PM
Date Processed	: 2/25/2022 11:04:00 PM

<Chromatogram View>

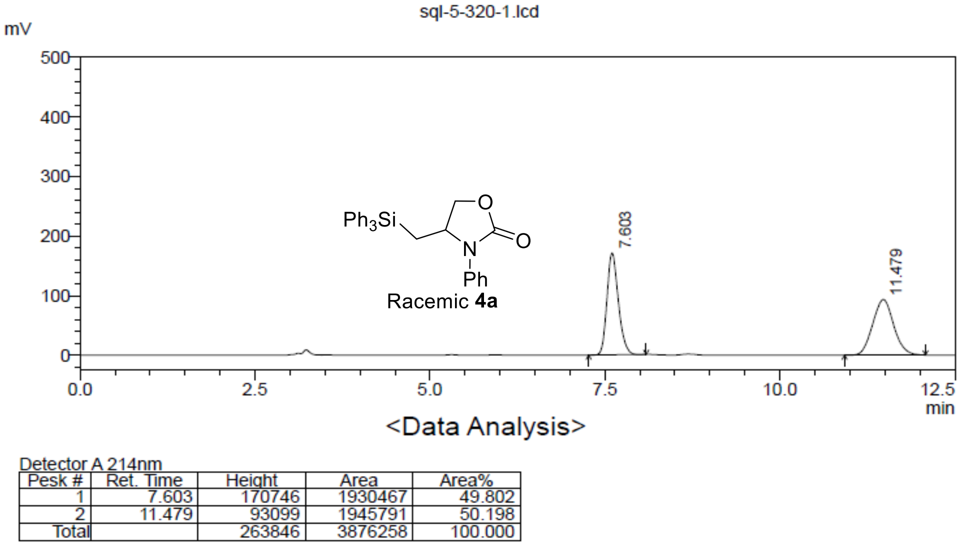

<Data Analysis>

Detector A 214nm

Pesk #	Ret. Time	Height	Area	Area%
1	7.603	170746	1930467	49.802
2	11.479	93099	1945791	50.198
Total		263846	3876258	100.000

Supplementary Figure 139. HPLC spectrum of racemic **4a**

HPLC spectrum of **4a** (gram-scale reaction)

Vial#	: 4
Data File	: YXY-3-63.lcd
Method File	: 3AD-H-90-1-214.lcm
Date Acquired	: 6/8/2022 11:08:25 PM
Date Processed	: 6/8/2022 11:44:09 PM

<Chromatogram View>

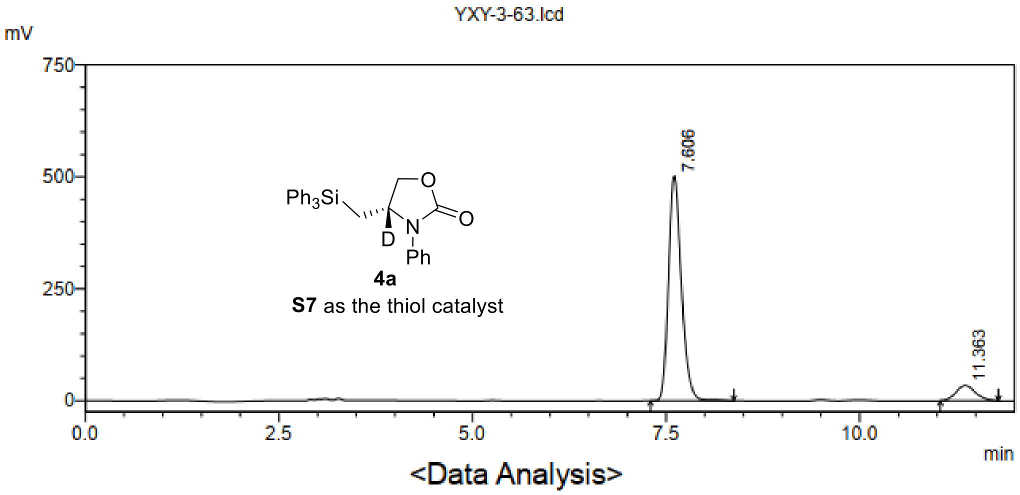

<Data Analysis>

Detector A 214nm

Pesk #	Ret. Time	Height	Area	Area%
1	7.606	500866	5797583	90.628
2	11.363	34056	599507	9.372
Total		534922	6397091	100.000

Supplementary Figure 140. HPLC spectrum of **4a** (gram-scale reaction)

^{1}H NMR (400 MHz, room temperature, $CDCl_3$)

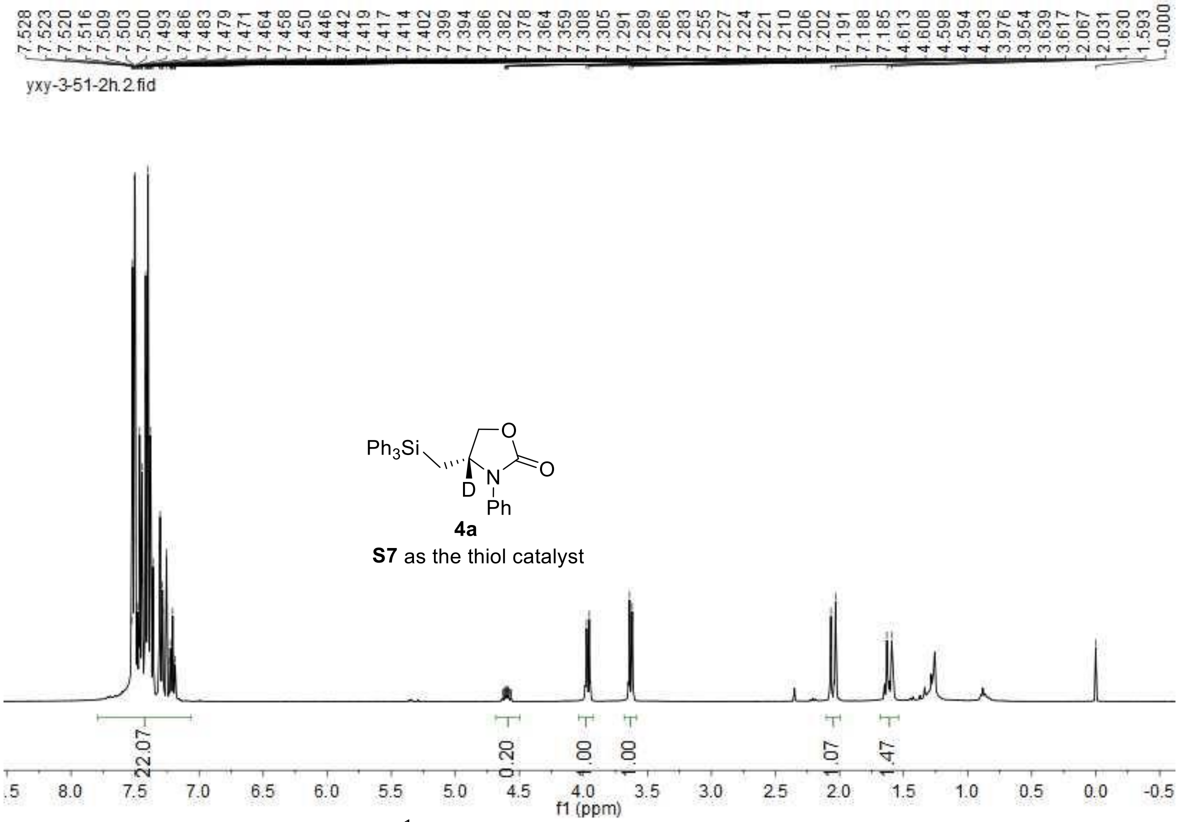

Supplementary Figure 141. ^{1}H NMR spectrum of compound **4a** (D_2O recycled from a 0.6 mmol scale reaction)

HPLC spectrum of racemic **4a**

Tray#	: 1
Vial#	: 46
Data File	: sql-5-320-1.lcd
Method File	: 3AD-H-90-1-214.lcm
Date Acquired	: 2/25/2022 9:19:32 PM
Date Processed	: 2/25/2022 11:04:00 PM

<Chromatogram View>

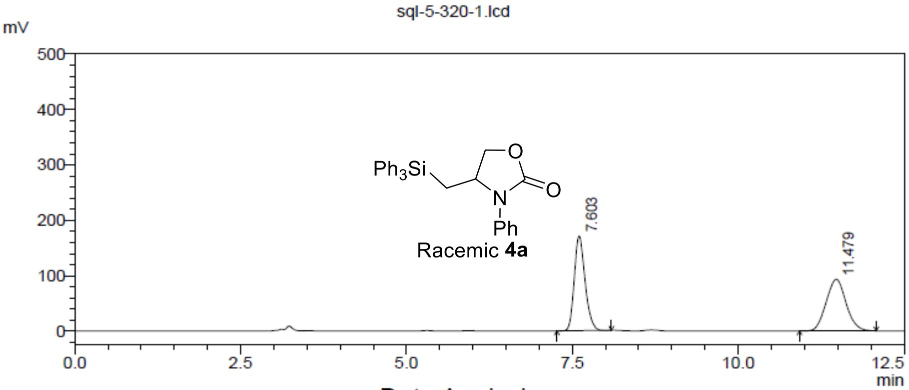

<Data Analysis>

Detector A 214nm

Pesk #	Ret. Time	Height	Area	Area%
1	7.603	170746	1930467	49.802
2	11.479	93099	1945791	50.198
Total		263846	3876258	100.000

Supplementary Figure 142. HPLC spectrum of racemic **4a**

HPLC spectrum of **4a** (D_2O recycled from a 0.6 mmol scale reaction)

Vial#	: 1
Data File	: YXY-3-51-3.lcd
Method File	: 3AD-H-90-1-214.lcm
Date Acquired	: 5/29/2022 10:16:09 PM
Date Processed	: 5/29/2022 10:28:38 PM

<Chromatogram View>

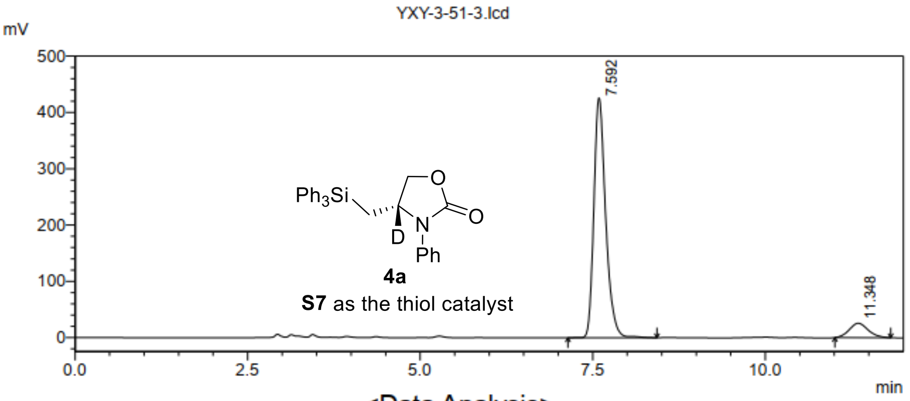

<Data Analysis>

Detector A 214nm

Pesk #	Ret. Time	Height	Area	Area%
1	7.592	426414	5081760	91.247
2	11.348	25571	487469	8.753
Total		451985	5569229	100.000

Supplementary Figure 143. HPLC spectrum of **4a** (D_2O recycled from a 0.6 mmol scale reaction)

^1H NMR (400 MHz, room temperature, $CDCl_3$)

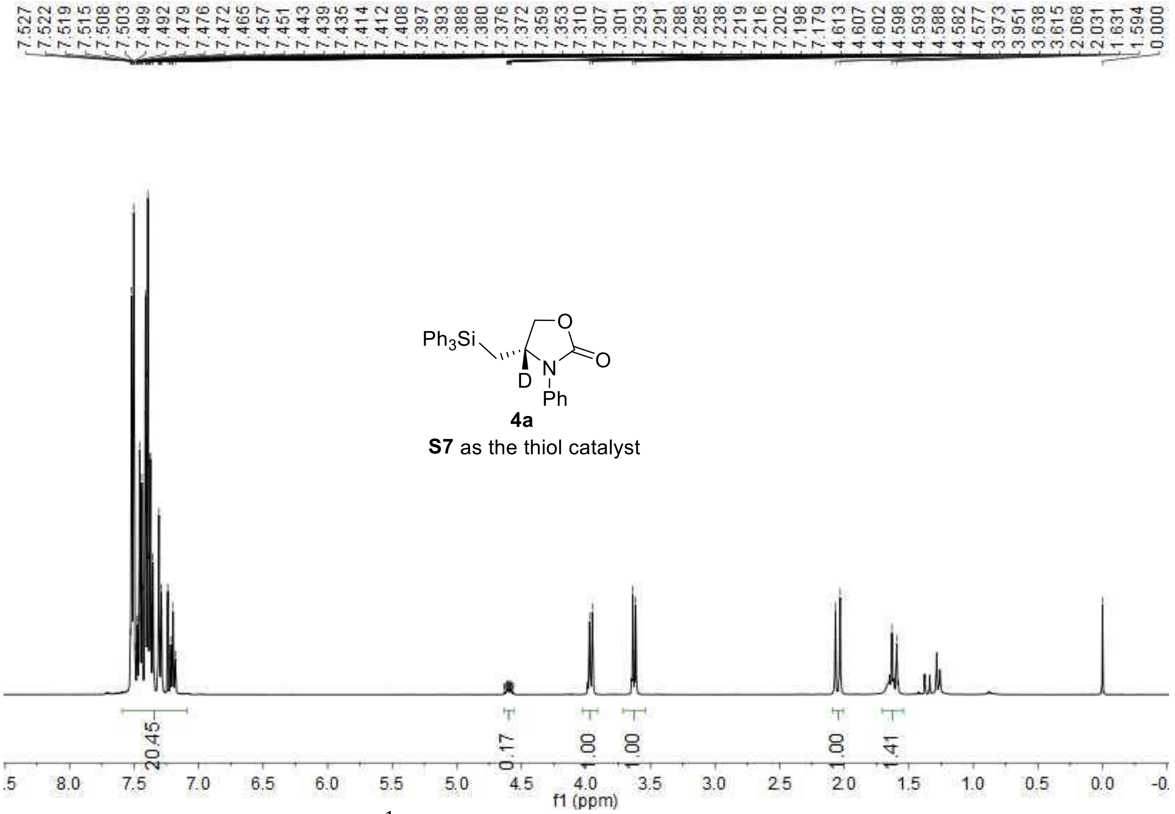

Supplementary Figure 144. ^1H NMR spectrum of compound **4a** (D_2O recycled from a 3.0 mmol scale reaction)

HPLC spectrum of racemic **4a**

Tray#	: 1
Vial#	: 46
Data File	: sql-5-320-1.lcd
Method File	: 3AD-H-90-1-214.lcm
Date Acquired	: 2/25/2022 9:19:32 PM
Date Processed	: 2/25/2022 11:04:00 PM

<Chromatogram View>

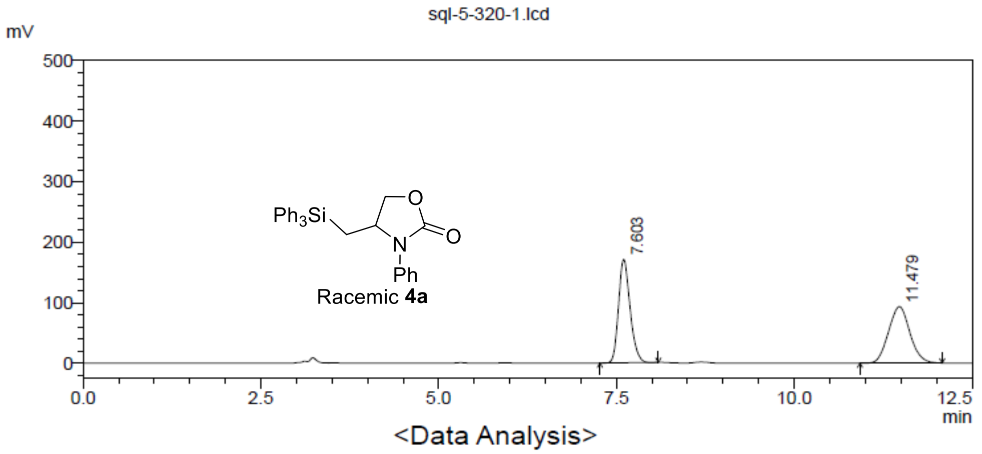

<Data Analysis>

Detector A 214nm

Pesk #	Ret. Time	Height	Area	Area%
1	7.603	170746	1930467	49.802
2	11.479	93099	1945791	50.198
Total		263846	3876258	100.000

Supplementary Figure 145. HPLC spectrum of racemic **4a**

HPLC spectrum of **4a** (D_2O recycled from a 3.0 mmol scale reaction)

Vial#	: 30
Data File	: sql-7-151-2.lcd
Method File	: 3AD-H-90-1-214.lcm
Date Acquired	: 6/16/2022 6:28:30 PM
Date Processed	: 6/16/2022 8:47:33 PM

<Chromatogram View>

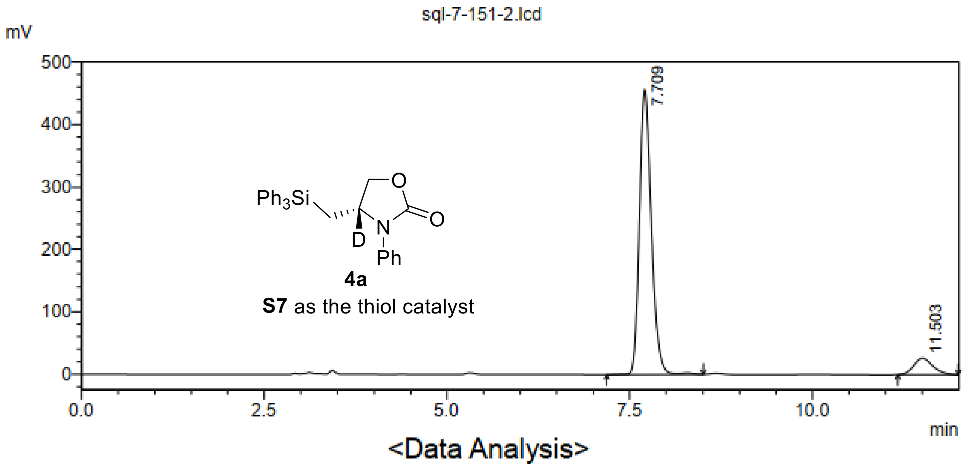

<Data Analysis>

Detector A 214nm

Pesk #	Ret. Time	Height	Area	Area%
1	7.709	455265	5151763	91.536
2	11.503	26354	476381	8.464
Total		481620	5628145	100.000

Supplementary Figure 146. HPLC spectrum of **4a** (D_2O recycled from a 3.0 mmol scale reaction)

^{1}H NMR (400 MHz, room temperature, $CDCl_3$)

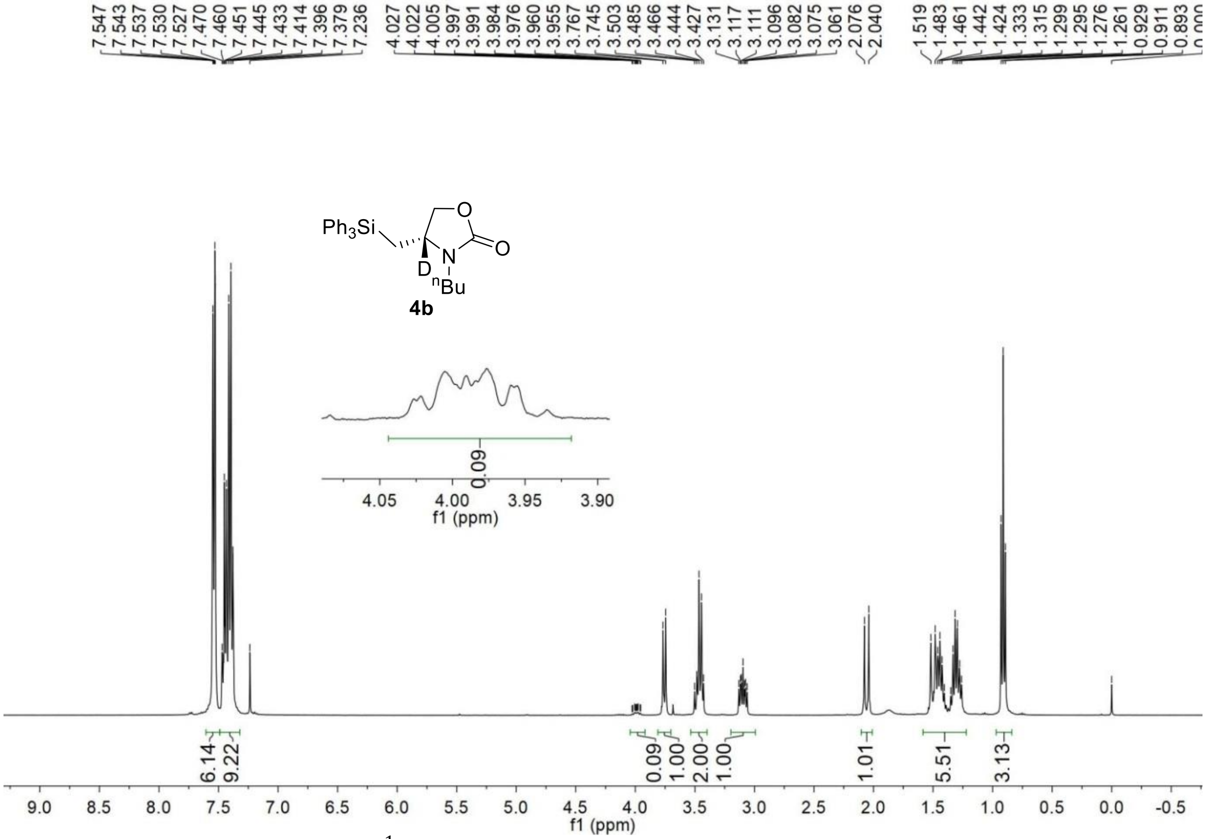

Supplementary Figure 147. ^{1}H NMR spectrum of compound **4b**

^{13}C NMR (100 MHz, room temperature, $CDCl_3$)

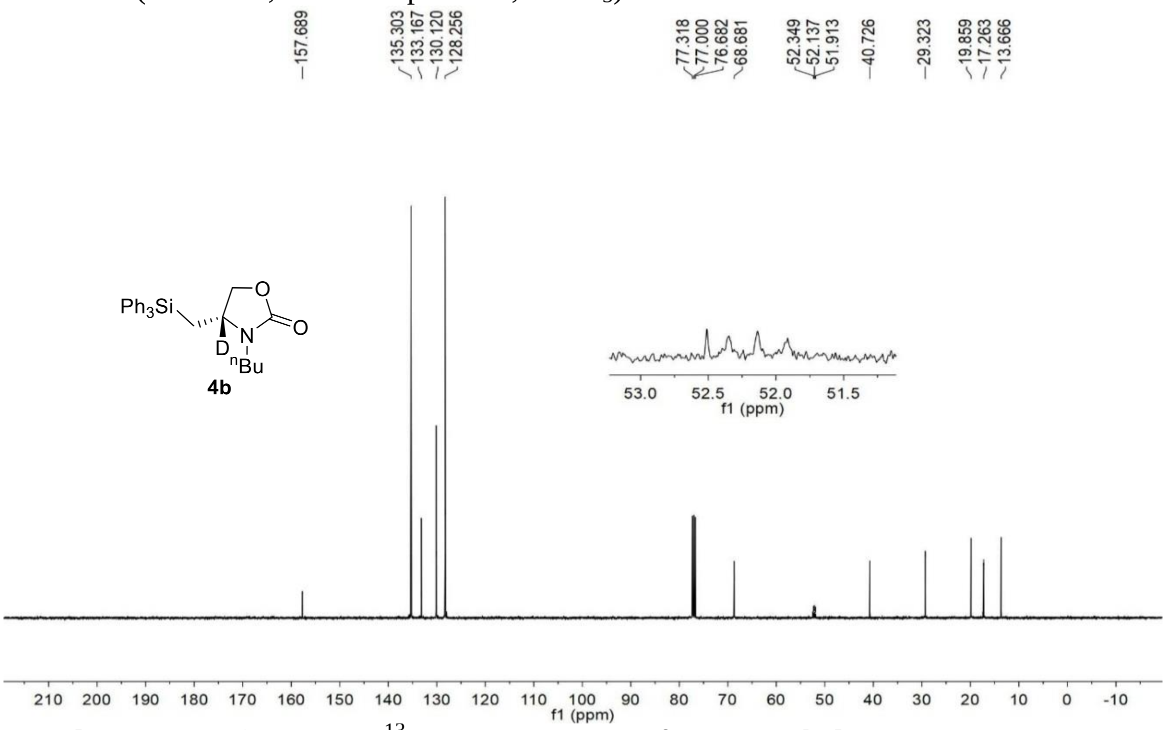

Supplementary Figure 148. ^{13}C NMR spectrum of compound **4b**

HPLC spectrum of racemic **4b**

Data File	: 21-RACEMIC-SQL-5-379-1.lcd
Method File	: 3AD-H-96-1-214-20min.lcm
Date Processed	: 8/8/2021 12:13:54 PM

<Chromatogram View>

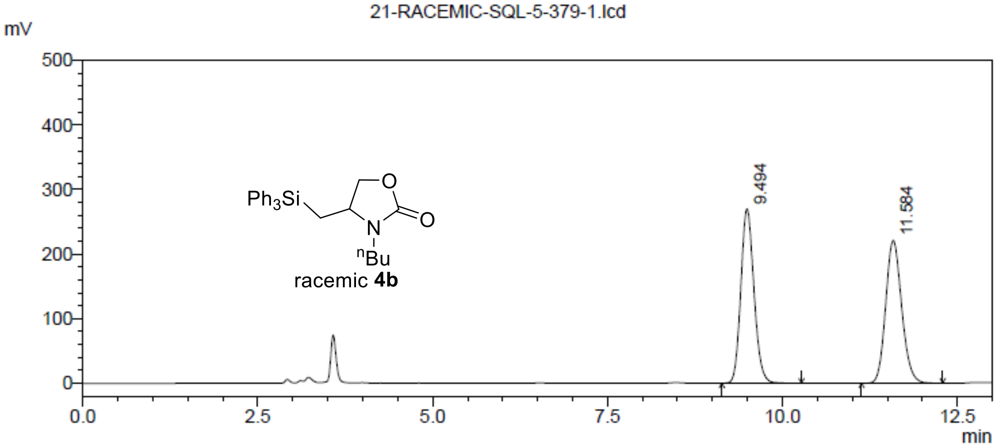

<Data Analysis>

Detector A 214nm

Pesk #	Ret. Time	Height	Area	Area%
1	9.494	269516	3515563	49.980
2	11.584	220736	3518428	50.020
Total		490252	7033991	100.000

Supplementary Figure 149. HPLC spectrum of racemic **4b**

HPLC spectrum of **4b**

Data File	: 21-CHIRAL-SQL-5-380.lcd
Method File	: 3AD-H-96-1-214-20min.lcm
Date Processed	: 8/8/2021 12:14:28 PM

<Chromatogram View>

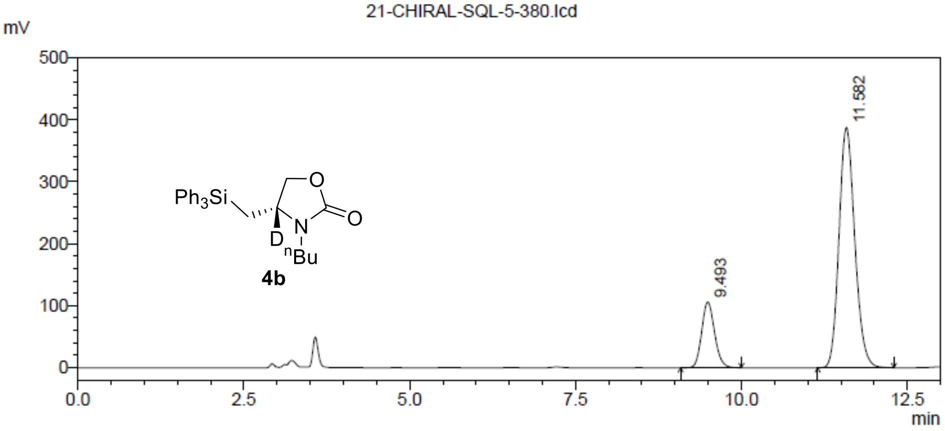

<Data Analysis>

Detector A 214nm

Pesk #	Ret. Time	Height	Area	Area%
1	9.493	106044	1432051	18.253
2	11.582	387117	6413398	81.747
Total		493161	7845449	100.000

Supplementary Figure 150. HPLC spectrum of **4b**

^{1}H NMR (400 MHz, room temperature, $CDCl_3$)

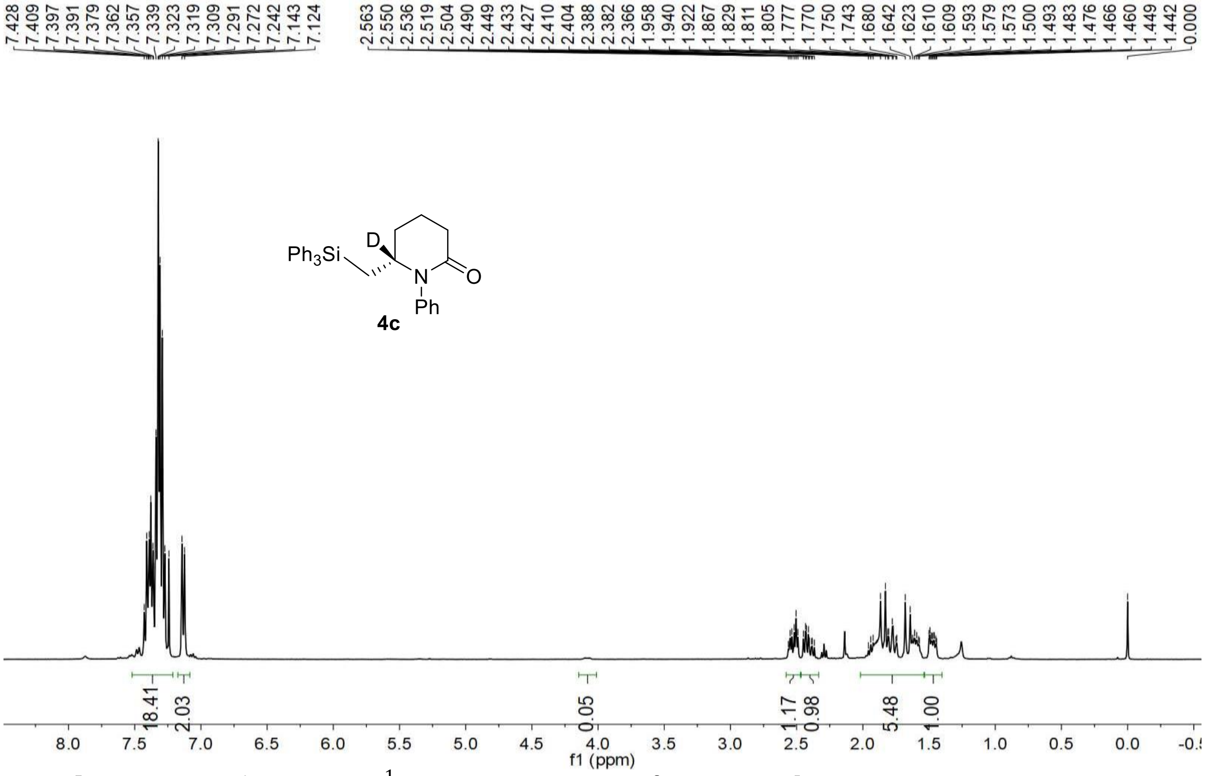

Supplementary Figure 151. ^{1}H NMR spectrum of compound **4c**

^{13}C NMR (100 MHz, room temperature, $CDCl_3$)

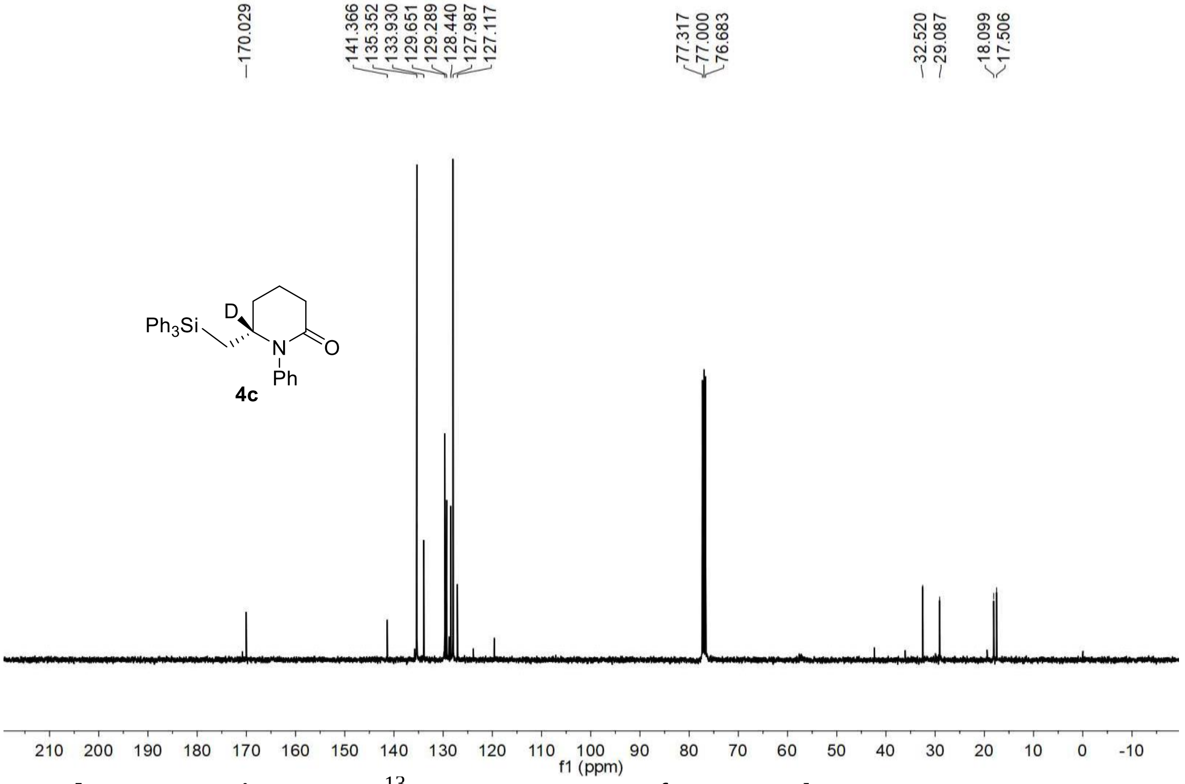

Supplementary Figure 152. ^{13}C NMR spectrum of compound **4c**

HPLC spectrum of racemic **4c**

Sample Name	:	
Tray#	:	1
Vial#	:	46
Data File	:	SQL-6-391-1-4.lcd
Method File	:	3AD-H-80-0.5-214.lcm
Date Acquired	:	1/24/2022 7:21:08 PM
Date Processed	:	1/24/2022 7:46:22 PM

<Chromatogram View>

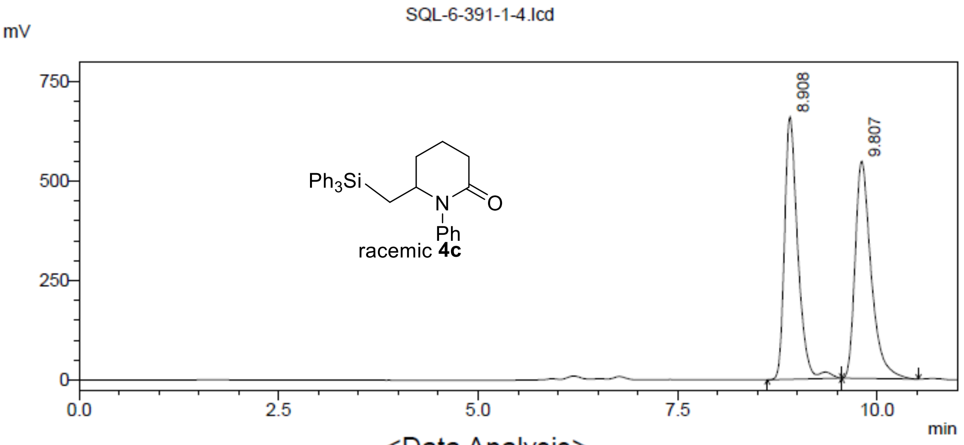

<Data Analysis>

Detector A 214nm

Pesk #	Ret. Time	Height	Area	Area%
1	8.908	659428	7613217	49.931
2	9.807	545984	7634394	50.069
Total		1205413	15247611	100.000

Supplementary Figure 153. HPLC spectrum of racemic **4c**

HPLC spectrum of **4c**

Sample Name	:	
Tray#	:	1
Vial#	:	47
Data File	:	SQL-7-35-1-1.lcd
Method File	:	3AD-H-80-0.5-214.lcm
Date Acquired	:	1/24/2022 7:47:15 PM
Date Processed	:	1/24/2022 8:12:39 PM

<Chromatogram View>

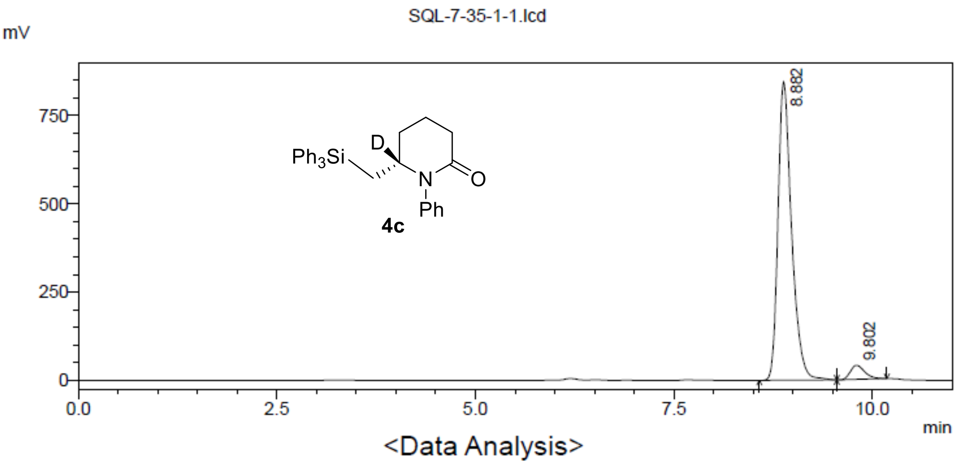

<Data Analysis>

Detector A 214nm

Pesk #	Ret. Time	Height	Area	Area%
1	8.882	845322	10063358	95.246
2	9.802	40029	502270	4.754
Total		885351	10565628	100.000

Supplementary Figure 154. HPLC spectrum of **4c**

^1H NMR (400 MHz, room temperature, $CDCl_3$)

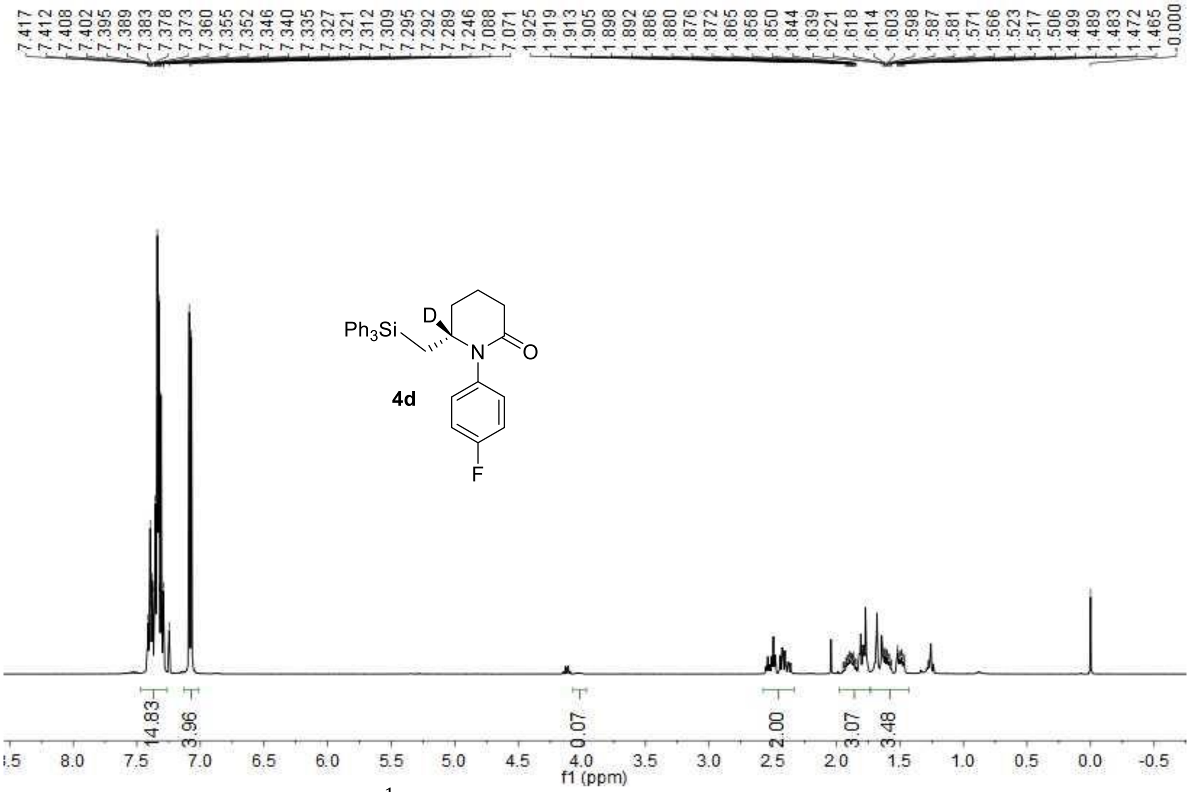

Supplementary Figure 155. ^1H NMR spectrum of compound **4d**

^{13}C NMR (100 MHz, room temperature, $CDCl_3$)

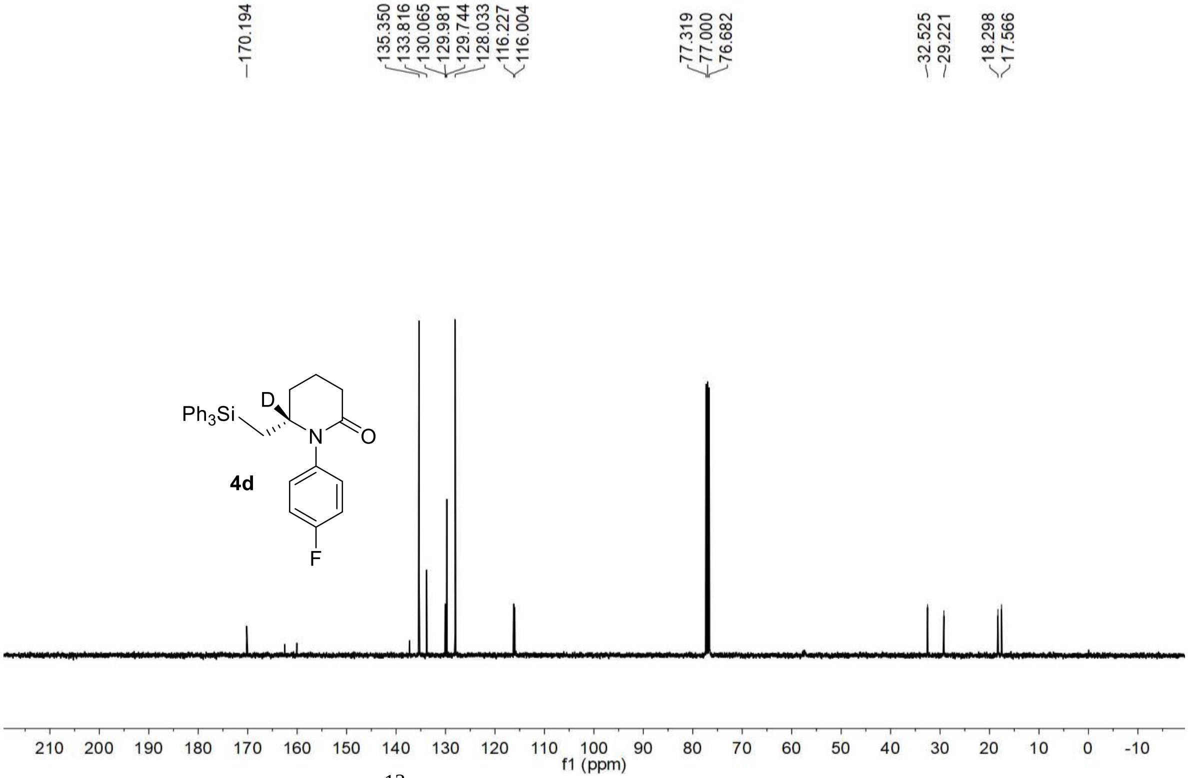

Supplementary Figure 156. ^{13}C NMR spectrum of compound **4d**

^{19}F NMR (376 MHz, room temperature, $CDCl_3$)

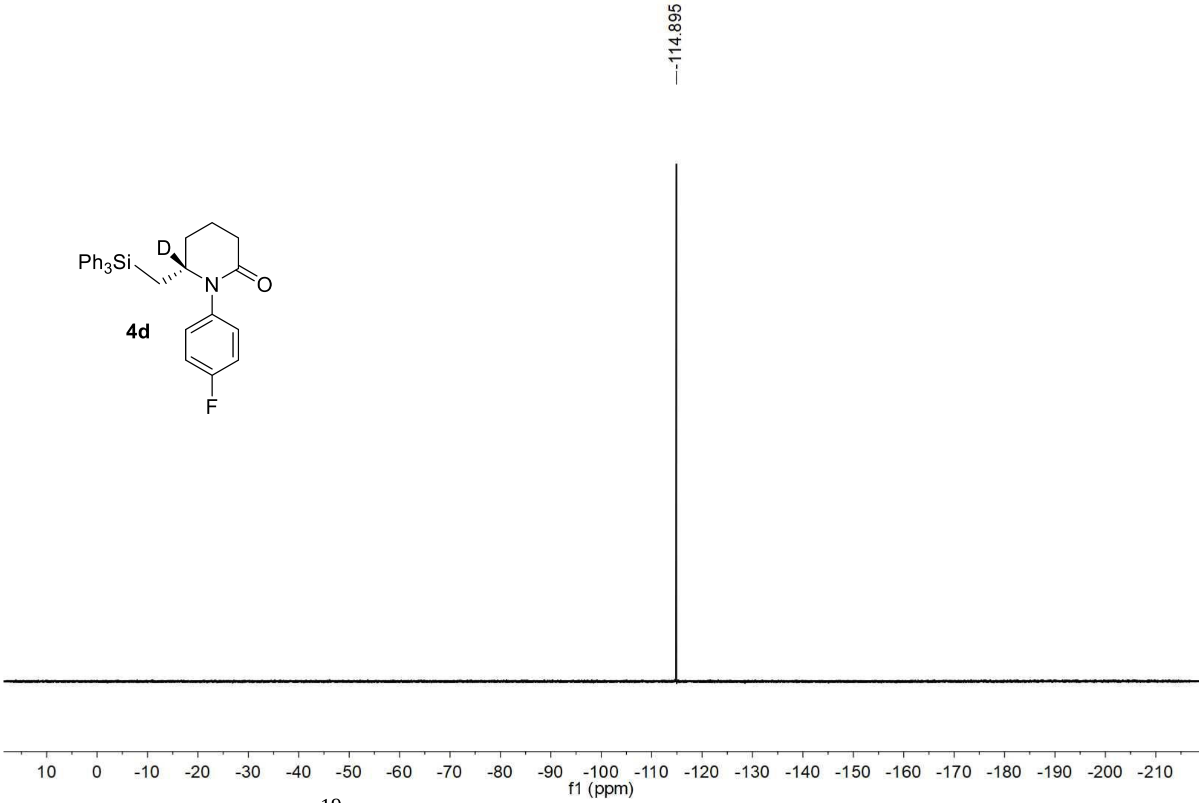

Supplementary Figure 157. ^{19}F NMR spectrum of compound **4d**

HPLC spectrum of racemic **4d**

Sample Name	:
Tray#	: 1
Vial#	: 20
Data File	: sql-7-72-1.lcd
Method File	: 4OD-H-80-0.5-214.lcm
Date Acquired	: 2/20/2022 11:00:16 PM
Date Processed	: 2/20/2022 11:32:56 PM

<Chromatogram View>

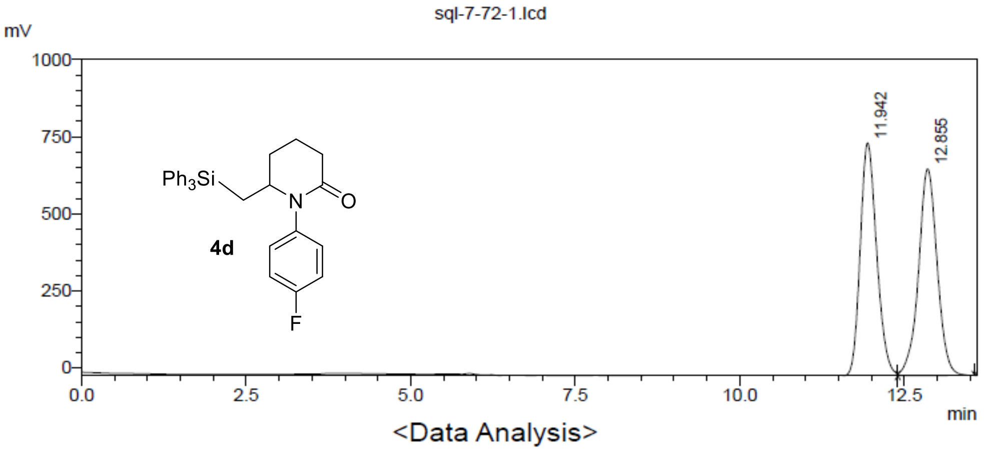

<Data Analysis>

Detector A 214nm

Pesk #	Ret. Time	Height	Area	Area%
1	11.942	755590	13124785	49.793
2	12.855	670987	13234119	50.207
Total		1426577	26358903	100.000

Supplementary Figure 158. HPLC spectrum of racemic **4d**

HPLC spectrum of **4d**

Sample Name :
Tray# : 1
Vial# : 20
Data File : sql-7-72-2.lcd
Method File : 4OD-H-80-0.5-214.lcm
Date Acquired : 2/20/2022 11:18:36 PM
Date Processed : 2/20/2022 11:32:54 PM

<Chromatogram View>

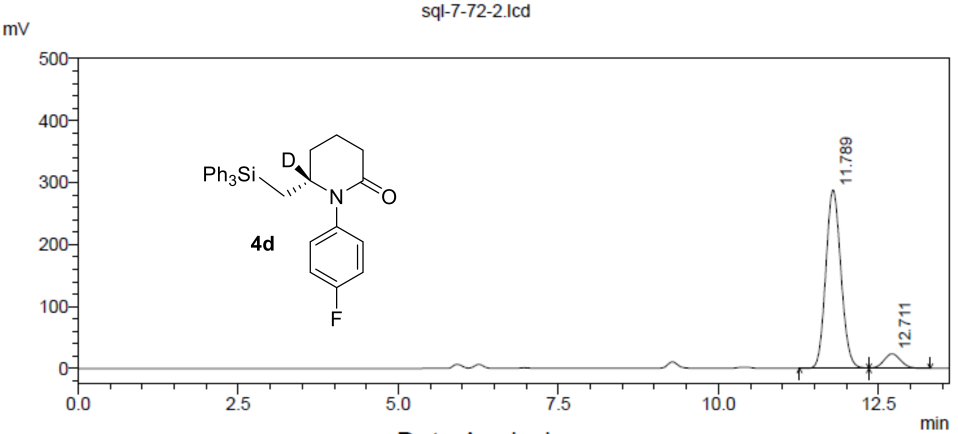

<Data Analysis>

Detector A 214nm

Pesk #	Ret. Time	Height	Area	Area%
1	11.789	287861	4734270	91.669
2	12.711	23476	430266	8.331
Total		311337	5164537	100.000

Supplementary Figure 159. HPLC spectrum of **4d**

^{1}H NMR (400 MHz, room temperature, $CDCl_3$)

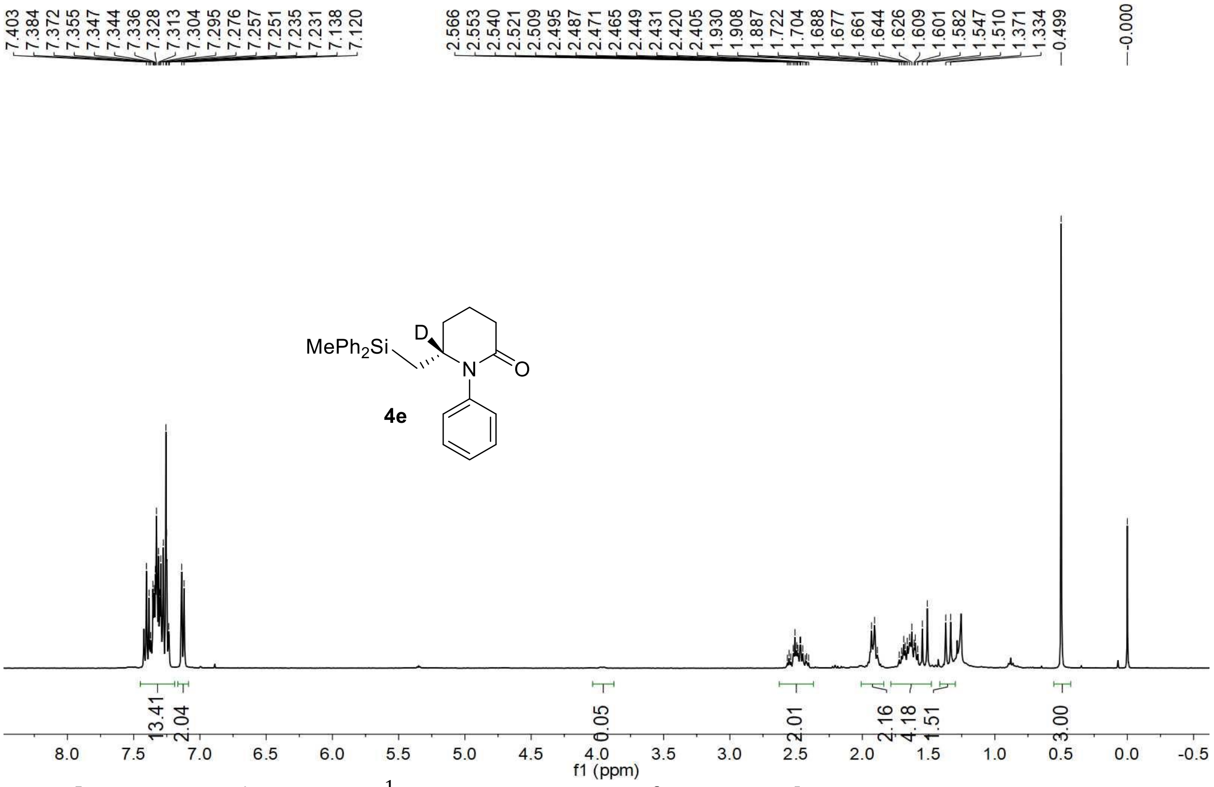

Supplementary Figure 160. ^{1}H NMR spectrum of compound **4e**

^{13}C NMR (100 MHz, room temperature, $CDCl_3$)

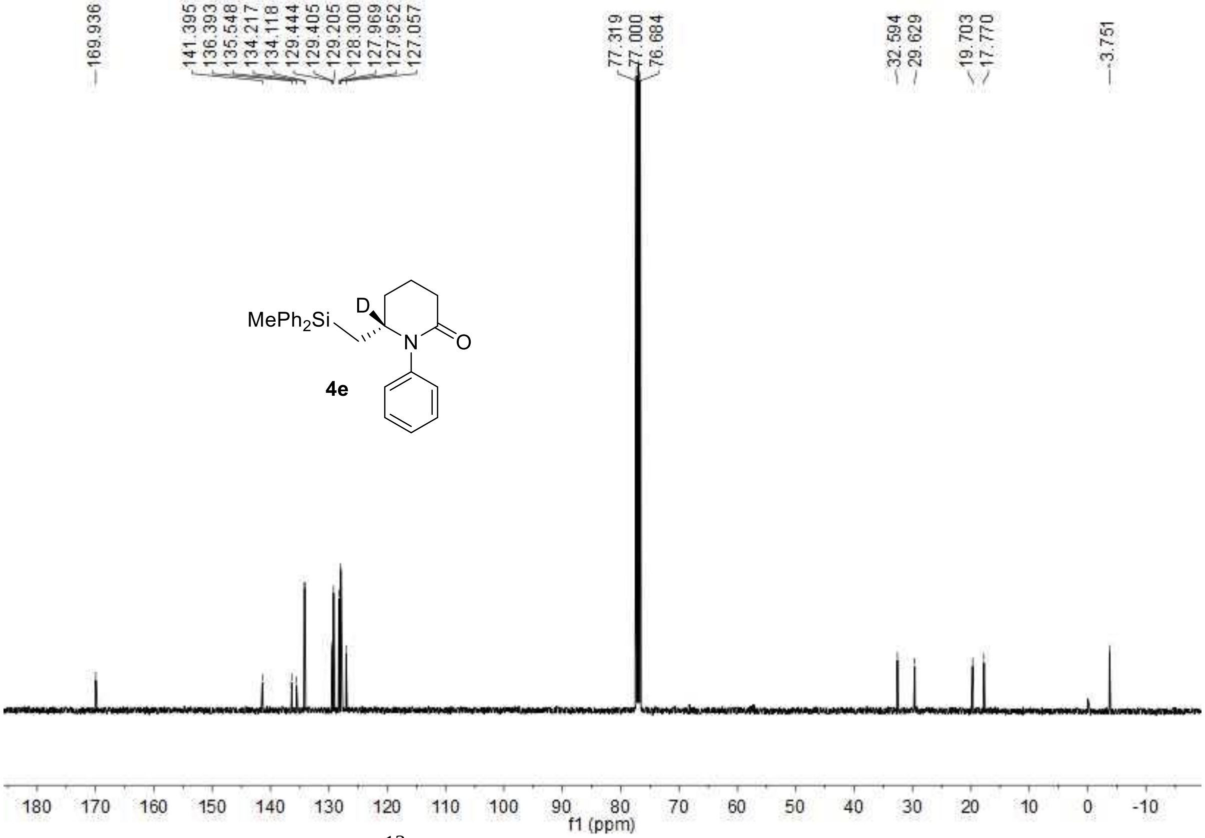

Supplementary Figure 161. ^{13}C NMR spectrum of compound **4e**

HPLC spectrum of racemic **4e**

Sample Name	:
Tray#	: 1
Vial#	: 28
Data File	: sql-7-59-1-8.lcd
Method File	: 4OD-H-90-0.5-214.lcm
Date Acquired	: 2/20/2022 12:03:21 AM
Date Processed	: 2/20/2022 2:44:47 PM

<Chromatogram View>

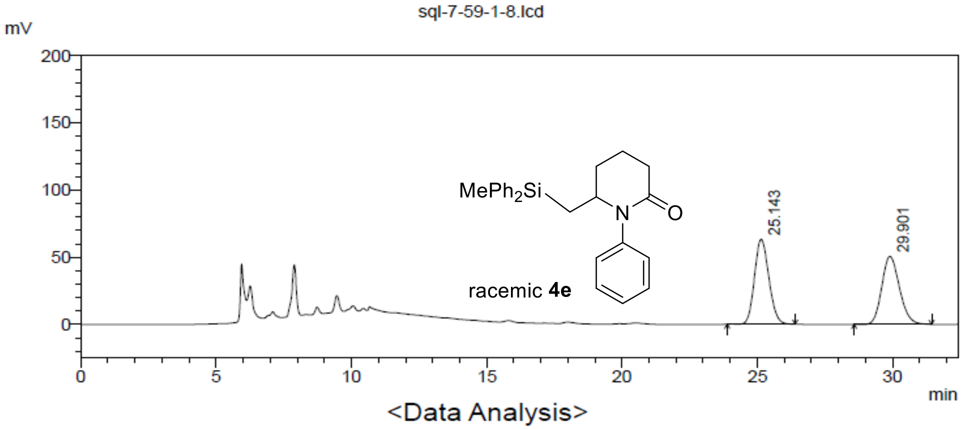

<Data Analysis>

Detector A 214nm

Pesk #	Ret. Time	Height	Area	Area%
1	25.143	63250	2336532	50.383
2	29.901	50652	2301045	49.617
Total		113901	4637577	100.000

Supplementary Figure 162. HPLC spectrum of racemic **4e**

HPLC spectrum of **4e**

Sample Name	:
Tray#	: 1
Vial#	: 30
Data File	: sql-7-71-2-4.lcd
Method File	: 4OD-H-90-0.5-214.lcm
Date Acquired	: 2/20/2022 12:54:26 AM
Date Processed	: 2/20/2022 7:19:19 PM

<Chromatogram View>

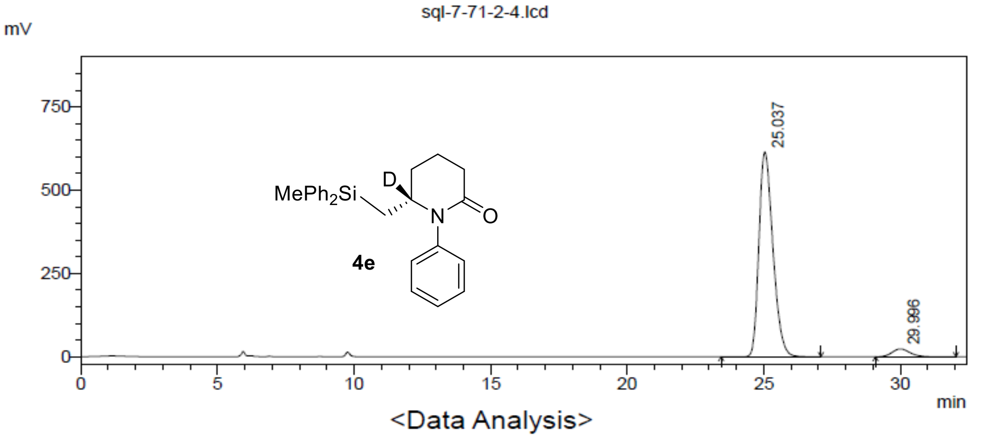

<Data Analysis>

Detector A 214nm

Pesk #	Ret. Time	Height	Area	Area%
1	25.037	615357	21893091	95.374
2	29.996	23471	1061837	4.626
Total		638829	22954928	100.000

Supplementary Figure 163. HPLC spectrum of **4e**

^{1}H NMR (400 MHz, room temperature, $CDCl_3$)

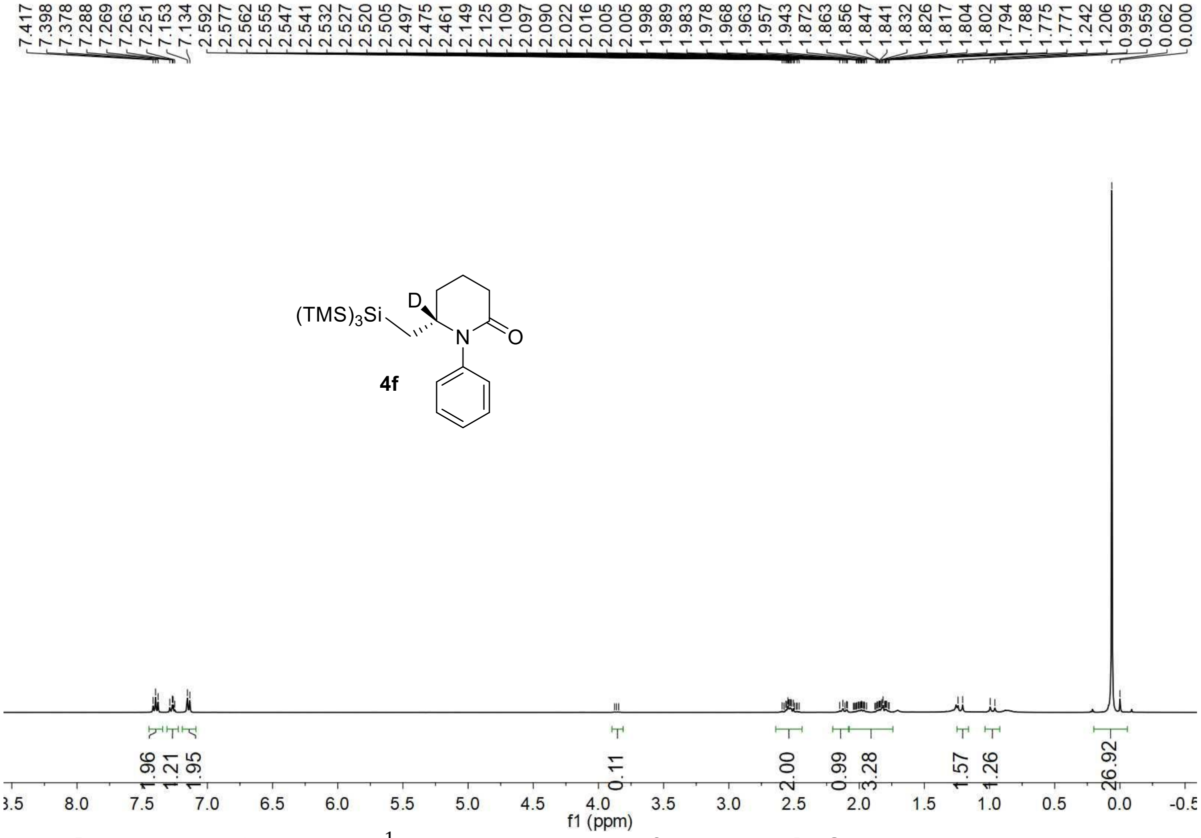

Supplementary Figure 164. ^{1}H NMR spectrum of compound **4f**

^{13}C NMR (100 MHz, room temperature, $CDCl_3$)

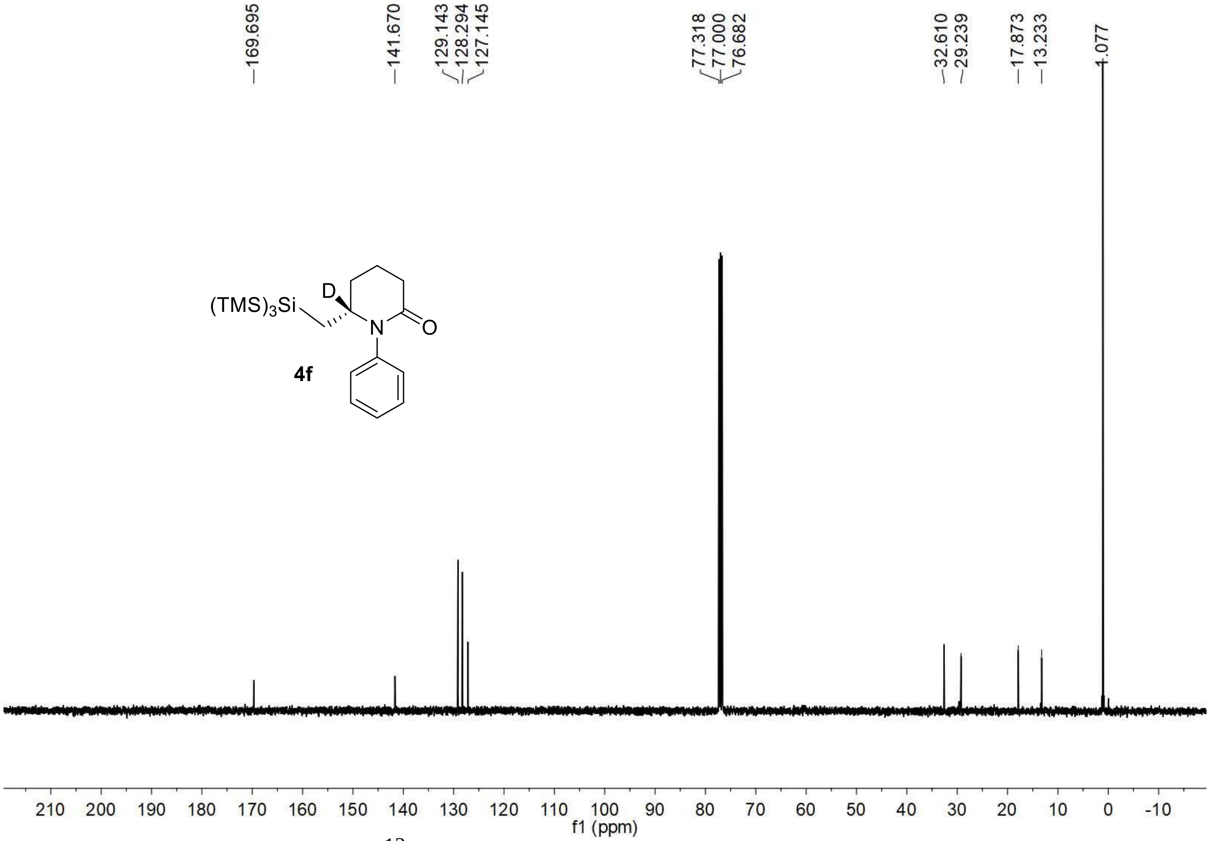

Supplementary Figure 165. ^{13}C NMR spectrum of compound **4f**

HPLC spectrum of racemic **4f**

Sample Name	:
Tray#	: 1
Vial#	: 19
Data File	: sql-7-58-1.lcd
Method File	: 4OD-H-90-0.5-214.lcm
Date Acquired	: 2/16/2022 12:59:30 AM
Date Processed	: 2/16/2022 1:18:36 AM

<Chromatogram View>

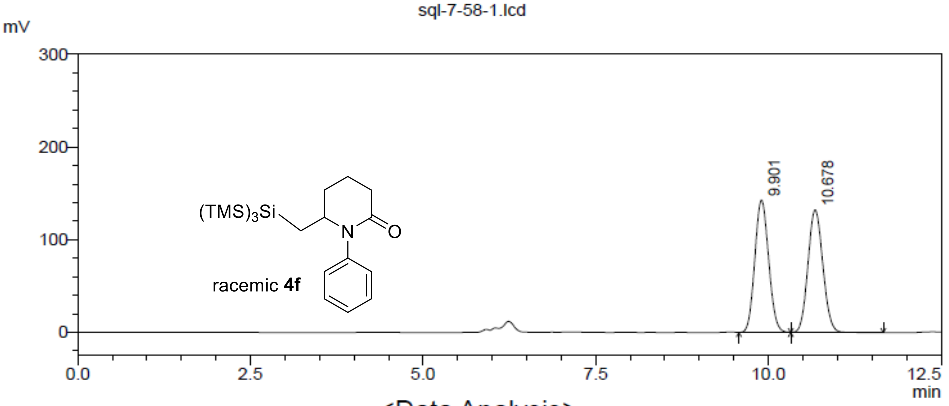

<Data Analysis>

Detector A 214nm

Pesk #	Ret. Time	Height	Area	Area%
1	9.901	142973	1996484	50.066
2	10.678	132311	1991205	49.934
Total		275284	3987689	100.000

Supplementary Figure 166. HPLC spectrum of racemic **4f**

HPLC spectrum of **4f**

Sample Name	:
Tray#	: 1
Vial#	: 20
Data File	: sql-7-58-2.lcd
Method File	: 4OD-H-90-0.5-214.lcm
Date Acquired	: 2/16/2022 1:40:28 AM
Date Processed	: 2/16/2022 1:53:57 AM

<Chromatogram View>

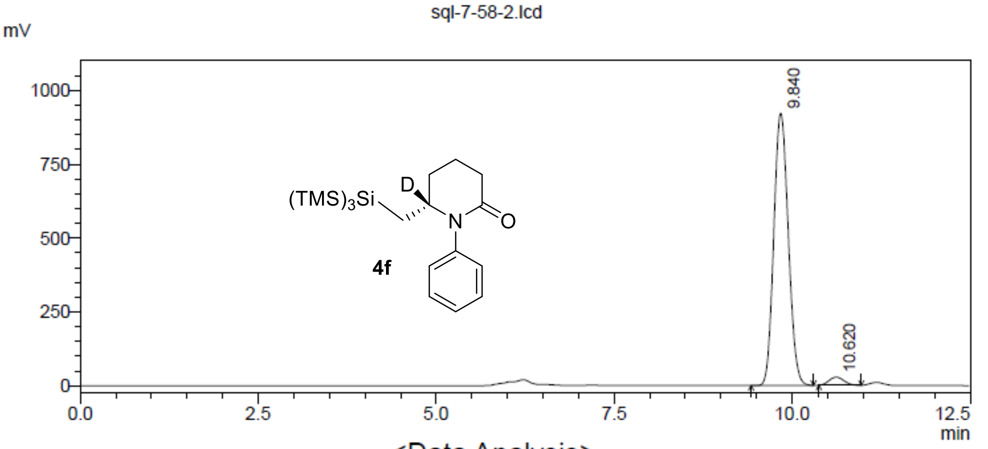

<Data Analysis>

Detector A 214nm

Pesk #	Ret. Time	Height	Area	Area%
1	9.840	921183	13039350	97.050
2	10.620	27517	396394	2.950
Total		948700	13435745	100.000

Supplementary Figure 167. HPLC spectrum of **4f**

^{1}H NMR (400 MHz, room temperature, $CDCl_3$)

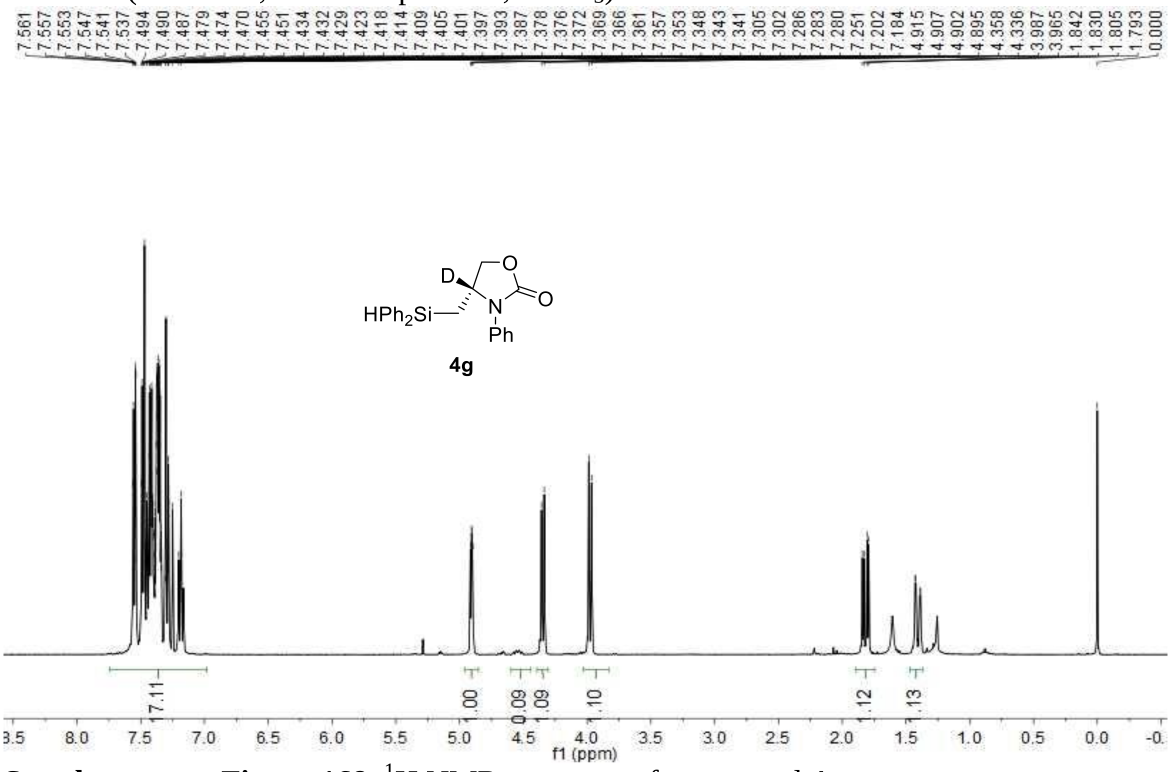

Supplementary Figure 168. ^{1}H NMR spectrum of compound **4g**

^{13}C NMR (100 MHz, room temperature, $CDCl_3$)

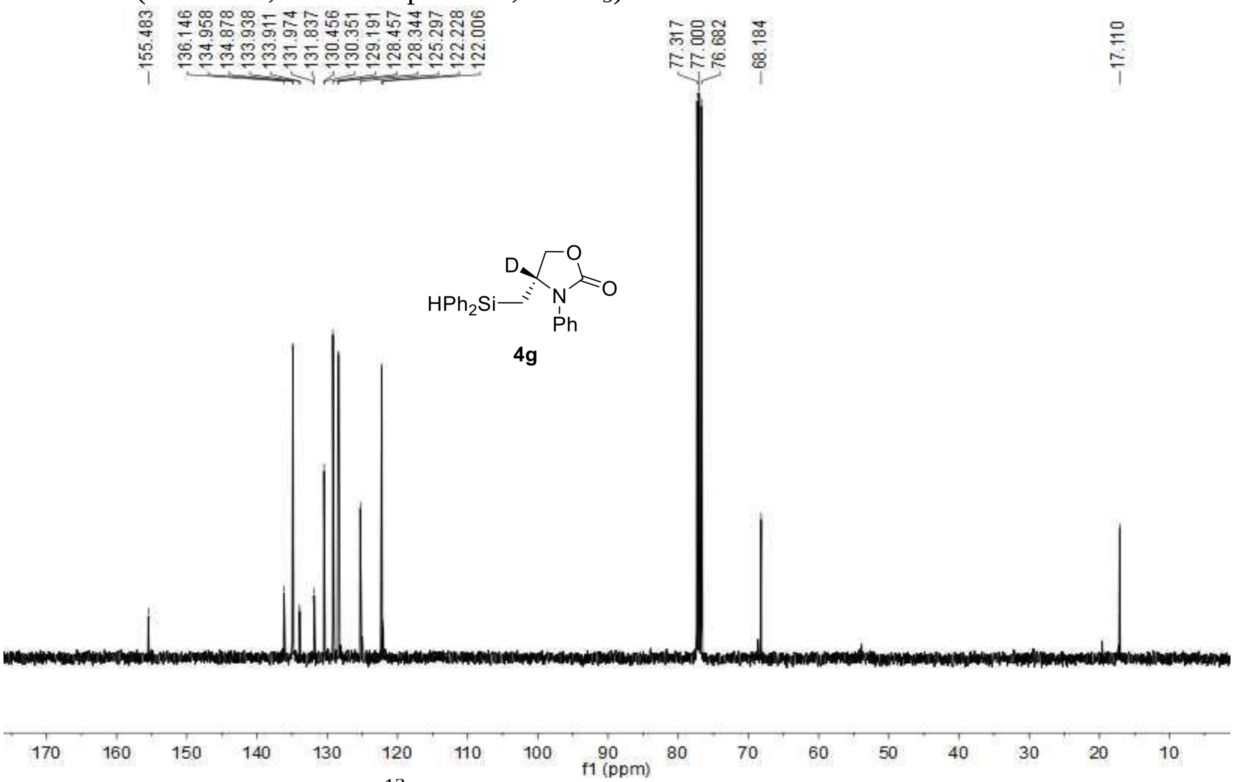

Supplementary Figure 169. ^{13}C NMR spectrum of compound **4g**

HPLC spectrum of racemic **4g**

Tray#	: 1
Vial#	: 22
Data File	: sql-6-404-1-2.lcd
Method File	: 3AD-H-80-1-214.lcm
Date Acquired	: 1/19/2022 8:39:28 PM
Date Processed	: 1/19/2022 9:02:27 PM

<Chromatogram View>

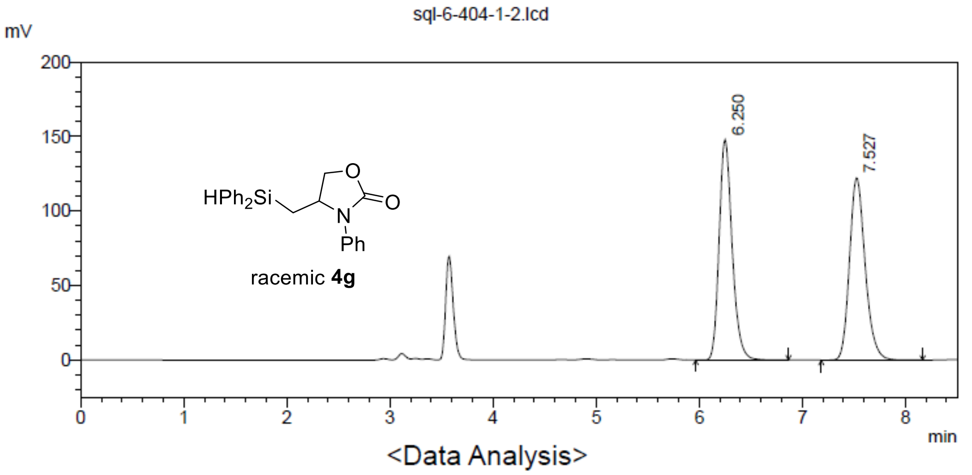

<Data Analysis>

Detector A 214nm

Pesk #	Ret. Time	Height	Area	Area%
1	6.250	148224	1298577	50.014
2	7.527	122521	1297852	49.986
Total		270745	2596429	100.000

Supplementary Figure 170. HPLC spectrum of racemic **4g**

HPLC spectrum of **4g**

Tray# : 1
Vial# : 47
Data File : YXY-2-225B.lcd
Method File : 3AD-H-80-1-214.lcm
Date Acquired : 1/23/2022 11:39:12 PM
Date Processed : 1/25/2022 1:31:31 AM

<Chromatogram View>

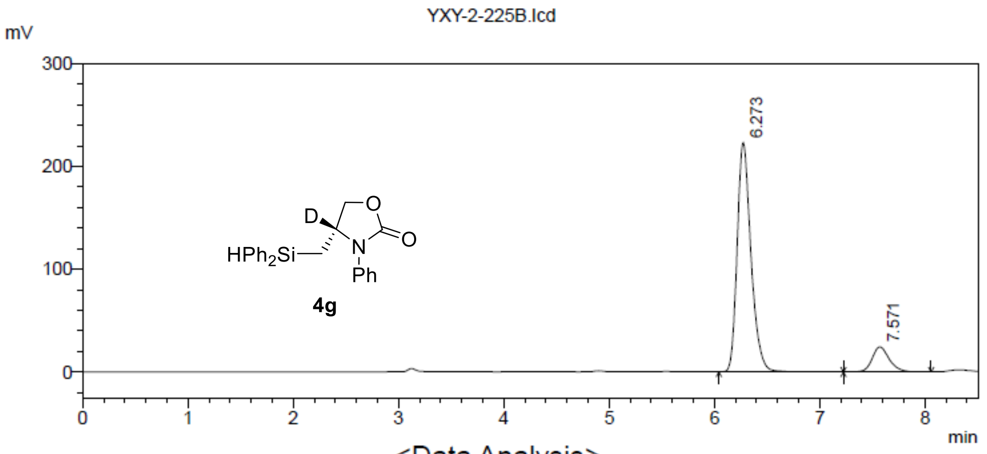

<Data Analysis>

Detector A 214nm

Pesk #	Ret. Time	Height	Area	Area%
1	6.273	223086	2034518	88.062
2	7.571	24366	275815	11.938
Total		247452	2310333	100.000

Supplementary Figure 171. HPLC spectrum of **4g**

^{1}H NMR (400 MHz, room temperature, $CDCl_3$)

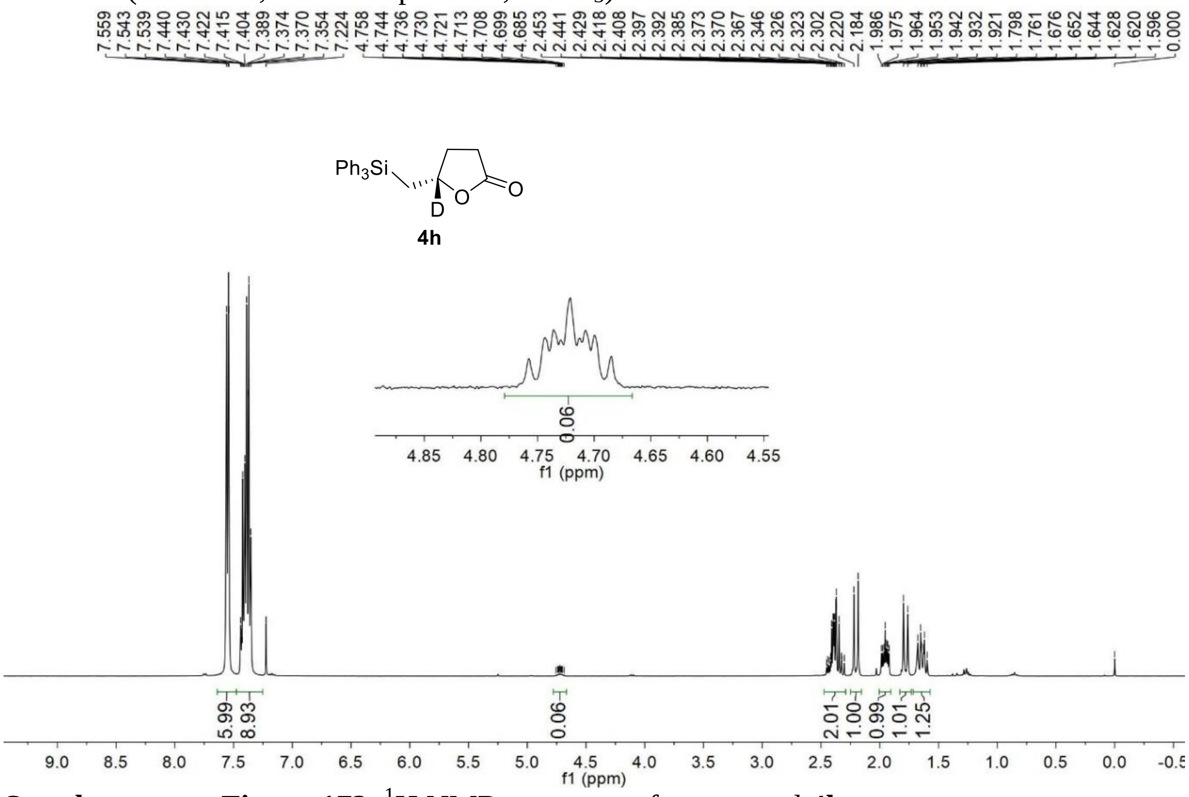

Supplementary Figure 172. ^{1}H NMR spectrum of compound **4h**

^{13}C NMR (100 MHz, room temperature, $CDCl_3$)

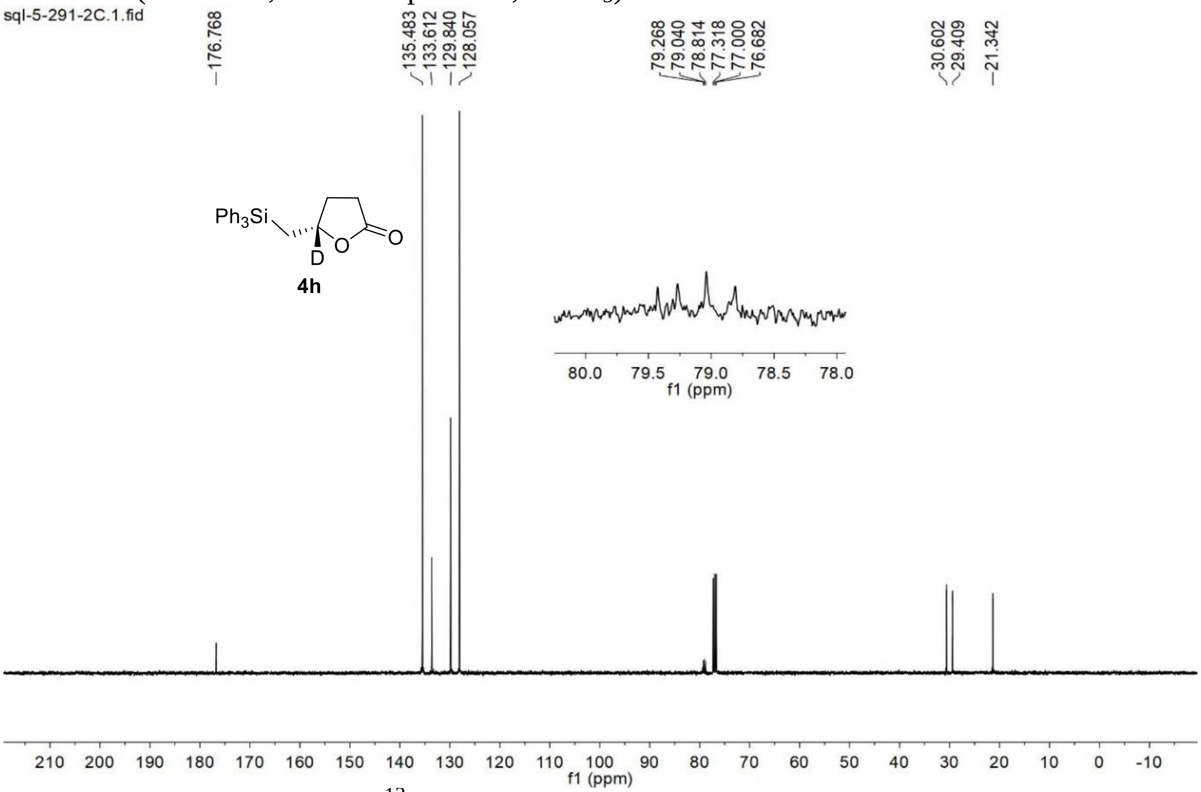

Supplementary Figure 173. ^{13}C NMR spectrum of compound **4h**

HPLC spectrum of racemic **4h**

Data File	: SQL-5-291-1-3.lcd
Method File	: 3AD-H-96-1-214-20min.lcm
Date Processed	: 9/2/2021 9:07:41 AM

<Chromatogram View>

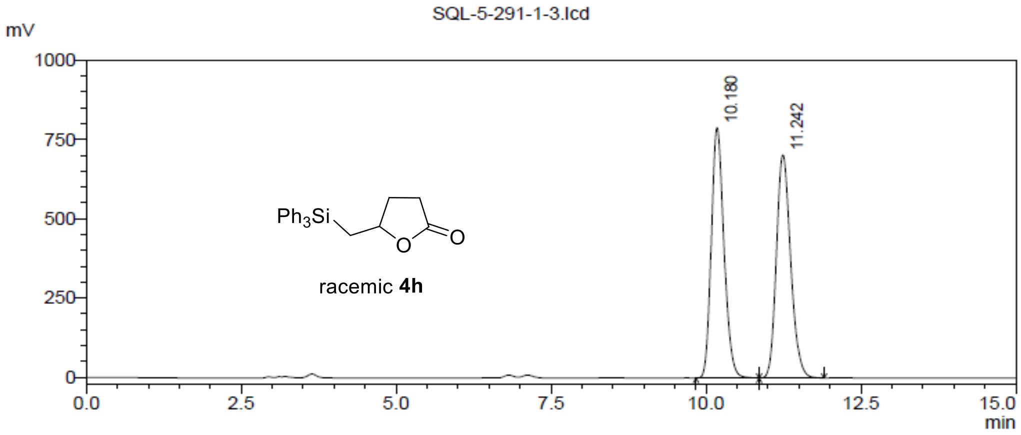

<Data Analysis>

Detector A 214nm

Pesk #	Ret. Time	Height	Area	Area%
1	10.180	789713	11268027	49.931
2	11.242	703279	11299176	50.069
Total		1492992	22567203	100.000

Supplementary Figure 174. HPLC spectrum of racemic **4h**

HPLC spectrum of **4h**

Data File : SQL-5-375-6.lcd
Method File : 3AD-H-96-1-214-20min.lcm
Date Processed : 9/2/2021 9:07:29 AM

<Chromatogram View>

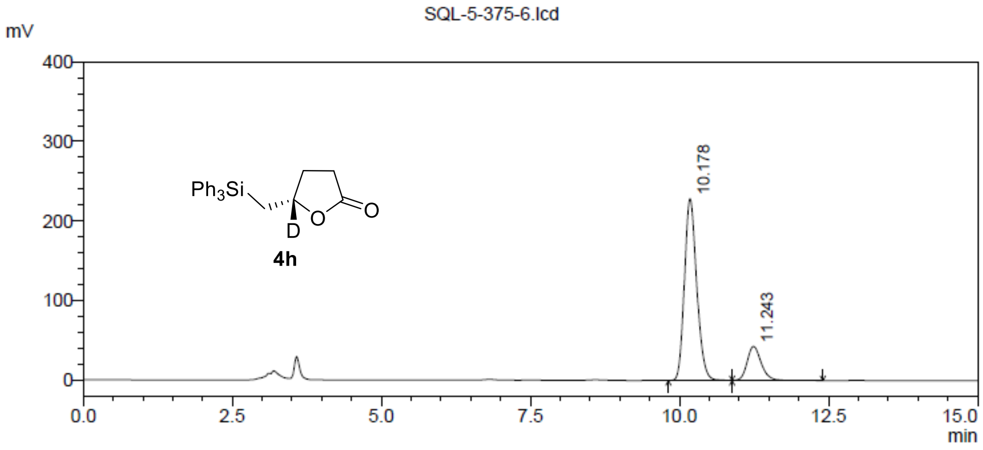

<Data Analysis>

Detector A 214nm

Pesk #	Ret. Time	Height	Area	Area%
1	10.178	228721	3288228	82.351
2	11.243	42756	704692	17.649
Total		271477	3992921	100.000

Supplementary Figure 175. HPLC spectrum of **4h**

^{1}H NMR (400 MHz, room temperature, $CDCl_3$)

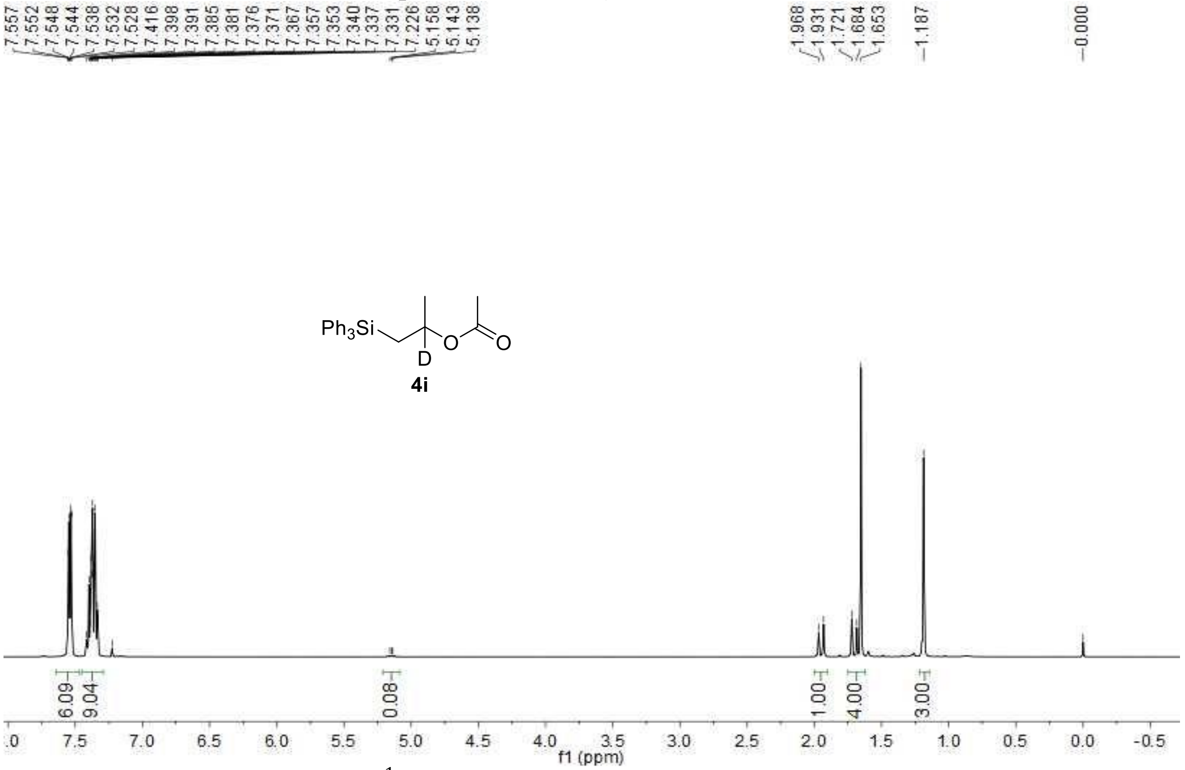

Supplementary Figure 176. ^{1}H NMR spectrum of compound **4i**

^{13}C NMR (100 MHz, room temperature, $CDCl_3$)

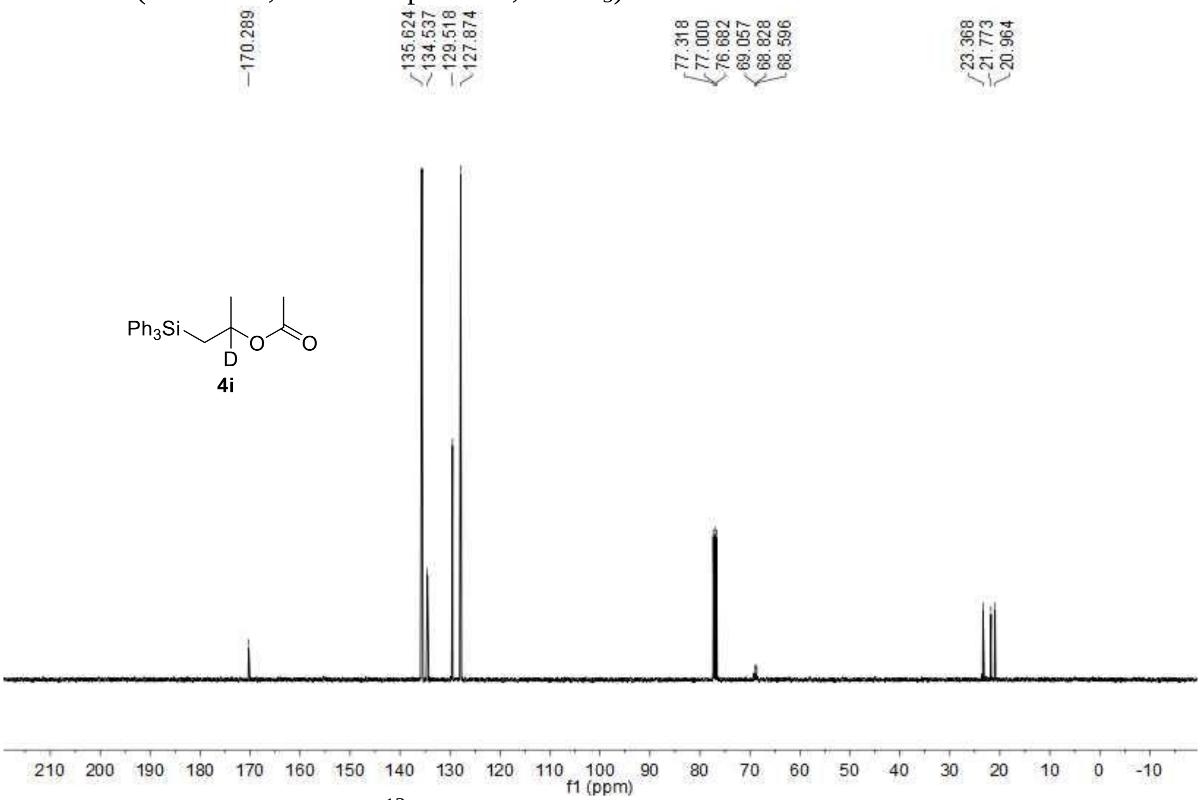

Supplementary Figure 177. ^{13}C NMR spectrum of compound **4i**

HPLC spectrum of racemic **4i**

Tray#	: 1
Vial#	: 19
Data File	: sql-5-284-1-1.lcd
Method File	: 1OJ-H-99-1-214.lcm
Date Acquired	: 2/24/2022 9:03:24 AM
Date Processed	: 2/24/2022 9:41:20 AM

<Chromatogram View>

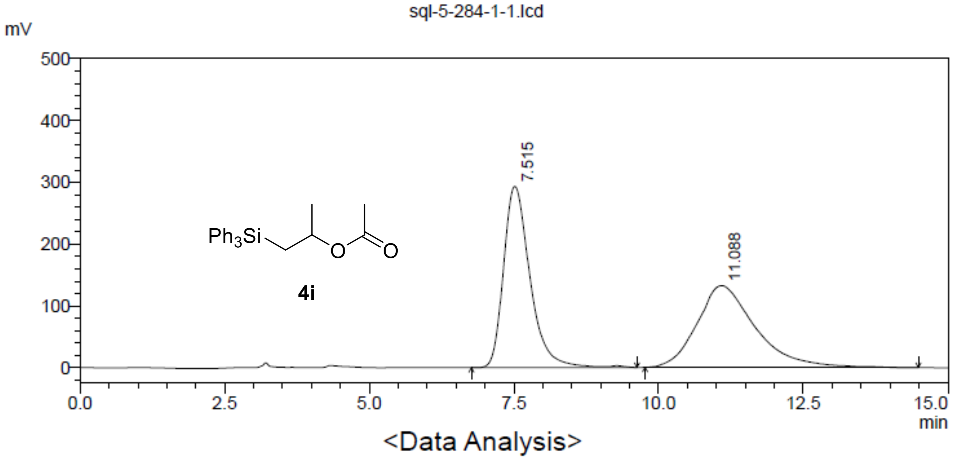

<Data Analysis>

Detector A 214nm

Pesk #	Ret. Time	Height	Area	Area%
1	7.515	293022	9329667	50.117
2	11.088	132355	9285944	49.883
Total		425377	18615611	100.000

Supplementary Figure 178. HPLC spectrum of racemic **4i**

HPLC spectrum of **4i**

Sample Name	:
Tray#	: 1
Vial#	: 20
Data File	: sql-5-284-2.lcd
Method File	: 1OJ-H-99-1-214.lcm
Date Acquired	: 2/24/2022 9:20:09 AM
Date Processed	: 2/24/2022 9:41:57 AM

<Chromatogram View>

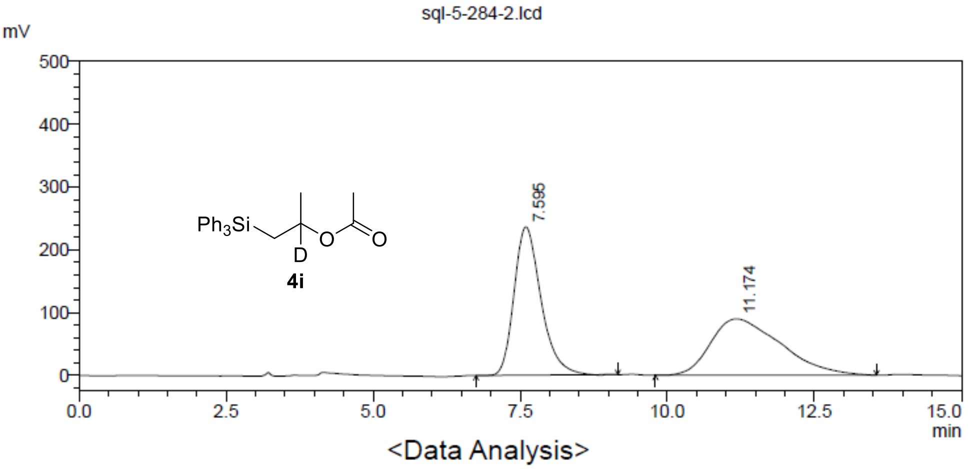

<Data Analysis>

Detector A 214nm

Pesk #	Ret. Time	Height	Area	Area%
1	7.595	236398	7534238	50.343
2	11.174	89784	7431712	49.657
Total		326182	14965950	100.000

Supplementary Figure 179. HPLC spectrum of **4i**

^{1}H NMR (400 MHz, room temperature, $CDCl_3$)

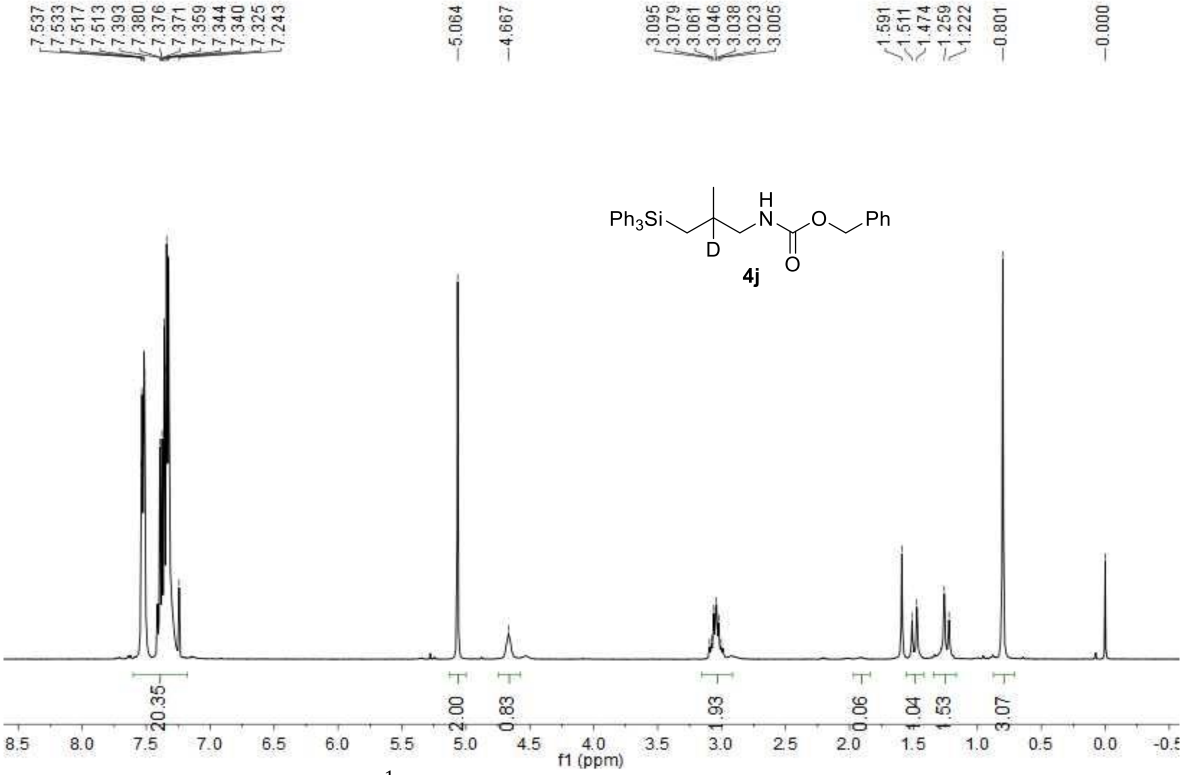

Supplementary Figure 180. ^{1}H NMR spectrum of compound **4j**

^{13}C NMR (100 MHz, room temperature, $CDCl_3$)

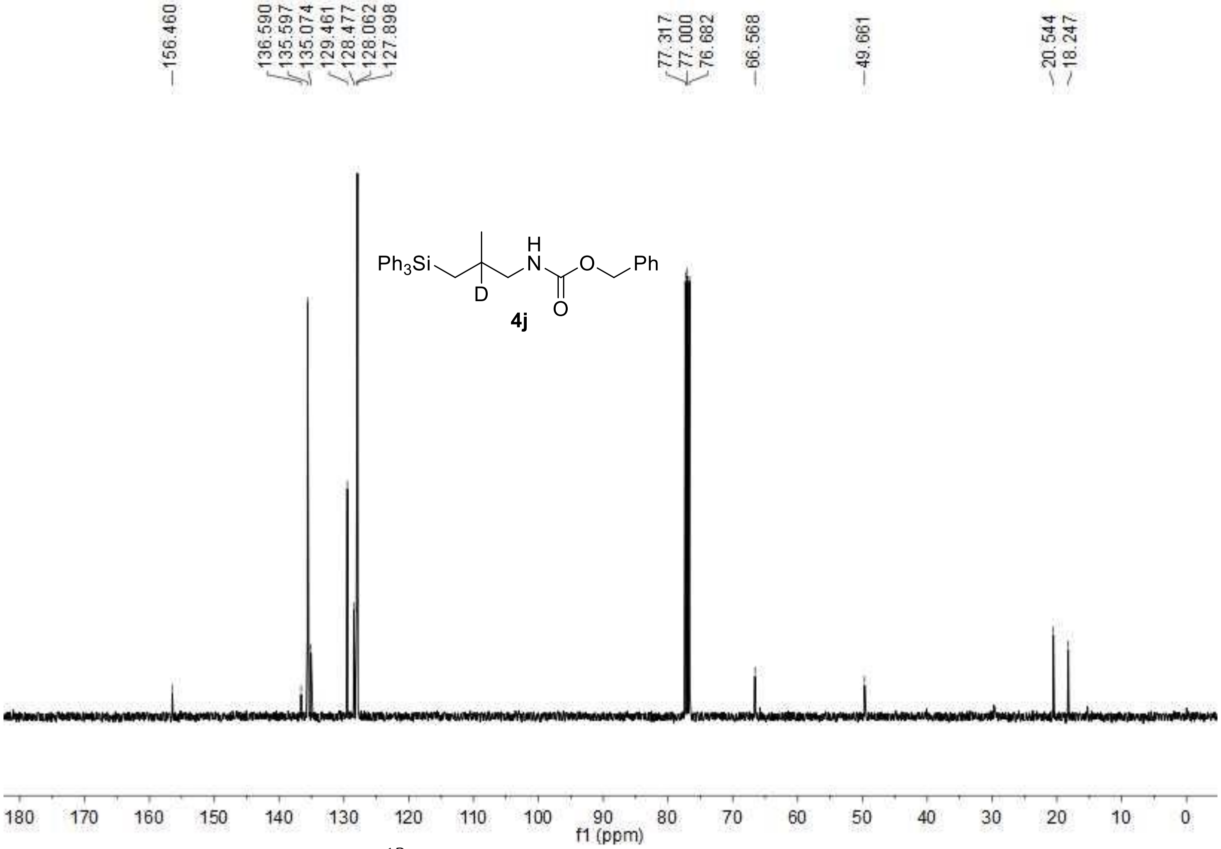

Supplementary Figure 181. ^{13}C NMR spectrum of compound **4j**

HPLC spectrum of racemic **4j**

Tray#	: 1
Vial#	: 19
Data File	: sql-6-217-1-1-3.lcd
Method File	: 3AD-H-96-1-214.lcm
Date Acquired	: 11/19/2021 5:07:52 PM
Date Processed	: 2/26/2022 12:31:48 AM

<Chromatogram View>

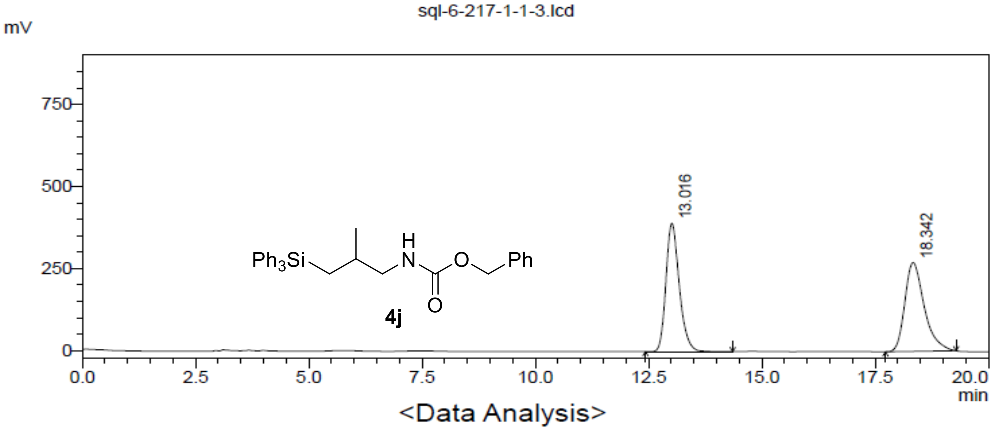

<Data Analysis>

Detector A 214nm

Pesk #	Ret. Time	Height	Area	Area%
1	13.016	391460	8033887	49.814
2	18.342	270979	8093922	50.186
Total		662438	16127810	100.000

Supplementary Figure 182. HPLC spectrum of racemic **4j**

HPLC spectrum of **4j**

Sample Name :
Tray# : 1
Vial# : 19
Data File : sql-6-217-2-1.lcd
Method File : 3AD-H-96-1-214.lcm
Date Acquired : 11/13/2021 3:10:08 PM
Date Processed : 11/13/2021 6:03:43 PM

<Chromatogram View>

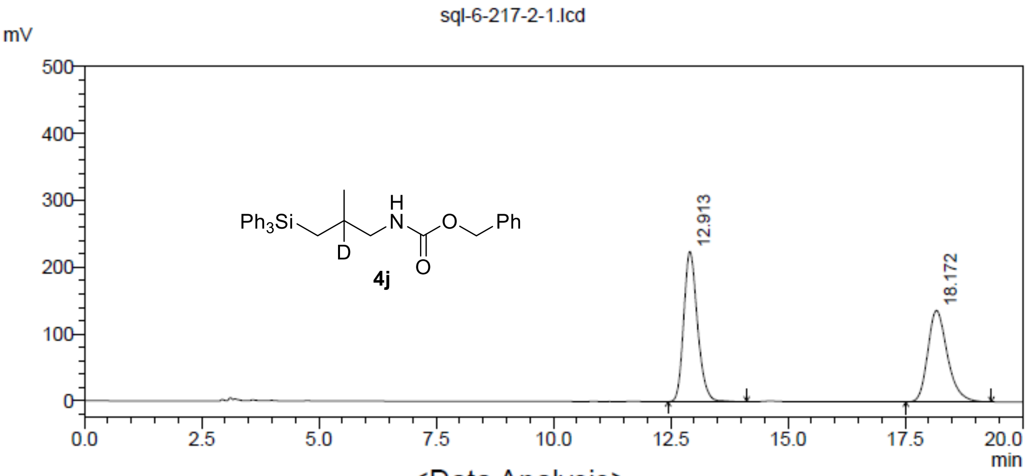

<Data Analysis>

Detector A 214nm

Pesk #	Ret. Time	Height	Area	Area%
1	12.913	224455	4578526	53.444
2	18.172	136651	3988398	46.556
Total		361106	8566924	100.000

Supplementary Figure 183. HPLC spectrum of **4j**

^{1}H NMR (400 MHz, room temperature, $CDCl_3$)

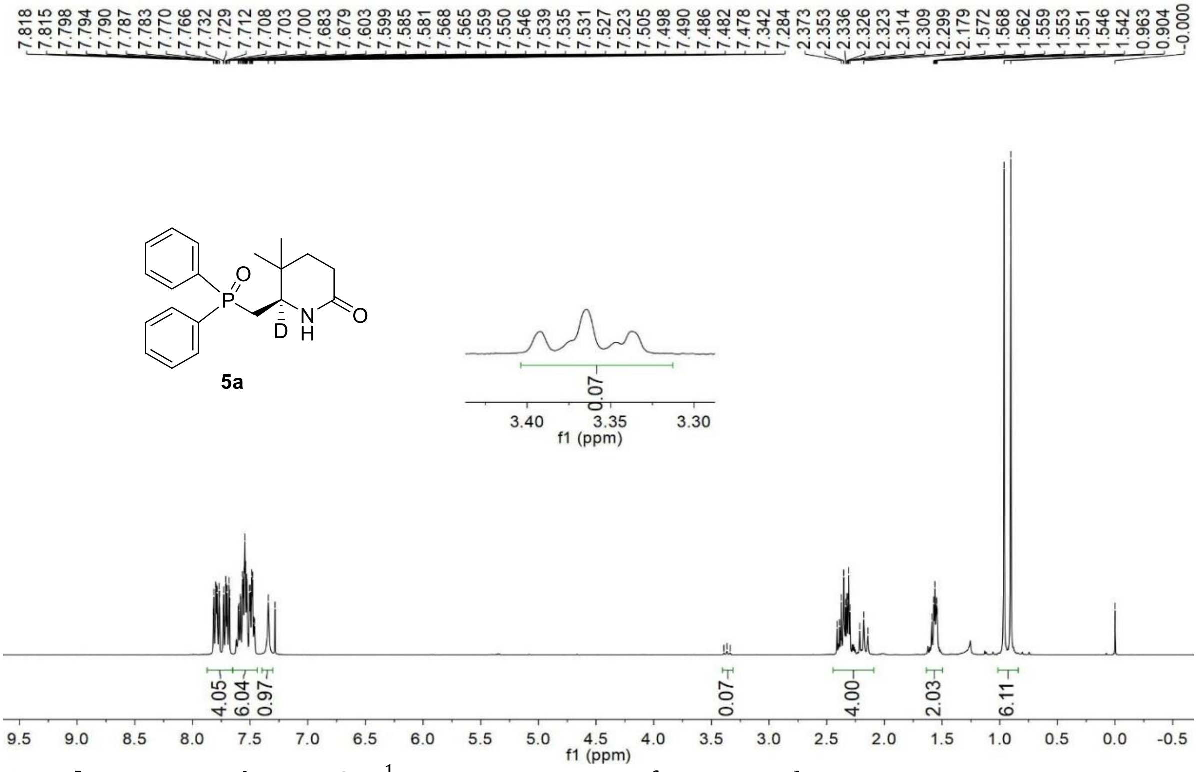

Supplementary Figure 184. ^{1}H NMR spectrum of compound **5a**

^{13}C NMR (100 MHz, room temperature, $CDCl_3$)

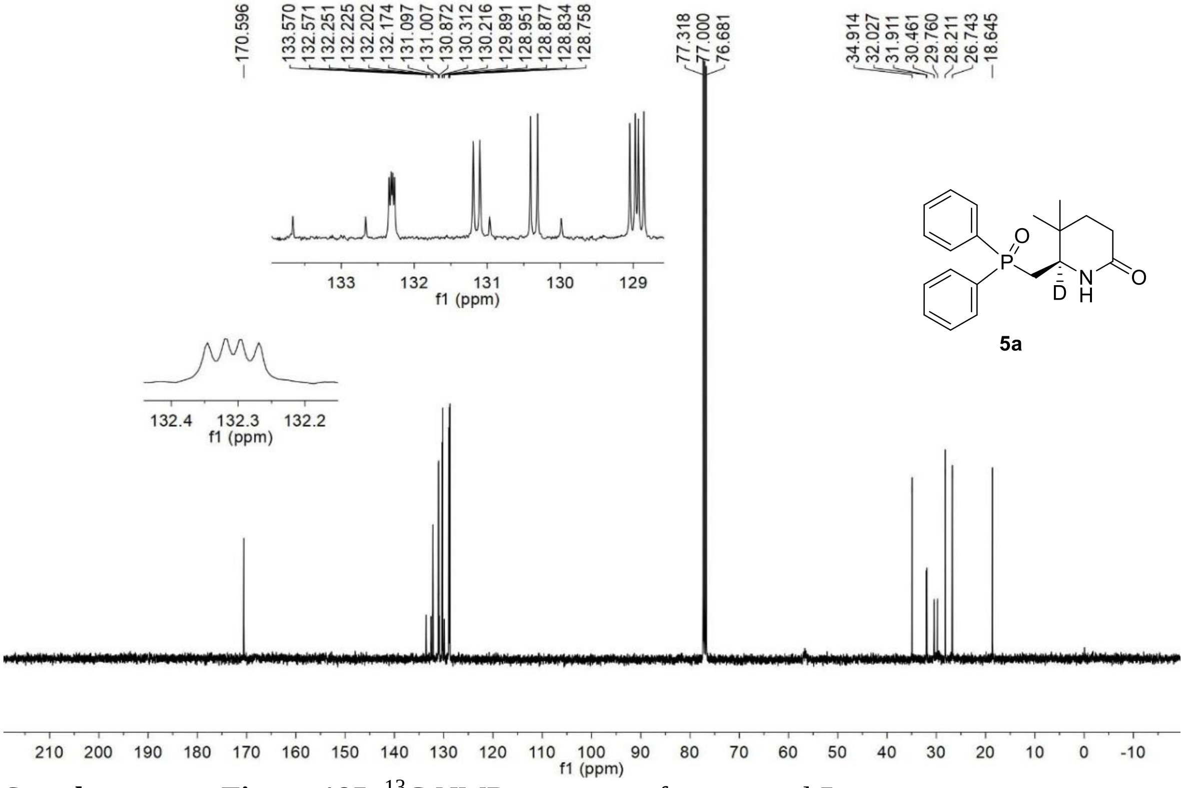

Supplementary Figure 185. ^{13}C NMR spectrum of compound **5a**

^{31}P NMR (162 MHz, room temperature, $CDCl_3$)

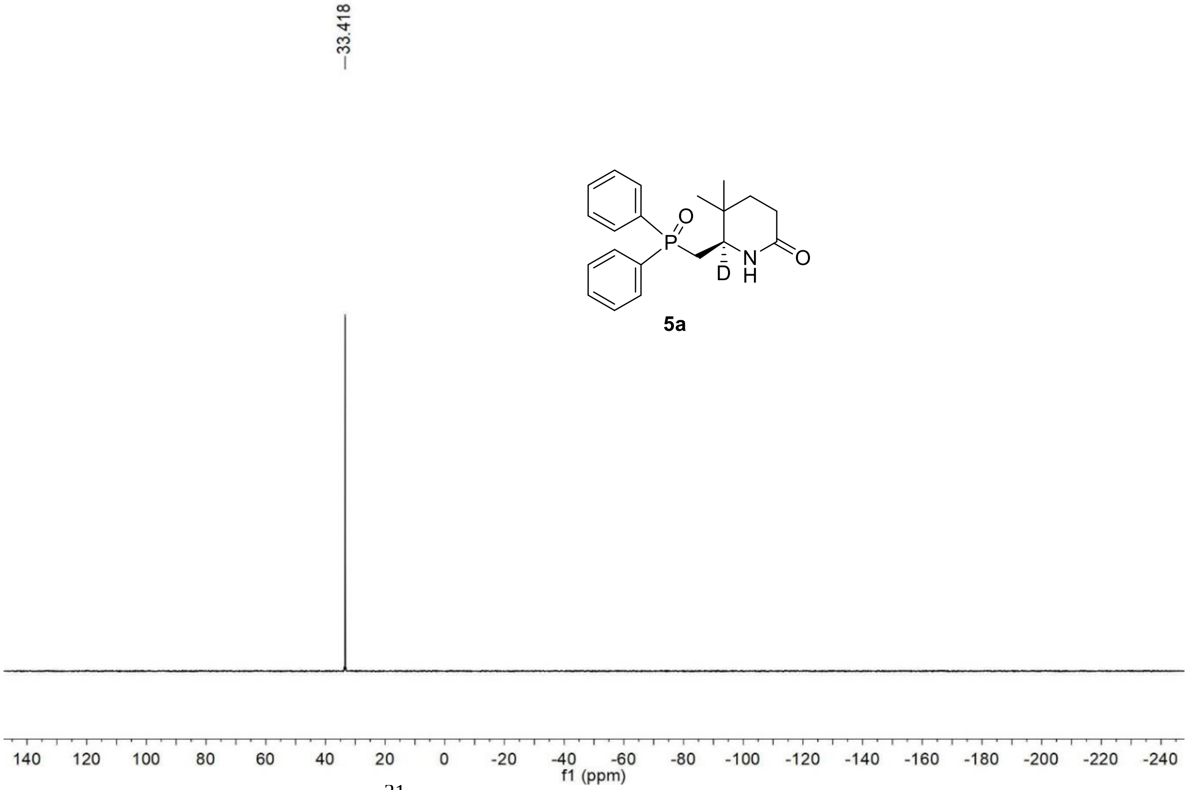

Supplementary Figure 186. ^{31}P NMR spectrum of compound **5a**

HPLC spectrum of racemic **5a**

Data File	: (39-racemic)SQL-4-235-1.lcd
Method File	: AD-H-65-0.5-214.lcm
Date Processed	: 7/17/2021 12:34:24 PM

<Chromatogram View>

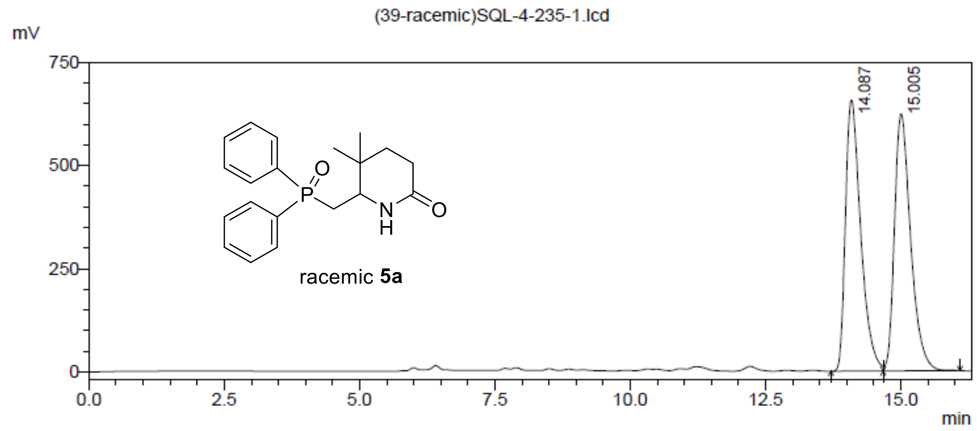

<Data Analysis>

DetA 214nm

Pesk #	Ret. Time	Height	Area	Area%
1	14.087	656825	12878308	49.830
2	15.005	622289	12966043	50.170
Total		1279115	25844350	100.000

Supplementary Figure 187. HPLC spectrum of racemic **5a**

HPLC spectrum of **5a**

Data File	: (39-chiral)SQL-4-258-2-.lcd
Method File	: 3AD-H-65-0.5-214.lcm
Date Processed	: 7/17/2021 12:34:07 PM

<Chromatogram View>

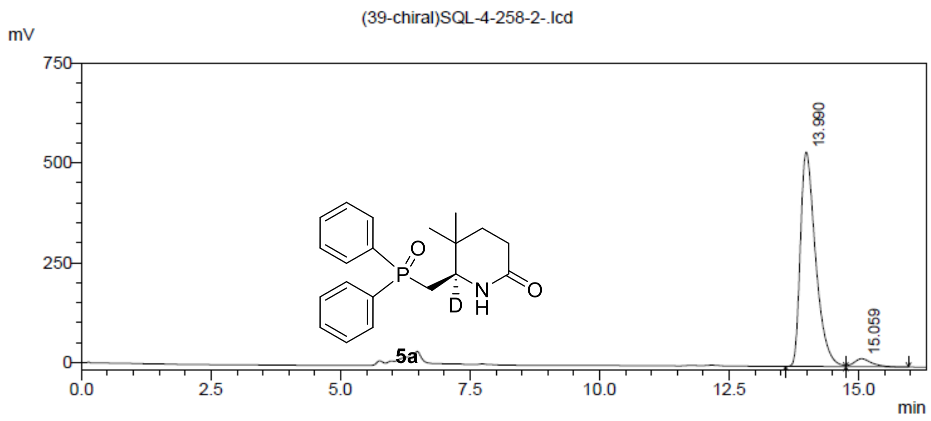

<Data Analysis>

DetA 214nm

Pesk #	Ret. Time	Height	Area	Area%
1	13.990	536413	10961497	95.919
2	15.059	19860	466318	4.081
Total		556273	11427816	100.000

Supplementary Figure 188. HPLC spectrum of **5a**

^{1}H NMR (400 MHz, room temperature, $CDCl_3$)

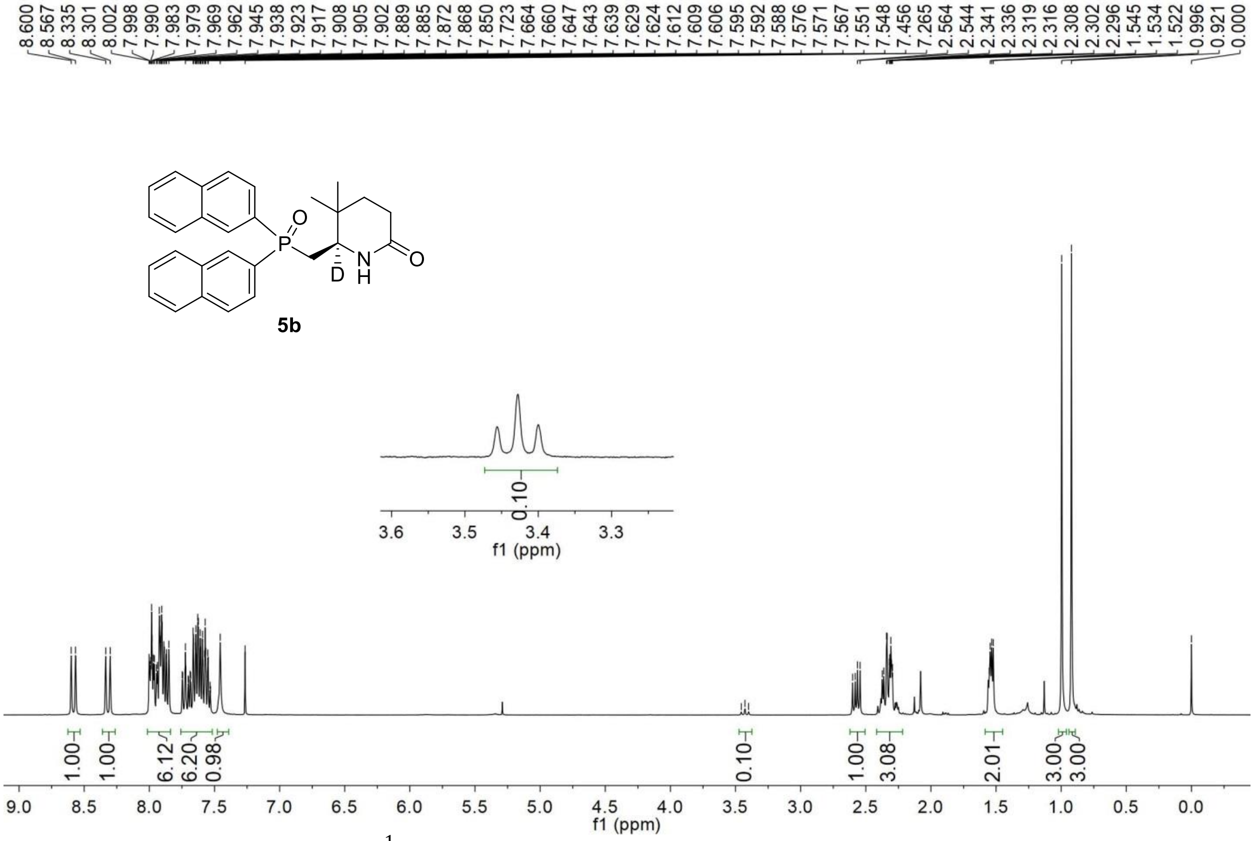

Supplementary Figure 189. ^{1}H NMR spectrum of compound **5b**

^{13}C NMR (100 MHz, room temperature, $CDCl_3$)

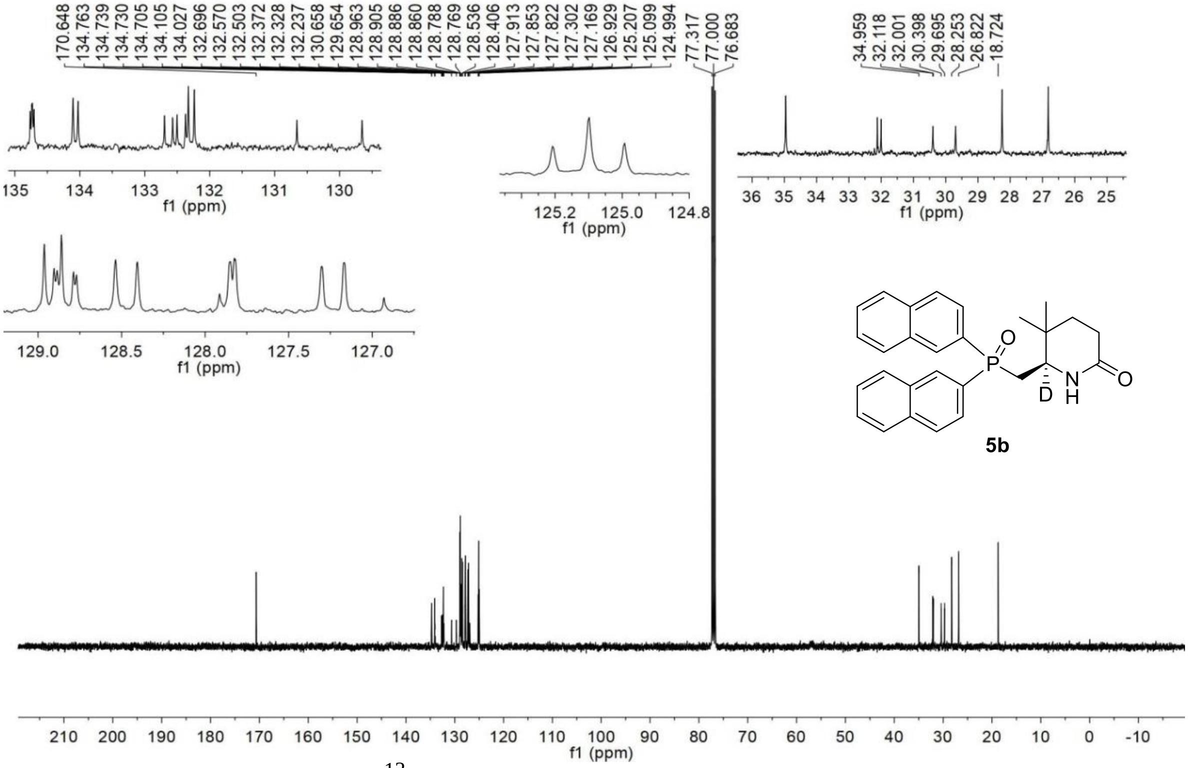

Supplementary Figure 190. ^{13}C NMR spectrum of compound **5b**

^{31}P NMR (162 MHz, room temperature, $CDCl_3$)

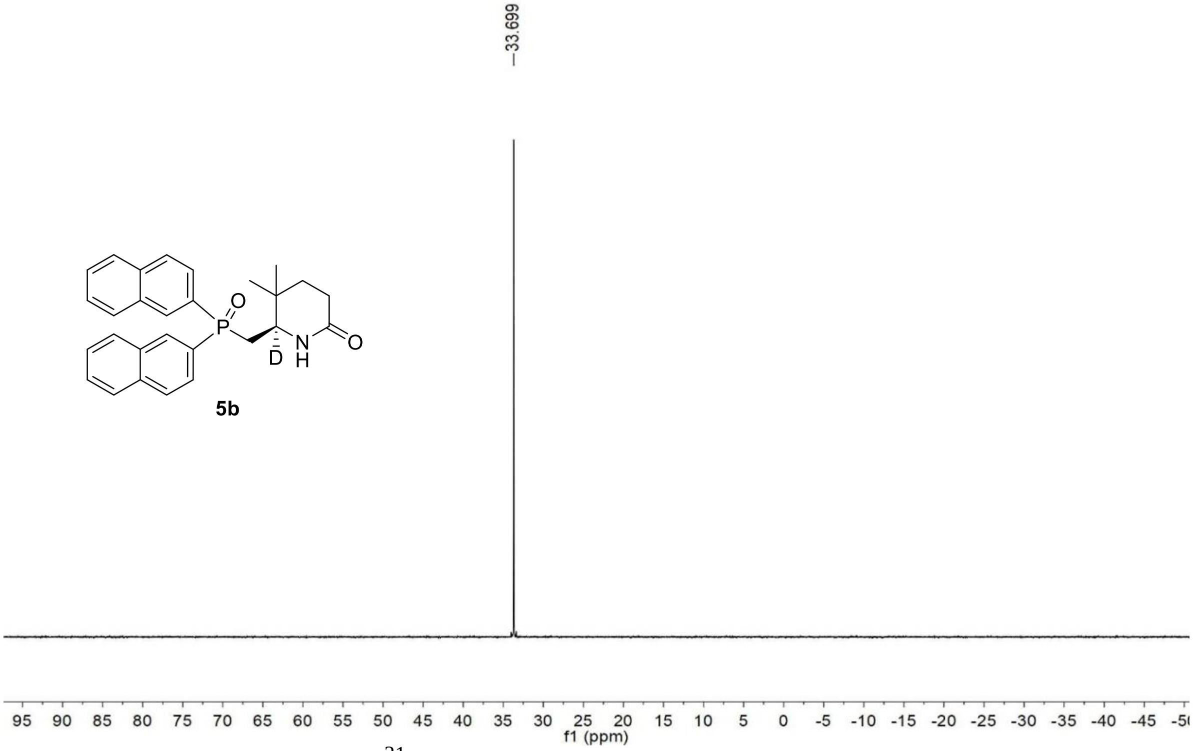

Supplementary Figure 191. ^{31}P NMR spectrum of compound **5b**

HPLC spectrum of racemic **5b**

Data File	: (40-racemic)SQL-4-323-1.lcd
Method File	: 4OD-H-80-1-214.lcm
Date Processed	: 7/17/2021 12:35:12 PM

<Chromatogram View>

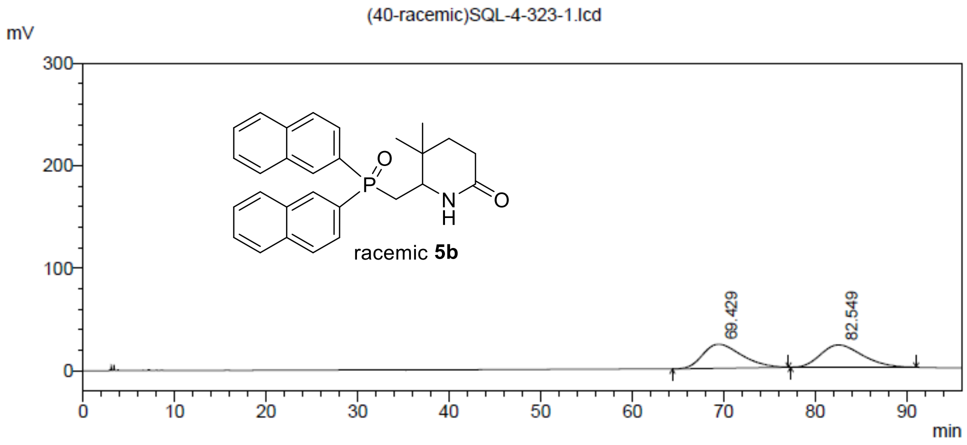

<Data Analysis>

DetA 214nm

Pesk #	Ret. Time	Height	Area	Area%
1	69.429	23424	6877168	49.802
2	82.549	21776	6931807	50.198
Total		45200	13808974	100.000

Supplementary Figure 192. HPLC spectrum of racemic **5b**

HPLC spectrum of **5b**

Data File	: (40-chiral)SQL-4-327.lcd
Method File	: 4OD-H-80-1-214.lcm
Date Processed	: 7/17/2021 12:34:55 PM

<Chromatogram View>

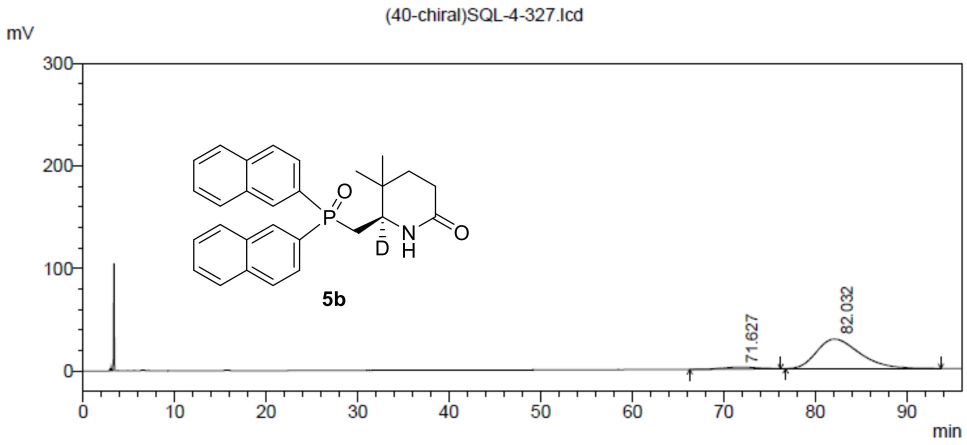

<Data Analysis>

DetA 214nm

Pesk #	Ret. Time	Height	Area	Area%
1	71.627	1711	467050	4.732
2	82.032	28748	9402629	95.268
Total		30459	9869679	100.000

Supplementary Figure 193. HPLC spectrum of **5b**

^{1}H NMR (400 MHz, room temperature, $CDCl_3$)

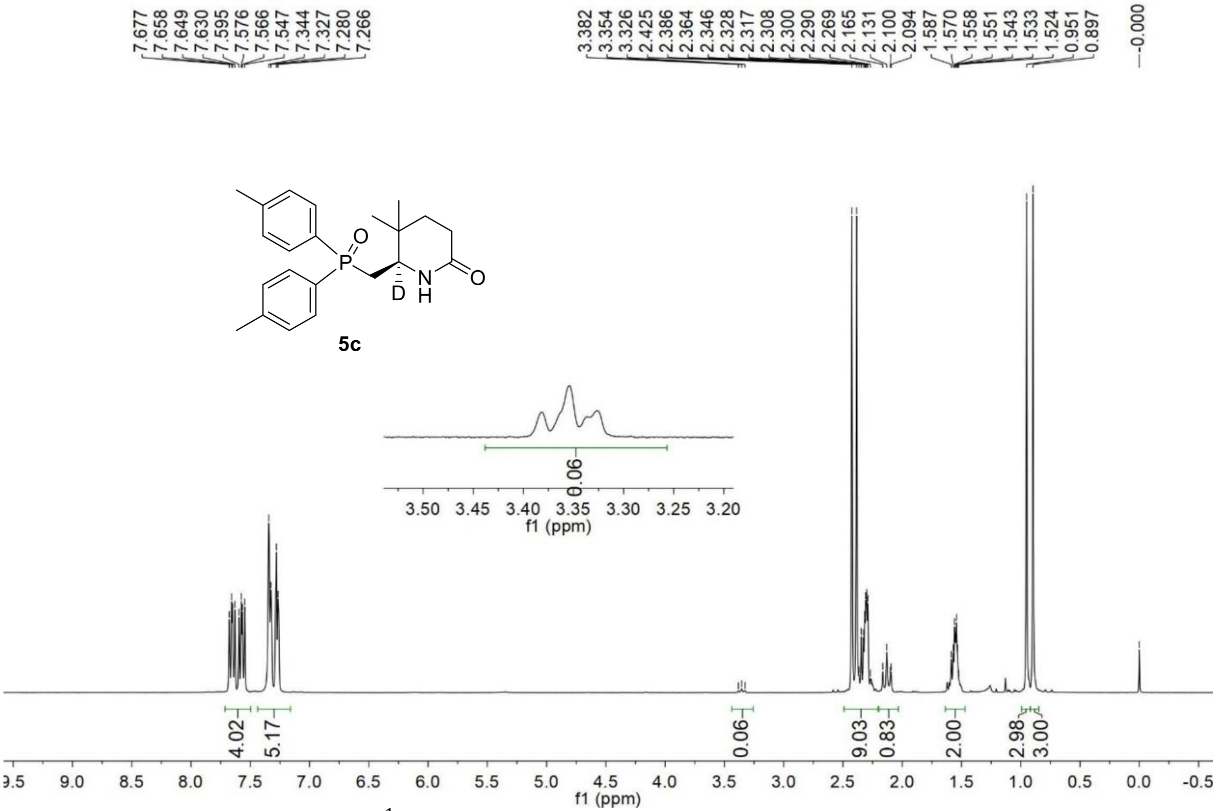

Supplementary Figure 194. ^{1}H NMR spectrum of compound **5c**

^{13}C NMR (100 MHz, room temperature, $CDCl_3$)

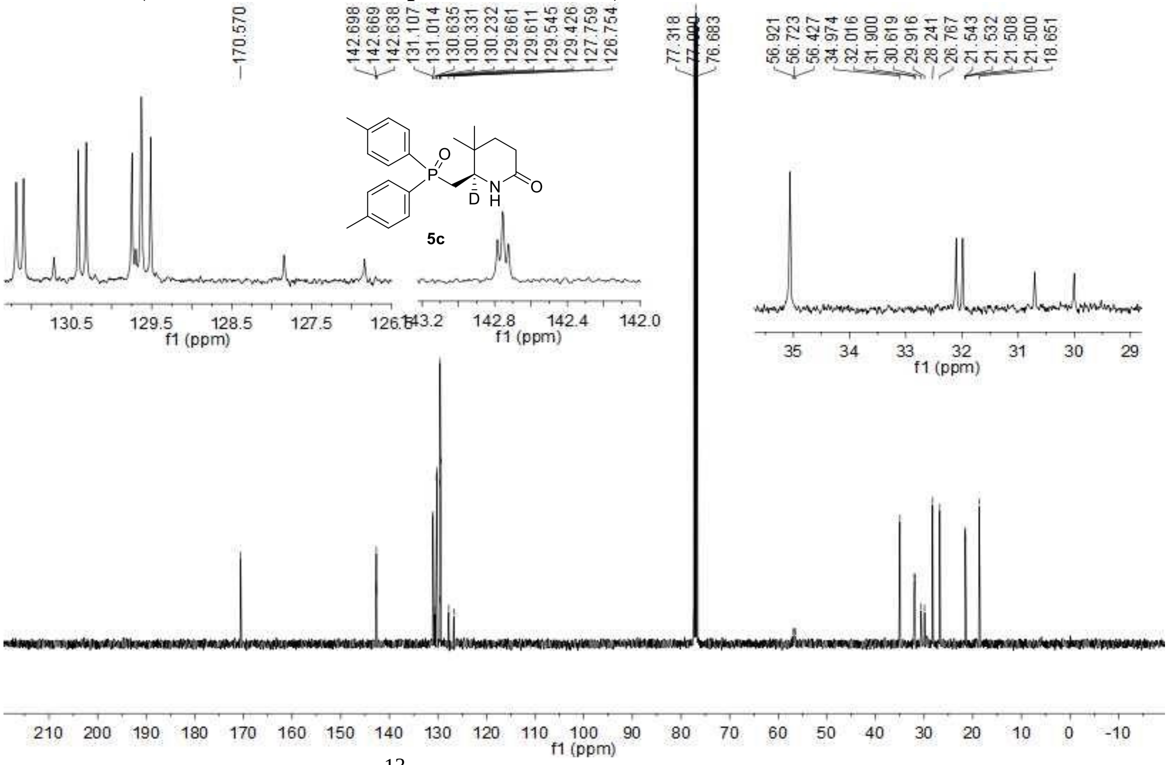

Supplementary Figure 195. ^{13}C NMR spectrum of compound **5c**

^{31}P NMR (162 MHz, room temperature, $CDCl_3$)

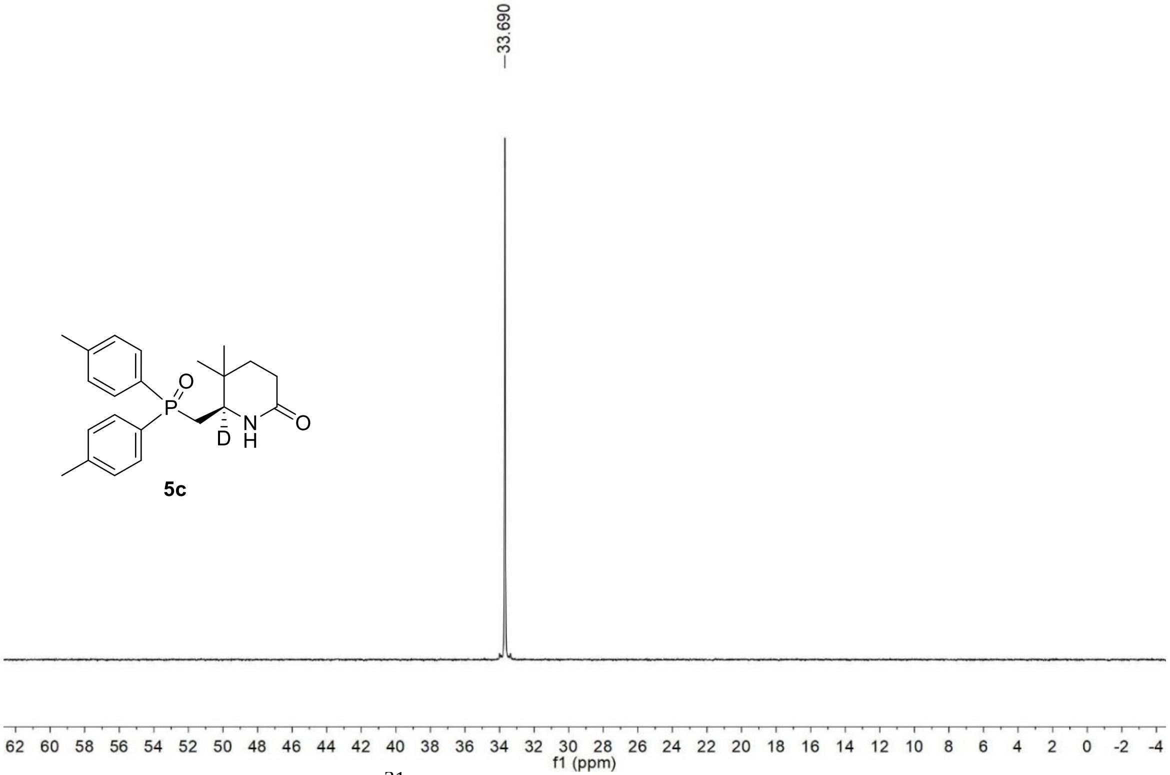

Supplementary Figure 196. ^{31}P NMR spectrum of compound **5c**

HPLC spectrum of racemic **5c**

Data File : (41-racemic)SQL-4-243-1.lcd
Method File : AD-H-60-0.5-214.lcm
Date Processed : 7/17/2021 12:35:49 PM

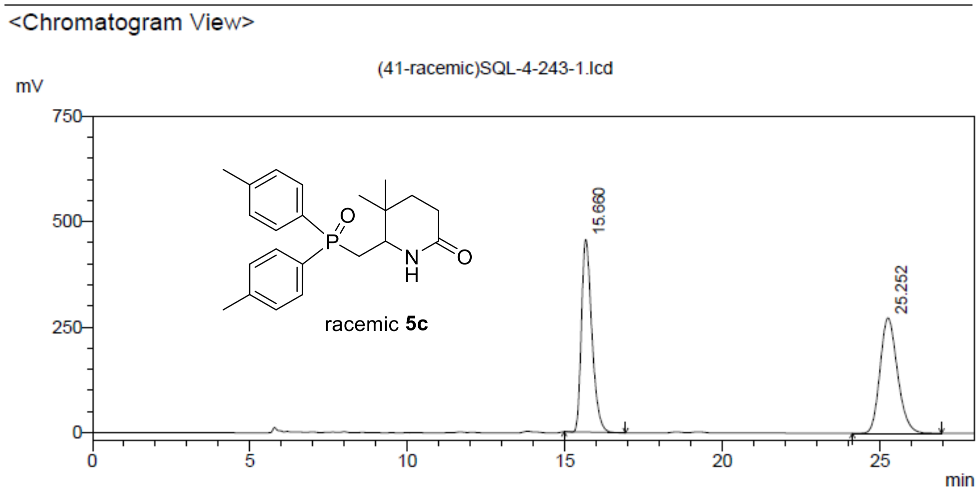

<Data Analysis>

DetA 214nm

Pesk #	Ret. Time	Height	Area	Area%
1	15.660	455855	10509646	49.931
2	25.252	273003	10538783	50.069
Total		728857	21048429	100.000

Supplementary Figure 197. HPLC spectrum of racemic **5c**

HPLC spectrum of **5c**

Data File : (41-chiral)SQL-4-300.lcd
Method File : 3AD-H-60-0.5-214.lcm
Date Processed : 7/17/2021 12:35:35 PM

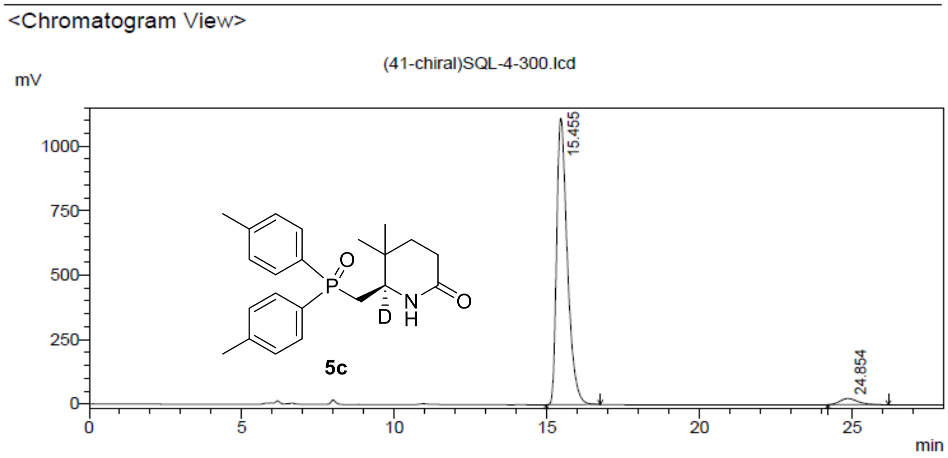

<Data Analysis>

DetA 214nm

Pesk #	Ret. Time	Height	Area	Area%
1	15.455	1112509	27098253	96.517
2	24.854	23974	977936	3.483
Total		1136483	28076190	100.000

Supplementary Figure 198. HPLC spectrum of **5c**

^{1}H NMR (400 MHz, room temperature, $CDCl_3$)

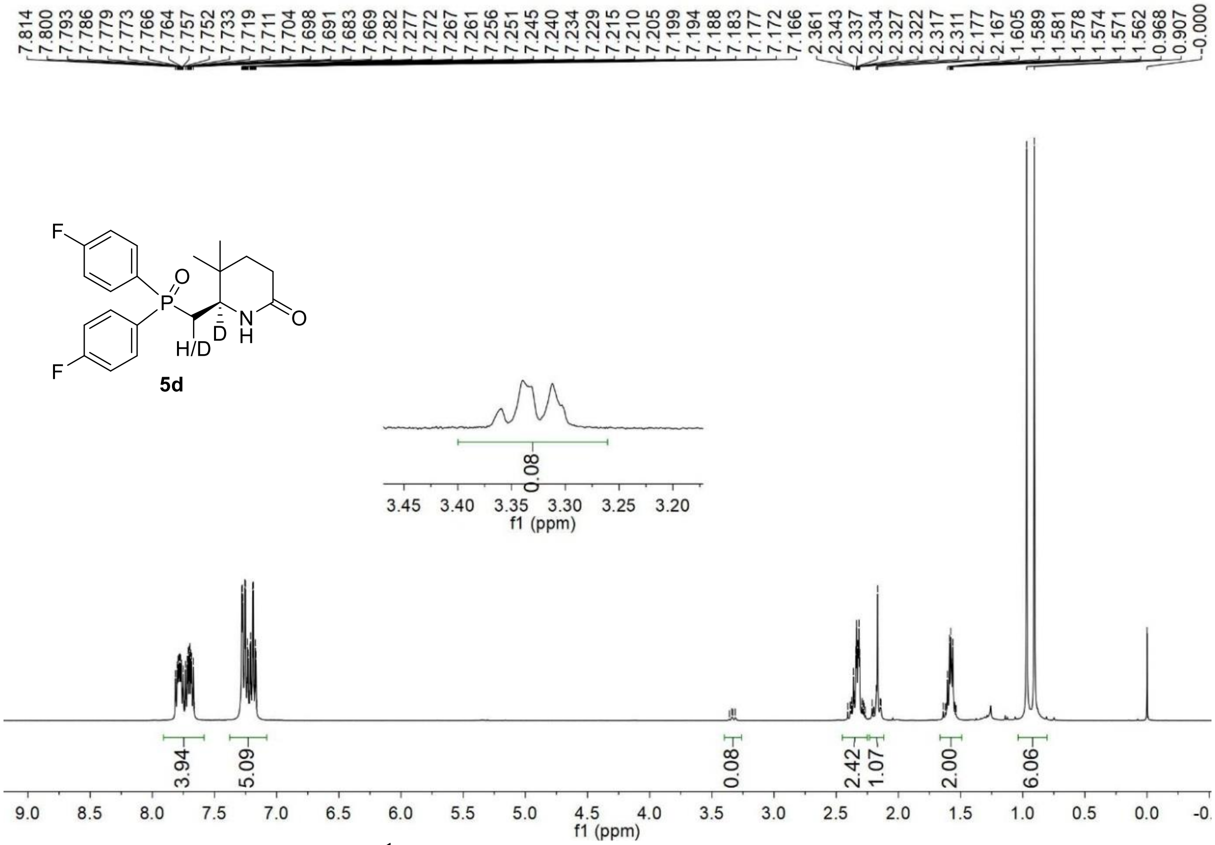

Supplementary Figure 199. ^{1}H NMR spectrum of compound **5d**

^{13}C NMR (100 MHz, room temperature, $CDCl_3$)

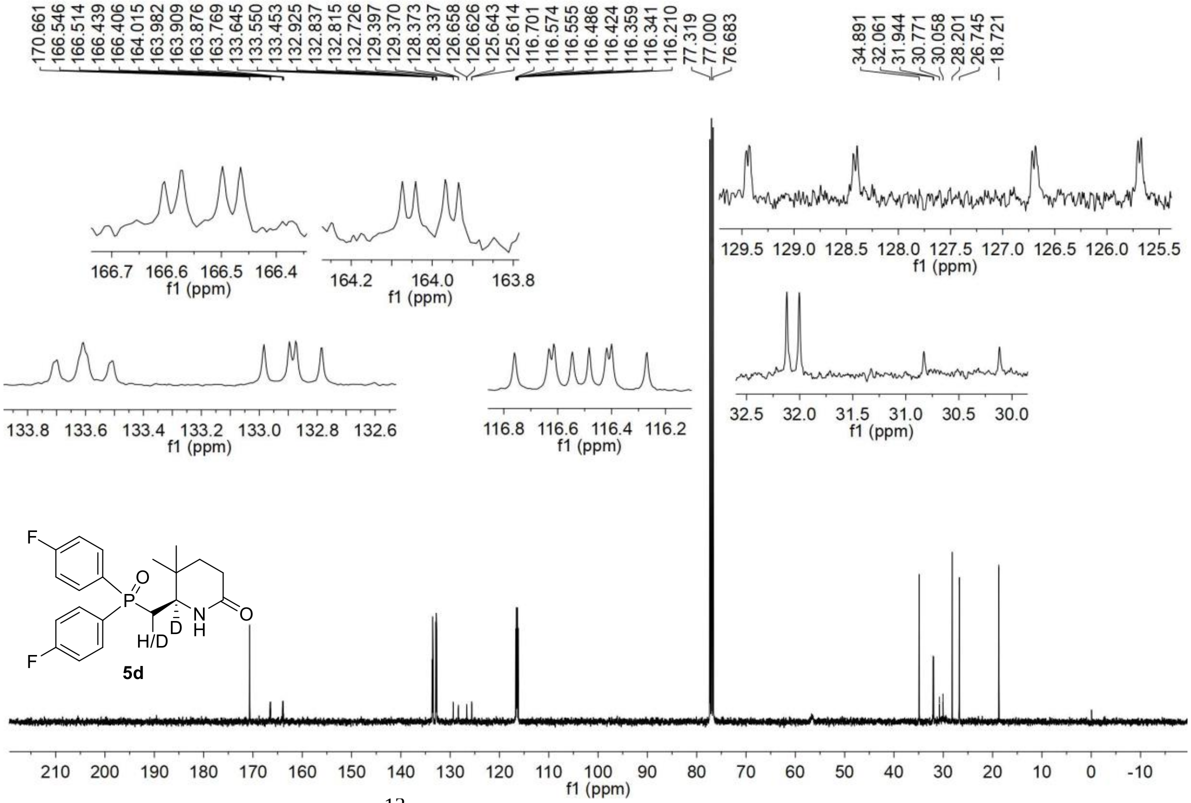

Supplementary Figure 200. ^{13}C NMR spectrum of compound **5d**

^{31}P NMR (162 MHz, room temperature, $CDCl_3$)

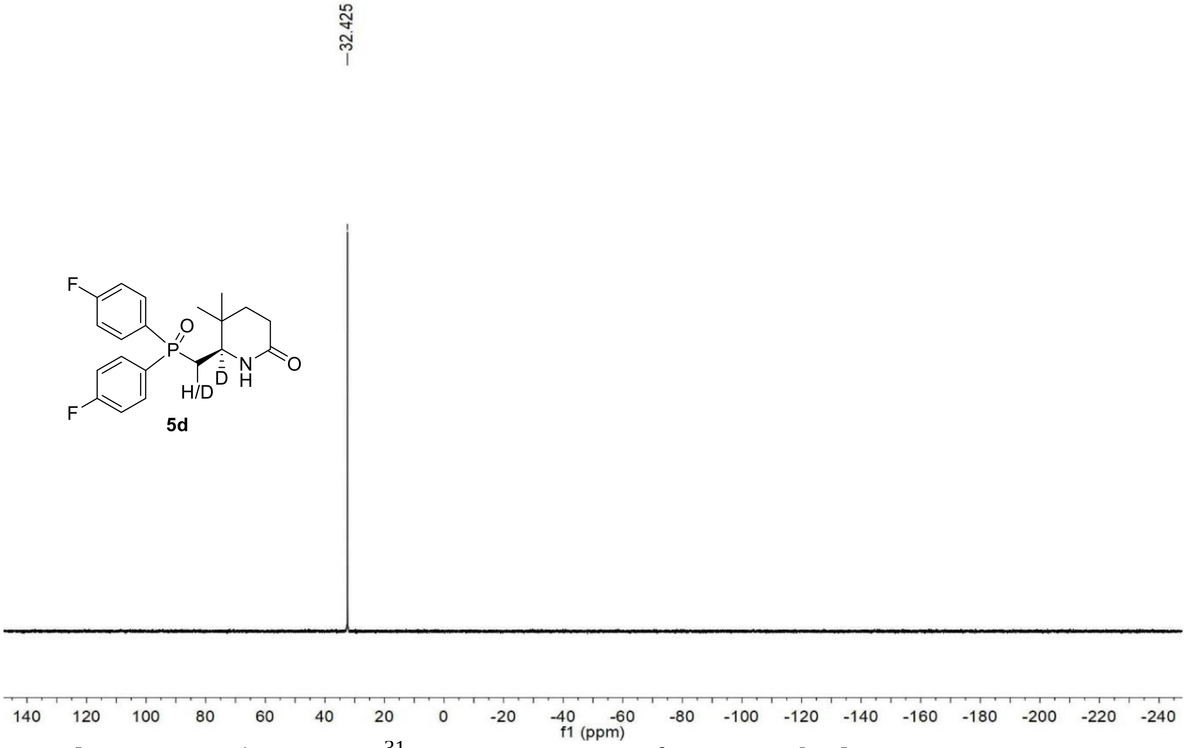

Supplementary Figure 201. ^{31}P NMR spectrum of compound **5d**

^{19}F NMR (376 MHz, room temperature, $CDCl_3$)

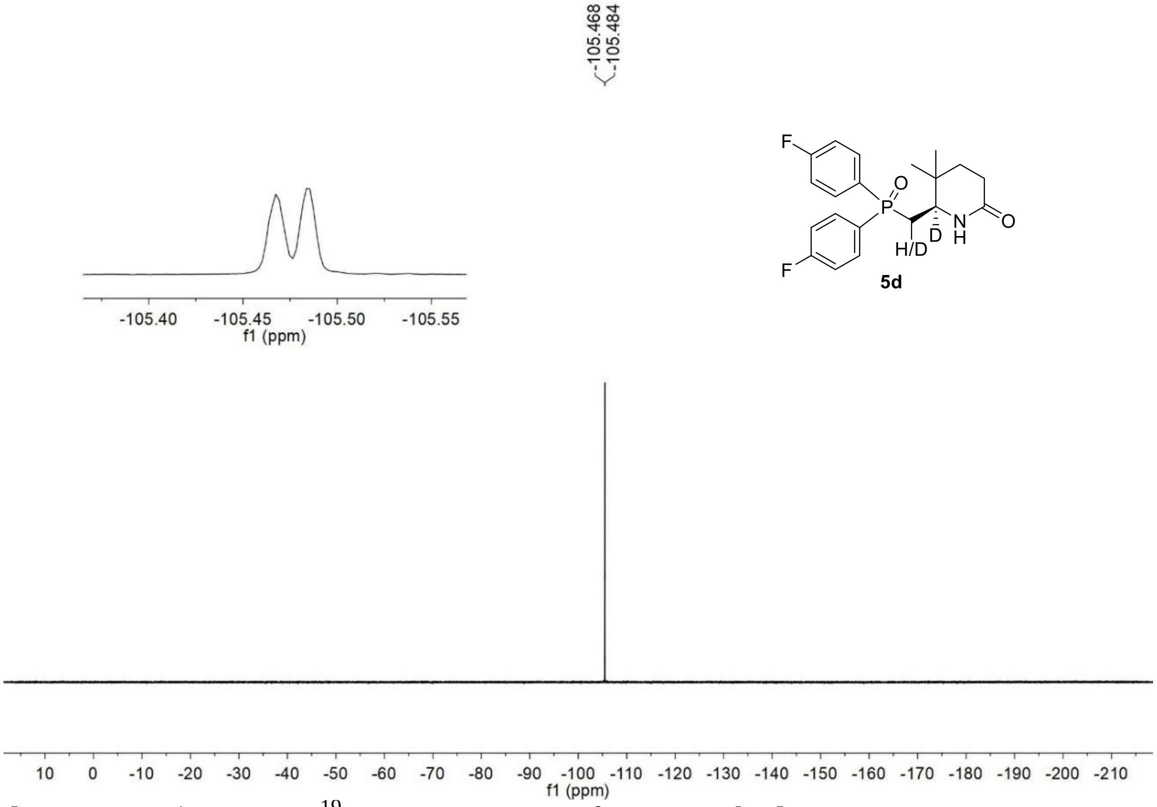

Supplementary Figure 202. ^{19}F NMR spectrum of compound **5d**

HPLC spectrum of racemic **5d**

Data File	: (42-racemic)SQL-4-256-1.lcd
Method File	: 2AS-H-80-0.5-214.lcm
Date Processed	: 7/17/2021 12:36:05 PM

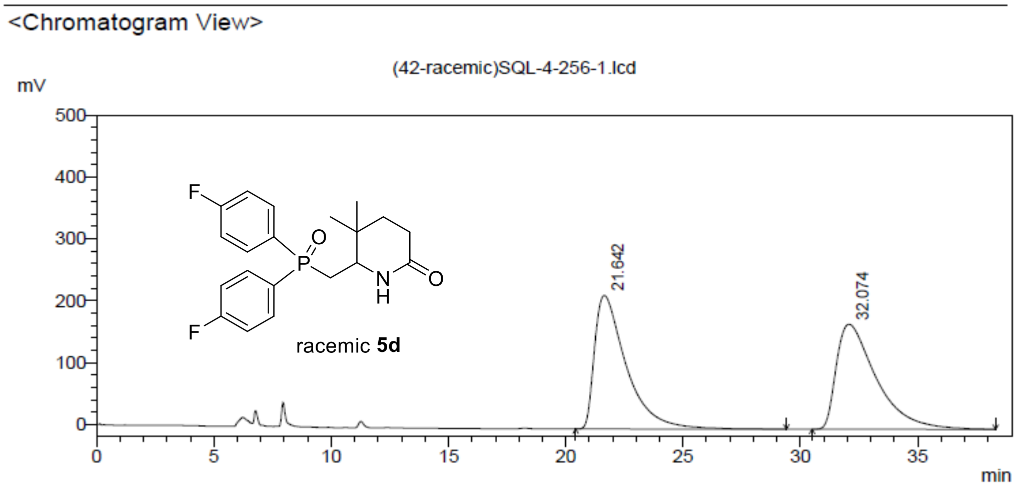

<Data Analysis>

DetA 214nm

Pesk #	Ret. Time	Height	Area	Area%
1	21.642	215870	20389489	50.278
2	32.074	169677	20164268	49.722
Total		385546	40553757	100.000

Supplementary Figure 203. HPLC spectrum of racemic **5d**

HPLC spectrum of **5d**

Data File	: (42-chiral)SQL-4-301.lcd
Method File	: 2AS-H-80-0.5-214.lcm
Date Processed	: 7/17/2021 12:36:24 PM

<Chromatogram View>

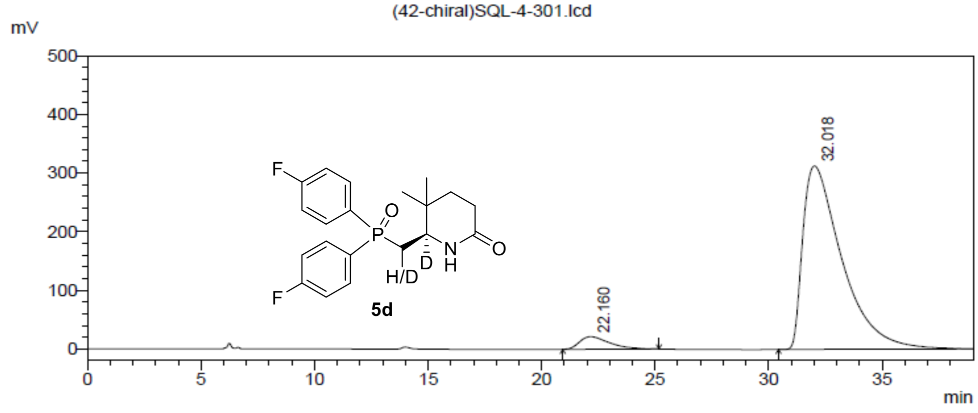

<Data Analysis>

DetA 214nm

Pesk #	Ret. Time	Height	Area	Area%
1	22.160	21547	1977618	4.993
2	32.018	312869	37630419	95.007
Total		334416	39608037	100.000

Supplementary Figure 204. HPLC spectrum of **5d**

^{1}H NMR (400 MHz, room temperature, $CDCl_3$)

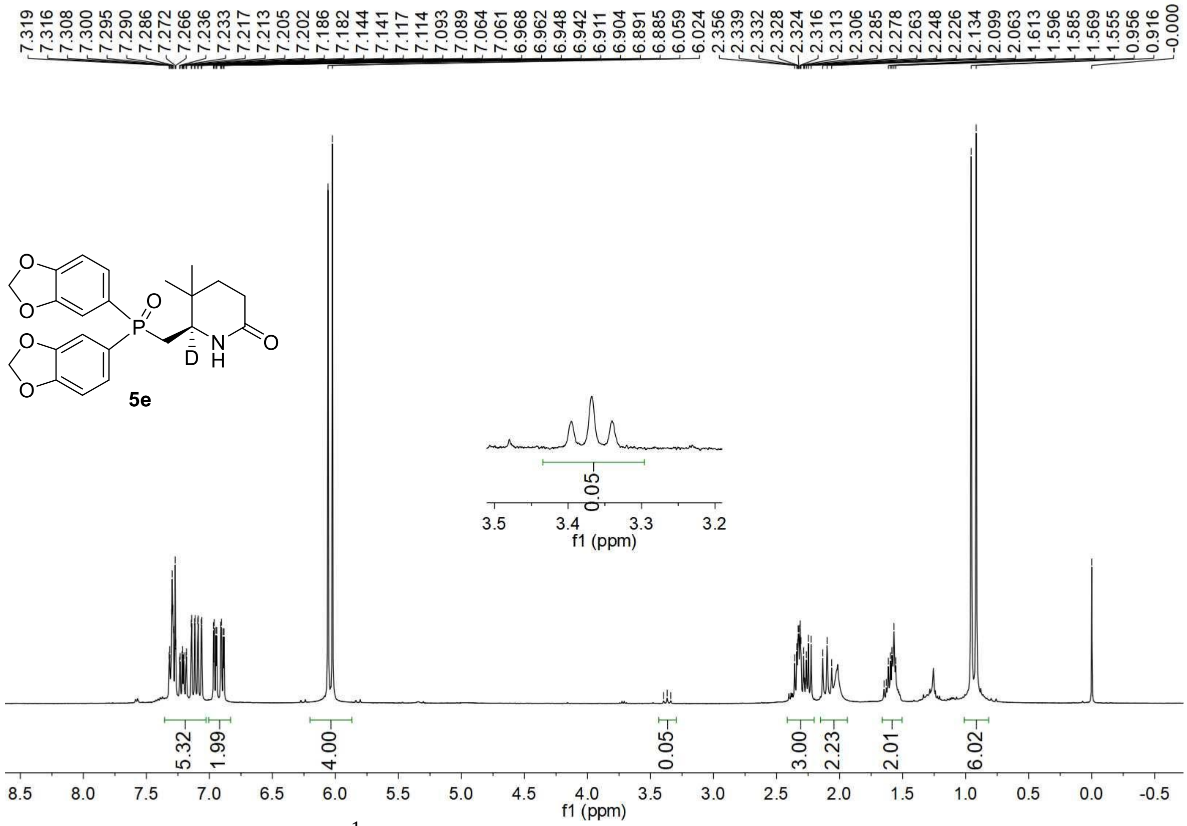

Supplementary Figure 205. ^{1}H NMR spectrum of compound **5e**

^{13}C NMR (100 MHz, room temperature, $CDCl_3$)

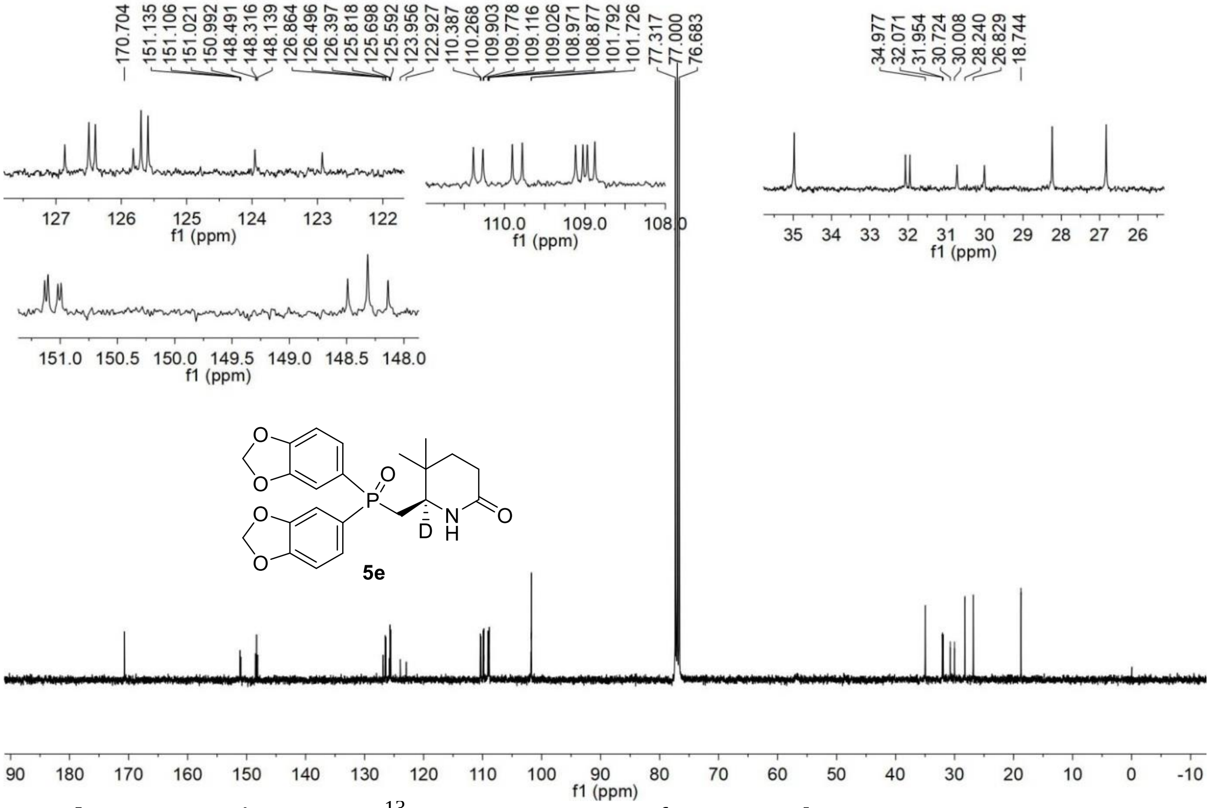

Supplementary Figure 206. ^{13}C NMR spectrum of compound **5e**

^{31}P NMR (162 MHz, room temperature, $CDCl_3$)

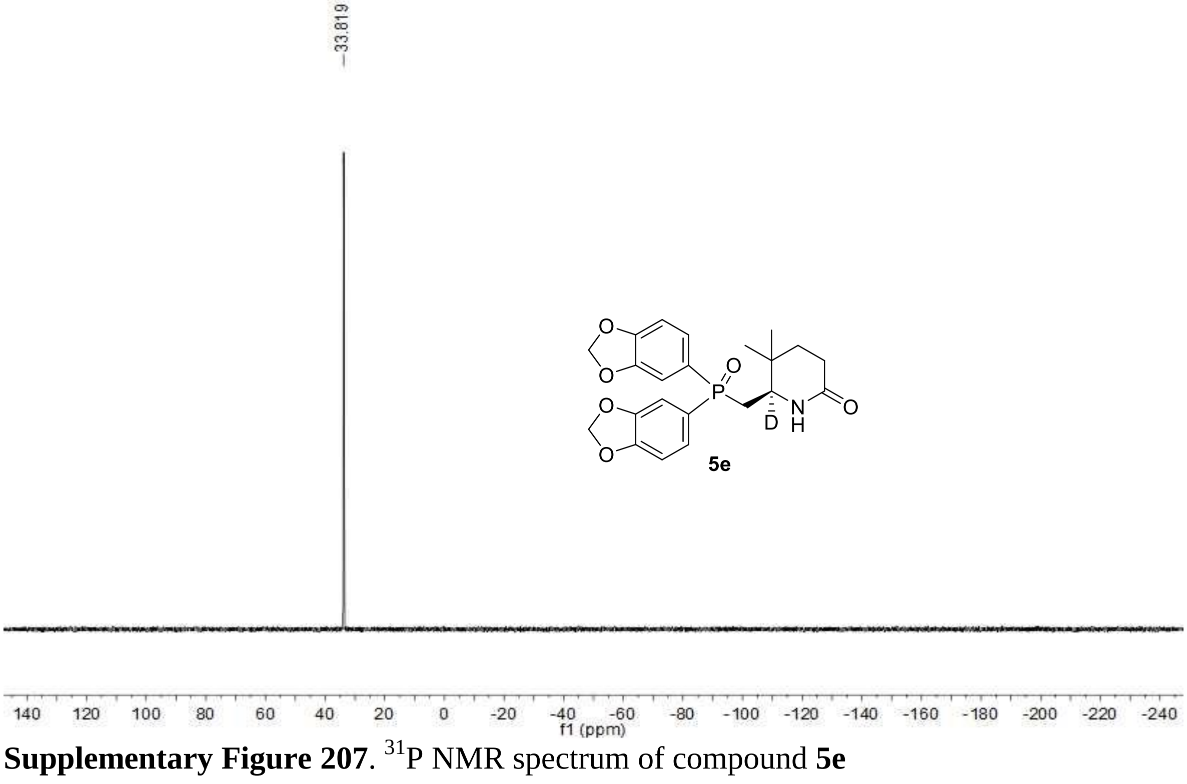

Supplementary Figure 207. ^{31}P NMR spectrum of compound **5e**

HPLC spectrum of racemic **5e**

Data File	: (43-racemic)SQL-4-336-1.lcd
Method File	: 3AD-H-70-1-214.lcm
Date Processed	: 7/17/2021 12:36:52 PM

<Chromatogram View>

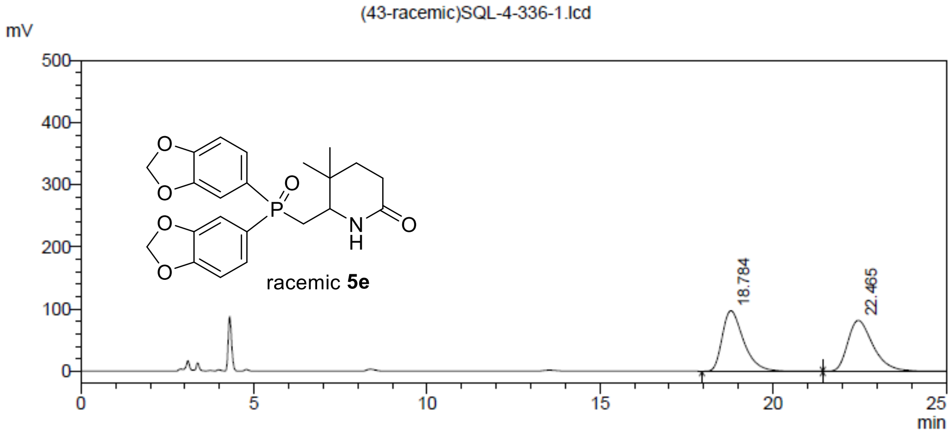

<Data Analysis>

DetA 214nm

Pesk #	Ret. Time	Height	Area	Area%
1	18.784	97534	4217447	49.993
2	22.465	82031	4218679	50.007
Total		179564	8436126	100.000

Supplementary Figure 208. HPLC spectrum of racemic **5e**

HPLC spectrum of **5e**

Data File	: (43-chiral)SQL-4-348-.lcd
Method File	: 3AD-H-70-1-214.lcm
Date Processed	: 7/17/2021 12:36:39 PM

<Chromatogram View>

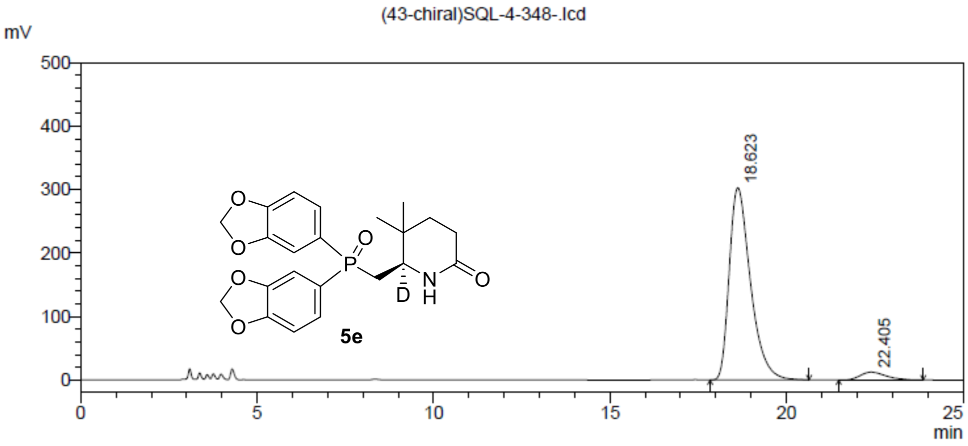

<Data Analysis>

DetA 214nm

Pesk #	Ret. Time	Height	Area	Area%
1	18.623	302727	12662082	95.239
2	22.405	12449	632928	4.761
Total		315176	13295010	100.000

Supplementary Figure 209. HPLC spectrum of **5e**

^{1}H NMR (400 MHz, room temperature, $CDCl_3$)

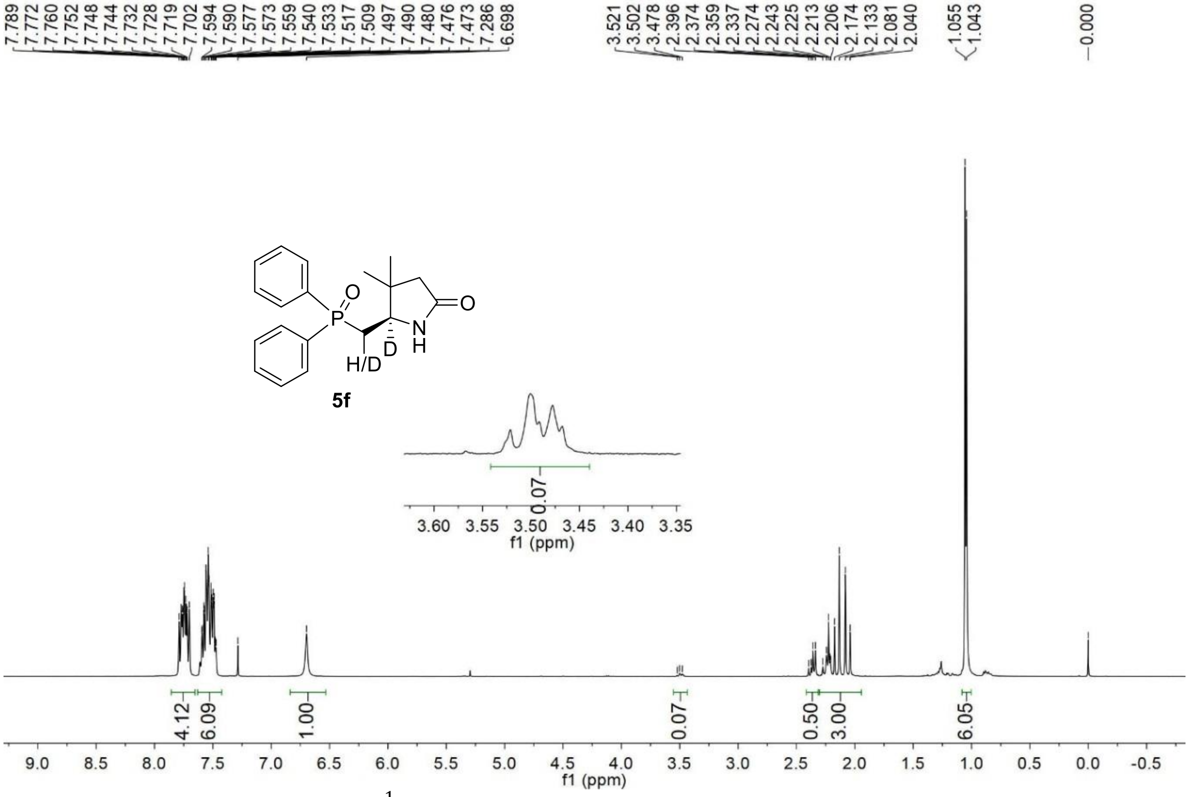

Supplementary Figure 210. ^{1}H NMR spectrum of compound **5f**

^{13}C NMR (100 MHz, room temperature, $CDCl_3$)

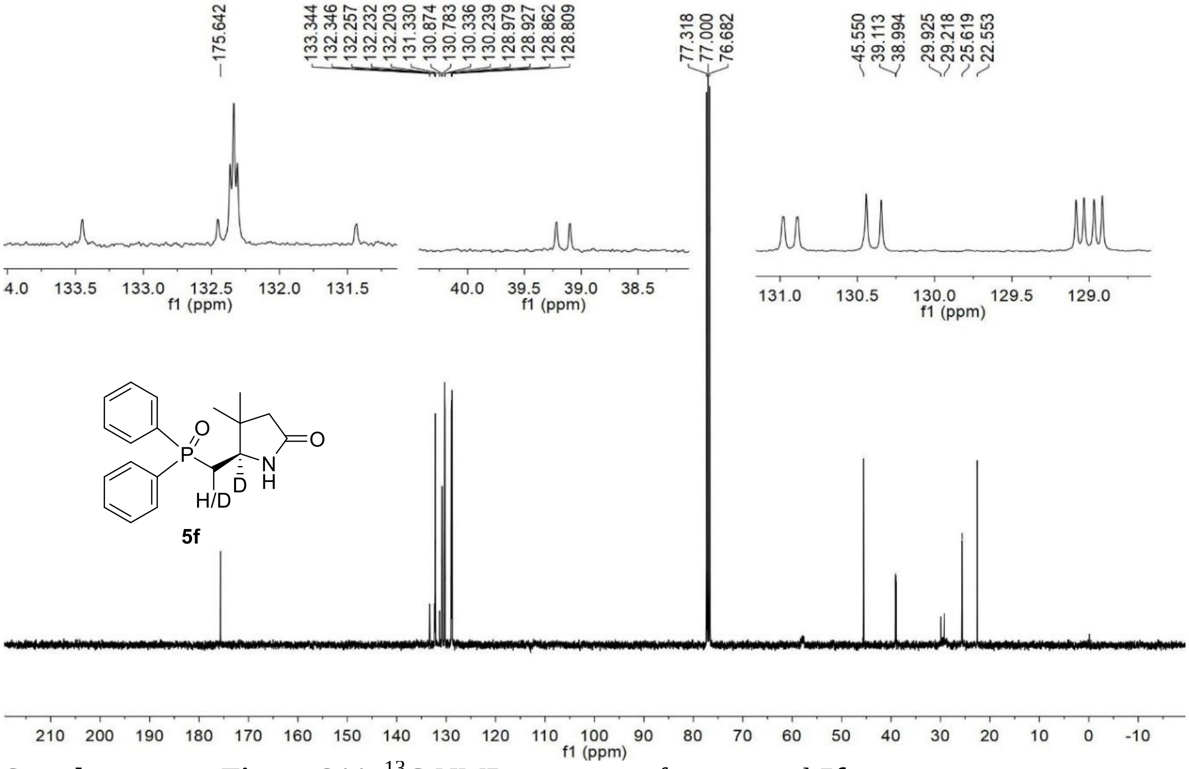

Supplementary Figure 211. ^{13}C NMR spectrum of compound **5f**

^{31}P NMR (162 MHz, room temperature, $CDCl_3$)

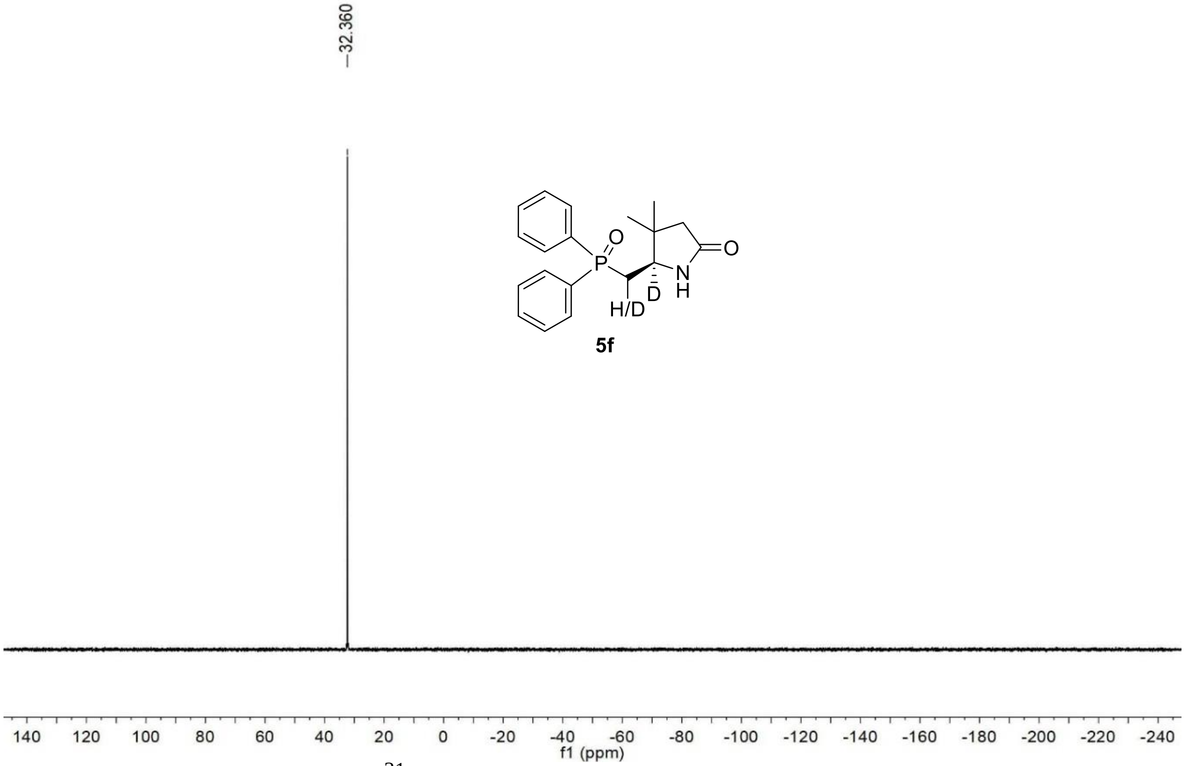

Supplementary Figure 212. ^{31}P NMR spectrum of compound **5f**

HPLC spectrum of racmic **5f**

Data File	: (45-racemic)SQL-4-257-1.lcd
Method File	: 3AD-H-60-0.5-214.lcm
Date Processed	: 7/17/2021 12:37:54 PM

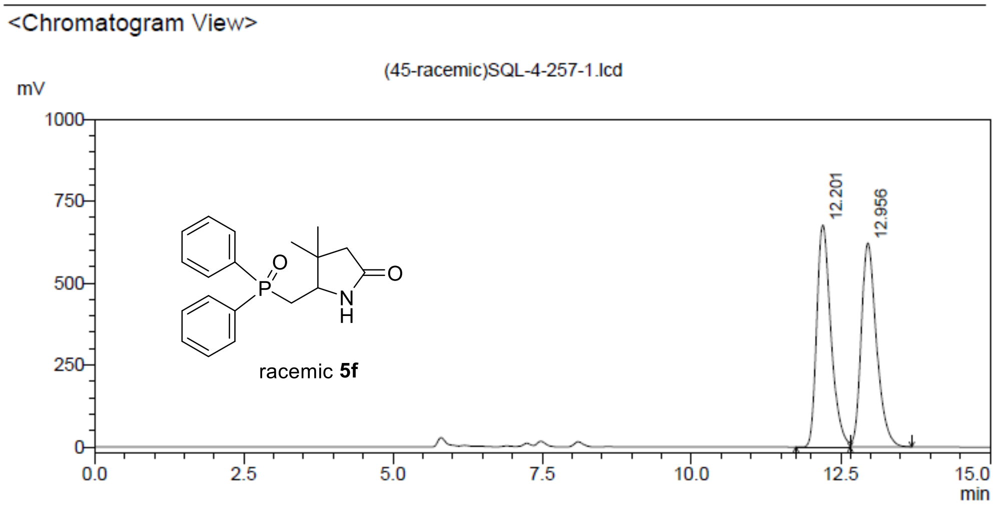

<Data Analysis>

DetA 214nm

Pesk #	Ret. Time	Height	Area	Area%
1	12.201	677394	11212447	50.272
2	12.956	622130	11090952	49.728
Total		1299525	22303399	100.000

Supplementary Figure 213. HPLC spectrum of racemic **5f**

HPLC spectrum of **5f**

Data File : (45-chiral) SQL-4-288.lcd
Method File : 3AD-H-60-0.5-214.lcm
Date Processed : 7/17/2021 12:37:41 PM

<Chromatogram View>

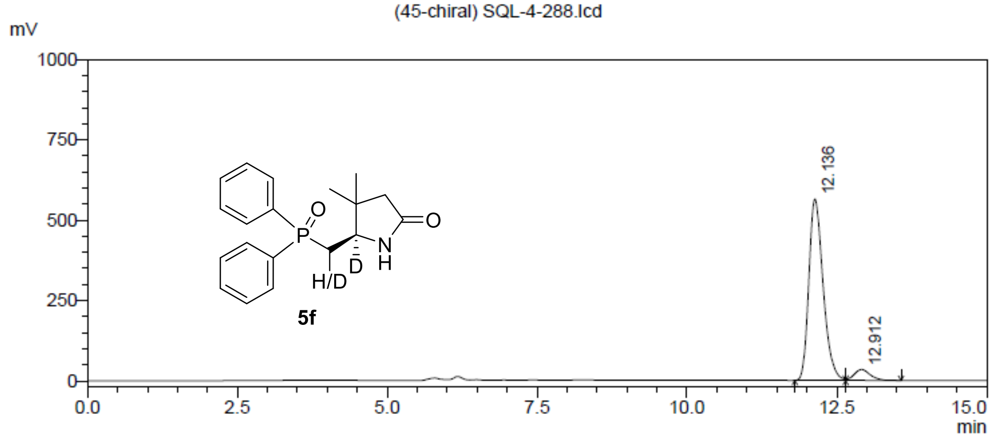

<Data Analysis>

DetA 214nm

Pesk #	Ret. Time	Height	Area	Area%
1	12.136	564140	9339706	93.695
2	12.912	33813	628468	6.305
Total		597953	9968173	100.000

Supplementary Figure 214. HPLC spectrum of **5f**

^{1}H NMR (400 MHz, room temperature, $CDCl_3$)

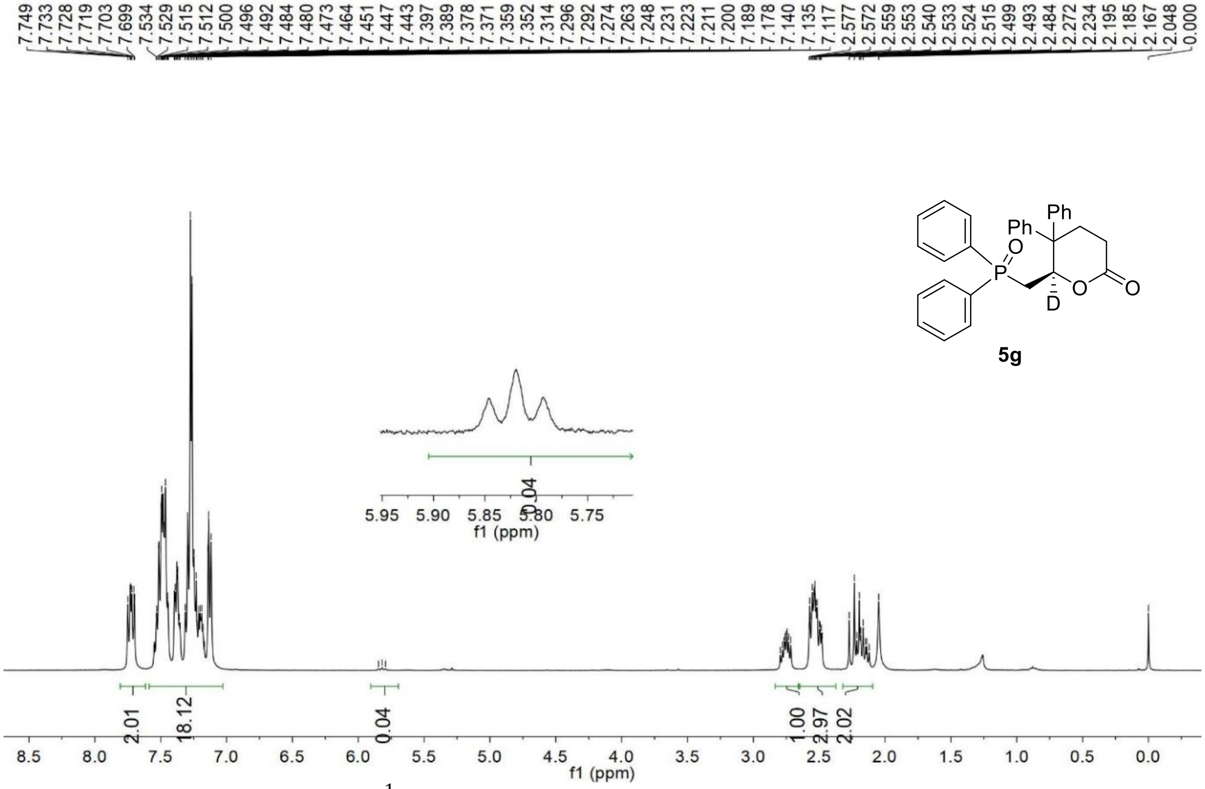

Supplementary Figure 215. ^{1}H NMR spectrum of compound **5g**

^{13}C NMR (100 MHz, room temperature, $CDCl_3$)

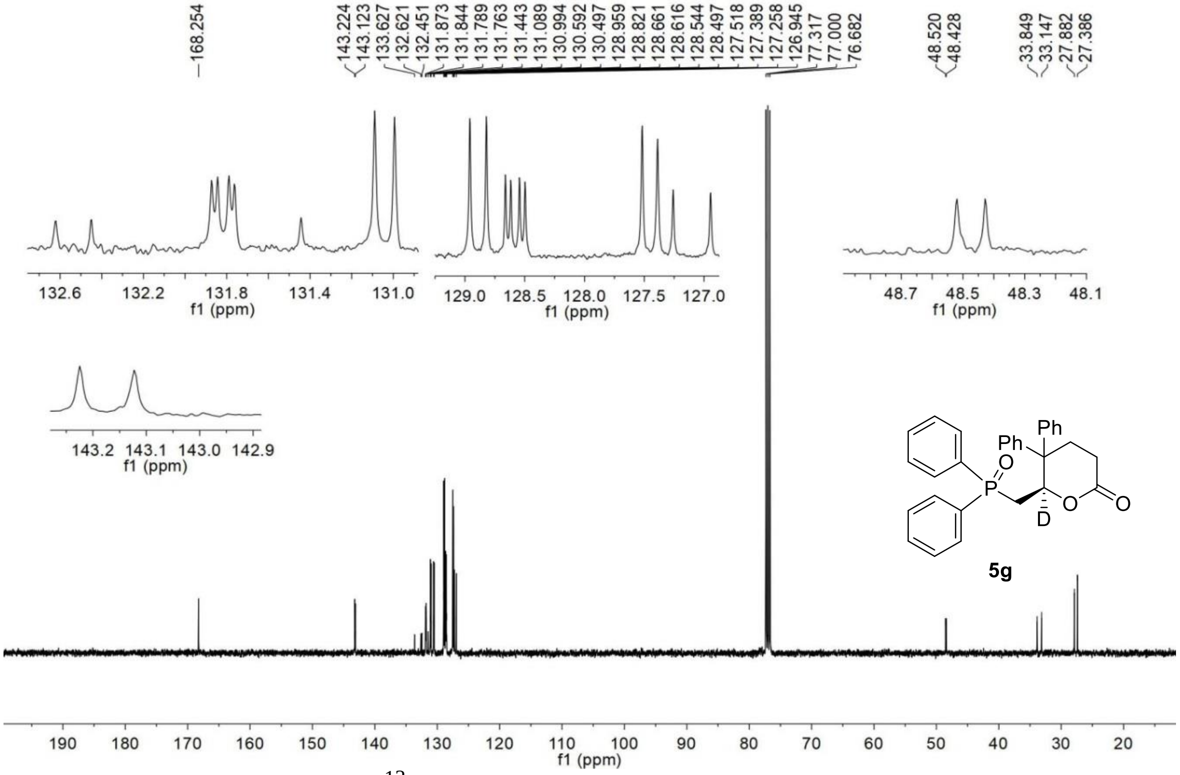

Supplementary Figure 216. ^{13}C NMR spectrum of compound **5g**

^{31}P NMR (162 MHz, room temperature, $CDCl_3$)

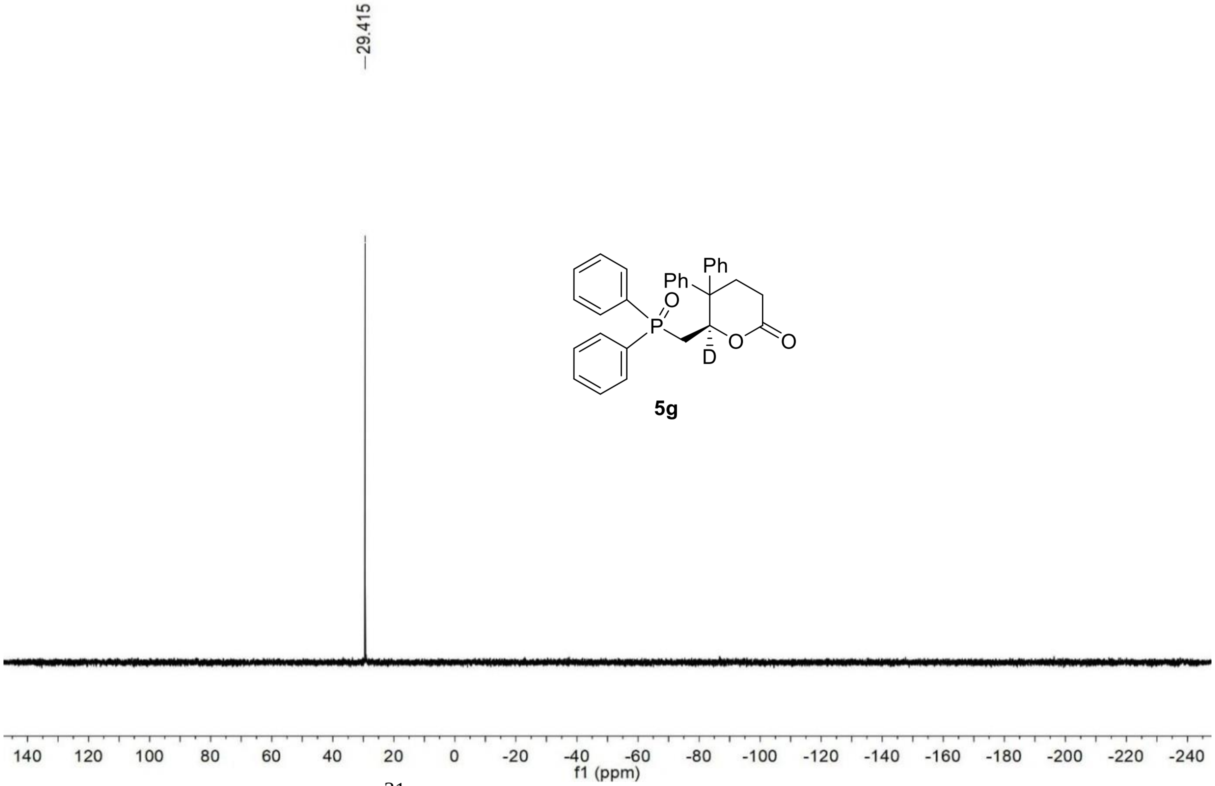

Supplementary Figure 217. ^{31}P NMR spectrum of compound **5g**

HPLC spectrum of racemic **5g**

Data File	: 46-RACEMIC-SQL-4-289-1.lcd
Method File	: 3AD-H-70-1-214.lcm
Date Processed	: 7/17/2021 12:40:21 PM

<Chromatogram View>

46-RACEMIC-SQL-4-289-1.lcd

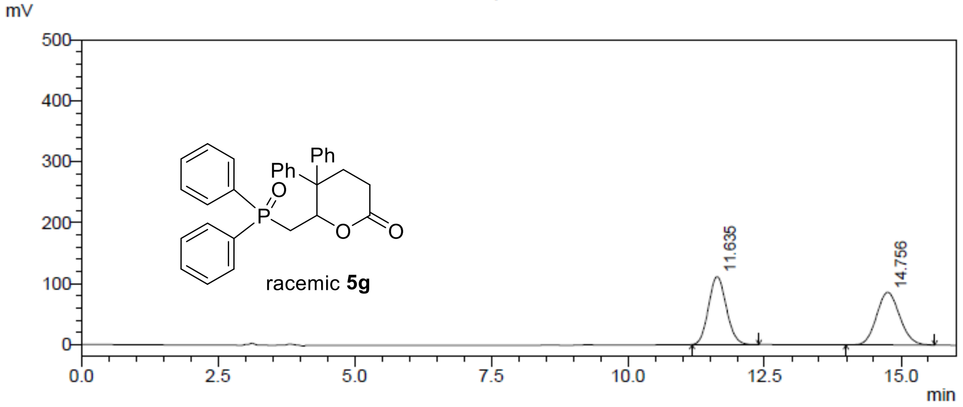

<Data Analysis>

DetA 214nm

Pesk #	Ret. Time	Height	Area	Area%
1	11.635	111521	2530479	49.917
2	14.756	86225	2538888	50.083
Total		197746	5069366	100.000

Supplementary Figure 218. HPLC spectrum of racemic **5g**

HPLC spectrum of **5g**

Data File	: 46-CHIRAL-SQL-4-287-4.lcd
Method File	: 3AD-H-70-1-214.lcm
Date Processed	: 7/17/2021 12:38:56 PM

<Chromatogram View>

46-CHIRAL-SQL-4-287-4.lcd

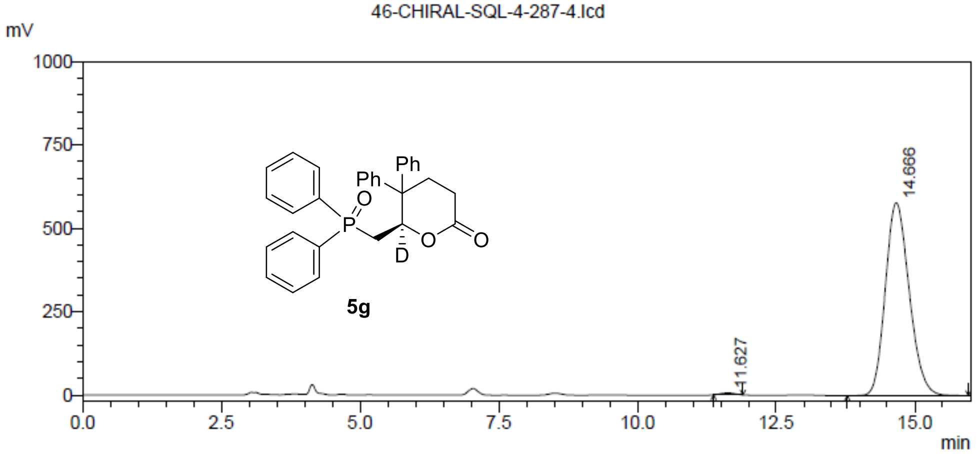

<Data Analysis>

DetA 214nm

Pesk #	Ret. Time	Height	Area	Area%
1	11.627	6014	105956	0.608
2	14.666	578274	17333080	99.392
Total		584288	17439035	100.000

Supplementary Figure 219. HPLC spectrum of **5g**

^{1}H NMR (400 MHz, room temperature, $CDCl_3$)

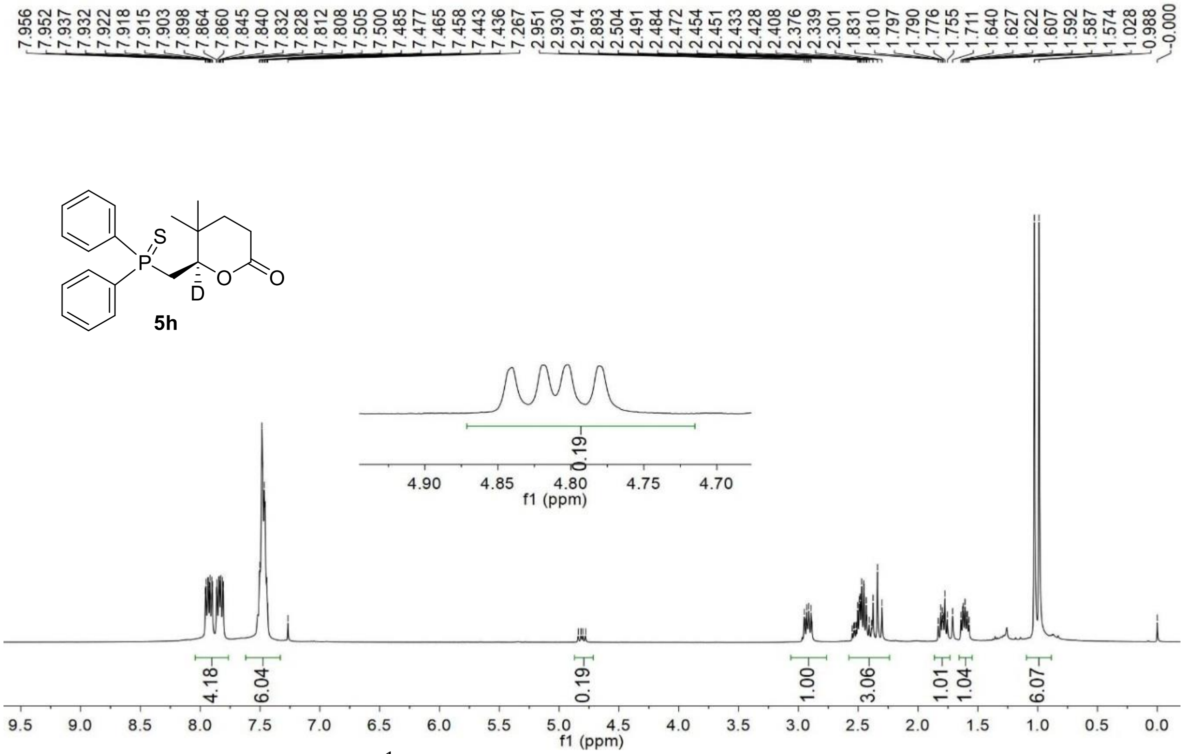

Supplementary Figure 220. ^{1}H NMR spectrum of compound **5h**

^{13}C NMR (100 MHz, room temperature, $CDCl_3$)

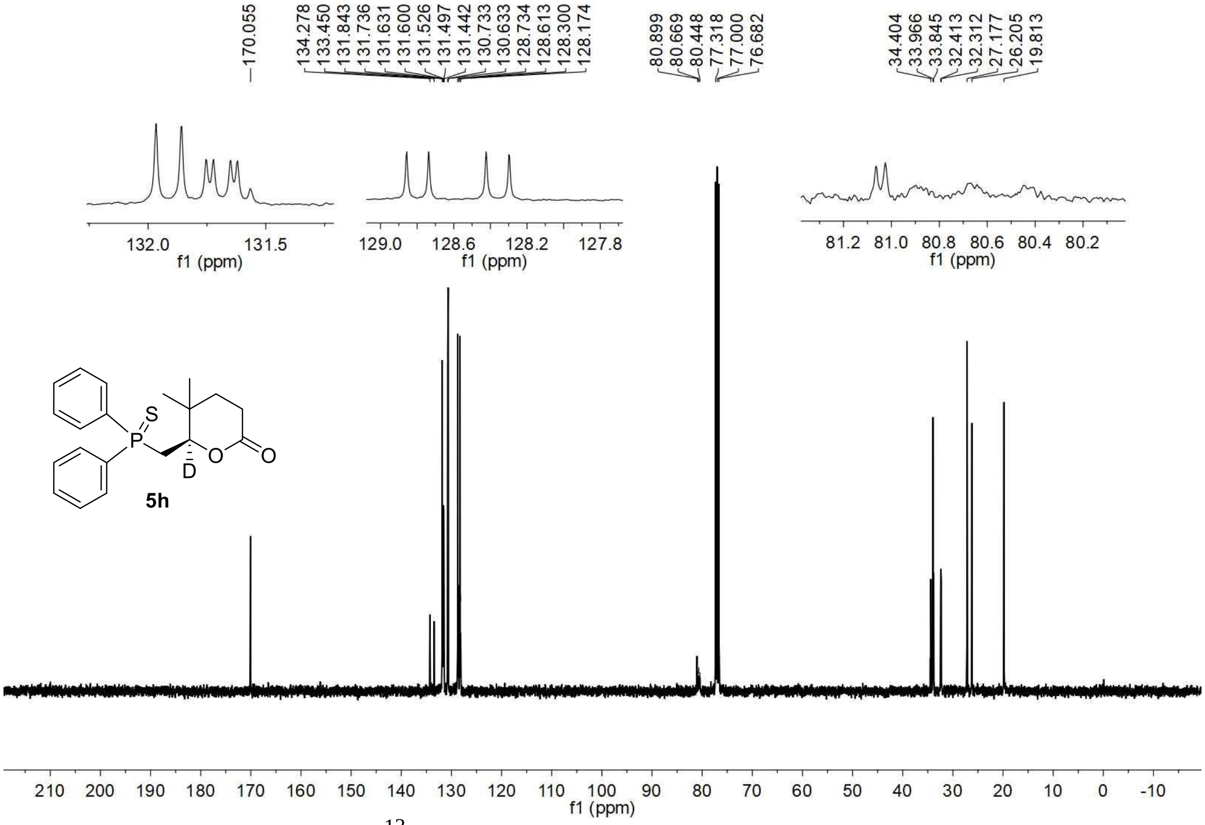

Supplementary Figure 221. ^{13}C NMR spectrum of compound **5h**

^{31}P NMR (162 MHz, room temperature, $CDCl_3$)

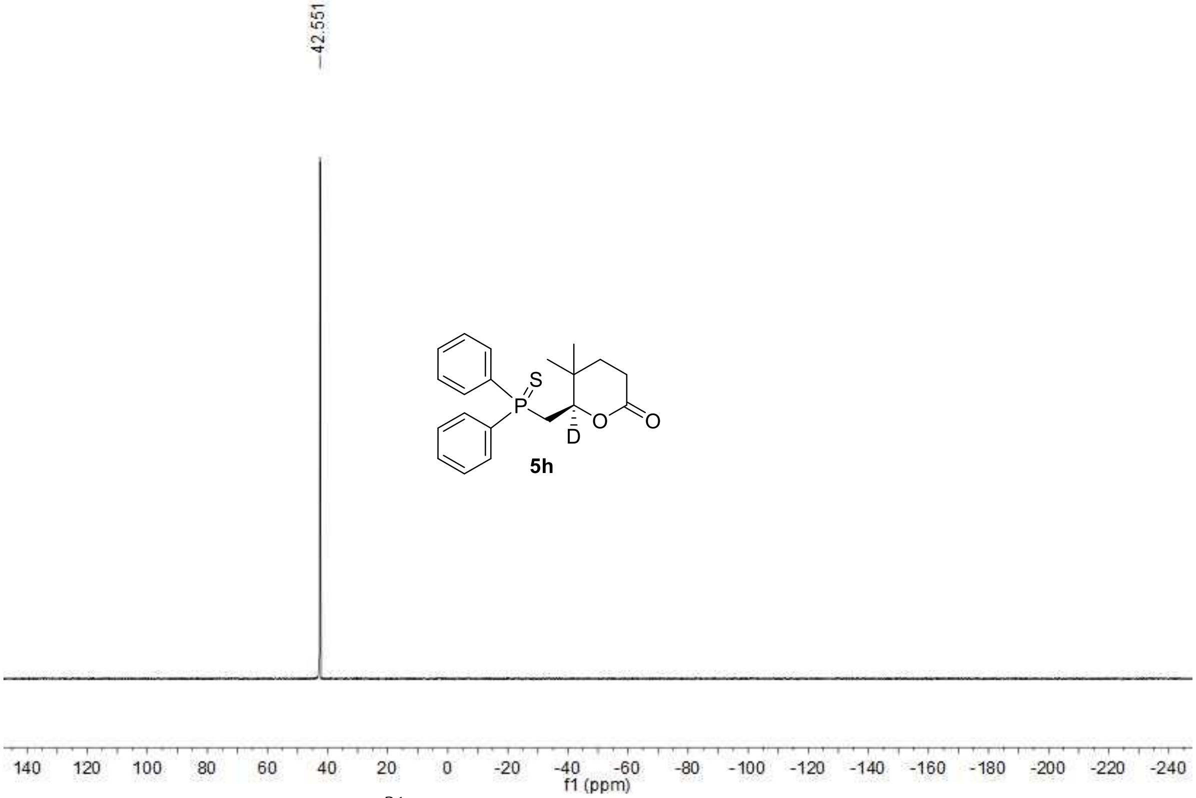

Supplementary Figure 222. ^{31}P NMR spectrum of compound **5h**

HPLC spectrum of racemic **5h**

Data File : SQL-4-293-1.lcd
Method File : 4OD-H-88-12-1-214.lcm
Date Processed : 12/29/2020 10:09:02 AM

<Chromatogram View>

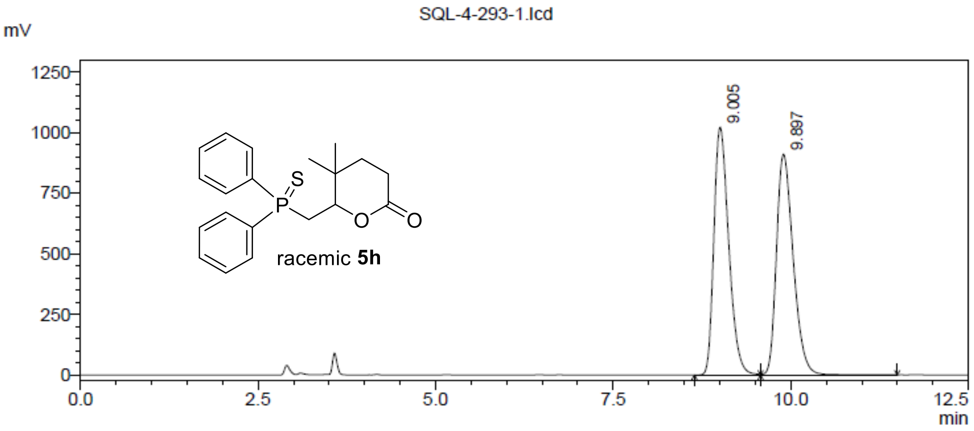

<Data Analysis>

DetA 214nm

Pesk #	Ret. Time	Height	Area	Area%
1	9.005	1021626	14843963	49.842
2	9.897	910249	14938074	50.158
Total		1931875	29782037	100.000

Supplementary Figure 223. HPLC spectrum of racemic **5h**

HPLC spectrum of **5h**

Data File : SQL-4-293-2-1.lcd
Method File : 4OD-H-88-12-1-214.lcm
Date Processed : 12/29/2020 10:48:54 AM

<Chromatogram View>

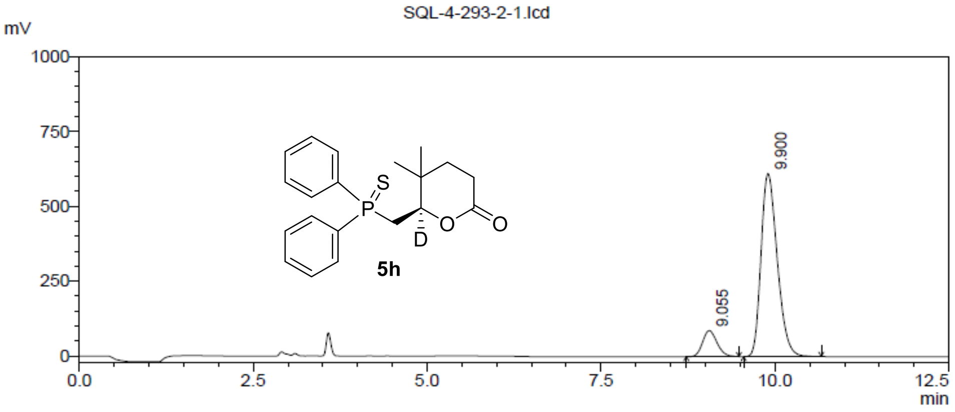

<Data Analysis>

DetA 214nm

Pesk #	Ret. Time	Height	Area	Area%
1	9.055	86780	1251077	11.206
2	9.900	610785	9912913	88.794
Total		697566	11163990	100.000

Supplementary Figure 224. HPLC spectrum of **5h**

^{1}H NMR (400 MHz, room temperature, $CDCl_3$)

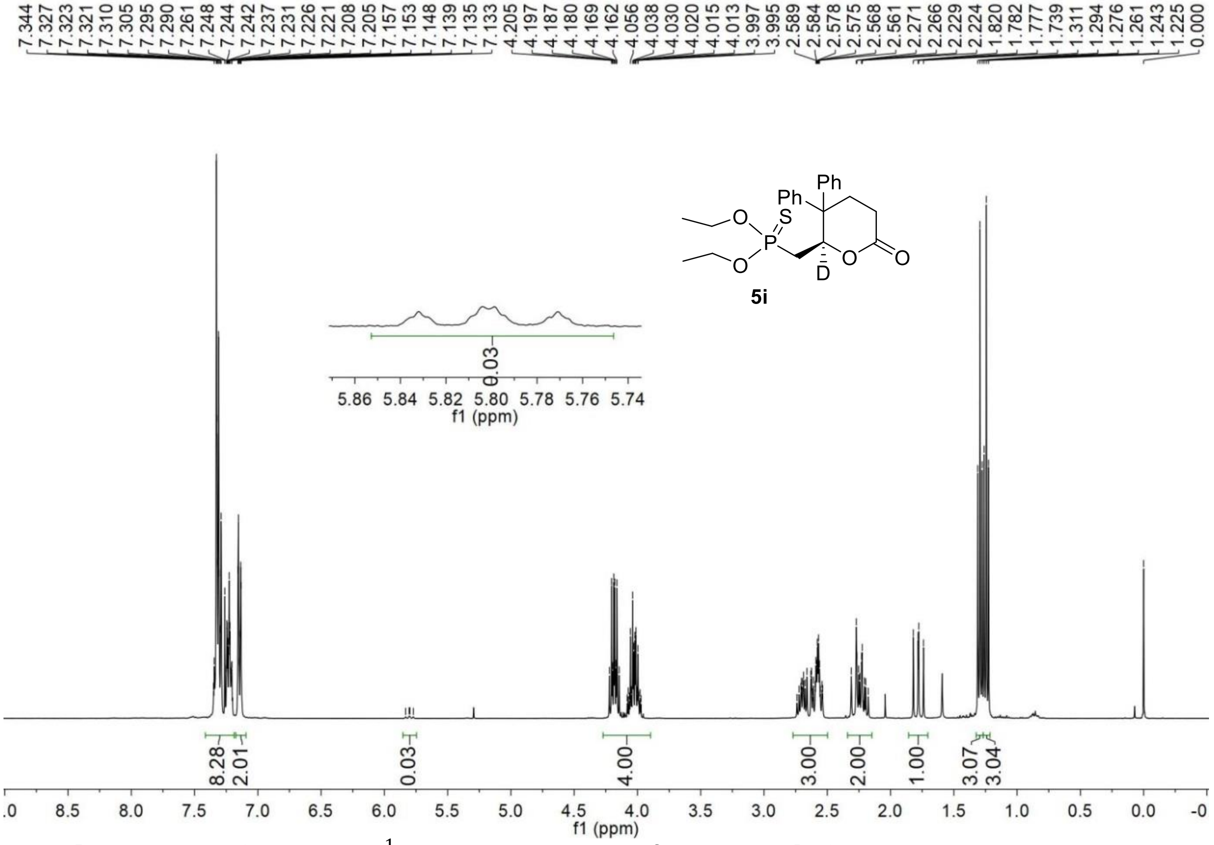

Supplementary Figure 225. ^{1}H NMR spectrum of compound **5i**

^{13}C NMR (100 MHz, room temperature, $CDCl_3$)

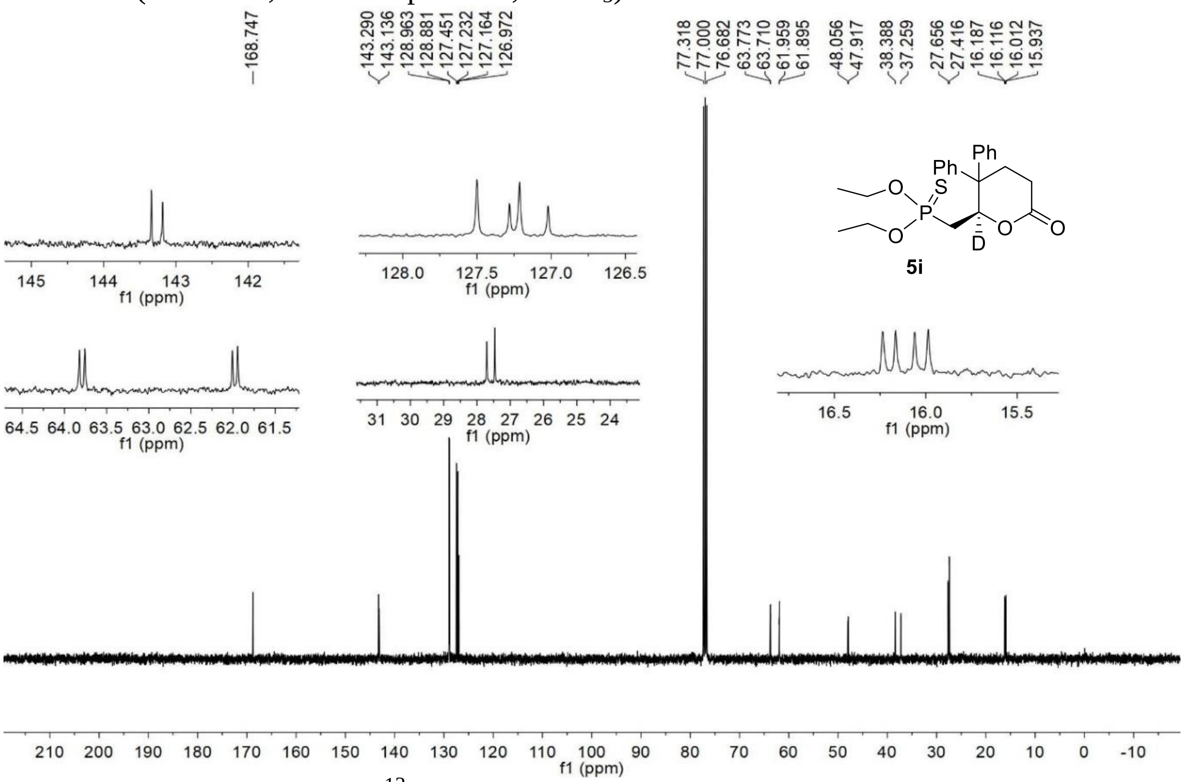

Supplementary Figure 226. ^{13}C NMR spectrum of compound **5i**

^{31}P NMR (162 MHz, room temperature, $CDCl_3$)

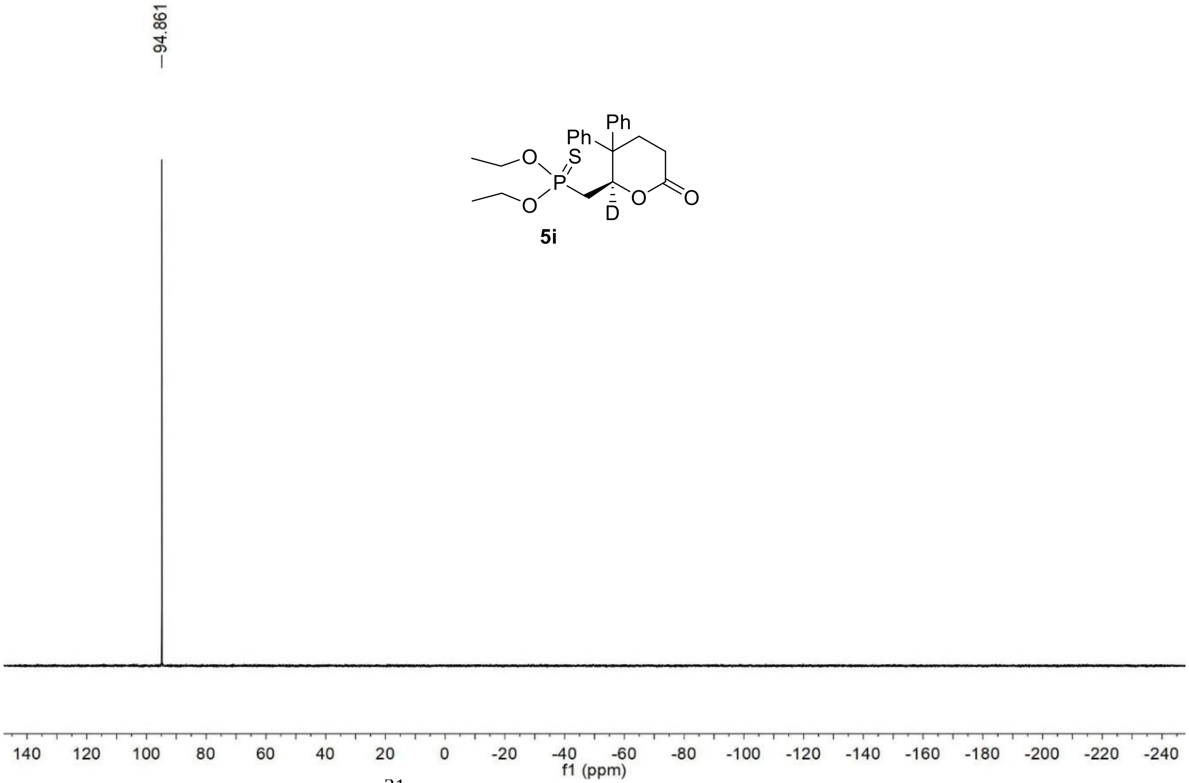

Supplementary Figure 227. ^{31}P NMR spectrum of compound **5i**

HPLC spectrum of racemic **5i**

Data File : (47-racemic)SQL-4-311-1.lcd
Method File : 6IC-H-80-0.5-214.lcm
Date Processed : 7/17/2021 12:41:00 PM

<Chromatogram View>

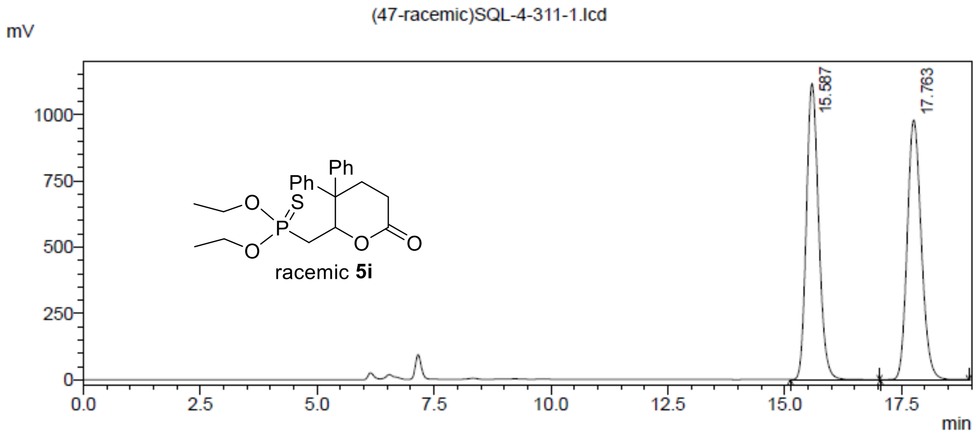

<Data Analysis>

DetA 214nm

Pesk #	Ret. Time	Height	Area	Area%
1	15.587	1115972	20368157	49.843
2	17.763	979003	20496395	50.157
Total		2094975	40864552	100.000

Supplementary Figure 228. HPLC spectrum of racemic **5i**

HPLC spectrum of **5i**

Data File : (47-chiral)SQL-4-311-2.lcd
Method File : 6IC-H-80-0.5-214.lcm
Date Processed : 7/17/2021 12:40:39 PM

<Chromatogram View>

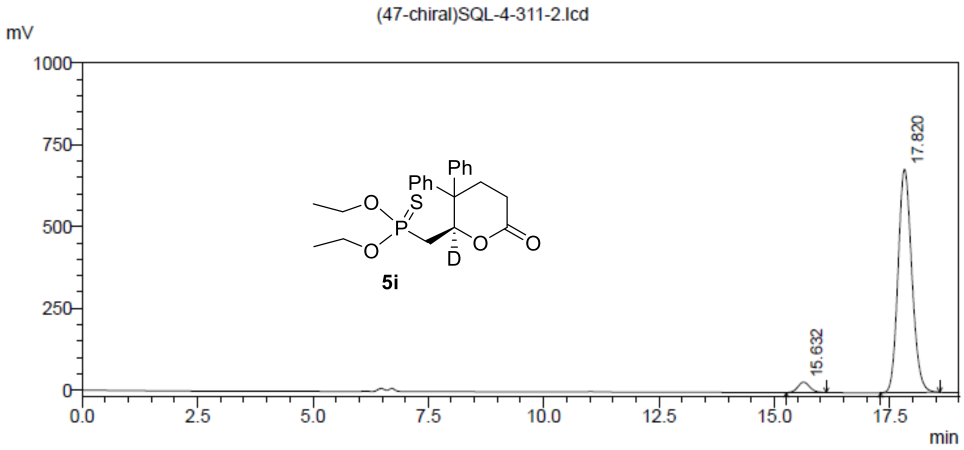

<Data Analysis>

DetA 214nm

Pesk #	Ret. Time	Height	Area	Area%
1	15.632	31613	567133	3.848
2	17.820	681972	14170277	96.152
Total		713585	14737410	100.000

Supplementary Figure 229. HPLC spectrum of **5i**

^{1}H NMR (400 MHz, room temperature, $CDCl_3$)

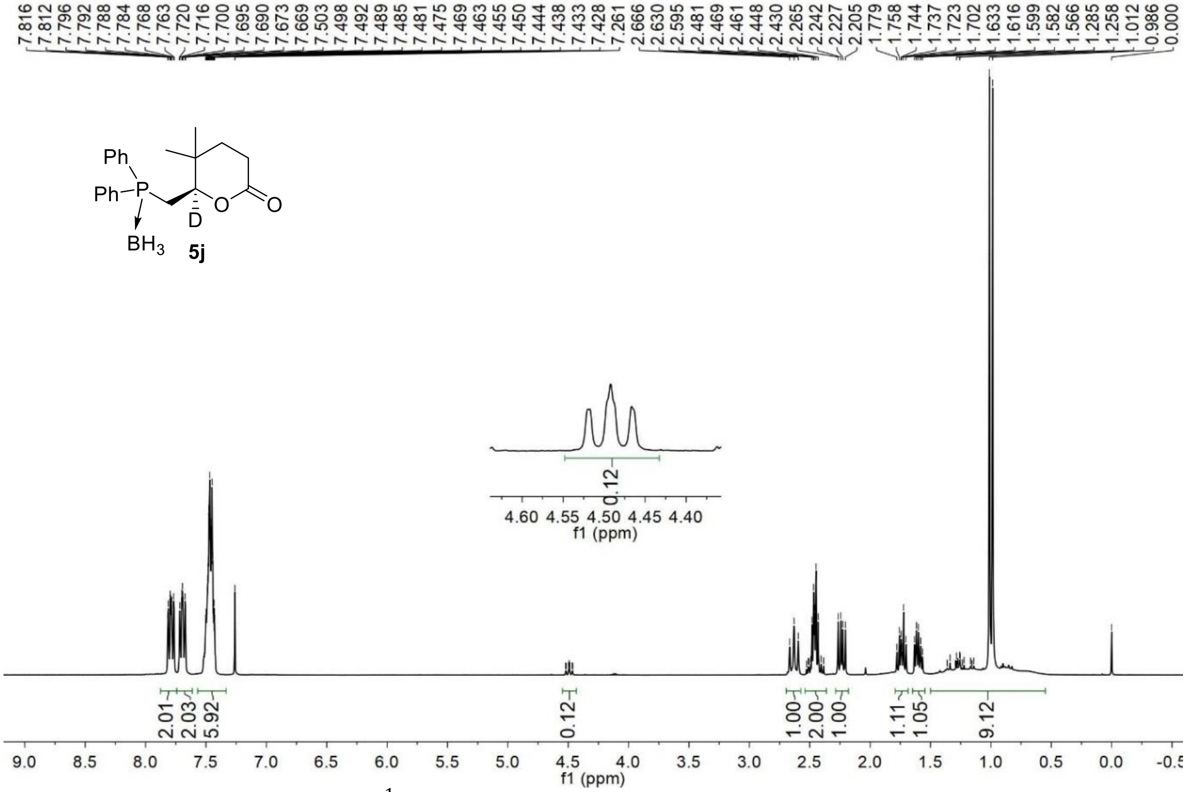

Supplementary Figure 230. ^{1}H NMR spectrum of compound **5j**

^{13}C NMR (100 MHz, room temperature, $CDCl_3$)

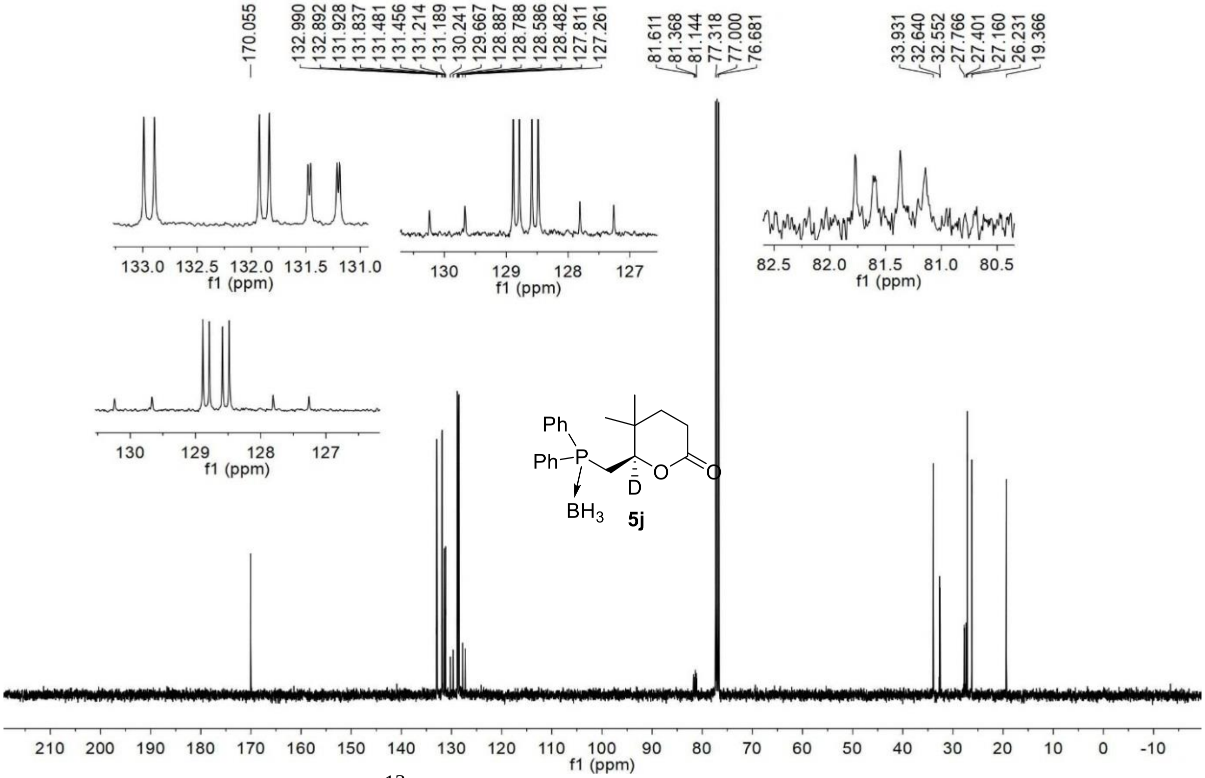

Supplementary Figure 231. ^{13}C NMR spectrum of compound **5j**

^{31}P NMR (162 MHz, room temperature, $CDCl_3$)

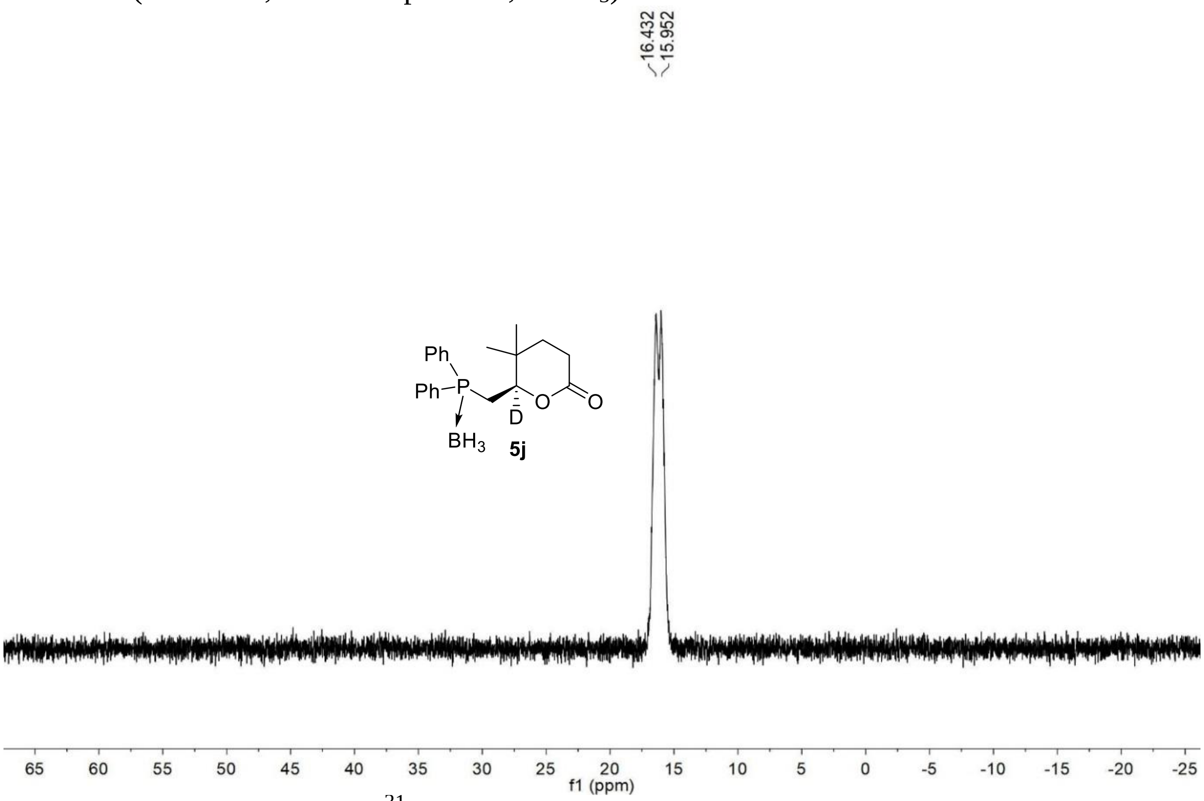

Supplementary Figure 232. ^{31}P NMR spectrum of compound **5j**

^{11}B NMR (128 MHz, room temperature, $CDCl_3$)

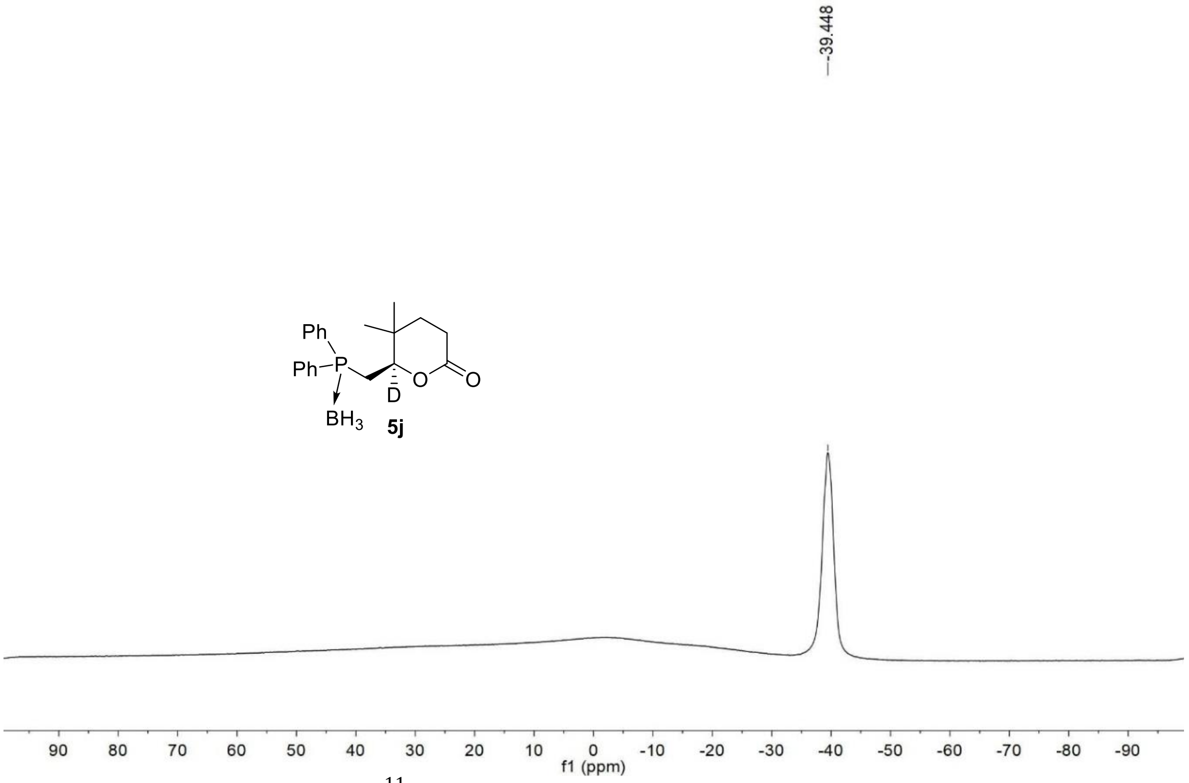

Supplementary Figure 233. ^{11}B NMR spectrum of compound **5j**

HPLC spectrum of racemic **5j**

Data File	: 48-sql-6-21-1racemic.lcd
Method File	: 3AD-H-70-0.8-214.lcm
Date Processed	: 8/8/2021 1:50:08 PM

<Chromatogram View>

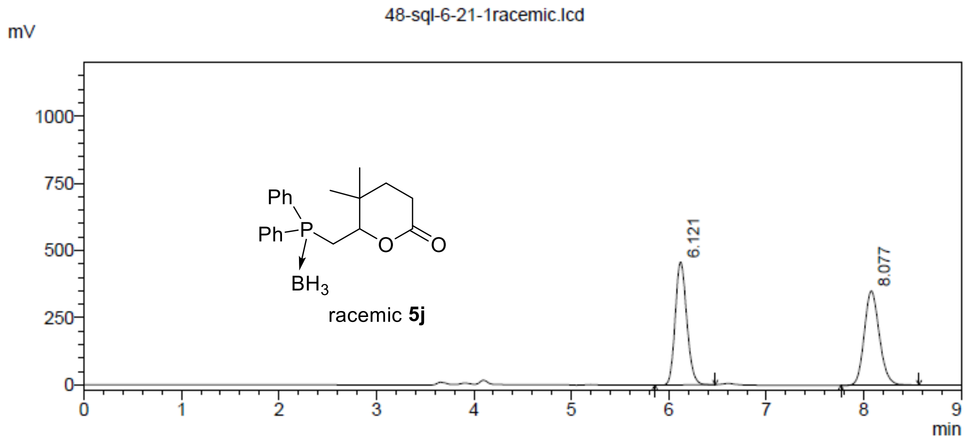

<Data Analysis>

Detector A 214nm

Pesk #	Ret. Time	Height	Area	Area%
1	6.121	457298	3768535	50.019
2	8.077	350424	3765707	49.981
Total		807723	7534242	100.000

Supplementary Figure 234. HPLC spectrum of racemic **5j**

HPLC spectrum of **5j**

Data File	: 48-SQL-6-21-2chiral.lcd
Method File	: 3AD-H-70-0.8-214.lcm
Date Processed	: 8/8/2021 1:50:54 PM

<Chromatogram View>

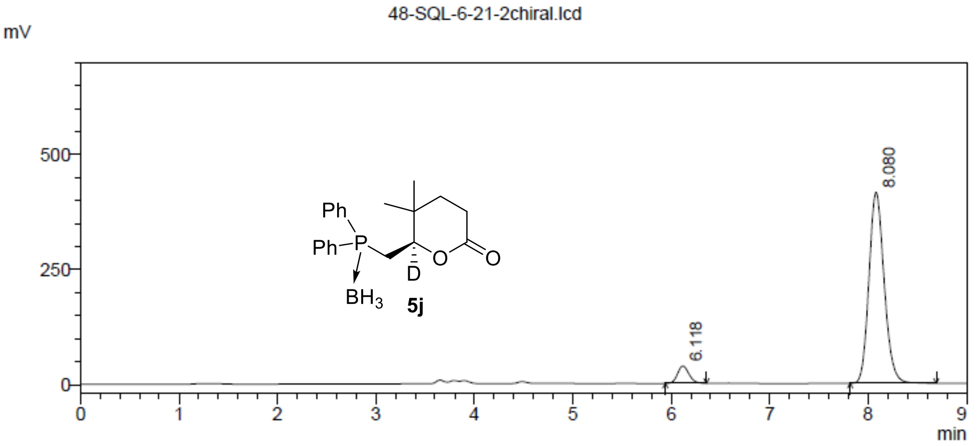

<Data Analysis>

Detector A 214nm

Pesk #	Ret. Time	Height	Area	Area%
1	6.118	37678	300613	6.303
2	8.080	414892	4468382	93.697
Total		452570	4768994	100.000

Supplementary Figure 235. HPLC spectrum of **5j**

^{1}H NMR (400 MHz, room temperature, $CDCl_3$)

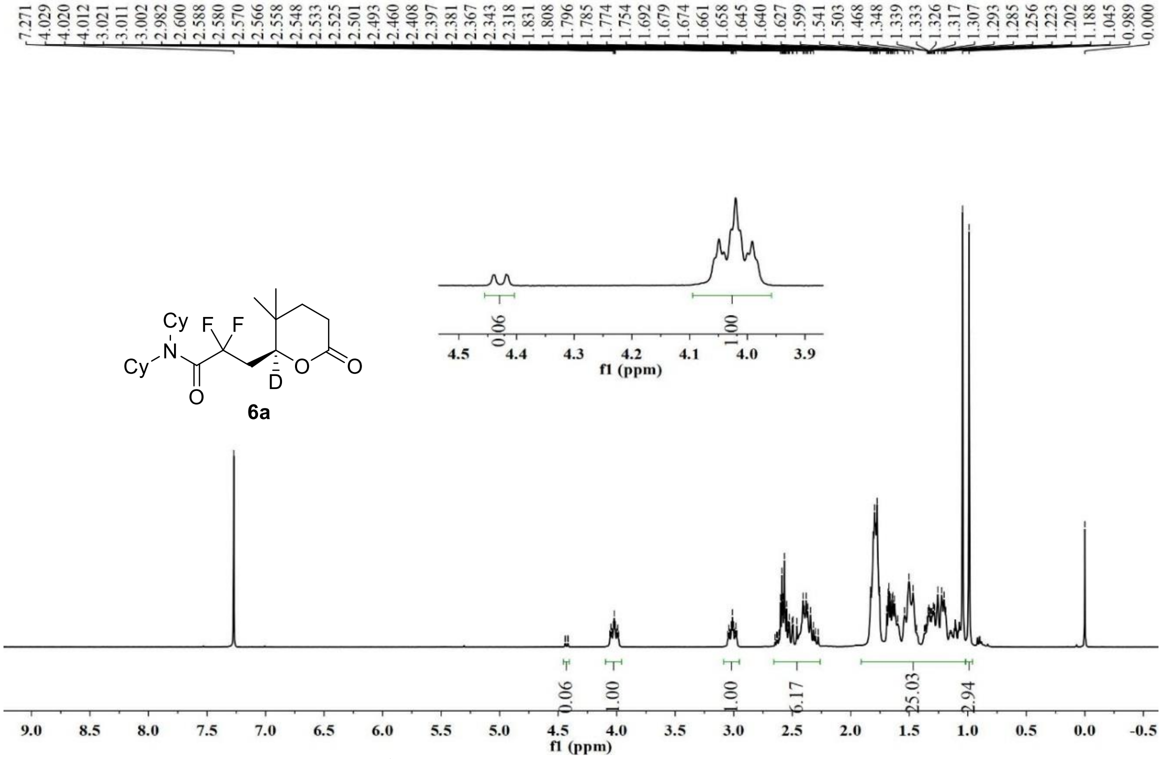

Supplementary Figure 236. ^{1}H NMR spectrum of compound **6a**

^{13}C NMR (100 MHz, room temperature, $CDCl_3$)

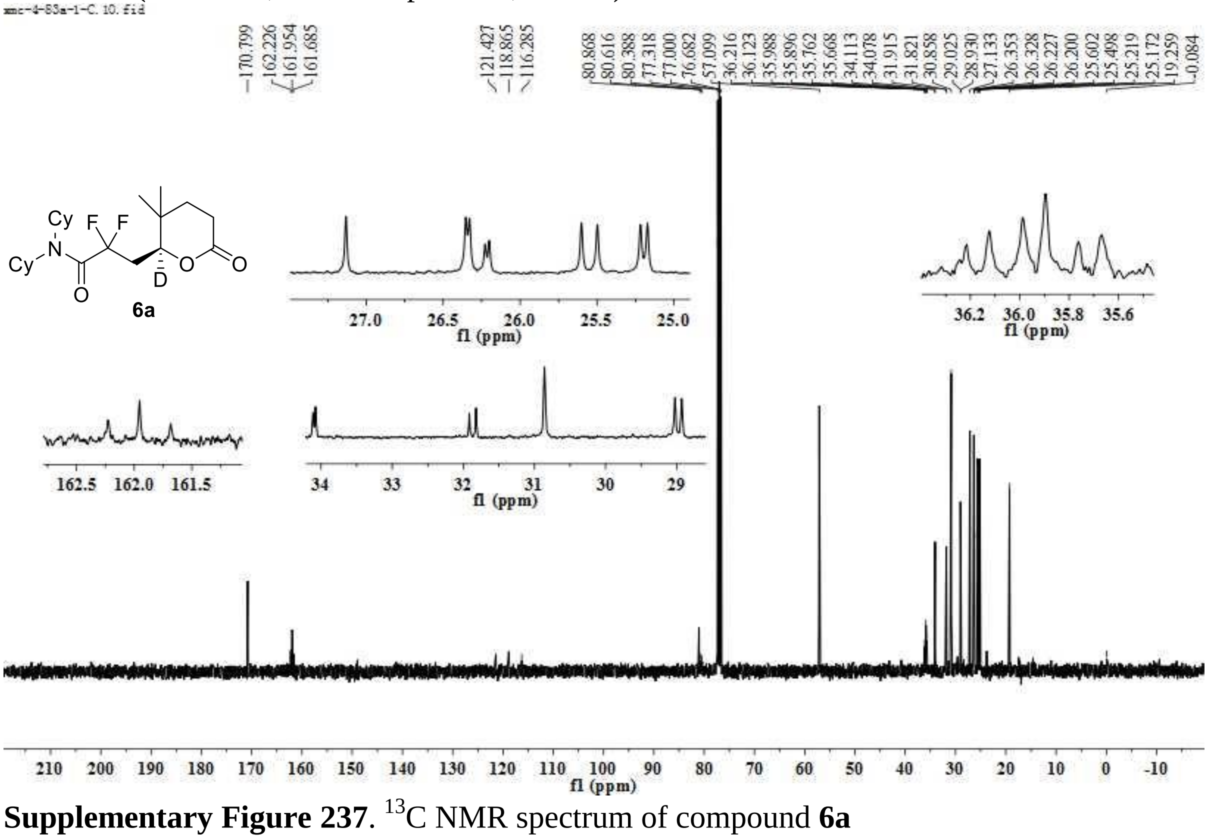

Supplementary Figure 237. ^{13}C NMR spectrum of compound **6a**

^{19}F NMR (376 MHz, room temperature, $CDCl_3$)

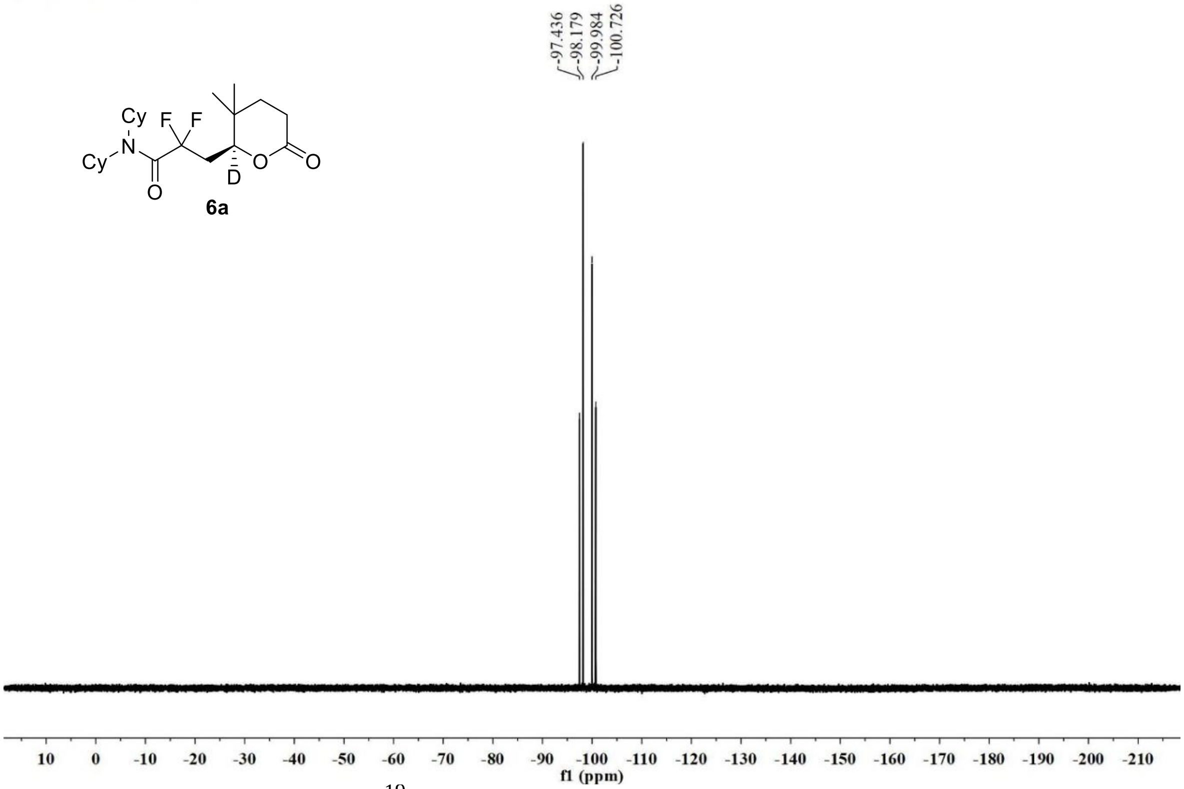

Supplementary Figure 238. ^{19}F NMR spectrum of compound **6a**

HPLC spectrum of racemic **6a**

Data File : XMC-2-85a-210805.lcd
Method File : 3AD-H-90-0.5-214.lcm
Date Processed : 8/5/2021 2:25:58 PM

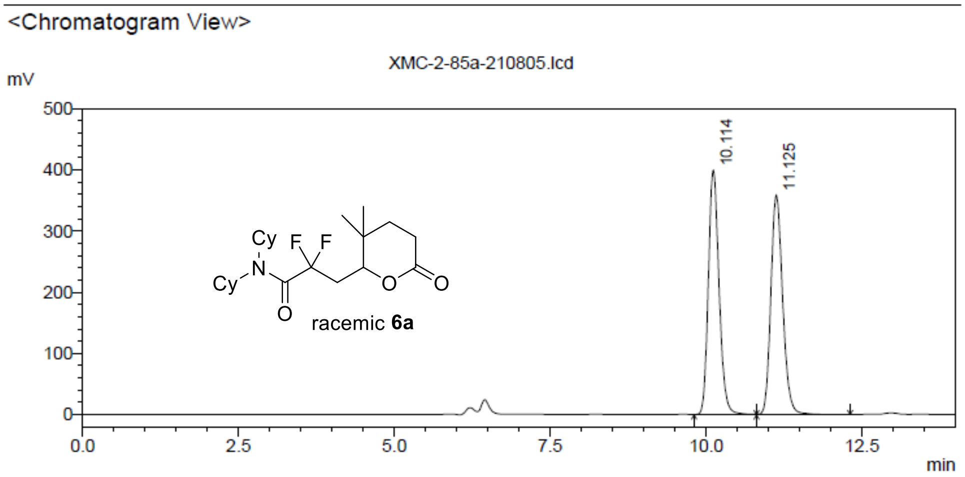

<Data Analysis>

Detector A 214nm

Pesk #	Ret. Time	Height	Area	Area%
1	10.114	399842	4782963	50.216
2	11.125	358998	4741808	49.784
Total		758840	9524771	100.000

Supplementary Figure 239. HPLC spectrum of racemic **6a**

HPLC spectrum of **6a**

Data File	: xmc-4-146-1-1.lcd
Method File	: 3AD-H-90-0.5-214.lcm
Date Processed	: 10/19/2021 2:44:29 PM

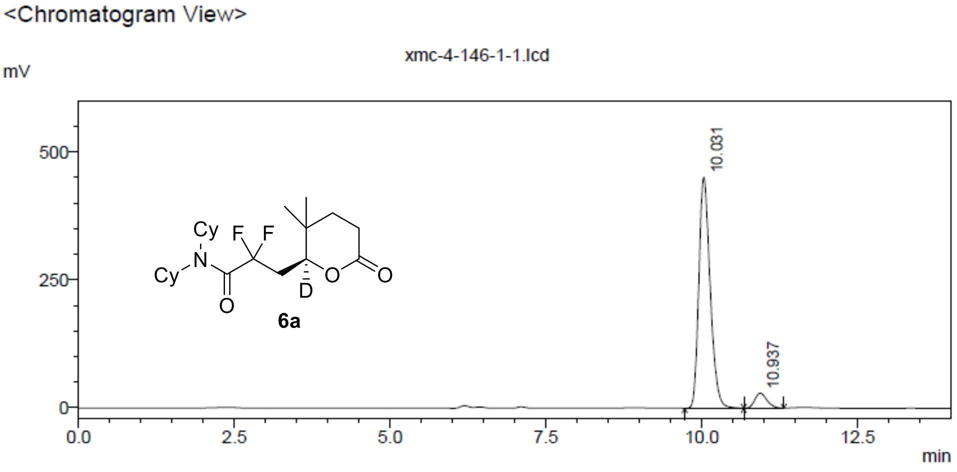

<Data Analysis>

Detector A 214nm

Pesk #	Ret. Time	Height	Area	Area%
1	10.031	451215	5719342	93.182
2	10.937	29929	418501	6.818
Total		481143	6137843	100.000

Supplementary Figure 240. HPLC spectrum of **6a**

^{1}H NMR (400 MHz, room temperature, $CDCl_3$)

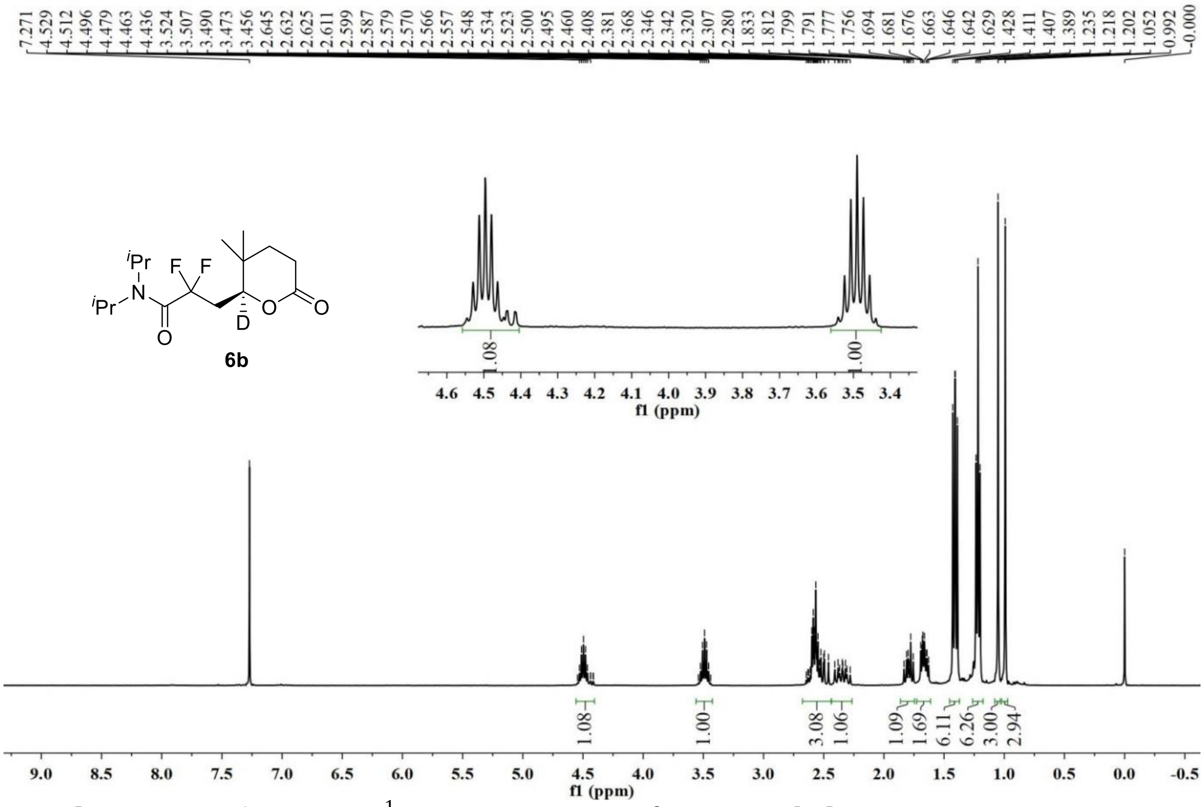

Supplementary Figure 241. ^{1}H NMR spectrum of compound **6b**

^{13}C NMR (100 MHz, room temperature, $CDCl_3$)

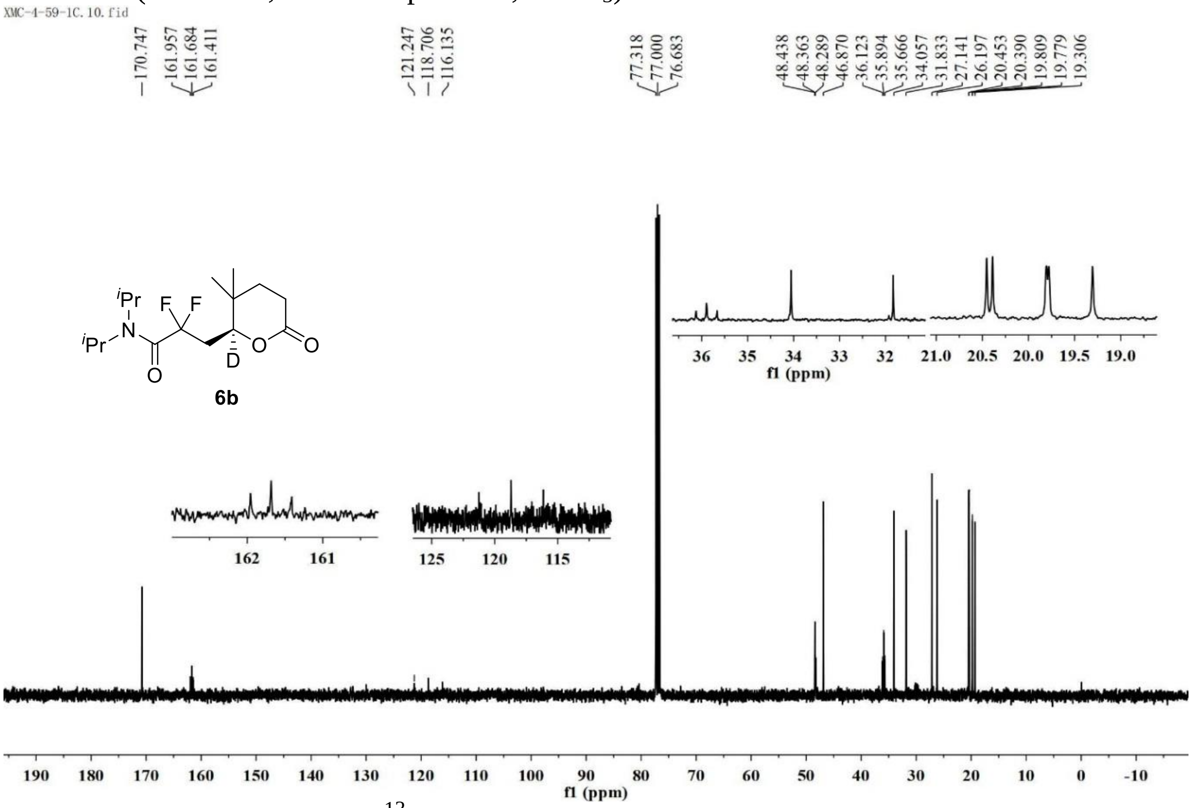

Supplementary Figure 242. ^{13}C NMR spectrum of compound **6b**

^{19}F NMR (376 MHz, room temperature, $CDCl_3$)

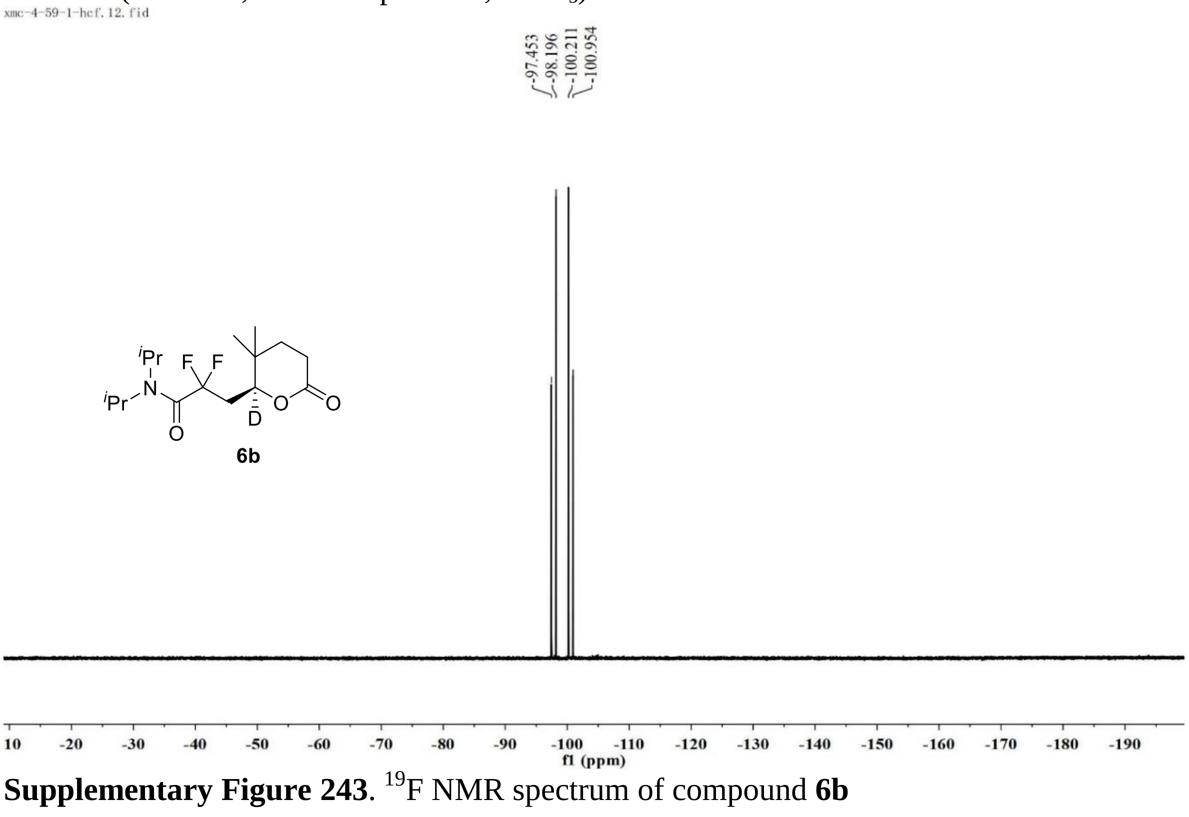

Supplementary Figure 243. ^{19}F NMR spectrum of compound **6b**

HPLC spectrum of racemic **6b**

Data File	: xmc-2-61a-1-100501.lcd
Method File	: 4OD-H-90-0.5-214.lcm
Date Processed	: 10/5/2021 9:19:44 PM

<Chromatogram View>

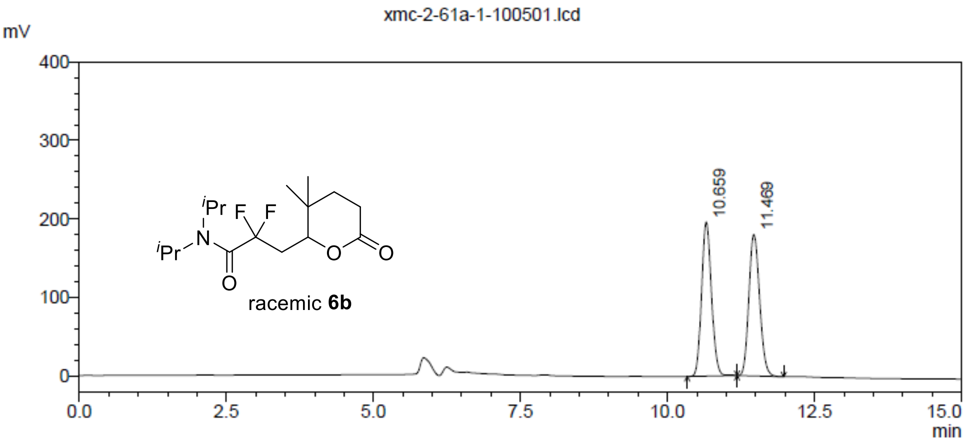

<Data Analysis>

Detector A 214nm

Pesk #	Ret. Time	Height	Area	Area%
1	10.659	196362	2303539	50.103
2	11.469	180255	2294061	49.897
Total		376617	4597600	100.000

Supplementary Figure 244. HPLC spectrum of racemic **6b**

HPLC spectrum of **6b**

Data File	: xmc-4-155-1-1-02.lcd
Method File	: 4OD-H-90-0.5-214.lcm
Date Processed	: 10/19/2021 8:02:22 PM

<Chromatogram View>

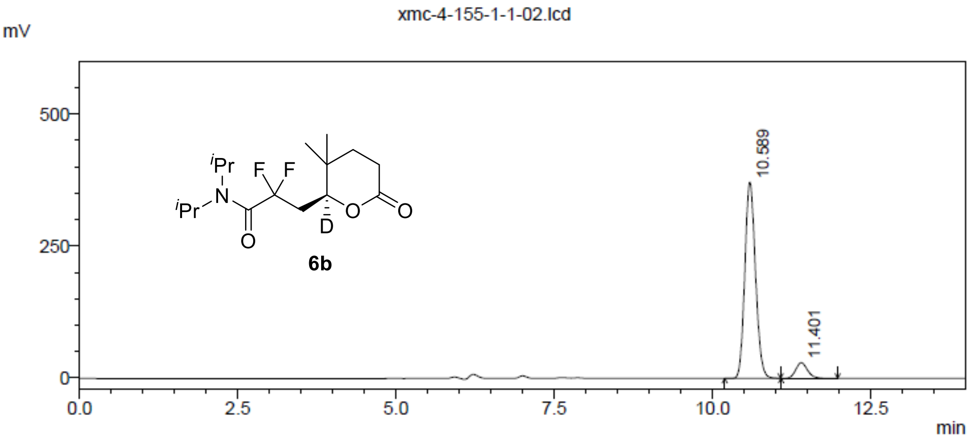

<Data Analysis>

Detector A 214nm

Pesk #	Ret. Time	Height	Area	Area%
1	10.589	371437	4358314	91.957
2	11.401	29545	381184	8.043
Total		400981	4739499	100.000

Supplementary Figure 245. HPLC spectrum of **6b**

^{1}H NMR (400 MHz, room temperature, $CDCl_3$)

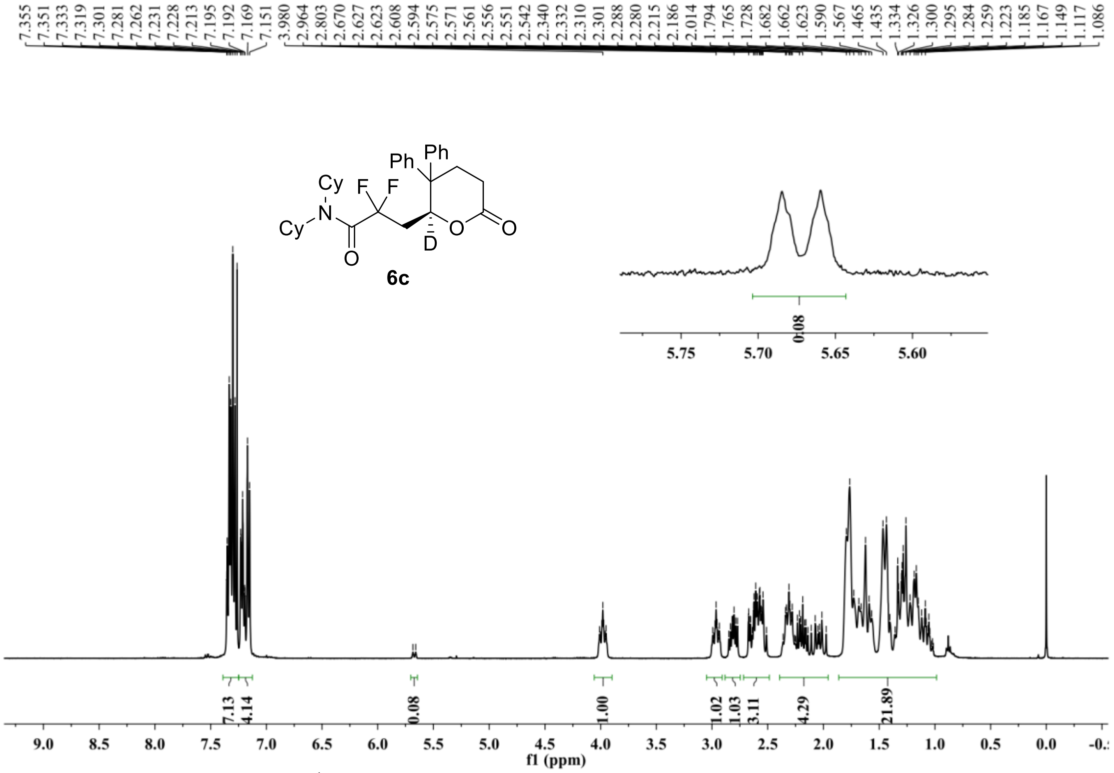

Supplementary Figure 246. ^{1}H NMR spectrum of compound **6c**

^{13}C NMR (100 MHz, room temperature, $CDCl_3$)

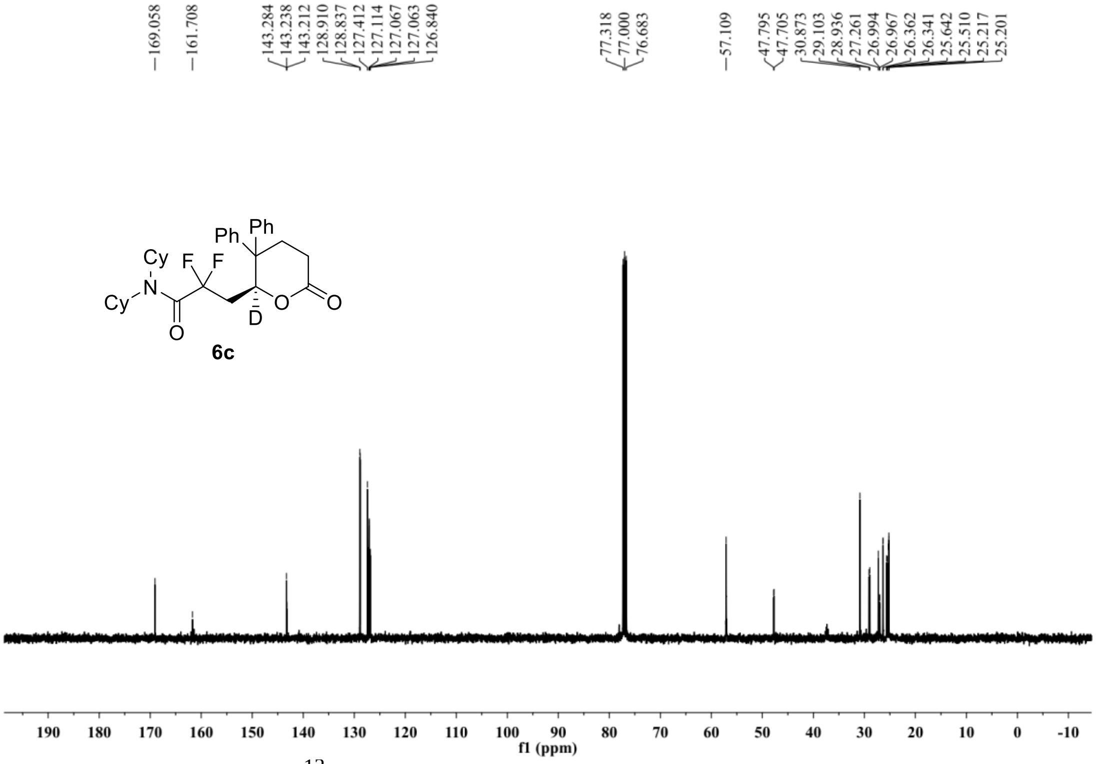

Supplementary Figure 247. ^{13}C NMR spectrum of compound **6c**

^{19}F NMR (376 MHz, room temperature, $CDCl_3$)

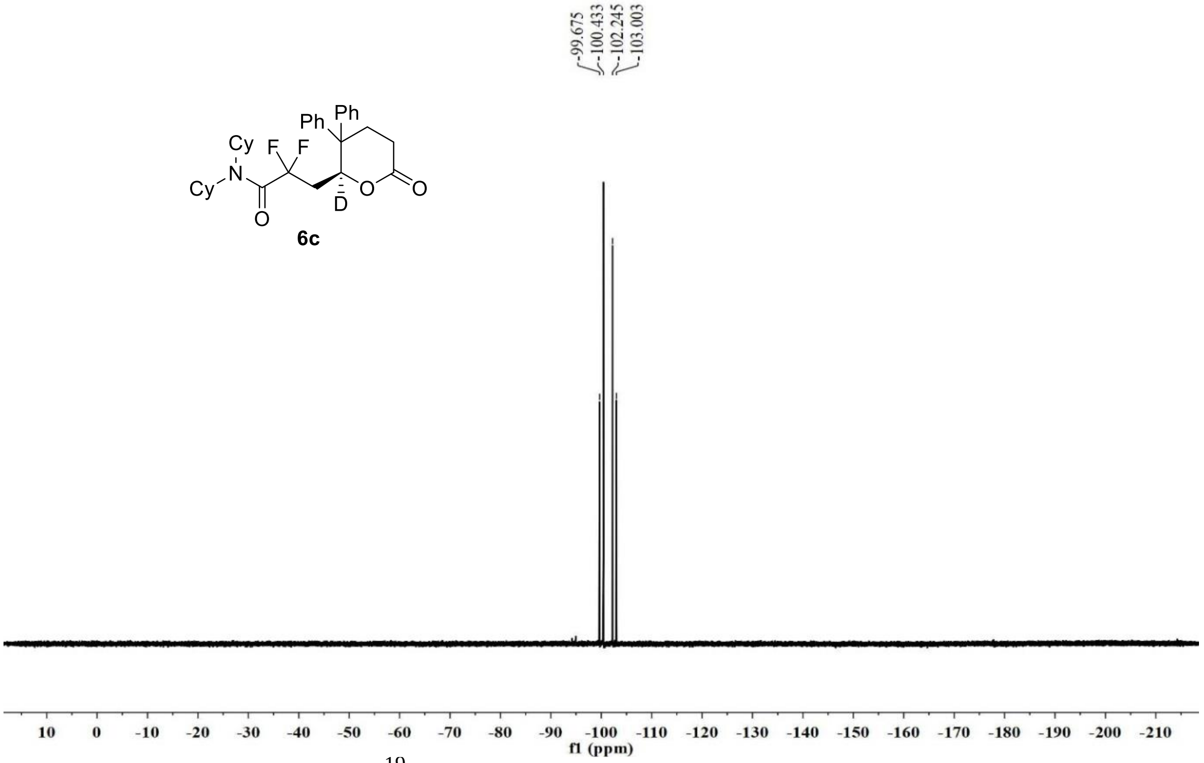

Supplementary Figure 248. ^{19}F NMR spectrum of compound **6c**

HPLC spectrum of racemic **6c**

Data File : xmc-4-22-1-1022.lcd
Method File : 3AD-H-95-1-214.lcm
Date Processed : 10/22/2021 6:10:07 PM

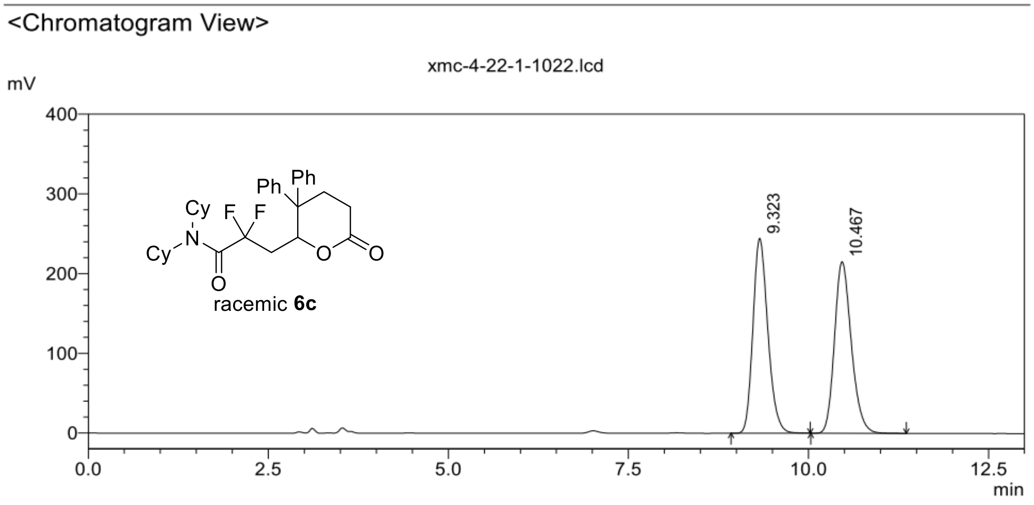

<Data Analysis>

Detector A 214nm

Pesk #	Ret. Time	Height	Area	Area%
1	9.323	244436	3521602	50.115
2	10.467	215063	3505400	49.885
Total		459499	7027002	100.000

Supplementary Figure 249. HPLC spectrum of racemic **6c**

HPLC spectrum of **6c**

Data File	: xmc-4-165-1.lcd
Method File	: 3AD-H-95-1-214.lcm
Date Processed	: 10/28/2021 10:49:37 PM

<Chromatogram View>

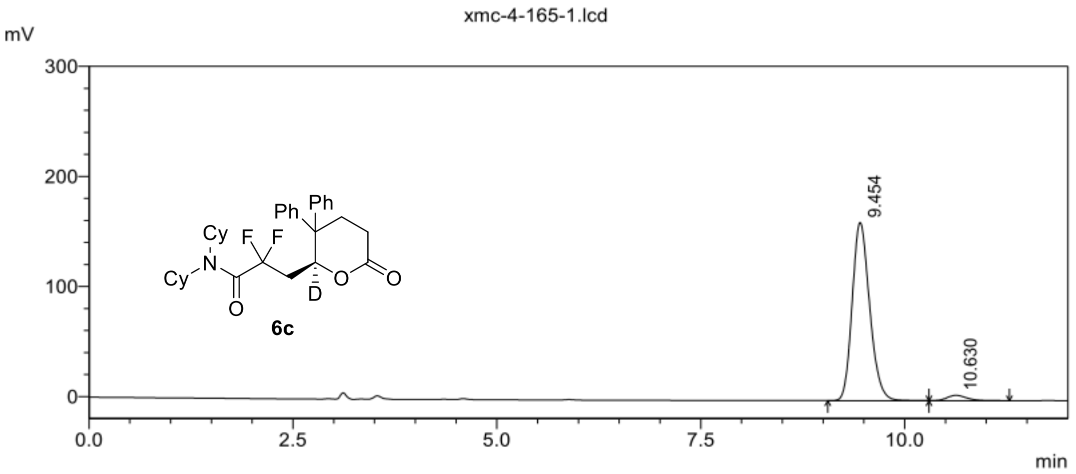

<Data Analysis>

Detector A 214nm

Pesk #	Ret. Time	Height	Area	Area%
1	9.454	161779	2413366	96.706
2	10.630	4813	82193	3.294
Total		166592	2495559	100.000

Supplementary Figure 250. HPLC spectrum of **6c**

^{1}H NMR (400 MHz, room temperature, $CDCl_3$)

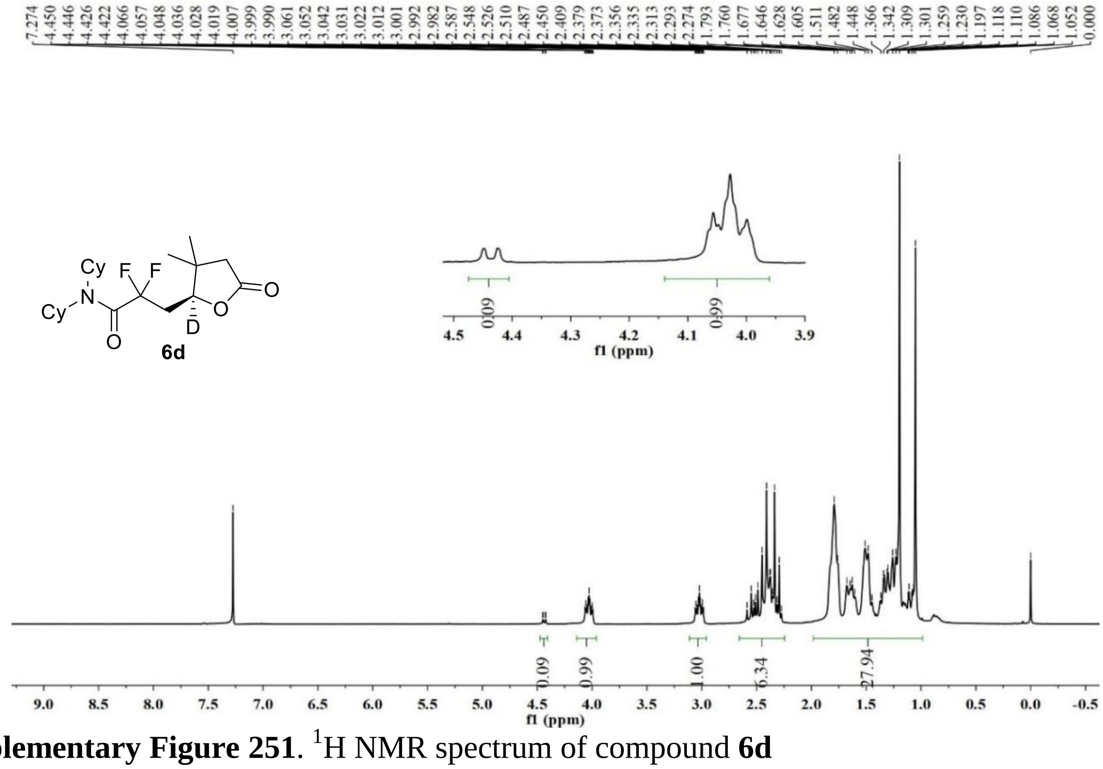

Supplementary Figure 251. ^{1}H NMR spectrum of compound **6d**

^{13}C NMR (100 MHz, room temperature, $CDCl_3$)

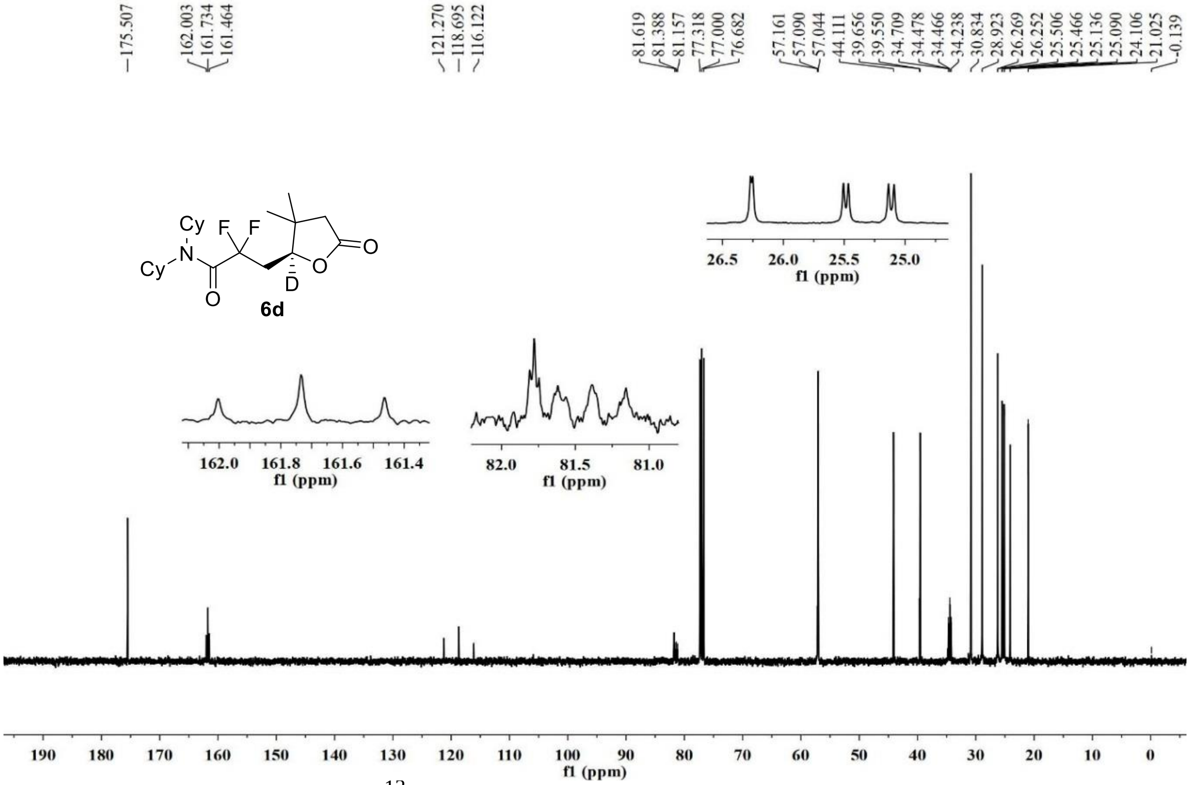

Supplementary Figure 252. ^{13}C NMR spectrum of compound **6d**

^{19}F NMR (376 MHz, room temperature, $CDCl_3$)

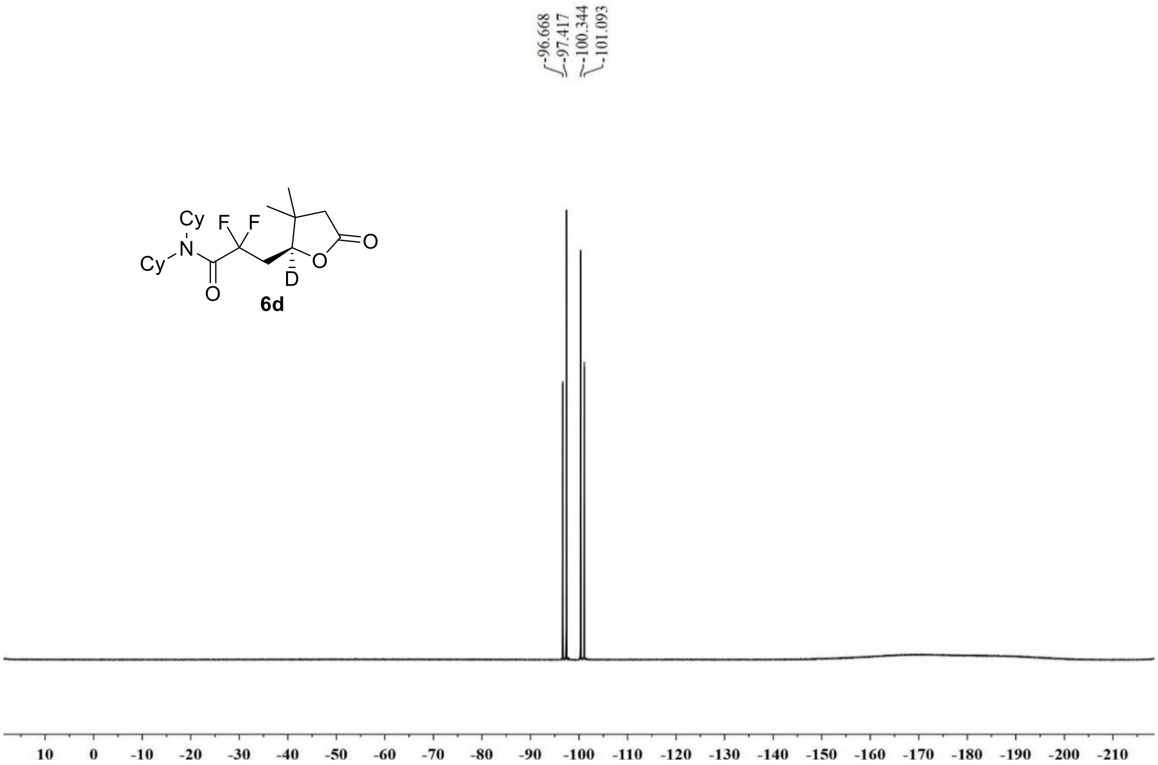

Supplementary Figure 253. ^{19}F NMR spectrum of compound **6d**

HPLC spectrum of racemic **6d**

Data File : yxy-1-63-01.lcd
Method File : 3AD-H-90-0.5-214-30min.lcm
Date Processed : 8/5/2021 2:11:20 PM

<Chromatogram View>

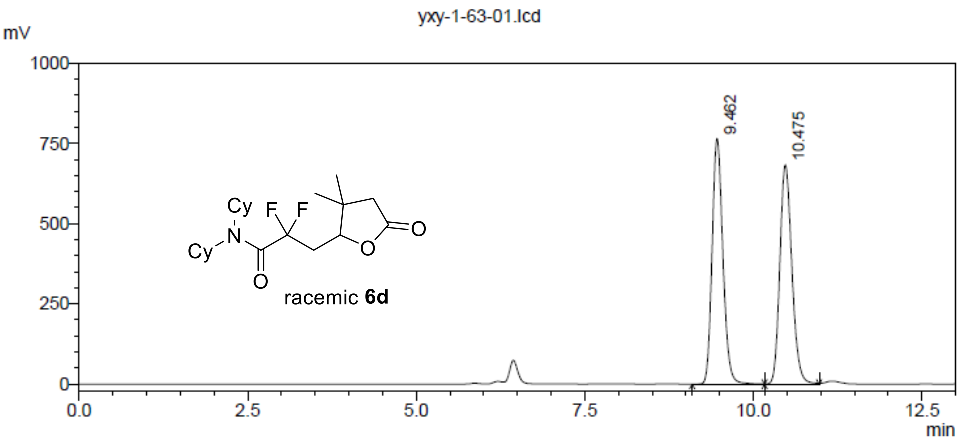

<Data Analysis>

Detector A 214nm

Pesk #	Ret. Time	Height	Area	Area%
1	9.462	765269	8570586	50.105
2	10.475	683338	8534804	49.895
Total		1448607	17105391	100.000

Supplementary Figure 254. HPLC spectrum of racemic **6d**

HPLC spectrum of **6d**

Data File : xmc-2-156-1.lcd
Method File : 3AD-H-90-0.5-214.lcm
Date Processed : 10/19/2021 2:44:16 PM

<Chromatogram View>

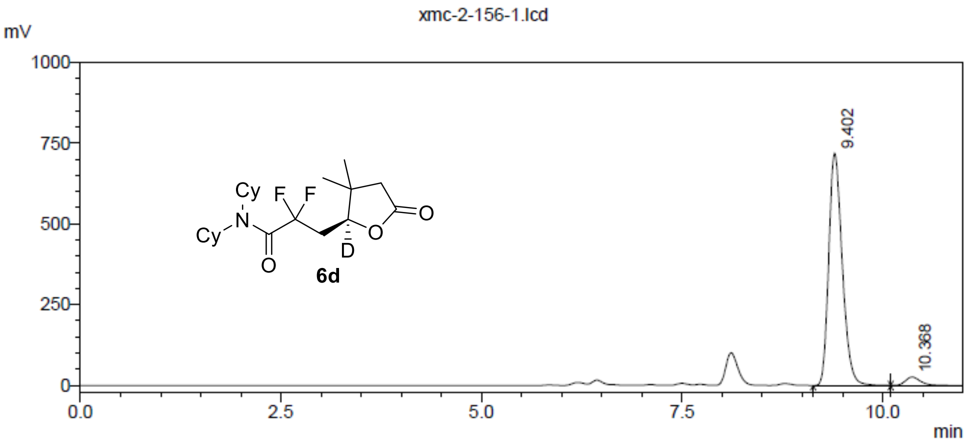

<Data Analysis>

Detector A 214nm

Pesk #	Ret. Time	Height	Area	Area%
1	9.402	718686	8447603	95.849
2	10.368	26516	365843	4.151
Total		745202	8813445	100.000

Supplementary Figure 255. HPLC spectrum of **6d**

^{1}H NMR (400 MHz, room temperature, $CDCl_3$)

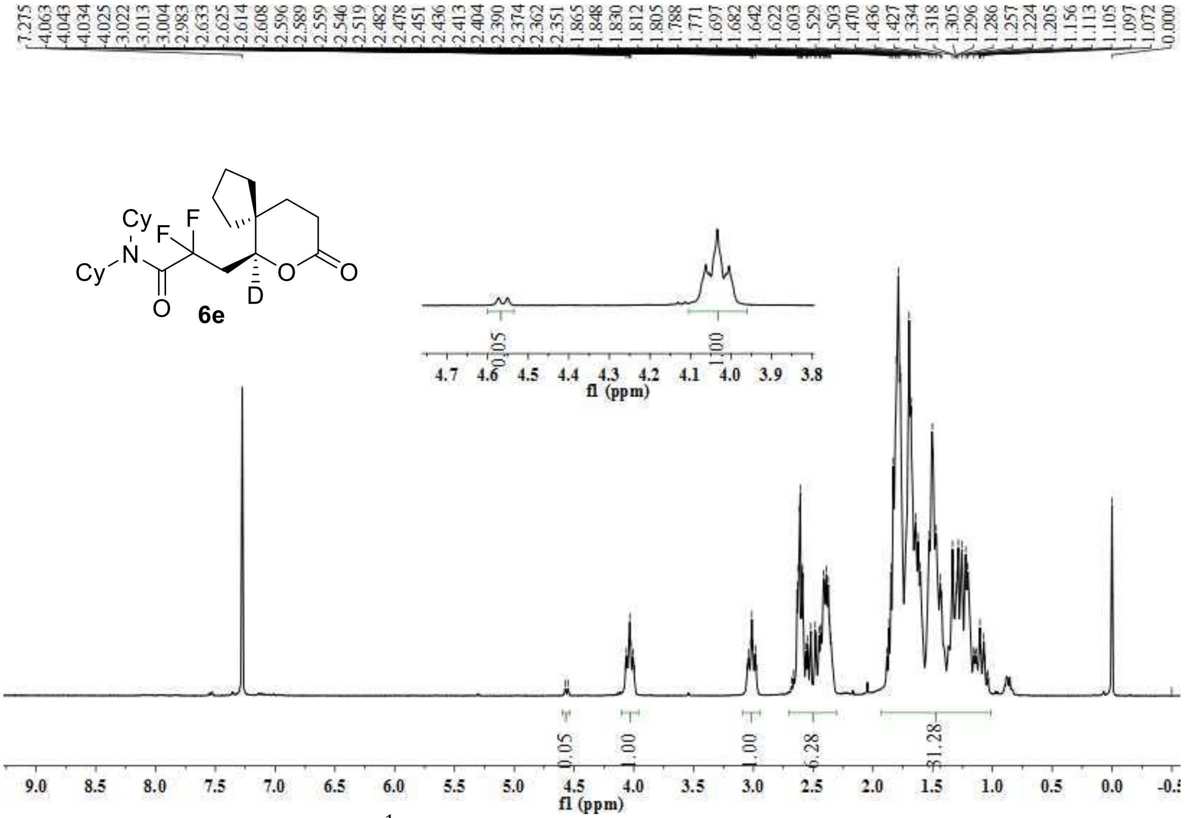

Supplementary Figure 256. ^{1}H NMR spectrum of compound **6e**

^{13}C NMR (100 MHz, room temperature, $CDCl_3$)

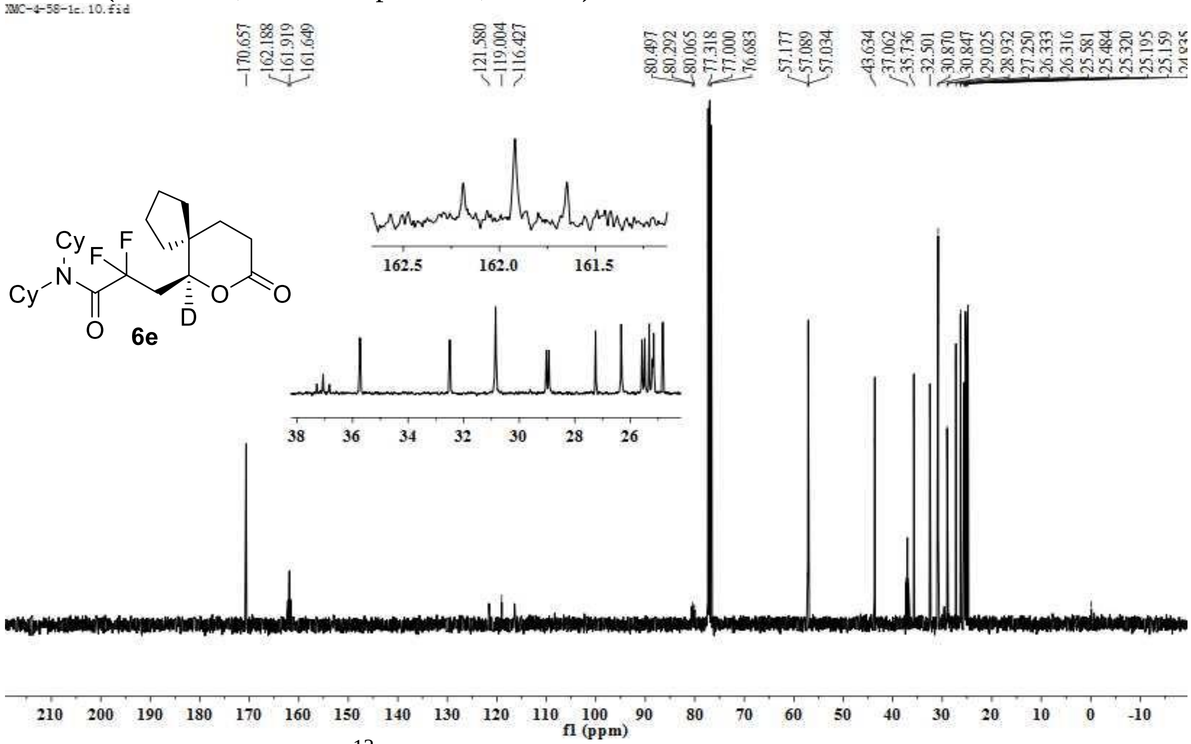

Supplementary Figure 257. ^{13}C NMR spectrum of compound **6e**

^{19}F NMR (376 MHz, room temperature, $CDCl_3$)

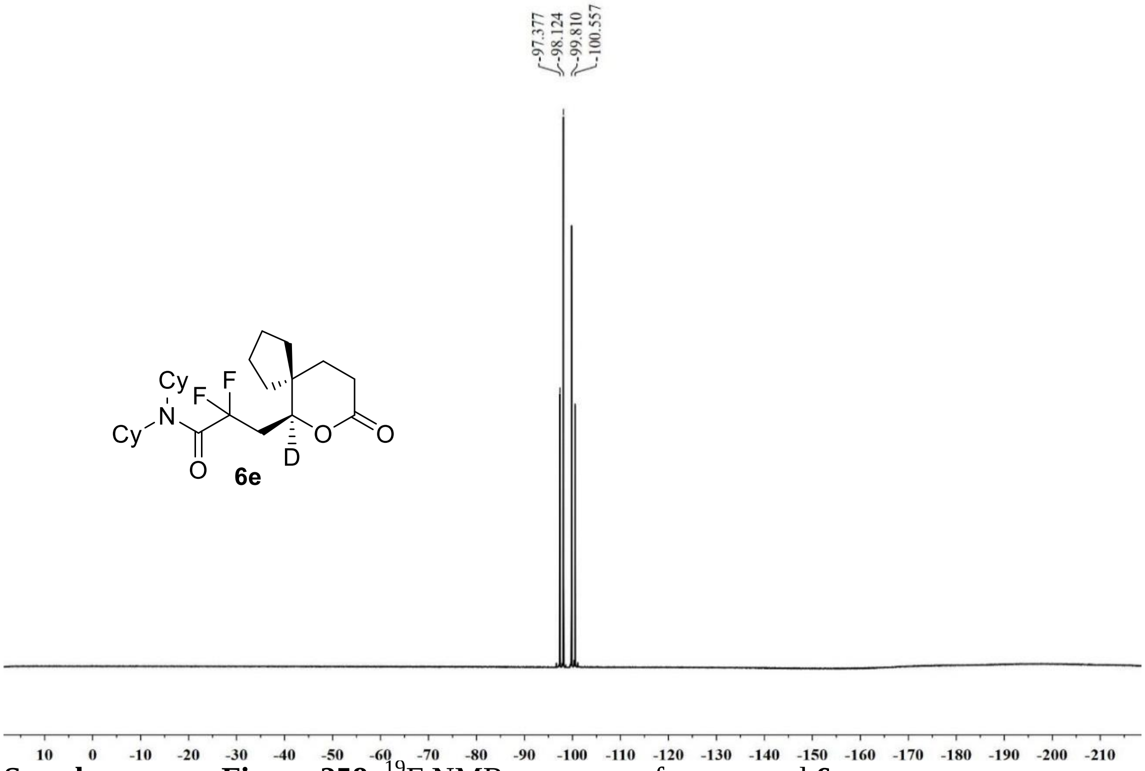

Supplementary Figure 258. ^{19}F NMR spectrum of compound **6e**

HPLC spectrum of racemic **6e**

Data File : xmc-4-21-2-01.lcd
Method File : 4OD-H-98-1-214.lcm
Date Processed : 10/13/2021 6:37:53 PM

<Chromatogram View>

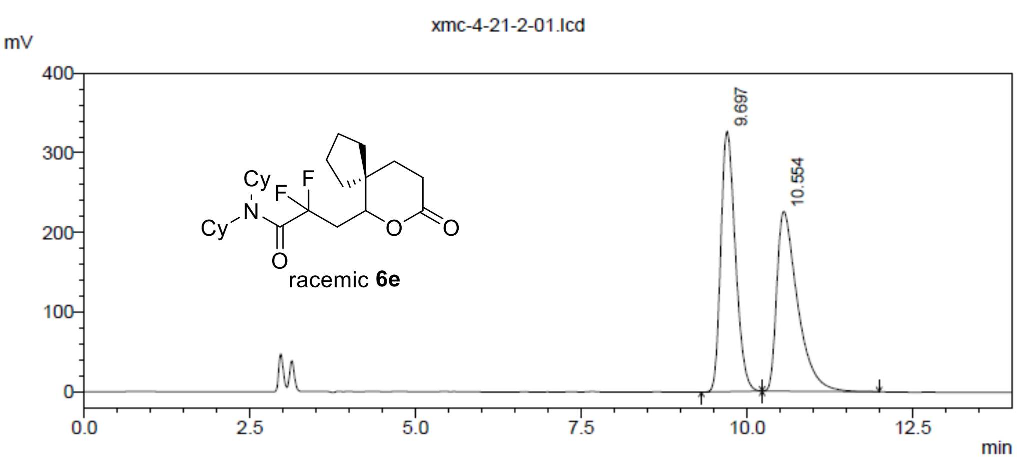

<Data Analysis>

Detector A 214nm

Pesk #	Ret. Time	Height	Area	Area%
1	9.697	327019	5003046	50.296
2	10.554	225829	4944142	49.704
Total		552848	9947188	100.000

Supplementary Figure 259. HPLC spectrum of racemic **6e**

HPLC spectrum of **6e**

Data File	: xmc-2-157-1-1.lcd
Method File	: 4OD-H-98-1-214.lcm
Date Processed	: 10/18/2021 10:33:06 PM

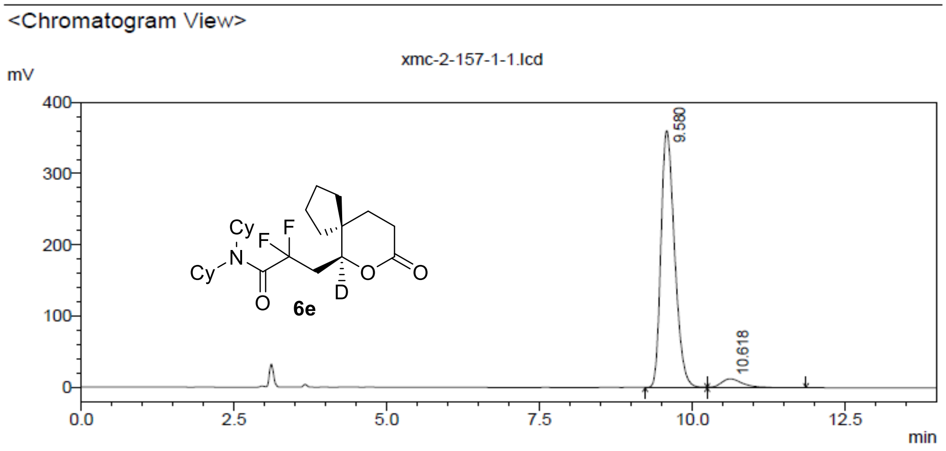

<Data Analysis>

Detector A 214nm

Pesk #	Ret. Time	Height	Area	Area%
1	9.580	360802	5613616	94.834
2	10.618	12443	305780	5.166
Total		373245	5919395	100.000

Supplementary Figure 260. HPLC spectrum of **6e**

^{1}H NMR (400 MHz, room temperature, $CDCl_3$)

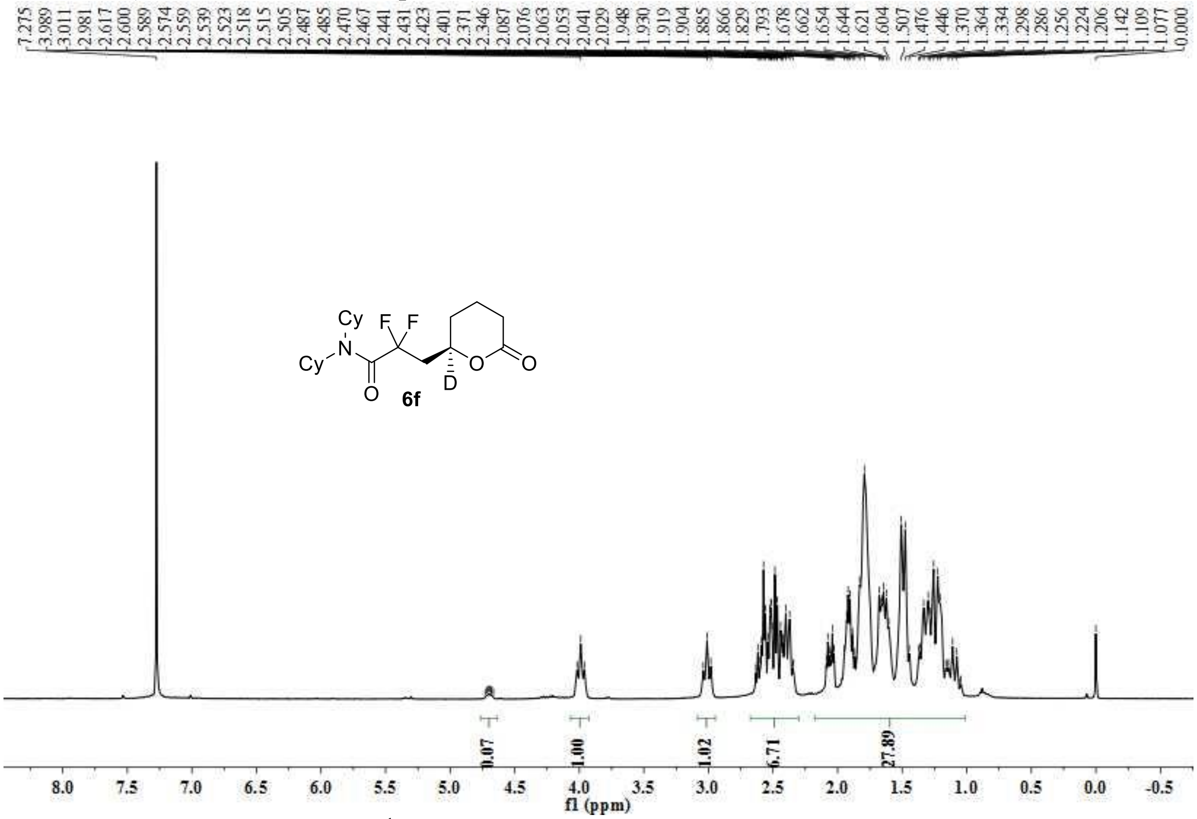

Supplementary Figure 261. ^{1}H NMR spectrum of compound **6f**

^{13}C NMR (100 MHz, room temperature, $CDCl_3$)

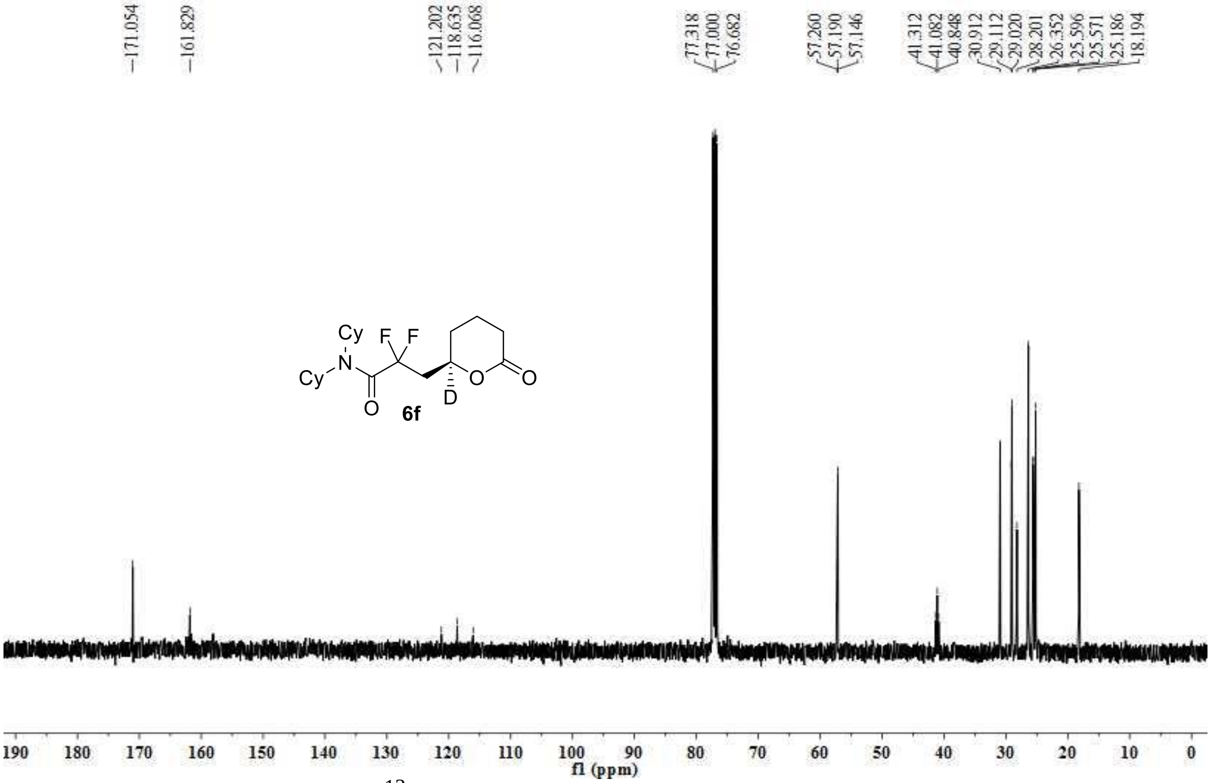

Supplementary Figure 262. ^{13}C NMR spectrum of compound **6f**

^{19}F NMR (376 MHz, room temperature, $CDCl_3$)

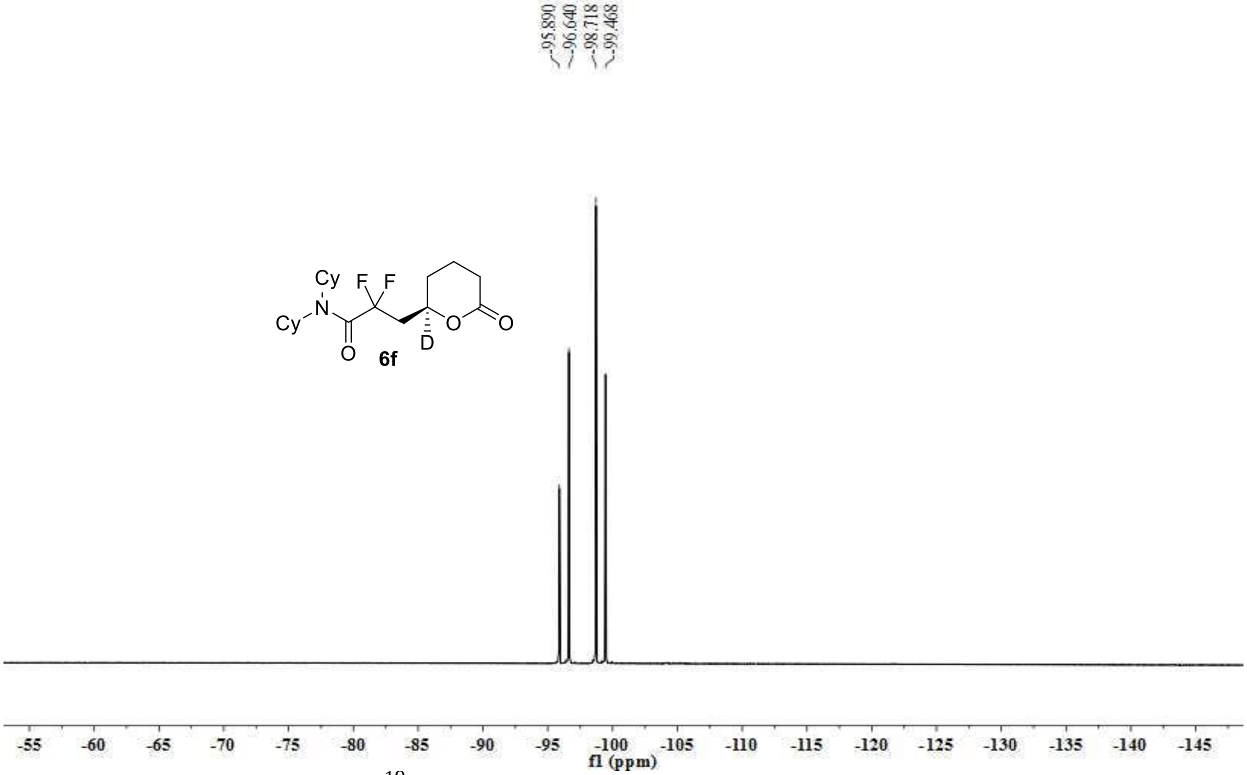

Supplementary Figure 263. ^{19}F NMR spectrum of compound **6f**

HPLC spectrum of racemic **6f**

Method File : 1OJ-H-99.7-0.5-214.lcm
Date Acquired : 6/14/2022 10:52:02 PM
Date Processed : 6/15/2022 9:23:53 AM

<Chromatogram View>

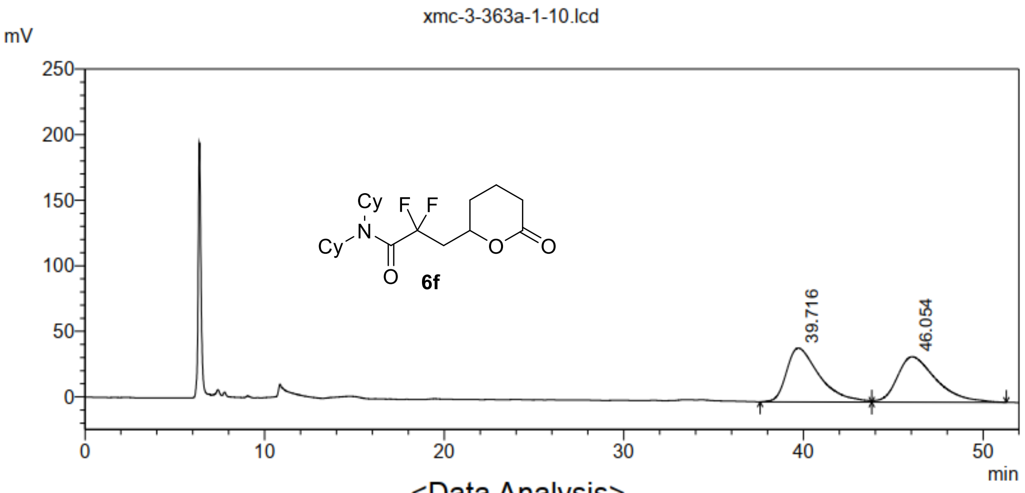

<Data Analysis>

Detector A 214nm

Pesk #	Ret. Time	Height	Area	Area%
1	39.716	41022	5214671	50.094
2	46.054	34604	5195173	49.906
Total		75626	10409845	100.000

Supplementary Figure 264. HPLC spectrum of racemic **6f**

HPLC spectrum of **6f**

Method File : 1OJ-H-99.7-0.5-214.lcm
Date Acquired : 6/14/2022 11:53:08 PM
Date Processed : 6/15/2022 9:26:27 AM

<Chromatogram View>

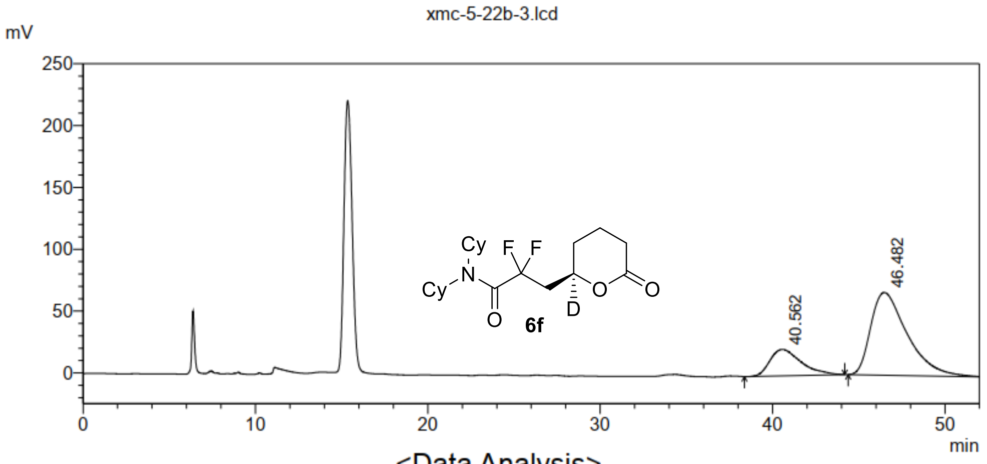

<Data Analysis>

Detector A 214nm

Pesk #	Ret. Time	Height	Area	Area%
1	40.562	21286	2689205	21.397
2	46.482	66929	9878909	78.603
Total		88214	12568114	100.000

Supplementary Figure 265. HPLC spectrum of **6f**

^{1}H NMR (400 MHz, room temperature, $CDCl_3$)

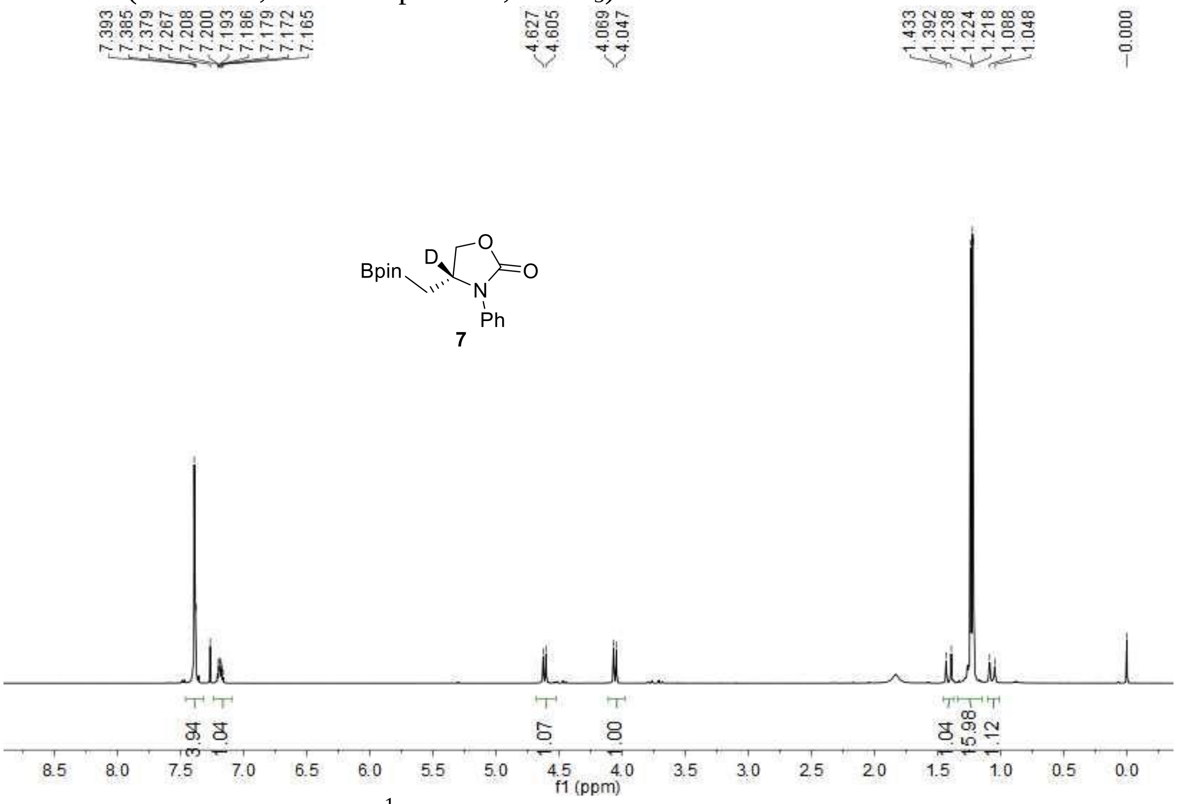

Supplementary Figure 266. ^{1}H NMR spectrum of compound **7**

^{13}C NMR (100 MHz, room temperature, $CDCl_3$)

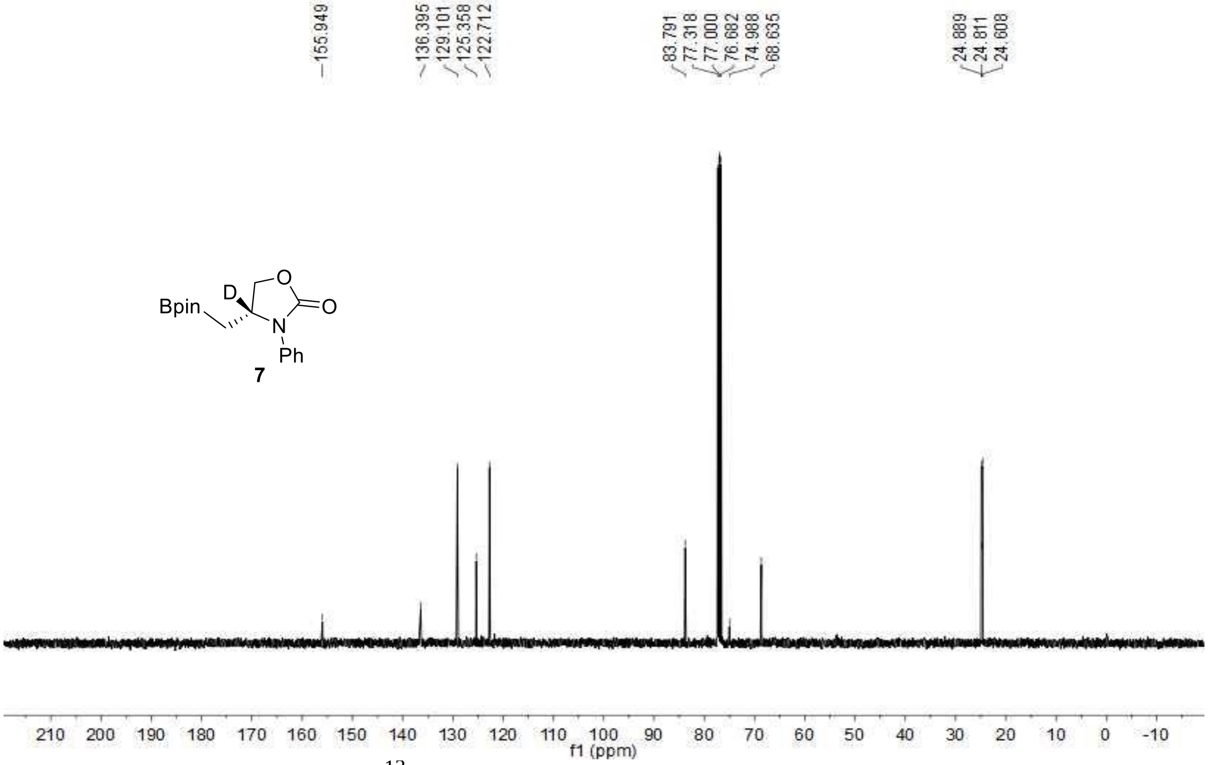

Supplementary Figure 267. ^{13}C NMR spectrum of compound **7**

^{11}B NMR (128 MHz, room temperature, $CDCl_3$)

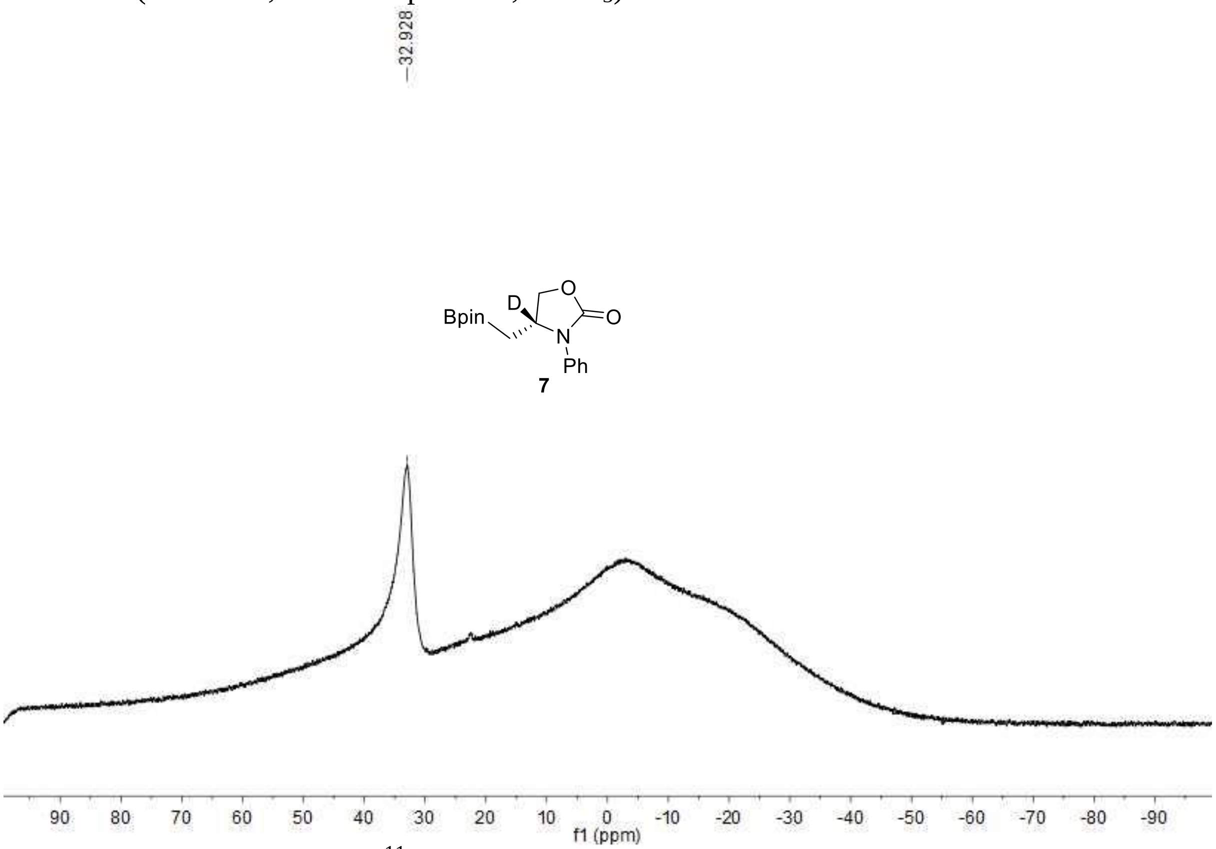

Supplementary Figure 268. ^{11}B NMR spectrum of compound **7**

HPLC spectrum of racemic **7**

Vial#	: 1
Data File	: sql-7-31-1-(Y3).lcd
Method File	: 4OD-H-80-0.5-214.lcm
Date Acquired	: 1/22/2022 7:06:47 PM
Date Processed	: 1/25/2022 1:39:01 AM

<Chromatogram View>

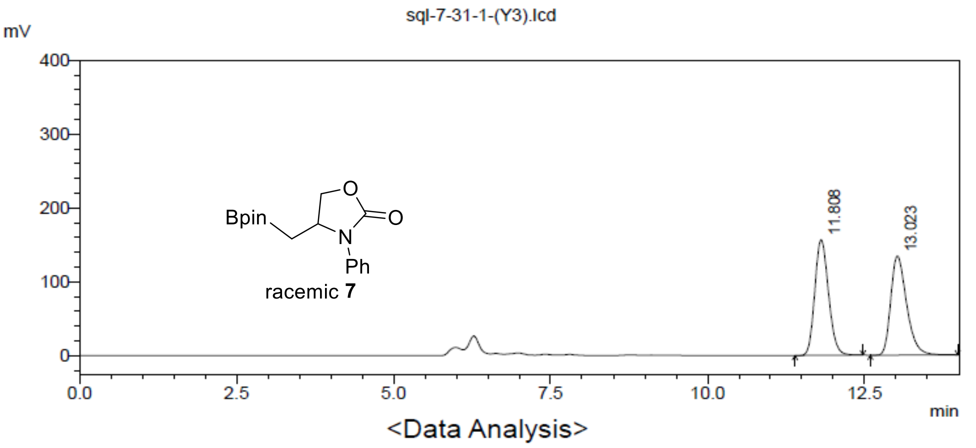

<Data Analysis>

Detector A 214nm

Pesk #	Ret. Time	Height	Area	Area%
1	11.808	156678	2386602	50.272
2	13.023	134074	2360734	49.728
Total		290752	4747336	100.000

Supplementary Figure 269. HPLC spectrum of racemic **7**

HPLC spectrum of **7**

Tray#	: 1
Vial#	: 2
Data File	: yxy-2-231-1-(Y3).lcd
Method File	: 4OD-H-80-0.5-214.lcm
Date Acquired	: 1/22/2022 10:24:22 PM
Date Processed	: 1/25/2022 1:38:21 AM

<Chromatogram View>

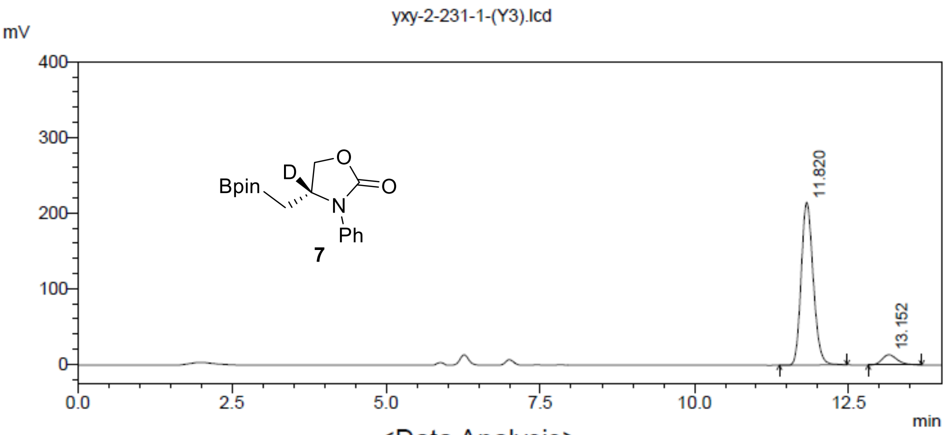

<Data Analysis>

Detector A 214nm

Pesk #	Ret. Time	Height	Area	Area%
1	11.820	215099	2925709	92.531
2	13.152	13567	236153	7.469
Total		228666	3161863	100.000

Supplementary Figure 270. HPLC spectrum of **7**

^{1}H NMR (400 MHz, room temperature, $CDCl_3$)

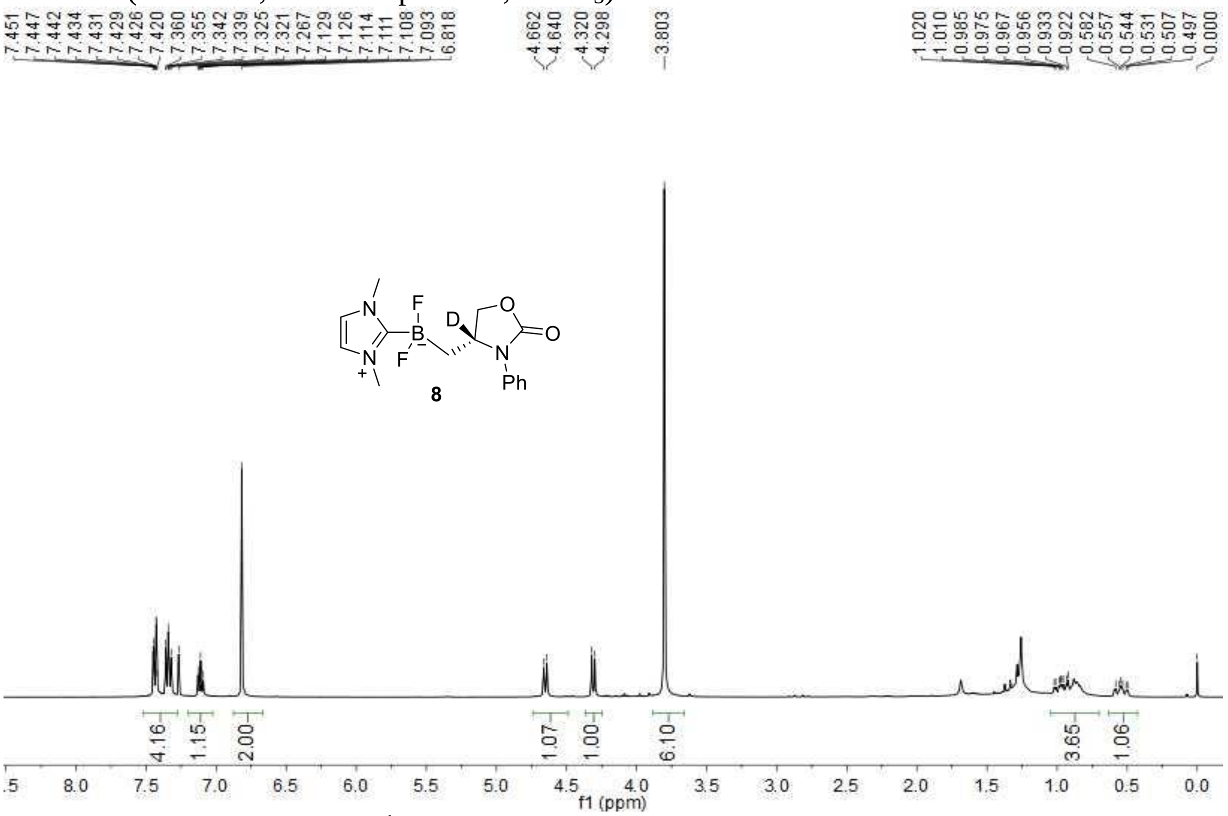

Supplementary Figure 271. ^{1}H NMR spectrum of compound **8**

^{13}C NMR (100 MHz, room temperature, $CDCl_3$)

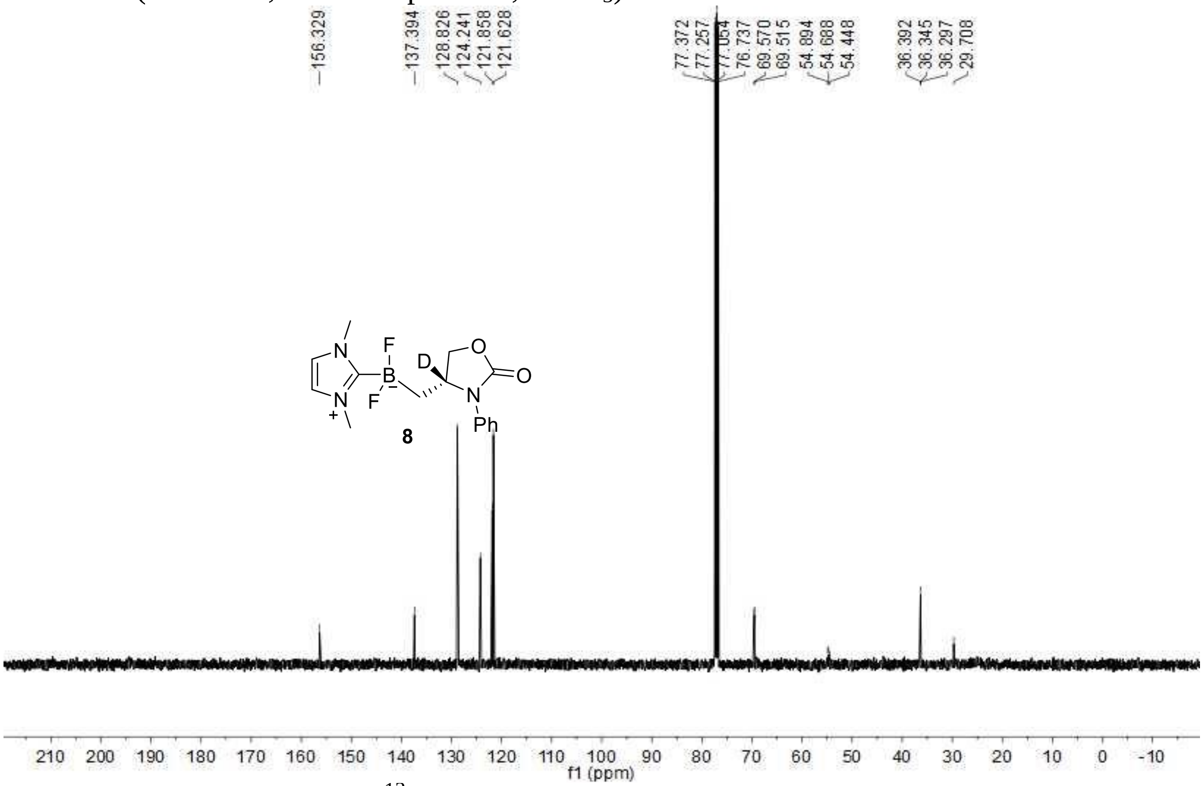

Supplementary Figure 272. ^{13}C NMR spectrum of compound **8**

^{11}B NMR (128 MHz, room temperature, $CDCl_3$)

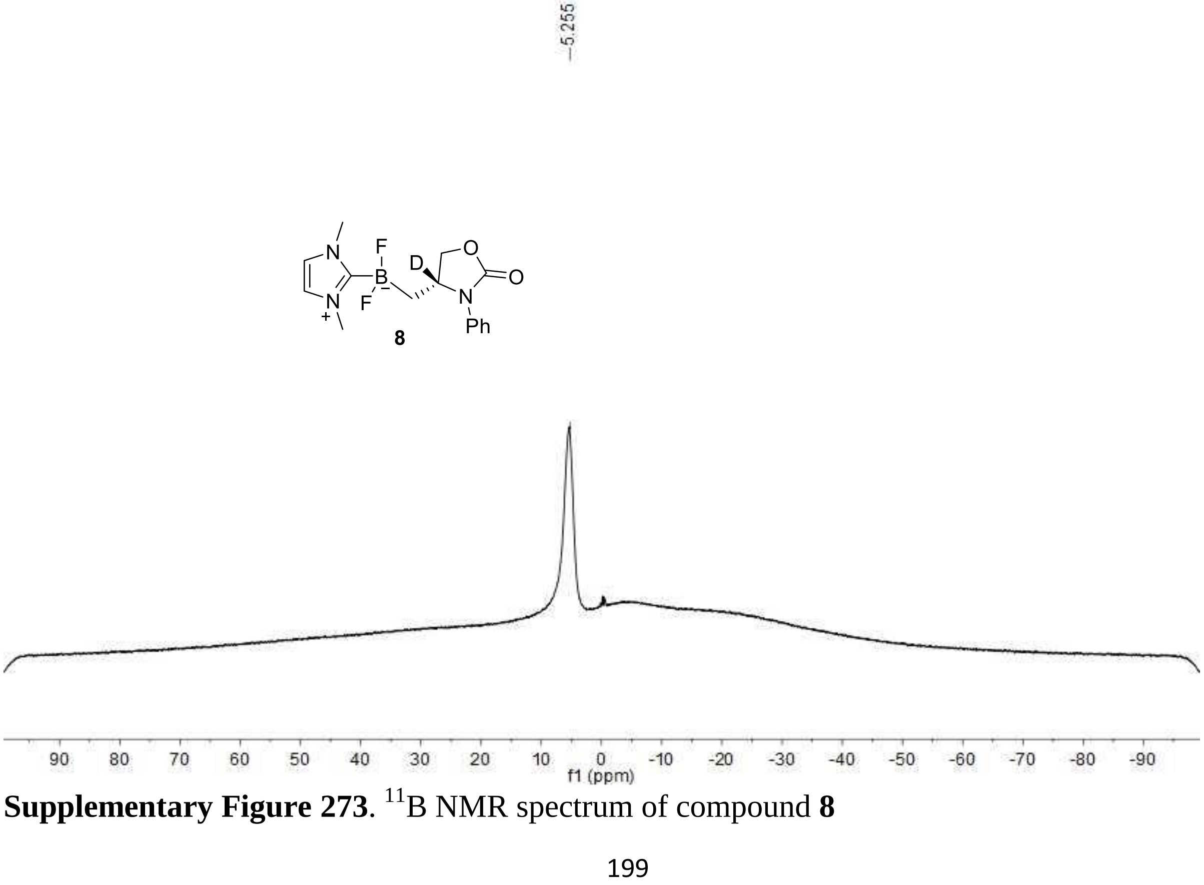

Supplementary Figure 273. ^{11}B NMR spectrum of compound **8**

^{19}F NMR (376 MHz, room temperature, $CDCl_3$)

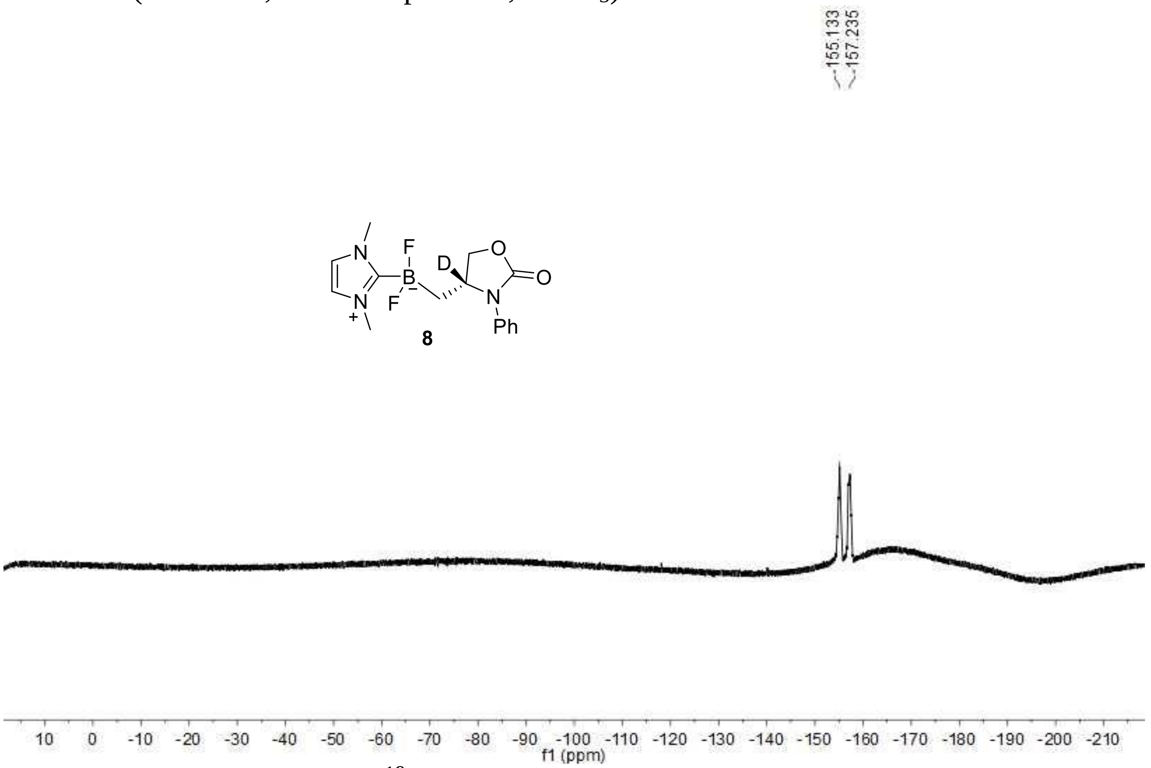

Supplementary Figure 274. ^{19}F NMR spectrum of compound **8**

HPLC spectrum of racemic **8**

Tray#	: 1
Vial#	: 2
Data File	: sql-7-25-2.lcd
Method File	: 4OD-H-65-0.5-214.lcm
Date Acquired	: 2/1/2022 12:33:38 PM
Date Processed	: 2/1/2022 1:23:11 PM

<Chromatogram View>

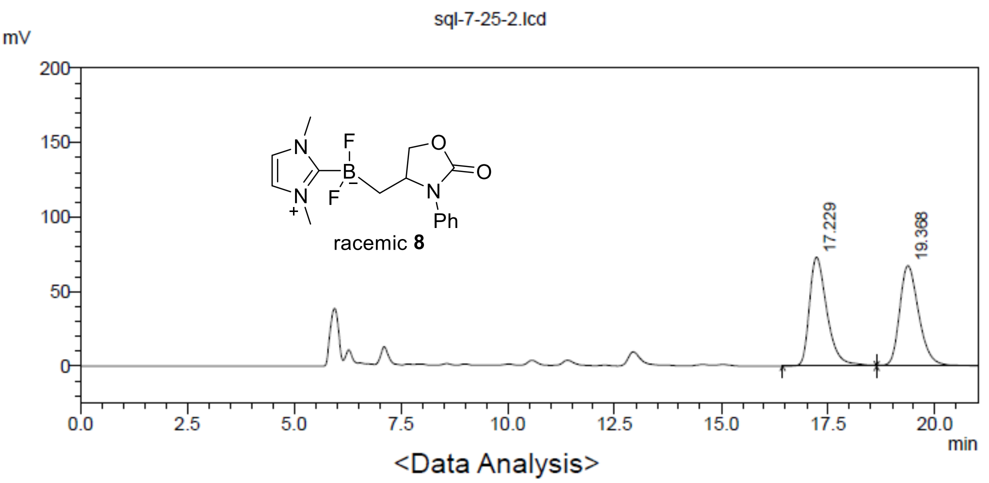

<Data Analysis>

Detector A 214nm

Pesk #	Ret. Time	Height	Area	Area%
1	17.229	73056	2103698	49.721
2	19.368	67394	2127346	50.279
Total		140450	4231044	100.000

Supplementary Figure 275. HPLC spectrum of racemic **8**

HPLC spectrum of **8**

Data File	: sql-7-26-5.lcd
Method File	: 4OD-H-65-0.5-214.lcm
Date Acquired	: 2/1/2022 12:07:32 PM
Date Processed	: 2/1/2022 1:23:19 PM

<Chromatogram View>

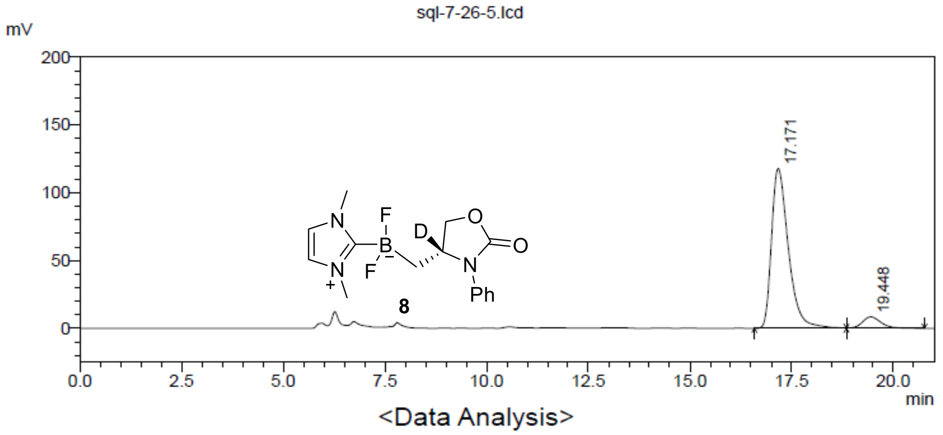

<Data Analysis>

Detector A 214nm

Pesk #	Ret. Time	Height	Area	Area%
1	17.171	117946	3407301	92.781
2	19.448	8375	265130	7.219
Total		126321	3672431	100.000

Supplementary Figure 276. HPLC spectrum of **8**

^{1}H NMR (400 MHz, room temperature, $CDCl_3$)

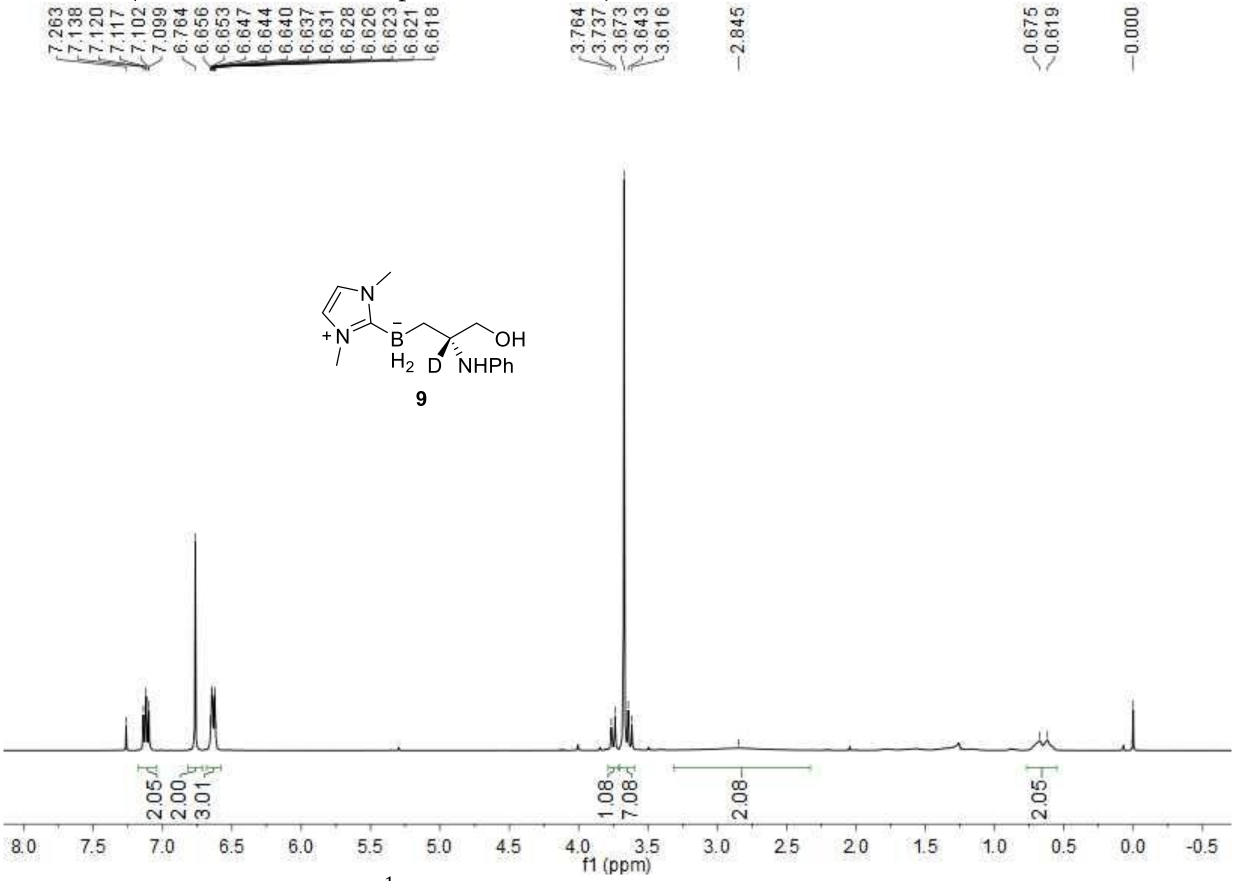

Supplementary Figure 277. ^{1}H NMR spectrum of compound **9**

^{13}C NMR (100 MHz, room temperature, $CDCl_3$)

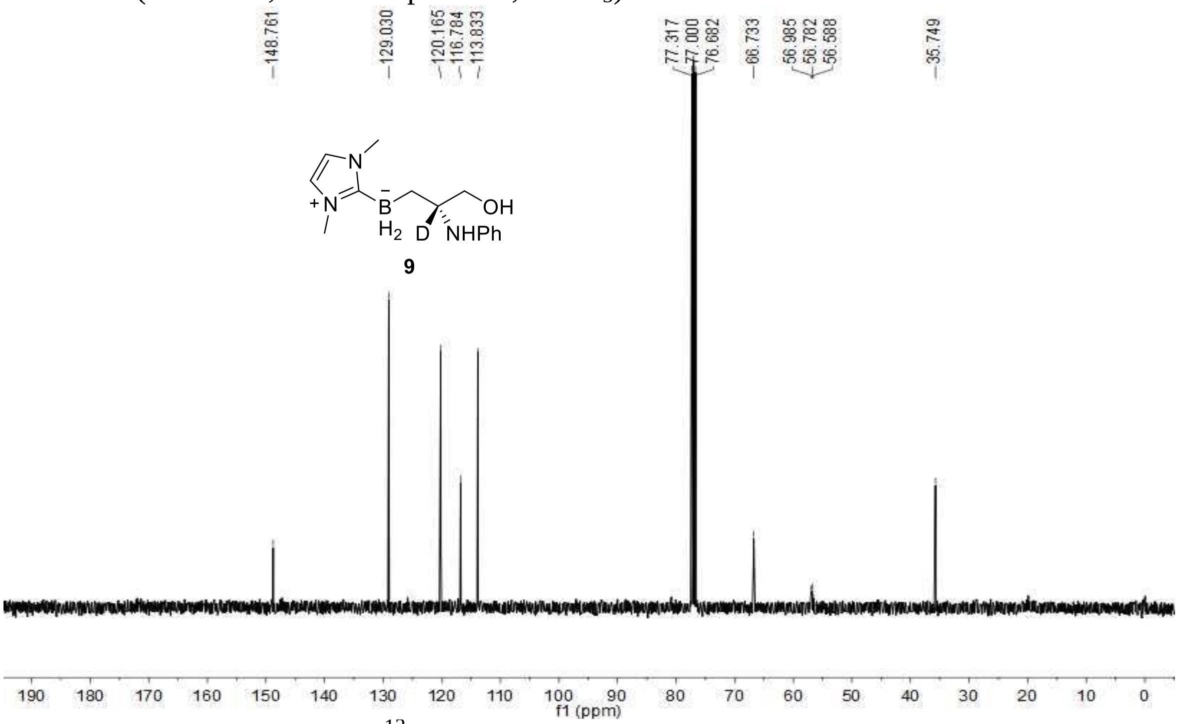

Supplementary Figure 278. ^{13}C NMR spectrum of compound **9**

^{11}B NMR (128 MHz, room temperature, $CDCl_3$)

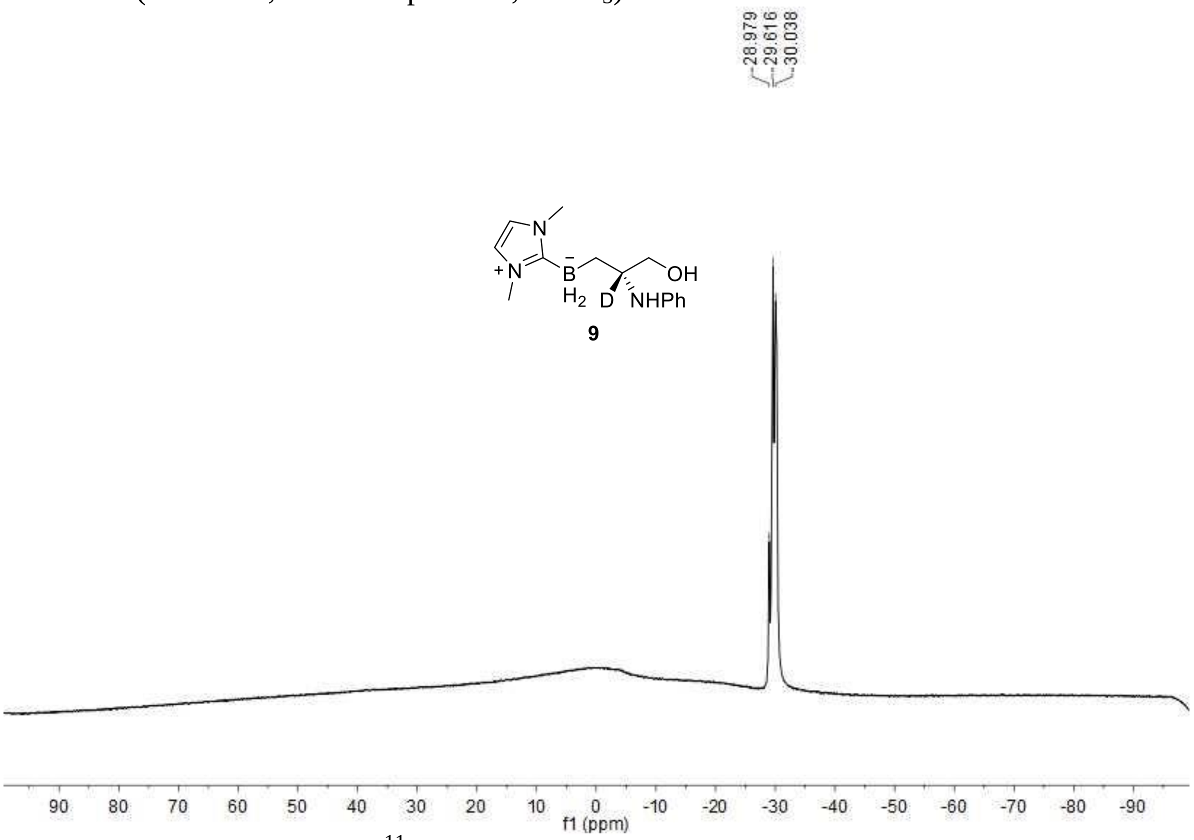

Supplementary Figure 279. ^{11}B NMR spectrum of compound **9**

HPLC spectrum of racemic **9**

Sample Name :
Tray# : 1
Vial# : 32
Data File : SQL-7-27-8-(Y2).lcd
Method File : 4OD-H-90-0.5-214-40min.lcm
Date Acquired : 1/22/2022 12:23:53 AM
Date Processed : 1/25/2022 1:36:17 AM

<Chromatogram View>

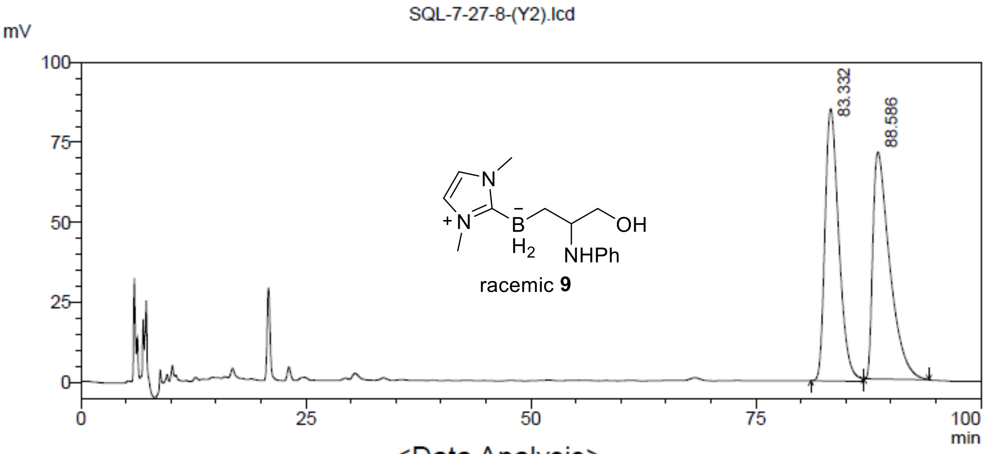

<Data Analysis>

Detector A 214nm

Pesk #	Ret. Time	Height	Area	Area%
1	83.332	85130	9105288	49.534
2	88.586	70990	9276560	50.466
Total		156120	18381848	100.000

Supplementary Figure 280. HPLC spectrum of racemic **9**

HPLC spectrum of **9**

Tray# : 1
Vial# : 33
Data File : SQL-7-36-(Y3).lcd
Method File : 4OD-H-90-0.5-214-40min.lcm
Date Acquired : 1/22/2022 2:04:59 AM
Date Processed : 1/25/2022 1:37:25 AM

<Chromatogram View>

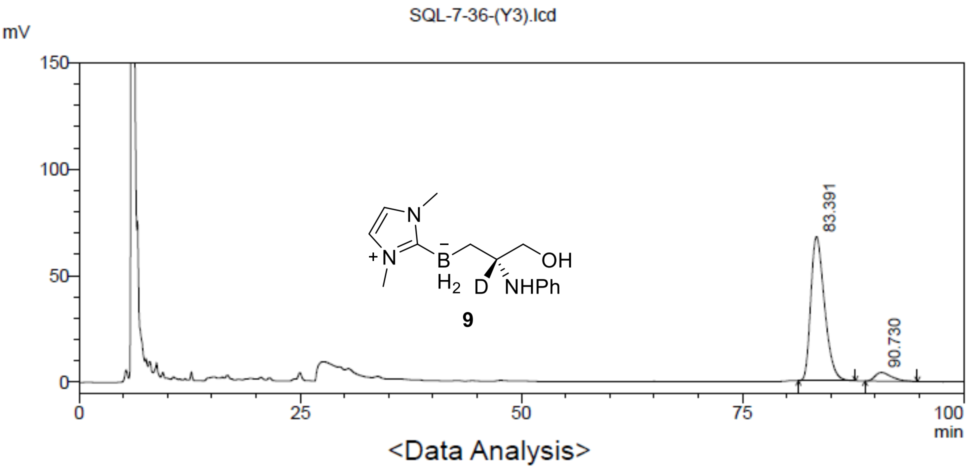

<Data Analysis>

Detector A 214nm

Pesk #	Ret. Time	Height	Area	Area%
1	83.391	67682	7328586	93.248
2	90.730	4130	530641	6.752
Total		71812	7859227	100.000

Supplementary Figure 281. HPLC spectrum of **9**

^{1}H NMR (400 MHz, room temperature, CD_3OD)

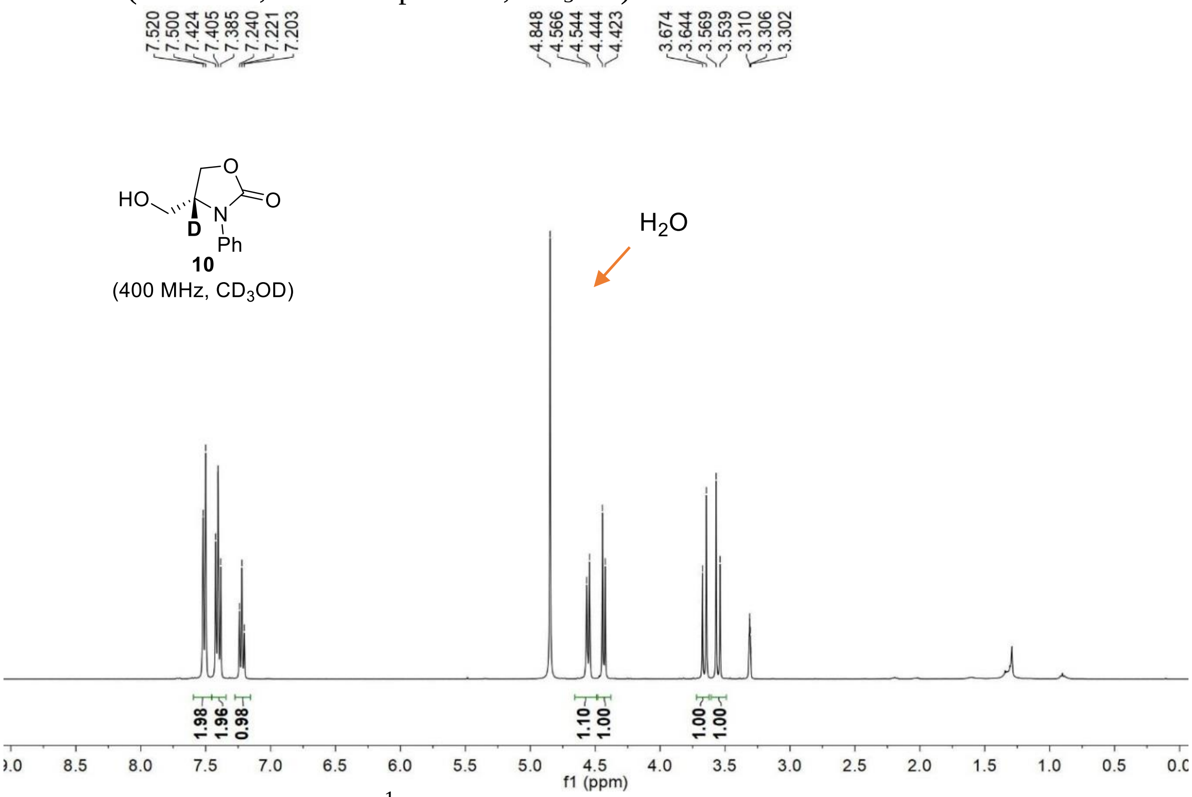

Supplementary Figure 282. ^{1}H NMR spectrum of compound **10**

^{13}C NMR (100 MHz, room temperature, CD_3OD)

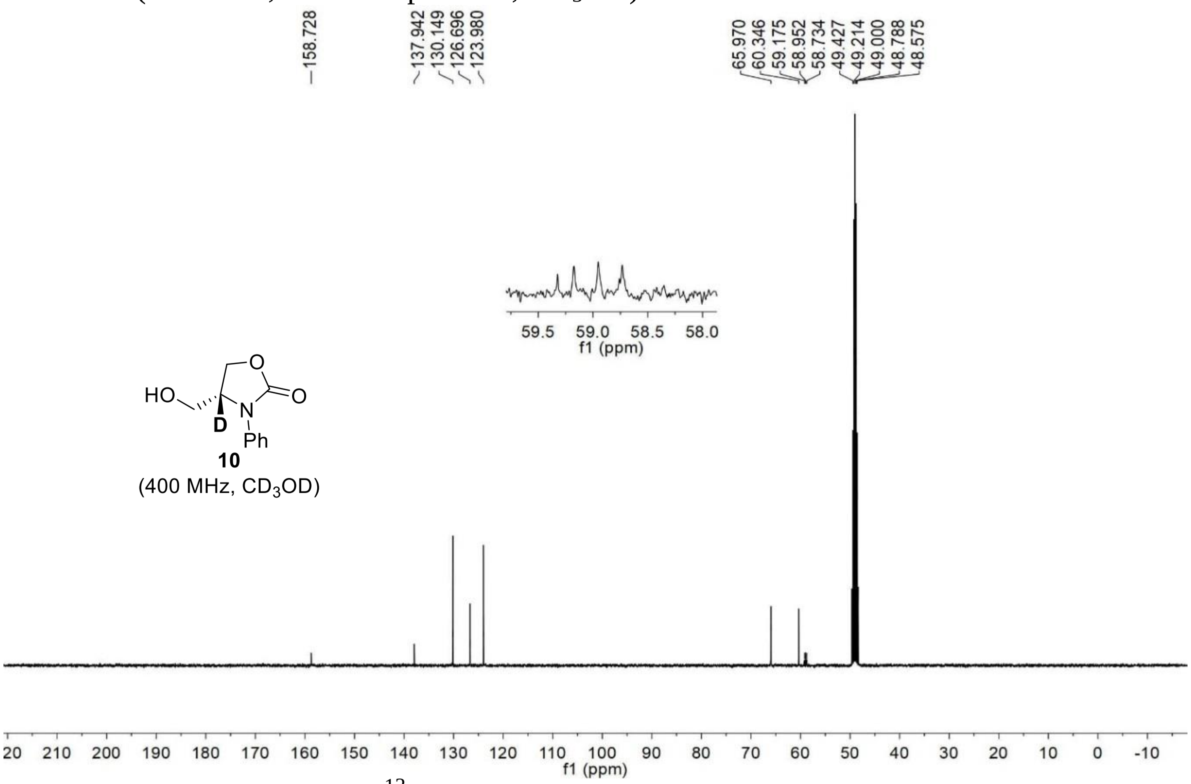

Supplementary Figure 283. ^{13}C NMR spectrum of compound **10**

HPLC spectrum of racemic **10**

Data File	: SQL-6-89-9.lcd
Method File	: 4OD-H-80-0.5-214.lcm
Date Acquired	: 9/1/2021 9:20:14 PM
Date Processed	: 9/1/2021 11:05:05 PM

<Chromatogram View>

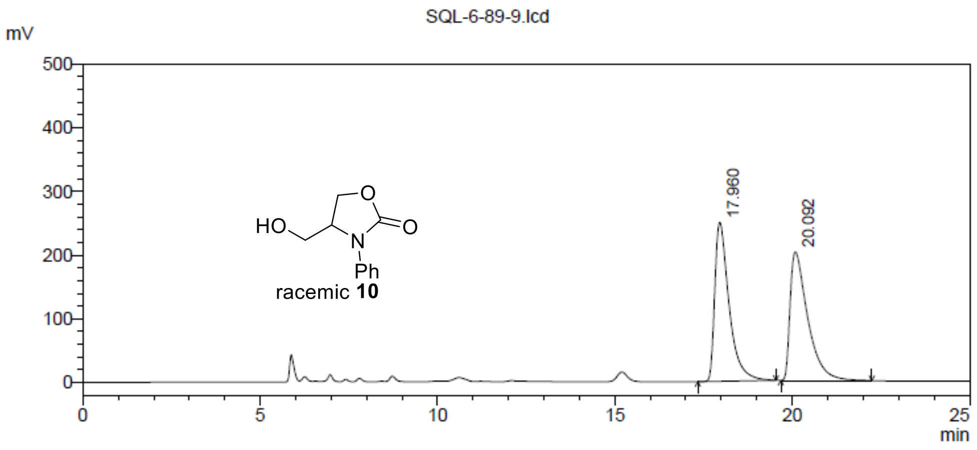

<Data Analysis>

Detector A 214nm

Pesk #	Ret. Time	Height	Area	Area%
1	17.960	249671	6989809	50.084
2	20.092	201908	6966295	49.916
Total		451579	13956103	100.000

Supplementary Figure 284. HPLC spectrum of racemic **10**

HPLC spectrum of **10**

Data File	: SQL-6-96.lcd
Method File	: 4OD-H-80-0.5-214.lcm
Date Acquired	: 9/1/2021 8:54:08 PM
Date Processed	: 9/1/2021 11:04:48 PM

<Chromatogram View>

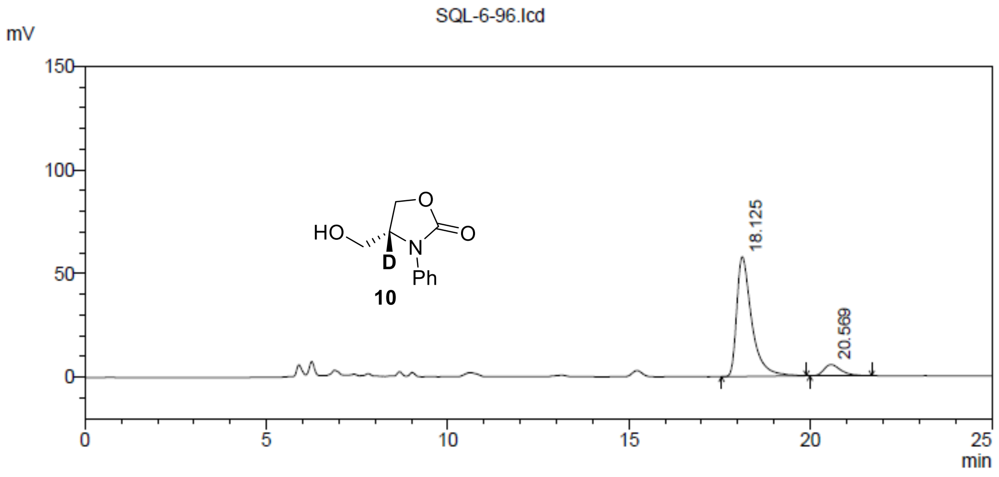

<Data Analysis>

Detector A 214nm

Pesk #	Ret. Time	Height	Area	Area%
1	18.125	57709	1657035	90.511
2	20.569	5353	173729	9.489
Total		63062	1830764	100.000

Supplementary Figure 285. HPLC spectrum of **10**

^{1}H NMR (400 MHz, room temperature, $CDCl_3$)

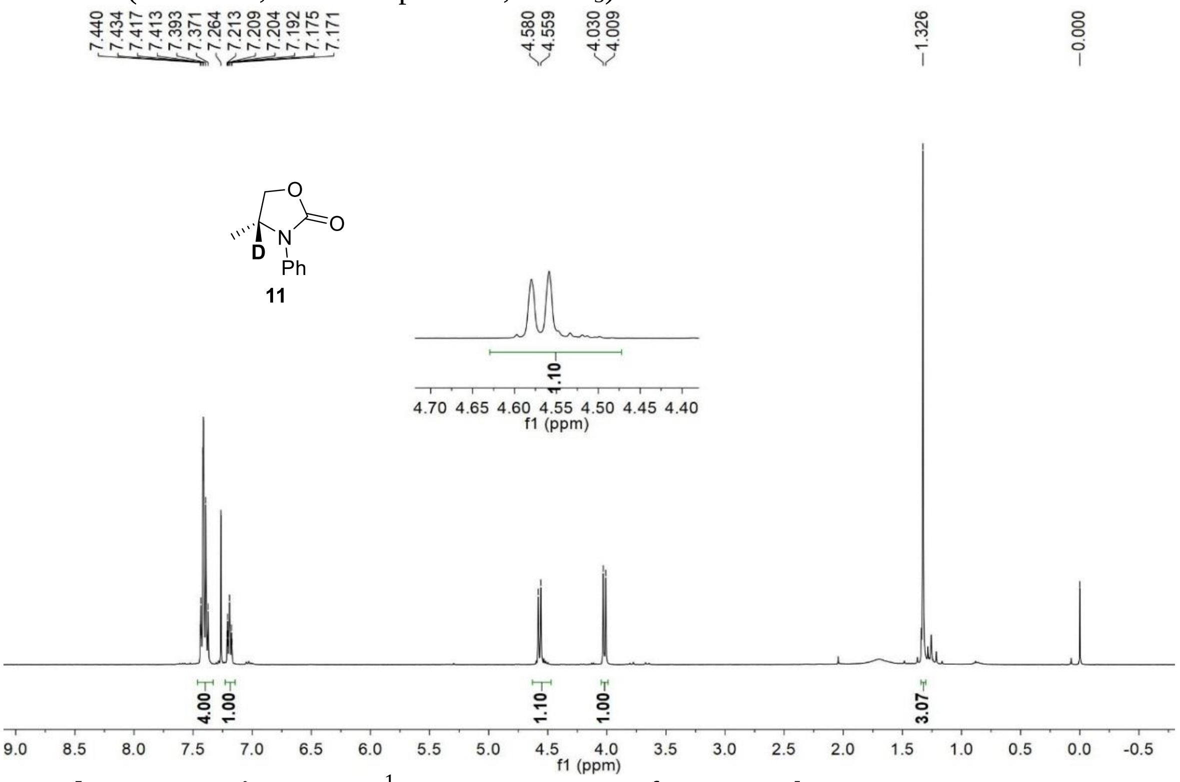

Supplementary Figure 286. ^{1}H NMR spectrum of compound **11**

^{13}C NMR (100 MHz, room temperature, $CDCl_3$)

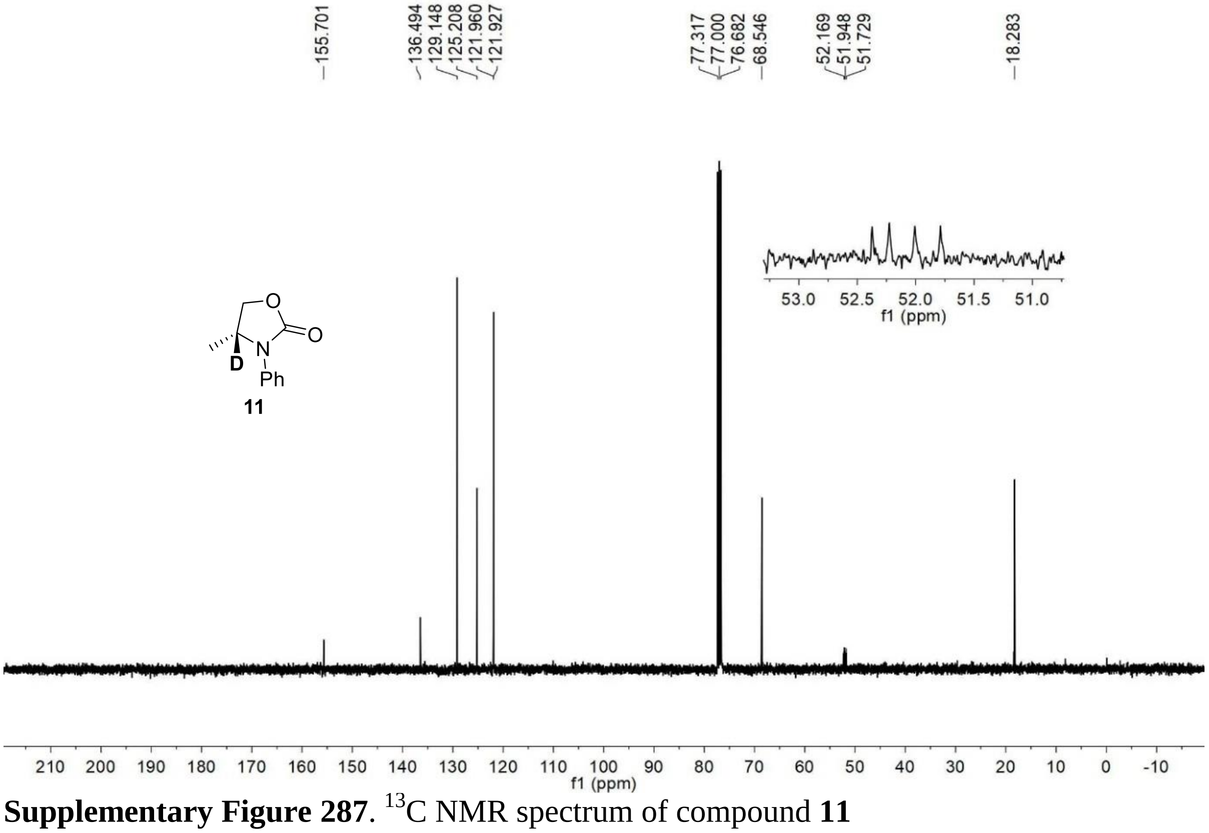

Supplementary Figure 287. ^{13}C NMR spectrum of compound **11**

HPLC spectrum of racemic **11**

Data File : sql-6-88-4.lcd
Method File : 3AD-H-90-1-214.lcm
Date Processed : 9/1/2021 4:27:09 PM

<Chromatogram View>

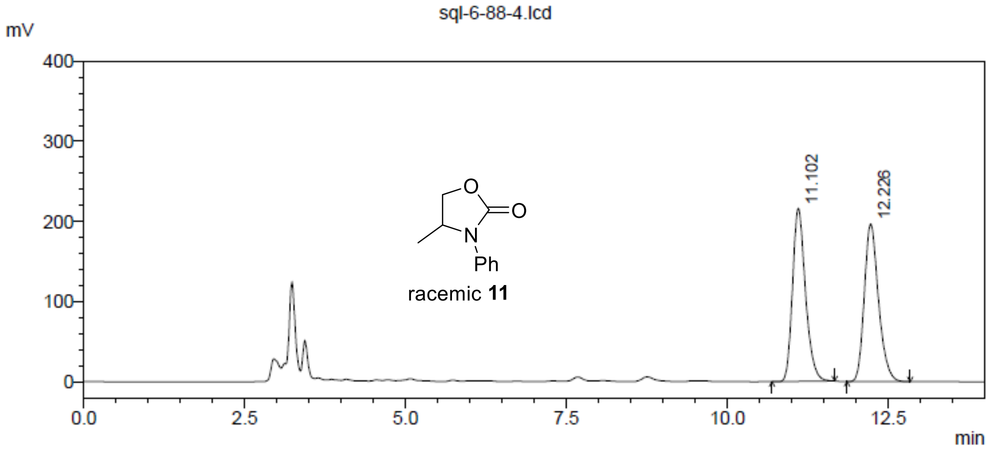

<Data Analysis>

Detector A 214nm

Pesk #	Ret. Time	Height	Area	Area%
1	11.102	215770	3035063	50.042
2	12.226	196721	3029964	49.958
Total		412491	6065028	100.000

Supplementary Figure 288. HPLC spectrum of racemic **11**

HPLC spectrum of **11**

Data File : sql-6-106.lcd
Method File : 3AD-H-90-1-214.lcm
Date Processed : 9/21/2021 4:03:02 PM

<Chromatogram View>

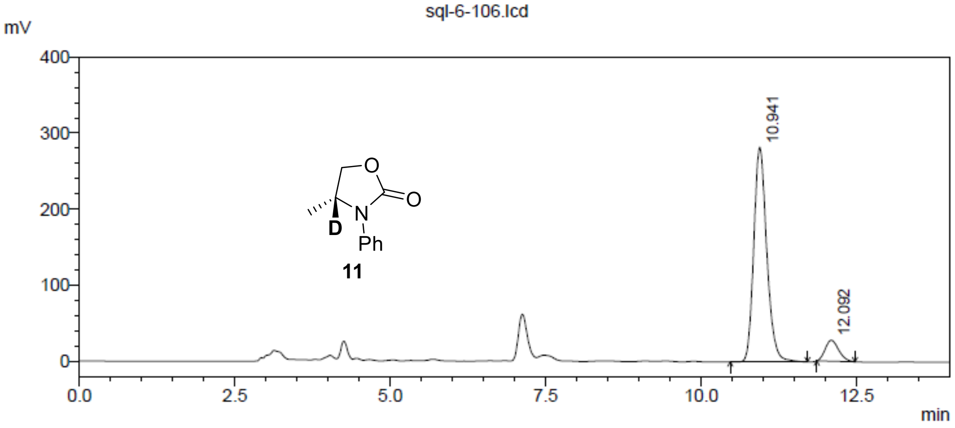

<Data Analysis>

Detector A 214nm

Pesk #	Ret. Time	Height	Area	Area%
1	10.941	282225	4104733	90.724
2	12.092	27718	419670	9.276
Total		309943	4524403	100.000

Supplementary Figure 289. HPLC spectrum of **11**

^{1}H NMR (400 MHz, room temperature, $CDCl_3$)

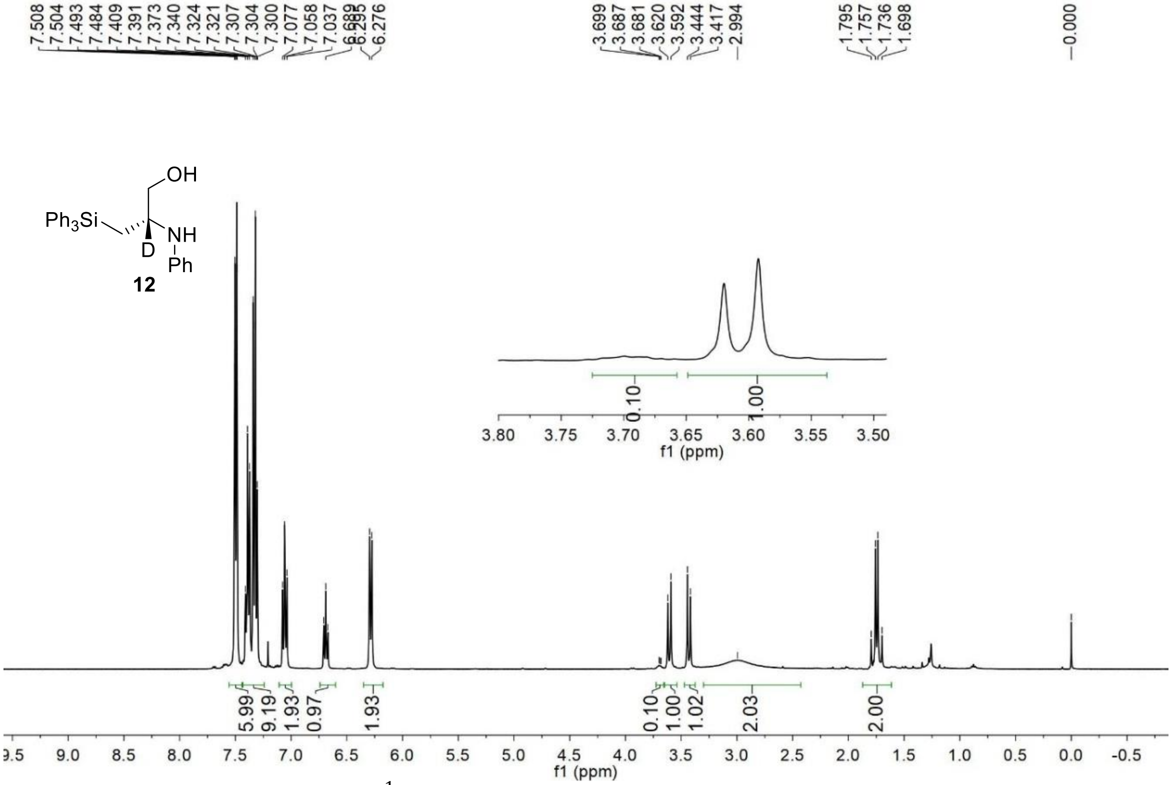

Supplementary Figure 290. ^{1}H NMR spectrum of compound **12**

^{13}C NMR (100 MHz, room temperature, $CDCl_3$)

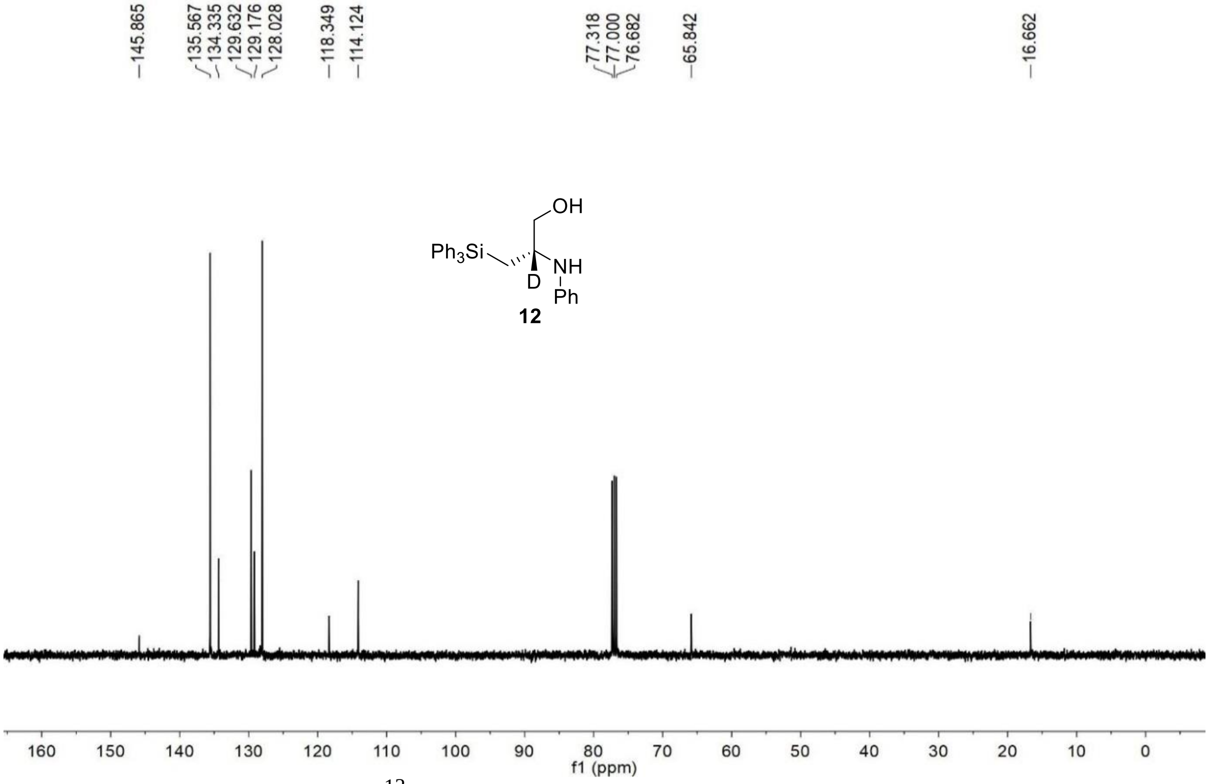

Supplementary Figure 291. ^{13}C NMR spectrum of compound **12**

HPLC spectrum of racemic **12**

Data File	: sql-6-97-1Rac-3.lcd
Method File	: 1OJ-H-85-1-214.lcm
Date Acquired	: 9/8/2021 10:31:41 PM
Date Processed	: 9/9/2021 10:27:51 PM

<Chromatogram View>

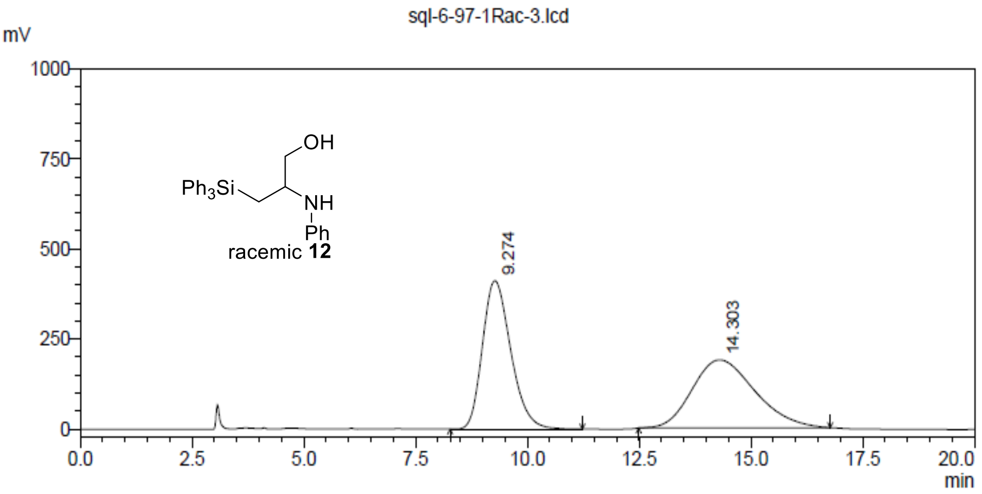

<Data Analysis>

Detector A 214nm

Pesk #	Ret. Time	Height	Area	Area%
1	9.274	411707	18182554	49.576
2	14.303	188759	18493694	50.424
Total		600466	36676248	100.000

Supplementary Figure 292. HPLC spectrum of racemic **12**

HPLC spectrum of **12**

Data File	: sql-6-97-2-chiral003.lcd
Method File	: 1OJ-H-85-1-214.lcm
Date Acquired	: 9/8/2021 10:00:59 PM
Date Processed	: 9/8/2021 11:30:18 PM

<Chromatogram View>

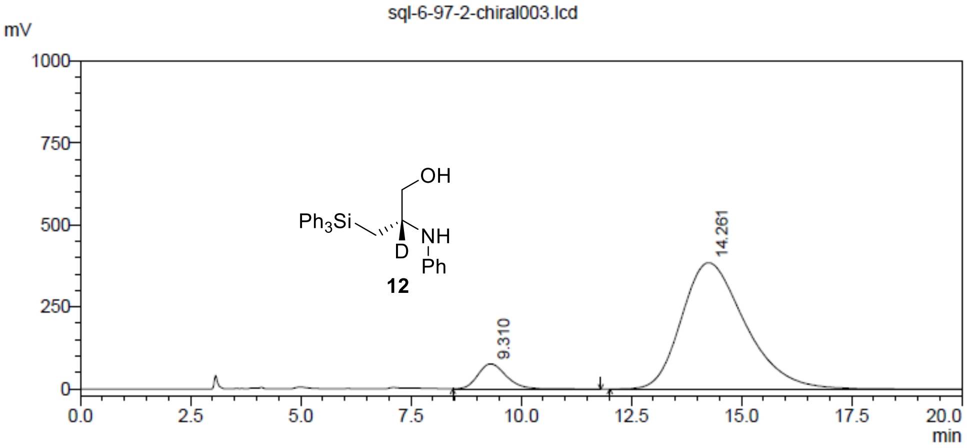

<Data Analysis>

Detector A 214nm

Pesk #	Ret. Time	Height	Area	Area%
1	9.310	76859	3466346	8.191
2	14.261	385056	38853332	91.809
Total		461915	42319679	100.000

Supplementary Figure 293. HPLC spectrum of **12**

3. Supplementary References

1. Luo, J., Zhang, J. Donor–acceptor fluorophores for visible-light-promoted organic synthesis: photoredox/Ni dual catalytic $C(sp^3)$–$C(sp^2)$ cross-coupling. *ACS Catal.* **6**, 873-877 (2016).
2. Garreau, M., Vaillant, F. L., Waser, J. C-terminal bioconjugation of peptides through photoredox catalyzed decarboxylative alkynylation. *Angew. Chem. Int. Ed.* **58**, 8182-8186 (2019).
3. Schoenauer, S., Schieberle, P. Structure–odor activity studies on monoterpenoid mercaptans synthesized by changing the structural motifs of the key food odorant 1-*p*-menthene-8-thiol. *J. Agric. Food Chem.* **64**, 3849-3861 (2016).
4. Pichota, A., V Gramlich, A., Beck K., Seebach, D. Bioinspired desaturation of alcohols enabled by photoredox proton-coupled electron transfer and cobalt dual catalysis. *Helv. Chim. Acta*. **95**, 1239-1272 (2012).
5. Seebach, D., Jaeschke, G., Pichota, A., Audergon, L. Enantioselective 1,4-addition of aliphatic grignard reagents to enones catalyzed by readily available copper(I) thiolates derived from TADDOL. Preliminary communication. *Helv. Chim. Acta.* **80**, 2515-2519 (1997).
6. Shin, N. Y., Ryss, J. M., Zhang, X., Miller, S. J., Knowles, R. R. Light-driven deracemization enabled by excited-state electron transfer. *Science* **366**, 364-369 (2019).
7. Featherston, A. L., Kwon, Y., Pompeo, M. M., Engl, O. D., Leahy, D. K., Miller, S. J. Catalytic asymmetric and stereodivergent oligonucleotide synthesis. *Science* **371**, 702-707 (2021).
8. Doyle, L. M., O’Sullivan, S., Salvo, C. D., McKinney, M., McArdle, P., Murphy, P.V. Stereoselective epimerizations of glycosyl thiols. *Org. Lett.* **19**, 5802-5805 (2017).
9. Abronina, P. I., Galkin, K. I., Backinowsky, L. V., Gracheva, A. A. Synthesis of a derivative of a pentasaccharide repeating unit of the O-antigenic polysaccharide of the bacterium Klebsiella pneumoniae O_3 as a benzoylated 2-methoxycarbonylethyl thioglycoside. *Russ. Chem. Bull.* **58**, 457-467 (2009).
10. Cai, Y., Roberts, B. P., Tocher, D. A. Carbohydrate-derived thiols as protic polarity-reversal catalysts for enantioselective radical-chain reactions. *J. Chem. Soc., Perkin Trans.*1, 1376-1386 (2002).

11. Haque, M. B., Roberts, B. P., Tocher, D. A. Enantioselective radical-chain hydrosilylation of alkenes using homochiral thiols as polarity-reversal catalysts. *J. Chem. Soc., Perkin Trans.*1, 2881-2889 (1998).
12. Larsen, J., Bechgaard, K. Thiaheterohelicenes 2. Synthesis of alkylated thiaheterohelicenes. *Acta Chem. Scand.* **50**, 77-82 (1996).
13. Wang, J. L., Li, H. J., Wang, H. S., Wu, Y. C. Regioselective 1,2-diol rearrangement by controlling the loading of $BF_3 \cdot Et_2O$ and its application to the synthesis of related nor-sesquiterene- and sesquiterene-type marine natural products. *Org. Lett.* **19**, 3811-3814 (2017).
14. Ramesh, R., Chandrasekaran, Y., Megha R., Chandrasekaran, S. Base catalyzed cyclization of N-aryl and N-alkyl-O-propargyl carbamates to 4-alkylidene-2-oxazolidinones. *Tetrahedron* **63**, 9153-9162 (2007).
15. Hu, T., Li, C. Synthesis of lactams via copper-catalyzed intramolecular vinylation of amides. *Org. Lett.* **7**, 2035-2038 (2005).
16. Solovyev, A., Ueng, S., Monot, J., Fensterbank, L., Malacria, M., Lacôte, E., and Curran, D. P. Estimated rate constants for hydrogen abstraction from N-heterocyclic carbene−borane complexes by an alkyl radical. *Org. Lett.* **12**, 2998–3001 (2010).
17. Gardner, S., Kawamoto, T., Curran, D. P. Synthesis of 1,3-dialkylimidazol-2-ylidene boranes from 1,3-dialkylimidazolium iodides and sodium borohydride. *J. Org. Chem.* **80**, 9794-9797 (2015).
18. Cismesia, M. A., Yoon, T. P. Characterizing chain processes in visible light photoredox catalysis. *Chem. Sci.* **6**, 5426-5434 (2015).
19. Ham,W. S., Hillenbrand, J., Jacq, J., Genicot, C., Ritter, T. Divergent late-stage (hetero)aryl C−H amination by the pyridinium radical cation. *Angew. Chem. Int. Ed.* **58**, 532-536 (2019).
20. Luis-Barrera, J., LainaMartin, V., Rigotti, T., Peccati, F., Solans-Monfort, X., Sodupe, M., Mas-Balleste, R., Liras, M., Alemán, J. Visible-light photocatalytic intramolecular cyclopropane ring expansion. *Angew. Chem. Int. Ed.* **56**, 7826-7830 (2017).
21. Klauck, F. J. R., James, M. J., Glorius, F. Deaminative strategy for the visible-light-mediated generation of alkyl radicals. *Angew. Chem. Int. Ed.* **56**, 12336-12339 (2017).
22. Quantum yield for the ferrioxalate actinometer was reported to be 1.11 at 436 nm. We thus chose 1.11 as quantum yield for the ferrioxalate actinometer at 441 nm to determine the

photo flux of the light source. For details, see: Kuhn, H. J., Braslavsky, S. E., Schmidt, R. Chemical actinometry (IUPAC technical report). *Pure Appl. Chem.* **76**, 2105-2146 (2004).

23. Monalti, M., Credi, A., Prodi, L., Gandolfi, M. T. *Chemical Actinometry. Handbook of Photochemistry, 3rd Ed.* 601-604 (2006).
24. Gaussian 16, Revision C.01, Frisch, M. J., Trucks, G. W., Schlegel, H. B., Scuseria, G. E., Robb, M. A., Cheeseman, J. R., Scalmani, G., Barone, V., Petersson, G. A., Nakatsuji, H., Li, X., Caricato, M., Marenich, A. V., Bloino, J., Janesko, B. G., Gomperts, R., Mennucci, B., Hratchian, H. P., Ortiz, J. V., Izmaylov, A. F., Sonnenberg, J. L., Williams-Young, D., Ding, F., Lipparini, F., Egidi, F., Goings, J., Peng, B., Petrone, A., Henderson, T., Ranasinghe, D., Zakrzewski, V. G., Gao, J., Rega, N., Zheng, G., Liang, W., Hada, M., Ehara, M., Toyota, K., Fukuda, R., Hasegawa, J., Ishida, M., Nakajima, T., Honda, Y., Kitao, O., Nakai, H., Vreven, T., Throssell, K., Montgomery, J. A., Jr., Peralta, J. E., Ogliaro, F., Bearpark, M. J., Heyd, J. J., Brothers, E. N., Kudin, K. N., Staroverov, V. N., Keith, T. A., Kobayashi, R., Normand, J., Raghavachari, K., Rendell, A. P., Burant, J. C., Iyengar, S. S., Tomasi, J., Cossi, M., Millam, J. M., Klene, M., Adamo, C., Cammi, R., Ochterski, J. W., Martin, R. L., Morokuma, K., Farkas, O., Foresman, J. B., Fox, D. J., *Gaussian, Inc., Wallingford CT* (2016).
25. Chai, J. D., Head-Gordon, M. Long-range corrected hybrid density functionals with damped atom–atom dispersion corrections. *Phys. Chem. Chem. Phys*. **10**, 6615-6620 (2008).
26. Luchini, G., Alegre-Requena, J. V., Funes-Ardoiz, I., Paton, R. S. GoodVibes: automated thermochemistry for heterogeneous computational chemistry data [version 1; peer review: 2 approved with reservations]. *F1000Research*. **9**, 291, 1-14, (2020).
27. Grimme, S. Supramolecular binding thermodynamics by dispersion-corrected density functional theory. *Chem. Eur. J.* **18**, 9955-9964 (2012).
28. Adamo C., Barone, V. Toward reliable density functional methods without adjustable parameters: The PBE0 model. *J. Chem. Phys.* **110**, 6158-6169 (1999).
29. Minenkov, Y., Singstad, Å., Occhipinti G., Jensen, V. R. The accuracy of DFT-optimized geometries of functional transition metal compounds: a validation study of catalysts for olefin metathesis and other reactions in the homogeneous phase. *Dalton Trans.* **41**, 5526-5541 (2012).
30. Barone, V., Cossi, M. Quantum calculation of molecular energies and energy gradients in solution by a conductor solvent model. *J. Phys. Chem. A.* **102**, 1995-2001 (1998).

31. Cossi, M., Rega, N., Scalmani, G., Barone, V. Energies, structures, and electronic properties of molecules in solution with the C-PCM solvation model. *J. Comp. Chem.* **24**, 669-681 (2003).
32. Metrano, A. J., Abascal, N. C., Mercado, B. Q., Paulson, E. K., Hurtley, A. E., Miller, S. J. Energies, structures, and electronic properties of molecules in solution with the C-PCM solvation model. *J. Am. Chem. Soc.* **139**, 492-516 (2017).
33. Johnson, E. R., Keinan, S., Mori-Sanchez P., Contreras-Garcia J., Cohen, A. J., Yang W. Revealing noncovalent interactions. *J. Am. Chem. Soc.* **132**, 6498-6506 (2010).
34. Contreras-Garcia, J., Johnson, E. R., Keinan, S., Chaudret, R., Piquemal, J. P., Beratan, D. N., Yang, W. NCIPLOT: a program for plotting noncovalent interaction regions. *J. Chem. Theory Comput*. **7**, 625-632 (2011).
