# Supplementary material for: Visible-light mediated catalytic asymmetric radical deuteration at non-benzylic positions
Source: Nat Commun. 2022 Aug 1;13:4453. doi: 10.1038/s41467-022-32238-8 (PMC9343372; doi:10.1038/s41467-022-32238-8)
Supplement: Supplementary file 2 — Description of Additional Supplementary Files [file 41467_2022_32238_MOESM2_ESM.pdf]

Supplementary Data File 1: XYZ Coordinates and Energies of the Calculated Species
