# Supplementary material for: Visible-light mediated catalytic asymmetric radical deuteration at non-benzylic positions
Source: Nat Commun. 2022 Aug 1;13:4453. doi: 10.1038/s41467-022-32238-8 (PMC9343372; doi:10.1038/s41467-022-32238-8)
Supplement: Supplementary file 3 — Supplementary Data 1 [file 41467_2022_32238_MOESM3_ESM.pdf]

XYZ Coordinates and Energies of the Calculated Species

Final free energies are calculated as the sum of E (large basis set) + G^{D}_{Corr}. G^{D}_{Corr} refers to the free energy correction of deuterated species. Energies for Distortion-Interaction analysis are included in the **TS-*Re*** and **TS-*Si*** as fragments 1 (radical) and 2 (RS-D).

TS-*Si*

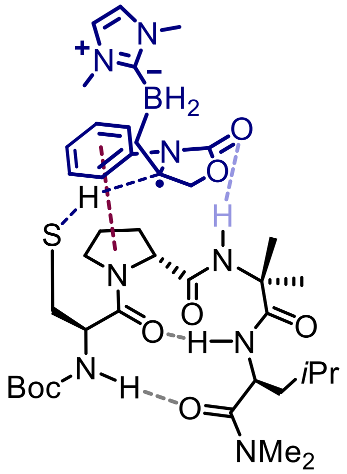

E (PBE0) = -3023.61556

E(rad-dist/int) = -921.8277869

E(RSD-dist/int) = -2101.742878

G^{D}_{Corr}= 0.963495

E(M062X) = -3025.556844

E(wB97xD) = -3025.868055

O	-3.32592400	1.17709300	-1.70481900
O	-1.66682500	4.87907600	1.29303500
O	-1.98536000	1.01539000	2.61029400
O	-0.21643300	0.23676000	-0.36083000
O	-3.38185900	-3.74398000	-0.64517700
O	-5.03512400	-2.16594200	-0.70390700
N	-4.28303300	2.17170300	0.08186400
N	-1.31734100	2.90617200	0.22221900
H	-0.82336900	2.01651300	0.21427000
N	0.10710000	1.88962100	2.32772600
H	1.05909800	1.64779400	2.06455900
N	-0.80840800	-1.27967200	1.19487500
N	-2.93907200	-1.51302800	-0.89773100
H	-3.34854800	-0.60128900	-1.10758900
C	-4.26905200	3.09875600	1.20558900
H	-3.75907000	2.64582700	2.06320300
H	-5.30213400	3.32954500	1.47579900
H	-3.76565100	4.03007000	0.95579600

C	-5.23248800	1.07213300	0.19691400
H	-5.41607200	0.62908700	-0.78035300
H	-6.17111600	1.45590600	0.60247700
H	-4.84838100	0.29357100	0.86662800
C	-3.33004400	2.09562100	-0.87088900
C	-2.20724100	3.15655800	-0.89973800
H	-2.63241400	4.15123900	-0.75103500
C	-1.14651500	3.76233700	1.24655900
C	-0.20659200	3.31517300	2.38806100
C	-0.77868000	0.87587400	2.41998600
C	-0.16064500	-0.52158000	2.27220400
H	0.90742500	-0.43983000	2.05282500
C	-0.39776500	-1.38316200	3.52234000
H	0.44196700	-1.31449600	4.21661100
H	-1.30310400	-1.03900200	4.03159300
C	-0.61218200	-2.79130900	2.96298100
H	0.34812800	-3.25645600	2.71818200
H	-1.15079600	-3.44488000	3.65225400
C	-1.39911100	-2.53023900	1.68269200
H	-1.30331100	-3.32898000	0.94721300
H	-2.46541400	-2.38139300	1.88926200
C	-0.77801000	-0.83110500	-0.06980700
C	-1.51718700	-1.65368300	-1.13870100
H	-1.27324300	-2.71327100	-1.03098600
C	-3.75806600	-2.58354400	-0.74605500
C	-6.13108600	-3.06652900	-0.36194400
C	-7.33719900	-2.13080500	-0.35858300
H	-7.20947000	-1.34288300	0.38952500
H	-8.24442700	-2.69337600	-0.12105500
H	-7.46286500	-1.66376100	-1.33953700
C	-5.90784300	-3.65700400	1.02929700
H	-5.05565100	-4.33803800	1.03879700
H	-6.80157500	-4.20928100	1.33504800
H	-5.73465300	-2.85649200	1.75559300
C	-6.28717900	-4.14376900	-1.43311000
H	-6.38904600	-3.68267100	-2.42013500
H	-7.19190700	-4.72548300	-1.23117900
H	-5.42938900	-4.81716500	-1.44205100
C	-1.45115100	3.10640100	-2.22934800
H	-2.15454800	3.36349400	-3.03148700
H	-1.14120000	2.07195900	-2.40838800
C	-0.22320400	4.02209800	-2.30686200
H	0.50058900	3.67578500	-1.55672000
C	-0.55378900	5.48731400	-2.00850400

H	-1.33062100	5.85246400	-2.69269900
H	0.33204300	6.11688500	-2.14472700
H	-0.90865700	5.62743900	-0.98332000
C	0.42460300	3.89007300	-3.68864000
H	0.63809100	2.84543800	-3.93958600
H	1.36505400	4.44885100	-3.73689000
H	-0.24104200	4.28555400	-4.46553200
C	-1.16561800	-1.19970000	-2.55601800
H	-1.92475800	-1.60627000	-3.22983000
H	-1.20866600	-0.11196100	-2.62472600
C	1.11450800	4.07411000	2.18923600
H	0.92366600	5.14854200	2.21038700
H	1.81349700	3.82196600	2.99231200
H	1.57620800	3.81477600	1.23142400
C	-0.83446700	3.68150500	3.73793700
H	-0.13604300	3.41924300	4.53793700
H	-1.03544000	4.75329100	3.77283800
H	-1.76929100	3.13959200	3.89197600
S	0.41439900	-1.82882500	-3.19993200
H	1.41985400	-1.07352600	-2.31497800
C	2.60422900	-0.41738100	-1.80096500
C	2.30866300	1.04475500	-2.07647400
C	2.61246200	0.79662600	0.16129800
N	2.67303600	-0.46448500	-0.37908600
H	3.01596200	1.48683600	-2.77984400
C	2.75564600	-1.65454100	0.38870200
C	3.44552500	-1.68141500	1.60343000
C	2.14221100	-2.81753300	-0.08625000
C	3.52054200	-2.86679300	2.32872900
H	3.92241500	-0.78462400	1.97440600
C	2.23326500	-3.99880300	0.64284400
H	1.59301700	-2.80600900	-1.02115500
C	2.92228000	-4.03198500	1.85343400
H	4.06118400	-2.87584700	3.27003800
H	1.75267800	-4.89358400	0.26049800
H	2.98778500	-4.95373100	2.42218500
C	3.68122800	-1.12523400	-2.57218500
H	3.66554400	-2.19212200	-2.31848200
H	3.42234900	-1.06229700	-3.63568700
O	2.46018000	1.70367700	-0.80454600
O	2.71570000	1.10720700	1.33705200
H	1.27999400	1.20838300	-2.40787900
B	5.18883600	-0.46779400	-2.39152400
H	5.96631500	-1.11085200	-3.08449700

H	5.19858400	0.69487900	-2.75673000
C	5.76208200	-0.54805100	-0.88216400
N	6.15254900	-1.67422700	-0.24201700
N	6.03833200	0.45843500	-0.02040400
C	6.67499400	-1.37832100	0.99859500
C	5.96474100	-3.02550900	-0.74742000
C	6.60707400	-0.03390200	1.13826400
C	5.80377400	1.88060500	-0.23817000
H	7.03190200	-2.14333600	1.66871400
H	6.75617000	-3.66352800	-0.35338300
H	4.99091200	-3.40915600	-0.43152300
H	6.01846600	-3.00662300	-1.83541600
H	6.90342000	0.61159000	1.94911400
H	5.15888200	2.26436700	0.55455300
H	6.75600600	2.41435700	-0.23596400
H	5.31684400	2.01300800	-1.20065500

TS-*Re*

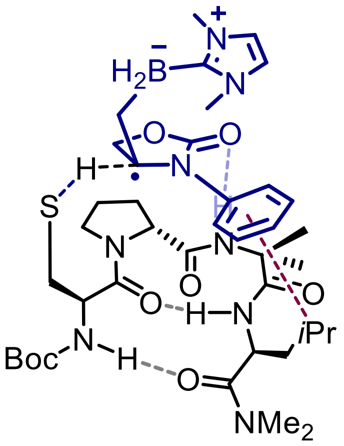

E (PBE0) = -3023.611538

E(rad-dist/int) = -921.828477

E(RSD-dist/int) = -2101.74075

G^{D}_{Corr}= 0.961447

E(M062X) = -3025.551376

E(wB97xD) = -3025.862599

O	-3.32592400	1.17709300	-1.70481900
O	-1.66682500	4.87907600	1.29303500
O	-1.98536000	1.01539000	2.61029400
O	-0.21643300	0.23676000	-0.36083000
O	-3.38185900	-3.74398000	-0.64517700
O	-5.03512400	-2.16594200	-0.70390700

N	-4.28303300	2.17170300	0.08186400
N	-1.31734100	2.90617200	0.22221900
H	-0.82336900	2.01651300	0.21427000
N	0.10710000	1.88962100	2.32772600
H	1.05909800	1.64779400	2.06455900
N	-0.80840800	-1.27967200	1.19487500
N	-2.93907200	-1.51302800	-0.89773100
H	-3.34854800	-0.60128900	-1.10758900
C	-4.26905200	3.09875600	1.20558900
H	-3.75907000	2.64582700	2.06320300
H	-5.30213400	3.32954500	1.47579900
H	-3.76565100	4.03007000	0.95579600
C	-5.23248800	1.07213300	0.19691400
H	-5.41607200	0.62908700	-0.78035300
H	-6.17111600	1.45590600	0.60247700
H	-4.84838100	0.29357100	0.86662800
C	-3.33004400	2.09562100	-0.87088900
C	-2.20724100	3.15655800	-0.89973800
H	-2.63241400	4.15123900	-0.75103500
C	-1.14651500	3.76233700	1.24655900
C	-0.20659200	3.31517300	2.38806100
C	-0.77868000	0.87587400	2.41998600
C	-0.16064500	-0.52158000	2.27220400
H	0.90742500	-0.43983000	2.05282500
C	-0.39776500	-1.38316200	3.52234000
H	0.44196700	-1.31449600	4.21661100
H	-1.30310400	-1.03900200	4.03159300
C	-0.61218200	-2.79130900	2.96298100
H	0.34812800	-3.25645600	2.71818200
H	-1.15079600	-3.44488000	3.65225400
C	-1.39911100	-2.53023900	1.68269200
H	-1.30331100	-3.32898000	0.94721300
H	-2.46541400	-2.38139300	1.88926200
C	-0.77801000	-0.83110500	-0.06980700
C	-1.51718700	-1.65368300	-1.13870100
H	-1.27324300	-2.71327100	-1.03098600
C	-3.75806600	-2.58354400	-0.74605500
C	-6.13108600	-3.06652900	-0.36194400
C	-7.33719900	-2.13080500	-0.35858300
H	-7.20947000	-1.34288300	0.38952500
H	-8.24442700	-2.69337600	-0.12105500
H	-7.46286500	-1.66376100	-1.33953700
C	-5.90784300	-3.65700400	1.02929700
H	-5.05565100	-4.33803800	1.03879700

H	-6.80157500	-4.20928100	1.33504800
H	-5.73465300	-2.85649200	1.75559300
C	-6.28717900	-4.14376900	-1.43311000
H	-6.38904600	-3.68267100	-2.42013500
H	-7.19190700	-4.72548300	-1.23117900
H	-5.42938900	-4.81716500	-1.44205100
C	-1.45115100	3.10640100	-2.22934800
H	-2.15454800	3.36349400	-3.03148700
H	-1.14120000	2.07195900	-2.40838800
C	-0.22320400	4.02209800	-2.30686200
H	0.50058900	3.67578500	-1.55672000
C	-0.55378900	5.48731400	-2.00850400
H	-1.33062100	5.85246400	-2.69269900
H	0.33204300	6.11688500	-2.14472700
H	-0.90865700	5.62743900	-0.98332000
C	0.42460300	3.89007300	-3.68864000
H	0.63809100	2.84543800	-3.93958600
H	1.36505400	4.44885100	-3.73689000
H	-0.24104200	4.28555400	-4.46553200
C	-1.16561800	-1.19970000	-2.55601800
H	-1.92475800	-1.60627000	-3.22983000
H	-1.20866600	-0.11196100	-2.62472600
C	1.11450800	4.07411000	2.18923600
H	0.92366600	5.14854200	2.21038700
H	1.81349700	3.82196600	2.99231200
H	1.57620800	3.81477600	1.23142400
C	-0.83446700	3.68150500	3.73793700
H	-0.13604300	3.41924300	4.53793700
H	-1.03544000	4.75329100	3.77283800
H	-1.76929100	3.13959200	3.89197600
S	0.41439900	-1.82882500	-3.19993200
H	1.41985400	-1.07352600	-2.31497800
C	2.60422900	-0.41738100	-1.80096500
C	2.30866300	1.04475500	-2.07647400
C	2.61246200	0.79662600	0.16129800
N	2.67303600	-0.46448500	-0.37908600
H	3.01596200	1.48683600	-2.77984400
C	2.75564600	-1.65454100	0.38870200
C	3.44552500	-1.68141500	1.60343000
C	2.14221100	-2.81753300	-0.08625000
C	3.52054200	-2.86679300	2.32872900
H	3.92241500	-0.78462400	1.97440600
C	2.23326500	-3.99880300	0.64284400
H	1.59301700	-2.80600900	-1.02115500

C	2.92228000	-4.03198500	1.85343400
H	4.06118400	-2.87584700	3.27003800
H	1.75267800	-4.89358400	0.26049800
H	2.98778500	-4.95373100	2.42218500
C	3.68122800	-1.12523400	-2.57218500
H	3.66554400	-2.19212200	-2.31848200
H	3.42234900	-1.06229700	-3.63568700
O	2.46018000	1.70367700	-0.80454600
O	2.71570000	1.10720700	1.33705200
H	1.27999400	1.20838300	-2.40787900
B	5.18883600	-0.46779400	-2.39152400
H	5.96631500	-1.11085200	-3.08449700
H	5.19858400	0.69487900	-2.75673000
C	5.76208200	-0.54805100	-0.88216400
N	6.15254900	-1.67422700	-0.24201700
N	6.03833200	0.45843500	-0.02040400
C	6.67499400	-1.37832100	0.99859500
C	5.96474100	-3.02550900	-0.74742000
C	6.60707400	-0.03390200	1.13826400
C	5.80377400	1.88060500	-0.23817000
H	7.03190200	-2.14333600	1.66871400
H	6.75617000	-3.66352800	-0.35338300
H	4.99091200	-3.40915600	-0.43152300
H	6.01846600	-3.00662300	-1.83541600
H	6.90342000	0.61159000	1.94911400
H	5.15888200	2.26436700	0.55455300
H	6.75600600	2.41435700	-0.23596400
H	5.31684400	2.01300800	-1.20065500

Radical Adduct

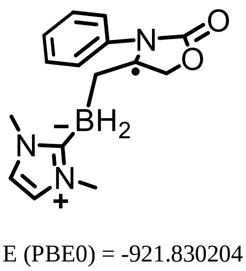

E (PBE0) = -921.830204

G^{D}_{Corr}= 0.287218

C	1.08522400	-1.46238200	1.21050000

C	2.48293200	-1.87898300	0.86290700
C	1.30302600	-1.62496300	-1.08441100
N	0.43174400	-1.38473700	-0.04150400
H	3.23087300	-1.16795400	1.23002300
C	-0.96299000	-1.20439900	-0.20071300
C	-1.47788300	-0.46580300	-1.26747100
C	-1.82925500	-1.75848600	0.74668400
C	-2.85263900	-0.27610400	-1.37316600
H	-0.80608800	-0.04215800	-2.00253700
C	-3.20113700	-1.55479700	0.63671700
H	-1.42151000	-2.34628800	1.56219300
C	-3.72018900	-0.81061000	-0.42200800
H	-3.24490900	0.30146000	-2.20445500
H	-3.86537100	-1.98661400	1.37855900
H	-4.79057700	-0.65410700	-0.50781800
C	0.79096700	-0.48268000	2.29113300
H	-0.28330600	-0.46539600	2.50975700
H	1.28246200	-0.82673300	3.20912100
O	2.52525700	-1.88449300	-0.58018800
O	1.07142100	-1.61140000	-2.27506800
H	2.74917200	-2.88660000	1.20230400
B	1.35948600	1.04534200	1.97570700
H	1.06276000	1.78000700	2.90975500
H	2.57318000	1.03282200	1.84664400
C	0.70023100	1.69388900	0.65065600
N	-0.56028400	2.17400700	0.54430600
N	1.26944400	1.93765600	-0.55272000
C	-0.77480300	2.72120600	-0.70250700
C	-1.59214500	2.02813600	1.55893900
C	0.37905100	2.57590400	-1.39435800
C	2.62079900	1.57747500	-0.96075800
H	-1.72345400	3.14605600	-0.98759200
H	-2.27653100	2.87448500	1.49356500
H	-2.14066300	1.09630100	1.39719800
H	-1.12104000	2.01232500	2.54104200
H	0.64410800	2.85759200	-2.40050300
H	2.57388000	0.89277500	-1.80996700
H	3.17212300	2.47695100	-1.24113300
H	3.12056300	1.09260200	-0.126085000

Peptide Catalyst S6

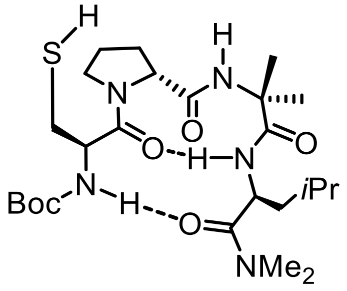

E (PBE0) = -2101.758765

G^{D}_{Corr}= 0.648984

O	-0.23509400	-2.17782100	-0.53133100
O	4.09935600	-0.05858600	-2.07207100
O	0.98975200	2.34117600	-1.38144000
O	0.72608300	0.41225200	1.74143700
O	-4.33935000	0.17146400	1.13722400
O	-3.88490200	-1.02906900	-0.75453800
N	0.24704800	-1.11242700	-2.46602100
N	2.46341800	-0.34042600	-0.52078400
H	1.96722300	0.05480600	0.27594400
N	2.59866700	2.29170900	0.23368600
H	2.72011400	2.29194000	1.23630400
N	-0.73226500	1.98340900	1.05607900
N	-2.24105300	-0.59706900	0.64953600
H	-1.69036700	-1.20426900	0.03857500
C	1.14539100	-0.37259400	-3.34473500
H	1.11973600	0.69506300	-3.10241700
H	0.82052200	-0.52249200	-4.37703300
H	2.17268200	-0.71964300	-3.25736900
C	-1.17354500	-0.95989300	-2.75233300
H	-1.73639400	-1.78320900	-2.31558200
H	-1.31814900	-0.95582100	-3.83462000
H	-1.55657100	-0.01873800	-2.33983800
C	0.60686900	-1.68688100	-1.29996700
C	2.09537500	-1.69493500	-0.90211000
H	2.70965700	-1.96043500	-1.76433700
C	3.42462300	0.37344500	-1.13721000
C	3.71367100	1.78164600	-0.56566000
C	1.33875400	2.49736400	-0.21554900
C	0.34009000	2.96533300	0.85294600
H	0.85485300	3.11262500	1.80943900
C	-0.40288100	4.23753000	0.41273900
H	0.08421000	5.13697100	0.79349300

H	-0.41261300	4.28142200	-0.68011300
C	-1.82690500	4.04306500	0.94125800
H	-1.88571300	4.30057700	2.00383300
H	-2.56252600	4.64197800	0.40087600
C	-2.05429900	2.54585600	0.75848300
H	-2.82205700	2.14019200	1.41700200
H	-2.33030600	2.30553400	-0.27528500
C	-0.44615700	0.76893000	1.55954800
C	-1.62278500	-0.16701100	1.88399800
H	-2.37962600	0.38509000	2.44740200
C	-3.56623500	-0.43722000	0.40807800
C	-5.20121300	-0.86993500	-1.36681400
C	-5.04894000	-1.63424400	-2.67920900
H	-4.26201500	-1.18842000	-3.29461200
H	-5.98799900	-1.60301700	-3.23870200
H	-4.79189100	-2.67969400	-2.48683500
C	-5.47586200	0.60830400	-1.63709500
H	-5.60113800	1.16521500	-0.70748100
H	-6.39129600	0.70296200	-2.22877000
H	-4.65193000	1.04665900	-2.20930200
C	-6.27479400	-1.51148300	-0.49071800
H	-6.01368800	-2.55177300	-0.27445400
H	-7.22968100	-1.50174500	-1.02516600
H	-6.39184700	-0.97092500	0.44915800
C	2.35354000	-2.67636000	0.24181100
H	2.04142200	-3.67638300	-0.08414300
H	1.70396400	-2.40547700	1.08062500
C	3.81070100	-2.72361000	0.71854300
H	4.09271900	-1.71277800	1.04306400
C	4.77870100	-3.15403900	-0.38761200
H	4.48816300	-4.13075500	-0.79507900
H	5.79640400	-3.24672600	0.00604300
H	4.80694900	-2.43195900	-1.20905900
C	3.92171200	-3.65190600	1.93115000
H	3.25155700	-3.33564800	2.73757600
H	4.94357300	-3.66400400	2.32421300
H	3.65625400	-4.68037400	1.65707400
C	-1.16169700	-1.36816200	2.70917900
H	-1.98055800	-2.08621500	2.77439400
H	-0.31745300	-1.85442800	2.21763800
C	4.91582400	1.63549400	0.37942800
H	5.76841600	1.23541200	-0.17237800
H	5.19117400	2.61242900	0.78834500
H	4.68972300	0.95416800	1.20662400

C	4.04952800	2.74697700	-1.70640600
H	4.30180500	3.72408100	-1.28478900
H	4.90288800	2.36729600	-2.26988100
H	3.20184900	2.85910500	-2.38400600
S	-0.61097700	-0.92915900	4.39362600
H	-1.83067400	-0.88777200	4.95344700
